# Identification and Classification of Polymyalgia Rheumatica (PMR) and PMR-Like Syndromes Following Immune Checkpoint Inhibitors (ICIs) Therapy: Discussion Points and Grey Areas Emerging from a Systematic Review of Published Literature

**DOI:** 10.3390/medicines7110068

**Published:** 2020-11-03

**Authors:** Ciro Manzo, Marco Isetta, Maria Natale, Alberto Castagna

**Affiliations:** 1 Azienda Sanitaria Locale Napoli 3 sud, Internal and Geriatric Medicine Department, Rheumatologic Outpatient Clinic, Health District No. 59, 80065 Sant’Agnello (Naples), Italy; 2 Library and Knowldege Services, Central and North West London NHS Foundation Trust, London NW1 3AX, UK; marco.isetta@nhs.net; 3 Azienda Sanitaria Locale Napoli 3 sud, Internal and Geriatric Medicine Department, Rheumatologic Outpatient Clinic, Health District No. 58, 80054 Gragnano (Naples), Italy; maria.natale.int@alice.it; 4 Geriatric Medicine Department Azienda Sanitaria Provinciale Catanzaro, Fragility Outpatient Clinic, Casa Della Salute di Chiaravalle Centrale, Chiaravalle, 88100 Catanzaro, Italy; albertocastagna78@gmail.com

**Keywords:** polymyalgia rheumatica, immunotherapy, immune checkpoint inhibitors, polymyalgia rheumatica-like syndromes, immune-related adverse events, adverse drug reaction, pharmacovigilance, diagnostic and classification criteria, anticancer therapeutics

## Abstract

**Background**: Polymyalgia Rheumatica (PMR) is one of the most frequent rheumatologic immune-related adverse effects (IRAEs) in cancer patients following therapy with immune checkpoint inhibitors (ICIs). Atypical findings in many patients often lead to diagnosing PMR-like syndromes. **Materials and methods**: The aim of our research was to review reported diagnoses of PMR and PMR-like syndromes following ICIs therapy, and assess whether they can be redefined as adverse drug reaction (ADR). In line with PRISMA guidelines, we carried out a systematic search on three main bibliographic databases, based on a combination of subject headings and free text. We included all studies and case-reports published after 2011 (when FDA approved the use of the first ICI) describing the association of PMR or PMR-like syndromes with all types of ICIs therapy. We excluded reviews, conference abstracts, comments, secondary articles, and non-English language studies. **Results**: We reviewed data from seven studies and eight case-reports, involving a total of 54 patients. Limitations included: the small size of all studies; only one retrospective study used validated criteria for PMR; most reports assessed IRAEs by clinical judgment only and did not seek validation through assessment scales. To date, it remains a conundrum whether IRAEs-PMR is identical to the idiopathic form of the disease, or whether it should be considered a subset of the disease or a new entity. **Conclusions:** Our review indicates that the relationship between PMR and ICIs therapy is yet to be clearly understood and defined and that future research should remedy the current limits in study design.

## 1. Introduction

Polymyalgia rheumatica (PMR) is estimated to be older adults’ most common inflammatory rheumatic disease. Worldwide, its incidence increases until the age of 90, with a peak around the age of 75 [1,2,3,4,5,6]. The onset of PMR in a centenarian man has been reported [7]. 

Typical in PMR patients is a sudden-onset bilateral pain in shoulder and pelvic girdles, sometimes associated with neck aching and morning stiffness lasting more than 45 min. Patients usually complain of significant restrictions in self-care activities of daily living (ADL). Additional symptoms such as fever, general discomfort, fatigue, loss of appetite, and loss of weight can be present in some patients [8,9,10,11]. At present, no specific laboratory tests are available. Inflammatory markers such as erythrocyte sedimentation rate (ESR) and C-reactive protein (CRP) concentrations are usually raised at the time of diagnosis, but the diagnosis of PMR is possible even if ESR and CRP are not increased [12,13]. 

There are several PMR-like diseases, and differential diagnosis is not always easy. Indeed, some patients diagnosed at first with PMR may be reclassified as having a different disease at follow-up [8,9]; and some patients with PMR-mimicking diseases can have a fast (but transitory) response to systemic glucocorticosteroids (GCs).

Shoulder and hip ultrasound (US) examinations can help differential diagnosis, as proposed by the 2012 EULAR/ACR classification criteria [14]. It is worth mentioning that these criteria were designed to discriminate patients with PMR from other mimics of PMR and are not meant for diagnostic purposes. On the other hand, several diagnostic measures have been proposed since Bird’s 1979 criteria, each one with different sensitivity and specificity [15]. Diagnostic or classification criteria should always be applied to avoid defaulting to PMR as a kind of “magic cauldron” in which to put every disease involving long-lasting pain localized to scapular and pelvic girdles and which responds to GCs [11]. 

Since 2011, when the Food and Drug Administration (FDA) approved the use of Ipilimumab, a fully human monoclonal antibody against cytotoxic-T-lymphocyte antigen-4 (CTLA4), for patients with metastatic melanoma, immune checkpoint inhibitors (ICIs) therapy has been recommended for an increasing variety of cancers, both in the metastatic and adjuvant settings. Our immune system has some regulatory receptors (named “checkpoints”) maintaining the balance between T cell lymphocyte activation and inhibition. CTLA-4, programmed death protein-1 (PD-1), and programmed death ligand-1 (PD-L1) are among the best studied checkpoints. ICIs reduce the suppression of effector T cells, mainly CD8+, with consequent up-regulation of tumor-specific immune responses [16,17,18,19,20].

Unfortunately, this same action mechanism can trigger immune-related adverse events (IRAEs), which can affect multiple organ systems; this risk is higher when two ICIs are used in combination [21,22,23,24,25]. Triggered by the growing use of ICIs, an increasingly wide range of rheumatologic IRAEs have been described. A recent pharmacovigilance study observed that the risk of developing PMR is five times higher in cancer patients treated with ICIs compared with patients on other treatments [26]. Moreover, ICIs therapy can cause the flaring up of prior autoimmune rheumatic diseases, PMR among these [27]. 

Atypical findings following ICIs therapy are reported in many patients, leading to diagnoses of PMR-like syndromes, as such patients do not meet standard classification or diagnostic criteria for PMR. A question worth exploring is whether PMR-like findings following ICIs therapy represent a subset of disease or a new clinical entity. 

Furthermore, onset of PMR or PMR-like syndromes can happen many months after the beginning of ICIs therapy. Are they to be considered as an adverse drug reaction (ADR)? Or is their appearance just a coincidence? 

**AIM:** (1) To evaluate how PMR and PMR-like syndromes are identified and classified following ICIs therapy; (2) To assess whether they can be considered as ADR. 

## 2. Materials and Methods 

We conducted a systematic review based on Preferred Reporting Items for Systematic Reviews and Meta-Analysis (PRISMA) guidelines [28]. There is no review protocol, and this systematic review has no registration number. 

### 2.1. Search Strategy 

One of the authors of this study (Isetta, M) carried out a comprehensive literature search in three main bibliographic databases: MEDLINE (OVID interface), EMBASE, and COCHRANE. The following main search terms were used: polymyalgia rheumatica, immune checkpoint blockade, rheumatic syndromes, checkpoint inhibitors therapy, polymyalgia rheumatica-like syndromes, immunotherapy, checkpoint inhibitor-associated polymyalgia rheumatica, anti-PD1, anti-PDL1, anti-PD1 antibody, anti-CTLA4, CTLA4 antibody, and anti-programmed death 1 monoclonal antibody. Searches were carried out on 2 March, 2020. After de-duplication, all retrieved studies were examined. In accordance with the PRISMA 2009 checklist, the full search strategy for one database (MEDLINE) is detailed in the Supplementary Materials (File S1).

### 2.2. Inclusion Criteria

This review included all studies and case-reports published after 2011 (when FDA approved the use of the first ICI) describing the association of PMR or PMR-like syndromes with all types of ICIs therapy. 

### 2.3. Exclusion Criteria

Reviews, conference abstracts, comments, and secondary articles were excluded. Each review’s reference list was scanned for additional publications meeting this study’s inclusion criteria. When papers reported data partially presented in previous articles, we referred to the most recent published data. 

The presence of the giant cell arteritis (GCA) was an additional exclusion criterion, unless all data concerning GCA were clearly distinct from PMR findings. GCA is a large-vessel vasculitis closely linked to PMR: 40–60% of GCA patients show signs of PMR, whereas 10–16% of PMR patients show signs of GCA. It is common knowledge that the association of GCA with PMR has several consequences: i.e., higher dosages of glucocorticoids, more frequent association with cancer, more frequent vascular and ocular complications, and more severe prognosis [29,30].

Finally, non-English language studies with no English abstract were excluded.

### 2.4. Data Extraction

All article titles identified were screened by a single reviewer (Isetta, M) against the inclusion and exclusion criteria. Two of the authors of this study (Manzo, C and Natale, M) independently reviewed the titles and abstracts of all identified records. After reviewing the abstracts, data comparisons were conducted to ensure completeness and reliability. Full-text versions of potentially relevant papers identified in the initial screening were sourced. Reasons for exclusion were recorded. Differing decisions were resolved by consensus. 

Data on study design, source of information, and participant characteristics were independently extracted by the same reviewers, using a standardized ad hoc form (File S2). The primary outcome of interest was the total number of patients who developed PMR or a PMR-like syndrome following ICIs therapy. Other data were collected: diagnostic or classification criteria used to diagnose PMR; its temporal relationship with ICI; study design; and if and how PMR and PMR-like syndromes were evaluated as an adverse drug reaction (ADR). Meta-data from each study, such as lead author name and publication year, were also listed. 

### 2.5. Quality and Bias Risk Assessment

A subjective assessment of the included studies’ methodological quality was performed by all the authors using the Newcastle-Ottawa Scale (NOS), a quality assessment tool for non-randomized studies, endorsed for use in systematic reviews of non-randomized trials by the Cochrane Collaboration [31,32]. NOS uses a “star system” based on three major criteria: study groups’ selection (0–4 stars, or 0–5 stars for cross-sectional studies); comparability of the groups according to key and additional factors (0–2 stars); and determination of the outcome of interest or exposure (0–3 stars). A total score of 3 or less was considered poor; 4–6 was considered moderate; and 7–10 was considered high quality. Studies scoring 3 or less were excluded from our review. The authors settled all disagreements through discussions and by consensus.

## 3. Results

### 3.1. Description of Included Studies

**Figure 1:**
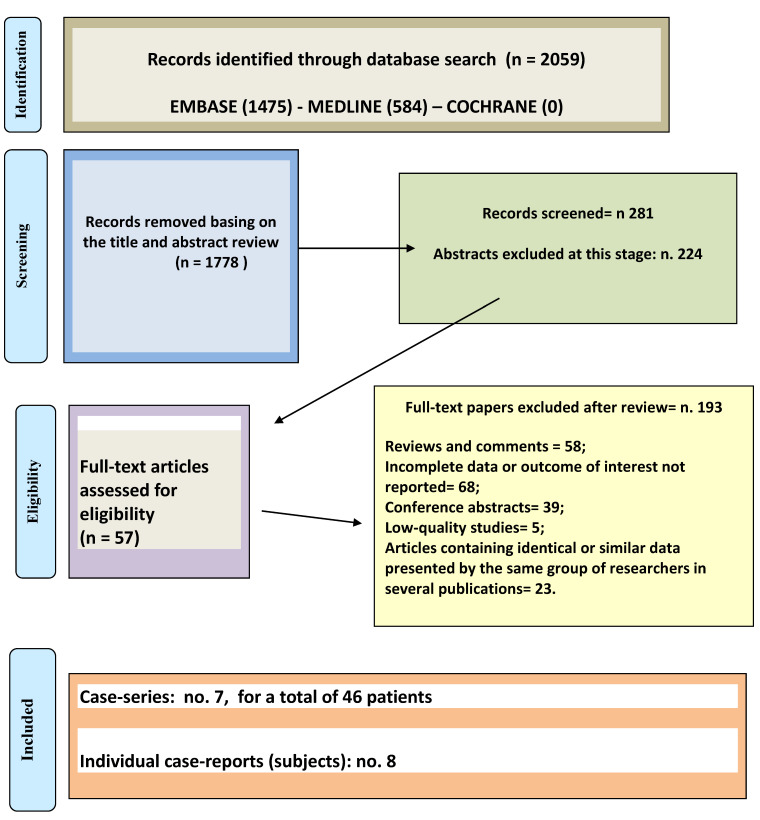
PRISMA Flowchart.

An overview of the study identification process is reported in Figure 1. The initial search yielded 2059 papers, of which 1778 articles were excluded based on title and abstract screening. A total of 281 articles underwent a full-length review; 207 full-text articles were assessed for eligibility; 193 articles were excluded (reviews and comments = 58; conference abstracts = 39; papers containing similar or identical data presented by the same group of researchers in several articles = 23; studies having poor quality = 5; no outcome of interest = 68). Finally, data were extracted from seven case-series and eight case-reports, concerning a total of 54 patients. 

### 3.2. Case-Reports

In Table 1**,** we listed the main characteristics of the eight case reports included in this review. 

### 3.3. Case-Series

In Table 2, we listed the main characteristics of the case-series included in our review. 

### 3.4. Risk of Bias Assessment in Analysis of the Included Studies

As stated, quality assessment of the included studies was performed using the NOS. In line with this scale, three major criteria were evaluated: (1) study groups’ selection; (2) comparability of the groups according to key and additional factors; and (3) determination of the outcome of interest or exposure. 

Table 3 lists the scores. Most studies were given a low score in the study group’s selection because no diagnostic or classification criteria were referenced, and in many studies PMR diagnosis was made by a managing non-rheumatologist. Furthermore, no scales or algorithms were used in evaluating PMR or PMR-like syndromes as ADR. The importance of these points is discussed in the specific section. 

All the included studies failed to recruit a comparator group, and used general population data as reference. Therefore, comparability scores were low, since lack of comparison groups limits the generalizability of the presented results. 

### 3.5. ICIs in Patients with Preexisting PMR 

Our literature search showed that the use of ICIs in patients with preexisting PMR has rarely been described: only four case reports were identified where ICI therapy caused a PMR flare and increase of GC dosages, but this event had no impact on the management of the oncological disease and associated immunotherapy. 

## 4. Discussion

Calabrese et al. first reported instances of PMR following ICIs therapy based on their clinical experience with 15 patients assessed for rheumatologic IRAEs, PMR among these. All cases required GCs, and temporary or permanent discontinuation of ICIs therapy in all but five patients [46]. This same group of researchers expanded their case-series into successive studies, including the one listed in Table 2. 

When our literature search was performed, only 54 patients affected by PMR (or PMR-like syndromes) following ICIs therapy were reported in published literature. However, as the use of ICIs continues to expand, it is highly probable that this group of patients will increase over time. 

The first question is whether these cases of IRAE-PMR represent a disorder identical to the idiopathic form of the disease or a new disease entity instead.

PMR etio-pathogenesis is still debated [47]. The absence of any highly specific diagnostic test is a significant diagnostic limit. As known, since Bird’s criteria published in 1979, different diagnostic and classification criteria have been proposed, with different specificity and sensitivity. Despite this, the risk that PMR could be used as an umbrella label to put every “glucocorticoid-responsive syndrome of shoulder and pelvic girdle pain and stiffness” under is always just around the corner [48,49]. Therefore, it is important to point out that in many IRAEs-PMR reports, no diagnostic or classification criteria were specified, most of the studies were retrospective, and in many studies PMR diagnosis was made by a managing non-rheumatologist. 

Calabrese et al. published in 2019 a retrospective study with data from 49 patients enrolled via two sources: 20 patients from three collaborative centers (group 1), and 29 cases found by systematic review (group 2). For the first time, EULAR/ACR classification criteria were applied; 25% of reported cases supplied insufficient data to enable the application of these criteria (10% among the 20 patients in group 1; 38% among the 29 patients in group 2). Confounding factors were present in 75% of the remaining cases. Namely, positive serology for rheumatoid arthritis, or more severe manifestations than generally present in patients with “idiopathic” PMR and that required a higher initial dose of GCs (possibly because some patients might have GCs started by their oncologist prior to referral to a rheumatologist). Among the 20 patients in group 1, seven had normal acute phase reactants at the time of PMR diagnosis. 

Abnormal CRP and/or ESR are required criteria according to the 2012 EULAR/ACR classification. However, 7 (1.52%) out of the sample of 460 PMR patients were described with normal values of both ESR and CRP concentrations at the time of diagnosis in a 2019 case-series. In these seven patients, all PMR-mimicking diseases were excluded during follow-ups lasting from 29 to 120 months [13]. In other words, normal values of acute phase reactants do not exclude a diagnosis of PMR. To date, whether such patients have an idiopathic PMR or have something that is PMR-like as a result of therapy with ICIs is still a matter of discussion [50]. 

In a single-center cohort of 61 patients with IRAEs, Richter et al. found one case of PMR flare and three cases of new-onset PMR-like syndrome. They speculated that some cases of inflammatory arthritis induced by ICIs may represent previously undiagnosed rheumatic disease rather than de novo IRAEs. No diagnostic or classification criteria were specified in this retrospective study. As in Calabrese’s study, in one patient the dosage of GCs reached 60 mg/day, an objectively high dosage in “isolated” and “idiopathic” PMR [41]. 

With the exception of Calabrese’s study, all our search findings showed that diagnosis of PMR was made according to clinical judgment, without specifying which diagnostic or classification criteria were used. Based on this finding, how did one diagnose a PMR-like syndrome if the boundaries of “idiopathic” PMR had not been defined? Confusingly, all patients were generically categorized as having inflammatory arthritis in other prospective studies, i.e., Kostine et al.’s account of 524 patients treated with ICIs, Braaeten et al. (60 patients, with an average follow-up of 12 months after ICIs cessation) [51], and Lidar M et al. (14 patients) [52]. 

A second discussion point was: how was PMR following ICIs therapy evaluated as an adverse drug reaction (ADR)? According to our literature search, only clinical judgment was used in all case reports and case-series, without the use of scales or algorithms. Algorithms typically use a set of specific ‘yes/no’ questions on ‘features’ of a drug-event pair that have associated scores for calculating a potential cause–effect relationship. Within the field of pharmacovigilance, clinical judgment is often inferior to more structured methods of decision making that use simple algorithms [53]. A number of algorithms have been proposed and some of these have been validated, but none of these have yet been accepted as gold standard [54]. One commonly used algorithm is the ADR Probability Scale developed in 1981 by Naranjo and colleagues to standardize causality assessments [55]. This scale classifies the probability that an adverse event is related to drug therapy. Naranjo’s scale is based on a list of 10 weighted questions which examine factors such as the temporal association of drug administration and event occurrence, alternative causes if any, drug levels, and previous patient experience with the same drug. The key advantages of this scale are its simplicity of use and clarity, and a significant increase in inter- and intra-rater agreement compared with standard clinical examination alone. The sums of the scores ranged from −4 to +13, and was interpreted to reflect the strength of the probability that a drug has caused an ADR rather than the complication being a manifestation of the disease. A score of greater than nine is empirically defined as “definitely” having caused the ADR; a score between five and eight indicates that the drug “probably” caused the ADR; scores between one and four indicate that the ADR was “possibly” caused by the drug; and a score of less than one indicates a “doubtful” association with the drug. When, using reported data, we were able to apply the Naranjo scale to patients described in all our search findings, patients’ scale score were almost never higher than four. A significant number of patients improved without ICI discontinuation, and there was no flare when ICI was re-introduced. It should be also pointed out that PMR/PMR-like syndromes could occur several months after starting ICIs therapy, and in some cases even years after treatment discontinuation. This is in part due to their pharmacodynamic properties [56,57]. 

## 5. Conclusions

Some discussion points and grey areas emerged from our review, namely:

(1) several studies were retrospective and not randomized, and in some cases, the diagnosis of PMR was made in non-rheumatologic settings;

(2) the lack of validated scales for ADR assessment was identified as another key critical point;

(3) whether patients had a real PMR or rather something that was PMR-like is still a matter of discussion. How did one diagnose a PMR-like syndrome if the boundaries of “typical” PMR had not been defined in the vast majority of published reports? In some cases, those that were defined as PMR-like syndromes could be new distinct entities.

According to our review, the relationship between PMR and ICIs therapy is not well defined. We hope that multicenter and prospective future research studies will be designed where, for example, validated scales for ADR assessment and diagnostic and/or classification criteria for PMR are utilized and reported on.

## Figures and Tables

**Table 1:** Immune-related adverse effects-Polymyalgia Rheumatica (IRAEs-PMR) case-reports included in our review.

1st Author and Year	ICI	PMR-Onset	PMR Diagnosis	GCs	ICI Stopped	W.a.r.
Bernier, 2017 [33]	nivolumab	after 13 cycles	n.c.	Yes	Yes	No
Chan, 2019 [34]	atezolizumab	after 6 months	n.c.	Yes	No	n.e.
Imay, 2019 [35]	nivolumab	after 12 cycles	clear	Yes	Yes	n.e.
Garel, 2017 [36]	pembrolizumab	after 1 day	clear	Yes	Yes	No
Garel, 2017 [36]	pembrolizumab	after 3 cycles	clear	Yes	No	n.e.
Iskandar, 2019 [37]	pembrolizumab	n.c.	n.c.	Yes	No	n.e.
Nakamagoe, 2017 [38]	nivolumab	n.c.	n.c.	Yes	Yes	n.e.
Maniu, 2016 [39]	ipilimumab	n.c.	n.c.	Yes	Yes	n.e.

ICI = immune checkpoint inhibitor; PMR = polymyalgia rheumatica; GCs = glucocorticoids; W.a.r. = worsening after reintroduction; n.c. = not clear; n.e. = not evaluable.

**Table 2:** IRAEs-PMR case-series and clinical studies included in our review.

1st Author, Year	Study	PMR(No.)	Onset (Median)	Diagnosis	GCs	ICI Stopped	W.a.r.
Calabrese, 2019 [40]	retrospective	20	12 week	ACR/EULAR	7.5–60	n.c.	n.r.
Richter, 2019 [41]	retrospective	4 *	4–8 weeks	n.r.	15–60	in a minority	n.r.
Kostine, 2018 [42]	observational	11		n.c	7.5–60	Yes	n.c.
Belkhir, 2017 [43]	retrospective	4			n.c.	yes	n.c.
Kuswanto, 2018 [44]	retrospective	4	n.c.	n.c.	yes	Yes	n.r.
Le Burel, 2017 [21]	retrospective	1	n.c.	n.r.	yes	Not	n.c.
Mitchell, 2018 [45]	retrospective	2 **	n.c.	n.r.	yes	Not	n.c.

* PMR flare in one patient. ** PMR flare in two patients, GCs = glucocorticoids; ACR/EULAR = American College of Rheumatology/European League against rheumatism; ICI = immune checkpoint inhibitor; W.a.r. = worsening after reintroduction; n.c. = not clear; n.r. = not reported.

**Table 3:** Quality assessment of the included studies.

First Author	Selection	Comparability	Outcome
Calabrese	3	1	2
Richter	2	1	2
Kostine	2	1	2
Belkhir	2	1	2
Kuswanto	2	1	2
Le Burel	1	1	2
Mitchell	1	1	2

## Supplementary Materials

The following are available online at https://www.mdpi.com/2305-6320/7/11/68/s1, File S1: PMR MEDLINE 2011-2020, File S2: Standardized ad hoc developed form used in this systematic review.

## References

[1] Raheel S., Shbeeb I., Crowson C.A., Matteson E.L. (2017). Epidemiology of polymyalgia rheumatica 2000–2014 and examination of incidence and survival trends over 45 years: A population-based study. Arthritis Care Res. (Hoboken).

[2] Cimmino M.A., Zaccaria A. (2000). Epidemiology of polymyalgia rheumatica. Clin. Exp. Rheumatol..

[3] Manzo C. (2019). Incidence and prevalence of polymyalgia rheumatica (PMR): The importance of the epidemiological context. The Italian case. Med. Sci..

[4] Gonzales-Gay M.A., Vazquez-Rodriguez T.R., Lopez-Diaz M.J., Miranda-Filloy J.A., Gonzales-Juanatey C., Martin J., Llorca J. (2009). Epidemiology of giant cell arteritis and polymyalgia rheumatica. Arthritis Rheum..

[5] Partington R.J., Muller S., Helliwell T., Mallen C.D., Abdul Sultan A. (2018). Incidence, Prevalence and Treatment Burden of Polymyalgia Rheumatica in the UK Over Two Decades: A Population-Based Study. Ann. Rheum. Dis..

[6] Sobrero A., Manzo C., Stimamiglio A. (2018). The role of general practictioner and out-of-hospital public rheumatologist in the diagnosis and follow-up of the patient with polymyalgia rheumatica. Reumatismo.

[7] Manzo C. (2018). Polymyalgia rheumatica (PMR) with normal values of both erythrocyte sedimentation rate (ESR) and C-reactive protein (CRP) concentration at the time of diagnosis in a centenarian man: A case report. Diseases.

[8] Gonzalez-Gay M.A., Matteson E.L., Castaneda S. (2017). Polymyalgia rheumatica. Lancet.

[9] Matteson E.L., Dejaco C. (2017). Polymyalgia rheumatica. Ann. Intern. Med..

[10] Milchert M., Brzosko M. (2017). Diagnosis of polymyalgia rheumatica usually means a favourable outcome for your patient. Indian J. Med. Res..

[11] Manzo C., Camellino D. (2017). La polimialgia reumatica: Difficoltà diagnostiche e terapeutiche per una malattia apparentemente “banale”. Recenti Progress. Med..

[12] Manzo C., Milchert M. (2018). Polymyalgia rheumatica with normal values of both erythrocyte sedimentation rate and C-reactive protein concentration at the time of diagnosis: A four-point guidance. Reumatologia.

[13] Manzo C., Milchert M., Natale M., Brzosko M. (2019). Polymyalgia rheumatica with normal values of both erythrocyte sedimentation rate and C-reactive protein concentration at the time of diagnosis. Rheumatology (Oxford).

[14] Dasgupta B., Cimmino M.A., Kremers H.M., Schmidt A., Schirmer M., Salvarani C., Bachta A., Dejaco C., Duftner C., Slott Jensen H. (2012). 2012 provisional classification criteria for polymyalgia rheumatica: A European League Against Rheumatism/American College of Rheumatology collaborative initiative. Arthritis Rheum..

[15] Bird H.A., Leeb B.F., Montecucco C.M., Misiuniene N., Nesher G., Pai S., Pease C., Rovensky J., Rozman B. (2005). A comparison of sensitivity of diagnostic criteria for polymyalgia rheumatica. Ann. Rheum. Dis..

[16] Graziani G., Tentori L., Navarra P. (2012). Ipilimumab: A novel immunostimulatory monoclonal antibody for the treatment of cancer. Pharmacol. Res..

[17] Hosseini A., Gharibi T., Marofi F., Babaloo Z., Baradaran B. (2020). CTLA-4: From mechanism to autoimmune therapy. Int. Immunopharmacol..

[18] Sharpe A.H. (2017). Introduction to checkpoint inhibitors and cancer immunotherapy. Immunol. Rev..

[19] Timeline of Progress in Immunotherapy–Cancer Research Institute. https://www.canceresearch.org/immunotherapy/timeline-of-progress.

[20] Sharma P., Allison J.P. (2015). The future of immune checkpoint therapy. Science.

[21] Le Burel S., Champiat S., Mateus C., Marabelle A., Michot J.M., Robert C. (2017). Prevalence of immune-related systemic adverse events in patients treated with anti-Programmed cell Death 1/anti-Programmed cell Death-Ligand 1 agents: A single-centre pharmacovigilance database analysis. Eur. J. Cancer.

[22] Schoenfeld S.R., Aronow M.E., Leaf R.K., Dougan M., Reynolds K.L. (2020). Diagnosis and Management of Rare Immune-Related Adverse Events. Oncologist.

[23] Postow M.A., Sidlow R., Hellmann M.D. (2018). Immune-related adverse events associated with immune checkpoint blockade. N. Engl. J. Med..

[24] Kumar V., Chaudhary N., Garg M., Floudas C.A., Soni P., Chandra A.B. (2017). Current diagnosis and management of immune related adverse events (irAEs) induced by immune checkpoint inhibitory therapy. Front. Pharmacol..

[25] Kostine M., Rouxel L., Barnetche T., Veillon R., Martin F., Dutriaux C., Dousset L., Pham-Ledard A., Prey S., Beylot-Barry M. (2018). Rheumatic disorders associated with immune checkpoint inhibitors in patients with cancer-clinical aspects and relationship with tumour response: A single-centre prospective cohort study. Ann. Rheum. Dis..

[26] Salem J.-E., Manouchehri A., Moey M., Lebrun-Vignes B., Bastarache L., Pariente A., Gobert A., Spano J.P., Balko J.M., Bonaca M.P. (2018). Cardiovascular toxicities associated with immune checkpoint inhibitors: An observational, retrospective, pharmacovigilance study. Lancet Oncol..

[27] Gediz F., Kobak S. (2019). Immune Checkpoint Inhibitors-related Rheumatic Diseases: What Rheumatologist Shoul Know?. Curr. Rheumatol. Rev..

[28] Moher D., Liberati A., Tetzlaff J., Altman D.G., The PRISMA Group (2009). Preferred Reporting Items for Systematic Reviews and Meta-Analyses: The PRISMA Statement. PLoS Med..

[29] Salvarani C., Cantini F., Hunder G.G. (2008). Polymyalgia rheumatica and giant-cell arteritis. Lancet.

[30] Dejaco C., Brouwer E., Mason J.C., Buttgereit F., Matteson E.L., Dasgupta B. (2017). Giant cell arteritis and polymyalgia rheumatica: Current challenges and opportunities. Nat. Rev. Rheumatol..

[31] Stang A. (2010). Critical evaluation of the Newcastle-Ottawa scale for the assessment of the quality of non-randomized studies in meta-analyses. Eur. J. Epidemiol..

[32] Higgins J.P.T., Green S. Cochrane Handbook for Systematic Reviews of Interventions. http://handbook.cochrane.org.

[33] Bernier M., Guillaume C., Leon N., Alexandre J., Hamel-Senecal L., Chretien B., Lecaignec F., Humbert X., Fedrizzi S., Madeleine J. (2017). Nivolumab Causing a Polymyalgia Rheumatica in a Patient with a Squamous Non-Small Cell Lung Cancer. J Immunother..

[34] Chan K.K., Bass A.R. (2019). Checkpoint inhibitor-induced polymyalgia rheumatica controlled by cobimetinib, a MEK ½ inhibitor. Ann. Rheum. Dis..

[35] Imai Y., Tanaka M., Fujii R., Uchitani K., Okazaki K. (2019). Effectiveness of a Low-dose Corticosteroid in a Patient with Polymyalgia Rheumatica Associated with Nivolumab Treatment. J. Pharm. Soc. Jpn..

[36] Garel B., Kramkimel N., Trouvin A.P., Frantz C., Dupin N. (2017). Pembrolizumab-induced polymyalgia rheumatica in two patients with metastatic melanoma. Jt. Bone Spine.

[37] Iskandar A., Hwang A., Dasanu C.A. (2019). Polymyalgia rheumatica due to pembrolizumab therapy. J. Oncol. Pharm. Pract..

[38] Nakamagoe K., Moriyama T., Maruyama H., Yokosawa M., Hara T., Tanaka S., Fujimoto M., Tamaoka A. (2017). Polymyalgia rheumatica in a melanoma patient due to nivolumab treatment. J. Cancer Res. Clin. Oncol..

[39] Maniu C., Kobe C., Schlaak M., Mauch C., Eming S.A. (2016). Polymyalgia rheumatic occurring during treatment with ipilimumab. Eur. J. Derm..

[40] Calabrese C., Cappelli L.C., Kostine M., Kirchner E., Braaten T., Calabrese L. (2019). Polymyalgia rheumatica-like syndrome from checkpoint inhibitor therapy: Case series and systematic review of the literature. RMD Open.

[41] Richter M.D., Crowson C., Kottschade L.A., Finnes H.D., Markovic S.N., Thanarajasingam U. (2019). Rheumatic syndromes associated with immune checkpoint inhibitors: A single-center cohort of sixty-one patients. Arthritis Rheumatol..

[42] Kostine M., Finckh A., Bingham C.O., Visser K., Leipe J., Schulze-Koops H., Choy E.H., Benesova C., Radstake T.R.D.J., Cope A.P. (2020). EULAR points to consider for the diagnosis and management of rheumatic immune-related adverse events due to cancer immunotherapy with checkpoint inhibitors. Ann. Rheum. Dis..

[43] Belkhir R., Le Burel S., Dunogeant L., Marabelle A., Hollebecque A., Besse B., Leary A., Voisin A.L., Ponoizeau C., Coutte L. (2017). Rheumatoid arthritris and polymyalgia rheumatica occurring after immune checkpoint inhibitor treatment. Ann. Rheum. Dis..

[44] Kuswanto W.F., MacFarlane L.A., Gedmintas L., Mulloy A., Choueiri T.K., Bermas B.L. (2018). Rheumatologic symptoms in oncologic patients on PD-1 inhibitors. Semin. Arthritis Rheum..

[45] Mitchell E.L., Lau P.K.H., Khoo C., Liew D., Leung J., Liu B., Rischin A., Frauman A.G., Kee D., Smith K. (2018). Rheumatic immune-related adverse events secondary to anti-programmed death-1 antibodies and preliminary analysis on the impact of corticosteroids on anti-tumour response: A case series. Eur. J. Cancer.

[46] Calabrese C., Kirchner E., Kontzias A., Velcheti V., Calabrese L.H. (2017). Rheumatic immune-related adverse events of checkpoint therapy for cancer: Case series of a new nosological entity. RMD Open.

[47] Guggino G., Ferrante A., Macaluso F., Triolo G., Ciccia F. (2018). Pathogenesis of polymyalgia rheumatica. Reumatismo.

[48] Camellino D., Cimmino M.A. (2016). Are the new ACR/EULAR criteria the ultimate answer for polymyalgia rheumatica classification?. J. Rheumatol..

[49] Liew D.F.L., Leung J.L.Y., Liu B., Cebon J., Frauman A.G., Buchanan R.R.C. (2019). Association of good oncological response to therapy with the development of rheumatic immune-related adverse events following PD-1 inhibitor therapy. Int. J. Rheum. Dis..

[50] Manzo C., Milchert M., Natale M., Brzosko M. (2020). Polymyalgia rheumatica with normal inflammatory indices at the time of diagnosis: Can we just move a step forward?. Reumatologia.

[51] Braaten T., Brahmer J.R., Forde P.M., Le D., Lipson E.J., Naidoo J., Schollenberger M., Zheng L., Bingham C.O., Shah A.A. (2020). Immune checkpoint inhibitor-induced inflammatory arthritis persists after immunotherapy cessation. Ann. Rheum. Dis..

[52] Lidar M., Giat E., Garelick D., Horowitz Y., Amital H., Steinberg-Silman Y., Schacther J., Shapira-Frommer R., Markel G. (2018). Rheumatic manifestations among cancer patients treated with immune checkpoint inhibitors. Autoimmun. Rev..

[53] Agbabiaka T.B., Savovic J., Ernst E. (2008). Methods for causality assessment of adverse drug reactions. Drug Saf..

[54] Srinivasan R., Ramya G. (2011). Adverse drug reaction–Causality assessment. Int. J. Res. Pharm. Chem..

[55] Naranjo C.A., Busto U., Sellers E.M., Sandor P., Ruiz I., Robert E.A., Janecek E., Domecq C., Greenblatt D.J. (1981). A method for estimating the probability of adverse drug reaction. Clin. Pharmacol. Ther..

[56] Ernstoff M., Ghandi S., Pandey M., Puzanov I., Grivas P., Montero A., Velcheti V., Turk M.J., Diaz-Montero C.M., Lewis L.D. (2017). Challenges faced when identifying patients for combination immunotherapy. Future Oncol..

[57] Coury M.A., Bell R.B., Patel A.A., Romba M.C., Crittenden M.R., Curti B.D., Urba W.J., Leidner R.S. (2019). Delayed immune-related events (dire) after discontinuation of immunotherapy: Diagnostic hazard of autoimmunity at a distance. J. Immunother. Cancer.

