# Supplementary material for: Identification and Classification of Polymyalgia Rheumatica (PMR) and PMR-Like Syndromes Following Immune Checkpoint Inhibitors (ICIs) Therapy: Discussion Points and Grey Areas Emerging from a Systematic Review of Published Literature
Source: Medicines (Basel). 2020 Nov 3;7(11):68. doi: 10.3390/medicines7110068 (PMC7693468; doi:10.3390/medicines7110068)
Supplement: Supplementary file 1 [file medicines-07-00068-s001.zip › medicines-971817 suppleS2 for final.pdf]

Supplementary Materials: Identification and Classification of Polymyalgia Rheumatica (PMR) and PMR-Like Syndromes Following Immune Checkpoint Inhibitors (ICIs) Therapy: Discussion Points and Grey Areas Emerging from a Systematic Review of Published Literature.

Ciro Manzo, Marco Isetta, Maria Natale and Alberto Castagna

Table 1. General data

First author
Title
Journal
Year
Volume and Page Numbers
Language of Publication
Population
Age
Sex

Table 2. PMR criteria used and study design.

PMR Criteria Used	N.	Study Design	N.
Chuang		Cohort	
Bird		Case control	
Jones		Retrospective	
Nobunaga		Observational	
Healey		Systematic Review	
Hunder		Case report	
2012 EULAR-ACR			
Not referenced			

Table 3. PMR and PMR-like syndromes as an adverse drug reaction (ADR) .

Temporal Relationship with ICIs Therapy :		
PMR evaluated as an ADR:	YES/NO	– If yes, specify how:
PMR-like syndromes evaluated as an ADR:	YES/NO	– If yes, specify how:
