# Supplementary material for: Identification and Classification of Polymyalgia Rheumatica (PMR) and PMR-Like Syndromes Following Immune Checkpoint Inhibitors (ICIs) Therapy: Discussion Points and Grey Areas Emerging from a Systematic Review of Published Literature
Source: Medicines (Basel). 2020 Nov 3;7(11):68. doi: 10.3390/medicines7110068 (PMC7693468; doi:10.3390/medicines7110068)
Supplement: Supplementary file 1 [file medicines-07-00068-s001.zip › PMR MEDLINE 2011-2020.pdf]

Database: Ovid MEDLINE(R) and Epub Ahead of Print, In-Process & Other Non-Indexed Citations, Daily and Versions(R) <1946 to March 2, 2020>

Search Strategy:

----

1 exp Polymyalgia Rheumatica/ (2486)
2 "polymyalgia rheum*".mp. (3224)
3 exp Rheumatic Diseases/ (222822)
4 polymyalg*.mp. (3297)
5 "Polymyalgia rheumatica-like".mp. (19)
6 1 or 2 or 3 or 4 or 5 (223492)
7 exp immunotherapy/ (270445)
8 "immune checkpoint blockad*".mp. (2092)
9 "immune checkpoint inhibitor*".mp. (6145)
10 "checkpoint inhibitor* therap*".mp. (547)
11 "checkpoint inhibit*".mp. (9403)
12 nivolumab.mp. (4497)
13 7 or 8 or 9 or 10 or 11 or 12 (280564)
14 6 and 13 (1846)
15 limit 14 to yr="2011 -Current" (584)

****

<1>

Unique Identifier

30801332

Title

Immune Checkpoint Inhibitor-Associated Polymyalgia Rheumatica/Giant Cell Arteritis Occurring in a Patient After Treatment With Nivolumab. JCR: Journal of Clinical Rheumatology. 2019 Feb 19.

Status

Publisher

Authors

Betrains A; Blockmans DE.

Authors Full Name

Betrains, Albrecht; Blockmans, Daniel Engelbert.

Institution

Betrains, Albrecht. Department of General Internal Medicine University Hospitals Leuven Herestraat 49, 3000 Leuven, Belgium albrecht.betrains@uzleuven.be.

Publication Type

Journal Article.

Year of Publication

2019

<2>

Unique Identifier

28267050

Title

Nivolumab Causing a Polymyalgia Rheumatica in a Patient With a Squamous Non-Small Cell Lung Cancer.

Source

Journal of Immunotherapy. 2017 Mar 06.

Status

Publisher

Authors

Bernier M; Guillaume C; Leon N; Alexandre J; Hamel-Senecal L; Chretien B; Lecaignec F; Humbert X; Fedrizzi S; Madelaine J; Sassier M.

Authors Full Name

Bernier, Marjorie; Guillaume, Cyril; Leon, Nathalie; Alexandre, Joachim; Hamel-Senecal, Lea; Chretien, Basile; Lecaignec, Florian; Humbert, Xavier; Fedrizzi, Sophie; Madelaine, Jeannick; Sassier, Marion.

Institution

Bernier, Marjorie. Departments of *Pharmacology Pain and Palliative Care ++Rheumatology Pneumology, CHU of Caen Department of General Medicine, Medical School, Normandie University, Caen, France.

Abstract

The anti-programmed cell-death-1 antibody, nivolumab, has been recently approved for the treatment of advanced non-small cell lung cancer. Although, today, immune-related adverse effects such as dermatologic, digestive, hepatic, and endocrine toxicities are well-known with immune checkpoint inhibitors, rheumatic diseases are less well described. Herein, we report the case of a patient without a history of arthritis who developed polymyalgia rheumatica after 13 cycles of nivolumab used for the treatment of advanced non-small cell lung cancer. Laboratory evidence of inflammatory syndrome, articular echography, and clinical presentation with classical symptoms and also distal manifestations were suggestive of this chronic inflammatory disorder. Because of a relevant pain, clinicians were forced to suspend immunotherapy. Nevertheless, due to glucocorticoid therapy, the patient's symptoms have decreased progressively. Moreover, nivolumab was reintroduced 8 weeks later, whereas prednisone (10 mg) was continued, without any recurrence symptoms. To conclude, our case suggests that polymyalgia rheumatica might be a very disabling anti-programmed cell-death-1 immune-related adverse effect.

Publication Type

Journal Article.

Year of Publication

2017

<3>

Unique Identifier

29925509

Title

Response to: 'Checkpoint inhibitor-induced polymyalgia rheumatica controlled by cobimetinib, a MEK 1/2 inhibitor' by Chan and Bass.

Source

Annals of the Rheumatic Diseases. 78(7):e71, 2019 07.

VI 1

Status

In-Process

Authors

Kostine M; Dousset L; Schaeverbeke T; FHU-ACRONIM.

Authors Full Name

Kostine, Marie; Dousset, Lea; Schaeverbeke, Thierry; FHU-ACRONIM.
Institution
Kostine, Marie. Department of Rheumatology, Centre Hospitalier Universitaire de Bordeaux, Bordeaux, France.
Dousset, Lea. Department of Dermatology, Centre Hospitalier Universitaire de Bordeaux, Bordeaux, France.
Schaeverbeke, Thierry. Department of Rheumatology, Centre Hospitalier Universitaire de Bordeaux, Bordeaux, France.
Comments
Comment on (CON)
Comment on (CON)
Publication Type
Letter. Comment.
Year of Publication
2019

<4>
Unique Identifier
29760158
Title
Checkpoint inhibitor-induced polymyalgia rheumatica controlled by cobimetinib, a MEK 1/2 inhibitor. Annals of the Rheumatic Diseases. 78(7):e70, 2019 07.

In-Process
Authors
Chan KK; Bass AR.
Authors Full Name
Chan, Karmela Kim; Bass, Anne R.
Institution
Chan, Karmela Kim. Department of Medicine, Division of Rheumatology, Hospital for Special Surgery, New York, New York, USA.
Bass, Anne R. Department of Medicine, Division of Rheumatology, Hospital for Special Surgery, New York, New York, USA.

Comments
  Comment on (CON)
   Comment in (CIN)
Publication Type
  Letter. Comment.
Year of Publication
  2019

<5>
Unique Identifier
  31168414
Title
  Polymyalgia rheumatica-like syndrome from checkpoint inhibitor therapy: case series and systematic review of the literature.
Source
  RMD Open. 5(1):e000906, 2019.
VI 1
Status
  In-Process
Authors
  Calabrese C; Cappelli LC; Kostine M; Kirchner E; Braaten T; Calabrese L.
Author NameID
  Calabrese, Cassandra; ORCID: https://orcid.org/0000-0002-8773-7739
Authors Full Name
  Calabrese, Cassandra; Cappelli, Laura C; Kostine, Marie; Kirchner, Elizabeth; Braaten, Tawnie; Calabrese, Leonard.
Institution
  Calabrese, Cassandra. Rheumatic & Immunologic Disease, Cleveland Clinic, Cleveland, Ohio, USA.
   Cappelli, Laura C. Division of Rheumatology, Johns Hopkins School of Medicine, Baltimore, Maryland, USA.
   Kostine, Marie. Rheumatology Department, Centre Hospitalier Universitaire de Bordeaux, Bordeaux, France.

Kirchner, Elizabeth. Rheumatic & Immunologic Disease, Cleveland Clinic, Cleveland, Ohio, USA.

Braaten, Tawnie. Division of Rheumatology, Johns Hopkins School of Medicine, Baltimore, Maryland, USA.

Calabrese, Leonard. Rheumatic & Immunologic Disease, Cleveland Clinic, Cleveland, Ohio, USA.

Abstract

Objective: To assess whether the polymyalgia rheumatica (PMR)-like syndrome reported as an immune related adverse event (irAE) from checkpoint inhibitor therapy is consistent with the 2012 European League Against Rheumatism (EULAR)/American College of Rheumatology (ACR) provisional criteria for PMR.

Methods: The cases were derived from two sources. Group 1 represents reported cases from three contributing centres. Group 2 was derived from a systematic review of the literature searching for all cases reported as PMR or PMR-like illness associated with checkpoint inhibitor therapy. Cases were assessed for the quality of reporting and then analysed to determine whether they fulfilled the 2012 EULAR/ACR provisional criteria for PMR.

Results: A total of 49 patients were included for analysis. Among the entire group, 37 (75%) were designated 'complete' indicating that they had sufficient data to reliably apply the 2012 EULAR/ACR criteria. 28 (75%) cases fulfilled complete criteria for PMR. A number of cases also demonstrated some clinical features unusual for idiopathic PMR.

Conclusion: This study suggests a high proportion of reported cases of checkpoint inhibitor-related PMR fulfil preliminary criteria for PMR, yet in one quarter clinical details were incomplete making verification problematic. Furthermore, in the absence of a gold standard for the diagnosis of PMR, the relationship of checkpoint inhibitor-related PMR to the idiopathic form remains unclear.

Publication Type

Research Support, N.I.H., Extramural. Journal Article.

Year of Publication

2019

<6>

Unique Identifier

31816084

Title

Frequency and distribution of various rheumatic disorders associated with checkpoint inhibitor therapy. Rheumatology. 58(Supplement_7):vii40-vii48, 2019 Dec 01.

Authors

Abdel-Wahab N; Suarez-Almazor ME.

Authors Full Name

Abdel-Wahab, Noha; Suarez-Almazor, Maria E.

Institution

Abdel-Wahab, Noha. Department of General Internal Medicine, Section of Rheumatology and Clinical Immunology, The University of Texas MD Anderson Cancer Center, Houston, TX, USA.

Abdel-Wahab, Noha. Department of Rheumatology and Rehabilitation, Faculty of Medicine, Assiut University Hospitals, Assiut, Egypt.

Suarez-Almazor, Maria E. Department of General Internal Medicine, Section of Rheumatology and Clinical Immunology, The University of Texas MD Anderson Cancer Center, Houston, TX, USA.

Abstract

Immune checkpoint inhibitors have advanced the treatment paradigm of various cancers, achieving remarkable survival benefits. However, a myriad of immune-related adverse events (irAE) has been recognized in almost every organ system, presumably because of persistent immune system activation. Rheumatic symptoms such as arthralgia or myalgia are very common. More specific irAE are increasingly being reported. The most frequent ones are inflammatory arthritis, polymyalgia-like syndromes, myositis and sicca manifestations. These rheumatic irAE can develop in ~5-10% of patients treated with immune checkpoint inhibitors, although true incidence rates cannot be estimated given the lack of prospective cohort studies, and likely underreporting of rheumatic irAE in oncology trials. In this review, we will provide a summary of the epidemiologic data reported for these rheumatic irAE, until more robust prospective longitudinal studies become available to further define the true incidence rate of rheumatic irAE in patients receiving these novel cancer therapies. Copyright © The Author(s) 2019. Published by Oxford University Press on behalf of the British Society for Rheumatology.

Publication Type

Journal Article.

Year of Publication

2019

<7>

Unique Identifier

31816082

Title

Clinical characteristics of rheumatic syndromes associated with checkpoint inhibitors therapy.

Source

Rheumatology. 58(Supplement_7):vii68-vii74, 2019 Dec 01.

VI 1

Status

Authors

Kostine M; Truchetet ME; Schaeverbeke T.

Authors Full Name

Kostine, Marie; Truchetet, Marie-Elise; Schaeverbeke, Thierry.

Institution

Kostine, Marie. Department of Rheumatology, Bordeaux University Hospital, Bordeaux, France.

Truchetet, Marie-Elise. Department of Rheumatology, Bordeaux University Hospital, Bordeaux, France.

Schaeverbeke, Thierry. Department of Rheumatology, Bordeaux University Hospital, Bordeaux, France.

Abstract

Compared with conventional cancer therapies, the spectrum of toxicities observed with checkpoint inhibitors is unique and can affect any organ system. Arthralgia and myalgia were by far the most commonly reported rheumatic immune-related adverse events in clinical trials, and there is now a growing number of case series and reports describing clinical features of de novo rheumatic immune-related adverse events, which will be the focus of this review. Some patients develop genuine classic rheumatic and musculoskeletal diseases, but a number of rheumatic immune-related adverse events mimic rheumatic and musculoskeletal diseases with atypical features, mainly polymyalgia rheumatica, rheumatoid arthritis and myositis, as well as several systemic conditions, including sicca syndrome, vasculitis, sarcoidosis, systemic sclerosis and lupus. Copyright © The Author(s) 2019. Published by Oxford University Press on behalf of the British Society for Rheumatology.

Publication Type

Journal Article.
Year of Publication
2019

<8>
Unique Identifier
31816079
Title
Management of rheumatic complications of immune checkpoint inhibitor therapy - an oncological perspective.
Source
Rheumatology. 58(Supplement_7):vii29-vii39, 2019 Dec 01.
VI 1
Status
Authors
Steven NM; Fisher BA.
Authors Full Name
Steven, Neil M; Fisher, Benjamin A.
Institution
Steven, Neil M. Institute of Immunology and Immunotherapy, Birmingham, UK.
Steven, Neil M. Cancer Centre, University Hospital Birmingham, Birmingham, UK.
Fisher, Benjamin A. Institute of Inflammation and Ageing, University of Birmingham, Birmingham, UK.
Fisher, Benjamin A. National Institute of Health Research Birmingham Biomedical Research Centre and Department of Rheumatology, University Hospital Birmingham, Birmingham, UK.
Abstract
Immune checkpoint inhibitors (CPIs) are an effective treatment for many cancers but cause diverse immune-related adverse events (IrAEs). Rheumatological IrAEs include arthralgia, arthritis, tenosynovitis, myositis, polymyalgia rheumatica and sicca syndrome. CPI use can unmask RA as well as causing flares of prior autoimmune or connective tissue disease. Oncologists categorize and grade IrAEs using the Common Terminology Criteria for Adverse Events and manage them according to international guidelines. However, rheumatological events are unfamiliar territory: oncologists need to work with rheumatologists to elicit and assess

symptoms, signs, results of imaging and autoantibody testing and to determine the use of steroids and DMARDs. Myositis may overlap with myasthenic crisis and myocarditis and can be life-threatening. Treatment should be offered on balance of risk and benefit, including whether to continue CPI treatment and recognizing the uncertainty over whether glucocorticoids and DMARDs might compromise cancer control. Copyright © The Author(s) 2019. Published by Oxford University Press on behalf of the British Society for Rheumatology.

Publication Type

Journal Article.

Year of Publication

2019

<9>

Unique Identifier

31379056

Title

Is Immune Checkpoint Inhibitor Treatment an Option for Patients With Rheumatic Diseases and Cancer?.

Source

Arthritis & Rheumatology. 71(12):1971-1973, 2019 12.

VI 1

Status

MEDLINE

Authors

Buckley LM; Suarez-Almazor ME.

Author NameID

Buckley, Lenore M; ORCID: https://orcid.org/0000-0001-7001-8036

Suarez-Almazor, Maria E; ORCID: https://orcid.org/0000-0001-5381-5797

Authors Full Name

Buckley, Lenore M; Suarez-Almazor, Maria E.

Institution

Buckley, Lenore M. Yale University School of Medicine, New Haven, Connecticut.

Suarez-Almazor, Maria E. University of Texas MD Anderson Cancer Center, Houston.

Comment on (CON)

Publication Type
  Editorial. Comment.
Year of Publication
  2019

<10>
Unique Identifier
  31111819
Title
  Vaccine Approaches To Protect against Group A Streptococcal Pharyngitis. [Review]
Source
  Microbiology Spectrum. 7(3), 2019 05.
VI 1
Status
  MEDLINE
Authors
  Fischetti VA.
Authors Full Name
  Fischetti, Vincent A.
Institution
  Fischetti, Vincent A. The Rockefeller University, New York, NY 10065.
Abstract
Streptococcal pharyngitis (or strep throat) is a common childhood disease affecting millions of children each year, but it is one of the only childhood diseases for which a vaccine does not exist. While for decades the development of a vaccine has been the center of attention in many laboratories worldwide, with some successes, no corporate development has yet to be initiated. The reason for this probably lies in our inability to conclusively identify the streptococcal molecule or molecules responsible for the heart cross-reactive antibodies observed in the serum of rheumatic fever patients. Without this specific knowledge, any streptococcal vaccine antigen is suspect and thus not the target for a billion-dollar investment, despite the fact that the exact role of cross-reactive antibodies in rheumatic fever is still questionable. This article will describe the development of several approaches to protect against Streptococcus pyogenes infections over the past several decades.

Publication Type

Journal Article. Review.

Year of Publication

2019

<11>

Unique Identifier

31650952

Title

[Management of Rheumatic Adverse Events Related to Immune Checkpoint Inhibitors]. [**Review**] [Chinese]

Source

Chinese Journal of Lung Cancer. 22(10):671-675, 2019 Oct 20.

VI 1

Status

MEDLINE

Authors

Zhou J; Wang Q; Duan L; Si X; Zhang L; Liu X; Li Y; Wang H; Guo X; Zhang W; Zhang L.

Authors Full Name

Zhou, Jiaxin; Wang, Qian; Duan, Lian; Si, Xiaoyan; Zhang, Li; Liu, Xiaowei; Li, Yue; Wang, Hanping; Guo, Xiaoxiao; Zhang, Wen; Zhang, Li.

Institution

Zhou, Jiaxin. Department of Rheumatology, Peking Union Medical College Hospital, Chinese Academy of Medical Sciences, Beijing 100730, China.

Wang, Qian. Department of Rheumatology, Peking Union Medical College Hospital, Chinese Academy of Medical Sciences, Beijing 100730, China.

Duan, Lian. Department of Endocrinology, Peking Union Medical College Hospital, Chinese Academy of Medical Sciences, Beijing 100730, China.

Si, Xiaoyan. Department of Respiratory medicine, Peking Union Medical College Hospital, Chinese Academy of Medical Sciences, Beijing 100730, China.

Zhang, Li. Department of Clinical Laboratory, Peking Union Medical College Hospital, Chinese Academy of Medical Sciences, Beijing 100730, China.

Liu, Xiaowei. Department of Ophthalmology, Peking Union Medical College Hospital, Chinese Academy of Medical Sciences, Beijing 100730, China.
Li, Yue. Department of Gastroenterology, Peking Union Medical College Hospital, Chinese Academy of Medical Sciences, Beijing 100730, China.
Wang, Hanping. Department of Respiratory medicine, Peking Union Medical College Hospital, Chinese Academy of Medical Sciences, Beijing 100730, China.
Guo, Xiaoxiao. Department of Cardiology, Peking Union Medical College Hospital, Chinese Academy of Medical Sciences, Beijing 100730, China.
Zhang, Wen. Department of Rheumatology, Peking Union Medical College Hospital, Chinese Academy of Medical Sciences, Beijing 100730, China.
Zhang, Li. Department of Respiratory medicine, Peking Union Medical College Hospital, Chinese Academy of Medical Sciences, Beijing 100730, China.

Abstract

Immune checkpoint inhibitors (ICIs) have made remarkable breakthroughs in cancer treatment. However, the widely use of ICIs is associated with a spectrum of immune-related adverse events (irAEs). These adverse events can affect any organ system. In this article, we have made a systemic review about the clinical characteristics of rheumatic irAEs, and also summarized irAEs in patients with pre-exsiting rheumatic disease. We also focus on the management of rheumatic irAEs.

Other Abstract

Publisher: : : , (immune checkpoint inhibitors, ICIs) ICIs , (immune-related adverse events, irAEs) ,irAEs ICIs , ICIs , : ; ; ; .; Language: Chinese

Publication Type

Journal Article. Review.

Year of Publication

2019

<12>

Unique Identifier

31477876

Title

Vaccination guidance updated.

Source

Nature Reviews Rheumatology. 15(10):574, 2019 10.

VI 1

Status

MEDLINE

Authors

Onuora S.

Authors Full Name

Onuora, Sarah.

Institution

Onuora, Sarah. Nature Reviews Rheumatology,. nrrheum@nature.com.

Comments

Comment on (CON)

Publication Type

Journal Article. Comment.

Year of Publication

2019

<13>

Unique Identifier

31233293

Title

Reply.

Source

Arthritis & Rheumatology. 71(11):1969-1970, 2019 11.

VI 1

Status

MEDLINE

Authors

Hinze CH; Foell D.

Author NameID

Hinze, Claas H; ORCID: https://orcid.org/0000-0001-9247-4729

Authors Full Name

Hinze, Claas H; Foell, Dirk.

Institution

Hinze, Claas H. University Hospital Munster, Munster, Germany.

Foell, Dirk. University Hospital Munster, Munster, Germany.

Comments

Comment on (CON)

Comment on (CON)

Publication Type

Letter. Comment.

Year of Publication

2019

<14>

Unique Identifier

31233280

Title

Questions Regarding the Relationship Between Serum S100A8/A9 and S100A12 Levels, and the Maintenance of Clinically Inactive Disease in Juvenile Idiopathic Arthritis: Comment on the Article by Hinze et al.

Source

Arthritis & Rheumatology. 71(11):1968-1969, 2019 11.

VI 1

Status

MEDLINE

Authors

Rochette E; Pereira B; Merlin E.

Author NameID

Rochette, Emmanuelle; ORCID: https://orcid.org/0000-0001-5180-9916

Authors Full Name

Rochette, Emmanuelle; Pereira, Bruno; Merlin, Etienne.

Institution

Rochette, Emmanuelle. Centre Hospitalier Universitaire de Clermont-Ferrand, Clermont-Ferrand, France.

Pereira, Bruno. Centre Hospitalier Universitaire de Clermont-Ferrand, Clermont-Ferrand, France.
Merlin, Etienne. Centre Hospitalier Universitaire de Clermont-Ferrand, Clermont-Ferrand, France.
Comments
Comment on (CON)
Comment in (CIN)
Publication Type
Letter. Comment.
Year of Publication
2019

<15>
Unique Identifier
30589082
Title
Dendritic cells, T cells and their interaction in rheumatoid arthritis. [Review]
Source
Clinical & Experimental Immunology. 196(1):12-27, 2019 04.
VI 1
Status
MEDLINE
Authors
Wehr P; Purvis H; Law SC; Thomas R.
Author NameID
Thomas, R; ORCID: https://orcid.org/0000-0002-0518-8386
Authors Full Name
Wehr, P; Purvis, H; Law, S-C; Thomas, R.
Institution
Wehr, P. The University of Queensland Diamantina Institute, Translational Research Institute, Princess Alexandra Hospital, Brisbane, Australia.

Purvis, H. King's College London, Academic Department of Rheumatology, Centre for Inflammation Biology and Cancer Immunology, School of Immunology and Microbial Sciences, Faculty of Life Sciences and Medicine, London, UK.
Law, S-C. The University of Queensland Diamantina Institute, Translational Research Institute, Princess Alexandra Hospital, Brisbane, Australia.
Thomas, R. The University of Queensland Diamantina Institute, Translational Research Institute, Princess Alexandra Hospital, Brisbane, Australia.
Abstract
Dendritic cells (DCs) are the key professional antigen-presenting cells which bridge innate and adaptive immune responses, inducing the priming and differentiation of naive to effector CD4+ T cells, the cross-priming of CD8+ T cells and the promotion of B cell antibody responses. DCs also play a critical role in the maintenance of immune homeostasis and tolerance. DC-T cell interactions underpin the generation of an autoimmune response in rheumatoid arthritis (RA). Here we describe the function of DCs and review evidence for DC and T cell involvement in RA pathogenesis, in particular through the presentation of self-peptide by DCs that triggers differentiation and activation of autoreactive T cells. Finally, we discuss the emerging field of targeting the DC-T cell interaction for antigen-specific immunotherapy of RA. Copyright © 2018 British Society for Immunology.
Publication Type
Journal Article. Research Support, Non-U.S. Gov't. Review.
Year of Publication
2019

<16>
Unique Identifier
30923347
Title
NGF vaccine reduces pain.
Source
Nature Reviews Rheumatology. 15(5):251, 2019 05.
VI 1
Status
MEDLINE

Authors

Bernard NJ.

Authors Full Name

Bernard, Nicholas J.

Institution

Bernard, Nicholas J. Nature Reviews Rheumatology,. nrrheum@nature.com.

Comments

Comment on (CON)

Publication Type

Journal Article. Comment.

Year of Publication

2019

<17>

Unique Identifier

31492169

Title

Dynamic profiles, biodistribution and integration evaluation after intramuscular/intravenous delivery of a novel therapeutic DNA vaccine encoding chicken type II collagen for rheumatoid arthritis in vaccinated normal rodent.

Source

Journal of Nanobiotechnology. 17(1):94, 2019 Sep 06.

VI 1

Status

MEDLINE

Authors

Zhao X; Long J; Liang F; Liu N; Sun Y; Xi Y.

Author NameID

Xi, Yongzhi; ORCID: http://orcid.org/0000-0001-9980-9245

Authors Full Name

Zhao, Xiao; Long, Juan; Liang, Fei; Liu, Nan; Sun, Yuying; Xi, Yongzhi.

Institution

Zhao, Xiao. Department of Immunology and National Center for Biomedicine Analysis, the Fifth Medical Center (formerly known as Beijing 307 Hospital), Chinese PLA General Hospital, No. 8, Dongda Ave, Fengtai District, Beijing, 100071, People's Republic of China.

Long, Juan. Department of Immunology and National Center for Biomedicine Analysis, the Fifth Medical Center (formerly known as Beijing 307 Hospital), Chinese PLA General Hospital, No. 8, Dongda Ave, Fengtai District, Beijing, 100071, People's Republic of China.

Liang, Fei. Department of Immunology and National Center for Biomedicine Analysis, the Fifth Medical Center (formerly known as Beijing 307 Hospital), Chinese PLA General Hospital, No. 8, Dongda Ave, Fengtai District, Beijing, 100071, People's Republic of China.

Liu, Nan. Department of Immunology and National Center for Biomedicine Analysis, the Fifth Medical Center (formerly known as Beijing 307 Hospital), Chinese PLA General Hospital, No. 8, Dongda Ave, Fengtai District, Beijing, 100071, People's Republic of China.

Sun, Yuying. Department of Immunology and National Center for Biomedicine Analysis, the Fifth Medical Center (formerly known as Beijing 307 Hospital), Chinese PLA General Hospital, No. 8, Dongda Ave, Fengtai District, Beijing, 100071, People's Republic of China.

Xi, Yongzhi. Department of Immunology and National Center for Biomedicine Analysis, the Fifth Medical Center (formerly known as Beijing 307 Hospital), Chinese PLA General Hospital, No. 8, Dongda Ave, Fengtai District, Beijing, 100071, People's Republic of China. xiyz@yahoo.com.

Abstract

BACKGROUND: The persistence, biodistribution, and risk of integration into the host genome of any new therapeutic DNA vaccine must be established in preclinical studies. We previously developed the DNA vaccine pcDNA-CCOL2A1 encoding chicken type II collagen (CCII) for the treatment of rheumatoid arthritis (RA). In the present study, we characterized its dynamic profile, biodistribution, and potential for genomic DNA integration in normal vaccinated rodent.

RESULTS: A real-time quantitative PCR analysis (RT-qPCR) of animals administered a single dose of pcDNA-CCOL2A1 (300 mug/kg by intramuscular injection) showed that CCOL2A1 mRNA level in the blood peaked between 2 and 6 h post-immunization and then rapidly declined, and was undetectable between day 1-42. CCOL2A1 transcript was detected at the muscle injection site on days 3-14 post-immunization. Starting from day 14, the transcript was detected in the heart, liver, lung, and kidney but not in the spleen or thymus, and was expressed only in the lung on day 28. There was no CCOL2A1 mRNA present in the testes or ovaries at any time point. Non-invasive in vivo fluorescence imaging revealed CCII protein expression from 2 h up to day 10 and from 2 h up to day 35 after administration of pcDNA-CCOL2A1 via the intravenous and intramuscular routes, respectively; the protein had disappeared by day 42. Importantly, CCOL2A1 was not integrated into the host genome.

CONCLUSIONS: These results indicate that pcDNA-CCOL2A1 vaccine is rapidly cleared within a short period of time and is therefore safe, and merits further development as a therapeutic vaccine for RA treatment.

Publication Type

Journal Article.

Year of Publication

2019

<18>

Unique Identifier

30590695

Title

Recommendations for the management of secondary hypogammaglobulinaemia due to B cell targeted therapies in autoimmune rheumatic diseases.

Source

Rheumatology. 58(5):889-896, 2019 05 01.

VI 1

Status

MEDLINE

Authors

Wijetilleka S; Jayne DR; Mukhtyar C; Ala A; Bright PD; Chinoy H; Harper L; Kazmi MA; Kiani-Alikhan S; Li CK; Misbah SA; Oni L; Price-Kuehne FE; Salama AD; Workman S; Wrench D; Karim MY.

Authors Full Name

Wijetilleka, Sonali; Jayne, David R; Mukhtyar, Chetan; Ala, Aftab; Bright, Philip D; Chinoy, Hector; Harper, Lorraine; Kazmi, Majid A; Kiani-Alikhan, Sorena; Li, Charles K; Misbah, Siraj A; Oni, Louise; Price-Kuehne, Fiona E; Salama, Alan D; Workman, Sarita; Wrench, David; Karim, Mohammed Yousuf.

Institution

Wijetilleka, Sonali. Department of Immunology, Frimley Health NHS Foundation Trust, Frimley.

Jayne, David R. Department of Medicine, University of Cambridge, Cambridge.

Mukhtyar, Chetan. Department of Rheumatology, Norfolk and Norwich University Hospitals NHS Foundation Trust, Norwich.

Ala, Aftab. Department of Gastroenterology and Hepatology, Royal Surrey County Hospital NHS Foundation Trust, Guildford.

Bright, Philip D. Department of Immunology, North Bristol NHS Trust, Bristol.

Chinoy, Hector. Department of Rheumatology, Salford Royal NHS Foundation Trust, Salford.

Harper, Lorraine. Department of Nephrology, Institute of Clinical Sciences-University of Birmingham, Birmingham.

Kazmi, Majid A. Department of Haematology, Guy's and St Thomas' NHS Foundation Trust.

Kiani-Alikhan, Sorena. Department of Immunology, Barts and The London NHS Trust, London.

Li, Charles K. Department of Rheumatology, Royal Surrey County Hospital NHS Foundation Trust, Guildford.

Misbah, Siraj A. Department of Immunology, Oxford University Hospitals, Oxford.

Oni, Louise. Department of Paediatric Nephrology, Alder Hey Children's NHS Foundation Trust Hospital, Liverpool.

Price-Kuehne, Fiona E. Department of Paediatrics, University of Cambridge School of Clinical Medicine, Cambridge.

Salama, Alan D. Department of Nephrology, University College London Centre for Nephrology.

Workman, Sarita. Department of Immunology, Royal Free London NHS Foundation Trust, London, UK.

Wrench, David. Department of Haematology, Guy's and St Thomas' NHS Foundation Trust.

Karim, Mohammed Yousuf. Department of Pathology, Sidra Medicine, Doha, Qatar.

Abstract

OBJECTIVES: The association of B cell targeted therapies with development of hypogammaglobulinaemia and infection is increasingly recognized. Our aim was to develop consensus recommendations for immunoglobulin replacement therapy for management of hypogammaglobulinaemia following B cell targeted therapies in autoimmune rheumatic diseases.

METHODS: A modified Delphi exercise involved a 17-member Taskforce committee, consisting of immunologists, rheumatologists, nephrologists, haematologists, a gastroenterologist, an immunology specialist nurse and a patient representative. The first round identified the most pertinent topics to address in the recommendations. A search string was agreed upon for the identification of publications in PubMed focusing on these areas, for a systematic literature review. Original data was presented from this review to the Taskforce committee. Recommendations from the British Society for Rheumatology, the UK Department of Health, EULAR, the ACR, and the American Academy of Allergy, Asthma, and Immunology were also reviewed. The evidence was discussed in a face-to-face meeting to formulate recommendation statements. The levels of evidence and statements were graded according to Scottish Intercollegiate Guidelines Network methodology.

RESULTS: Three overarching principles, eight recommendation statements and a research agenda were formulated. The Taskforce committee voted on these statements, achieving 82-100% agreement for each recommendation. The strength of the recommendations was restricted by the low quality of the available evidence, with no randomized controlled trial data. The recommendations cover risk factors, monitoring, referral for hypogammaglobulinaemia; indications, dosage and discontinuation of immunoglobulin replacement therapy.

CONCLUSION: These are the first recommendations specifically formulated for B cell targeted therapies related to hypogammaglobulinaemia in autoimmune rheumatic diseases. The recommendations are to aid health-care professionals with clinical decision making for patients with hypogammaglobulinaemia. Copyright © The Author(s) 2018. Published by Oxford University Press on behalf of the British Society for Rheumatology. All rights reserved. For permissions, please email: journals.permissions@oup.com.
Publication Type
Journal Article. Research Support, Non-U.S. Gov't.
Year of Publication
2019

<19>
Unique Identifier
30659547
Title
Immune Checkpoint Inhibitors-related Rheumatic Diseases: What Rheumatologist Should Know?. **[Review]**
Source
Current Rheumatology Reviews. 15(3):201-208, 2019.
VI 1
Status
MEDLINE
Authors
Gediz F; Kobak S.
Authors Full Name

Gediz, Fusun; Kobak, Senol.
Institution
Gediz, Fusun. Department of Hematology, Transplantation Unit, Izmir Training and Research Hospital, Bone Marrow, Izmir, Turkey.
Kobak, Senol. Department of Rheumatology, Istinye University Faculty of Medicine, Liv Hospital, Istanbul, Turkey.
Abstract
Immune checkpoint inhibitors are revolutionized drugs for cancer immunotherapy in the last years. The mechanism of action of CPIs including the limitation of the activation of Tcells, and thus enhancing the self-immune response against tumour cells. Checkpointinhibitors(CPIs) may dysregulate the immune system, resulting in some toxicities. These toxicities or side effects are called Immune-related Adverse Events (IRAEs) that can potentially affect any organ and tissue. Rheumatic diseases due to checkpoint inhibitors are also reported in the literature. The spectrum of rheumatic manifestations are quite wide; the most common are arthralgia/arthritis, myalgia/myositis, polimyalgia rheumatica, lupus, rheumatoid arthritis, Sjogren's syndrome. At the same time, these drugs can also cause an exacerbation of known rheumatologic disease. Treatment approaches for developing rheumatic findings due to checkpoint inhibitors should be multidisciplinary. There should be a close relationship between oncologists who follow-up these patients and rheumatologists. The rheumatic manifestations should be defined and treated early. In general, the musculoskeletal side effects are transient and may regress after stopping CPIs. The most commonly used medications are corticosteroids. Immunosuppressive drugs (HQ, MTX, anti-TNF-alpha, anti-IL-6) should be preferred when treatment is unresponsive or as steroid-sparing agents. The aim of this review was to evaluate the checkpoint inhibitors-related rheumatologic findings and therapeutic strategies in light of recent literature data. Copyright© Bentham Science Publishers; For any queries, please email at epub@benthamscience.net.
Publication Type
Journal Article. Review.
Year of Publication
2019

<20>
Unique Identifier
30837988

Title

B Cells as a Therapeutic Target in Paediatric Rheumatic Disease. [Review]

Source

Frontiers in Immunology. 10:214, 2019.

VI 1

Status

MEDLINE

Authors

Wilkinson MGL; Rosser EC.

Authors Full Name

Wilkinson, Meredyth G Ll; Rosser, Elizabeth C.

Institution

Wilkinson, Meredyth G Ll. Infection, Immunity, Inflammation Programme, UCL Great Ormond Street Institute of Child Health, London, United Kingdom.

Wilkinson, Meredyth G Ll. Arthritis Research UK Centre for Adolescent Rheumatology, University College London, UCLH and GOSH, London, United Kingdom.

Wilkinson, Meredyth G Ll. NIHR Biomedical Research Centre, Great Ormond Street Hospital, London, United Kingdom.

Rosser, Elizabeth C. Infection, Immunity, Inflammation Programme, UCL Great Ormond Street Institute of Child Health, London, United Kingdom.

Rosser, Elizabeth C. Arthritis Research UK Centre for Adolescent Rheumatology, University College London, UCLH and GOSH, London, United Kingdom.

Rosser, Elizabeth C. NIHR Biomedical Research Centre, Great Ormond Street Hospital, London, United Kingdom.

Abstract

B cells carry out a central role in the pathogenesis of autoimmune disease. In addition to the production of autoantibodies, B cells can contribute to disease development by presenting autoantigens to autoreactive T cells and by secreting pro-inflammatory cytokines and chemokines which leads to the amplification of the inflammatory response. Targeting both the antibody-dependent and antibody-independent function of B cells in adult rheumatic disease has led to the advent of B cell targeted therapies in clinical practice. To date, whether B cell depletion could also be utilized for the treatment of pediatric disease is relatively under explored. In this review, we will discuss the role of B cells in the pathogenesis of the pediatric rheumatic diseases Juvenile Idiopathic Arthritis (JIA), Juvenile Systemic Lupus Erythematosus (JSLE) and Juvenile Dermatomyositis (JDM). We will also explore the rationale behind the use of B cell-targeted therapies in pediatric rheumatic disease by highlighting new case studies that points to their efficacy in JIA, JSLE, and JDM.

Publication Type
  Journal Article. Research Support, Non-U.S. Gov't. Review.
Year of Publication
  2019

<21>
Unique Identifier
  30837986
Title
  Humanized Mouse Models of Rheumatoid Arthritis for Studies on Immunopathogenesis and Preclinical Testing of Cell-Based Therapies. [Review]
Source
  Frontiers in Immunology. 10:203, 2019.
VI 1
Status
  MEDLINE
Authors
  Schinnerling K; Rosas C; Soto L; Thomas R; Aguillon JC.
Authors Full Name
  Schinnerling, Katina; Rosas, Carlos; Soto, Lilian; Thomas, Ranjeny; Aguillon, Juan Carlos.
Institution
  Schinnerling, Katina. Programa Disciplinario de Inmunologia, Immune Regulation and Tolerance Research Group, Facultad de Medicina, Instituto de Ciencias Biomedicas, Universidad de Chile, Santiago, Chile.
  Schinnerling, Katina. Departamento de Ciencias Biologicas, Facultad de Ciencias de la Vida, Universidad Andres Bello, Santiago, Chile.
  Rosas, Carlos. Departamento de Ciencias Morfologicas, Facultad de Medicina y Ciencia, Universidad San Sebastian, Santiago, Chile.
  Soto, Lilian. Programa Disciplinario de Inmunologia, Immune Regulation and Tolerance Research Group, Facultad de Medicina, Instituto de Ciencias Biomedicas, Universidad de Chile, Santiago, Chile.
  Soto, Lilian. Unidad de Dolor, Departamento de Medicina, Hospital Clinico Universidad de Chile, Santiago, Chile.

Thomas, Ranjeny. Diamantina Institute, Translational Research Institute, Princess Alexandra Hospital, University of Queensland, Brisbane, QLD, Australia.
Aguillon, Juan Carlos. Programa Disciplinario de Inmunologia, Immune Regulation and Tolerance Research Group, Facultad de Medicina, Instituto de Ciencias Biomedicas, Universidad de Chile, Santiago, Chile.
Abstract
Rodent models of rheumatoid arthritis (RA) have been used over decades to study the immunopathogenesis of the disease and to explore intervention strategies. Nevertheless, mouse models of RA reach their limit when it comes to testing of new therapeutic approaches such as cell-based therapies. Differences between the human and the murine immune system make it difficult to draw reliable conclusions about the success of immunotherapies. To overcome this issue, humanized mouse models have been established that mimic components of the human immune system in mice. Two main strategies have been pursued for humanization: the introduction of human transgenes such as human leukocyte antigen molecules or specific T cell receptors, and the generation of mouse/human chimera by transferring human cells or tissues into immunodeficient mice. Recently, both approaches have been combined to achieve more sophisticated humanized models of autoimmune diseases. This review discusses limitations of conventional mouse models of RA-like disease and provides a closer look into studies in humanized mice exploring their usefulness and necessity as preclinical models for testing of cell-based therapies in autoimmune diseases such as RA.
Publication Type
Journal Article. Research Support, Non-U.S. Gov't. Review.
Year of Publication
2019

<22>
Unique Identifier
30927735
Title
alpha-Difluoromethylornithine suppresses inflammatory arthritis by impairing myeloid-derived suppressor cells.
Source
International Immunopharmacology. 71:251-258, 2019 Jun.

VI 1
Status
MEDLINE
Authors
Geng Z; Ming B; Hu S; Dong L; Ye C.
Authors Full Name
Geng, Zhe; Ming, Bingxia; Hu, Shaoxian; Dong, Lingli; Ye, Cong.
Institution
Geng, Zhe. Department of Hematology, Tongji Hospital, Tongji Medical College, Huazhong University of Science and Technology, Wuhan 430030, China.

Ming, Bingxia. Department of Rheumatology and Immunology, Tongji Hospital, Tongji Medical College, Huazhong University of Science and Technology, Wuhan 430030, China.

Hu, Shaoxian. Department of Rheumatology and Immunology, Tongji Hospital, Tongji Medical College, Huazhong University of Science and Technology, Wuhan 430030, China.

Dong, Lingli. Department of Rheumatology and Immunology, Tongji Hospital, Tongji Medical College, Huazhong University of Science and Technology, Wuhan 430030, China.

Ye, Cong. Department of Rheumatology and Immunology, Tongji Hospital, Tongji Medical College, Huazhong University of Science and Technology, Wuhan 430030, China. Electronic address: yecong@tjh.tjmu.edu.cn.
Abstract
OBJECTIVES: The chemopreventive drug alpha-difluoromethylornithine (DFMO) has been shown to have an antinociceptive effect on mechanical allodynia in inflammatory arthritis by directly inhibiting ornithine decarboxylase (ODC) and decreasing polyamine production in inflammatory sites. However, little is known about the effect of DFMO on the immune system of inflammatory arthritis. Here, we investigated the effect of DFMO in a well-established collagen-induced arthritis (CIA) mouse model and explored its effect on the immune system.

METHODS: The effect of DFMO on the frequency of myeloid-derived suppressor cells (MDSCs) in the spleens of CIA mice and their associations with disease severity, tissue inflammation and the levels of proinflammatory T-helper (Th) 17 cells in lymphoid tissues were investigated. The effects of DFMO on disease severity and Th17 cells were compared with those of antibody depletion of MDSCs. The arthritis severity was also evaluated by adoptive transfer of MDSCs derived from DFMO- or dH2O-treated mice.

RESULTS: In this study, we showed that both MDSCs and Th17 cells were significantly expanded in CIA mice. Treatment by DFMO at the onset of CIA suppressed the development of arthritis and decreased the frequency of MDSCs and Th17 cells. MDSC depletion by anti-Gr-1

mAb achieved a similar result, while combination treatment of both methods did not achieve a significant difference compared to either of the single treatments. In addition, the adoptive transfer of MDSCs derived from dH2O-treated mice with CIA restored the arthritis severity of CIA in mice treated with anti-Gr-1 mAb, while the transfer of MDSCs from DFMO-treated mice did not have such an effect.

CONCLUSIONS: Our results identified DFMO as a potential therapeutic drug for the treatment of inflammatory arthritis. Copyright © 2019 Elsevier B.V. All rights reserved.

Publication Type

Journal Article.

Year of Publication

2019

<23>

Unique Identifier

30508191

Title

Association of HLA-DRB1 shared epitope alleles and immune checkpoint inhibitor-induced inflammatory arthritis. Rheumatology. 58(3):476-480, 2019 03 01.

Authors

Cappelli LC; Dorak MT; Bettinotti MP; Bingham CO; Shah AA.

Authors Full Name

Cappelli, Laura C; Dorak, Mehmet T; Bettinotti, Maria P; Bingham, Clifton O; Shah, Ami A.

Institution

Cappelli, Laura C. Division of Rheumatology, The Johns Hopkins University School of Medicine, Baltimore, MD, USA.

Dorak, Mehmet T. School of Life Sciences, Pharmacy & Chemistry, Kingston University London, London, UK.

Bettinotti, Maria P. Immunogenetics Laboratory, Department of Medicine, The Johns Hopkins University School of Medicine, Baltimore, MD, USA.

Bingham, Clifton O. Division of Rheumatology, The Johns Hopkins University School of Medicine, Baltimore, MD, USA.

Shah, Ami A. Division of Rheumatology, The Johns Hopkins University School of Medicine, Baltimore, MD, USA.

Abstract

OBJECTIVE: To evaluate the frequency of HLA class I and II alleles associated with traditional forms of inflammatory arthritis in patients with immune checkpoint inhibitor (ICI)-induced inflammatory arthritis as compared with population controls.

METHODS: High-resolution HLA typing was performed on 27 patients with ICI-induced inflammatory arthritis and 726 healthy controls. Genotyping at the shared epitope (SE) locus (HLA DRB1) was performed on 220 RA cases. Allele-positivity rates and frequency of having at least one SE allele were compared using Fisher's exact test between ICI-induced inflammatory arthritis and healthy controls. Frequency of having at least one SE allele was also compared between ICI-induced inflammatory arthritis and RA cases.

RESULTS: Twenty-six patients with ICI-induced inflammatory arthritis were of European descent, and one was African American. In those 26 patients, 16 (61.5%) had at least one SE allele, significantly different from healthy controls of European descent, in whom 299 (41.2%) had at least one SE allele (odds ratio 2.3, P = 0.04). The allele-positivity rate of DRB1*04: 05 was also higher in the ICI-induced inflammatory arthritis group. The ICI-induced inflammatory arthritis population and RA patients of European descent did not differ in frequency of having at least one SE allele, but ICI-induced inflammatory arthritis patients were more likely to be autoantibody-negative for RF and anti-CCP antibodies.

CONCLUSION: Patients with ICI-induced inflammatory arthritis of European descent were more likely to have at least one SE allele than healthy controls. Further studies are needed to validate these findings and investigate whether a unique immunogenetic framework increases risk for different immune-related adverse events. Copyright © The Author(s) 2018. Published by Oxford University Press on behalf of the British Society for Rheumatology. All rights reserved. For permissions, please email: journals.permissions@oup.com.

Publication Type

Journal Article. Research Support, N.I.H., Extramural. Research Support, Non-U.S. Gov't.

Year of Publication

2019

<24>

Unique Identifier

29572292

Title

Checkpoint inhibitors and arthritis.

Source

Annals of the Rheumatic Diseases. 78(6):e58, 2019 06.

VI 1

Status

MEDLINE

Authors

Manolios N; Schrieber L.

Authors Full Name

Manolios, Nicholas; Schrieber, Leslie.

Institution

Manolios, Nicholas. Faculty of Medicine, University of Sydney, Sydney, New South Wales, Australia.

Manolios, Nicholas. Department of Rheumatology, Westmead Hospital, Sydney, New South Wales, Australia.

Schrieber, Leslie. Faculty of Medicine, University of Sydney, Sydney, New South Wales, Australia.

Schrieber, Leslie. Department of Rheumatology, Royal North Shore Hospital, Sydney, New South Wales, Australia.

Comments

Comment on (CON)

Publication Type

Letter. Comment.

Year of Publication

2019

<25>

Unique Identifier

30474934

Title

Ibudilast Inhibits Chemokine Expression in Rheumatoid Arthritis Synovial Fibroblasts and Exhibits Immunomodulatory Activity in Experimental Arthritis.

Source

Arthritis & Rheumatology. 71(5):703-711, 2019 05.

VI 1

Status

MEDLINE

Authors

Clanchy FIL; Williams RO.

Authors Full Name

Clanchy, Felix I L; Williams, Richard O.

Institution

Clanchy, Felix I L. Kennedy Institute of Rheumatology, University of Oxford, Oxford, UK.
Williams, Richard O. Kennedy Institute of Rheumatology, University of Oxford, Oxford, UK.

Abstract

OBJECTIVE: Ibudilast is a well-tolerated, orally available phosphodiesterase 4 (PDE4) inhibitor used to treat asthma and stroke. Since PDE4 inhibition suppresses inflammatory mediator production and cell proliferation in leukocytes, ibudilast may be a valuable therapy for the treatment of inflammatory autoimmune diseases such as rheumatoid arthritis (RA). This study was undertaken to assess the therapeutic potential of ibudilast by measuring its capacity to modulate inflammation in human leukocytes and RA synovial fibroblasts (RASFs) and in experimental arthritis.

METHODS: Using standard curve quantitative polymerase chain reaction, the effect of ibudilast on gene expression in activated human leukocytes and RASFs was measured. Ibudilast was used to treat DBA/1 mice with collagen-induced arthritis, and an adoptive transfer model was used to assess its tolerogenic capacity.

RESULTS: Ibudilast inhibited the expression of TNF, IL12A, and IL12B and the secretion of tumor necrosis factor (TNF) and interleukin-12 (IL-12)/23p40 from leukocytes, and reduced the expression of CCL5 and CCL3 in activated RASFs. Treatment of experimental arthritis with ibudilast resulted in a reduction in IL-17-producing cells and inhibition of disease progression. When combined with a TNF inhibitor, ibudilast caused marked suppression of active disease. Exposure of leukocytes from type II collagen-immunized DBA/1 mice to ibudilast in vitro

attenuated their ability to adoptively transfer arthritis to DBA/1J-PrkdcSCID mice, providing evidence of an immunomodulatory effect.

CONCLUSION: Our findings indicate that ibudilast reduces the expression and/or secretion of inflammatory mediators from activated human leukocytes and RASFs, inhibits Th17 cell responses in vivo, and improves established arthritis. Given the established safety profile of ibudilast in humans, its clinical evaluation in RA, either alone or in combination with a TNF inhibitor, should be considered. Copyright © 2018, American College of Rheumatology.

Publication Type

Journal Article.

Year of Publication

2019

<26>

Unique Identifier

29451845

Title

Relapse of Juvenile Idiopathic Arthritis-Associated Uveitis after Discontinuation of Immunomodulatory Therapy.

Source

Ocular Immunology & Inflammation. 27(4):686-692, 2019.

VI 1

Status

MEDLINE

Authors

Acharya NR; Patel S; Homayounfar G; Enanoria WTA; Shakoor A; Chakrabarti A; Goldstein DA.

Authors Full Name

Acharya, Nisha R; Patel, Sarju; Homayounfar, Gelareh; Enanoria, Wayne T A; Shakoor, Akbar; Chakrabarti, Anindita; Goldstein, Debra A.

Institution

Acharya, Nisha R. a F.I. Proctor Foundation, University of California San Francisco, San Francisco, California, USA.

Acharya, Nisha R. b Department of Ophthalmology, University of California San Francisco, San Francisco, California, USA.
Acharya, Nisha R. c Department of Epidemiology and Biostatistics, University of California San Francisco, San Francisco, California, USA.
Patel, Sarju. d Department of Ophthalmology, University of Illinois at Chicago, Chicago, Illinois, USA.
Patel, Sarju. e Retina Consultants Medical Group, Inc, Sacramento, CA, USA.
Homayounfar, Gelareh. a F.I. Proctor Foundation, University of California San Francisco, San Francisco, California, USA.
Enanoria, Wayne T A. a F.I. Proctor Foundation, University of California San Francisco, San Francisco, California, USA.
Shakoor, Akbar. a F.I. Proctor Foundation, University of California San Francisco, San Francisco, California, USA.
Shakoor, Akbar. f Moran Eye Center, University of Utah, Salt Lake City, Utah, USA.
Chakrabarti, Anindita. d Department of Ophthalmology, University of Illinois at Chicago, Chicago, Illinois, USA.
Goldstein, Debra A. d Department of Ophthalmology, University of Illinois at Chicago, Chicago, Illinois, USA.
Goldstein, Debra A. g Department of Ophthalmology, Northwestern University, Evanston, Illinois, USA.

Abstract

Purpose: To assess treatment outcomes in juvenile idiopathic arthritis (JIA)-associated uveitis and relapse rates upon discontinuation of immunomodulatory therapy (IMT). Methods: Medical records of patients with JIA-associated uveitis seen at the University of Illinois at Chicago and the F.I. Proctor Foundation uveitis clinics from September 14, 1988 to January 5, 2011 were reviewed. The main outcome was time to relapse after attempting to discontinue IMT. Results: Of 66 patients with JIA-associated uveitis, 51 (77%) received IMT as either sole or combination therapy. Of a total of 51, 41 (80%) patients achieved corticosteroid-sparing control. Attempts were made to discontinue treatment in 19/51 (37%) patients. Of a total of 19 patients, 13 (68%) attempting to discontinue IMT relapsed, with a median time to relapse of 288 days from the time of attempted taper/discontinuation (IQR: 108-338). Conclusions: Corticosteroid-sparing control of inflammation was achieved in the majority of patients; however, attempts to stop IMT were often unsuccessful. Close follow-up of patients after discontinuation of therapy is warranted.

Publication Type

Journal Article. Multicenter Study.

Year of Publication

2019

<27>

Unique Identifier

30862648

Title

Active immunisation targeting nerve growth factor attenuates chronic pain behaviour in murine osteoarthritis.

Source

Annals of the Rheumatic Diseases. 78(5):672-675, 2019 05.

VI 1

Status

MEDLINE

Authors

von Loga IS; El-Turabi A; Jostins L; Miotla-Zarebska J; Mackay-Alderson J; Zeltins A; Parisi I; Bachmann MF; Vincent TL.

Author NameID

von Loga, Isabell S; ORCID: https://orcid.org/0000-0001-7949-8654

El-Turabi, Aadil; ORCID: https://orcid.org/0000-0003-1092-8155

Authors Full Name

von Loga, Isabell S; El-Turabi, Aadil; Jostins, Luke; Miotla-Zarebska, Jadwiga; Mackay-Alderson, Jennifer; Zeltins, Andris; Parisi, Ida; Bachmann, Martin F; Vincent, Tonia L.

Institution

von Loga, Isabell S. Kennedy Institute of Rheumatology, University of Oxford, Oxford, UK.

El-Turabi, Aadil. The Jenner Institute, University of Oxford Medical Sciences Division, Oxford, UK.

Jostins, Luke. Kennedy Institute of Rheumatology, University of Oxford, Oxford, UK.

Miotla-Zarebska, Jadwiga. Kennedy Institute of Rheumatology, University of Oxford, Oxford, UK.

Mackay-Alderson, Jennifer. Kennedy Institute of Rheumatology, University of Oxford, Oxford, UK.

Zeltins, Andris. Molecular Microbiology and Virology, Latvian Biomedical Research & Study Centre, Riga, Latvia.

Parisi, Ida. Kennedy Institute of Rheumatology, University of Oxford, Oxford, UK.

Bachmann, Martin F. The Jenner Institute, University of Oxford Medical Sciences Division, Oxford, UK tonia.vincent@kennedy.ox.ac.uk martin.bachmann@ndm.ox.ac.uk.
Bachmann, Martin F. RIA, Immunology, Inselspital, 3010 Bern, Switzerland.
Vincent, Tonia L. Kennedy Institute of Rheumatology, University of Oxford, Oxford, UK tonia.vincent@kennedy.ox.ac.uk martin.bachmann@ndm.ox.ac.uk.
Abstract
OBJECTIVES: Nerve growth factor (NGF) has emerged as a key driver of pain in osteoarthritis (OA) and antibodies to NGF are potent analgesics in human disease. Here, we validate a novel vaccine strategy to generate anti-NGF antibodies for reversal of pain behaviour in a surgical model of OA.

METHODS: Virus-like particles were derived from the cucumber mosaic virus (CuMV) and coupled to expressed recombinant NGF to create the vaccine. 10-week-old male mice underwent partial meniscectomy to induce OA or sham-surgery. Spontaneous pain behaviour was measured by Linton incapacitance and OA severity was quantified using OARSI histological scoring. Mice (experimental and a sentinel cohort) were inoculated with CuMVttNGF (Vax) or CuMVttctrl (Mock) either before surgery or once pain was established. Efficacy of anti-NGF from the plasma of sentinel vaccinated mice was measured in vitro using a neurite outgrowth assay in PC12 cells.

RESULTS: Anti-NGF titres were readily detectable in the vaccinated but not mock vaccinated mice. Regular boosting with fresh vaccine was required to maintain anti-NGF titres as measured in the sentinel cohort. Both prophylactic and therapeutic vaccination demonstrated a reversal of pain behaviour by incapacitance testing, and a meta-analysis of the two studies showing analgesia at peak anti-NGF titres was highly statistically significant. Serum anti-NGF was able to inhibit neurite outgrowth equivalent to around 150 ug/mL of recombinant monoclonal antibody.

CONCLUSIONS: This study demonstrates therapeutic efficacy of a novel NGF vaccine strategy that reversibly alleviates spontaneous pain behaviour in surgically induced murine OA. Copyright © Author(s) (or their employer(s)) 2019. Re-use permitted under CC BY. Published by BMJ.
Publication Type
Journal Article. Research Support, Non-U.S. Gov't.
Year of Publication
2019

<28>

Unique Identifier

31527369

Title

Simultaneous Development of Progressive Multifocal Leukoencephalopathy and Cryptococcal Meningitis during Methotrexate and Infliximab Treatment.

Source

Internal Medicine. 58(18):2703-2709, 2019.

VI 1

Status

MEDLINE

Authors

Nosaki Y; Ohyama K; Watanabe M; Yokoi T; Nakamichi K; Saijo M; Miura Y; Iwai K.

Authors Full Name

Nosaki, Yasunobu; Ohyama, Ken; Watanabe, Maki; Yokoi, Takamasa; Nakamichi, Kazuo; Saijo, Masayuki; Miura, Yoshiharu; Iwai, Katsushige.

Institution

Nosaki, Yasunobu. Department of Neurology, Toyohashi Municipal Hospital, Japan.

Ohyama, Ken. Department of Neurology, Toyohashi Municipal Hospital, Japan.

Watanabe, Maki. Department of Neurology, Toyohashi Municipal Hospital, Japan.

Yokoi, Takamasa. Department of Neurology, Toyohashi Municipal Hospital, Japan.

Nakamichi, Kazuo. Department of Virology 1, National Institute of Infectious Diseases, Japan.

Saijo, Masayuki. Department of Virology 1, National Institute of Infectious Diseases, Japan.

Miura, Yoshiharu. Department of Neurology, Tokyo Metropolitan Cancer and Infectious Diseases Center Komagome Hospital, Japan.

Iwai, Katsushige. Department of Neurology, Toyohashi Municipal Hospital, Japan.

Abstract

Progressive multifocal leukoencephalopathy (PML) is a demyelinating disease of the central nervous system caused by reactivation of the JC virus under an immunosuppressed state. This condition carries a high risk of cryptococcal meningitis. We herein report a 65-year-old woman who simultaneously developed PML and cryptococcal meningitis and presented with bilateral sixth nerve palsy. She had been treated with methotrexate and infliximab for rheumatoid arthritis. Her symptoms improved with antifungal drug treatment and discontinuation of immunosuppression therapy. Although concurrent PML and cryptococcal meningitis is rare, it should be considered in immunosuppressed patients.

Publication Type

Case Reports. Journal Article.
Year of Publication
2019

<29>
Unique Identifier
30285531
Title
Rheumatic diseases associated with immune checkpoint inhibitors in cancer immunotherapy. [Review]
Source
Modern Rheumatology. 29(5):721-732, 2019 Sep.
VI 1
Status
MEDLINE
Authors
Ohnuma K; Hatano R; Dang NH; Morimoto C.
Authors Full Name
Ohnuma, Kei; Hatano, Ryo; Dang, Nam H; Morimoto, Chikao.
Institution
Ohnuma, Kei. Department of Therapy Development and Innovation for Immune Disorders and Cancers, Graduate School of Medicine, Juntendo University, Tokyo, Japan.
Hatano, Ryo. Department of Therapy Development and Innovation for Immune Disorders and Cancers, Graduate School of Medicine, Juntendo University, Tokyo, Japan.
Dang, Nam H. Division of Hematology/Oncology, University of Florida, Gainesville, FL, USA.
Morimoto, Chikao. Department of Therapy Development and Innovation for Immune Disorders and Cancers, Graduate School of Medicine, Juntendo University, Tokyo, Japan.
Abstract
Immune checkpoint inhibitors (ICIs) have drastically altered cancer treatment paradigms, with increasing numbers of novel ICIs being currently evaluated in numerous clinical trials for various cancers. ICIs release 'brakes' against tumor immunity to control cancer growth through T cell-dependent anti-tumor activity. Meanwhile, side effects associated with ICIs are directly related to their mechanism of action, as nonspecific immune activation targeting non-tumor organs results

in undesirable off-target inflammation and autoimmunity. Accumulating data reveal that immune-related adverse events (irAEs) of ICIs in cancer patients can resemble various rheumatic diseases. Moreover, while patients with preexisting rheumatic diseases can theoretically experience irAEs and disease flares, observational studies have shown that ICIs can be used successfully in these patients. As ICIs continue to provide long-lasting disease control in cancer patients and their usage correspondingly increases, the rheumatologist will be managing new ICI-associated clinical entities mimicking common autoimmune diseases and will need to be prepared to rapidly diagnose and treat these irAEs. Early recognition and treatment of these rheumatic adverse events will allow for improved outcomes and quality of life for cancer patients faced with previously rapidly fatal disease.

Publication Type

Journal Article. Review.

Year of Publication

2019

<30>

Unique Identifier

29972778

Title

Group 2 Innate Lymphoid Cells Attenuate Inflammatory Arthritis and Protect from Bone Destruction in Mice.

Source

Cell Reports. 24(1):169-180, 2018 07 03.

VI 1

Status

MEDLINE

Authors

Omata Y; Frech M; Primbs T; Lucas S; Andreev D; Scholtysek C; Sarter K; Kindermann M; Yeremenko N; Baeten DL; Andreas N; Kamradt T; Bozec A; Ramming A; Kronke G; Wirtz S; Schett G; Zaiss MM.

Authors Full Name

Omata, Yasunori; Frech, Michael; Primbs, Tatjana; Lucas, Sebastien; Andreev, Darja; Scholtysek, Carina; Sarter, Kerstin; Kindermann, Markus; Yeremenko, Nataliya; Baeten,

Dominique L; Andreas, Nico; Kamradt, Thomas; Bozec, Aline; Ramming, Andreas; Kronke, Gerhard; Wirtz, Stefan; Schett, Georg; Zaiss, Mario M.
Institution
Omata, Yasunori. Department of Internal Medicine 3, Rheumatology and Immunology, Friedrich-Alexander-University Erlangen-Nurnberg (FAU) and Universitatsklinikum Erlangen, Erlangen, Germany.
Frech, Michael. Department of Internal Medicine 3, Rheumatology and Immunology, Friedrich-Alexander-University Erlangen-Nurnberg (FAU) and Universitatsklinikum Erlangen, Erlangen, Germany.
Primbs, Tatjana. Department of Internal Medicine 1, University of Erlangen-Nuremberg, Erlangen, Germany.
Lucas, Sebastien. Department of Internal Medicine 3, Rheumatology and Immunology, Friedrich-Alexander-University Erlangen-Nurnberg (FAU) and Universitatsklinikum Erlangen, Erlangen, Germany.
Andreev, Darja. Department of Internal Medicine 3, Rheumatology and Immunology, Friedrich-Alexander-University Erlangen-Nurnberg (FAU) and Universitatsklinikum Erlangen, Erlangen, Germany.
Scholtysek, Carina. Department of Internal Medicine 3, Rheumatology and Immunology, Friedrich-Alexander-University Erlangen-Nurnberg (FAU) and Universitatsklinikum Erlangen, Erlangen, Germany.
Sarter, Kerstin. Department of Internal Medicine 3, Rheumatology and Immunology, Friedrich-Alexander-University Erlangen-Nurnberg (FAU) and Universitatsklinikum Erlangen, Erlangen, Germany.
Kindermann, Markus. Department of Internal Medicine 1, University of Erlangen-Nuremberg, Erlangen, Germany.
Yeremenko, Nataliya. Department of Clinical Immunology and Rheumatology and Department of Experimental Immunology, Academic Medical Center/University of Amsterdam, Amsterdam, the Netherlands.
Baeten, Dominique L. Department of Clinical Immunology and Rheumatology and Department of Experimental Immunology, Academic Medical Center/University of Amsterdam, Amsterdam, the Netherlands.
Andreas, Nico. Institute of Immunology, Jena University Hospital, Leutragraben 3, 07743 Jena, Germany.
Kamradt, Thomas. Institute of Immunology, Jena University Hospital, Leutragraben 3, 07743 Jena, Germany.

Bozec, Aline. Department of Internal Medicine 3, Rheumatology and Immunology, Friedrich-Alexander-University Erlangen-Nurnberg (FAU) and Universitatsklinikum Erlangen, Erlangen, Germany.

Ramming, Andreas. Department of Internal Medicine 3, Rheumatology and Immunology, Friedrich-Alexander-University Erlangen-Nurnberg (FAU) and Universitatsklinikum Erlangen, Erlangen, Germany.

Kronke, Gerhard. Department of Internal Medicine 3, Rheumatology and Immunology, Friedrich-Alexander-University Erlangen-Nurnberg (FAU) and Universitatsklinikum Erlangen, Erlangen, Germany.

Wirtz, Stefan. Department of Internal Medicine 1, University of Erlangen-Nuremberg, Erlangen, Germany.

Schett, Georg. Department of Internal Medicine 3, Rheumatology and Immunology, Friedrich-Alexander-University Erlangen-Nurnberg (FAU) and Universitatsklinikum Erlangen, Erlangen, Germany.

Zaiss, Mario M. Department of Internal Medicine 3, Rheumatology and Immunology, Friedrich-Alexander-University Erlangen-Nurnberg (FAU) and Universitatsklinikum Erlangen, Erlangen, Germany. Electronic address: mario.zaiss@uk-erlangen.de.

Abstract

Group 2 innate lymphoid cells (ILC2s) were detected in the peripheral blood and the joints of rheumatoid arthritis (RA) patients, serum-induced arthritis (SIA), and collagen-induced arthritis (CIA) using flow cytometry. Circulating ILC2s were significantly increased in RA patients compared with healthy controls and inversely correlated with disease activity. Induction of arthritis in mice led to a fast increase in ILC2 number. To elucidate the role of ILC2 in arthritis, loss- and gain-of-function mouse models for ILC2 were subjected to arthritis. Reduction of ILC2 numbers in RORalphacre/GATA3fl/fl and Tie2cre/RORalphafl/fl mice significantly exacerbated arthritis. Increasing ILC2 numbers in mice by IL-25/IL-33 mini-circles or IL-2/IL-2 antibody complex and the adoptive transfer of wild-type (WT) ILC2s significantly attenuated arthritis by affecting the initiation phase. In addition, adoptive transfer of IL-4/13-competent WT but not IL-4/13-/- ILC2s and decreased cytokine secretion by macrophages. These data show that ILC2s have immune-regulatory functions in arthritis. Copyright © 2018 The Authors. Published by Elsevier Inc. All rights reserved.

Publication Type

Journal Article. Research Support, Non-U.S. Gov't.

Year of Publication

2018

<31>

Unique Identifier

31168760

Title

Autoimmunity in 2018.

Source

Clinical Reviews in Allergy & Immunology. 56(3):375-384, 2019 Jun.

VI 1

Status

MEDLINE

Authors

Selmi C.

Author NameID

Selmi, Carlo; ORCID: http://orcid.org/0000-0002-0323-0376

Authors Full Name

Selmi, Carlo.

Institution

Selmi, Carlo. Division of Rheumatology and Clinical Immunology, Humanitas Clinical and Research Center - IRCCS -, via A. Manzoni 56, 20089, Rozzano, Milan, Italy. carlo.selmi@humanitas.it.

Selmi, Carlo. BIOMETRA Department, University of Milan, Milan, Italy. carlo.selmi@humanitas.it.

Abstract

In the vast database of peer-reviewed articles, the number of 2018 papers published retrieved using the "autoimmunity" keyword remained unchanged compared with the brilliant results of 2017 while returning above a 5% share within the immunology field, after the brisk decrease of this ratio in 2017. As in the past 12 years, we have now searched PubMed for publications related to autoimmunity in the major immunology and autoimmunity peer-reviewed journals and provide here an arbitrary discussion of the major themes encountered. Once again, we are happy to notice that similarities between autoimmune diseases and the common mechanisms significantly outnumber differences. Some examples include data on Th17 cells, cytokines, or other mediators variably involved in the autoimmunity mechanisms such as BLIMP-1, IL-10, IFN, or NF-kB. The study of the microbiome remains central to autoimmunity development and data are being gathered in a growing number of conditions, similar to epigenetics and long non-coding RNA. In

the cases of specific diseases, such as systemic lupus erythematosus, rheumatoid arthritis, or psoriatic arthritis, multiple encouraging findings underline the importance of a strict relationship between basic and clinical science to define new pathogenetic and therapeutic developments. Cumulatively, the present scenario of autoimmunity appears bright and should be regarded as one of the fastest growing in the scientific field of immunology, despite the enormous attention paid to cancer immune mechanisms. The parallel observation that the rheumatology therapeutic pipeline is second only to oncology increases the hopes that more and more patients will be satisfactorily treated in the near future.
Publication Type
  Journal Article.
Year of Publication
  2019

<32>
Unique Identifier
  29419465
Title
  Safety, and Humoral and Cell-mediated Immune Responses to Herpes Zoster Vaccine in Patients with Rheumatoid Arthritis.
Source
  Journal of Rheumatology. 45(4):465-469, 2018 04.
VI 1
Status
  MEDLINE
Authors
  Koh JH; Lee J; Kim SH; Kwok SK; Ju JH; Park SH.
Authors Full Name
  Koh, Jung Hee; Lee, Jaeseon; Kim, Seo Hwa; Kwok, Seung-Ki; Ju, Ji Hyeon; Park, Sung-Hwan.
Institution
  Koh, Jung Hee. From the Division of Rheumatology, Department of Internal Medicine, St. Mary's Hospital, College of Medicine, The Catholic University of Korea, Seoul, South Korea.
   Koh, Jung Hee. J.H. Koh, MD, Division of Rheumatology, Department of Internal Medicine, St. Mary's Hospital, College of Medicine, The Catholic University of Korea; S.H. Kim, MD, Division of

Rheumatology, Department of Internal Medicine, St. Mary's Hospital, College of Medicine, The Catholic University of Korea; S.K. Kwok, MD, PhD; Division of Rheumatology, Department of Internal Medicine, St. Mary's Hospital, and Rheumatism Research Center, College of Medicine, The Catholic University of Korea; J.H. Ju, MD, PhD, Division of Rheumatology, Department of Internal Medicine, St. Mary's Hospital, College of Medicine, The Catholic University of Korea; S.H. Park, MD, PhD, Division of Rheumatology, Department of Internal Medicine, St. Mary's Hospital, and Rheumatism Research Center, College of Medicine, The Catholic University of Korea; J. Lee, MS, Rheumatism Research Center, College of Medicine, The Catholic University of Korea.

Lee, Jaeseon. From the Division of Rheumatology, Department of Internal Medicine, St. Mary's Hospital, College of Medicine, The Catholic University of Korea, Seoul, South Korea.

Lee, Jaeseon. J.H. Koh, MD, Division of Rheumatology, Department of Internal Medicine, St. Mary's Hospital, College of Medicine, The Catholic University of Korea; S.H. Kim, MD, Division of Rheumatology, Department of Internal Medicine, St. Mary's Hospital, College of Medicine, The Catholic University of Korea; S.K. Kwok, MD, PhD; Division of Rheumatology, Department of Internal Medicine, St. Mary's Hospital, and Rheumatism Research Center, College of Medicine, The Catholic University of Korea; J.H. Ju, MD, PhD, Division of Rheumatology, Department of Internal Medicine, St. Mary's Hospital, College of Medicine, The Catholic University of Korea; S.H. Park, MD, PhD, Division of Rheumatology, Department of Internal Medicine, St. Mary's Hospital, and Rheumatism Research Center, College of Medicine, The Catholic University of Korea; J. Lee, MS, Rheumatism Research Center, College of Medicine, The Catholic University of Korea.

Kim, Seo Hwa. From the Division of Rheumatology, Department of Internal Medicine, St. Mary's Hospital, College of Medicine, The Catholic University of Korea, Seoul, South Korea.

Kim, Seo Hwa. J.H. Koh, MD, Division of Rheumatology, Department of Internal Medicine, St. Mary's Hospital, College of Medicine, The Catholic University of Korea; S.H. Kim, MD, Division of Rheumatology, Department of Internal Medicine, St. Mary's Hospital, College of Medicine, The Catholic University of Korea; S.K. Kwok, MD, PhD; Division of Rheumatology, Department of Internal Medicine, St. Mary's Hospital, and Rheumatism Research Center, College of Medicine, The Catholic University of Korea; J.H. Ju, MD, PhD, Division of Rheumatology, Department of Internal Medicine, St. Mary's Hospital, College of Medicine, The Catholic University of Korea; S.H. Park, MD, PhD, Division of Rheumatology, Department of Internal Medicine, St. Mary's Hospital, and Rheumatism Research Center, College of Medicine, The Catholic University of Korea; J. Lee, MS, Rheumatism Research Center, College of Medicine, The Catholic University of Korea.

Kwok, Seung-Ki. From the Division of Rheumatology, Department of Internal Medicine, St. Mary's Hospital, College of Medicine, The Catholic University of Korea, Seoul, South Korea.

Kwok, Seung-Ki. J.H. Koh, MD, Division of Rheumatology, Department of Internal Medicine, St. Mary's Hospital, College of Medicine, The Catholic University of Korea; S.H. Kim, MD, Division of Rheumatology, Department of Internal Medicine, St. Mary's Hospital, College of Medicine, The Catholic University of Korea; S.K. Kwok, MD, PhD; Division of Rheumatology, Department of Internal Medicine, St. Mary's Hospital, and Rheumatism Research Center, College of Medicine, The Catholic University of Korea; J.H. Ju, MD, PhD, Division of Rheumatology, Department of Internal Medicine, St. Mary's Hospital, College of Medicine, The Catholic University of Korea; S.H. Park, MD, PhD, Division of Rheumatology, Department of Internal Medicine, St. Mary's Hospital, and Rheumatism Research Center, College of Medicine, The Catholic University of Korea; J. Lee, MS, Rheumatism Research Center, College of Medicine, The Catholic University of Korea.

Ju, Ji Hyeon. From the Division of Rheumatology, Department of Internal Medicine, St. Mary's Hospital, College of Medicine, The Catholic University of Korea, Seoul, South Korea.

Ju, Ji Hyeon. J.H. Koh, MD, Division of Rheumatology, Department of Internal Medicine, St. Mary's Hospital, College of Medicine, The Catholic University of Korea; S.H. Kim, MD, Division of Rheumatology, Department of Internal Medicine, St. Mary's Hospital, College of Medicine, The Catholic University of Korea; S.K. Kwok, MD, PhD; Division of Rheumatology, Department of Internal Medicine, St. Mary's Hospital, and Rheumatism Research Center, College of Medicine, The Catholic University of Korea; J.H. Ju, MD, PhD, Division of Rheumatology, Department of Internal Medicine, St. Mary's Hospital, College of Medicine, The Catholic University of Korea; S.H. Park, MD, PhD, Division of Rheumatology, Department of Internal Medicine, St. Mary's Hospital, and Rheumatism Research Center, College of Medicine, The Catholic University of Korea; J. Lee, MS, Rheumatism Research Center, College of Medicine, The Catholic University of Korea.

Park, Sung-Hwan. From the Division of Rheumatology, Department of Internal Medicine, St. Mary's Hospital, College of Medicine, The Catholic University of Korea, Seoul, South Korea. rapark@catholic.ac.kr.

Park, Sung-Hwan. J.H. Koh, MD, Division of Rheumatology, Department of Internal Medicine, St. Mary's Hospital, College of Medicine, The Catholic University of Korea; S.H. Kim, MD, Division of Rheumatology, Department of Internal Medicine, St. Mary's Hospital, College of Medicine, The Catholic University of Korea; S.K. Kwok, MD, PhD; Division of Rheumatology, Department of Internal Medicine, St. Mary's Hospital, and Rheumatism Research Center, College of Medicine, The Catholic University of Korea; J.H. Ju, MD, PhD, Division of Rheumatology, Department of Internal Medicine, St. Mary's Hospital, College of Medicine, The Catholic University of Korea; S.H. Park, MD, PhD, Division of Rheumatology, Department of Internal Medicine, St. Mary's Hospital, and Rheumatism Research Center, College of Medicine, The

Catholic University of Korea; J. Lee, MS, Rheumatism Research Center, College of Medicine, The Catholic University of Korea. rapark@catholic.ac.kr.
Abstract
OBJECTIVE: To examine humoral and cellular immune responses induced by a live attenuated herpes zoster (HZ) vaccine in patients with rheumatoid arthritis (RA) compared with osteoarthritis (OA) patients.

METHODS: This was an observational study of a live attenuated HZ vaccine in 41 patients with RA receiving conventional disease-modifying antirheumatic drugs (cDMARD) and/or low-dose glucocorticoids (GC) and in 28 patients with OA. Blood samples were obtained before and at 12 weeks after HZ vaccination. Immunogenicity was assessed using varicella zoster virus (VZV)-specific interferon gamma ELISA and an in-house ELISA. Clinical outcomes, including adverse events, HZ occurrence, and RA flares, were analyzed.

RESULTS: No patients developed vaccination-induced HZ during the followup period (median = 1.6 yrs). The HZ vaccine induced a significant increase in the VZV-specific enzyme-linked immunospot spot-forming units and anti-VZV immunoglobulin G antibodies in patients with RA and OA. The number of spot-forming units was lower in patients with RA than in patients with OA both at baseline and at 12 weeks after vaccination. The disease activity index for patients with RA was similar at baseline and at 12 weeks after vaccination. However, 6 patients with RA (14.6%) experienced a flare during the 12 weeks. Overall, 17 (24.6%) participants reported a mild adverse event such as an injection site reaction (11.6%).

CONCLUSION: The HZ vaccine induced VZV-specific cellular and humoral responses in patients with RA. Although patients with RA showed a weaker vaccine-induced VZV-specific cellular immune response than patients with OA, the vaccine may be considered in patients with RA receiving cDMARD and/or low dose GC.
Publication Type
Journal Article. Observational Study.
Year of Publication
2018

<33>

Unique Identifier

30648228

Title

Interventions to improve vaccine acceptance among rheumatoid arthritis patients: a systematic review.

Source

Clinical Rheumatology. 38(6):1537-1544, 2019 Jun.

VI 1

Status

MEDLINE

Authors

Gosselin Boucher V; Colmegna I; Gemme C; Labbe S; Pelaez S; Lavoie KL.

Authors Full Name

Gosselin Boucher, Vincent; Colmegna, Ines; Gemme, Claudia; Labbe, Sara; Pelaez, Sandra; Lavoie, Kim L.

Institution

Gosselin Boucher, Vincent. Department of Psychology, University of Quebec at Montreal (UQAM), CP 8888, Sucursale Centre-Ville, Montreal, Quebec, H3C 3P8, Canada.

Gosselin Boucher, Vincent. Montreal Behavioural Medicine Centre, Centre Integre Universitaire de sante et services sociaux du Nord-de-l'Ile-de-Montreal (CIUSSS-NIM), Hopital du Sacre-Coeur de Montreal Canada, Montreae, Canada.

Colmegna, Ines. Department of Medicine, Division of Rheumatology, McGill University, Montreal, Canada.

Gemme, Claudia. Department of Psychology, University of Quebec at Montreal (UQAM), CP 8888, Sucursale Centre-Ville, Montreal, Quebec, H3C 3P8, Canada.

Gemme, Claudia. Montreal Behavioural Medicine Centre, Centre Integre Universitaire de sante et services sociaux du Nord-de-l'Ile-de-Montreal (CIUSSS-NIM), Hopital du Sacre-Coeur de Montreal Canada, Montreae, Canada.

Labbe, Sara. Department of Psychology, University of Quebec at Montreal (UQAM), CP 8888, Sucursale Centre-Ville, Montreal, Quebec, H3C 3P8, Canada.

Labbe, Sara. Montreal Behavioural Medicine Centre, Centre Integre Universitaire de sante et services sociaux du Nord-de-l'Ile-de-Montreal (CIUSSS-NIM), Hopital du Sacre-Coeur de Montreal Canada, Montreae, Canada.

Pelaez, Sandra. Department of Educational and Counselling Psychology, McGill University, Montreal, Canada.

Lavoie, Kim L. Department of Psychology, University of Quebec at Montreal (UQAM), CP 8888, Sucursale Centre-Ville, Montreal, Quebec, H3C 3P8, Canada. kiml_lavoie@yahoo.ca.

Lavoie, Kim L. Montreal Behavioural Medicine Centre, Centre Integre Universitaire de sante et services sociaux du Nord-de-l'Ile-de-Montreal (CIUSSS-NIM), Hopital du Sacre-Coeur de Montreal Canada, Montreae, Canada. kiml_lavoie@yahoo.ca.
Comments
Erratum in (EIN)
Abstract
INTRODUCTION/OBJECTIVE: National guidelines emphasize the importance of annual immunization for patients living with rheumatoid arthritis (RA), but vaccination rates remain suboptimal in this population. Evaluating the efficacy of patient and/or provider-targeted interventions to improve vaccination uptake among RA patients could inform practice.

METHODS: We conducted a systematic review (SR) to examine the efficacy of interventions (exposure) aiming to improve vaccination uptake in patients with RA (outcome). English and French language, peer-reviewed interventional studies to improve vaccination rates in RA patients published between 2009 and 2018 were included.

RESULTS: The search yielded a total of 450 records. Five articles met inclusion criteria. All interventions focused on changing provider behavior using some form of vaccination reminder as the primary intervention strategy, though only two studies reported provider prescribing behavior as an outcome (which was 4% and 58%). Overall, studies varied greatly regarding intervention delivery mode (e.g., educational sessions, e-mail reminders, best practice alerts), and behavior change techniques used to encourage providers to prescribe vaccination (e.g., feedback and monitoring, shaping knowledge, self-regulation). For influenza, pneumococcal and herpes zoster, post-intervention (mean 12-16 months follow-up) vaccination rates increased by a mean of 16.6% (+/- 15.4%).

CONCLUSIONS: Interventions to enhance vaccine uptake in RA focused almost exclusively on improving provider prescription of vaccines using reminder-type interventions. Although effective in improving vaccination rates, those studies used heterogeneous interventions and behavior change techniques. Few studies measured provider prescribing behavior as an outcome. Future studies targeting providers should measure relevant provided-related outcomes and their impact on patient outcomes, to determine overall efficacy.
Publication Type
Journal Article. Systematic Review.
Year of Publication
2019

<34>

Unique Identifier

30740105

Title

Targeting Tregs in Juvenile Idiopathic Arthritis and Juvenile Dermatomyositis-Insights From Other Diseases. [Review]

Source

Frontiers in Immunology. 10:46, 2019.

VI 1

Status

MEDLINE

Authors

Hoeppli RE; Pesenacker AM.

Authors Full Name

Hoeppli, Romy E; Pesenacker, Anne M.

Institution

Hoeppli, Romy E. Department of Surgery, British Columbia Children's Hospital Research Institute, University of British Columbia, Vancouver, BC, Canada.

Pesenacker, Anne M. Division of Infection and Immunity, Institute of Immunity and Transplantation, University College London, London, United Kingdom.

Abstract

Regulatory T cells (Tregs) are believed to be dysfunctional in autoimmunity. Juvenile idiopathic arthritis (JIA) and juvenile dermatomyositis (JDM) result from a loss of normal immune regulation in specific tissues such as joints or muscle and skin, respectively. Here, we discuss recent findings in regard to Treg biology in oligo-/polyarticular JIA and JDM, as well as what we can learn about Treg-related disease mechanism, treatment and biomarkers in JIA/JDM from studies of other diseases. We explore the potential use of Treg immunoregulatory markers and gene signatures as biomarkers for disease course and/or treatment success. Further, we discuss how Tregs are affected by several treatment strategies already employed in the therapy of JIA and JDM and by alternative immunotherapies such as anti-cytokine or co-receptor targeting. Finally, we review recent successes in using Tregs as a treatment target with low-dose IL-2 or cellular immunotherapy. Thus, this mini review will highlight our current understanding and identify open

questions in regard to Treg biology, and how recent findings may advance biomarkers and new therapies for JIA and JDM.

Publication Type

Journal Article. Research Support, Non-U.S. Gov't. Review.

Year of Publication

2019

<35>

Unique Identifier

27726761

Title

Anti-TNF Therapy. [Review]

Source

Microbiology Spectrum. 4(4), 2016 08.

VI 1

Status

MEDLINE

Authors

Udalova I; Monaco C; Nanchahal J; Feldmann M.

Authors Full Name

Udalova, Irina; Monaco, Claudia; Nanchahal, Jagdeep; Feldmann, Marc.

Institution

Udalova, Irina. Kennedy Institute of Rheumatology, NDORMS, University of Oxford, Botnar Research Centre, Headington, Oxford OX3 7LD, United Kingdom.

Monaco, Claudia. Kennedy Institute of Rheumatology, NDORMS, University of Oxford, Botnar Research Centre, Headington, Oxford OX3 7LD, United Kingdom.

Nanchahal, Jagdeep. Kennedy Institute of Rheumatology, NDORMS, University of Oxford, Botnar Research Centre, Headington, Oxford OX3 7LD, United Kingdom.

Feldmann, Marc. Kennedy Institute of Rheumatology, NDORMS, University of Oxford, Botnar Research Centre, Headington, Oxford OX3 7LD, United Kingdom.

Abstract

Tumor necrosis factor (TNF) is one of the most important cytokines produced by macrophages. TNF is a very important component of host defense, released very rapidly after all types of

injuries and stimuli. The kinetics of TNF release are short, and so it is perhaps not surprising that prolonged TNF production is associated with pathology. This was first elucidated in rheumatoid arthritis but extends to other chronic inflammatory diseases such as Crohn's disease and psoriasis. In this chapter, the discovery of anti-TNF therapy is reviewed, with its benefit but also its limitations. The potential of anti-TNF therapy in other diseases, e.g., cardiovascular and fibrosis, is discussed, as is the opportunity to define ways of blocking TNF synthesis.

Publication Type

Journal Article. Review.

Year of Publication

2016

<36>

Unique Identifier

24911069

Title

Skin-induced tolerance as a new needle free therapeutic strategy. [Review]

Source

Pharmacological Reports: PR. 66(2):192-7, 2014 Apr.

VI 1

Status

MEDLINE

Authors

Szczepanik M.

Authors Full Name

Szczepanik, Marian.

Institution

Szczepanik, Marian. Department of Medical Biology, Jagiellonian University College of Medicine, Krakow, Poland. Electronic address: mmszczep@cyf-kr.edu.pl.

Abstract

This article summarizes current knowledge about a new subject called "skin induced tolerance". Suppression is induced via epicutaneous (EC) immunization with a protein antigen and is described in Th1, Tc1 and NK mediated contact hypersensitivity (CHS) reactions. The subject of skin-induced suppression is also described in the regulation of experimental models of

autoimmune diseases like experimental autoimmune encephalomyelitis (EAE), collagen induced arthritis (CIA) and inflammatory bowel disease (IBD) and finally in an animal model of graft rejection. The potential clinical use of this approach to regulate human diseases is also discussed. Copyright © 2014 Institute of Pharmacology, Polish Academy of Sciences. Published by Elsevier Urban & Partner Sp. z o.o. All rights reserved.

Publication Type

Journal Article. Research Support, Non-U.S. Gov't. Review.

Year of Publication

2014

<37>

Unique Identifier

28600350

Title

Rheumatoid arthritis and polymyalgia rheumatica occurring after immune checkpoint inhibitor treatment. Annals of the Rheumatic Diseases. 76(10):1747-1750, 2017 Oct.

Authors

Belkhir R; Burel SL; Dunogeant L; Marabelle A; Hollebecque A; Besse B; Leary A; Voisin AL; Pontoizeau C; Coutte L; Pertuiset E; Mouterde G; Fain O; Lambotte O; Mariette X.

Authors Full Name

Belkhir, Rakiba; Burel, Sebastien Le; Dunogeant, Laetitia; Marabelle, Aurelien; Hollebecque, Antoine; Besse, Benjamin; Leary, Alexandra; Voisin, Anne-Laure; Pontoizeau, Clemence; Coutte, Laetitia; Pertuiset, Edouard; Mouterde, Gael; Fain, Olivier; Lambotte, Olivier; Mariette, Xavier.

Institution

Belkhir, Rakiba. Rheumatology, Assistance Publique-Hopitaux de Paris (AP-HP), Hopitaux Universitaires Paris-Sud, Le Kremlin-Bicetre, France.

Burel, Sebastien Le. AP-HP, Hopital Bicetre, Service de Medecine Interne et Immunologie clinique, Le Kremlin-Bicetre, France.

Dunogeant, Laetitia. Service de rhumatologie et medecine interne, Centre Hospitalier du Pays d'Aix, Aix-en-Provence, France.

Marabelle, Aurelien. Departement d'Innovation Therapeutique et Essais Precoces (DITEP), Gustave Roussy Cancer Campus, France.

Hollebecque, Antoine. Departement d'Innovation Therapeutique et Essais Precoces (DITEP), Gustave Roussy Cancer Campus, France.
Besse, Benjamin. Gustave Roussy Cancer Campus, Villejuif; Univ. Paris-Sud, Universite Paris-Saclay, Villejuif, France.
Leary, Alexandra. Gustave Roussy Cancer Campus, Villejuif; Univ. Paris-Sud, Universite Paris-Saclay, Villejuif, France.
Voisin, Anne-Laure. Institut Gustave Roussy, Unite Fonctionnelle de Pharmacovigilance, Villejuif, France.
Pontoizeau, Clemence. Service de medecine nucleaire, Centre Hospitalier de Saint Brieuc, 22000 Saint Brieuc, France.
Coutte, Laetitia. AP-HP, universite Paris-Descartes, hopital Cochin, centre de reference maladies auto-immunes et systemiques rares, service de medecine interne, Paris, France.
Pertuiset, Edouard. Rheumatology Department, Rene Dubos Hospital, Pontoise, France.
Mouterde, Gael. Rheumatology Department, Lapeyronie Hospital and &EA 2415, Montpellier, France.
Fain, Olivier. AP-HP, Service de Medecine Interne, Hopital Saint-Antoine, Universite Paris, France.
Lambotte, Olivier. AP-HP, Hopital Bicetre, Service de Medecine Interne et Immunologie clinique, Le Kremlin-Bicetre, France.
Lambotte, Olivier. CEA, DSV/iMETI, Division d'Immunovirologie, IDMIT, Fontenay-aux-Roses, France.
Mariette, Xavier. Rheumatology, Assistance Publique-Hopitaux de Paris (AP-HP), Hopitaux Universitaires Paris-Sud, Le Kremlin-Bicetre, France.
Mariette, Xavier. Universite Paris Sud, INSERM, Center for Immunology of Viral Infections and Autoimmune Diseases, Le Kremlin-Bicetre, France.
Comments
Comment in (CIN)
Abstract
OBJECTIVES: Immune checkpoint inhibitors (ICIs) targeting cytotoxic T-lymphocyte-associated protein 4 and programmed cell death protein 1 (PD-1) have demonstrated improved survival for multiple cancers. However, these new drug classes have led to increased immune-related adverse events (IrAE). Rheumatic IrAEs have not been well described in clinical trials. We report here cases of rheumatoid arthritis (RA) and polymyalgia rheumatica (PMR) occurring after ICI treatment.

METHODS: This was a retrospective study of patients receiving an ICI in whom symptoms of arthritis or arthralgia developed and revealed a diagnosis of RA or PMR.

RESULTS: In 10 patients who received ICI therapy (all anti-PD-1 or anti-PDL1 antibodies), RA or PMR developed at a median of 1 month (1 to 9) after exposure. No patient had pre-existing rheumatic or autoimmune disease. RA developed in six patients; all six were positive for anti-cyclic citrullinated peptide (anti-CCP) antibodies and four for rheumatoid factor. Anti-CCP antibodies were detected in two out of three patients tested before immunotherapy. Disease-modifying antirheumatic drugs were needed for three patients; the three others received corticosteroids or non-steroid anti-inflammatory drugs. PMR was diagnosed in four patients, all responded to corticosteroids. Despite these IrAEs, immunotherapy was pursued for all but one patient until cancer progression.

CONCLUSIONS: This is the first description of RA occurring after ICI therapy for cancer. PMR can also occur after ICI, particularly after anti-PD-1 therapy. All cases responded to corticosteroids or with immunosuppressive therapy. Collaboration between rheumatologists and oncologists is crucial and could lead to better recognition and care of these patients. Copyright © Article author(s) (or their employer(s) unless otherwise stated in the text of the article) 2017. All rights reserved. No commercial use is permitted unless otherwise expressly granted.
Publication Type
Journal Article.
Year of Publication
2017

<38>
Unique Identifier
31464681
Title
B cell depletion with rituximab in the treatment of primary Sjogren's syndrome: what have we learnt?. [Review]
Source
Clinical & Experimental Rheumatology. 37 Suppl 118(3):217-224, 2019 May-Jun.
VI 1
Status
MEDLINE

Authors
Grigoriadou S; Chowdhury F; Pontarini E; Tappuni A; Bowman SJ; Bombardieri M.
Authors Full Name
Grigoriadou, Sofia; Chowdhury, Farzana; Pontarini, Elena; Tappuni, Anwar; Bowman, Simon J; Bombardieri, Michele.
Institution
Grigoriadou, Sofia. Centre for Experimental Medicine and Rheumatology, William Harvey Research Institute, Queen Mary University of London, UK.
Chowdhury, Farzana. Centre for Experimental Medicine and Rheumatology, William Harvey Research Institute, and Department of Oral Medicine, Institute of Dentistry, Bart's and The London School of Medicine and Dentistry, Queen Mary University of London, UK.
Pontarini, Elena. Centre for Experimental Medicine and Rheumatology, William Harvey Research Institute, Queen Mary University of London, UK.
Tappuni, Anwar. Department of Oral Medicine, Institute of Dentistry, Bart's and The London School of Medicine and Dentistry, Queen Mary University of London, UK.
Bowman, Simon J. Rheumatology Research Group, Institute of Inflammation and Ageing, University of Birmingham, and Rheumatology Department, University Hospitals Birmingham NHS Foundation Trust, Birmingham, UK.
Bombardieri, Michele. Centre for Experimental Medicine and Rheumatology, William Harvey Research Institute, Queen Mary University of London, UK. m.bombardieri@qmul.ac.uk.
Abstract
Despite the well-established role of B cells in the pathogenesis of primary Sjogren's syndrome (pSS), the beneficial role of B-cell depletion therapy with rituximab remains elusive in this condition, contrary to other autoimmune diseases. Although early, small-scale studies showed promising results, two recent large randomised controlled trials did not meet their primary end-points. It is evident from most trials that rituximab has a positive impact on B-cell numbers and activity, both in the peripheral blood and in salivary glands, but clinical outcomes vary among studies. We review here the evidence to date of B-cell depletion in pSS, analysing the underlying causes for the discrepancies in different studies and their limitations. We also discuss the potential use of peripheral and salivary gland biomarkers for patient stratification and targeted patient selection. Overall, rituximab remains a plausible treatment for pSS provided future studies address the shortfalls that emerged from our current knowledge of the use of B-cell depletion in this condition.
Publication Type
Journal Article. Review.
Year of Publication
2019

<39>

Unique Identifier

25891013

Title

The anergy induction of M3 muscarinic acetylcholine receptor-reactive CD4+ T cells suppresses experimental sialadenitis-like Sjogren's syndrome.

Source

Arthritis & Rheumatology. 67(8):2213-25, 2015 May.

VI 1

Status

MEDLINE

Authors

Asashima H; Tsuboi H; Takahashi H; Hirota T; Iizuka M; Kondo Y; Matsui M; Matsumoto I; Sumida T.

Authors Full Name

Asashima, Hiromitsu; Tsuboi, Hiroto; Takahashi, Hiroyuki; Hirota, Tomoya; Iizuka, Mana; Kondo, Yuya; Matsui, Minoru; Matsumoto, Isao; Sumida, Takayuki.

Institution

Asashima, Hiromitsu. University of Tsukuba, Tsukuba, Japan.

Tsuboi, Hiroto. University of Tsukuba, Tsukuba, Japan.

Takahashi, Hiroyuki. University of Tsukuba, Tsukuba, Japan.

Hirota, Tomoya. University of Tsukuba, Tsukuba, Japan.

Iizuka, Mana. University of Tsukuba, Tsukuba, Japan.

Kondo, Yuya. University of Tsukuba, Tsukuba, Japan.

Matsui, Minoru. Fureai Higashitotsuka Hospital, Yokohama, Japan.

Matsumoto, Isao. University of Tsukuba, Tsukuba, Japan.

Sumida, Takayuki. University of Tsukuba, Tsukuba, Japan.

Abstract

OBJECTIVE: Autoreactive CD4+ T cells are involved in the pathogenesis of Sjogren's syndrome (SS). The aim of the present study was to clarify the dominant T cell epitopes of M3 muscarinic acetylcholine receptor (M3R) and to establish a new antigen-specific therapy for SS using an experimental mouse model.

METHODS: Production of cytokines from M3R-reactive CD4+ T cells, after culture with various M3R peptides, was analyzed by enzyme-linked immunosorbent assay. Adoptive cell transfer was performed using splenocytes from M3R(-/-) mice that were immunized with M3R peptides or phosphate buffered saline plus H37Ra as a control. Rag1(-/-) mice were inoculated with the splenocytes and examined for the development of sialadenitis. Altered peptide ligands (APLs) of the T cell epitopes, with substitutions in amino acid residues at T cell receptor contact sites, were synthesized, and the ability of the APLs to suppress sialadenitis was evaluated. The mechanisms underlying such effects were assessed.

RESULTS: CD4+ M3R-reactive T cells produced interleukin-17 (IL-17) and interferon-gamma (IFNgamma) in response to the N-terminal 1 (N1) and 1st extracellular loop peptides of M3R, and Rag1(-/-) mice that received N1- and/or 1st peptide-immunized splenocytes developed sialadenitis. Among the designed APLs, N1-APL7 (N->S at amino acid 15) significantly suppressed IFNgamma production in vitro, and also suppressed sialadenitis in vivo. Levels of early growth response 2 in CD4+ T cells from the cervical lymph nodes of N1-APL7-treated mice were significantly higher than those of control mice, and cell proliferation was reversed by administration of exogenous IL-2. Levels of the anergy-related molecules itchy homolog E3 ubiquitin-protein ligase, Casitas B-lineage lymphoma b, gene related to anergy in lymphocytes, and Deltex-1 were significantly higher in CD4+ T cells cultured with N1-APL7.

CONCLUSION: The major T cell epitopes were from the N1 and 1st peptide regions. Moreover, N1-APL7, selected as the antagonistic APL in vitro, also suppressed sialadenitis through the induction of anergy. This is a potentially useful strategy for regulating pathogenic T cell infiltration in SS. Copyright © 2015, American College of Rheumatology.

Publication Type

Journal Article. Research Support, Non-U.S. Gov't.

Year of Publication

2015

<40>

Unique Identifier

31464670

Title

Sicca/Sjogren's syndrome triggered by PD-1/PD-L1 checkpoint inhibitors. Data from the International ImmunoCancer Registry (ICIR).

Source

Clinical & Experimental Rheumatology. 37 Suppl 118(3):114-122, 2019 May-Jun.

VI 1

Status

MEDLINE

Authors

Ramos-Casals M; Maria A; Suarez-Almazor ME; Lambotte O; Fisher BA; Hernandez-Molina G; Guilpain P; Pundole X; Flores-Chavez A; Baldini C; Bingham Iii CO; Brito-Zeron P; Gottenberg JE; Kostine M; Radstake TRD; Schaeverbeke T; Schulze-Koops H; Calabrese L; Khamashta MA; Mariette X; ICIR.

Authors Full Name

Ramos-Casals, Manuel; Maria, Alexandre; Suarez-Almazor, Maria E; Lambotte, Olivier; Fisher, Benjamin A; Hernandez-Molina, Gabriela; Guilpain, Philippe; Pundole, Xerxes; Flores-Chavez, Alejandra; Baldini, Chiara; Bingham Iii, Clifton O; Brito-Zeron, Pilar; Gottenberg, Jacques-Eric; Kostine, Marie; Radstake, Timothy R D; Schaeverbeke, Thierry; Schulze-Koops, Hendrik; Calabrese, Leonard; Khamashta, Munther A; Mariette, Xavier; ICIR.

Institution

Ramos-Casals, Manuel. Department of Autoimmune Diseases, ICMiD, Barcelona; Sjogren Syndrome Research Group (AGAUR), Laboratory of Autoimmune Diseases Josep Font, IDIBAPS-CELLEX, Barcelona; and Department of Medicine, University of Barcelona, Hospital Clinic, Barcelona, Spain. mramos@clinic.cat

Maria, Alexandre. Department of Internal Medicine Multi-Organic Diseases, Local Referral Center for Autoimmune Diseases, Saint Eloi Hospital, Montpellier University Hospital, France.

Suarez-Almazor, Maria E. Section of Rheumatology and Clinical Immunology, Department of General Internal Medicine, The University of Texas MD Anderson Cancer Center, Houston, TX, USA.

Lambotte, Olivier. APHP Medecine Interne/Immunologie Clinique, Hopital Bicetre, Paris, and Universite Paris Sud - INSERM U1184 - CEA, Immunology of Viral Infections and Autoimmune Diseases, IDMIT Department, IBFJ, Fontenay-aux-Roses & Le Kremlin- Bicetre, France.

Fisher, Benjamin A. Institute of Inflammation and Ageing, University of Birmingham; and National Institute of Health Research Birmingham Biomedical Research Centre and Department of Rheumatology, University Hospitals Birmingham NHS Foundation Trust, Birmingham, UK.

Hernandez-Molina, Gabriela. Immunology and Rheumatology Department, Instituto Nacional de Ciencias Medicas y Nutricion Salvador Subiran, Mexico City, Mexico.

Guilpain, Philippe. Department of Internal Medicine Multi-Organic Diseases, Local Referral Center for Autoimmune Diseases, Saint Eloi Hospital, Montpellier University Hospital, France.
Pundole, Xerxes. Section of Rheumatology and Clinical Immunology, Department of General Internal Medicine, The University of Texas MD Anderson Cancer Center, Houston, TX, USA.
Flores-Chavez, Alejandra. Department of Autoimmune Diseases, ICMiD, Barcelona, Spain.
Baldini, Chiara. Rheumatology Unit, Department of Clinical and Experimental Medicine, University of Pisa, Italy.
Bingham Iii, Clifton O. Department of Rheumatology, The Johns Hopkins University School of Medicine, Baltimore, MD, USA.
Brito-Zeron, Pilar. Department of Autoimmune Diseases, ICMiD, Barcelona, and Department of Internal Medicine, Hospital CIMA-Sanitas, Barcelona, Spain.
Gottenberg, Jacques-Eric. Dept. of Rheumatology, Strasbourg University Hospital, National Centre For Rare Systemic Autoimmune Diseases, CNRS, Institut de Biologie Moleculaire et Cellulaire, Immunologie, Immunopathologie et Chimie Therapeutique, University of Strasbourg, France.
Kostine, Marie. Department of Rheumatology, Centre Hospitalier Universitaire, Bordeaux, France.
Radstake, Timothy R D. Department of Rheumatology/Clinical Immunology, University Medical Center Utrecht, The Netherlands.
Schaeverbeke, Thierry. Department of Rheumatology, Centre Hospitalier Universitaire, Bordeaux, France.
Schulze-Koops, Hendrik. Department of Rheumatology/Clinical Immunology, Ludwig-Maximilians-University Munich, Germany.
Calabrese, Leonard. Department of Rheumatic/Immunologic Diseases, Cleveland Clinic University, Cleveland, OH, USA.
Khamashta, Munther A. Lupus Clinic, Rheumatology Department, Dubai Hospital, UAE.
Mariette, Xavier. Center for Immunology of Viral Infections and Autoimmune Diseases, Assistance Publique - Hopitaux de Paris, Hopitaux Universitaires Paris-Sud, Le Kremlin-Bicetre, Universite Paris Sud, INSERM, Paris, France.
Abstract
OBJECTIVES: To analyse the worldwide occurrence of sicca/Sjogren's (SS) syndrome associated with the use of immune checkpoint inhibitors (ICI) in patients with cancer.

METHODS: The ImmunoCancer International Registry (ICIR) is a Big Data-Sharing multidisciplinary network composed by 40 specialists in Rheumatology, Internal Medicine, Immunology and Oncology from 18 countries focused on the clinical and basic research of the immune-related adverse events (irAEs) related to cancer immunotherapies. For this study,

patients who were investigated for a clinical suspicion of SS after being exposed to ICI were included.

RESULTS: We identified 26 patients (11 women and 15 men, with a mean age at diagnosis of 63.57 years). Underlying cancer included lung (n=12), renal (n=7), melanoma (n=4), and other (n=3) neoplasia. Cancer immunotherapies consisted of monotherapy (77%) and combined regimens (23%). In those patients receiving monotherapy, all patients were treated with PD-1/PD-L1 inhibitors (nivolumab in 9, pembrolizumab in 7 and durvalumab in 4); no cases associated with CTLA-4 inhibitors were identified. The main SS-related features consisted of dry mouth in 25 (96%) patients, dry eye in 17 (65%), abnormal ocular tests in 10/16 (62%) and abnormal oral diagnostic tests in 12/14 (86%) patients. Minor salivary gland biopsy was carried out in 15 patients: histopathological findings consisted of mild chronic sialadenitis in 8 (53%) patients and focal lymphocytic sialadenitis in the remaining 7 (47%); a focus score was measured in 5 of the 6 patients (mean of 1.8, range 1-4). Immunological markers included positive ANA in 13/25 (52%), anti-Ro/ SS-A in 5/25 (20%), RF in 2/22 (9%), anti-La/SS-B in 2/25 (8%), low C3/C4 levels in 1/17 (6%) and positive cryoglobulins in 1/10 (10%). Classification criteria for SS were fulfilled by 10 (62%) out of 16 patients in whom the two key classificatory features were carried out. Among the 26 patients, there were only 3 (11%) who presented exclusively with sicca syndrome without organ-specific autoimmune manifestations. Therapeutic management included measures directed to treat sicca symptoms and therapies against autoimmune-mediated manifestations (glucocorticoids in 42%, second/third-line therapies in 31%); therapeutic response for systemic features was observed in 8/11 (73%). No patient died due to autoimmune involvement.

CONCLUSIONS: Patients with Sjogren's syndrome triggered by ICI display a very specific profile different from that reported in idiopathic primary SS, including more frequent occurrence in men, a higher mean age, a predominant immunonegative serological profile, and a notable development of organ-specific autoimmune involvement in spite of the poor immunological profile. The close association found between sicca/Sjogren's syndrome and primarily PD-1 blockade requires further specific investigation.
Publication Type
Journal Article.
Year of Publication
2019

<41>

Unique Identifier

31176871

Title

Rheumatic immune related adverse events in patients treated with checkpoint inhibitors for immunotherapy of cancer. [Review]

Source

Autoimmunity Reviews. 18(8):805-813, 2019 Aug.

VI 1

Status

MEDLINE

Authors

Sebastiani GD; Scirocco C; Galeazzi M.

Authors Full Name

Sebastiani, Gian Domenico; Scirocco, Chiara; Galeazzi, Mauro.

Institution

Sebastiani, Gian Domenico. Rheumatology Unit, Azienda Ospedaliera San Camillo - Forlanini, Rome, Italy. Electronic address: GSebastiani@scamilloforlanini.rm.it.

Scirocco, Chiara. Rheumatology Unit, Azienda Ospedaliera San Camillo - Forlanini, Rome, Italy. Electronic address: chiascir@hotmail.it.

Galeazzi, Mauro. Rheumatology Institute, University of Siena, Italy.

Abstract

Immune checkpoints are small molecules expressed by immune cells that play critical roles in maintaining immune homeostasis. Immune checkpoint inhibitors (ICPIs) are new cancer drugs that target self-tolerance pathways exploited by tumors to escape immune destruction, such as cytotoxic T-lymphocyte antigen 4 (CTLA-4) and programmed cell death 1 (PD-1) or its ligand (PD-L1). Several ICPIs have been approved by Food and Drug Administration, increasing overall survival with different cancers. However, their use can determine development of many different inflammatory side effects, that are defined immune-related adverse effects (irAEs); among others, rheumatological irAEs can develop in these patients. Currently, we have limited data about these adverse effects; particularly, few evidence come from clinical trials about patients with pre-existing autoimmune diseases because they were excluded from them. Therefore we analysed the existing scientific literature dealing with this issue, in order to answer to different clinical questions. According to all reviewed data, rheumatological irAEs are not infrequent, in both previously diseased and undiseased patients, but they are often mild and reversible. Close monitoring and interdisciplinary management and monitoring is necessary in order to ensure best

care. Many questions remain unanswered or not completely answered; further data are necessary to implement our knowledge in this field and to standardize and optimize clinical practice. Copyright © 2019 Elsevier B.V. All rights reserved.

Publication Type

Journal Article. Review.

Year of Publication

2019

<42>

Unique Identifier

30959220

Title

The pipeline of targeted therapies under clinical development for primary Sjogren's syndrome: A systematic review of trials.

Source

Autoimmunity Reviews. 18(6):576-582, 2019 Jun.

VI 1

Status

MEDLINE

Authors

Felten R; Scher F; Sibilia J; Gottenberg JE; Arnaud L.

Authors Full Name

Felten, Renaud; Scher, Florence; Sibilia, Jean; Gottenberg, Jacques-Eric; Arnaud, Laurent.

Institution

Felten, Renaud. Service de rhumatologie, Hopitaux Universitaires de Strasbourg, F-67000 Strasbourg, France.; Laboratoire d'Immunologie, Immunopathologie et Chimie Therapeutique, Institut de Biologie Moleculaire et Cellulaire (IBMC), CNRS UPR3572, France; RESO : Centre de Reference des Maladies Autoimmunes Systemiques Rares Est Sud-Ouest, France.

Scher, Florence. Service de Pharmacie-Sterilisation, Hopitaux Universitaires de Strasbourg, France.

Sibilia, Jean. Service de rhumatologie, Hopitaux Universitaires de Strasbourg, F-67000 Strasbourg, France.; RESO : Centre de Reference des Maladies Autoimmunes Systemiques

Rares Est Sud-Ouest, France; INSERM UMR_S1109, Universite de Strasbourg, F-67000 Strasbourg, France.
Gottenberg, Jacques-Eric. Service de rhumatologie, Hopitaux Universitaires de Strasbourg, F-67000 Strasbourg, France.; Laboratoire d'Immunologie, Immunopathologie et Chimie Therapeutique, Institut de Biologie Moleculaire et Cellulaire (IBMC), CNRS UPR3572, France; RESO : Centre de Reference des Maladies Autoimmunes Systemiques Rares Est Sud-Ouest, France.
Arnaud, Laurent. Service de rhumatologie, Hopitaux Universitaires de Strasbourg, F-67000 Strasbourg, France.; RESO : Centre de Reference des Maladies Autoimmunes Systemiques Rares Est Sud-Ouest, France; INSERM UMR_S1109, Universite de Strasbourg, F-67000 Strasbourg, France. Electronic address: Laurent.arnaud@chru-strasbourg.fr.
Abstract
To date, no immunomodulatory drug has proved efficacious in primary Sjogren's syndrome (pSS). In pSS, difficulties in drug efficacy assessment is related to the large spectrum of clinical involvements (glandular/extraglandular involvement), to the lack of correlation between symptoms of dryness and glandular function assessed by objective measurements, as well as between symptoms and systemic complications of the disease. Severe organ manifestations are generally treated by off-label therapies in accordance with current practice and guidelines for Systemic Lupus Erythematosus or other connective-tissue diseases. Despite a much greater understanding of the pathogenesis of pSS, modern drug development has resulted in no approval of therapy so far. In this study, we performed a systematic review of all targeted therapies under clinical development in pSS, in 17 main online registries of clinical trials. Our search identified 264 trials, from which 25 targeted therapies for pSS were included. The molecules under current clinical development for pSS target B cells (n=4), T cells or T/B cells costimulation (n=5), inflammatory cytokines or chemokines and their receptors (n=5), intracellular signalling pathways (n=7) and various other targets identified in pSS (n=4). The current drug development pipeline in pSS may lead to valuable strategies for the treatment of this currently difficult-to-treat disease. Copyright © 2019. Published by Elsevier B.V.
Publication Type
Journal Article. Systematic Review.
Year of Publication
2019

<43>

Unique Identifier

30946325

Title

Risk of Parkinson disease in Sjogren syndrome administered ineffective immunosuppressant therapies: A nationwide population-based study.

Source

Medicine. 98(14):e14984, 2019 Apr.

VI 1

Status

MEDLINE

Authors

Ju UH; Liu FC; Lin CS; Huang WY; Lin TY; Shen CH; Chou YC; Lin CL; Lin KT; Kao CH; Chen CH; Yang TY.

Authors Full Name

Ju, Uei-Han; Liu, Feng-Cheng; Lin, Chin-Sheng; Huang, Wen-Yen; Lin, Te-Yu; Shen, Chih-Hao; Chou, Yu-Ching; Lin, Cheng-Li; Lin, Kuen-Tze; Kao, Chia-Hung; Chen, Chao-Hsien; Yang, Tse-Yen.

Institution

Ju, Uei-Han. Division of Rheumatology/Immunology and Allergy.

Liu, Feng-Cheng. Division of Rheumatology/Immunology and Allergy.

Lin, Chin-Sheng. Division of Cardiology.

Huang, Wen-Yen. Department of Radiation Oncology.

Lin, Te-Yu. Department of Radiation Oncology.

Lin, Te-Yu. Division of Infectious disease, Department of Internal Medicine.

Shen, Chih-Hao. Division of Pulmonary and Critical Care Medicine, Department of Internal Medicine, Tri-Service General Hospital, National Defense Medical Center, Taipei.

Chou, Yu-Ching. School of Public Health, National Defense Medical Center, Taipei.

Lin, Cheng-Li. College of Medicine, China Medical University.

Lin, Cheng-Li. Management Office for Health Data, China Medical University Hospital.

Lin, Kuen-Tze. Department of Radiation Oncology.

Kao, Chia-Hung. Department of Nuclear Medicine and PET Center, China Medical University Hospital.

Kao, Chia-Hung. Graduate Institute of Clinical Medical Science and School of Medicine, College of Medicine, China Medical University.

Kao, Chia-Hung. Department of Bioinformatics and Medical Engineering, Asia University.

Chen, Chao-Hsien. Department of Medical Laboratory Science and Biotechnology, China Medical University, Taichung.
Yang, Tse-Yen. Department of Medical Research, China Medical University HsinChu Hospital, HsinChu County, China Medical University.
Yang, Tse-Yen. Department of Medical Research, China Medical University Hospital, China Medical University, Taichung.
Yang, Tse-Yen. Molecular and Genomic Epidemiology Center, China Medical University Hospital, Taichung, Taiwan.
Yang, Tse-Yen. Division of Nephrology, Department of Internal Medicine, Changhua Christian Hospital, Changhua, Taiwan.
Abstract
To determine the incidence and risk of Parkinson disease (PD) in patients with Sjogren syndrome (SS) according to a nationwide population-based database. In total, 12,640 patients in the SS cohort and 50,560 in the non-SS cohort were enrolled from Taiwan's National Health Insurance Research Database from 2000 to 2010. We used the Cox multivariable proportional hazards model to determine the risk factors for PD in the SS cohort. We observed an increased incidence of PD in patients with SS, with a crude hazard ratio (HR) of 1.40 and an adjusted HR (aHR) of 1.23. The cumulative incidence of PD was 1.95% higher in the SS cohort than in the non-SS cohort. The SS cohort had an elevated HR under medication use, namely cevimeline and pilocarpine (crude HR, 1.28), hydroxychloroquine (crude HR, 1.43; aHR, 1.46), and methylprednisolone (crude HR, 2.21; aHR, 1.49). Patients receiving other non-hydroxychloroquine immunosuppressant therapies had a lower risk (aHR, 0.86) of PD. Furthermore, patients with SS aged 20 to 49 years had a 1.93-fold higher risk of PD than did those without SS (aHR, 1.93). The risk of PD was higher (aHR, 2.20) in patients with SS without comorbidities than in those with comorbidities. The aHR of PD significantly increased when the follow-up period exceeded 9 years (aHR, 1.93). We determined an increased risk of PD in patients with SS. Further investigation is warranted to determine the possible underlying mechanisms and the potential role of non-hydroxychloroquine immunosuppressants in ameliorating PD.
Publication Type
Journal Article. Observational Study.
Year of Publication
2019

<44>

Unique Identifier

30915372

Title

Efficient Therapeutic Function and Mechanisms of Human Polyclonal CD8+CD103+Foxp3+ Regulatory T Cells on Collagen-Induced Arthritis in Mice.

Source

Journal of Immunological Research. 2019:8575407, 2019.

VI 1

Status

MEDLINE

Authors

Sun J; Yang Y; Huo X; Zhu B; Li Z; Jiang X; Xie R; Gao L; Sun Y; Fan H; Zhu Y; Yang J.

Author NameID

Sun, Juan; ORCID: https://orcid.org/0000-0001-6352-0589

Yang, Yiming; ORCID: https://orcid.org/0000-0001-9189-7121

Huo, Xiaona; ORCID: https://orcid.org/0000-0003-3128-8089

Zhu, Beibei; ORCID: https://orcid.org/0000-0002-5265-3412

Li, Zhenhua; ORCID: https://orcid.org/0000-0002-1075-0214

Jiang, Xueyu; ORCID: https://orcid.org/0000-0002-4857-448X

Xie, Rufeng; ORCID: https://orcid.org/0000-0002-6900-7417

Gao, Li; ORCID: https://orcid.org/0000-0002-5757-2830

Sun, Ying; ORCID: https://orcid.org/0000-0002-3104-4394

Fan, Huahua; ORCID: https://orcid.org/0000-0002-7425-683X

Zhu, Yongming; ORCID: https://orcid.org/0000-0003-4952-4393

Yang, Jie; ORCID: https://orcid.org/0000-0003-3031-0504

Authors Full Name

Sun, Juan; Yang, Yiming; Huo, Xiaona; Zhu, Beibei; Li, Zhenhua; Jiang, Xueyu; Xie, Rufeng; Gao, Li; Sun, Ying; Fan, Huahua; Zhu, Yongming; Yang, Jie.

Institution

Sun, Juan. Institute of Biomedical Sciences, School of Life Sciences, East China Normal University, Shanghai 200241, China.

Sun, Juan. Blood Engineering Laboratory, Shanghai Blood Center, Shanghai 200051, China.

Yang, Yiming. Blood Engineering Laboratory, Shanghai Blood Center, Shanghai 200051, China.

Huo, Xiaona. Institute of Biomedical Sciences, School of Life Sciences, East China Normal University, Shanghai 200241, China.
Huo, Xiaona. Blood Engineering Laboratory, Shanghai Blood Center, Shanghai 200051, China.
Zhu, Beibei. Institute of Biomedical Sciences, School of Life Sciences, East China Normal University, Shanghai 200241, China.
Zhu, Beibei. Blood Engineering Laboratory, Shanghai Blood Center, Shanghai 200051, China.
Li, Zhenhua. Institute of Biomedical Sciences, School of Life Sciences, East China Normal University, Shanghai 200241, China.
Li, Zhenhua. Blood Engineering Laboratory, Shanghai Blood Center, Shanghai 200051, China.
Jiang, Xueyu. Blood Engineering Laboratory, Shanghai Blood Center, Shanghai 200051, China.
Xie, Rufeng. Blood Engineering Laboratory, Shanghai Blood Center, Shanghai 200051, China.
Gao, Li. Blood Engineering Laboratory, Shanghai Blood Center, Shanghai 200051, China.
Sun, Ying. Blood Engineering Laboratory, Shanghai Blood Center, Shanghai 200051, China.
Fan, Huahua. Blood Engineering Laboratory, Shanghai Blood Center, Shanghai 200051, China.
Zhu, Yongming. Blood Engineering Laboratory, Shanghai Blood Center, Shanghai 200051, China.
Yang, Jie. Blood Engineering Laboratory, Shanghai Blood Center, Shanghai 200051, China.
Abstract
Objective: To investigate the potential therapeutic effect in a rheumatoid arthritis model of stable human CD8+ regulatory T cells (hCD8+Tregs) induced by TGF-beta1 and rapamycin (RAPA) in vitro.

Methods: Human CD8+T cells were isolated from human peripheral blood mononuclear cells and induced/expanded with TGF-beta1 and RAPA along with anti-CD3/28 beads and IL-2 in vitro and harvested as hCD8+Tregs. The phenotypes, suppressive characteristics, and stability of the hCD8+Tregs in an inflammatory microenvironment were examined in vitro. Human CD8+Tregs were transfused into an acollagen-induced arthritis (CIA) mouse model, and their therapeutic effects and related mechanisms were investigated.

Results: Human CD8+Tregs induced by TGF-beta1/RAPA showed high expression of Foxp3 and CD103, exhibited vigorous suppression ability, and were stable in inflammatory microenvironments. In CIA mice, the clinical scores, levels of anti-collagen IgG antibody, and cartilage destruction were significantly reduced after adoptive transfusion with hCD8+Tregs. Moreover, hCD8+Treg treatment significantly reduced the number of Th17 cells, increased the number of CD4+IFN-gamma +T cells, and produced self CD4+Foxp3+Tregs in vivo. In an in vitro cell coculture assay, hCD8+Tregs significantly inhibited mouse CD4+ effector T cell proliferation,

induced mouse CD4+Foxp3+Treg and CD4+IFN-gamma +Th1 cell production, reduced Th17 cell development, and downregulated CD80/86 expression on mature DCs (mDCs).

Conclusion: TGF-beta1/RAPA can induce hCD8+Tregs with stable suppressive characteristics, which could significantly alleviate the severity of CIA based on their stable suppressive ability in an inflammatory microenvironment and further influence the function of other downstream cell subtypes. Human CD8+Tregs might be a therapeutic strategy for rheumatoid arthritis.

Publication Type

Journal Article.

Year of Publication

2019

<45>

Unique Identifier

30856393

Title

T-cells interact with B cells, dendritic cells, and fibroblast-like synoviocytes as hub-like key cells in rheumatoid arthritis. [Review]

Source

International Immunopharmacology. 70:428-434, 2019 May.

VI 1

Status

MEDLINE

Authors

Hu XX; Wu YJ; Zhang J; Wei W.

Authors Full Name

Hu, Xiao-Xi; Wu, Yu-Jing; Zhang, Jing; Wei, Wei.

Institution

Hu, Xiao-Xi. Institute of Clinical Pharmacology, Anhui Medical University, Key Laboratory of Anti-inflammatory and Immune Medicine, Ministry of Education, Anhui Collaborative Innovation Center of Anti-inflammatory and Immune Medicine, Hefei, 230032, China.

Wu, Yu-Jing. Institute of Clinical Pharmacology, Anhui Medical University, Key Laboratory of Anti-inflammatory and Immune Medicine, Ministry of Education, Anhui Collaborative Innovation Center of Anti-inflammatory and Immune Medicine, Hefei, 230032, China.
Zhang, Jing. Institute of Clinical Pharmacology, Anhui Medical University, Key Laboratory of Anti-inflammatory and Immune Medicine, Ministry of Education, Anhui Collaborative Innovation Center of Anti-inflammatory and Immune Medicine, Hefei, 230032, China.
Wei, Wei. Institute of Clinical Pharmacology, Anhui Medical University, Key Laboratory of Anti-inflammatory and Immune Medicine, Ministry of Education, Anhui Collaborative Innovation Center of Anti-inflammatory and Immune Medicine, Hefei, 230032, China. Electronic address: wwei@ahmu.edu.cn.
Abstract
Rheumatoid arthritis (RA) is a chronic inflammatory synovitis-based systemic disease characterized by invasive joint inflammation and synovial hyperplasia, which can lead to arthrentasis and defunctionalization. Previous research has shown that T cells, B cells, dendritic cells (DCs), and fibroblast-like synoviocytes (FLSs) play vital roles in the regulation of RA. Both T follicular helper (Tfh) cells and helper T (Th) 17 cells play immunomodulatory roles in RA. Moreover, interleukin-23 (IL-23), and IL-17 are vital to the pathogenesis of RA. T cells behave as a hub, in that B cells, DCs, and FLSs can interact with T cells to inhibit their activation and interfere with the process of RA. T cells cooperate with B cells, DCs, and FLSs to maintain the stability of the immune system under physiological conditions. However, under pathological conditions, the balance is disrupted, and the interaction of T cells with other cells may intensify disease progression. This review focuses on the interaction of T cells with B cells, DCs, and FLSs in different tissues and organs of RA patients and animal models, and highlight that the interplay between immune cells may underline the unique function of T cells and the application prospect of targeting T cell treatment for RA. Copyright © 2019 Elsevier B.V. All rights reserved.
Publication Type
Journal Article. Review.
Year of Publication
2019

<46>
Unique Identifier
30844552

Title

Immunoglobulin replacement for secondary immunodeficiency after B-cell targeted therapies in autoimmune rheumatic disease: Systematic literature review.

Source

Autoimmunity Reviews. 18(5):535-541, 2019 May.

VI 1

Status

MEDLINE

Authors

Wijetilleka S; Mukhtyar C; Jayne D; Ala A; Bright P; Chinoy H; Harper L; Kazmi M; Kiani-Alikhan S; Li C; Misbah S; Oni L; Price-Kuehne F; Salama A; Workman S; Wrench D; Karim MY.

Authors Full Name

Wijetilleka, Sonali; Mukhtyar, Chetan; Jayne, David; Ala, Aftab; Bright, Philip; Chinoy, Hector; Harper, Lorraine; Kazmi, Majid; Kiani-Alikhan, Sorena; Li, Charles; Misbah, Siraj; Oni, Louise; Price-Kuehne, Fiona; Salama, Alan; Workman, Sarita; Wrench, David; Karim, Mohammed Yousuf.

Institution

Wijetilleka, Sonali. Frimley Health NHS Foundation Trust, Portsmouth Rd, Frimley, UK. Electronic address: sonaliwijetilleka@doctors.org.uk.

Mukhtyar, Chetan. Department of Rheumatology, Norfolk and Norwich University Hospitals NHS Foundation Trust, Norwich, Norfolk, UK. Electronic address: chetan.mukhtyar@nnuh.nhs.uk.

Jayne, David. Department of Medicine, University of Cambridge School of Clinical Medicine, Cambridge, UK. Electronic address: dj106@cam.ac.uk.

Ala, Aftab. Department of Gastroenterology and Hepatology, Royal Surrey County Hospital, Guildford, UK; Department of Clinical and Experimental Medicine, University of Surrey, Guildford, UK. Electronic address: aftabala@nhs.net.

Bright, Philip. Department of Immunology, North Bristol NHS Trust, Bristol, UK. Electronic address: phil.bright@nhs.net.

Chinoy, Hector. Department of Rheumatology, Salford Royal NHS Foundation Trust, Manchester Academic Health Science Centre, Salford, UK. Electronic address: Hector.Chinoy@manchester.ac.uk.

Harper, Lorraine. Department of Nephrology, Institute of Clinical Sciences, College of Medical and Dental Science, University of Birmingham, Birmingham, UK. Electronic address: l.harper@bham.ac.uk.

Kazmi, Majid. Department of Haematology, Guy's and St Thomas' NHS Foundation Trust, London, UK. Electronic address: majid.kazmi@gstt.nhs.uk.

Kiani-Alikhan, Sorena. Department of Immunology, Barts and The London NHS Trust, London, UK. Electronic address: skiani@nhs.net.

Li, Charles. Department of Rheumatology, Royal Surrey County Hospital, Guildford, UK. Electronic address: charles.li@nhs.net.

Misbah, Siraj. Department of Immunology, Oxford University Hospitals, Oxford, UK. Electronic address: siraj.misbah@ouh.nhs.uk.

Oni, Louise. Department of Paediatric Nephrology, Alder Hey Children's NHS Foundation Trust Hospital, Liverpool, UK. Electronic address: Louise.Oni@liverpool.ac.uk.

Price-Kuehne, Fiona. Department of Paediatrics, University of Cambridge School of Clinical Medicine, Cambridge, UK. Electronic address: fep30@cam.ac.uk.

Salama, Alan. Department of Nephrology, University College London Centre for Nephrology, Royal Free Hospital, London, UK. Electronic address: a.salama@ucl.ac.uk.

Workman, Sarita. Department of Immunology, Royal Free London NHS Foundation Trust, London, UK. Electronic address: sarita.workman@nhs.net.

Wrench, David. Department of Haematology, Guy's and St Thomas' NHS Foundation Trust, London, UK. Electronic address: david.wrench@gstt.nhs.uk.

Karim, Mohammed Yousuf. Frimley Health NHS Foundation Trust, Portsmouth Rd, Frimley, UK. Electronic address: youshimmi@gmail.com.

Abstract

BACKGROUND: Consensus guidelines are not available for the use of immunoglobulin replacement therapy (IGRT) in patients developing iatrogenic secondary antibody deficiency following B-cell targeted therapy (BCTT) in autoimmune rheumatic disease.

OBJECTIVES: To evaluate the role of IGRT to manage hypogammaglobulinemia following BCTT in autoimmune rheumatic disease (AIRD).

METHODS: Using an agreed search string we performed a systematic literature search on Medline with Pubmed as vendor. We limited the search to English language papers with abstracts published over the last 10years. Abstracts were screened for original data regarding hypogammaglobulinemia following BCTT and the use of IGRT for hypogammaglobulinemia following BCTT. We also searched current recommendations from national/international organisations including British Society for Rheumatology, UK Department of Health, American College of Rheumatology, and American Academy of Asthma, Allergy and Immunology.

RESULTS: 222 abstracts were identified. Eight papers had original relevant data that met our search criteria. These studies were largely retrospective cohort studies with small patient numbers receiving IGRT. The literature highlights the induction of a sustained antibody

deficiency, risk factors for hypogammaglobulinemia after BCTT including low baseline serum IgG levels, how to monitor patients for the development of hypogammaglobulinemia and the limited evidence available on intervention thresholds for commencing IGRT.

CONCLUSION: The benefit of BCTT needs to be balanced against the risk of inducing a sustained secondary antibody deficiency. Consensus guidelines would be useful to enable appropriate assessment prior to and following BCTT in preventing and diagnosing hypogammaglobulinemia. Definitions for symptomatic hypogammaglobulinemia, intervention thresholds and treatment targets for IGRT, and its cost-effectiveness are required. Crown Copyright © 2019. Published by Elsevier B.V. All rights reserved.

Publication Type
Journal Article. Systematic Review.
Year of Publication
2019

<47>
Unique Identifier
30828027
Title
[Effectiveness of a Low-dose Corticosteroid in a Patient with Polymyalgia Rheumatica Associated with Nivolumab Treatment]. [Japanese]
Yakugaku Zasshi - \\\\\\\\\\\

Authors
Imai Y; Tanaka M; Fujii R; Uchitani K; Okazaki K.
Authors Full Name
Imai, Yusuke; Tanaka, Masayuki; Fujii, Ryohei; Uchitani, Kazuki; Okazaki, Kazuichi.
Institution
Imai, Yusuke. Department of Hospital Pharmacy, Kansai Medical University.
Tanaka, Masayuki. Department of Hospital Pharmacy, Kansai Medical University.
Fujii, Ryohei. Department of Hospital Pharmacy, Kansai Medical University.
Uchitani, Kazuki. Department of Hospital Pharmacy, Kansai Medical University.
Okazaki, Kazuichi. Department of Hospital Pharmacy, Kansai Medical University.

Abstract

Nivolumab, an anti-programmed cell death 1 antibody, has been approved for the treatment of unresectable advanced non-small-cell lung cancer (NSCLC). Although immune-related adverse events (irAEs) such as dermatologic, digestive, endocrine, hepatic, and pulmonary toxicities are known to occur upon administration of immune checkpoint inhibitors, case reports of polymyalgia rheumatica (PMR) associated with nivolumab use are rare. We report a case of an NSCLC patient who developed PMR during treatment with nivolumab and received corticosteroids. A 74-year-old man without a history of autoimmune diseases received nivolumab at a dosage of 3 mg/kg once every 2 weeks for the treatment of stage IIIB squamous cell carcinoma. After 12 cycles of nivolumab treatment, he developed grade 3 muscle pain and arthralgia, requiring hospitalization and discontinuation of nivolumab. A bone scintigraphy revealed no bone metastasis. Serological tests showed that although creatine phosphokinase did not increase, C-reactive protein and the erythrocyte sedimentation rate were both high. Tests for the rheumatoid factor, anti-cyclic citrullinated peptide antibody, and anti-nuclear antibody were negative. In addition to the serological findings, joint ultrasonography data and clinical symptoms were evaluated, leading to the diagnosis of PMR. Oral prednisolone 20 mg/d was started to treat the PMR without giant-cell arteritis. The patient's symptoms improved within 5 d of the initiation of treatment. Prednisolone was tapered to 5 mg/d without recurrence of PMR. Although grade 3 or 4 irAEs (except in type 1 diabetes) are generally treated with high-dose corticosteroids, grade 3 PMR associated with nivolumab use may be treatable with low-dose corticosteroids.

Publication Type

Case Reports. Journal Article.

Year of Publication

2019

<48>

Unique Identifier

30819683

Title

Immune checkpoint inhibitor-induced rheumatoid arthritis: insights into an increasingly common aetiology of polyarthritis.

Source

BMJ Case Reports. 12(2), 2019 Feb 27.

VI 1

Status

MEDLINE

Authors

Kwok TSH; Bell MJ.

Authors Full Name

Kwok, Timothy Shun Him; Bell, Mary Jane.

Institution

Kwok, Timothy Shun Him. Department of Medicine, University of Toronto, Toronto, Ontario, Canada.

Bell, Mary Jane. Sunnybrook Health Sciences Centre, Toronto, Ontario, Canada.

Bell, Mary Jane. Division of Rheumatology, Department of Medicine, University of Toronto, Toronto, Ontario, Canada.

Abstract

Nivolumab is an immune checkpoint inhibitor that is used in the treatment of a variety of cancers in the adjuvant or metastatic setting. Adverse effects include non-specific activation of T cells, leading to immune-related adverse events in downstream organs. We present a case of a 36-year-old man with unresectable oropharyngeal squamous cell carcinoma who developed nivolumab-induced rheumatoid arthritis. As immune checkpoint inhibitor use is becoming widespread in the medical oncology domain, the purpose of this case report is to increase awareness of an increasingly common cause of rheumatic disease and to alert clinicians to consider immunotherapy in their differential diagnosis of polyarthritis. This case also highlights the importance of working in an interdisciplinary manner to enhance cancer care for the patient as well as to increase awareness of the potential adverse effects of immunotherapy in patients with cancer. Copyright © BMJ Publishing Group Limited 2019. No commercial re-use. See rights and permissions. Published by BMJ.

Publication Type

Case Reports. Journal Article.

Year of Publication

2019

<49>

Unique Identifier

30776645

Title

Novel aspects of the activation of NADPH oxidase in neutrophils of rheumatic patients on biological therapy.

Source

International Immunopharmacology. 69:368-372, 2019 Apr.

VI 1

Status

MEDLINE

Authors

Jancinova V; Drabikova K; Killinger Z; Pazourekova S; Nosal R; Payer J.

Authors Full Name

Jancinova, Viera; Drabikova, Katarina; Killinger, Zdenko; Pazourekova, Silvia; Nosal, Radomir; Payer, Juraj.

Institution

Jancinova, Viera. Institute of Experimental Pharmacology and Toxicology, Centre of Experimental Medicine, Slovak Academy of Sciences, Dubravska cesta 9, 841 04 Bratislava, Slovak Republic. Electronic address: Viera.Jancinova@savba.sk.

Drabikova, Katarina. Institute of Experimental Pharmacology and Toxicology, Centre of Experimental Medicine, Slovak Academy of Sciences, Dubravska cesta 9, 841 04 Bratislava, Slovak Republic.

Killinger, Zdenko. 5th Department of Internal Medicine, Medical Faculty of Comenius University, University Hospital, Ruzinovska 6, 826 06 Bratislava, Slovak Republic.

Pazourekova, Silvia. Institute of Experimental Pharmacology and Toxicology, Centre of Experimental Medicine, Slovak Academy of Sciences, Dubravska cesta 9, 841 04 Bratislava, Slovak Republic.

Nosal, Radomir. Institute of Experimental Pharmacology and Toxicology, Centre of Experimental Medicine, Slovak Academy of Sciences, Dubravska cesta 9, 841 04 Bratislava, Slovak Republic.

Payer, Juraj. 5th Department of Internal Medicine, Medical Faculty of Comenius University, University Hospital, Ruzinovska 6, 826 06 Bratislava, Slovak Republic.

Abstract

The relationship between inflammation and formation of reactive oxygen species (ROS) is still not completely understood and excessive inflammatory reaction is attributed to increased yet also to reduced ROS formation. To compare ROS formation in severe and low inflammation, neutrophil oxidative burst was analyzed in rheumatic patients before and during therapy with TNFalpha- or interleukin-6 receptor-neutralizing antibodies. Intracellular and extracellular ROS

productions were evaluated on the basis of luminol- and isoluminol-enhanced chemiluminescence in isolated peripheral neutrophils. Disease activity score DAS28 and platelet to lymphocyte ratio were used as markers of arthritis activity and the intensity of systemic inflammation. Biological therapy effectively reduced the intensity of inflammation. Of the twenty-six patients studied eighteen achieved remission or low disease activity. Highly active arthritis persisted only in one patient, though prior to the therapy it was evident in all subjects tested. In patients receiving biological therapy, intracellular chemiluminescence was significantly higher than in patients before this therapy; ROS produced by neutrophils extracellularly were not affected. The increased ROS formation associated with reduced inflammation supports the need to revise the view of the role of ROS in inflammation - from toxic agents promoting inflammation towards a more complex view of ROS as regulators of immune pathways with inflammation-limiting capacity. From this perspective, the interference with neutrophil-derived oxidants may represent a new mechanism involved in the anti-inflammatory activity of biological therapy.

Copyright © 2019. Published by Elsevier B.V.

Publication Type

Journal Article.

Year of Publication

2019

<50>

Unique Identifier

30701346

Title

Hydroxychloroquine is a safe and effective steroid-sparing agent for immune checkpoint inhibitor-induced inflammatory arthritis. Clinical Rheumatology. 38(5):1513-1519, 2019 May.

Authors

Roberts J; Smylie M; Walker J; Basappa NS; Chu Q; Kolinsky M; Lyddell C; Ye C.

Author NameID

Roberts, Janet; ORCID: http://orcid.org/0000-0002-3787-8541

Authors Full Name

Roberts, Janet; Smylie, Michael; Walker, John; Basappa, Naveen S; Chu, Quincy; Kolinsky, Michael; Lyddell, Christopher; Ye, Carrie.

Institution

Roberts, Janet. Division of Rheumatology, Department of Medicine, Dalhousie University, Suite 245 Nova Scotia Rehabilitation Building, 1341 Summer Street, Halifax, NS, B3H 4K4, Canada. roberts5@ualberta.ca.
Smylie, Michael. Division of Medical Oncology, Department of Oncology, Cross Cancer Institute, University of Alberta, 11560 University Avenue, Edmonton, Alberta, T6G 1Z2, Canada.
Walker, John. Division of Medical Oncology, Department of Oncology, Cross Cancer Institute, University of Alberta, 11560 University Avenue, Edmonton, Alberta, T6G 1Z2, Canada.
Basappa, Naveen S. Division of Medical Oncology, Department of Oncology, Cross Cancer Institute, University of Alberta, 11560 University Avenue, Edmonton, Alberta, T6G 1Z2, Canada.
Chu, Quincy. Division of Medical Oncology, Department of Oncology, Cross Cancer Institute, University of Alberta, 11560 University Avenue, Edmonton, Alberta, T6G 1Z2, Canada.
Kolinsky, Michael. Division of Medical Oncology, Department of Oncology, Cross Cancer Institute, University of Alberta, 11560 University Avenue, Edmonton, Alberta, T6G 1Z2, Canada.
Lyddell, Christopher. Division of Rheumatology, Department of Medicine, University of Alberta, 8-120 Clinical Sciences Building, 8440 112 St NW, Edmonton, AB, T6G 2G3, Canada.
Ye, Carrie. Division of Rheumatology, Department of Medicine, University of Alberta, 8-120 Clinical Sciences Building, 8440 112 St NW, Edmonton, AB, T6G 2G3, Canada.
Abstract
Immunotherapy for cancer treatment continues to evolve, and immune checkpoints have proven successful therapeutic targets. With success has come the challenge of managing the commonly associated immune-related toxicities. Arthralgias and arthritis are a common immune-related adverse event (IrAE), well described in the literature (Pardoll Nat Rev Cancer 12:252-264, 2012; Diesendruck and Benhar Drug Resist Updat 30:39-47, 2017; Cappelli et al. Arthritis Care Res 69:1751-1763, 2017; Brahmer et al. J Clin Oncol 36:1714-1768, 2018; Smith and Bass (2017). The optimal management of immune checkpoint inhibitor (ICI)-induced arthritis remains unclear. We describe the first series using hydroxychloroquine as a first-line disease-modifying antirheumatic drug (DMARD) for patients without pre-existing autoimmune disease, who developed arthritis secondary to ICI's. This was a single-center retrospective observational study reporting all patients evaluated by rheumatologists affiliated with the University of Alberta, a large tertiary health care center in Northern Alberta, Canada, deemed to have inflammatory arthritis (IA) following ICIs. We identified 11 patients, without pre-existing autoimmune disease, who developed IA following ICIs. Most patients presented with a symmetrical polyarthritis with both large and small joint involvement. All patients were treated according to the outlined treatment protocol with hydroxychloroquine as a first-line steroid-sparing agent: either as monotherapy or in combination with tapering doses of systemic corticosteroids (3) or intra-articular steroid injections (6). One patient required the addition of methotrexate to control symptoms and none required biologic therapy. There were no reported adverse effects from hydroxychloroquine. Inflammatory

arthritis is an important complication of ICIs leading to significant impact on patient quality of life. In our experience, in patients without pre-existing autoimmune disease, hydroxychloroquine is an effective first-line therapy for IA secondary to ICI therapy.

Publication Type

Journal Article. Observational Study.

Year of Publication

2019

<51>

Unique Identifier

30659358

Title

[Infections]. [Review] [German]

Source

Zeitschrift fur Rheumatologie. 78(3):236-242, 2019 Apr.

VI 1

Status

MEDLINE

Authors

Lorenz HM; Kneitz C.

Authors Full Name

Lorenz, H-M; Kneitz, C.

Institution

Lorenz, H-M. Medizinische Universitatsklinik V: Hamatologie, Onkologie, Rheumatologie, Universitatsklinikum Heidelberg, Heidelberg, Deutschland.

Lorenz, H-M. ACURA Rheumazentrum Baden-Baden, Baden-Baden, Deutschland.

Kneitz, C. Rheumatologische Facharztpraxis Schwerin, Beethovenstr. 3, 19053, Schwerin, Deutschland. christian.kneitz@rheuma-schwerin.de.

Abstract

The individual risk assessment concerning infections in patients with rheumatic diseases is based on the detailed personalized documentation of relevant risk factors, such as the underlying disease itself, the intensity of immunosuppressive therapy and the severity of any comorbidities. From the perspective of infectiology, the history of repeated and severe infections as well as

previous illnesses, such as (latent) tuberculosis and chronic hepatitis B or C need to be considered. In some instances prophylactic antibiotic therapy might be required, which should otherwise be avoided in order to prevent selection of resistant pathogens. Furthermore, vaccinations are particularly suitable to specifically minimize the risk for frequent infectious diseases.

Publication Type

Journal Article. Review.

Year of Publication

2019

<52>

Unique Identifier

30642768

Title

Effectiveness and safety of lower dose sulfamethoxazole/trimethoprim therapy for Pneumocystis jirovecii pneumonia in patients with systemic rheumatic diseases: A retrospective multicenter study.

Source

Journal of Infection & Chemotherapy. 25(4):253-261, 2019 Apr.

VI 1

Status

MEDLINE

Authors

Ohmura SI; Naniwa T; Tamechika SY; Miyamoto T; Shichi D; Kazawa N; Iwagaitsu S; Maeda S; Wada JI; Niimi A.

Authors Full Name

Ohmura, Shin-Ichiro; Naniwa, Taio; Tamechika, Shin-Ya; Miyamoto, Toshiaki; Shichi, Daisuke; Kazawa, Nobukata; Iwagaitsu, Shiho; Maeda, Shinji; Wada, Jun-Ichi; Niimi, Akio.

Institution

Ohmura, Shin-Ichiro. Division of Rheumatology, Department of Internal Medicine, Nagoya City University Hospital, Nagoya, Aichi, Japan; Department of Respiratory Medicine, Allergy and Clinical Immunology, Nagoya City University Graduate School of Medical Sciences, Nagoya, Aichi, Japan.

Naniwa, Taio. Division of Rheumatology, Department of Internal Medicine, Nagoya City University Hospital, Nagoya, Aichi, Japan; Department of Respiratory Medicine, Allergy and Clinical Immunology, Nagoya City University Graduate School of Medical Sciences, Nagoya, Aichi, Japan. Electronic address: tnaniwa@med.nagoya-cu.ac.jp.

Tamechika, Shin-Ya. Division of Rheumatology, Department of Internal Medicine, Nagoya City University Hospital, Nagoya, Aichi, Japan; Department of Respiratory Medicine, Allergy and Clinical Immunology, Nagoya City University Graduate School of Medical Sciences, Nagoya, Aichi, Japan.

Miyamoto, Toshiaki. Department of Rheumatology, Seirei Hamamatsu General Hospital, Hamamatsu, Shizuoka, Japan.

Shichi, Daisuke. Department of Infectious Diseases and Rheumatology, Seirei Mikatahara General Hospital, Hamamatsu, Shizuoka, Japan.

Kazawa, Nobukata. Department of Radiology, Kansai Medical University Medical Center, Moriguchi, Osaka, Japan.

Iwagaitsu, Shiho. Department of Internal Medicine, Division of Rheumatology and Nephrology, Aichi Medical University School of Medicine, Nagakute, Aichi, Japan.

Maeda, Shinji. Division of Rheumatology, Department of Internal Medicine, Nagoya City University Hospital, Nagoya, Aichi, Japan; Department of Respiratory Medicine, Allergy and Clinical Immunology, Nagoya City University Graduate School of Medical Sciences, Nagoya, Aichi, Japan.

Wada, Jun-Ichi. Division of Rheumatology, Department of Internal Medicine, Nagoya City University Hospital, Nagoya, Aichi, Japan; Department of Internal Medicine, Toyokawa City Hospital, Toyokawa, Aichi, Japan.

Niimi, Akio. Department of Respiratory Medicine, Allergy and Clinical Immunology, Nagoya City University Graduate School of Medical Sciences, Nagoya, Aichi, Japan.

Abstract

OBJECTIVES: To evaluate the effectiveness and safety of lower-dose sulfamethoxazole/trimethoprim therapy (SMX/TMP) for Pneumocystis jirovecii pneumonia (PCP) in patients with systemic rheumatic diseases.

METHODS: In this multicenter retrospective study, we compared effectiveness and safety of SMX/TMP for the treatment of PCP among patients divided into three groups according to the initial dosage of SMX/TMP: the low, <=10 mg/kg/day; the intermediate, 10-15 mg/kg/day; and the high and conventional, 15-20 mg/kg/day for TMP dose.

RESULTS: Eighty-one patients, including 22, 30, and 29 patients in the low-, the intermediate- and the high-dose group could be analyzed and the 30-day survival rate were 100%, 93.3%, and

96.7%, respectively (P = 0.28). There were significant dose-dependent increasing trends of severe adverse drug reactions (ADRs) for SMX/TMP that were graded as >=3 according to the Common Terminology Criteria for Adverse Events. When stratified by presence of severe hypoxemia defined by alveolar-arterial O2 gradient >=45 mmHg, the 30-day survival and treatment modification rate were similar among the three groups, but frequency of severe ADRs were significantly decreased in the low-dose group. The low-dose group was independently and negatively associated with treatment modification within 14 days and severe ADRs.

CONCLUSIONS: Lower dose SMX/TMP therapy with <=10 mg/kg/day for TMP was as effective as higher dose therapy for the treatment of PCP and associated with lower rates of treatment modification and severe ADRs in patients with systemic rheumatic diseases. Copyright © 2018 Japanese Society of Chemotherapy and The Japanese Association for Infectious Diseases. Published by Elsevier Ltd. All rights reserved.

Publication Type
Journal Article. Multicenter Study.
Year of Publication
2019

<53>
Unique Identifier
30593416
Title
Pleiotropic functions of plasmacytoid dendritic cells in the pathogenesis of the rheumatoid arthritis.
Source
Medical Hypotheses. 122:26-30, 2019 Jan.
VI 1
Status
MEDLINE
Authors
Kupriyanov SV; Sinitskii AI.
Authors Full Name
Kupriyanov, Semyon V; Sinitskii, Anton I.

Institution
Kupriyanov, Semyon V. Department of Biochemistry, South-Ural State Medical University, Vorovskogo St., 64, Chelyabinsk 454092, Russia. Electronic address: pfft@mail.ru.
Sinitskii, Anton I. Department of Biochemistry, South-Ural State Medical University, Vorovskogo St., 64, Chelyabinsk 454092, Russia.
Abstract
Rheumatoid arthritis is a chronic autoimmune inflammatory disease with an unclear etiology. The disease is characterized by infiltration of synovial tissue with immune cells, among which there are dendritic cells that play multifaceted roles in the pathogenesis of the disease. Here we shall assume that plasmacytoid dendritic cells are able to change their phenotype under the influence of various stimuli, thereby modulating the course of the disease, contributing to both the development of exacerbations and the induction of remissions depending on the phenotype they have acquired. This property can be used to develop new methods of immunotherapy. Copyright © 2018 Elsevier Ltd. All rights reserved.
Publication Type
Journal Article.
Year of Publication
2019

<54>
Unique Identifier
30586666
Title
Study of the adoptive immunotherapy on rheumatoid arthritis with Thymus-derived invariant natural killer T cells.
Source
International Immunopharmacology. 67:427-440, 2019 Feb.
VI 1
Status
MEDLINE
Authors
Chen D; Liu H; Wang Y; Chen S; Liu J; Li W; Dou H; Hou W; Meng M.
Authors Full Name

Chen, Dongzhi; Liu, Huifang; Wang, Yuanyuan; Chen, Shengde; Liu, Jialin; Li, Wenjuan; Dou, Haiyang; Hou, Wenguang; Meng, Ming.
Institution
Chen, Dongzhi. Medical School of Hebei University, Baoding, China.
Liu, Huifang. Medical School of Hebei University, Baoding, China.
Wang, Yuanyuan. Medical School of Hebei University, Baoding, China.
Chen, Shengde. Medical School of Hebei University, Baoding, China.
Liu, Jialin. Medical School of Hebei University, Baoding, China.
Li, Wenjuan. Medical School of Hebei University, Baoding, China.
Dou, Haiyang. Medical School of Hebei University, Baoding, China.
Hou, Wenguang. People's Hospital of Nankai University, Tianjin, China. Electronic address: 940373961@qq.com.
Meng, Ming. Medical School of Hebei University, Baoding, China. Electronic address: mengming127@163.com.
Abstract
BACKGROUND: The therapeutic effect of adoptive infusion of specific thymus-derived invariant natural killer T (iNKT) cells in a mouse model of rheumatoid arthritis (RA) was observed, and the mechanism of cellular immunotherapy was preliminarily explored.

METHODS: Thymus-derived iNKT cells were infused to RA model mice, with alpha-GalCer as a positive control. Then, ankle swelling was examined, as well as inflammatory cell infiltration to the joint tissue (hematoxylin-eosin [H&E] staining). Flow cytometry (FCM) was used to assess iNKT cell and helper T lymphocyte (Th) subsets. Serum cytokine levels were determined with cytometric bead array (CBA), with protein expression levels of related transcription factors assessed by Western blot.

RESULTS: The joint swelling in RA model animals were significantly improved in the cell therapy and alpha-GalCer positive control groups ($P<0.05$). In addition, iNKT frequencies in peripheral blood, the thymus and spleen were increased significantly ($P<0.05$). Meanwhile, iNKT1 subset frequencies in the thymus and spleen were decreased, as well as splenic Th1 and Th17 cell subset rates, and serum TNF-alpha, IFN-gamma and IL-6 levels. The rates of iNKT2 and Th2 subsets as well as IL-4 and IL-10 levels were increased ($P<0.05$). Thymus GATA-3 and splenic PLZF protein levels were increased ($P<0.05$).

CONCLUSIONS: Adoptive infusion of thymus-derived iNKT cells exerts therapeutic effects in RA mice by increasing iNKT frequency, altering the proportions of iNKT cell subsets, correcting

Th cell subset imbalance and reducing the amounts of inflammatory cytokines. Copyright © 2018. Published by Elsevier B.V.
Publication Type
Journal Article.
Year of Publication
2019

<55>
Unique Identifier
30552174
Title
Targeting early changes in the synovial microenvironment: a new class of immunomodulatory therapy?.
Source
Annals of the Rheumatic Diseases. 78(2):186-191, 2019 02.
VI 1
Status
MEDLINE
Authors
Aungier SR; Cartwright AJ; Schwenzer A; Marshall JL; Dyson MR; Slavny P; Parthiban K; Karatt-Vellatt A; Sahbudin I; Culbert E; Hextall P; Clanchy FI; Williams R; Marsden BD; Raza K; Filer A; Buckley CD; McCafferty J; Midwood KS.
Authors Full Name
Aungier, Susan R; Cartwright, Alison J; Schwenzer, Anja; Marshall, Jennifer L; Dyson, Michael R; Slavny, Peter; Parthiban, Kothai; Karatt-Vellatt, Aneesh; Sahbudin, Ilfita; Culbert, Eric; Hextall, Patrick; Clanchy, Felix Il; Williams, Richard; Marsden, Brian D; Raza, Karim; Filer, Andrew; Buckley, Christopher Dominic; McCafferty, John; Midwood, Kim S.
Institution
Aungier, Susan R. Kennedy Institute of Rheumatology, Nuffield Department of Orthopaedics, Rheumatology and Musculoskeletal Sciences, University of Oxford, Oxford, UK.
Cartwright, Alison J. Kennedy Institute of Rheumatology, Nuffield Department of Orthopaedics, Rheumatology and Musculoskeletal Sciences, University of Oxford, Oxford, UK.

Schwenzer, Anja. Kennedy Institute of Rheumatology, Nuffield Department of Orthopaedics, Rheumatology and Musculoskeletal Sciences, University of Oxford, Oxford, UK.

Marshall, Jennifer L. Institute of Inflammation and Ageing, University of Birmingham, Queen Elizabeth Hospital, Birmingham, UK.

Dyson, Michael R. IONTAS Ltd, Cambridge, UK.

Slavny, Peter. IONTAS Ltd, Cambridge, UK.

Parthiban, Kothai. IONTAS Ltd, Cambridge, UK.

Karatt-Vellatt, Aneesh. IONTAS Ltd, Cambridge, UK.

Sahbudin, Ilfita. Institute of Inflammation and Ageing, University of Birmingham, Queen Elizabeth Hospital, Birmingham, UK.

Culbert, Eric. Nascient Ltd, Cambridge, UK.

Hextall, Patrick. Nascient Ltd, Cambridge, UK.

Clanchy, Felix II. Kennedy Institute of Rheumatology, Nuffield Department of Orthopaedics, Rheumatology and Musculoskeletal Sciences, University of Oxford, Oxford, UK.

Williams, Richard. Kennedy Institute of Rheumatology, Nuffield Department of Orthopaedics, Rheumatology and Musculoskeletal Sciences, University of Oxford, Oxford, UK.

Marsden, Brian D. Kennedy Institute of Rheumatology, Nuffield Department of Orthopaedics, Rheumatology and Musculoskeletal Sciences, University of Oxford, Oxford, UK.

Marsden, Brian D. Structural Genomics Consortium, Nuffield Department of Clinical Medicine, University of Oxford, Oxford, UK.

Raza, Karim. Institute of Inflammation and Ageing, University of Birmingham, Queen Elizabeth Hospital, Birmingham, UK.

Raza, Karim. Department of Rheumatology, Sandwell and West Birmingham Hospitals NHS Trust, Birmingham, UK.

Filer, Andrew. Institute of Inflammation and Ageing, University of Birmingham, Queen Elizabeth Hospital, Birmingham, UK.

Buckley, Christopher Dominic. Kennedy Institute of Rheumatology, Nuffield Department of Orthopaedics, Rheumatology and Musculoskeletal Sciences, University of Oxford, Oxford, UK.

Buckley, Christopher Dominic. Institute of Inflammation and Ageing, University of Birmingham, Queen Elizabeth Hospital, Birmingham, UK.

McCafferty, John. Nascient Ltd, Cambridge, UK.

Midwood, Kim S. Kennedy Institute of Rheumatology, Nuffield Department of Orthopaedics, Rheumatology and Musculoskeletal Sciences, University of Oxford, Oxford, UK kim.midwood@kennedy.ox.ac.uk.

Abstract

OBJECTIVES: Controlled immune responses rely on integrated crosstalk between cells and their microenvironment. We investigated whether targeting proinflammatory signals from the

extracellular matrix that persist during pathological inflammation provides a viable strategy to treat rheumatoid arthritis (RA).

METHODS: Monoclonal antibodies recognising the fibrinogen-like globe (FBG) of tenascin-C were generated by phage display. Clones that neutralised FBG activation of toll-like receptor 4 (TLR4), without impacting pathogenic TLR4 activation, were epitope mapped by crystallography. Antibodies stained synovial biopsies of patients at different stages of RA development. Antibody efficacy in preventing RA synovial cell cytokine release, and in modulating collagen-induced arthritis in rats, was assessed.

RESULTS: Tenascin-C is expressed early in the development of RA, even before disease diagnosis, with higher levels in the joints of people with synovitis who eventually developed RA than in people whose synovitis spontaneously resolved. Anti-FBG antibodies inhibited cytokine release by RA synovial cells and prevented disease progression and tissue destruction during collagen-induced arthritis.

CONCLUSIONS: Early changes in the synovial microenvironment contribute to RA progression; blocking proinflammatory signals from the matrix can ameliorate experimental arthritis. These data highlight a new drug class that could offer early, disease-specific immune modulation in RA, without engendering global immune suppression. Copyright © Author(s) (or their employer(s)) 2018. Re-use permitted under CC BY. Published by BMJ.

Publication Type

Journal Article. Research Support, Non-U.S. Gov't.

Year of Publication

2019

<56>

Unique Identifier

30549256

Title

Association of good oncological response to therapy with the development of rheumatic immune-related adverse events following PD-1 inhibitor therapy. International Journal of Rheumatic Diseases. 22(2):297-302, 2019 Feb.

Authors
Liew DFL; Leung JLY; Liu B; Cebon J; Frauman AG; Buchanan RRC.
Author NameID
Liew, David F L; ORCID: http://orcid.org/0000-0001-8451-8883
Authors Full Name
Liew, David F L; Leung, Jessica L Y; Liu, Bonnia; Cebon, Jonathan; Frauman, Albert G; Buchanan, Russell R C.
Institution
Liew, David F L. Department of Rheumatology, Austin Health, Melbourne, VIC, Australia.
Liew, David F L. Department of Clinical Pharmacology and Therapeutics, Austin Health, Melbourne, VIC, Australia.
Liew, David F L. Department of Medicine, University of Melbourne, Melbourne, VIC, Australia.
Leung, Jessica L Y. Department of Rheumatology, Austin Health, Melbourne, VIC, Australia.
Liu, Bonnia. Department of Rheumatology, Austin Health, Melbourne, VIC, Australia.
Cebon, Jonathan. Olivia Newton-John Cancer Wellness & Research Centre, Melbourne, VIC, Australia.
Cebon, Jonathan. School of Cancer Medicine, La Trobe University, Melbourne, VIC, Australia.
Frauman, Albert G. Department of Clinical Pharmacology and Therapeutics, Austin Health, Melbourne, VIC, Australia.
Frauman, Albert G. Department of Medicine, University of Melbourne, Melbourne, VIC, Australia.
Buchanan, Russell R C. Department of Rheumatology, Austin Health, Melbourne, VIC, Australia.
Buchanan, Russell R C. Department of Medicine, University of Melbourne, Melbourne, VIC, Australia.
Abstract
AIM: To investigate whether any patient or treatment characteristics are associated with the development of rheumatic immune-related adverse events (irAEs) following programmed cell death protein 1 (PD-1) inhibitor therapy for cancer.

METHOD: This was a retrospective chart review of all patients who were dispensed nivolumab or pembrolizumab at a single center before 1 January, 2017, with follow-up until 1 July, 2017. Patients with any diagnosis of a non-cutaneous irAE were identified, regardless of severity, and rheumatic irAEs were characterized.

RESULTS: Of 244 episodes of therapy, a non-cutaneous irAE occurred in 72 (29.5%). Rheumatic irAEs were diagnosed in 19 episodes of therapy (7.8%), with 12 de novo diagnoses (5.1% of episodes without a pre-existing autoimmune rheumatic disease) and 7 exacerbations of existing disease. Review by a rheumatologist occurred in only 11 of these. Rheumatic irAEs were more common in patients with a good oncological response to therapy (relative risk [RR] 11.16), those being treated for melanoma (RR 2.94) and those who developed another non-cutaneous irAE (RR 2.64).

CONCLUSION: Rheumatic irAEs are relatively common with PD-1 inhibitor therapy, and appear to be associated with a good oncological response to therapy. Many rheumatic irAEs were not referred to rheumatological services. Prospective systematic investigation would be of benefit to explore these characteristics. Copyright © 2018 Asia Pacific League of Associations for Rheumatology and John Wiley & Sons Australia, Ltd.

Publication Type
Journal Article.
Year of Publication
2019

<57>
Unique Identifier
30418127
Title
Reply to: Vaccination for hepatitis B virus in an Australian pre-biologic population with rheumatoid arthritis.
Source
Clinical & Experimental Rheumatology. 37(1):164, 2019 Jan-Feb.
VI 1
Status
MEDLINE
Authors
Meroni PL; Zavaglia D; Girmenia C.
Authors Full Name
Meroni, Pier Luigi; Zavaglia, Daniela; Girmenia, Corrado.

Institution

Meroni, Pier Luigi. Immunorheumatology Research Laboratory, IRCCS Istituto Auxologico Italiano, Milan, Italy. pierluigi.meroni@unimi.it.

Zavaglia, Daniela. Medical Department, Pfizer Italia, Rome, Italy.

Girmenia, Corrado. Department of Haematology, Oncology, and Dermatology, Azienda Policlinico Umberto I, Sapienza University, Rome, Italy.

Publication Type

Letter.

Year of Publication

2019

<58>

Unique Identifier

30418119

Title

Vaccination for hepatitis B virus in an Australian pre-biologic population with rheumatoid arthritis.

Source

Clinical & Experimental Rheumatology. 37(1):164, 2019 Jan-Feb.

VI 1

Status

MEDLINE

Authors

Ihdayhid D; Edelman J; Keen HI.

Authors Full Name

Ihdayhid, Dana; Edelman, Jack; Keen, Helen I.

Institution

Ihdayhid, Dana. Fiona Stanley Hospital, University of Western Australia, Perth, Western Australia.

Edelman, Jack. Sir Charles Gardiner Hospital, University of Western Australia, Perth, Western Australia.

Keen, Helen I. Royal Perth Hospital Unit, University of Western Australia, Perth, Western Australia. helen.keen@uwa.edu.au.

Publication Type

Letter.
Year of Publication
2019

<59>
Unique Identifier
30307604
Title
IL4-10 fusion protein: a novel immunoregulatory drug combining activities of interleukin 4 and interleukin 10.
Source
Clinical & Experimental Immunology. 195(1):1-9, 2019 01.
VI 1
Status
MEDLINE
Authors
Steen-Louws C; Hartgring SAY; Popov-Celeketic J; Lopes AP; de Smet MBM; Eijkelkamp N; Lafeber FPJG; Hack CE; van Roon JAG.
Author NameID
Steen-Louws, C; ORCID: https://orcid.org/0000-0001-5775-3917
Eijkelkamp, N; ORCID: https://orcid.org/0000-0003-0039-7063
Authors Full Name
Steen-Louws, C; Hartgring, S A Y; Popov-Celeketic, J; Lopes, A P; de Smet, M B M; Eijkelkamp, N; Lafeber, F P J G; Hack, C E; van Roon, J A G.
Institution
Steen-Louws, C. Laboratory of Translational Immunology, University Medical Center Utrecht, the Netherlands.
Steen-Louws, C. Department of Rheumatology and Clinical Immunology, Utrecht, the Netherlands.
Hartgring, S A Y. Laboratory of Translational Immunology, University Medical Center Utrecht, the Netherlands.
Hartgring, S A Y. Department of Rheumatology and Clinical Immunology, Utrecht, the Netherlands.

Popov-Celeketic, J. Department of Rheumatology and Clinical Immunology, Utrecht, the Netherlands.
Lopes, A P. Laboratory of Translational Immunology, University Medical Center Utrecht, the Netherlands.
Lopes, A P. Department of Rheumatology and Clinical Immunology, Utrecht, the Netherlands.
de Smet, M B M. Laboratory of Translational Immunology, University Medical Center Utrecht, the Netherlands.
Eijkelkamp, N. Laboratory of Translational Immunology, University Medical Center Utrecht, the Netherlands.
Eijkelkamp, N. Laboratory of Neuroimmunology and Developmental Origins of Disease, Utrecht, the Netherlands.
Lafeber, F P J G. Department of Rheumatology and Clinical Immunology, Utrecht, the Netherlands.
Hack, C E. Laboratory of Translational Immunology, University Medical Center Utrecht, the Netherlands.
Hack, C E. Department of Rheumatology and Clinical Immunology, Utrecht, the Netherlands.
van Roon, J A G. Laboratory of Translational Immunology, University Medical Center Utrecht, the Netherlands.
van Roon, J A G. Department of Rheumatology and Clinical Immunology, Utrecht, the Netherlands.

Abstract

The objective of this study was to test the capacity of a newly developed fusion protein of interleukin 4 (IL-4) and IL-10 [IL4-10 fusion protein (FP)] to shift multiple pro-inflammatory pathways towards immune regulation, and to inhibit pro-inflammatory activity in arthritis models. The effects of IL4-10 FP in comparison with IL-4, IL-10 and IL-4 plus IL-10 on pro- and anti-inflammatory mediators, T cells and immunoglobulin (Ig) receptors in favour of immunoregulatory activity were studied. In addition, the capacity of IL4-10 FP to inhibit pro-inflammatory activity in ex-vivo and in-vivo arthritis models was investigated. IL4-10 FP robustly inhibited pro-inflammatory cytokine [IL-1beta, tumour necrosis factor (TNF)-alpha, IL-6 and IL-8] production in whole blood cultures, mediated by both the IL-10 and the IL-4 moiety. IL4-10 fusion protein induced IL-1 receptor antagonist (IL-1RA) production and preserved soluble TNF receptor (sTNFR) levels, strongly increasing IL-1RA/IL-1beta and sTNFR/TNF-alpha ratios. In addition, IL4-10 FP strongly inhibited T helper (Th) type 1 and 17 cytokine secretion, while maintaining FoxP3 expression and up-regulating Th2 activity. In addition, while largely leaving expression of activating Fc gamma receptor (FcgammaR)I, III and Fc epsilon receptor (FcepsilonR) unaffected, it significantly shifted the FcgammaRIIa/FcgammaRIIb ratio in favour of the inhibitory FcgammaRIIb. Moreover, IL4-10 FP robustly inhibited secretion of pro-inflammatory cytokines by

rheumatoid arthritis synovial tissue and suppressed experimental arthritis in mice, without inducing B cell hyperactivity. IL4-10 fusion protein is a novel drug, signalling cells to induce immunoregulatory activity that overcomes limitations of IL-4 and IL-10 stand-alone therapy, and therefore has therapeutic potential for inflammatory diseases such as rheumatoid arthritis.
Copyright © 2018 The Authors. Clinical & Experimental Immunology published by John Wiley & Sons Ltd on behalf of British Society for Immunology.
Publication Type
 Journal Article. Research Support, Non-U.S. Gov't.
Year of Publication
 2019

<60>
Unique Identifier
 30281202
Title
 Rheumatic Syndromes Associated With Immune Checkpoint Inhibitors: A Single-Center Cohort of Sixty-One Patients. Arthritis & Rheumatology. 71(3):468-475, 2019 03.

Authors
 Richter MD; Crowson C; Kottschade LA; Finnes HD; Markovic SN; Thanarajasingam U.
Authors Full Name
 Richter, Michael D; Crowson, Cynthia; Kottschade, Lisa A; Finnes, Heidi D; Markovic, Svetomir N; Thanarajasingam, Uma.
Institution
 Richter, Michael D. Mayo Clinic, Rochester, Minnesota.
 Crowson, Cynthia. Mayo Clinic, Rochester, Minnesota.
 Kottschade, Lisa A. Mayo Clinic, Rochester, Minnesota.
 Finnes, Heidi D. Mayo Clinic, Rochester, Minnesota.
 Markovic, Svetomir N. Mayo Clinic, Rochester, Minnesota.
 Thanarajasingam, Uma. Mayo Clinic, Rochester, Minnesota.
Abstract
 OBJECTIVE: To describe the prevalence, clinical presentation, and management of rheumatic immune-related adverse effects (Rh-irAEs) from immune checkpoint inhibitor (ICI) therapy.

METHODS: From a database of all patients who received any ICI at the Mayo Clinic Rochester, Minnesota campus between January 1, 2011 and March 1, 2018, we retrospectively identified those with Rh-irAEs, using diagnostic codes, search terms, and manual chart review.

RESULTS: Of the 1,293 patients who received any ICI, Rh-irAEs were clinically diagnosed in 43. Eighteen patients with Rh-irAEs who received ICI therapy elsewhere were also analyzed. Clinical syndromes included inflammatory arthritis (n = 34 [prevalence 2%]), myopathy (n = 10), and other rheumatic syndromes (n = 17). Inflammatory arthritis was most commonly polyarticular, and glucocorticoid treatment was required in 26 patients (76%). The mean +/- SD duration of treatment was 18 +/- 18 weeks. Five patients (15%) also received disease-modifying antirheumatic drugs, and ICI therapy had to be discontinued in 3 patients (9%). Myopathy was treated with glucocorticoids in all cases (mean +/- SD treatment duration 15 +/- 17 weeks) and led to 2 deaths and permanent ICI discontinuation in 9 patients (90%). Other syndromes included connective tissue diseases, vasculitis, polymyalgia rheumatica-like syndrome, and flare of preexisting rheumatic disease. Most (71%) were treated with immunosuppression, with 12% requiring ICI discontinuation.

CONCLUSION: This study represents the largest cohort of patients with Rh-irAEs reported to date. Most patients received long courses of immunosuppressive treatment, although discontinuation of ICI therapy was required in only a minority. Copyright © 2019, American College of Rheumatology.

Publication Type

Evaluation Study. Journal Article.

Year of Publication

2019

<61>

Unique Identifier

30249155

Title

Polymyalgia rheumatica due to pembrolizumab therapy. Journal of Oncology Pharmacy Practice. 25(5):1282-1284, 2019 Jul.

Authors

Iskandar A; Hwang A; Dasanu CA.

Authors Full Name

Iskandar, Andrew; Hwang, Andrew; Dasanu, Constantin A.

Institution

Iskandar, Andrew. 1 Department of Medicine, Eisenhower Medical Center, Rancho Mirage, CA, USA.

Hwang, Andrew. 1 Department of Medicine, Eisenhower Medical Center, Rancho Mirage, CA, USA.

Dasanu, Constantin A. 2 Lucy Curci Cancer Center, Eisenhower Medical Center, Rancho Mirage, CA, USA.

Dasanu, Constantin A. 3 UC San Diego Health: Department of Oncology.

Abstract

Pembrolizumab is a humanized anti-programmed cell death 1 antibody used for the therapy of several malignancies. While autoimmune adverse events are not uncommon with this agent, they are typically mild and self-limiting. Severe autoimmunity is rare but can be life-threatening. Herein, we describe a unique case of severe proximal muscle weakness and joint pain shortly after beginning therapy with pembrolizumab. Work-up revealed elevated pro-inflammatory markers leading to the diagnosis of polymyalgia rheumatica. Steroids allowed for resolution of the joint pain. We call for awareness of this rare autoimmune toxicity with pembrolizumab.

Publication Type

Case Reports. Journal Article.

Year of Publication

2019

<62>

Unique Identifier

30137623

Title

The British Society for Rheumatology biologic DMARD safety guidelines in inflammatory arthritis-Executive summary.

Source

Rheumatology. 58(2):220-226, 2019 02 01.
VI 1
Status
MEDLINE
Authors
Holroyd CR; Seth R; Bukhari M; Malaviya A; Holmes C; Curtis E; Chan C; Yusuf MA; Litwic A; Smolen S; Topliffe J; Bennett S; Humphreys J; Green M; Ledingham J.
Authors Full Name
Holroyd, Christopher R; Seth, Rakhi; Bukhari, Marwan; Malaviya, Anshuman; Holmes, Claire; Curtis, Elizabeth; Chan, Christopher; Yusuf, Mohammed A; Litwic, Anna; Smolen, Susan; Topliffe, Joanne; Bennett, Sarah; Humphreys, Jennifer; Green, Muriel; Ledingham, Jo.
Institution
Holroyd, Christopher R. Rheumatology Department, University Hospital Southampton NHS Foundation Trust, Southampton, UK.
Seth, Rakhi. Rheumatology Department, University Hospital Southampton NHS Foundation Trust, Southampton, UK.
Bukhari, Marwan. Rheumatology Department, University Hospitals of Morecombe Bay NHS Foundation Trust, Lancaster, UK.
Malaviya, Anshuman. Rheumatology Department, Mid Essex Hospital NHS Trust, Chelmsford, UK.
Holmes, Claire. Rheumatology Department, University Hospital Southampton NHS Foundation Trust, Southampton, UK.
Curtis, Elizabeth. MRC Lifecourse Epidemiology Unit, University of Southampton, Southampton, UK.
Chan, Christopher. Rheumatology Department, University Hospital Southampton NHS Foundation Trust, Southampton, UK.
Yusuf, Mohammed A. Rheumatology Department, Mid Essex Hospital NHS Trust, Chelmsford, UK.
Litwic, Anna. MRC Lifecourse Epidemiology Unit, University of Southampton, Southampton, UK.
Litwic, Anna. Rheumatology Department, Salisbury District Hospital, Salisbury, UK.
Smolen, Susan. Rheumatology Department, Mid Essex Hospital NHS Trust, Chelmsford, UK.
Topliffe, Joanne. Rheumatology Department, Mid Essex Hospital NHS Trust, Chelmsford, UK.
Bennett, Sarah. Rheumatology Department, University Hospital Southampton NHS Foundation Trust, Southampton, UK.
Humphreys, Jennifer. Arthritis Research UK Centre for Epidemiology, University of Manchester, Manchester, UK.

Green, Muriel. National Rheumatoid Arthritis Society, Queen Alexandra Hospital, Portsmouth, UK.
Ledingham, Jo. Rheumatology Department, Queen Alexandra Hospital, Portsmouth, UK.
Comments
Erratum in (EIN)
Publication Type
Journal Article. Practice Guideline.
Year of Publication
2019

<63>
Unique Identifier
30137552
Title
The British Society for Rheumatology biologic DMARD safety guidelines in inflammatory arthritis.
Source
Rheumatology. 58(2):e3-e42, 2019 02 01.
VI 1
Status
MEDLINE
Authors
Holroyd CR; Seth R; Bukhari M; Malaviya A; Holmes C; Curtis E; Chan C; Yusuf MA; Litwic A; Smolen S; Topliffe J; Bennett S; Humphreys J; Green M; Ledingham J.
Authors Full Name
Holroyd, Christopher R; Seth, Rakhi; Bukhari, Marwan; Malaviya, Anshuman; Holmes, Claire; Curtis, Elizabeth; Chan, Christopher; Yusuf, Mohammed A; Litwic, Anna; Smolen, Susan; Topliffe, Joanne; Bennett, Sarah; Humphreys, Jennifer; Green, Muriel; Ledingham, Jo.
Institution
Holroyd, Christopher R. Rheumatology Department, University Hospital Southampton NHS Foundation Trust, Southampton, UK.
Seth, Rakhi. Rheumatology Department, University Hospital Southampton NHS Foundation Trust, Southampton, UK.

Bukhari, Marwan. Rheumatology Department, University Hospitals of Morecombe Bay NHS Foundation Trust, Lancaster, UK.
Malaviya, Anshuman. Rheumatology Department, Mid Essex hospitals NHS Trust, Chelmsford, UK.
Holmes, Claire. Rheumatology Department, University Hospital Southampton NHS Foundation Trust, Southampton, UK.
Curtis, Elizabeth. MRC Lifecourse Epidemiology Unit, University of Southampton, Southampton, UK.
Chan, Christopher. Rheumatology Department, University Hospital Southampton NHS Foundation Trust, Southampton, UK.
Yusuf, Mohammed A. Rheumatology Department, Mid Essex hospitals NHS Trust, Chelmsford, UK.
Litwic, Anna. MRC Lifecourse Epidemiology Unit, University of Southampton, Southampton, UK.
Litwic, Anna. Rheumatology Department, Salisbury District Hospital, Salisbury, UK.
Smolen, Susan. Rheumatology Department, Mid Essex hospitals NHS Trust, Chelmsford, UK.
Topliffe, Joanne. Rheumatology Department, Mid Essex hospitals NHS Trust, Chelmsford, UK.
Bennett, Sarah. Rheumatology Department, University Hospital Southampton NHS Foundation Trust, Southampton, UK.
Humphreys, Jennifer. Arthritis Research UK Centre for Epidemiology, University of Manchester, Manchester, UK.
Green, Muriel. National Rheumatoid Arthritis Society, Queen Alexandra Hospital, Portsmouth, UK.
Ledingham, Jo. Rheumatology Department, Queen Alexandra Hospital, Portsmouth, UK.
Comments
Erratum in (EIN)
Publication Type
Journal Article. Practice Guideline.
Year of Publication
2019

<64>
Unique Identifier

30053610

Title

Reply to the comment of Fayet et al. Obstacles and motivations to influenza and pneumococcal vaccination in patients with rheumatoid arthritis. A qualitative study.

Source

Joint, Bone, Spine: Revue du Rhumatisme. 86(1):119, 2019 01.

VI 1

Status

MEDLINE

Authors

Assala M; Groh M; Blanche P; Vinter C; Cohen P; Le Guern V; Puechal X; Mouthon L; Le Jeunne C; Launay O; Kerneis S.

Authors Full Name

Assala, Manal; Groh, Matthieu; Blanche, Philippe; Vinter, Christine; Cohen, Patrice; Le Guern, Veronique; Puechal, Xavier; Mouthon, Luc; Le Jeunne, Claire; Launay, Odile; Kerneis, Solen.

Institution

Assala, Manal. Department of internal medicine, Assistance publique-Hopitaux de Paris, hopitaux universitaires Paris Centre-Cochin, 75014 Paris, France.

Groh, Matthieu. Department of internal medicine, Assistance publique-Hopitaux de Paris, hopitaux universitaires Paris Centre-Cochin, 75014 Paris, France; Paris Descartes university, Sorbonne Paris Cite, 75006 Paris, France.

Blanche, Philippe. Department of internal medicine, Assistance publique-Hopitaux de Paris, hopitaux universitaires Paris Centre-Cochin, 75014 Paris, France.

Vinter, Christine. Department of internal medicine, Assistance publique-Hopitaux de Paris, hopitaux universitaires Paris Centre-Cochin, 75014 Paris, France.

Cohen, Patrice. Department of internal medicine, Assistance publique-Hopitaux de Paris, hopitaux universitaires Paris Centre-Cochin, 75014 Paris, France.

Le Guern, Veronique. Department of internal medicine, Assistance publique-Hopitaux de Paris, hopitaux universitaires Paris Centre-Cochin, 75014 Paris, France.

Puechal, Xavier. Department of internal medicine, Assistance publique-Hopitaux de Paris, hopitaux universitaires Paris Centre-Cochin, 75014 Paris, France.

Mouthon, Luc. Department of internal medicine, Assistance publique-Hopitaux de Paris, hopitaux universitaires Paris Centre-Cochin, 75014 Paris, France; Paris Descartes university, Sorbonne Paris Cite, 75006 Paris, France.

Le Jeunne, Claire. Department of internal medicine, Assistance publique-Hopitaux de Paris, hopitaux universitaires Paris Centre-Cochin, 75014 Paris, France; Paris Descartes university, Sorbonne Paris Cite, 75006 Paris, France.

Launay, Odile. CIC Cochin Pasteur, Assistance publique-Hopitaux de Paris, hopitaux universitaires Paris Centre-Cochin, 75014 Paris, France; Inserm CIC 1417, Paris Descartes university, Sorbonne Paris Cite, Paris, France.

Kerneis, Solen. Inserm CIC 1417, Paris Descartes university, Sorbonne Paris Cite, Paris, France; Antimicrobial Stewardship Team, Assistance publique-Hopitaux de Paris, hopitaux universitaires Paris Centre-Cochin, 75014 Paris, France. Electronic address: solen.kerneis@aphp.fr.

Comments

Comment on (CON)

Publication Type

Letter. Comment.

Year of Publication

2019

<65>

Unique Identifier

30025958

Title

Obstacles and motivations to influenza and pneumococcal vaccination in patients with rheumatoid arthritis. A qualitative study. Comment on: "Pneumococcal and influenza vaccination rates in patients treated with corticosteroids and/or immunosuppressive therapies for systemic auto-immune diseases: A cross-sectional study" by Assala et al., Joint Bone Spine 2017;84:365-6.

Source

Joint, Bone, Spine: Revue du Rhumatisme. 86(1):117-118, 2019 01.

VI 1

Status

MEDLINE

Authors

Fayet F; Savel C; Rodere M; Soubrier M; Mathieu S.

Authors Full Name

Fayet, Francoise; Savel, Carine; Rodere, Malory; Soubrier, Martin; Mathieu, Sylvain.

Institution

Fayet, Francoise. Service de rhumatologie, CHU Gabriel-Montpied, 58, rue Montalembert, 63003 Clermont-Ferrand, France.
Savel, Carine. Service de rhumatologie, CHU Gabriel-Montpied, 58, rue Montalembert, 63003 Clermont-Ferrand, France.
Rodere, Malory. Service de rhumatologie, CHU Gabriel-Montpied, 58, rue Montalembert, 63003 Clermont-Ferrand, France.
Soubrier, Martin. Service de rhumatologie, CHU Gabriel-Montpied, 58, rue Montalembert, 63003 Clermont-Ferrand, France.
Mathieu, Sylvain. Service de rhumatologie, CHU Gabriel-Montpied, 58, rue Montalembert, 63003 Clermont-Ferrand, France. Electronic address: smathieu@chu-clermontferrand.fr.
Comments
Comment on (CON)
Comment in (CIN)
Publication Type
Letter. Comment.
Year of Publication
2019

<66>
Unique Identifier
29125905
Title
Arthritis After Cancer Immunotherapy: Symptom Duration and Treatment Response.
Arthritis care & research. 71(3):362-366, 2019 03.

Authors
Smith MH; Bass AR.
Authors Full Name
Smith, Melanie H; Bass, Anne R.
Institution
Smith, Melanie H. New York-Presbyterian Hospital/Weill Cornell Medicine, New York, New York.
Bass, Anne R. Weill Cornell Medicine and Hospital for Special Surgery, New York, New York.
Abstract

OBJECTIVE: Musculoskeletal manifestations of immune-related adverse events (irAEs) after checkpoint inhibitor immunotherapy for cancer remain incompletely characterized and poorly understood. A recently published case series suggested that immunotherapy-induced arthritis is an aggressive process requiring high-dose corticosteroids.

METHODS: This was a retrospective chart review of all patients with musculoskeletal irAEs first seen by one of the authors between 2014 and 2016. All patients had been treated for a malignancy with immune checkpoint inhibitors targeting PD-1 (nivolumab, pembrolizumab), PD-L1 (durvalumab), and/or CTLA-4 (ipilimumab, tremelimumab) at Memorial Sloan Kettering Cancer Center.

RESULTS: We identified 10 patients with a mean +/- SD age of 63.2 +/- 9.7 years. Seven were treated with a combination of checkpoint inhibitors and 3 with nivolumab monotherapy. Four patients developed inflammatory polyarthritis, 4 oligoarthritis, and 2 tenosynovitis. Six were antinuclear antibody positive and 2 had anti-cyclic citrullinated peptide antibodies. Mean +/- SD time from the first dose of immunotherapy until joint involvement was 6.3 +/- 4.3 months. All 10 patients were treated with systemic corticosteroids, but 6 of 10 required <=20 mg per day of prednisone. Five patients received steroid-sparing agents. Mean +/- SD time until resolution of joint symptoms after the last dose of immunotherapy was 9.2 +/- 6.1 months.

CONCLUSION: Musculoskeletal irAEs can manifest as a rheumatoid arthritis-like polyarthritis, oligoarthritis, tenosynovitis, or polymyalgia rheumatica. Musculoskeletal symptoms can last more than a year, but they can generally be managed with low to moderate doses of corticosteroids.
Copyright © 2017, American College of Rheumatology.
Publication Type
Journal Article.
Year of Publication
2019

<67>
Unique Identifier
28379880
Title

Immunoresponsive Autonomic Neuropathy in Sjogren Syndrome-Case Series and Literature Review. [Review]

Source

American Journal of Therapeutics. 26(1):e66-e71, 2019 Jan/Feb.

VI 1

Status

MEDLINE

Authors

Goodman BP.

Authors Full Name

Goodman, Brent P.

Abstract

BACKGROUND: Sjogren syndrome (SS) is one of the most common autoimmune disorders that classically affects exocrine glands, resulting in keratoconjunctivitis sicca and xerostomia, and frequently is associated with other systemic symptoms. SS appears to have a particular predilection for involving the autonomic nervous system.

STUDY QUESTION: Does immunotherapy improve signs and symptoms of autonomic nervous system impairment in SS?

STUDY DESIGN: This is a retrospective review of patients seen in the autonomic clinic at our institution who underwent an evaluation for a suspected autonomic disorder that ultimately was attributed to SS. SS patients who were treated with immunotherapy and completed autonomic testing before and after treatment were included in this review.

RESULTS: A total of 4 patients were identified who were treated for SS-related autonomic dysfunction with immunotherapy and underwent repeat autonomic testing after treatment. Marked clinical and functional improvement was seen after treatment with intravenous immunoglobulin in all patients and adjunctive rituximab therapy in 1 patient. The clinical improvement with immunotherapy in these patients correlated with markedly improved findings on autonomic testing in all.

MEASURES AND OUTCOMES: Clinical symptoms and results of autonomic testing prior to and following immunotherapy were assessed.

CONCLUSIONS: Autonomic signs and symptoms in SS are potentially immunoresponsive, but immunotherapy in these patients may require repeated, ongoing, or adjunctive therapy for optimal and sustained improvement.

Publication Type

Case Reports. Journal Article. Review.

Year of Publication

2019

<68>

Unique Identifier

31427051

Title

Management of vaccination in rheumatic disease. [Review]

Source

Best Practice & Research in Clinical Rheumatology. 32(6):720-734, 2018 12.

VI 1

Status

MEDLINE

Authors

Wong PKK; Hanrahan P.

Authors Full Name

Wong, Peter K K; Hanrahan, Patrick.

Institution

Wong, Peter K K. Mid-North Coast Arthritis Clinic, Coffs Harbour and University of New South Wales Rural Clinical School, PO Box 6307, Coffs Harbour, NSW, 2450, Australia. Electronic address: Peter.Wong2@health.nsw.gov.au.

Hanrahan, Patrick. Private Rheumatology Practice, South Perth, WA and Medical School, University of Western Australia, 13/38 Meadowvale Avenue, South Perth, WA, 6151, Australia. Electronic address: p.hanrahan@mac.com.

Abstract

Autoimmune inflammatory rheumatic diseases (AIIRD) such as rheumatoid arthritis and spondyloarthritis, including psoriatic arthritis and ankylosing spondylitis are associated with an increased risk of infection due to a combination of the immunosuppressive effect of the AIIRD,

comorbidities, and use of corticosteroids and the immunosuppressive effect of conventional synthetic disease-modifying antirheumatic drugs (DMARDs), targeted synthetic (ts-) DMARDs, and biologic (b-) DMARDs. Many infections are preventable with vaccination. However, as the protective immune responses induced by vaccination may be impaired by immunosuppression, vaccination should be considered before the commencement of immunosuppression. Another opportune time to review vaccination status is when planning overseas travel, as destination-specific vaccines are often required. Although limited published data regarding vaccine efficacy in patients with AIIRD make prescriptive guidelines difficult, a vaccination history should be part of the initial workup in all patients with AIIRD. Unfortunately, this is often not done by rheumatologists. This paper encourages those caring for patients with AIIRD to regularly review vaccination status. Copyright © 2019 Elsevier Ltd. All rights reserved.

Publication Type

Journal Article. Review.

Year of Publication

2018

<69>

Unique Identifier

30543737

Title

[A Case of Rheumatoid Arthritis Developed during Treatment with Nivolumab for Renal Cell Carcinoma]. [Review] [Japanese]

Source

Hinyokika Kiyo - Acta Urologica Japonica. 64(10):397-401, 2018 10.

VI 1

Status

MEDLINE

Authors

Tomizawa M; Nakai Y; Maesaka F; Owari T; Hara R; Miyake M; Inoue T; Anai S; Tanaka N; Fujimoto K.

Authors Full Name

Tomizawa, Mitsuru; Nakai, Yasushi; Maesaka, Fumisato; Owari, Takuya; Hara, Ryota; Miyake, Makito; Inoue, Takeshi; Anai, Satoshi; Tanaka, Nobumichi; Fujimoto, Kiyohide.

Institution
Tomizawa, Mitsuru. The Department of Urology, Nara Medical University.
Nakai, Yasushi. The Department of Urology, Nara Medical University.
Maesaka, Fumisato. The Department of Urology, Nara Medical University.
Owari, Takuya. The Department of Urology, Nara Medical University.
Hara, Ryota. The Department af Orthapedics, Nara Medical University.
Miyake, Makito. The Department of Urology, Nara Medical University.
Inoue, Takeshi. The Department of Urology, Nara Medical University.
Anai, Satoshi. The Department of Urology, Nara Medical University.
Tanaka, Nobumichi. The Department of Urology, Nara Medical University.
Fujimoto, Kiyohide. The Department of Urology, Nara Medical University.
Abstract
A 66-year-old man underwent nephrectomy for right renal cell carcinoma (cT3bNOMl (PUL)). Thereafter, he was treated with sunitinib for lung metastasis as the first-line therapy for 5 months and then axitinib as the second-line therapy for 2 months. Because lung metastasis progressed despite molecular targeted therapies, nivolumab was used as the third-line treatment. Three months later, he complained of painful stiffness in hands and wrist joints symmetrically. He was diagnosed as having rheumatoid arthritis. Treatment with nivolumab was discontinued and prednisolone and methotrexate were started. Although the painful stiffness in joints was improved l month later, synovitis remained partially 6 months after starting treatment of disease with anti-rheumatic drugs. Therefore, treatment for rheumatoid arthritis was continued. On the other hand, because the lung lesion had progressed 2 months after discontining nivolumab, everolimus was used as the fourth-line therapy.
Publication Type
Case Reports. Journal Article. Review.
Year of Publication
2018

<70>
Unique Identifier
30537799
Title

Collagen II-primed Foxp3 Transduced T Cells Ameliorate Collagen-induced Arthritis in Rats: The Effect of Antigenic Priming on T Regulatory Cell Function.

Source

Iranian Journal of Allergy Asthma & Immunology. 17(4):361-371, 2018 Aug 12.

VI 1

Status

MEDLINE

Authors

Zavvar M; Abdolmaleki M; Farajifard H; Noorbakhsh F; Azadmanesh K; Vojgani M; Nikcnam MH.

Authors Full Name

Zavvar, Mahdi; Abdolmaleki, Mohsen; Farajifard, Hamid; Noorbakhsh, Farshid; Azadmanesh, Kayhan; Vojgani, Mohammad; Nikcnam, Mohammad Hossein.

Institution

Zavvar, Mahdi. Department of Immunology, School of Medicine, Tehran University of Medical Sciences, Tehran, Iran. Mahdi.zavvar@gmail.com.

Abdolmaleki, Mohsen. Department of Immunology, School of Medicine, Tehran University of Medical Sciences, Tehran, Iran. mohsenmaleki1@yahoo.com.

Farajifard, Hamid. Department of Immunology, School of Medicine, Tehran University of Medical Sciences, Tehran, Iran. hamid.farajifard@yahoo.com.

Noorbakhsh, Farshid. Department of Immunology, School of Medicine, Tehran University of Medical Sciences, Tehran, Iran. f-noorbakhsh@tums.ac.ir.

Azadmanesh, Kayhan. Department of Virology, Pasteur Institute of Iran, Tehran, Iran. azadmanesh@pasteur.ac.ir.

Vojgani, Mohammad. Department of Immunology, School of Medicine, Tehran University of Medical Sciences, Tehran, Iran. Vojganim@tums.ac.ir.

Nikcnam, Mohammad Hossein. Department of Immunology, School of Medicine, Tehran University of Medical Sciences, Tehran, Iran AND Molecular Immunology Research Center, Children's Medical Center, Tehran University of Medical Sciences, Tehran, Iran. nicknam_m@yahoo.com.

Abstract

Regulatory T cells (Tregs) play a major role in the prevention of autoimmune diseases. Transfer of Foxp3 gene into conventional T cells converts their phenotype to regulatory T cells. Therefore, the question arises as to whether adoptively transferred in vitro differentiated Treg cells specific for a locally expressed antigen might have better inhibitory effects on the progression of the disease as compared with antigen-nonspecific T reg cells. Herein, we investigated the therapeutic potential of primed and unprimed retrovirus mediated Foxp3-overexpression T cells following

intravenously injected of these cells into affected rats with collagen-induced arthritis (CIA), an animal model of rheumatoid arthritis. Our analyses demonstrate that systemic administration of collagen II primed Foxp3-transduced T cells could markedly ameliorate CIA inflammatory responses at clinical (p<0.0014) and pathological exchanges including cellular infiltration (p=0.002), bone erosion (p=0.0013) and synovial hyperplasia (p=0.002). In contrast, collagen II unprimed Foxp3-transduced T cells like as collagen II primed or unprimed GFP-transduced T cells did not reveal any beneficial effects on arthritis features as compared with untreated group (p>0.05). Therefore, we believe that collagen II primed Foxp3-transduced T cells are interacting locally and systemically with immune cells which reveled with decreasing of T cells infiltration into joints along with specific CII IgG production. Considering the results described here, it appears that the using patients' T cells which previously exposed to specific antigens may have more effective therapeutic advantage in the production of induced regulatory T cells in the treatment of arthritis.

Publication Type

Journal Article.

Year of Publication

2018

<71>

Unique Identifier

30530397

Title

Successful Slow Desensitization to Tocilizumab in a 15-Year-Old Patient.

Source

Journal of Investigational Allergology & Clinical Immunology. 28(6):436-438, 2018 Dec.

VI 1

Status

MEDLINE

Authors

Cansever M; Sahin N; Dursun I; Geyik C; Dusunsel R; Bektas Kut F; Tahan F.

Authors Full Name

Cansever, M; Sahin, N; Dursun, I; Geyik, C; Dusunsel, R; Bektas Kut, F; Tahan, F.

Institution

Cansever, M. Erciyes University, School of Medicine, Department of Pediatrics, Division of Allergy, Kayseri, Turkey.
Sahin, N. Erciyes University, School of Medicine, Department of Pediatrics, Division of Rheumatology, Kayseri, Turkey.
Dursun, I. Erciyes University, School of Medicine, Department of Pediatrics, Division of Rheumatology, Kayseri, Turkey.
Geyik, C. Erciyes University, School of Medicine, Department of Pediatrics, Kayseri, Turkey.
Dusunsel, R. Erciyes University, School of Medicine, Department of Pediatrics, Division of Rheumatology, Kayseri, Turkey.
Bektas Kut, F. Erciyes University, School of Medicine, Department of Pediatrics, Division of Allergy, Kayseri, Turkey.
Tahan, F. Erciyes University, School of Medicine, Department of Pediatrics, Division of Allergy, Kayseri, Turkey.

Publication Type
Journal Article.
Year of Publication
2018

<72>
Unique Identifier
30487791
Title
Sulforaphane Inhibits Inflammatory Responses of Primary Human T-Cells by Increasing ROS and Depleting Glutathione.
Source
Frontiers in Immunology. 9:2584, 2018.
VI 1
Status
MEDLINE
Authors
Liang J; Jahraus B; Balta E; Ziegler JD; Hubner K; Blank N; Niesler B; Wabnitz GH; Samstag Y.
Authors Full Name

Liang, Jie; Jahraus, Beate; Balta, Emre; Ziegler, Jacqueline D; Hubner, Katrin; Blank, Norbert; Niesler, Beate; Wabnitz, Guido H; Samstag, Yvonne.
Institution
Liang, Jie. Section Molecular Immunology, Institute of Immunology, Heidelberg University, Heidelberg, Germany.
Jahraus, Beate. Section Molecular Immunology, Institute of Immunology, Heidelberg University, Heidelberg, Germany.
Balta, Emre. Section Molecular Immunology, Institute of Immunology, Heidelberg University, Heidelberg, Germany.
Ziegler, Jacqueline D. Section Molecular Immunology, Institute of Immunology, Heidelberg University, Heidelberg, Germany.
Hubner, Katrin. Section Molecular Immunology, Institute of Immunology, Heidelberg University, Heidelberg, Germany.
Blank, Norbert. Division of Rheumatology, Department of Internal Medicine V, Heidelberg University, Heidelberg, Germany.
Niesler, Beate. Department of Human Molecular Genetics, Heidelberg University, Heidelberg, Germany.
Niesler, Beate. nCounter Core Facility, Department of Human Molecular Genetics, Heidelberg University, Heidelberg, Germany.
Wabnitz, Guido H. Section Molecular Immunology, Institute of Immunology, Heidelberg University, Heidelberg, Germany.
Samstag, Yvonne. Section Molecular Immunology, Institute of Immunology, Heidelberg University, Heidelberg, Germany.
Abstract
The activity and function of T-cells are influenced by the intra- and extracellular redox milieu. Oxidative stress induces hypo responsiveness of untransformed T-cells. Vice versa increased glutathione (GSH) levels or decreased levels of reactive oxygen species (ROS) prime T-cell metabolism for inflammation, e.g., in rheumatoid arthritis. Therefore, balancing the T-cell redox milieu may represent a promising new option for therapeutic immune modulation. Here we show that sulforaphane (SFN), a compound derived from plants of the Brassicaceae family, e.g., broccoli, induces a pro-oxidative state in untransformed human T-cells of healthy donors or RA patients. This manifested as an increase of intracellular ROS and a marked decrease of GSH. Consistently, increased global cysteine sulfenylation was detected. Importantly, a major target for SFN-mediated protein oxidation was STAT3, a transcription factor involved in the regulation of TH17-related genes. Accordingly, SFN significantly inhibited the activation of untransformed human T-cells derived from healthy donors or RA patients, and downregulated the expression of the transcription factor RORgammat, and the TH17-related cytokines IL-17A, IL-17F, and IL-22,

which play a major role within the pathophysiology of many chronic inflammatory/autoimmune diseases. The inhibitory effects of SFN could be abolished by exogenously supplied GSH and by the GSH replenishing antioxidant N-acetylcysteine (NAC). Together, our study provides mechanistic insights into the mode of action of the natural substance SFN. It specifically exerts TH17 prone immunosuppressive effects on untransformed human T-cells by decreasing GSH and accumulation of ROS. Thus, SFN may offer novel clinical options for the treatment of TH17 related chronic inflammatory/autoimmune diseases such as rheumatoid arthritis.

Publication Type

Journal Article. Research Support, Non-U.S. Gov't.

Year of Publication

2018

<73>

Unique Identifier

30442497

Title

Cardiovascular toxicities associated with immune checkpoint inhibitors: an observational, retrospective, pharmacovigilance study.

Source

Lancet Oncology. 19(12):1579-1589, 2018 12.

VI 1

Status

MEDLINE

Authors

Salem JE; Manouchehri A; Moey M; Lebrun-Vignes B; Bastarache L; Pariente A; Gobert A; Spano JP; Balko JM; Bonaca MP; Roden DM; Johnson DB; Moslehi JJ.

Authors Full Name

Salem, Joe-Elie; Manouchehri, Ali; Moey, Melissa; Lebrun-Vignes, Benedicte; Bastarache, Lisa; Pariente, Antoine; Gobert, Aurelien; Spano, Jean-Philippe; Balko, Justin M; Bonaca, Marc P; Roden, Dan M; Johnson, Douglas B; Moslehi, Javid J.

Institution

Salem, Joe-Elie. Regional Pharmacovigilance Centre, Department of Pharmacology, Sorbonne Universite, INSERM CIC Paris-Est, Assistance Publique Hopitaux de Paris, Institute of

Cardiometabolism and Nutrition, Pitie-Salpetriere Hospital, Paris, France; Cardio-Oncology Program, Department of Medicine, Vanderbilt University Medical Center, Nashville, TN, USA.
Manouchehri, Ali. Cardio-Oncology Program, Department of Medicine, Vanderbilt University Medical Center, Nashville, TN, USA.
Moey, Melissa. Cardio-Oncology Program, Department of Medicine, Vanderbilt University Medical Center, Nashville, TN, USA.
Lebrun-Vignes, Benedicte. Regional Pharmacovigilance Centre, Department of Pharmacology, Sorbonne Universite, INSERM CIC Paris-Est, Assistance Publique Hopitaux de Paris, Institute of Cardiometabolism and Nutrition, Pitie-Salpetriere Hospital, Paris, France.
Bastarache, Lisa. Department of Biomedical Informatics, Vanderbilt University Medical Center, Nashville, TN, USA.
Pariente, Antoine. INSERM UMR 1219, Bordeaux Population Health Research Center, Team Pharmacoepidemiology, CHU de Bordeaux, Pole de Sante Publique, Pharmacologie Medicale, Centre de Pharmacovigilance de Bordeaux, University of Bordeaux, Bordeaux, France.
Gobert, Aurelien. Department of Medicine, Sorbonne Universite, INSERM CIC Paris-Est, Assistance Publique Hopitaux de Paris, Institute of Cardiometabolism and Nutrition, Pitie-Salpetriere Hospital, Paris, France.
Spano, Jean-Philippe. Department of Medicine, Sorbonne Universite, INSERM CIC Paris-Est, Assistance Publique Hopitaux de Paris, Institute of Cardiometabolism and Nutrition, Pitie-Salpetriere Hospital, Paris, France.
Balko, Justin M. Cardio-Oncology Program, Department of Medicine, Vanderbilt University Medical Center, Nashville, TN, USA.
Bonaca, Marc P. Brigham and Women's Hospital, Harvard Medical School, Boston, MA, USA.
Roden, Dan M. Cardio-Oncology Program, Department of Medicine, Vanderbilt University Medical Center, Nashville, TN, USA; Department of Biomedical Informatics, Vanderbilt University Medical Center, Nashville, TN, USA.
Johnson, Douglas B. Cardio-Oncology Program, Department of Medicine, Vanderbilt University Medical Center, Nashville, TN, USA.
Moslehi, Javid J. Cardio-Oncology Program, Department of Medicine, Vanderbilt University Medical Center, Nashville, TN, USA. Electronic address: javid.moslehi@vanderbilt.edu.
Comments
Comment in (CIN)
Abstract
BACKGROUND: Immune checkpoint inhibitors (ICIs) have substantially improved clinical outcomes in multiple cancer types and are increasingly being used in early disease settings and in combinations of different immunotherapies. However, ICIs can also cause severe or fatal

immune-related adverse-events (irAEs). We aimed to identify and characterise cardiovascular irAEs that are significantly associated with ICIs.

METHODS: In this observational, retrospective, pharmacovigilance study, we used VigiBase, WHO's global database of individual case safety reports, to compare cardiovascular adverse event reporting in patients who received ICIs (ICI subgroup) with this reporting in the full database. This study included all cardiovascular irAEs classified by group queries according to the Medical Dictionary for Regulatory Activities, between inception on Nov 14, 1967, and Jan 2, 2018. We evaluated the association between ICIs and cardiovascular adverse events using the reporting odds ratio (ROR) and the information component (IC). IC is an indicator value for disproportionate Bayesian reporting that compares observed and expected values to find associations between drugs and adverse events. IC025 is the lower end of the IC 95% credibility interval, and an IC025 value of more than zero is deemed significant. This study is registered with ClinicalTrials.gov, number NCT03387540.

FINDINGS: We identified 31 321 adverse events reported in patients who received ICIs and 16 343 451 adverse events reported in patients treated with any drugs (full database) in VigiBase. Compared with the full database, ICI treatment was associated with higher reporting of myocarditis (5515 reports for the full database vs 122 for ICIs, ROR 11.21 [95% CI 9.36-13.43]; IC025 3.20), pericardial diseases (12 800 vs 95, 3.80 [3.08-4.62]; IC025 1.63), and vasculitis (33 289 vs 82, 1.56 [1.25-1.94]; IC025 0.03), including temporal arteritis (696 vs 18, 12.99 [8.12-20.77]; IC025 2.59) and polymyalgia rheumatica (1709 vs 16, 5.13 [3.13-8.40]; IC025 1.33). Pericardial diseases were reported more often in patients with lung cancer (49 [56%] of 87 patients), whereas myocarditis (42 [41%] of 103 patients) and vasculitis (42 [60%] of 70 patients) were more commonly reported in patients with melanoma (chi2 test for overall subgroup comparison, p<0.0001). Vision was impaired in five (28%) of 18 patients with temporal arteritis. Cardiovascular irAEs were severe in the majority of cases (>80%), with death occurring in 61 (50%) of 122 myocarditis cases, 20 (21%) of 95 pericardial disease cases, and five (6%) of 82 vasculitis cases (chi2 test for overall comparison between pericardial diseases, myocarditis, and vasculitis, p<0.0001).

INTERPRETATION: Treatment with ICIs can lead to severe and disabling inflammatory cardiovascular irAEs soon after commencement of therapy. In addition to life-threatening myocarditis, these toxicities include pericardial diseases and temporal arteritis with a risk of blindness. These events should be considered in patient care and in combination clinical trial designs (ie, combinations of different immunotherapies as well as immunotherapies and chemotherapy).

FUNDING: The Cancer Institut Thematique Multi-Organisme of the French National Alliance for Life and Health Sciences (AVIESAN) Plan Cancer 2014-2019; US National Cancer Institute, National Institutes of Health; the James C. Bradford Jr. Melanoma Fund; and the Melanoma Research Foundation. Copyright © 2018 Elsevier Ltd. All rights reserved.

Publication Type

Journal Article. Observational Study. Research Support, N.I.H., Extramural. Research Support, Non-U.S. Gov't.

Year of Publication

2018

<74>

Unique Identifier

30439628

Title

Rheumatic immune-related adverse events secondary to anti-programmed death-1 antibodies and preliminary analysis on the impact of corticosteroids on anti-tumour response: A case series. European Journal of Cancer. 105:88-102, 2018 12.

Authors

Mitchell EL; Lau PKH; Khoo C; Liew D; Leung J; Liu B; Rischin A; Frauman AG; Kee D; Smith K; Brady B; Rischin D; Gibson A; Mileshkin L; Klein O; Weickhardt A; Arulananda S; Shackleton M; McArthur G; Ostor A; Cebon J; Solomon B; Buchanan RR; Wicks IP; Lo S; Hicks RJ; Sandhu S.

Authors Full Name

Mitchell, Emma L; Lau, Peter Kar Han; Khoo, Chloe; Liew, David; Leung, Jessica; Liu, Bonnia; Rischin, Adam; Frauman, Albert G; Kee, Damien; Smith, Kortnye; Brady, Benjamin; Rischin, Danny; Gibson, Andrew; Mileshkin, Linda; Klein, Oliver; Weickhardt, Andrew; Arulananda, Surein; Shackleton, Mark; McArthur, Grant; Ostor, Andrew; Cebon, Jonathan; Solomon, Benjamin; Buchanan, Russell Rc; Wicks, Ian P; Lo, Serigne; Hicks, Rodney J; Sandhu, Shahneen.

Institution

Mitchell, Emma L. Department of Rheumatology, Royal Melbourne Hospital, Australia; Department of Rheumatology, Austin Health, Melbourne, Australia.

Lau, Peter Kar Han. Department of Medical Oncology, Peter MacCallum Cancer Centre, Melbourne, Australia.

Khoo, Chloe. Department of Medical Oncology, Peter MacCallum Cancer Centre, Melbourne, Australia.

Liew, David. Department of Rheumatology, Austin Health, Melbourne, Australia; Department of Clinical Pharmacology and Therapeutics, Austin Health, Melbourne, Australia; Department of Medicine, University of Melbourne, Australia.

Leung, Jessica. Department of Rheumatology, Royal Melbourne Hospital, Australia; Department of Rheumatology, Austin Health, Melbourne, Australia.

Liu, Bonnia. Department of Rheumatology, Austin Health, Melbourne, Australia.

Rischin, Adam. Alfred Hospital, Melbourne, Australia.

Frauman, Albert G. Department of Clinical Pharmacology and Therapeutics, Austin Health, Melbourne, Australia; Department of Medicine, University of Melbourne, Australia.

Kee, Damien. Department of Medical Oncology, Peter MacCallum Cancer Centre, Melbourne, Australia.

Smith, Kortnye. Department of Medical Oncology, Peter MacCallum Cancer Centre, Melbourne, Australia.

Brady, Benjamin. Department of Medical Oncology, Peter MacCallum Cancer Centre, Melbourne, Australia; Cabrini Health, Melbourne, Australia.

Rischin, Danny. Department of Medical Oncology, Peter MacCallum Cancer Centre, Melbourne, Australia; Sir Peter MacCallum Department, University of Melbourne, Melbourne, Australia.

Gibson, Andrew. Cabrini Health, Melbourne, Australia.

Mileshkin, Linda. Department of Medical Oncology, Peter MacCallum Cancer Centre, Melbourne, Australia; Sir Peter MacCallum Department, University of Melbourne, Melbourne, Australia.

Klein, Oliver. Olivia Newton-John Cancer and Wellness Centre, Heidelberg, Victoria, Australia.

Weickhardt, Andrew. Olivia Newton-John Cancer and Wellness Centre, Heidelberg, Victoria, Australia.

Arulananda, Surein. Olivia Newton-John Cancer and Wellness Centre, Heidelberg, Victoria, Australia.

Shackleton, Mark. Alfred Hospital, Melbourne, Australia.

McArthur, Grant. Department of Medical Oncology, Peter MacCallum Cancer Centre, Melbourne, Australia; Sir Peter MacCallum Department, University of Melbourne, Melbourne, Australia.

Ostor, Andrew. Cabrini Health, Melbourne, Australia.

Cebon, Jonathan. Department of Medicine, University of Melbourne, Australia; Olivia Newton-John Cancer and Wellness Centre, Heidelberg, Victoria, Australia.

Solomon, Benjamin. Department of Medical Oncology, Peter MacCallum Cancer Centre, Melbourne, Australia; Sir Peter MacCallum Department, University of Melbourne, Melbourne, Australia.

Buchanan, Russell Rc. Department of Rheumatology, Austin Health, Melbourne, Australia; Department of Medicine, University of Melbourne, Australia.

Wicks, Ian P. Department of Rheumatology, Royal Melbourne Hospital, Australia; Walter and Eliza Hall Institute, Melbourne, Australia.

Lo, Serigne. Melanoma Institute Australia, University of Sydney, Sydney, New South Wales, Australia; Institute for Research and Medical Consultations, University of Dammam, Dammam, Saudi Arabia.

Hicks, Rodney J. Sir Peter MacCallum Department, University of Melbourne, Melbourne, Australia.

Sandhu, Shahneen. Department of Medical Oncology, Peter MacCallum Cancer Centre, Melbourne, Australia; Sir Peter MacCallum Department, University of Melbourne, Melbourne, Australia. Electronic address: shahneen.sandhu@petermac.org.

Abstract

IMPORTANCE: Rheumatic immune-related adverse events (irAEs) occur in approximately 10-20% of anti-programmed death 1 (anti-PD1)-treated cancer patients. There are limited data on the natural history, optimal treatment and long-term oncological outcomes of patients with rheumatic irAEs.

OBJECTIVE: The objective of the study was to describe the spectrum and natural history of rheumatic irAEs and the potential impact of rheumatic irAEs and immunomodulators on anti-PD1 tumour efficacy.

METHODS: Cancer patients with pre-existing rheumatic disease before anti-PD1 therapy or de novo rheumatic irAEs on anti-PD1 therapy were retrospectively reviewed across three sites. Patient demographics, treatment history, anti-PD1 irAEs, and anti-PD1 responses were evaluated. Relationships between the development or pre-existence of rheumatic irAE, use of immunomodulatory agents and outcomes were evaluated.

RESULTS: This multicenter case series describes 36 cancer patients who had rheumatic disease before anti-PD1 therapy (n = 12) or developed de novo rheumatic irAEs (n = 24). Thirty-four of the 36 patients sustained rheumatic irAEs (median time to rheumatic irAE: 14.5 weeks), including 24 de novo (18 inflammatory arthritis, three myositis, two polymyalgia rheumatica, one

fasciitis) and 10 flares in 12 patients with pre-existing rheumatic disease. Corticosteroids were used in 30 of 36 patients (median duration: 10 months), and disease-modifying antirheumatic drugs were used in 14 of 36 patients (median duration: 5.5 months). The objective response rate to anti-PD1 therapy was 69% (n = 25/36) overall and 81% (n = 21/26) in the melanoma subgroup.

CONCLUSIONS: Rheumatic irAEs are often chronic and require prolonged immunomodulatory therapy. Prospective studies are required to define optimal management of rheumatic irAEs that maintain long-term anticancer outcomes. Crown Copyright © 2018. Published by Elsevier Ltd. All rights reserved.

Publication Type

Comparative Study. Journal Article. Multicenter Study.

Year of Publication

2018

<75>

Unique Identifier

30417018

Title

Anti-ICOSL New Antigen Receptor Domains Inhibit T Cell Proliferation and Reduce the Development of Inflammation in the Collagen-Induced Mouse Model of Rheumatoid Arthritis.

Source

Journal of Immunological Research. 2018:4089459, 2018.

VI 1

Status

MEDLINE

Authors

O'Dwyer R; Kovaleva M; Zhang J; Steven J; Cummins E; Luxenberg D; Darmanin-Sheehan A; Carvalho MF; Whitters M; Saunders K; Barelle CJ.

Author NameID

Kovaleva, Marina; ORCID: https://orcid.org/0000-0001-7614-8261

Darmanin-Sheehan, Alfredo; ORCID: https://orcid.org/0000-0001-5280-7090

Carvalho, Miguel F; ORCID: https://orcid.org/0000-0002-0644-3860

Barelle, Caroline J; ORCID: https://orcid.org/0000-0003-1872-3373

Authors Full Name

O'Dwyer, Ronan; Kovaleva, Marina; Zhang, Jiquan; Steven, John; Cummins, Emma; Luxenberg, Deborah; Darmanin-Sheehan, Alfredo; Carvalho, Miguel F; Whitters, Matthew; Saunders, Kenneth; Barelle, Caroline J.

Institution

O'Dwyer, Ronan. Immunocore, Long Wittenham, Oxfordshire OX14 4RY, UK.

Kovaleva, Marina. Elasmogen Ltd, Aberdeen AB25 2ZP, UK.

Zhang, Jiquan. Novartis Institutes for BioMedical Research (NIBR), Shanghai 201203, China.

Steven, John. Elasmogen Ltd, Aberdeen AB25 2ZP, UK.

Cummins, Emma. The Centre for Drug Research and Development (CDRD), Vancouver, BC, Canada V6T 1Z3.

Luxenberg, Deborah. Pfizer, Cambridge Park Drive, Cambridge, MA 02140, USA.

Darmanin-Sheehan, Alfredo. Pfizer; Global Biotherapeutics Technologies, Dublin D22, Ireland.

Carvalho, Miguel F. Pfizer, Cambridge Park Drive, Cambridge, MA 02140, USA.

Whitters, Matthew. Pfizer, Cambridge Park Drive, Cambridge, MA 02140, USA.

Saunders, Kenneth. UCB New Medicines, B1420 Braine-l'Alleud, Belgium.

Barelle, Caroline J. Elasmogen Ltd, Aberdeen AB25 2ZP, UK.

Abstract

Lymphocyte costimulation plays a central role in immunology, inflammation, and immunotherapy. The inducible T cell costimulator (ICOS) is expressed on T cells following peptide: MHC engagement with CD28 costimulation. The interaction of ICOS with its sole ligand, the inducible T cell costimulatory ligand (ICOSL; also known as B7-related protein-1), triggers a number of key activities of T cells including differentiation and cytokine production. Suppression of T cell activation can be achieved by blocking this interaction and has been shown to be an effective means of ameliorating disease in models of autoimmunity. In this study, we isolated specific anti-ICOSL new antigen receptor domains from a synthetic phage display library and demonstrated their ability to block the ICOS/ICOSL interaction and inhibit T cell proliferation. Anti-mouse ICOSL domains, considered here as surrogates for the use of anti-human ICOSL domains in patient therapy, were tested for efficacy in a collagen-induced mouse model of rheumatoid arthritis where they significantly decreased the inflammation of joints and delayed and reduced overall disease progression and severity.

Publication Type

Journal Article.

Year of Publication

2018

<76>
Unique Identifier
30366509
Title
Suppressive oligodeoxynucleotide-induced dendritic cells rein the aggravation of osteoarthritis in mice.
Source
Immunopharmacology & Immunotoxicology. 40(5):430-436, 2018 Oct.
VI 1
Status
MEDLINE
Authors
Guo W; Wei B; Sun J; Chen T; Wei J; Hu Z; Chen S; Xiang M; Shu Y; Peng Z.
Authors Full Name
Guo, Weixiong; Wei, Bo; Sun, Jiecong; Chen, Tingting; Wei, Jinsong; Hu, Zibing; Chen, Siyuan; Xiang, Min; Shu, Yang; Peng, Zhiheng.
Institution
Guo, Weixiong. a Department of Orthopedics, Affiliated Hospital of Guangdong Medical University, Zhanjiang, Guangdong, People's Republic of China.
Wei, Bo. a Department of Orthopedics, Affiliated Hospital of Guangdong Medical University, Zhanjiang, Guangdong, People's Republic of China.
Sun, Jiecong. a Department of Orthopedics, Affiliated Hospital of Guangdong Medical University, Zhanjiang, Guangdong, People's Republic of China.
Chen, Tingting. a Department of Orthopedics, Affiliated Hospital of Guangdong Medical University, Zhanjiang, Guangdong, People's Republic of China.
Wei, Jinsong. a Department of Orthopedics, Affiliated Hospital of Guangdong Medical University, Zhanjiang, Guangdong, People's Republic of China.
Hu, Zibing. a Department of Orthopedics, Affiliated Hospital of Guangdong Medical University, Zhanjiang, Guangdong, People's Republic of China.
Chen, Siyuan. a Department of Orthopedics, Affiliated Hospital of Guangdong Medical University, Zhanjiang, Guangdong, People's Republic of China.
Xiang, Min. a Department of Orthopedics, Affiliated Hospital of Guangdong Medical University, Zhanjiang, Guangdong, People's Republic of China.

Shu, Yang. b Department of Orthopedics, Wuhan Sixth Hospital Affiliated to Jianghan University, Wuhan, Hubei, People's Republic of China.
Peng, Zhiheng. a Department of Orthopedics, Affiliated Hospital of Guangdong Medical University, Zhanjiang, Guangdong, People's Republic of China.
Abstract
OBJECTIVE: To explore the effect of suppressive oligodeoxynucleotide-induced dendritic cells (S-DCs) in the osteoarthritis (OA) therapy.

METHODS: S-DCs were prepared from splenic CD11c + cells by in vitro culture with suppressive oligodeoxynucleotide. The function and phenotypes of S-DCs were measured by ELISA and flow cytometry. The innate immune signaling pathways were detected by western blotting in the non-treated DCs and S-DCs upon stimulation. In vivo, we employed an iodoacetate-induced OA mice model. S-DCs were transferred by intravenous route. The weight bearing of mice was evaluated and pro-inflammatory factors in OA joint were measured by real-time PCR. Treg cell ratio and CD4 + IL10+ cells in spleen were detected by flow cytometry at day 5 post OA induction.

RESULTS: The S-DCs showed less inflammatory phenotypes upon stimulation. The expression of pro-inflammatory cytokines and mature makers in the S-DCs were blunt, due to the impaired innate immune signal transduction. In an iodoacetate-induced OA model, transfer of S-DCs significantly controlled the process of OA. Restricted inflammatory responses were observed in the joint of S-DC recipients. Moreover, after S-DC transfer, Tregs and CD4 + IL10+ cells were mounted in the spleen.

CONCLUSION: Transfer of suppressive oligodeoxynucleotides-induced autologous DCs may represent a potential agent to control the aggravation of OA in patients.
Publication Type
Journal Article.
Year of Publication
2018

<77>
Unique Identifier

30348223

Title

A severe case of neuro-Sjogren's syndrome induced by pembrolizumab.

Source

Journal for Immunotherapy of Cancer. 6(1):110, 2018 10 22.

VI 1

Status

MEDLINE

Authors

Ghosn J; Vicino A; Michielin O; Coukos G; Kuntzer T; Obeid M.

Authors Full Name

Ghosn, Jaqueline; Vicino, Alex; Michielin, Olivier; Coukos, George; Kuntzer, Thierry; Obeid, Michel.

Institution

Ghosn, Jaqueline. Department of Medicine, Division of Immunology and Allergy, Lausanne University Hospital CHUV, Rue du Bugnon 46, CH-1011, Lausanne, Switzerland.

Vicino, Alex. Department of Neurology, Lausanne University Hospital CHUV, Rue du Bugnon 46, CH-1011, Lausanne, Switzerland.

Michielin, Olivier. Department of Medical Oncology, Lausanne University Hospital CHUV, Rue du Bugnon 46, CH-1011, Lausanne, Switzerland.

Coukos, George. Department of Medical Oncology, Lausanne University Hospital CHUV, Rue du Bugnon 46, CH-1011, Lausanne, Switzerland.

Coukos, George. Ludwig Institute for Cancer Research, Chemin des Boveresses 155, CH-1066, Epalinges, Switzerland.

Kuntzer, Thierry. Department of Neurology, Lausanne University Hospital CHUV, Rue du Bugnon 46, CH-1011, Lausanne, Switzerland.

Obeid, Michel. Department of Medicine, Division of Immunology and Allergy, Lausanne University Hospital CHUV, Rue du Bugnon 46, CH-1011, Lausanne, Switzerland. michel.obeid@chuv.ch.

Obeid, Michel. Vaccination and Immunotherapy Center, Lausanne University Hospital CHUV, Rue du Bugnon 17, CH-1011, Lausanne, Switzerland. michel.obeid@chuv.ch.

Obeid, Michel. Medical School Pitie-Salpetriere, Sorbonne University, 91 Boulevard de l'Hopital, F-75013, Paris, France. michel.obeid@chuv.ch.

Abstract

BACKGROUND: The prevalence of connective tissue disease (CTD) induced by immune checkpoint inhibitors (CPIs) in the absence of pre-existing autoimmunity is unknown.

CASE PRESENTATION: We report the case of a melanoma patient treated for 8 months with pembrolizumab who developed a subacute ataxic sensory neuronopathy (SNN), including a right trigeminal neuropathy. Salivary gland biopsy showed inflammatory changes suggestive of Sjogren's syndrome, while brain MRI revealed enhancement of the right trigeminal ganglia. A high level of protein and pleocytosis was found in the cerebrospinal fluid, with negative cultures. Nerve conduction studies revealed the absence of sensory nerve action potentials in the upper and lower limbs and reduced motor responses in the upper limbs, fulfilling criteria for SNN. Blood tests revealed an important inflammatory syndrome, hemolytic anemia, elevation of total IgG levels and the presence of ANA autoantibodies specific to anti-SSA (52 and 60 kd). All these elements were absent before the initiation of the treatment with pembrolizumab. Initially, there was a clinical response following intravenous frontline methylprednisone, but the subacute relapse required the introduction of second-line treatment with intravenous immunoglobulins and then rituximab, which led to a quick clinical improvement.

CONCLUSIONS: Herein, we describe the first case of a patient who developed a typical SNN as a complication of severe neuro-Sjogren's syndrome induced by pembrolizumab treatment.

Publication Type

Case Reports. Journal Article.

Year of Publication

2018

<78>

Unique Identifier

30246657

Title

TST, QuantiFERON-TB Gold test and T-SPOT.TB test for detecting latent tuberculosis infection in patients with rheumatic disease prior to anti-TNF therapy.

Source

Tuberkuloz ve Toraks. 66(2):136-143, 2018 06.

VI 1

Status

MEDLINE

Authors

Sargin G; Senturk T; Ceylan E; Telli M; Cildag S; Dogan H.
Authors Full Name
Sargin, Gokhan; Senturk, Taskin; Ceylan, Emel; Telli, Murat; Cildag, Songul; Dogan, Hakan.
Institution
Sargin, Gokhan. Department of Rheumatology, Faculty of Medicine, Adnan Menderes University, Aydin, Turkey.
Senturk, Taskin. Department of Rheumatology, Faculty of Medicine, Adnan Menderes University, Aydin, Turkey.
Ceylan, Emel. Department of Chest Diseases, Faculty of Medicine, Adnan Menderes University, Aydin, Turkey.
Telli, Murat. Department of Medical Microbiology, Faculty of Medicine, Adnan Menderes University, Aydin, Turkey.
Cildag, Songul. Department of Rheumatology, Faculty of Medicine, Adnan Menderes University, Aydin, Turkey.
Dogan, Hakan. Clinic of Internal Medicine, Izmir Bozyaka Training and Research Hospital, Izmir, Turkey.
Abstract
Introduction: Before starting tumour necrosis factor (TNF)-alpha blocking agents, standard tests should be used for the diagnosis of tuberculosis infection. The specificity of traditional tuberculin skin test (TST) is low in immunosuppressed patients due to prior Bacille Calmette Guerin (BCG) vaccination, non-tuberculous mycobacteria infections, false positive and negative results. In this study, we aimed to compare TST and Interferon-Gamma Release Assay (IGRA) tests for detecting latent tuberculosis infection in patients with rheumatic disease planned to receive TNF-alpha blocking agents.

Materials and Methods: One hundred and nine patients (45 male, 64 female) with the diagnosis of rheumatoid arthritis (RA) (n= 70) and ankylosing spondylitis (AS) (n= 39) were included in the study. Age, sex, number of BCG scar, results of TST (using the Mantoux method), QuantiFERON-TB Gold test and T-SPOT.TB test were recorded for all patients. Correlation between the tests was assessed by Pearson correlation coefficient.

Result: The mean age of RA and AS patients were 50 +/- 13 (19-78 years). The prevalence of latent tuberculosis was 43.1% for TST, 39.4% for QuantiFERON-TB Gold test and 13.8% for T-SPOT.TB test, compared with the evaluation using the composite criteria such as close contact with active tuberculosis infection and/or suspicious fibrotic/calcific lesions on chest X-Ray without active tuberculosis infection. There was a moderate correlation between BCG scar number and TST ($p < 0.001$, $r = 0.495$), T-SPOT.TB test and QuantiFERON-TB Gold test ($p = 0.007$, $r = 0.406$),

T-SPOT.TB test and composite criteria (p= 0.024, r= 0.343). The specificity of QuantiFERON-TB Gold test was 85.7%, and sensitivity was 73.9% for all patients with rheumatic disease. It was 73.5% and 66.7% for T-SPOT.TB test, respectively. The specificity of TST was 60.3% and sensitivity was 47.8% for TST.

Conclusions: IGRA tests are not affected prior vaccination and useful for detecting latent tuberculosis infection in patients treated with corticosteroid due to lack of correlation between test negativity and corticosteroid therapy. Also, they are useful tests for diagnosis of latent tuberculosis infection as an alternative to TST due to their specificity and sensitivity.

Publication Type

Journal Article.

Year of Publication

2018

<79>

Unique Identifier

30185135

Title

Bone tumors developed in patients with juvenile inflammatory arthritis after anti-TNFalpha therapy.

Source

Immunotherapy. 10(12):1033-1039, 2018 09.

VI 1

Status

MEDLINE

Authors

Chi H; Teng J; Yang C; Su Y.

Authors Full Name

Chi, Huihui; Teng, Jialin; Yang, Chengde; Su, Yutong.

Institution

Chi, Huihui. Department of Rheumatology & Immunology, Ruijin Hospital, Shanghai Jiao Tong University School of Medicine, Number 197 Ruijin Second Road, Shanghai 200025, China.

Teng, Jialin. Department of Rheumatology & Immunology, Ruijin Hospital, Shanghai Jiao Tong University School of Medicine, Number 197 Ruijin Second Road, Shanghai 200025, China.
Yang, Chengde. Department of Rheumatology & Immunology, Ruijin Hospital, Shanghai Jiao Tong University School of Medicine, Number 197 Ruijin Second Road, Shanghai 200025, China.
Su, Yutong. Department of Rheumatology & Immunology, Ruijin Hospital, Shanghai Jiao Tong University School of Medicine, Number 197 Ruijin Second Road, Shanghai 200025, China.
Abstract
Administration of anti-TNFalpha agents has become a mainstay in the treatment of chronic inflammatory arthritis such as rheumatoid arthritis (RA) and spondyloarthritis. Adverse events, including infections and allergic reactions, have been reported. Malignancies are rare but potentially life threatening. The existence of bone tumor in those patients is very rare, only five cases of bone tumors were mentioned in juvenile idiopathic arthritis (JIA) in the literature. We describe three patients in whom bone neoplasms developed after years of anti-TNFalpha therapy for JIA or juvenile ankylosing spondylitis (JAS). One patient developed chondroblastoma, and the other two were diagnosed with osteosarcoma. Rheumatologists should increase their awareness of bone neoplasia in JIA or juvenile ankylosing spondylitis patients after anti-TNFalpha treatment.
Publication Type
Case Reports. Journal Article. Research Support, Non-U.S. Gov't.
Year of Publication
2018

<80>
Unique Identifier
30176211
Title
[De-sensitization to allopurinol in a patient with tophi gout]. [Spanish]
Source
Revista Alergia Mexico. 65(3):316-320, 2018 Jul-Sep.
VI 1
Status
MEDLINE
Authors
Lopez-Rocha EG; Hernandez-Montoya G; Rodriguez-Pesina AH; Rodriguez-Mireles KA.

Authors Full Name
Lopez-Rocha, Eunice Giselle; Hernandez-Montoya, Gerardo; Rodriguez-Pesina, Alberto Hernan; Rodriguez-Mireles, Karen Alicia.
Institution
Lopez-Rocha, Eunice Giselle. Alergia e Inmunologia Clinica, practica privada, San Luis Potosi, Mexico. aeri_05@hotmail.com.
Abstract
BACKGROUND: Allopurinol is a xanthine oxidase inhibitor used in the treatment of patients with gout. Approximately 2% of patients are affected by adverse reactions to this drug. Severity ranges from mild rashes to severe reactions in up to 0.4% of cases. De-sensitization is carried out by administering increasing doses of the drug.

CASE REPORT: Thirty-year old man diagnosed with hypercholesterolemia and hypertriglyceridemia treated with bezafibrate and pravastatin, systemic arterial hypertension treated with losartan and a 10-year history of hyperuricemia with gout. Tophi were found in metacarpophalangeal joints and elbows. Treatment was started with allopurinol 300 mg/day. Two weeks later, he experienced facial erythema with itching and maculopapular lesions on the malar region 1 hour after the medication was ingested. An outpatient drug de-sensitization protocol was initiated, starting with 5 mg, and with gradual dose increases every 4 to 5 days for 59 days until the desired maintenance dose (300 mg) was reached.

CONCLUSIONS: Experience shows that de-sensitization to allopurinol is a safe alternative when there is hypersensitivity and treatment with this drug is required.
Other Abstract
Publisher: Antecedentes: El alopurinol es un inhibidor de la xantinooxidasa usado en el tratamiento de pacientes con gota. Aproximadamente 2 % de los pacientes son afectados por reacciones adversas a dicho farmaco. La severidad varia de erupciones cutaneas leves a reacciones graves hasta en 0.4 % de los casos. La desensibilizacion se lleva a cabo incrementando paulatinamente la dosis del farmaco. Reporte de caso: Hombre de 30 anos con diagnostico de hipercolesterolemia e hipertrigliceridemia tratadas con bezafibrato y pravastatina, hipertension arterial sistemica tratada con losartan e hiperuricemia con gota de 10 anos de diagnostico. Los tofos se encontraban en articulaciones metacarpofalangicas y codos. Se inicio tratamiento con 300 mg diarios de alopurinol, a las dos semanas el paciente presento eritema facial con prurito y lesiones maculopapulares en region malar una hora despues de la ingesta del medicamento. Se inicio protocolo ambulatorio de desensibilizacion a dicho farmaco comenzando con 5 mg, con incremento gradual de la dosis cada cuatro o cinco dias por 59 dias, hasta llegar a la dosis de mantenimiento deseada (300 mg). Conclusiones: La experiencia muestra que la

desensibilizacion a alopurinol es una alternativa segura cuando existe hipersensibilidad y necesidad de tratamiento con este farmaco.; Language: Spanish

Publication Type

Case Reports. Journal Article.

Year of Publication

2018

<81>

Unique Identifier

30171203

Title

Rheumatic immune-related adverse events from cancer immunotherapy. [Review]

Source

Nature Reviews Rheumatology. 14(10):569-579, 2018 10.

VI 1

Status

MEDLINE

Authors

Calabrese LH; Calabrese C; Cappelli LC.

Authors Full Name

Calabrese, Leonard H; Calabrese, Cassandra; Cappelli, Laura C.

Institution

Calabrese, Leonard H. Department of Rheumatic and Immunologic Diseases, Cleveland Clinic Lerner College of Medicine of Case Western Reserve University, Cleveland Clinic Foundation, Cleveland, OH, USA. calabrl@ccf.org.

Calabrese, Cassandra. Department of Rheumatic and Immunologic Diseases, Cleveland Clinic Lerner College of Medicine of Case Western Reserve University, Cleveland Clinic Foundation, Cleveland, OH, USA.

Cappelli, Laura C. Division of Rheumatology, Johns Hopkins School of Medicine, Baltimore, MD, USA.

Abstract

Immunotherapy has revolutionized the treatment of cancer, but a rapid rise in the use of the family of therapeutic agents known as checkpoint inhibitors (CPIs) is associated with a new group

of immune-related adverse events (irAEs) in almost any organ system. Among these irAEs, rheumatic complications are common and seem to have features that are distinct from irAEs in other organ systems, including a highly variable time of clinical onset and the capacity to persist, possibly indefinitely, even after cessation of CPI therapy. In this Review, mechanisms of action of CPIs and how they might cause rheumatic irAEs are described. Also covered are epidemiology and clinical descriptions of rheumatic irAEs, plus guiding principles for managing irAEs. Finally, we outline future directions that must be taken in response to a series of unanswered questions and unmet needs that now confront rheumatologists who are, or will be, engaged in this new area of rheumatology.

Publication Type

Journal Article. Review.

Year of Publication

2018

<82>

Unique Identifier

30148175

Title

Influence of Biological Therapeutics, Cytokines, and Disease Activity on Depression in Rheumatoid Arthritis.

Source

Journal of Immunological Research. 2018:5954897, 2018.

VI 1

Status

MEDLINE

Authors

Figueiredo-Braga M; Cornaby C; Cortez A; Bernardes M; Terroso G; Figueiredo M; Mesquita CDS; Costa L; Poole BD.

Author NameID

Figueiredo-Braga, Margarida; ORCID: https://orcid.org/0000-0003-2374-4371

Bernardes, Miguel; ORCID: https://orcid.org/0000-0002-1849-5465

Poole, Brian D; ORCID: https://orcid.org/0000-0002-3205-640X

Authors Full Name

Figueiredo-Braga, Margarida; Cornaby, Caleb; Cortez, Alice; Bernardes, Miguel; Terroso, Georgina; Figueiredo, Marta; Mesquita, Cristina Dos Santos; Costa, Lucia; Poole, Brian D.
Institution
Figueiredo-Braga, Margarida. Medical Psychology Unit, Department of Clinical Neurosciences and Mental Health, Faculty of Medicine, University of Porto, Porto, Portugal.
Figueiredo-Braga, Margarida. I3S Instituto de Investigacao e Inovacao em Saude, Porto, Portugal.
Cornaby, Caleb. Department of Microbiology and Molecular Biology, Brigham Young University, Provo, UT, USA.
Cortez, Alice. Laboratorio Nobre, Faculty of Medicine, University of Porto, Porto, Portugal.
Bernardes, Miguel. Rheumatology Department, Hospital of Sao Joao EPE, Porto, Portugal.
Terroso, Georgina. Rheumatology Department, Hospital of Sao Joao EPE, Porto, Portugal.
Figueiredo, Marta. Medical Psychology Unit, Department of Clinical Neurosciences and Mental Health, Faculty of Medicine, University of Porto, Porto, Portugal.
Mesquita, Cristina Dos Santos. Departamento de Psicologia Aplicada (DPA), Universidade do Minho, Braga, Minho, Portugal.
Costa, Lucia. Rheumatology Department, Hospital of Sao Joao EPE, Porto, Portugal.
Poole, Brian D. Department of Microbiology and Molecular Biology, Brigham Young University, Provo, UT, USA.
Abstract
Purpose: Rheumatoid arthritis (RA) is an often debilitating autoinflammatory disease. Patients with rheumatoid arthritis are often troubled by co-occurring depression or other psychological manifestations. RA patients have a variety of treatment options available, including biologicals that inhibit cytokines or immune cells. If these cytokines influence the psychological symptoms, then the use of cytokine inhibitors should modulate these symptoms.

Methods: A cohort of 209 individuals was recruited. This group included 82 RA patients, 22 healthy subjects, 32 depressed control subjects, and 73 subjects with systemic lupus erythematosus. Of the RA patients, 51% were on a biological therapeutic. ELISA was used to measure cytokine levels. A variety of psychological assessments were used to evaluate depression, anxiety, sleep, fatigue, and relationship status. Clinical values were obtained from medical records.

Results: IL-10 concentration was associated with depressive symptoms in the RA patients, healthy controls, and the lupus patients. In the patients with primary depression, depressive symptoms were associated with IL-6 and TNF-alpha. In RA patients, Tocilizumab use was associated with decreased depressive symptoms. 14 RA patients who were not using biologicals

began using them by a one-month follow-up. In these patients, there was no significant change to any value except for fatigue.

Conclusions: A variety of both biological and social factors influences depressive symptoms in RA. IL-10 and IL-6 are likely to be involved, since IL-10 concentration was associated with depression and Tocilizumab decreased depressive symptoms in the RA patients. The roles of these cytokines are different in RA and lupus, as high IL-10 in RA is associated with increased depressive symptoms, but high IL-10 in the lupus patients is associated with decreased depression. IL-6 was also associated with depressive symptoms in the patients with primary depression. These results strongly indicate that disease activity, including cytokine levels, has a strong impact on depressive symptoms.

Publication Type

Journal Article.

Year of Publication

2018

<83>

Unique Identifier

30135988

Title

[Sexuality in adolescents with rheumatic diseases : Contraception, HPV vaccination and pregnancy]. [Review] [German]

Source

Zeitschrift fur Rheumatologie. 77(8):667-676, 2018 Oct.

VI 1

Status

MEDLINE

Authors

Fischer-Betz R; Haase I; Oommen PT.

Authors Full Name

Fischer-Betz, R; Haase, I; Oommen, P T.

Institution

Fischer-Betz, R. Poliklinik fur Rheumatologie und Hiller Forschungszentrum, Universitatsklinikum Dusseldorf, Moorenstr. 5, 40225, Dusseldorf, Deutschland. rebecca.fischer@med.uni-duesseldorf.de.
Haase, I. Poliklinik fur Rheumatologie und Hiller Forschungszentrum, Universitatsklinikum Dusseldorf, Moorenstr. 5, 40225, Dusseldorf, Deutschland.
Oommen, P T. Klinik fur Kinder-Onkologie, Hamatologie und Klinische Immunologie, Universitatsklinikum Dusseldorf, Dusseldorf, Deutschland.
Abstract
Young patients and adolescents with chronic rheumatic diseases have the same desires, fears and needs in terms of sexuality and pregnancy as their healthy peers. In most cases adolescents are already sexually active before transition from pediatric to adult rheumatological care takes place. Pregnancies in women with rheumatic diseases are associated with increased maternal and fetal risks, especially when they occur unplanned in the course of active disease or under teratogenic drugs. Safe contraception is therefore crucial in preventing unwanted pregnancies. The choice of contraception should anticipate the safety of the method of contraception as well as age-dependent practicability. A strategy of "double protection" through the use of condoms for contraception and prevention of sexually transmitted diseases combined with another safe contraception method should be recommended. Women with rheumatic diseases are more susceptible to acquire persisting human papilloma virus (HPV) infections and the subsequent progression to cervical cancer. In women with rheumatic diseases HPV vaccination induces high seroconversion rates, is safe and does not seem to induce disease activity. The care of adolescent women with rheumatic diseases before, during and after medical transition needs to encompass an open, early and continuous counselling regarding these topics in order to retain the individual health-related quality of life and to adapt this care to age-specific needs.
Publication Type
Journal Article. Review.
Year of Publication
2018

<84>
Unique Identifier
30103042
Title

Rheumatic immune-related adverse events in patients on anti-PD-1 inhibitors: Fasciitis with myositis syndrome as a new complication of immunotherapy. [Review]

Source

Autoimmunity Reviews. 17(10):1040-1045, 2018 Oct.

VI 1

Status

MEDLINE

Authors

Narvaez J; Juarez-Lopez P; LLuch J; Narvaez JA; Palmero R; Garcia Del Muro X; Nolla JM; Domingo-Domenech E.

Authors Full Name

Narvaez, J; Juarez-Lopez, P; LLuch, J; Narvaez, J A; Palmero, R; Garcia Del Muro, X; Nolla, J M; Domingo-Domenech, E.

Institution

Narvaez, J. Department of Rheumatology, Hospital Universitario de Bellvitge-IDIBELL, Barcelona, Spain. Electronic address: fjnarvaez@bellvitgehospital.cat.

Juarez-Lopez, P. Department of Rheumatology, Hospital Universitario de Bellvitge-IDIBELL, Barcelona, Spain.

LLuch, J. Department of Rheumatology, Hospital Universitario de Bellvitge-IDIBELL, Barcelona, Spain.

Narvaez, J A. Department of Radiology, Hospital Universitario de Bellvitge-IDIBELL, Barcelona, Spain.

Palmero, R. Department of Medical Oncology, Institut Catala d'Oncologia, Hospital Duran i Reynals, Barcelona, Spain.

Garcia Del Muro, X. Department of Medical Oncology, Institut Catala d'Oncologia, Hospital Duran i Reynals, Barcelona, Spain.

Nolla, J M. Department of Rheumatology, Hospital Universitario de Bellvitge-IDIBELL, Barcelona, Spain.

Domingo-Domenech, E. Department of Hematology, Institut Catala d'Oncologia, Hospital Duran i Reynals, Barcelona, Spain.

Abstract

OBJECTIVE: To evaluate the prevalence and type of rheumatic immune-related adverse events (IRAEs) in patients receiving programmed cell death protein-1 (PD-1) inhibitors.

METHODS: This is a single-center prospective observational study, including all cancer patients receiving PD-1 inhibitors between January 2016 and January 2018.

RESULTS: During the period analyzed, we evaluated a total of 11 patients. No patient had pre-existing rheumatic or autoimmune disease. In this period, a total of 220 patients were treated with PD1 inhibitors in our center; therefore, the estimated minimum prevalence of rheumatic IRAEs related to these therapies in our population was 5%. The rheumatic IRAEs evaluated included 5 cases of oligo- or polyarthritis, 1 with a polymialgia rheumatica-type syndrome, 2 cases of immunotherapy-induced sicca syndrome, 2 patients who presented symptomatic inflammatory myositis with fasciitis in lower extremities, and 1 patient with a paraneoplastic acral vascular syndrome. The median time to IRAE after anti-PD1 exposure was 8weeks (range: 2-24). In 5 patients, immunotherapy was discontinued (due to the adverse effect in three and cancer progression in two). In general terms the symptoms resolved completely with symptomatic treatment. Disease-modifying antirheumatic drugs were needed for 2 patients.

CONCLUSION: Rheumatic IRAEs should be kept in mind during the follow-up and evaluation of patients treated with PD-1 inhibitors. The concomitant development of symptomatic inflammatory myositis with fasciitis in lower extremities appears to be a new adverse effect of anti-PD-1 immunotherapy. Additional studies are needed to determine how to adequately control and manage these complications. Copyright © 2018 Elsevier B.V. All rights reserved.

Publication Type

Journal Article. Review.

Year of Publication

2018

<85>

Unique Identifier

30036638

Title

Anti-polysaccharide and anti-diphtheria protective antibodies after 13-valent pneumococcal conjugate vaccination in rheumatoid arthritis patients under immunosuppressive therapy.

Source

Clinical Immunology. 195:18-27, 2018 10.

VI 1

Status

MEDLINE

Authors
Caporuscio S; Ieraci R; Valesini G; Teloni R; Mariotti S; Spinelli FR; Ferlito C; Salemi S; Picchianti Diamanti A; Meneguzzi G; Markovic M; Sgrulletti M; von Hunolstein C; Ralli L; Pinto A; Salerno G; Canzoni M; Sorgi ML; Lagana B; Di Rosa R; Nisini R; D'Amelio R.
Authors Full Name
Caporuscio, Sara; Ieraci, Roberto; Valesini, Guido; Teloni, Raffaela; Mariotti, Sabrina; Spinelli, Francesca Romana; Ferlito, Claudia; Salemi, Simonetta; Picchianti Diamanti, Andrea; Meneguzzi, Giorgia; Markovic, Milica; Sgrulletti, Mayla; von Hunolstein, Christina; Ralli, Luisa; Pinto, Antonietta; Salerno, Gerardo; Canzoni, Marco; Sorgi, Maria Laura; Lagana, Bruno; Di Rosa, Roberta; Nisini, Roberto; D'Amelio, Raffaele.
Institution
Caporuscio, Sara. Sapienza Universita di Roma, Azienda Ospedaliera Universitaria Sant'Andrea, Roma, Italy.
Ieraci, Roberto. UOC Vaccinazioni Internazionali, ASL RM1, Roma, Italy.
Valesini, Guido. Dipartimento di Medicina Interna e Specialita Mediche, Reumatologia, Universita La Sapienza, Roma, Italy.
Teloni, Raffaela. Dipartimento Malattie Infettive, Istituto Superiore di Sanita, Roma, Italy.
Mariotti, Sabrina. Dipartimento Malattie Infettive, Istituto Superiore di Sanita, Roma, Italy.
Spinelli, Francesca Romana. Dipartimento di Medicina Interna e Specialita Mediche, Reumatologia, Universita La Sapienza, Roma, Italy.
Ferlito, Claudia. Sapienza Universita di Roma, Azienda Ospedaliera Universitaria Sant'Andrea, Roma, Italy.
Salemi, Simonetta. Sapienza Universita di Roma, Azienda Ospedaliera Universitaria Sant'Andrea, Roma, Italy.
Picchianti Diamanti, Andrea. Sapienza Universita di Roma, Azienda Ospedaliera Universitaria Sant'Andrea, Roma, Italy.
Meneguzzi, Giorgia. Sapienza Universita di Roma, Azienda Ospedaliera Universitaria Sant'Andrea, Roma, Italy.
Markovic, Milica. Sapienza Universita di Roma, Azienda Ospedaliera Universitaria Sant'Andrea, Roma, Italy.
Sgrulletti, Mayla. Sapienza Universita di Roma, Azienda Ospedaliera Universitaria Sant'Andrea, Roma, Italy.
von Hunolstein, Christina. Centro Nazionale per il Controllo e la Valutazione dei Farmaci, Istituto Superiore di Sanita, Roma, Italy.
Ralli, Luisa. Centro Nazionale per il Controllo e la Valutazione dei Farmaci, Istituto Superiore di Sanita, Roma, Italy.

Pinto, Antonietta. Centro Nazionale per il Controllo e la Valutazione dei Farmaci, Istituto Superiore di Sanita, Roma, Italy.
Salerno, Gerardo. Sapienza Universita di Roma, Azienda Ospedaliera Universitaria Sant'Andrea, Roma, Italy.
Canzoni, Marco. Servizio di Reumatologia ASL RM1, Roma, Italy.
Sorgi, Maria Laura. Sapienza Universita di Roma, Azienda Ospedaliera Universitaria Sant'Andrea, Roma, Italy.
Lagana, Bruno. Sapienza Universita di Roma, Azienda Ospedaliera Universitaria Sant'Andrea, Roma, Italy.
Di Rosa, Roberta. Sapienza Universita di Roma, Azienda Ospedaliera Universitaria Sant'Andrea, Roma, Italy.
Nisini, Roberto. Dipartimento Malattie Infettive, Istituto Superiore di Sanita, Roma, Italy. Electronic address: roberto.nisini@iss.it.
D'Amelio, Raffaele. Sapienza Universita di Roma, Azienda Ospedaliera Universitaria Sant'Andrea, Roma, Italy.
Abstract
Immunogenicity of 13-valent pneumococcal polysaccharide (PnPS) conjugate vaccine (PCV13) was evaluated in 38 rheumatoid arthritis patients under immunosuppressive treatment and 20 healthy controls (HC). Antibodies to all PnPS and diphtheria-toxin analogue conjugate protein were measured pre- (T0), 1 (T1), 6 (T2), 12 (T3) months post-immunization. Patients and HC had similar response to individual PnPS. Mean antibody levels to all PnPS but one doubled at T1 compared with T0, with T3 persistence for only 8-7/13 PnPS. Baseline antibody levels was inversely associated with the rate of responders at T1 (T1/T0>=2) to 11/13 PnPS. Few subjects reached protective IgG levels against some serotypes frequently isolated in Italian patients with invasive pneumococcal disease. Antibody response was not influenced by therapy, except the one to PS7F, which was reduced by tumor necrosis factor-alpha-inhibitors. Vaccination increased also anti-diphtheria IgG. Despite this study substantially confirmed the PCV13 immunogenicity in immunocompromised patients, it also revealed some limitations. Copyright © 2018 Elsevier Inc. All rights reserved.
Publication Type
Journal Article. Research Support, Non-U.S. Gov't.
Year of Publication
2018

<86>

Unique Identifier

30031500

Title

Complications of Treatments for Pediatric Rheumatic Diseases. [Review]

Source

Pediatric Clinics of North America. 65(4):827-854, 2018 08.

VI 1

Status

MEDLINE

Authors

Higgins GC.

Authors Full Name

Higgins, Gloria C.

Institution

Higgins, Gloria C. Pediatric Rheumatology, Nationwide Children's Hospital, 700 Children's Drive, Columbus, OH 43205, USA. Electronic address: gloria.higgins@nationwidechildrens.org.

Abstract

Medications to treat children with rheumatic disease include disease-modifying antirheumatic drugs, glucocorticosteroids, and biologic response modifiers that target mediators and cells involved in autoimmunity and inflammation. Although usually well-tolerated, such medications have many possible side effects, of which primary care and emergency providers should be aware. Both disease and immunosuppression contribute to susceptibility to unusual and opportunistic infections, in addition to usual childhood infections for which these children should receive all applicable nonlive vaccines. Close coordination between the rheumatologist and other medical care providers is essential, because medication side effects, infections, and disease flares are difficult to distinguish, and may occur together. Copyright © 2018 Elsevier Inc. All rights reserved.

Publication Type

Journal Article. Review.

Year of Publication

2018

<87>

Unique Identifier

30029059

Title

Myeloid-derived suppressor cells exacerbate Sjogren's syndrome by inhibiting Th2 immune responses.

Source

Molecular Immunology. 101:251-258, 2018 09.

VI 1

Status

MEDLINE

Authors

Qi J; Li D; Shi G; Zhang X; Pan Y; Dou H; Yao G; Hou Y.

Authors Full Name

Qi, Jingjing; Li, Dan; Shi, Guoping; Zhang, Xuefang; Pan, Yuchen; Dou, Huan; Yao, Genhong; Hou, Yayi.

Institution

Qi, Jingjing. The State Key Laboratory of Pharmaceutical Biotechnology, Division of Immunology, Medical School, Nanjing University, Nanjing, PR China.

Li, Dan. The State Key Laboratory of Pharmaceutical Biotechnology, Division of Immunology, Medical School, Nanjing University, Nanjing, PR China.

Shi, Guoping. The State Key Laboratory of Pharmaceutical Biotechnology, Division of Immunology, Medical School, Nanjing University, Nanjing, PR China.

Zhang, Xuefang. The State Key Laboratory of Pharmaceutical Biotechnology, Division of Immunology, Medical School, Nanjing University, Nanjing, PR China.

Pan, Yuchen. The State Key Laboratory of Pharmaceutical Biotechnology, Division of Immunology, Medical School, Nanjing University, Nanjing, PR China.

Dou, Huan. The State Key Laboratory of Pharmaceutical Biotechnology, Division of Immunology, Medical School, Nanjing University, Nanjing, PR China; Jiangsu Key Laboratory of Molecular Medicine, Nanjing, PR China.

Yao, Genhong. The State Key Laboratory of Pharmaceutical Biotechnology, Division of Immunology, Medical School, Nanjing University, Nanjing, PR China; Department of Rheumatology and Immunology, The Affiliated Drum Tower Hospital of Nanjing University Medical School, Nanjing, PR China. Electronic address: yaogenhong@nju.edu.cn.

Hou, Yayi. The State Key Laboratory of Pharmaceutical Biotechnology, Division of Immunology, Medical School, Nanjing University, Nanjing, PR China; Jiangsu Key Laboratory of Molecular Medicine, Nanjing, PR China. Electronic address: yayihou@nju.edu.cn.
Abstract
Myeloid-derived suppressor cells (MDSCs) can regulate various aspects of immune responses based on their potent immune-suppressive activity. Studies reported that MDSCs participated in many autoimmune diseases. However, the role of MDSCs in Sjogren's syndrome (SS) is unknown. In this study, we determined the frequencies and function of MDSCs in non-obese diabetic (NOD) mice and SS patients. The NOD mice were adoptively transferred with MDSCs or treated with anti-Gr1 antibody. Results showed that peripheral MDSCs increased significantly with the development of SS-like syndrome in NOD mice and the percentage of MDSCs was higher in SS patients than healthy controls. The SS-like syndrome aggravated after transfer of MDSCs in NOD mice. The deletion of MDSCs in NOD mice alleviated SS-like syndrome. Mechanistically, MDSCs down-regulated the percentages of Th2 cells in NOD mice and SS patients. In summary, our findings suggested that MDSCs exacerbated Sjogren's syndrome by inhibiting Th2 cells. Copyright © 2018 Elsevier Ltd. All rights reserved.
Publication Type
Journal Article. Research Support, Non-U.S. Gov't.
Year of Publication
2018

<88>
Unique Identifier
30017556
Title
Successful slow tocilizumab desensitization in a patient with adult onset Still disease.
Source
Biologicals. 55:17-18, 2018 Sep.
VI 1
Status
MEDLINE
Authors
Erdogan T; Yasar Bilge NS; Kasifoglu T.

Authors Full Name
  Erdogan, Tuba; Yasar Bilge, Nazife Sule; Kasifoglu, Timucin.
Institution
  Erdogan, Tuba. Eskisehir Osmangazi University, Medical Faculty, Department of Chest Disease, Division of Immunology and Allergy, Turkey.
  Yasar Bilge, Nazife Sule. Eskisehir Osmangazi University, Medical Faculty, Department of Internal Medicine, Division of Rheumatology, Turkey. Electronic address: suleyasar@yahoo.com.
  Kasifoglu, Timucin. Eskisehir Osmangazi University, Medical Faculty, Department of Internal Medicine, Division of Rheumatology, Turkey.
Publication Type
  Case Reports. Letter.
Year of Publication
  2018

<89>
Unique Identifier
  30001173
Title
  Treponema denticola enolase contributes to the production of antibodies against ENO1 but not to the progression of periodontitis.
Source
  Virulence. 9(1):1263-1272, 2018.
VI 1
Status
  MEDLINE
Authors
  Lee A; Kim YC; Baek K; Alam J; Choi YS; Rheu Y; Shin YJ; Kim S; Kim HD; Song YW; Choi Y.
Author NameID
  Song, Yeong W; ORCID: https://orcid.org/0000-0002-5384-3437
Authors Full Name
  Lee, Ahreum; Kim, Yong C; Baek, Keumjin; Alam, Jehan; Choi, Yun S; Rheu, Yaeeun; Shin, Yoo Jin; Kim, Sungtae; Kim, Hyun-Duck; Song, Yeong W; Choi, Youngnim.
Institution

Lee, Ahreum. a Department of Immunology and Molecular Microbiology, School of Dentistry and Dental Research Institute, Seoul National University, Seoul, Korea.

Kim, Yong C. a Department of Immunology and Molecular Microbiology, School of Dentistry and Dental Research Institute, Seoul National University, Seoul, Korea.

Baek, Keumjin. a Department of Immunology and Molecular Microbiology, School of Dentistry and Dental Research Institute, Seoul National University, Seoul, Korea.

Alam, Jehan. a Department of Immunology and Molecular Microbiology, School of Dentistry and Dental Research Institute, Seoul National University, Seoul, Korea.

Choi, Yun S. a Department of Immunology and Molecular Microbiology, School of Dentistry and Dental Research Institute, Seoul National University, Seoul, Korea.

Rheu, Yaeeun. b Department of Periodontology, School of Dentistry and Dental Research Institute, Seoul National University, Seoul, Korea.

Shin, Yoo Jin. c Department of Preventive and Social Dentistry, School of Dentistry, Seoul National University.

Kim, Sungtae. b Department of Periodontology, School of Dentistry and Dental Research Institute, Seoul National University, Seoul, Korea.

Kim, Hyun-Duck. c Department of Preventive and Social Dentistry, School of Dentistry, Seoul National University.

Song, Yeong W. d Department of Internal Medicine, Seoul National University Hospital.

Song, Yeong W. e Department of Molecular Medicine and Biopharmaceutical Sciences, Graduate School of Convergence Science and Technology and College of Medicine, Seoul National University, Seoul, Korea.

Choi, Youngnim. a Department of Immunology and Molecular Microbiology, School of Dentistry and Dental Research Institute, Seoul National University, Seoul, Korea.

Abstract

Autoantibodies against alpha-enolase (ENO1) are often detected in various infectious and autoimmune diseases. Anti-ENO1 antibody titers were reported to be associated with the severity of periodontitis in patients with rheumatoid arthritis. Because the enolase of the periodontal pathogen Treponema denticola (TdEno) has the highest homology with ENO1 among the enolases of human-associated bacteria, we hypothesized that anti-ENO1 autoantibodies produced during the immune response to TdEno may contribute to the progression of periodontitis and tested it in human and mouse systems. In human subjects with healthy periodontium or chronic periodontitis, a strong positive correlation between the levels of anti-TdEno and anti-ENO1 antibodies was observed. In addition, the purified anti-TdEno antibodies recognized ENO1 as well as TdEno in a dot blot, confirming the cross-reactivity between TdEno and ENO1. However, anti-ENO1 antibody titers were not associated with the severity of periodontitis. To further investigate the role of TdEno in the production of anti-ENO1 antibodies

and the progression of periodontitis, mice received an oral gavage of P. gingivalis alone, subcutaneous immunization with TdEno alone, or both P. gingivalis oral gavage and TdEno immunization. Immunization with TdEno induced not only anti-TdEno but also anti-mouse Eno1 (mEno1) antibodies and increased the expression of TNFalpha in the gingival tissues. However, alveolar bone loss was not increased by TdEno immunization. In conclusion, autoreactive anti-ENO1/mEno1 antibodies that are produced as byproducts during the antibody response to TdEno play a minimal role in the progression of periodontitis in the absence of rheumatoid arthritis.
Publication Type
Journal Article. Research Support, Non-U.S. Gov't.
Year of Publication
2018

<90>
Unique Identifier
29980390
Title
Predictors for influenza vaccine acceptance among patients with inflammatory rheumatic diseases.
Source
Vaccine. 36(32 Pt B):4875-4879, 2018 08 06.
VI 1
Status
MEDLINE
Authors
Harrison N; Poeppl W; Miksch M; Machold K; Kiener H; Aletaha D; Smolen JS; Forstner C; Burgmann H; Lagler H.
Authors Full Name
Harrison, Nicole; Poeppl, Wolfgang; Miksch, Manuel; Machold, Klaus; Kiener, Hans; Aletaha, Daniel; Smolen, Josef S; Forstner, Christina; Burgmann, Heinz; Lagler, Heimo.
Institution
Harrison, Nicole. Department of Medicine I, Division of Infectious Diseases and Tropical Medicine, Medical University of Vienna, Vienna, Austria.

Poeppl, Wolfgang. Department of Medicine I, Division of Infectious Diseases and Tropical Medicine, Medical University of Vienna, Vienna, Austria; Department of Dermatology and Tropical Medicine, Military Medical Cluster East, Austrian Armed Forces, Austria.

Miksch, Manuel. Department of Medicine I, Division of Infectious Diseases and Tropical Medicine, Medical University of Vienna, Vienna, Austria.

Machold, Klaus. Department of Medicine III, Division of Rheumatology, Medical University of Vienna, Vienna, Austria.

Kiener, Hans. Department of Medicine III, Division of Rheumatology, Medical University of Vienna, Vienna, Austria.

Aletaha, Daniel. Department of Medicine III, Division of Rheumatology, Medical University of Vienna, Vienna, Austria.

Smolen, Josef S. Department of Medicine III, Division of Rheumatology, Medical University of Vienna, Vienna, Austria.

Forstner, Christina. Department of Medicine I, Division of Infectious Diseases and Tropical Medicine, Medical University of Vienna, Vienna, Austria; Institute of Infectious Diseases and Infection Control, Jena University Hospital, Jena, Germany.

Burgmann, Heinz. Department of Medicine I, Division of Infectious Diseases and Tropical Medicine, Medical University of Vienna, Vienna, Austria.

Lagler, Heimo. Department of Medicine I, Division of Infectious Diseases and Tropical Medicine, Medical University of Vienna, Vienna, Austria. Electronic address: heimo.lagler@meduniwien.ac.at.

Abstract

BACKGROUND: Patients with inflammatory rheumatic diseases are at higher risk for influenza and current guidelines recommend vaccination for this group of patients. The aim of this study was to evaluate the vaccination coverage and predictors for influenza vaccination among patients with inflammatory rheumatic diseases.

METHODS: This survey was conducted at the outpatient rheumatology clinic at the Medical University of Vienna between July and October 2017. All patients diagnosed with an inflammatory rheumatic disease and receiving immunosuppressive therapy were asked to complete a questionnaire about their influenza vaccination status for 2016/17.

RESULTS: 490 patients with rheumatic diseases completed a questionnaire (33% male, mean age 55.3years). The influenza vaccination rate for the previous season was 25.3% (n=124/490). Predictors for a positive influenza vaccination status were higher age (Adjusted Odds Ratio 5.0, 95% Confidence Interval 2.4-10.4) and treatment with biological disease-modifying antirheumatic drugs (AOR 2.0, 95% CI 1.3-3.1). Patients who received a recommendation for influenza

vaccination by their general practitioner were significantly more likely to be vaccinated than those who did not (57% vs. 15%, AOR 6.6, 95% CI 4.1-10.8); even more so if they received a recommendation by their rheumatologist (62% vs. 19%, AOR 9.0, 95% CI 4.9-16.5). The main reasons for patients to decline influenza vaccination were fear of side effects (36%), concerns that vaccination might not be effective due to their immunosuppressed condition (38%) or that it might worsen the rheumatic disease (20%).

CONCLUSIONS: A moderate influenza vaccination rate of 25.3% was detected among patients with inflammatory rheumatic diseases. Recommendation of the influenza vaccine by a physician exerts the most effective impact on a positive vaccination status. Copyright © 2018 Elsevier Ltd. All rights reserved.

Publication Type

Journal Article.

Year of Publication

2018

<91>

Unique Identifier

29969365

Title

The mechanism of GM-CSF inhibition by human GM-CSF auto-antibodies suggests novel therapeutic opportunities.

Source

mAbs. 10(7):1018-1029, 2018 10.

VI 1

Status

MEDLINE

Authors

Dhagat U; Hercus TR; Broughton SE; Nero TL; Cheung Tung Shing KS; Barry EF; Thomson CA; Bryson S; Pai EF; McClure BJ; Schrader JW; Lopez AF; Parker MW.

Author NameID

Dhagat, Urmi; ORCID: https://orcid.org/0000-0002-5650-5767

Cheung Tung Shing, Karen S; ORCID: https://orcid.org/0000-0002-3817-0860

Parker, Michael W; ORCID: https://orcid.org/0000-0002-3101-1138
Authors Full Name
Dhagat, Urmi; Hercus, Timothy R; Broughton, Sophie E; Nero, Tracy L; Cheung Tung Shing, Karen S; Barry, Emma F; Thomson, Christy A; Bryson, Steve; Pai, Emil F; McClure, Barbara J; Schrader, John W; Lopez, Angel F; Parker, Michael W.
Institution
Dhagat, Urmi. a St. Vincent's Institute of Medical Research, Australian Cancer Research Foundation Rational Drug Discovery Centre, Fitzroy, Victoria, Australia.
Dhagat, Urmi. c Department of Biochemistry and Molecular Biology, Bio21 Molecular Science and Biotechnology Institute, University of Melbourne, Parkville, Victoria, Australia.
Hercus, Timothy R. b The Centre for Cancer Biology, SA Pathology and the University of South Australia, Adelaide, South Australia, Australia.
Broughton, Sophie E. a St. Vincent's Institute of Medical Research, Australian Cancer Research Foundation Rational Drug Discovery Centre, Fitzroy, Victoria, Australia.
Broughton, Sophie E. c Department of Biochemistry and Molecular Biology, Bio21 Molecular Science and Biotechnology Institute, University of Melbourne, Parkville, Victoria, Australia.
Nero, Tracy L. a St. Vincent's Institute of Medical Research, Australian Cancer Research Foundation Rational Drug Discovery Centre, Fitzroy, Victoria, Australia.
Nero, Tracy L. c Department of Biochemistry and Molecular Biology, Bio21 Molecular Science and Biotechnology Institute, University of Melbourne, Parkville, Victoria, Australia.
Cheung Tung Shing, Karen S. a St. Vincent's Institute of Medical Research, Australian Cancer Research Foundation Rational Drug Discovery Centre, Fitzroy, Victoria, Australia.
Cheung Tung Shing, Karen S. c Department of Biochemistry and Molecular Biology, Bio21 Molecular Science and Biotechnology Institute, University of Melbourne, Parkville, Victoria, Australia.
Barry, Emma F. b The Centre for Cancer Biology, SA Pathology and the University of South Australia, Adelaide, South Australia, Australia.
Thomson, Christy A. d The Biomedical Research Centre, University of British Columbia, Vancouver, British Columbia, Canada.
Bryson, Steve. e Princess Margaret Cancer Centre, University Health Network, University of Toronto, Toronto, Ontario, Canada.
Bryson, Steve. f Department of Biochemistry, University of Toronto, Toronto, Ontario, Canada.
Pai, Emil F. e Princess Margaret Cancer Centre, University Health Network, University of Toronto, Toronto, Ontario, Canada.
Pai, Emil F. f Department of Biochemistry, University of Toronto, Toronto, Ontario, Canada.
Pai, Emil F. g Department of Medical Biophysics, University of Toronto, Toronto, Ontario, Canada.

Pai, Emil F. h Department of Molecular Genetics, University of Toronto, Toronto, Ontario, Canada.
McClure, Barbara J. b The Centre for Cancer Biology, SA Pathology and the University of South Australia, Adelaide, South Australia, Australia.
Schrader, John W. d The Biomedical Research Centre, University of British Columbia, Vancouver, British Columbia, Canada.
Schrader, John W. g Department of Medical Biophysics, University of Toronto, Toronto, Ontario, Canada.
Lopez, Angel F. b The Centre for Cancer Biology, SA Pathology and the University of South Australia, Adelaide, South Australia, Australia.
Lopez, Angel F. i Department of Medicine, University of Adelaide, Adelaide, South Australia, Australia.
Parker, Michael W. a St. Vincent's Institute of Medical Research, Australian Cancer Research Foundation Rational Drug Discovery Centre, Fitzroy, Victoria, Australia.
Parker, Michael W. c Department of Biochemistry and Molecular Biology, Bio21 Molecular Science and Biotechnology Institute, University of Melbourne, Parkville, Victoria, Australia.
Abstract
Granulocyte-macrophage colony-stimulating factor (GM-CSF) is a hematopoietic growth factor that can stimulate a variety of cells, but its overexpression leads to excessive production and activation of granulocytes and macrophages with many pathogenic effects. This cytokine is a therapeutic target in inflammatory diseases, and several anti-GM-CSF antibodies have advanced to Phase 2 clinical trials in patients with such diseases, e.g., rheumatoid arthritis. GM-CSF is also an essential factor in preventing pulmonary alveolar proteinosis (PAP), a disease associated with GM-CSF malfunction arising most typically through the presence of GM-CSF neutralizing auto-antibodies. Understanding the mechanism of action for neutralizing antibodies that target GM-CSF is important for improving their specificity and affinity as therapeutics and, conversely, in devising strategies to reduce the effects of GM-CSF auto-antibodies in PAP. We have solved the crystal structures of human GM-CSF bound to antigen-binding fragments of two neutralizing antibodies, the human auto-antibody F1 and the mouse monoclonal antibody 4D4. Coordinates and structure factors of the crystal structures of the GM-CSF:F1 Fab and the GM-CSF:4D4 Fab complexes have been deposited in the RCSB Protein Data Bank under the accession numbers 6BFQ and 6BFS, respectively. The structures show that these antibodies bind to mutually exclusive epitopes on GM-CSF; however, both prevent the cytokine from interacting with its alpha receptor subunit and hence prevent receptor activation. Importantly, identification of the F1 epitope together with functional analyses highlighted modifications to GM-CSF that would abolish auto-antibody recognition whilst retaining GM-CSF function. These results provide a framework

for developing novel GM-CSF molecules for PAP treatment and for optimizing current anti-GM-CSF antibodies for use in treating inflammatory disorders.

Publication Type

Journal Article. Research Support, Non-U.S. Gov't.

Year of Publication

2018

<92>

Unique Identifier

29925278

Title

Sirukumab for the treatment of rheumatoid arthritis: update on sirukumab, 2018. [Review]

Source

Expert Review of Clinical Immunology. 14(7):539-547, 2018 07.

VI 1

Status

MEDLINE

Authors

Bartoli F; Bae S; Cometi L; Matucci Cerinic M; Furst DE.

Authors Full Name

Bartoli, Francesca; Bae, Sangmee; Cometi, Laura; Matucci Cerinic, Marco; Furst, Daniel E.

Institution

Bartoli, Francesca. a Department of Experimental and Clinical Medicine, University of Florence & Department of Geriatric Medicine, Division of Rheumatology, AOUC, Florence, Italy.

Bae, Sangmee. b Division of Rheumatology Fellow, Geffen School of Medicine at the University of California in Los Angeles, Los Angeles, California, USA.

Cometi, Laura. a Department of Experimental and Clinical Medicine, University of Florence & Department of Geriatric Medicine, Division of Rheumatology, AOUC, Florence, Italy.

Matucci Cerinic, Marco. a Department of Experimental and Clinical Medicine, University of Florence & Department of Geriatric Medicine, Division of Rheumatology, AOUC, Florence, Italy.

Furst, Daniel E. c Geffen School of Medicine at the University of California in Los Angeles (Emeritus), California, USA.

Furst, Daniel E. d Department of Experimental and Clinical Medicine, University of Florence, Florence, Italy.
Furst, Daniel E. e University of Washington, Seattle, Washington DC, USA.
Furst, Daniel E. f Pacific Arthritis, Los Angeles, California, USA.
Furst, Daniel E. g Seattle Rheumatology Associates, Seattle, Washington, DC, USA.
Abstract
INTRODUCTION: Interleukin-6 (IL-6) is well-known for its pro-inflammatory properties, has been proven to target a wide range of cells in the joint, and has been implicated in extra-articular and articular manifestations in rheumatoid arthritis (RA). Tocilizumab (TCZ) is now widely used in patients with active RA and a number of additional agents that target the IL-6 pathways are under development, including sirukumab (SRK). Areas covered: SRK is an IgG1kappa human anti-IL-6 monoclonal antibody which binds to IL-6 and prevents IL-6-mediated downstream effects. Initial trial results in phase-III studies in patients with RA seemed promising, showing improved results in patients with moderate-to-severe RA. Data derive from the phase-II study and the various SIRROUND studies (phase III). Expert commentary: The available data show that SRK50 mg every 4 weeks or 100 mg every 2 weeks will be effective in treating the RA population, with clinical improvements as early as week 2 and sustained over time. The adverse-event profile seems to be similar to TCZ, except for an increased mortality post open-label studies due to infections and cardiovascular events, our knowledge of which will be deepened with post-marketing surveillance and registry data.
Publication Type
Journal Article. Review.
Year of Publication
2018

<93>
Unique Identifier
29901743
Title
Predictors and temporal trend of flu vaccination in auto-immune rheumatic diseases in the UK: a nationwide prospective cohort study.
Source
Rheumatology. 57(10):1726-1734, 2018 10 01.

VI 1

Status

MEDLINE

Authors

Nakafero G; Grainge MJ; Myles PR; Mallen CD; Zhang W; Doherty M; Nguyen-Van-Tam JS; Abhishek A.

Authors Full Name

Nakafero, Georgina; Grainge, Matthew J; Myles, Puja R; Mallen, Christian D; Zhang, Weiya; Doherty, Michael; Nguyen-Van-Tam, Jonathan S; Abhishek, Abhishek.

Institution

Nakafero, Georgina. Academic Rheumatology, University of Nottingham, Nottingham, UK.

Grainge, Matthew J. Epidemiology and Public Health, University of Nottingham, Nottingham, UK.

Myles, Puja R. Epidemiology and Public Health, University of Nottingham, Nottingham, UK.

Mallen, Christian D. Primary Care Sciences, Keele University, Keele, UK.

Zhang, Weiya. Academic Rheumatology, University of Nottingham, Nottingham, UK.

Doherty, Michael. Academic Rheumatology, University of Nottingham, Nottingham, UK.

Nguyen-Van-Tam, Jonathan S. Epidemiology and Public Health, University of Nottingham, Nottingham, UK.

Abhishek, Abhishek. Academic Rheumatology, University of Nottingham, Nottingham, UK.

Abstract

Objectives: To examine temporal trend in uptake of seasonal influenza vaccine (SIV) in the UK and explore disease and demographic factors associated with vaccination.

Methods: From the Clinical Practice Research Datalink, 32 751 people with auto-immune rheumatic diseases prescribed DMARDs between 2006 and 2016 were identified. The proportion vaccinated between 1 September of one year and 31 March of the next year was calculated and stratified by age, other indications for vaccination, auto-immune rheumatic diseases type and number of DMARDs prescribed. Stata and Joinpoint regression programs were used.

Results: SIV uptake was high in those aged 65 years (82.3 and 80.7% in 2006-07 and 2015-16, respectively). It was significantly lower in other age groups, but improved over time with 51.9 and 61.9% in the 45-64 year age group, and 32.3 and 50.1% in the <45 year age group being vaccinated in 2006-07 and 2015-16, respectively. While 64.9% of the vaccinations in those 65 years old occurred by 3 November, in time to mount a protective immune response before the influenza activity becomes substantial in the UK, only 38.9% in the 45-64 year and 26.2% in the <45 year age group without any other reason for vaccination received SIV by this date. Women,

those with additional indications for vaccination, on multiple DMARDs and with SLE were more likely to be vaccinated.

Conclusion: SIV uptake is low in the under 65s, and the majority of them are not vaccinated in time. Additional effort is required to promote timely uptake of SIV in this population.

Publication Type

Journal Article. Research Support, Non-U.S. Gov't.

Year of Publication

2018

<94>

Unique Identifier

29879317

Title

Clinical effectiveness of influenza vaccination in patients with rheumatoid arthritis.

Source

International Journal of Rheumatic Diseases. 21(6):1246-1253, 2018 Jun.

VI 1

Status

MEDLINE

Authors

Chen CM; Chen HJ; Chen WS; Lin CC; Hsu CC; Hsu YH.

Author NameID

Hsu, Yueh-Han; ORCID: http://orcid.org/0000-0003-3619-468X

Authors Full Name

Chen, Chun-Ming; Chen, Hsuan-Ju; Chen, Wei-Shan; Lin, Che-Chen; Hsu, Chih-Cheng; Hsu, Yueh-Han.

Institution

Chen, Chun-Ming. Division of Allergy, Immunology, and Rheumatology, Department of Internal Medicine, Ditmanson Medical Foundation Chiayi Christian Hospital, Chiayi City, Taiwan.

Chen, Hsuan-Ju. Management Office for Health Data, China Medical University Hospital, Taichung, Taiwan.

Chen, Hsuan-Ju. College of Medicine, China Medical University, Taichung, Taiwan.

Chen, Wei-Shan. Management Office for Health Data, China Medical University Hospital, Taichung, Taiwan.

Chen, Wei-Shan. College of Medicine, China Medical University, Taichung, Taiwan.

Lin, Che-Chen. Healthcare Service Research Center, Taichung Veterans General Hospital, Taichung, Taiwan.

Hsu, Chih-Cheng. Institute of Population Health Sciences, National Health Research Institutes, Miaoli County, Taiwan.

Hsu, Chih-Cheng. Department of Family Medicine, Min-Sheng General Hospital, Taoyuan, Taiwan.

Hsu, Yueh-Han. Department of Medical Research, China Medical University Hospital, China Medical University, Taichung, Taiwan.

Hsu, Yueh-Han. Department of Internal Medicine, Ditmanson Medical Foundation Chia-Yi Christian Hospital, Chia-Yi City, Taiwan.

Hsu, Yueh-Han. Department of Nursing, Min-Hwei College of Health Care Management, Tainan City, Taiwan.

Abstract

OBJECTIVE: To determine the clinical effectiveness of influenza vaccination in patients with rheumatoid arthritis (RA).

METHODS: The present study was conducted by using the Taiwan National Health Insurance Research Database. In this retrospective nationwide study, we included 3748 RA patients who received influenza vaccinations in 2008, 2009 and 2010, and 3748 matched RA patients who did not receive influenza vaccinations. We followed the patients from 4 weeks after influenza vaccination to the end of the influenza season in each year. After adjustment for potential confounding factors, including disease-modifying anti-rheumatic drugs, we used the Cox proportional hazards regression model to analyze the clinical effectiveness of influenza vaccination.

RESULTS: The influenza vaccination rate in RA patients was 14.8% in 2008, 19.8% in 2009 and 9.50% in 2010. Receiving influenza vaccine was associated with reduced risk of hospitalization for septicemia, bacteremia or viremia (hazards ratio [HR] = 0.65, 95% CI = 0.45-0.94), and lower risk of mortality (HR = 0.62, 95% CI = 0.39-0.97). The effectiveness was particularly significant in elderly patients.

CONCLUSIONS: RA patients receiving influenza vaccine have significantly lower morbidity and mortality, particularly in elderly patients. Further studies are needed to explore effective policies to

increase the vaccination rate in elderly RA patients. Copyright © 2018 Asia Pacific League of Associations for Rheumatology and John Wiley & Sons Australia, Ltd.
Publication Type
Journal Article.
Year of Publication
2018

<95>
Unique Identifier
29790291
Title
The Eyes Have it: A Rheumatologist's View of Uveitis. [Review]
Source
Arthritis & Rheumatology. 70(10):1533-1543, 2018 10.
VI 1
Status
MEDLINE
Authors
Rosenbaum JT; Dick AD.
Authors Full Name
Rosenbaum, James T; Dick, Andrew D.
Institution
Rosenbaum, James T. Oregon Health & Science University and Legacy Devers Eye Institute, Portland, Oregon.
Dick, Andrew D. University College London, National Institute for Health Research Biomedical Research Centre at Moorfields Eye Hospital, London, UK, and University of Bristol, Bristol Eye Hospital, Bristol, UK.
Abstract
Uveitis is defined as intraocular inflammation. It is an extraarticular manifestation of many forms of joint disease, which include spondyloarthritis, juvenile idiopathic arthritis, and Behcet's disease. Rheumatologists may be asked to consult on the ophthalmologic care of patients with uveitis in order to identify an associated systemic illness. Diagnoses such as spondyloarthritis, sarcoidosis, and interstitial nephritis with uveitis are frequently overlooked by referring ophthalmologists.

Alternatively, rheumatologists may be asked to help manage the patient's immunosuppression, including biologic therapy, which can be required to treat a subset of patients with uveitis. This review is intended to provide rheumatologists with the necessary information to facilitate collaboration in the comanagement of patients with uveitis. Copyright © 2018, American College of Rheumatology.

Publication Type

Journal Article. Research Support, N.I.H., Extramural. Research Support, Non-U.S. Gov't. Review.

Year of Publication

2018

<96>

Unique Identifier

29772545

Title

Autoantibodies against muscarinic acetylcholine receptor M3 in Sjogren's syndrome and corresponding mouse models. [Review]

Source

Frontiers in Bioscience (Landmark Edition). 23:2053-2064, 2018 06 01.

VI 1

Status

MEDLINE

Authors

Yu X; Riemekasten G; Petersen F.

Authors Full Name

Yu, Xinhua; Riemekasten, Gabriela; Petersen, Frank.

Institution

Yu, Xinhua. Priority Area Asthma & Allergy, Research Center Borstel, 23845 Borstel, Germany, Airway Research Center North (ARCN), Member of the German Center for Lung Research (DZL), xinhuayu@fz-borstel.de.

Riemekasten, Gabriela. Department of Rheumatology, University of Lubeck, 23538, Lubeck, Germany.

Petersen, Frank. Priority Area Asthma and Allergy, Research Center Borstel, 23845 Borstel, Germany, Airway Research Center North (ARCN), Member of the German Center for Lung Research (DZL).
Abstract
Muscarinic acetylcholine receptor M3 (M3R) is a GPCR on exocrine gland cells involved in fluid secretion. In the last two decades, evidence has been accumulated arguing for a role of autoantibodies (aab) against M3R in the development of Sjogren's syndrome (SS). In this review, we provide an updated overview on this issue and critically discuss the relation between autoimmunity to M3R and SS pathogenesis. Clinical data as well as findings from experimental disease were summarized in categories addressing the presence of aab against M3R in SS patients, the function of anti-M3R aab, the association of aab against M3R with SS-related phenotypes, in vivo pathogenicity of transferred aab against M3R in mice, and mouse models induced via immunization with M3R. Based on these comprehensive data, we propose a hypothetic model for the role of aab against M3R in the pathogenesis of SS.
Publication Type
Journal Article. Research Support, Non-U.S. Gov't. Review.
Year of Publication
2018

<97>
Unique Identifier
29764963
Title
Reducing Missed Opportunities for Influenza Vaccination in Patients with Rheumatoid Arthritis: Evaluation of a Multisystem Intervention.
Source
Journal of Rheumatology. 45(9):1220-1228, 2018 08.
VI 1
Status
MEDLINE
Authors
Broderick R; Ventura I; Soroosh S; Franco L; Giles JT.
Authors Full Name

Broderick, Rachel; Ventura, Iazsmin; Soroosh, Sunoz; Franco, Lourdes; Giles, Jon T.
Institution
Broderick, Rachel. From the Division of Rheumatology, Columbia University, College of Physicians and Surgeons, New York, New York, USA.
Broderick, Rachel. R. Broderick, RS, Division of Rheumatology, Columbia University, College of Physicians and Surgeons; I. Ventura, MD, Division of Rheumatology, Columbia University, College of Physicians and Surgeons; S. Soroosh, RN, Division of Rheumatology, Columbia University, College of Physicians and Surgeons; L. Franco, BA, Division of Rheumatology, Columbia University, College of Physicians and Surgeons; J.T. Giles, MD, MPH, Division of Rheumatology, Columbia University, College of Physicians and Surgeons.
Ventura, Iazsmin. From the Division of Rheumatology, Columbia University, College of Physicians and Surgeons, New York, New York, USA.
Ventura, Iazsmin. R. Broderick, RS, Division of Rheumatology, Columbia University, College of Physicians and Surgeons; I. Ventura, MD, Division of Rheumatology, Columbia University, College of Physicians and Surgeons; S. Soroosh, RN, Division of Rheumatology, Columbia University, College of Physicians and Surgeons; L. Franco, BA, Division of Rheumatology, Columbia University, College of Physicians and Surgeons; J.T. Giles, MD, MPH, Division of Rheumatology, Columbia University, College of Physicians and Surgeons.
Soroosh, Sunoz. From the Division of Rheumatology, Columbia University, College of Physicians and Surgeons, New York, New York, USA.
Soroosh, Sunoz. R. Broderick, RS, Division of Rheumatology, Columbia University, College of Physicians and Surgeons; I. Ventura, MD, Division of Rheumatology, Columbia University, College of Physicians and Surgeons; S. Soroosh, RN, Division of Rheumatology, Columbia University, College of Physicians and Surgeons; L. Franco, BA, Division of Rheumatology, Columbia University, College of Physicians and Surgeons; J.T. Giles, MD, MPH, Division of Rheumatology, Columbia University, College of Physicians and Surgeons.
Franco, Lourdes. From the Division of Rheumatology, Columbia University, College of Physicians and Surgeons, New York, New York, USA.
Franco, Lourdes. R. Broderick, RS, Division of Rheumatology, Columbia University, College of Physicians and Surgeons; I. Ventura, MD, Division of Rheumatology, Columbia University, College of Physicians and Surgeons; S. Soroosh, RN, Division of Rheumatology, Columbia University, College of Physicians and Surgeons; L. Franco, BA, Division of Rheumatology, Columbia University, College of Physicians and Surgeons; J.T. Giles, MD, MPH, Division of Rheumatology, Columbia University, College of Physicians and Surgeons.
Giles, Jon T. From the Division of Rheumatology, Columbia University, College of Physicians and Surgeons, New York, New York, USA. jtg2122@columbia.edu.

Giles, Jon T. R. Broderick, RS, Division of Rheumatology, Columbia University, College of Physicians and Surgeons; I. Ventura, MD, Division of Rheumatology, Columbia University, College of Physicians and Surgeons; S. Soroosh, RN, Division of Rheumatology, Columbia University, College of Physicians and Surgeons; L. Franco, BA, Division of Rheumatology, Columbia University, College of Physicians and Surgeons; J.T. Giles, MD, MPH, Division of Rheumatology, Columbia University, College of Physicians and Surgeons.
jtg2122@columbia.edu.
Abstract

OBJECTIVE: To assess a multimodal intervention for reducing missed opportunities for outpatient influenza vaccination in individuals with rheumatoid arthritis (RA).

METHODS: Patients with RA were enrolled from a single center and each rheumatology outpatient visit was tracked for missed opportunities for influenza vaccination, defined as a visit in which an unvaccinated patient without contraindications remained unvaccinated or lacked documentation of vaccine recommendation in the electronic medical record (EMR). Providers then received a multimodal intervention consisting of an education session, EMR alerts, and weekly provider-specific e-mail reminders. Missed opportunities before and after the intervention were compared, and the determinants of missed opportunities were analyzed.

RESULTS: A total of 228 patients with RA were enrolled (904 preintervention visits) and 197 returned for at least 1 postintervention visit (721 postintervention visits). The preintervention frequency of any missed opportunities for influenza vaccination was 47%. This was reduced to 23% postintervention ($p < 0.001$). Among those vaccinated, the relative hazard for influenza vaccination post- versus preintervention period was 1.24 ($p = 0.038$). Younger age, less frequent office visits, higher erythrocyte sedimentation rate, and negative attitudes about vaccines were each independently associated with missed opportunities preintervention. Postintervention, these factors were no longer associated with missed opportunities; however, the intervention was not as effective in non-Hispanic black patients, non-English speakers, those residing outside of the New York City metropolitan area, and those reporting prior adverse reactions to vaccines.

CONCLUSION: Improved uptake of influenza vaccination in patients with RA is possible using a multimodal approach. Certain subgroups may need a more potent intervention for equivalent efficacy.
Publication Type
Journal Article. Research Support, Non-U.S. Gov't.
Year of Publication
2018

<98>

Unique Identifier

29763424

Title

Prevalence of primary Sjogren's syndrome in patients undergoing evaluation for pulmonary arterial hypertension.

Source

PLoS ONE [Electronic Resource]. 13(5):e0197297, 2018.

VI 1

Status

MEDLINE

Authors

Sato T; Hatano M; Iwasaki Y; Maki H; Saito A; Minatsuki S; Inaba T; Amiya E; Fujio K; Watanabe M; Yamamoto K; Komuro I.

Author NameID

Sato, Tatsuyuki; ORCID: https://orcid.org/0000-0002-9229-9016

Authors Full Name

Sato, Tatsuyuki; Hatano, Masaru; Iwasaki, Yukiko; Maki, Hisataka; Saito, Akihito; Minatsuki, Shun; Inaba, Toshiro; Amiya, Eisuke; Fujio, Keishi; Watanabe, Masafumi; Yamamoto, Kazuhiko; Komuro, Issei.

Institution

Sato, Tatsuyuki. Department of Cardiovascular Medicine, Graduate School of Medicine, The University of Tokyo, Tokyo, Japan.

Hatano, Masaru. Department of Therapeutic Strategy for Heart Failure, Graduate School of Medicine, The University of Tokyo, Tokyo, Japan.

Iwasaki, Yukiko. Department of Allergy and Rheumatology, Graduate School of Medicine, The University of Tokyo, Tokyo, Japan.

Maki, Hisataka. Department of Cardiovascular Medicine, Graduate School of Medicine, The University of Tokyo, Tokyo, Japan.

Saito, Akihito. Department of Cardiovascular Medicine, Graduate School of Medicine, The University of Tokyo, Tokyo, Japan.

Minatsuki, Shun. Department of Cardiovascular Medicine, Graduate School of Medicine, The University of Tokyo, Tokyo, Japan.
Inaba, Toshiro. Department of Cardiovascular Medicine, Graduate School of Medicine, The University of Tokyo, Tokyo, Japan.
Amiya, Eisuke. Department of Cardiovascular Medicine, Graduate School of Medicine, The University of Tokyo, Tokyo, Japan.
Fujio, Keishi. Department of Allergy and Rheumatology, Graduate School of Medicine, The University of Tokyo, Tokyo, Japan.
Watanabe, Masafumi. Department of Cardiovascular Medicine, Graduate School of Medicine, The University of Tokyo, Tokyo, Japan.
Yamamoto, Kazuhiko. Department of Allergy and Rheumatology, Graduate School of Medicine, The University of Tokyo, Tokyo, Japan.
Yamamoto, Kazuhiko. Laboratory for Autoimmune Diseases, RIKEN Center for Integrative Medical Sciences, Yokohama, Japan.
Komuro, Issei. Department of Cardiovascular Medicine, Graduate School of Medicine, The University of Tokyo, Tokyo, Japan.
Abstract
BACKGROUND: The prevalence of pulmonary arterial hypertension (PAH) in primary Sjogren's syndrome (SS) had been reported to be rare. However, recent studies using echocardiography as a screening method showed conflicting results, and the true prevalence is still unclear. Since diagnosing primary SS is difficult because of its heterogeneous nature, a number of patients with primary-SS-associated PAH may be misdiagnosed with idiopathic PAH, losing their chance to undergo immunosuppressive therapy. Therefore, we sought to elucidate the prevalence of primary SS among patients who initially present with PAH.

METHODS: From our prospective institutional PAH database, 40 consecutive patients without any obvious cause of PAH at the time of PAH diagnosis were identified. We retrospectively evaluated the prevalence of primary SS diagnosed during or after the initial assessment of PAH.

RESULTS: During the initial assessment, one patient was diagnosed with primary-SS-associated PAH. Among the 25 patients who were initially diagnosed with idiopathic PAH, five were diagnosed with primary SS during their course of the disease. Of the five patients, three had key signs suggesting primary SS and were probably underdiagnosed at the time of initial evaluation. The remaining two patients, who were finally diagnosed with primary SS, did not have any specific signs suggesting primary SS at the time of initial evaluation but showed positive conversion of their autoantibodies during the course of PAH.

CONCLUSION: The prevalence of primary-SS-associated PAH may be relatively high among patients who undergo initial evaluation for PAH. Furthermore, primary-SS-associated PAH may be underdiagnosed with routine evaluation for the primary cause of PAH. Clinicians should pay specific attention and carefully evaluate the possibility of primary SS in patients with PAH.

Publication Type

Journal Article.

Year of Publication

2018

<99>

Unique Identifier

29762203

Title

Preparing patients for biologic medications for dermatologic and rheumatic diseases.

Source

JAAPA. 31(6):23-28, 2018 Jun.

VI 1

Status

MEDLINE

Authors

Smith BJ; Nuccio BC; Graves KY; McMillan VM.

Authors Full Name

Smith, Benjamin J; Nuccio, Brigitta C; Graves, Katelyn Y; McMillan, Victor M.

Institution

Smith, Benjamin J. In the PA program at Florida State University in Tallahassee, Benjamin J. Smith is director of didactic education and Brigitta C. Nuccio and Katelyn Y. Graves are assistant professors. Victor M. McMillan practices at McIntosh Clinic in Thomasville, Ga. The authors have disclosed no potential conflicts of interest, financial or otherwise.

Abstract

Psoriasis, psoriatic arthritis, and rheumatoid arthritis are prevalent conditions that often require a team of primary care and specialist healthcare professionals for the most optimum patient outcomes. Primary care providers can facilitate referrals to dermatology and rheumatology specialists by obtaining the needed screening workup for patients who need treatment with

immunosuppressive therapies. This article reviews tuberculosis screening, hepatitis screening, and vaccinations to be administered before patients begin biologic medications.

Publication Type

Journal Article.

Year of Publication

2018

<100>

Unique Identifier

29750331

Title

Salivary-gland-protective regulatory T-cell dysfunction underlies female-specific sialadenitis in the non-obese diabetic mouse model of Sjogren syndrome.

Source

Immunology. 155(2):225-237, 2018 10.

VI 1

Status

MEDLINE

Authors

Barr JY; Wang X; Kreiger PA; Lieberman SM.

Author NameID

Lieberman, Scott M; ORCID: https://orcid.org/0000-0003-4082-7582

Authors Full Name

Barr, Jennifer Y; Wang, Xiaofang; Kreiger, Portia A; Lieberman, Scott M.

Institution

Barr, Jennifer Y. Stead Family Department of Pediatrics, Carver College of Medicine, University of Iowa, Iowa City, IA, USA.

Wang, Xiaofang. Stead Family Department of Pediatrics, Carver College of Medicine, University of Iowa, Iowa City, IA, USA.

Kreiger, Portia A. Department of Pathology and Laboratory Medicine, Perelman School of Medicine, University of Pennsylvania, Philadelphia, PA, USA.

Kreiger, Portia A. Division of Anatomic Pathology, Children's Hospital of Philadelphia, Philadelphia, PA, USA.

Lieberman, Scott M. Stead Family Department of Pediatrics, Carver College of Medicine, University of Iowa, Iowa City, IA, USA.
Lieberman, Scott M. Interdisciplinary Graduate Program in Immunology, University of Iowa, Iowa City, IA, USA.
Abstract
Immune cell-mediated destruction of salivary glands is a hallmark feature of Sjogren syndrome. Similar to the female predominance in humans, female non-obese diabetic (NOD) mice develop spontaneous salivary gland autoimmunity. However, in both humans and mice it is unclear what factors contribute to the initial immune infiltration of the salivary glands. Here, we used an adoptive transfer model of Sjogren syndrome to determine if female mice harbor a sex-specific defect in salivary-gland-protective regulatory T (Treg) cells. Transfer of cervical lymph node (LN) cells from female NOD mice into sex-matched NOD-severe combined immunodeficient (SCID) recipients resulted in sialadenitis, regardless of the presence or absence of Treg cells. In contrast, transfer of cervical LN cells from male NOD mice into sex-matched NOD-SCID recipients only resulted in sialadenitis when Treg cells were depleted before transfer, suggesting that male NOD mice have functional salivary-gland-protective Treg cells. Notably, the host environment affected the ability of Treg cells to prevent sialadenitis with testosterone promoting salivary gland protection. Treg cells from male mice did not protect against sialadenitis in female recipients. Testosterone treatment of female recipients of bulk cervical LN cells decreased sialadenitis, and Treg cells from female mice were capable of protecting against development of sialadenitis in male recipients. Hence, our data demonstrate that female NOD mice develop sialadenitis through a defect in salivary-gland-protective Treg cells that can be reversed in the presence of testosterone. Copyright © 2018 John Wiley & Sons Ltd.
Publication Type
Journal Article. Research Support, N.I.H., Extramural. Research Support, Non-U.S. Gov't.
Year of Publication
2018

<101>
Unique Identifier
29745339
Title

Musculoskeletal and Rheumatic Diseases Induced by Immune Checkpoint Inhibitors: A Review of the Literature. [REVIEW]

Source

Current Drug Safety. 13(3):150-164, 2018.

VI 1

Status

MEDLINE

Authors

Benfaremo D; Manfredi L; Luchetti MM; Gabrielli A.

Authors Full Name

Benfaremo, Devis; Manfredi, Lucia; Luchetti, Michele Maria; Gabrielli, Armando.

Institution

Benfaremo, Devis. Dipartimento di Scienze Cliniche e Molecolari, Universita Politecnica delle Marche, Ancona, Italy.

Manfredi, Lucia. Dipartimento di Scienze Cliniche e Molecolari, Universita Politecnica delle Marche, Ancona, Italy.

Luchetti, Michele Maria. Dipartimento di Scienze Cliniche e Molecolari, Universita Politecnica delle Marche, Ancona, Italy.

Gabrielli, Armando. Dipartimento di Scienze Cliniche e Molecolari, Universita Politecnica delle Marche, Ancona, Italy.

Abstract

BACKGROUND: Immune checkpoint inhibitors are a new promising class of antitumor drugs that have been associated with a number of immune-related Adverse Events (AEs), including musculoskeletal and rheumatic disease.

METHODS: We searched Medline reviewing reports of musculoskeletal and rheumatic AEs induced by immune checkpoint inhibitors.

RESULTS: Several musculoskeletal and rheumatic AEs associated with immune checkpoint inhibitors treatment are reported in the literature. In particular, arthralgia and myalgia were the most common reported AEs, whereas the prevalence of arthritis, myositis and vasculitis is less characterized and mainly reported in case series and case reports. Other occasionally described AEs are sicca syndrome, polymyalgia rheumatica, systemic lupus erythematosus and sarcoidosis.

CONCLUSION: Newly induced musculoskeletal and rheumatic diseases are a frequent adverse event associated with immune checkpoint inhibitors treatment. Copyright© Bentham Science Publishers; For any queries, please email at epub@benthamscience.org.

Publication Type

Journal Article. Review.

Year of Publication

2018

<102>

Unique Identifier

29740441

Title

Targeting B Cells and Plasma Cells in Autoimmune Diseases. [Review]

Source

Frontiers in Immunology. 9:835, 2018.

VI 1

Status

MEDLINE

Authors

Hofmann K; Clauder AK; Manz RA.

Authors Full Name

Hofmann, Katharina; Clauder, Ann-Katrin; Manz, Rudolf Armin.

Institution

Hofmann, Katharina. Institute for Systemic Inflammation Research, University of Luebeck, Luebeck, Schleswig-Holstein, Germany.

Clauder, Ann-Katrin. Institute for Systemic Inflammation Research, University of Luebeck, Luebeck, Schleswig-Holstein, Germany.

Manz, Rudolf Armin. Institute for Systemic Inflammation Research, University of Luebeck, Luebeck, Schleswig-Holstein, Germany.

Abstract

Success with B cell depletion using rituximab has proven the concept that B lineage cells represent a valid target for the treatment of autoimmune diseases, and has promoted the development of other B cell targeting agents. Present data confirm that B cell depletion is

beneficial in various autoimmune disorders and also show that it can worsen the disease course in some patients. These findings suggest that B lineage cells not only produce pathogenic autoantibodies, but also significantly contribute to the regulation of inflammation. In this review, we will discuss the multiple pro- and anti-inflammatory roles of B lineage cells play in autoimmune diseases, in the context of recent findings using B lineage targeting therapies.

Publication Type

Journal Article. Research Support, Non-U.S. Gov't. Review.

Year of Publication

2018

<103>

Unique Identifier

29733768

Title

In this issue: Role of immune cells and molecules in rheumatoid arthritis pathogenesis and cancer immunotherapy.

Source

International Reviews of Immunology. 37(3):127-128, 2018 05 04.

VI 1

Status

MEDLINE

Authors

Kumar H; Bot A.

Authors Full Name

Kumar, Himanshu; Bot, Adrian.

Institution

Kumar, Himanshu. a International Reviews of Immunology, Associate Professor, Laboratory of Immunology and Infectious Disease Biology, Indian Institute of Science Education and Research (IISER), Bhopal, India.

Bot, Adrian. b International Reviews of Immunology and Vice President, Translational Sciences, Kite Pharma Inc., Santa Monica, CA, USA.

Publication Type

Editorial. Introductory Journal Article.

Year of Publication
2018

<104>
Unique Identifier
29724733
Title
Effects of immune checkpoint inhibitors on B cells: relationship to immune-related adverse events. Annals of the Rheumatic Diseases. 77(6):795-796, 2018 06.
VI 1
Status
MEDLINE
Authors
Pisetsky DS.
Authors Full Name
Pisetsky, David S.
Institution
Pisetsky, David S. Department of Medicine, Duke University Medical Center and Medical Research Service, VA Medical Center, Durham, NC, USA.
Comment on (CON)
Publication Type
Journal Article. Comment.
Year of Publication
2018

<105>
Unique Identifier
29722740
Title

Rheumatoid Meningitis: A Case Review.

Source

Neurologist. 23(3):83-85, 2018 May.

VI 1

Status

MEDLINE

Authors

Parsons AM; Zuniga LA; Hoxworth JM; Lyons M; Aslam F; Goodman BP.

Authors Full Name

Parsons, Angela M; Zuniga, Leslie A; Hoxworth, Joseph M; Lyons, Mark; Aslam, Fawad; Goodman, Brent P.

Institution

Parsons, Angela M. Department of Neurology.
Zuniga, Leslie A. Department of Neurology.
Hoxworth, Joseph M. Department of Radiology.
Hoxworth, Joseph M. Division of Otolaryngology Head and Neck Surgery.
Lyons, Mark. Department of Neurosurgery, Mayo Clinic Hospital, Phoenix, AZ.
Aslam, Fawad. Division of Rheumatology, Mayo Clinic, Scottsdale.
Goodman, Brent P. Department of Neurology.

Abstract

INTRODUCTION: Rheumatoid meningitis (RM) is a rare complication of rheumatoid arthritis (RA) and has a high mortality rate. It can present as a first diagnosis of RA, in long-standing disease, or in active or well-controlled disease. Neurological manifestations vary widely.

CASE REPORT: A patient with a 30-year history of RA, well controlled with methotrexate therapy, presented with new-onset seizures. Magnetic resonance imaging showed leptomeningeal and pachymeningeal enhancement. A de novo workup resulted in diagnosis of RM.

CONCLUSIONS: Cerebrospinal fluid findings for RM are nonspecific, typically lymphocytic pleocytosis; however, they can be neutrophilic, as in this case. Magnetic resonance imaging findings consist of leptomeningeal and pachymeningeal enhancement but can also involve the parenchyma. The diagnosis is typically confirmed with meningeal biopsy. Treatment involves high-dose corticosteroids or immunomodulatory therapy, or both. Long-term follow-up with radiologic surveillance typically ranges from improvement to resolution.

Publication Type

Case Reports. Journal Article.

Year of Publication
 2018

<106>
Unique Identifier
 29694398
Title
 Commensurate incidence and outcomes of liver enzyme elevation between anti-tumor necrosis factor users with or without prior hepatitis B virus infections.
Source
 PLoS ONE [Electronic Resource]. 13(4):e0196210, 2018.
VI 1
Status
 MEDLINE
Authors
 Chiu YM; Lai MS; Chan KA.
Author NameID
 Chiu, Ying-Ming; ORCID: https://orcid.org/0000-0003-4548-5331
Authors Full Name
 Chiu, Ying-Ming; Lai, Mei-Shu; Chan, K Arnold.
Institution
 Chiu, Ying-Ming. Division of Allergy, Immunology and Rheumatology, Changhua Christian Hospital, Changhua City, Taiwan.
 Chiu, Ying-Ming. Department of Nursing, College of Medicine and Nursing, Hungkuang University, Taichung City, Taiwan.
 Lai, Mei-Shu. Graduate Institute of Epidemiology and Preventive Medicine, College of Public Health, National Taiwan University, Taipei, Taiwan.
 Chan, K Arnold. Department of Medical Research, National Taiwan University Hospital, Taipei, Taiwan.
 Chan, K Arnold. Graduate Institute of Oncology, College of Medicine, National Taiwan University, Taipei, Taiwan.
Abstract

BACKGROUND AND OBJECTIVE: Potential hepatoxicity is an important clinical concern when administering immunosuppressive therapies to patients infected by hepatitis B virus (HBV). Tumor necrosis factor inhibitors (anti-TNF) increase the likelihood of hepatitis consequent to HBV reactivation, but reported risks and outcomes vary. We determined the risks of liver enzyme elevation in anti-rheumatic drug users from an HBV-endemic region with differing HBV serostatus.

METHODS: We established retrospective cohorts with rheumatoid arthritis, ankylosing spondylitis, or psoriasis/psoriatic arthritis who: 1) received anti-TNF agents from 1 January 2004 to 30 June 2013; 2) received care from 1 June 2011 to 30 June 2013 but only ever used conventional disease-modifying anti-rheumatic drugs (DMARDs). Serology results defined three subgroups: HBV surface antigen positive (HBsAg+), HBsAg negative/HBV core antibody positive (HBsAg-/HBcAb+), or uninfected. We compared incidences of serum alanine aminotransferase (ALT) exceeding twice the upper reference limit between HBV serostatus subgroups in each treatment cohort.

RESULTS: Among 783 patients treated with anti-TNF (n = 472) or DMARDs only (n = 311), HBsAg-/HBcAb+ anti-TNF users had incidence of ALT elevation commensurate with uninfected counterparts (6.1 vs. 6.0/100 person-years), compared to 19.6/100 person-years in HBsAg+ patients (standardized rate ratio 3.3, 95% CI 1.3-8.2); none effected had severe or fatal hepatitis and ALT levels in all HBsAg-/HBcAb+ patients remained stable, mostly normalizing spontaneously, or after moderating treatment. Patterns of of ALT elevation associated with differing HBV serostatus in the DMARD cohort, resembled those in anti-TNF users.

CONCLUSIONS: In this large HBV-endemic cohort, the absolute incidence of ALT elevation in anti-TNF users was more than three-fold higher in HBsAg+ patients than in uninfected counterparts; however, no such association was evident in patients with HBsAg-/HBcAb+ serotype, whose risk and outcomes of liver enzyme elevation were similar to uninfected patients, suggesting that anti-TNF use by HBsAg-/HBcAb+ patients is probably safe.

Publication Type

Journal Article. Research Support, Non-U.S. Gov't.

Year of Publication

2018

<107>
Unique Identifier
29676371
Title
Methotrexate-associated Epstein-Barr virus mucocutaneous ulcer: A case report and review of literature. [Review]
Source
Indian Journal of Pathology & Microbiology. 61(2):255-257, 2018 Apr-Jun.
VI 1
Status
MEDLINE
Authors
Ravi PY; Sigamani E; Jeelani Y; Manipadam MT.
Authors Full Name
Ravi, Priyanka Yogendra; Sigamani, Elanthenral; Jeelani, Yasir; Manipadam, Marie Therese.
Institution
Ravi, Priyanka Yogendra. Department of General Pathology, Christian Medical College, Vellore, Tamil Nadu, India.
Sigamani, Elanthenral. Department of General Pathology, Christian Medical College, Vellore, Tamil Nadu, India.
Jeelani, Yasir. Department of General Pathology, Christian Medical College, Vellore, Tamil Nadu, India.
Manipadam, Marie Therese. Department of General Pathology, Christian Medical College, Vellore, Tamil Nadu, India.
Abstract
Epstein-Barr virus-positive mucocutaneous ulcer (EBVMCU) comprises part of the spectrum of B-cell lymphoproliferative disorders, reported in settings of immunosenescence and iatrogenic immunosuppression, affecting the oropharyngeal mucosa, skin, and gastrointestinal tract. We report a case of a 59-year-old female, known case of rheumatoid arthritis on methotrexate (MTX) for 15 years, who presented with an ulcer in the inner aspect of her cheek region for 2 years. Clinical examination revealed an infiltrative lesion involving the lower gingivobuccal sulcus of size 2 cm x 3 cm extending to the alveolus with level I lymph nodes, suspicious for carcinoma buccal mucosa. Anti-EBV-capsid antigen-immunoglobulin M and qualitative EBV polymerase chain reaction of peripheral blood were negative. Histopathological examination revealed atypical lymphoid cells with enlarged vesicular nuclei, prominent nucleoli, and moderate eosinophilic cytoplasm, few with binucleation (CD20 focally positive, CD79a focally positive, CD30+, EBV

LMP-1+, MIB-I 60%) consistent with EBVMCU, MTX-associated. This is the first case report from India.

Publication Type

Case Reports. Journal Article. Review.

Year of Publication

2018

<108>

Unique Identifier

29675950

Title

Risk factors for red blood cell alloimmunization in the Recipient Epidemiology and Donor Evaluation Study (REDS-III) database.

Source

British Journal of Haematology. 181(5):672-681, 2018 06.

VI 1

Status

MEDLINE

Authors

Karafin MS; Westlake M; Hauser RG; Tormey CA; Norris PJ; Roubinian NH; Wu Y; Triulzi DJ; Kleinman S; Hendrickson JE; NHLBI Recipient Epidemiology and Donor Evaluation Study-III (REDS-III).

Author NameID

Tormey, Christopher A; ORCID: https://orcid.org/0000-0003-1785-2245

Hendrickson, Jeanne E; ORCID: https://orcid.org/0000-0002-7928-3132

Authors Full Name

Karafin, Matthew S; Westlake, Matt; Hauser, Ronald G; Tormey, Christopher A; Norris, Philip J; Roubinian, Nareg H; Wu, Yanyun; Triulzi, Darrell J; Kleinman, Steve; Hendrickson, Jeanne E; NHLBI Recipient Epidemiology and Donor Evaluation Study-III (REDS-III).

Institution

Karafin, Matthew S. Blood Center of Wisconsin, Milwaukee, WI, USA.

Westlake, Matt. RTI International, Rockville, MD, USA.

Hauser, Ronald G. Department of Laboratory Medicine, Yale University, New Haven, CT, USA.

Hauser, Ronald G. VA CT, West Haven, CT, USA.
Tormey, Christopher A. Department of Laboratory Medicine, Yale University, New Haven, CT, USA.
Tormey, Christopher A. VA CT, West Haven, CT, USA.
Norris, Philip J. University of California, San Francisco, CA, USA.
Norris, Philip J. Blood Systems Research Institute, San Francisco, CA, USA.
Roubinian, Nareg H. University of California, San Francisco, CA, USA.
Roubinian, Nareg H. Blood Systems Research Institute, San Francisco, CA, USA.
Wu, Yanyun. Department of Laboratory Medicine, Yale University, New Haven, CT, USA.
Wu, Yanyun. Bloodworks Northwest, Seattle, WA, USA.
Triulzi, Darrell J. University of Pittsburgh and Institute of Transfusion Medicine, Pittsburgh, PA, USA.
Kleinman, Steve. University of British Columbia, Vancouver, British Columbia, Canada.
Hendrickson, Jeanne E. Department of Laboratory Medicine, Yale University, New Haven, CT, USA.
Hendrickson, Jeanne E. Department of Pediatrics, Yale University, New Haven, CT, USA.
Abstract
Despite the significance of red blood cell (RBC) alloimmunization, the lack of standardized registries in the US has prevented the completion of large studies. Data from 3.5 years of the Recipient Epidemiology and Donor Evaluation Study-III (REDS-III) recipient database, containing information from 12 hospitals, were studied. A RBC alloantibody responder had an antibody identified at any point during the study, and a non-responder had a negative antibody screen at least 15 days post-RBC transfusion. Demographics, blood type, ICD9/10 codes, and other potential correlates were evaluated. Of 319 177 (2.07%) screened patients, 6597 had a total of 8892 clinically significant RBC alloantibodies identified, with 75% being in the Rh or Kell families. Alloimmunization was more common in females (2.38%) than males (1.68%), and in RhD negative (2.82%) than RhD positive (1.94%) patients. Age, sex, RhD status and race were associated with being a responder, and certain diagnoses (including sickle cell disease or trait, systemic lupus erythematosus, rheumatoid arthritis and myelodysplastic syndrome) were more common among responders than non-responders. Data collected in this multi-centre recipient database provide the largest RBC alloimmunized patient cohort studied in the US, with previously known demographic and disease associations of responder status confirmed, and new associations identified. Copyright © 2018 John Wiley & Sons Ltd.
Publication Type
Evaluation Study. Journal Article. Multicenter Study. Research Support, N.I.H., Extramural.
Year of Publication
2018

<109>
Unique Identifier
29663162
Title
Functional Defects of Treg Cells: New Targets in Rheumatic Diseases, Including Ankylosing Spondylitis. [Review]
Source
Current Rheumatology Reports. 20(5):30, 2018 04 16.
VI 1
Status
MEDLINE
Authors
Miao J; Zhu P.
Authors Full Name
Miao, Jinlin; Zhu, Ping.
Institution
Miao, Jinlin. Department of Clinical Immunology, Xijing Hospital, Fourth Military Medical University, No. 127 West Changle Road, Xi'an, 710032, Shaanxi Province, People's Republic of China.
Zhu, Ping. Department of Clinical Immunology, Xijing Hospital, Fourth Military Medical University, No. 127 West Changle Road, Xi'an, 710032, Shaanxi Province, People's Republic of China. zhuping@fmmu.edu.cn.
Abstract
PURPOSE OF REVIEW: This study aims to review the advances of Treg cell biology, the functional defects of Treg cells, and the potential strategies for the experimental, preclinical or clinical application of Treg cell therapy in the context of autoimmune/immune-mediated rheumatic diseases.

RECENT FINDINGS: CD4+CD25+ regulatory T (Treg) cells are a phenotypically and functionally heterogeneous subset of lymphocytes that prevent a variety of autoimmune diseases. As in many autoimmune diseases, the functional defects of Treg cells are supposed to play relevant roles in the pathogenesis and development of systemic lupus erythematosus,

rheumatoid arthritis, ankylosing spondylitis, and other autoimmune/immune-mediated rheumatic diseases. Consequently, manipulation and modulation of Treg cells represent a potent strategy for therapeutic benefit in many such diseases. A further understanding of the functional defects of Treg cells in rheumatic diseases will contribute to find new targets and therapies in rheumatic diseases, including ankylosing spondylitis.

Publication Type

Journal Article. Review.

Year of Publication

2018

<110>

Unique Identifier

29661455

Title

Management of Juvenile Idiopathic Arthritis in ABO-incompatible Kidney Transplantation: A Case Report.

Source

Transplantation Proceedings. 50(3):869-872, 2018 Apr.

VI 1

Status

MEDLINE

Authors

Ishikawa S; Tasaki M; Kuroda T; Kobayashi D; Saito K; Nakagawa Y; Ikeda M; Takahashi K; Tomita Y.

Authors Full Name

Ishikawa, S; Tasaki, M; Kuroda, T; Kobayashi, D; Saito, K; Nakagawa, Y; Ikeda, M; Takahashi, K; Tomita, Y.

Institution

Ishikawa, S. Division of Urology, Department of Regenerative and Transplant Medicine, Niigata Graduate School of Medical and Dental Sciences, Niigata, Japan.

Tasaki, M. Division of Urology, Department of Regenerative and Transplant Medicine, Niigata Graduate School of Medical and Dental Sciences, Niigata, Japan. Electronic address: masa1214@med.niigata-u.ne.jp.

Kuroda, T. Division of Clinical Nephrology and Rheumatology, Graduate School of Medical and Dental Sciences, Niigata University, Niigata, Japan.
Kobayashi, D. Division of Clinical Nephrology and Rheumatology, Graduate School of Medical and Dental Sciences, Niigata University, Niigata, Japan.
Saito, K. Division of Urology, Department of Regenerative and Transplant Medicine, Niigata Graduate School of Medical and Dental Sciences, Niigata, Japan.
Nakagawa, Y. Division of Urology, Department of Regenerative and Transplant Medicine, Niigata Graduate School of Medical and Dental Sciences, Niigata, Japan.
Ikeda, M. Division of Urology, Department of Regenerative and Transplant Medicine, Niigata Graduate School of Medical and Dental Sciences, Niigata, Japan.
Takahashi, K. Division of Urology, Department of Regenerative and Transplant Medicine, Niigata Graduate School of Medical and Dental Sciences, Niigata, Japan.
Tomita, Y. Division of Urology, Department of Regenerative and Transplant Medicine, Niigata Graduate School of Medical and Dental Sciences, Niigata, Japan.
Abstract
Biologic agents are a beneficial therapy for juvenile idiopathic arthritis (JIA). However, there is a lack of evidence with regard to management of these agents for JIA patients who undergo kidney transplantation (KTx). A 36-year-old woman with JIA who was treated with tocilizumab targeting interleukin-6 (IL-6) receptor underwent ABO-incompatible kidney transplantation (ABOi KTx). To prevent over-immunosuppression, tocilizumab was discontinued before ABOi KTx. Rituximab, tacrolimus, mycophenolate mofetil, everolimus, and methylprednisolone were used for immunosuppression. Clinical remission of joint pain was maintained for over 3 years despite complete discontinuation of tocilizumab. Both serum IL-6 and soluble IL-6 receptor levels were markedly decreased, suggesting that multitargeted immunosuppression for ABOi KTx induced long-term clinical remission of JIA through inhibition of the IL-6 pathway. However, levels of C-reactive protein (CRP) and matrix metalloproteinase-3 (MMP-3) gradually increased thereafter and abatacept was initiated to prevent joint deterioration. These levels decreased without any adverse events. The patient's renal graft function was well maintained. Copyright © 2018 Elsevier Inc. All rights reserved.
Publication Type
Case Reports. Journal Article.
Year of Publication
2018

<111>
Unique Identifier
29631064
Title
Autoimmune phenomena and disease in cancer patients treated with immune checkpoint inhibitors. [REVIEW]
Source
Autoimmunity Reviews. 17(6):610-616, 2018 Jun.
VI 1
Status
MEDLINE
Authors
Tocut M; Brenner R; Zandman-Goddard G.
Authors Full Name
Tocut, Milena; Brenner, Ronen; Zandman-Goddard, Gisele.
Institution
Tocut, Milena. Dept. of Internal Medicine C, Wolfson Medical Center, Israel.
Brenner, Ronen. Dept. of Oncology, Wolfson Medical Center, Israel; Sackler Faculty of Medicine, Tel-Aviv University, Israel.
Zandman-Goddard, Gisele. Dept. of Internal Medicine C, Wolfson Medical Center, Israel; Sackler Faculty of Medicine, Tel-Aviv University, Israel. Electronic address: goddard@wmc.gov.il.
Abstract
The discovery and approved treatment with immune checkpoint inhibitors (ICIs) for a variety of cancers has changed dramatically the morbidity and mortality rates for these patients. Despite the obvious benefits, their use is associated with unique immune-related adverse effects (irAEs), including autoimmune conditions such as: inflammatory arthritis, myositis, vasculitis and Sicca syndrome. The appearance of ICIs-induced autoimmune irAE requires from oncologists and rheumatologists a different approach to the identification and treatment of these conditions, which may differ from the classic and traditional approach to rheumatologic diseases. It should be taken into consideration that ICIs therapy in patients with preexisting autoimmunity could be possible, but with a cost of causing disease exacerbation. In this extensive review, we present the autoimmune irAEs, mostly as phenomena, but also as classic autoimmune diseases as well as therapeutic options for the side effects. Copyright © 2018. Published by Elsevier B.V.
Publication Type
Journal Article. Review.
Year of Publication

2018

<112>

Unique Identifier

29618462

Title

Allogeneic hematopoietic stem cell transplantation for severe, refractory juvenile idiopathic arthritis.

Source

Blood Advances. 2(7):777-786, 2018 04 10.

VI 1

Status

MEDLINE

Authors

M F Silva J; Ladomenou F; Carpenter B; Chandra S; Sedlacek P; Formankova R; Grandage V; Friswell M; Cant AJ; Nademi Z; Slatter MA; Gennery AR; Hambleton S; Flood TJ; Lucchini G; Chiesa R; Rao K; Amrolia PJ; Brogan P; Wedderburn LR; Glanville JM; Hough R; Marsh R; Abinun M; Veys P.

Authors Full Name

M F Silva, Juliana; Ladomenou, Fani; Carpenter, Ben; Chandra, Sharat; Sedlacek, Petr; Formankova, Renata; Grandage, Vicky; Friswell, Mark; Cant, Andrew J; Nademi, Zohreh; Slatter, Mary A; Gennery, Andrew R; Hambleton, Sophie; Flood, Terence J; Lucchini, Giovanna; Chiesa, Robert; Rao, Kanchan; Amrolia, Persis J; Brogan, Paul; Wedderburn, Lucy R; Glanville, Julie M; Hough, Rachael; Marsh, Rebecca; Abinun, Mario; Veys, Paul.

Institution

M F Silva, Juliana. Department of BMT, Great Ormond Street Hospital for Children, National Health Service (NHS) Foundation Trust, London, United Kingdom.

Ladomenou, Fani. Department of BMT, Great Ormond Street Hospital for Children, National Health Service (NHS) Foundation Trust, London, United Kingdom.

Carpenter, Ben. Department of Adolescent BMT, University College Hospital, NHS Foundation Trust, London, United Kingdom.

Chandra, Sharat. Division of Bone Marrow Transplantation and Immune Deficiency, Cincinnati Children's Hospital Medical Center, Cincinnati, OH.

Chandra, Sharat. Department of Pediatrics, University of Cincinnati, Cincinnati, OH.

Sedlacek, Petr. Department of Pediatric Hematology and Oncology, Teaching Hospital Motol, Prague, Czech Republic.

Formankova, Renata. Department of Pediatric Hematology and Oncology, Teaching Hospital Motol, Prague, Czech Republic.

Grandage, Vicky. Department of Adolescent BMT, University College Hospital, NHS Foundation Trust, London, United Kingdom.

Friswell, Mark. Great North Children's Hospital, Royal Victoria Infirmary, Newcastle upon Tyne, United Kingdom.

Friswell, Mark. Primary Immunodeficiency Group, Institute of Cellular Medicine, Newcastle University, Newcastle upon Tyne, United Kingdom.

Cant, Andrew J. Great North Children's Hospital, Royal Victoria Infirmary, Newcastle upon Tyne, United Kingdom.

Cant, Andrew J. Primary Immunodeficiency Group, Institute of Cellular Medicine, Newcastle University, Newcastle upon Tyne, United Kingdom.

Nademi, Zohreh. Great North Children's Hospital, Royal Victoria Infirmary, Newcastle upon Tyne, United Kingdom.

Nademi, Zohreh. Primary Immunodeficiency Group, Institute of Cellular Medicine, Newcastle University, Newcastle upon Tyne, United Kingdom.

Slatter, Mary A. Great North Children's Hospital, Royal Victoria Infirmary, Newcastle upon Tyne, United Kingdom.

Slatter, Mary A. Primary Immunodeficiency Group, Institute of Cellular Medicine, Newcastle University, Newcastle upon Tyne, United Kingdom.

Gennery, Andrew R. Great North Children's Hospital, Royal Victoria Infirmary, Newcastle upon Tyne, United Kingdom.

Gennery, Andrew R. Primary Immunodeficiency Group, Institute of Cellular Medicine, Newcastle University, Newcastle upon Tyne, United Kingdom.

Hambleton, Sophie. Great North Children's Hospital, Royal Victoria Infirmary, Newcastle upon Tyne, United Kingdom.

Hambleton, Sophie. Primary Immunodeficiency Group, Institute of Cellular Medicine, Newcastle University, Newcastle upon Tyne, United Kingdom.

Flood, Terence J. Great North Children's Hospital, Royal Victoria Infirmary, Newcastle upon Tyne, United Kingdom.

Flood, Terence J. Primary Immunodeficiency Group, Institute of Cellular Medicine, Newcastle University, Newcastle upon Tyne, United Kingdom.

Lucchini, Giovanna. Department of BMT, Great Ormond Street Hospital for Children, National Health Service (NHS) Foundation Trust, London, United Kingdom.

Chiesa, Robert. Department of BMT, Great Ormond Street Hospital for Children, National Health Service (NHS) Foundation Trust, London, United Kingdom.
Rao, Kanchan. Department of BMT, Great Ormond Street Hospital for Children, National Health Service (NHS) Foundation Trust, London, United Kingdom.
Amrolia, Persis J. Department of BMT, Great Ormond Street Hospital for Children, National Health Service (NHS) Foundation Trust, London, United Kingdom.
Brogan, Paul. Department of Rheumatology, Great Ormond Street Hospital for Children, NHS Foundation Trust, London, United Kingdom; and.
Wedderburn, Lucy R. Department of Rheumatology, Great Ormond Street Hospital for Children, NHS Foundation Trust, London, United Kingdom; and.
Wedderburn, Lucy R. University College London Great Ormond Street Institute of Child Health and NIHR GOSH Biomedical Research Centre, NHS Trust, London, United Kingdom.
Glanville, Julie M. Department of BMT, Great Ormond Street Hospital for Children, National Health Service (NHS) Foundation Trust, London, United Kingdom.
Hough, Rachael. Department of Adolescent BMT, University College Hospital, NHS Foundation Trust, London, United Kingdom.
Marsh, Rebecca. Division of Bone Marrow Transplantation and Immune Deficiency, Cincinnati Children's Hospital Medical Center, Cincinnati, OH.
Marsh, Rebecca. Department of Pediatrics, University of Cincinnati, Cincinnati, OH.
Abinun, Mario. Great North Children's Hospital, Royal Victoria Infirmary, Newcastle upon Tyne, United Kingdom.
Abinun, Mario. Primary Immunodeficiency Group, Institute of Cellular Medicine, Newcastle University, Newcastle upon Tyne, United Kingdom.
Veys, Paul. Department of BMT, Great Ormond Street Hospital for Children, National Health Service (NHS) Foundation Trust, London, United Kingdom.
Abstract
Patients with juvenile idiopathic arthritis (JIA) can experience a severe disease course, with progressive destructive polyarthritis refractory to conventional therapy with disease-modifying antirheumatic drugs including biologics, as well as life-threatening complications including macrophage activation syndrome (MAS). Allogeneic hematopoietic stem cell transplantation (allo-HSCT) is a potentially curative immunomodulatory strategy for patients with such refractory disease. We treated 16 patients in 5 transplant centers between 2007 and 2016: 11 children with systemic JIA and 5 with rheumatoid factor-negative polyarticular JIA; all were either refractory to standard therapy, had developed secondary hemophagocytic lymphohistiocytosis/MAS poorly responsive to treatment, or had failed autologous HSCT. All children received reduced toxicity fludarabine-based conditioning regimens and serotherapy with alemtuzumab. Fourteen of 16 patients are alive with a median follow-up of 29 months (range, 2.8-96 months). All patients had

hematological recovery. Three patients had grade II-IV acute graft-versus-host disease. The incidence of viral infections after HSCT was high, likely due to the use of alemtuzumab in already heavily immunosuppressed patients. All patients had significant improvement of arthritis, resolution of MAS, and improved quality of life early following allo-HSCT; most importantly, 11 children achieved complete drug-free remission at the last follow-up. Allo-HSCT using alemtuzumab and reduced toxicity conditioning is a promising therapeutic option for patients with JIA refractory to conventional therapy and/or complicated by MAS. Long-term follow-up is required to ascertain whether disease control following HSCT continues indefinitely. Copyright © 2018 by The American Society of Hematology.

Publication Type

Journal Article. Multicenter Study. Research Support, Non-U.S. Gov't.

Year of Publication

2018

<113>

Unique Identifier

29604049

Title

Aberrant cell signalling in PBMCs upon IFN-alpha stimulation in primary Sjogren's syndrome patients associates with type I interferon signature.

Source

European Journal of Immunology. 48(7):1217-1227, 2018 07.

VI 1

Status

MEDLINE

Authors

Davies R; Hammenfors D; Bergum B; Vogelsang P; Gavasso S; Brun JG; Jonsson R; Appel S.

Author NameID

Appel, Silke; ORCID: https://orcid.org/0000-0002-2199-2315

Authors Full Name

Davies, Richard; Hammenfors, Daniel; Bergum, Brith; Vogelsang, Petra; Gavasso, Sonia; Brun, Johan G; Jonsson, Roland; Appel, Silke.

Institution

Davies, Richard. Broegelmann Research Laboratory, Department of Clinical Science, University of Bergen, Bergen, Norway.
Hammenfors, Daniel. Broegelmann Research Laboratory, Department of Clinical Science, University of Bergen, Bergen, Norway.
Hammenfors, Daniel. Department of Rheumatology, Haukeland University Hospital, Bergen, Norway.
Bergum, Brith. Broegelmann Research Laboratory, Department of Clinical Science, University of Bergen, Bergen, Norway.
Vogelsang, Petra. Broegelmann Research Laboratory, Department of Clinical Science, University of Bergen, Bergen, Norway.
Gavasso, Sonia. Department of Rheumatology, Haukeland University Hospital, Bergen, Norway.
Brun, Johan G. Department of Rheumatology, Haukeland University Hospital, Bergen, Norway.
Brun, Johan G. Department of Clinical Science, University of Bergen, Bergen, Norway.
Jonsson, Roland. Broegelmann Research Laboratory, Department of Clinical Science, University of Bergen, Bergen, Norway.
Jonsson, Roland. Department of Rheumatology, Haukeland University Hospital, Bergen, Norway.
Appel, Silke. Broegelmann Research Laboratory, Department of Clinical Science, University of Bergen, Bergen, Norway.
Comments
Erratum in (EIN)
Abstract
Primary Sjogren's syndrome (pSS) is a complex systemic autoimmune disease with heterogeneous disease manifestations. Genetic predisposition, hormonal and environmental factors are all thought to contribute to disease etiology and pathogenesis. A better understanding of the disease pathogenesis is required in order to establish new targeted therapies. We analysed MAPK/ERK and JAK/STAT signalling networks in peripheral blood mononuclear cells (PBMCs) upon stimulation with interferon alpha 2b (IFN-alpha2b) by flow cytometry to define potentially dysfunctional intracellular signalling pathways involved in disease pathogenesis. Cells derived from pSS patients displayed small but significant increases in basal phosphorylation levels of numerous signalling proteins compared to cells from healthy donors. The phosphorylation profiles following stimulation with IFNalpha2b differed significantly between pSS patients and healthy donors, especially regarding STAT1 Y701. PCA further grouped patients according to clinical characteristics. Type I IFN induced gene expression was found to negatively correlate with the IFN-alpha2b induced phosphorylation of STAT3 S727 in T cells and positively with pSTAT1 Y701 in B cells. Increases in pSTAT1 Y701 were associated with the presence of autoantibodies. Our

results indicate involvement of both STAT3 S727 and STAT1 Y701 pathways in pSS patients. Therapies targeting these pathways might therefore be beneficial for certain subgroups of patients. Copyright © 2018 WILEY-VCH Verlag GmbH & Co. KGaA, Weinheim.

Publication Type

Journal Article. Research Support, Non-U.S. Gov't.

Year of Publication

2018

<114>

Unique Identifier

29573850

Title

Clinical presentation of immune checkpoint inhibitor-induced inflammatory arthritis differs by immunotherapy regimen. Seminars in Arthritis & Rheumatism. 48(3):553-557, 2018 12.

Authors

Cappelli LC; Brahmer JR; Forde PM; Le DT; Lipson EJ; Naidoo J; Zheng L; Bingham CO 3rd; Shah AA.

Authors Full Name

Cappelli, Laura C; Brahmer, Julie R; Forde, Patrick M; Le, Dung T; Lipson, Evan J; Naidoo, Jarushka; Zheng, Lei; Bingham, Clifton O 3rd; Shah, Ami A.

Institution

Cappelli, Laura C. Johns Hopkins University, Division of Rheumatology, 5501 Hopkins Bayview Circle, Suite 1.B.1, Baltimore, MD. Electronic address: lcappel1@jhmi.edu.

Brahmer, Julie R. Johns Hopkins University, Department of Oncology, Baltimore, MD.

Forde, Patrick M. Johns Hopkins University, Department of Oncology, Baltimore, MD.

Le, Dung T. Johns Hopkins University, Department of Oncology, Baltimore, MD.

Lipson, Evan J. Johns Hopkins University, Department of Oncology, Baltimore, MD.

Naidoo, Jarushka. Johns Hopkins University, Department of Oncology, Baltimore, MD.

Zheng, Lei. Johns Hopkins University, Department of Oncology, Baltimore, MD.

Bingham, Clifton O 3rd. Johns Hopkins University, Division of Rheumatology, 5501 Hopkins Bayview Circle, Suite 1.B.1, Baltimore, MD.

Shah, Ami A. Johns Hopkins University, Division of Rheumatology, 5501 Hopkins Bayview Circle, Suite 1.B.1, Baltimore, MD.
Abstract
INTRODUCTION: Immune checkpoint inhibitors (ICIs) are a class of cancer immunotherapy, increasingly utilized to treat malignancies. Inflammatory arthritis (IA) is a potential consequence of ICI use, but there is limited information to guide evaluation and management of this immune-related adverse event (irAE). This study aimed to characterize clinical phenotypes, IA treatment and response in the largest cohort of patients with ICI-induced IA reported to date.

METHODS: Patients with rheumatologist-confirmed IA occurring during or after ICI treatment with no prior history of autoimmune disease were included. Data were analyzed by ICI treatment regimen; treatments included combination CTLA-4/PD-1 inhibition, anti-PD-1 or anti-PD-L1 monotherapy. Relationship to the development of other irAEs, management of IA, and outcomes of IA management were evaluated.

RESULTS: Of 30 patients identified, those treated with combination ICI therapy were more likely to present with knee arthritis, to have higher levels of C-reactive protein, to have already had another irAE, and to have a reactive arthritis-like phenotype. In contrast, patients treated with ICI monotherapy were more likely to have initial small joint involvement and to have IA as their only irAE. Ten patients required additional immunosuppression beyond corticosteroids, with TNF-inhibitors and/or methotrexate. Tumor progression while on non-corticosteroid immunosuppression was not seen in those with initial tumor response to ICIs.

CONCLUSION: These data suggest that distinct IA phenotypes may emerge with exposure to different ICI regimens. The majority of patients referred to rheumatology required systemic immunosuppression to manage their IA symptoms. Tumor progression was not seen in patients requiring TNF-inhibitors. Copyright © 2018 Elsevier Inc. All rights reserved.
Publication Type
Journal Article. Research Support, N.I.H., Extramural. Research Support, Non-U.S. Gov't.
Year of Publication
2018

<115>

Unique Identifier

29572291

Title

Impact of temporary methotrexate discontinuation for 2 weeks on immunogenicity of seasonal influenza vaccination in patients with rheumatoid arthritis: a randomised clinical trial.

Source

Annals of the Rheumatic Diseases. 77(6):898-904, 2018 06.

VI 1

Status

MEDLINE

Authors

Park JK; Lee YJ; Shin K; Ha YJ; Lee EY; Song YW; Choi Y; Winthrop KL; Lee EB.

Authors Full Name

Park, Jin Kyun; Lee, Yun Jong; Shin, Kichul; Ha, You-Jung; Lee, Eun Young; Song, Yeong Wook; Choi, Yunhee; Winthrop, Kevin L; Lee, Eun Bong.

Institution

Park, Jin Kyun. Division of Rheumatology, Department of Internal Medicine, Seoul National University College of Medicine, Seoul, Korea.

Lee, Yun Jong. Division of Rheumatology, Department of Internal Medicine, Seoul National University Bundang Hospital, Gyeonggi-do, Korea.

Shin, Kichul. Department of Internal Medicine, Seoul Metropolitan Government-Seoul National University Boramae Medical Center, Seoul, Korea.

Ha, You-Jung. Division of Rheumatology, Department of Internal Medicine, Seoul National University Bundang Hospital, Gyeonggi-do, Korea.

Lee, Eun Young. Division of Rheumatology, Department of Internal Medicine, Seoul National University College of Medicine, Seoul, Korea.

Song, Yeong Wook. Division of Rheumatology, Department of Internal Medicine, Seoul National University College of Medicine, Seoul, Korea.

Choi, Yunhee. Division of Medical Statistics, Medical Research Collaborating Center, Seoul National University College of Medicine, Seoul National University Hospital, Seoul, Korea.

Winthrop, Kevin L. Division of Infectious Diseases, Oregon Health and Science University, Portland, Oregon, USA.

Lee, Eun Bong. Division of Rheumatology, Department of Internal Medicine, Seoul National University College of Medicine, Seoul, Korea.

Abstract

OBJECTIVE: To determine whether a 2-week methotrexate (MTX) discontinuation after vaccination improves the efficacy of seasonal influenza vaccination in patients with rheumatoid arthritis (RA).

METHODS: In this prospective randomised parallel-group multicentre study, patients with RA on stable dose of MTX were randomly assigned at a ratio of 1:1 to continue MTX or to hold MTX for 2 weeks after 2016-2017 quadrivalent seasonal influenza vaccine containing H1N1, H3N2, B-Yamagata and B-Victoria. The primary outcome was frequency of satisfactory vaccine response, defined as greater than or equal to fourfold increase of haemagglutination inhibition (HI) antibody titre at 4 weeks after vaccination against >=2 of four vaccine strains. Secondary endpoints included seroprotection (ie, HI titre >=1:40) rate, fold change in antibody titres.

RESULTS: The modified intention-to-treat population included 156 patients in the MTX-continue group and 160 patients in the MTX-hold group. More patients in MTX-hold group achieved satisfactory vaccine response than the MTX-continue group (75.5% vs 54.5%, p<0.001). Seroprotection rate was higher in the MTX-hold group than the MTX-continue group for all four antigens (H1N1: difference 10.7%, 95% CI 2.0% to 19.3%; H3N2: difference 15.9%, 95% CI 5.9% to 26.0%; B-Yamagata: difference13.7%, 95% CI 5.2% to 22.4%; B-Victoria: difference 14.7%, 95% CI 4.5% to 25.0%). The MTX-hold group showed higher fold increase in their antibody titres against all four influenza antigens (all p<0.05). Change in disease activity was similar between groups.

CONCLUSIONS: A temporary MTX discontinuation for 2 weeks after vaccination improves the immunogenicity of seasonal influenza vaccination in patients with RA without increasing RA disease activity.

TRIAL REGISTRATION: NCT02897011. Copyright © Article author(s) (or their employer(s) unless otherwise stated in the text of the article) 2018. All rights reserved. No commercial use is permitted unless otherwise expressly granted.

Publication Type

Journal Article. Multicenter Study. Randomized Controlled Trial. Research Support, Non-U.S. Gov't.

Year of Publication

2018

<116>

Unique Identifier

29545454

Title

A Systematic Review and Metaanalysis of Antirheumatic Drugs and Vaccine Immunogenicity in Rheumatoid Arthritis.

Source

Journal of Rheumatology. 45(6):733-744, 2018 06.

VI 1

Status

MEDLINE

Authors

Subesinghe S; Bechman K; Rutherford AI; Goldblatt D; Galloway JB.

Authors Full Name

Subesinghe, Sujith; Bechman, Katie; Rutherford, Andrew I; Goldblatt, David; Galloway, James B.

Institution

Subesinghe, Sujith. From the Department of Academic Rheumatology, King's College London, Weston Education Centre; Institute of Child Health, University College London, London, UK. ssubesinghe@nhs.net.

Subesinghe, Sujith. S. Subesinghe, BSc, MBBS, MSc, MRCP, Clinical Research Fellow and Rheumatology Registrar, Department of Academic Rheumatology, King's College London, Weston Education Centre; K. Bechman, BSc, MBBS, MRCP, Clinical Research Fellow and Rheumatology Registrar, Department of Academic Rheumatology, King's College London, Weston Education Centre; A.I. Rutherford, BSc, MBBS, MSc, MRCP, Clinical Research Fellow and Rheumatology Registrar, Department of Academic Rheumatology, King's College London, Weston Education Centre; D. Goldblatt, MBChB, FRCPCH, FRCP, PhD, Prof, Vaccinology and Immunology, Institute of Child Health, University College London; J.B. Galloway, MBChB, MSc, CHP, FRCP, PhD, FAcadMedEd, Senior Lecturer and Honorary Consultant Rheumatologist, Department of Academic Rheumatology, King's College London, Weston Education Centre. ssubesinghe@nhs.net.

Bechman, Katie. From the Department of Academic Rheumatology, King's College London, Weston Education Centre; Institute of Child Health, University College London, London, UK.

Bechman, Katie. S. Subesinghe, BSc, MBBS, MSc, MRCP, Clinical Research Fellow and Rheumatology Registrar, Department of Academic Rheumatology, King's College London,

Weston Education Centre; K. Bechman, BSc, MBBS, MRCP, Clinical Research Fellow and Rheumatology Registrar, Department of Academic Rheumatology, King's College London, Weston Education Centre; A.I. Rutherford, BSc, MBBS, MSc, MRCP, Clinical Research Fellow and Rheumatology Registrar, Department of Academic Rheumatology, King's College London, Weston Education Centre; D. Goldblatt, MBChB, FRCPCH, FRCP, PhD, Prof, Vaccinology and Immunology, Institute of Child Health, University College London; J.B. Galloway, MBChB, MSc, CHP, FRCP, PhD, FAcadMedEd, Senior Lecturer and Honorary Consultant Rheumatologist, Department of Academic Rheumatology, King's College London, Weston Education Centre.

Rutherford, Andrew I. From the Department of Academic Rheumatology, King's College London, Weston Education Centre; Institute of Child Health, University College London, London, UK.

Rutherford, Andrew I. S. Subesinghe, BSc, MBBS, MSc, MRCP, Clinical Research Fellow and Rheumatology Registrar, Department of Academic Rheumatology, King's College London, Weston Education Centre; K. Bechman, BSc, MBBS, MRCP, Clinical Research Fellow and Rheumatology Registrar, Department of Academic Rheumatology, King's College London, Weston Education Centre; A.I. Rutherford, BSc, MBBS, MSc, MRCP, Clinical Research Fellow and Rheumatology Registrar, Department of Academic Rheumatology, King's College London, Weston Education Centre; D. Goldblatt, MBChB, FRCPCH, FRCP, PhD, Prof, Vaccinology and Immunology, Institute of Child Health, University College London; J.B. Galloway, MBChB, MSc, CHP, FRCP, PhD, FAcadMedEd, Senior Lecturer and Honorary Consultant Rheumatologist, Department of Academic Rheumatology, King's College London, Weston Education Centre.

Goldblatt, David. From the Department of Academic Rheumatology, King's College London, Weston Education Centre; Institute of Child Health, University College London, London, UK.

Goldblatt, David. S. Subesinghe, BSc, MBBS, MSc, MRCP, Clinical Research Fellow and Rheumatology Registrar, Department of Academic Rheumatology, King's College London, Weston Education Centre; K. Bechman, BSc, MBBS, MRCP, Clinical Research Fellow and Rheumatology Registrar, Department of Academic Rheumatology, King's College London, Weston Education Centre; A.I. Rutherford, BSc, MBBS, MSc, MRCP, Clinical Research Fellow and Rheumatology Registrar, Department of Academic Rheumatology, King's College London, Weston Education Centre; D. Goldblatt, MBChB, FRCPCH, FRCP, PhD, Prof, Vaccinology and Immunology, Institute of Child Health, University College London; J.B. Galloway, MBChB, MSc, CHP, FRCP, PhD, FAcadMedEd, Senior Lecturer and Honorary Consultant Rheumatologist, Department of Academic Rheumatology, King's College London, Weston Education Centre.

Galloway, James B. From the Department of Academic Rheumatology, King's College London, Weston Education Centre; Institute of Child Health, University College London, London, UK.

Galloway, James B. S. Subesinghe, BSc, MBBS, MSc, MRCP, Clinical Research Fellow and Rheumatology Registrar, Department of Academic Rheumatology, King's College London,

Weston Education Centre; K. Bechman, BSc, MBBS, MRCP, Clinical Research Fellow and Rheumatology Registrar, Department of Academic Rheumatology, King's College London, Weston Education Centre; A.I. Rutherford, BSc, MBBS, MSc, MRCP, Clinical Research Fellow and Rheumatology Registrar, Department of Academic Rheumatology, King's College London, Weston Education Centre; D. Goldblatt, MBChB, FRCPCH, FRCP, PhD, Prof, Vaccinology and Immunology, Institute of Child Health, University College London; J.B. Galloway, MBChB, MSc, CHP, FRCP, PhD, FAcadMedEd, Senior Lecturer and Honorary Consultant Rheumatologist, Department of Academic Rheumatology, King's College London, Weston Education Centre.

Abstract

OBJECTIVE: Vaccination is a key strategy to reduce infection risk in patients with rheumatoid arthritis (RA) and is advocated in internationally recognized rheumatology society guidelines. The aim was to evaluate to the effect of antirheumatic drugs on influenza and pneumococcal vaccine immunogenicity.

METHODS: We conducted a systematic literature review and metaanalysis comparing the humoral response to influenza (pandemic and seasonal trivalent subunit vaccines) and pneumococcal (23-valent pneumococcal polysaccharide vaccine, 7- and 13-valent pneumococcal conjugated vaccines) vaccination in adult patients with RA treated with antirheumatic drugs. Vaccine immunogenicity was assessed by seroprotection rates measured 3 to 6 weeks postimmunization. Risk ratios (RR) and 95% CI were pooled.

RESULTS: Nine studies were included in the metaanalysis (7 studies investigating antirheumatic drug exposures and influenza humoral response, 2 studies investigating pneumococcal vaccine response). Influenza vaccine responses to all subunit strains (H1N1, H3N2, B strain) were preserved with methotrexate (MTX) and tumor necrosis factor inhibitor (TNFi) drug exposure. MTX but not TNFi drug exposure was associated with reduced 6B and 23F serotype pneumococcal vaccine response (RR 0.42, 95% CI 0.28-0.63 vs RR 0.98, 95% CI 0.58-1.67); however, limited data were available to draw any firm conclusions. Combination of MTX with tocilizumab or tofacitinib was associated with reduced pneumococcal and influenza vaccine responses.

CONCLUSION: Antirheumatic drugs may limit humoral responses to vaccination as evidenced by pneumococcal responses with MTX exposure; however, they are safe and should not preclude immunization against vaccine-preventable disease. Vaccination should be considered in all patients with RA and encouraged as part of routine care. (Systematic review registration number: PROSPERO 2016: CRD42016048093.).

Publication Type

Journal Article. Meta-Analysis. Research Support, Non-U.S. Gov't. Systematic Review.
Year of Publication
2018

<117>
Unique Identifier
29529488
Title
Amelioration of collagen antibody induced arthritis in mice by an antibody directed against the fibronectin type III repeats of tenascin-C: Targeting fibronectin type III repeats of tenascin-C in rheumatoid arthritis.
Source
International Immunopharmacology. 58:15-23, 2018 May.
VI 1
Status
MEDLINE
Authors
Mehta BB; Tiwari A; Sharma S; Shukla A; Sharma M; Vasishta RK; Sen RK; Sharma A; Luthra-Guptasarma M.
Authors Full Name
Mehta, Brij Bhushan; Tiwari, Anil; Sharma, Saniya; Shukla, Ashu; Sharma, Maryada; Vasishta, Rakesh K; Sen, Ramesh K; Sharma, Aman; Luthra-Guptasarma, Manni.
Institution
Mehta, Brij Bhushan. Department of Immunopathology, Postgraduate Institute of Medical Education and Research, Chandigarh, 160012, India.
Tiwari, Anil. Department of Immunopathology, Postgraduate Institute of Medical Education and Research, Chandigarh, 160012, India.
Sharma, Saniya. Department of Histopathology, Postgraduate Institute of Medical Education and Research, Chandigarh, 160012, India.
Shukla, Ashu. Department of Immunopathology, Postgraduate Institute of Medical Education and Research, Chandigarh, 160012, India.
Sharma, Maryada. Department of Immunopathology, Postgraduate Institute of Medical Education and Research, Chandigarh, 160012, India.

Vasishta, Rakesh K. Department of Histopathology, Postgraduate Institute of Medical Education and Research, Chandigarh, 160012, India.
Sen, Ramesh K. Department of Orthopaedics, Postgraduate Institute of Medical Education and Research, Chandigarh, 160012, India.
Sharma, Aman. Department of Internal Medicine, Postgraduate Institute of Medical Education and Research, Chandigarh, 160012, India.
Luthra-Guptasarma, Manni. Department of Immunopathology, Postgraduate Institute of Medical Education and Research, Chandigarh, 160012, India. Electronic address: guptasarma.manni@pgimer.edu.in.
Abstract
Tenascin-C (TN-C) levels are elevated in the synovial tissue and fluid, as well as cartilage of rheumatoid arthritis (RA) patients. In addition, the presence of TN-C fragments has also been documented in arthritic cartilage. We have previously shown that a single chain variable fragment antibody (TN64), directed against the fibronectin type III repeats 1-5 (TNfnIII 1-5) of TN-C, effectively inhibits fibrotic pathology. Given that fibrosis results from chronic inflammation, and the fact that increased levels of TN-C in the synovial fluid of patients with RA contributes to synovial inflammation and joint destruction, we aimed to investigate the role of TNfnIII 1-5 region of TN-C in RA pathogenesis. Using either the wild type or variants of the two integrin-binding motifs (RGD and AEIDGIEL) present within the TNfnIII 1-5 polypeptide, we demonstrate that the adhesion and migration of synovial fibroblasts is RGD-dependent. The antibody TN64 is effective in inhibiting migration of cells in response to TnfnIII 1-5, and prevents fibroblast-mediated destruction of cartilage. The TN64 antibody was further tested in collagen antibody induced arthritic (CAIA) mice. Our data shows the efficacy of TN64 in preventing induction of arthritis, with significant downregulation of RA-associated cytokines. This suggests that components of the extracellular matrix such as the TNfnIII 1-5 region of TN-C could be exploited to develop therapies to suppress inflammation seen in RA. The TN64 antibody is one such promising candidate in the development of novel treatments for RA. Copyright © 2018 Elsevier B.V. All rights reserved.
Publication Type
Journal Article.
Year of Publication
2018

<118>

Unique Identifier

29504436

Title

Tocilizumab in the treatment of giant cell arteritis. [Review]

Source

Immunotherapy. 10(6):465-472, 2018 03 01.

VI 1

Status

MEDLINE

Authors

Leuchten N; Aringer M.

Authors Full Name

Leuchten, Nicolai; Aringer, Martin.

Institution

Leuchten, Nicolai. Division of Rheumatology, Department of Medicine III, University Medical Center & Faculty of Medicine Carl Gustav Carus at the TU Dresden, Dresden, Germany.

Aringer, Martin. Division of Rheumatology, Department of Medicine III, University Medical Center & Faculty of Medicine Carl Gustav Carus at the TU Dresden, Dresden, Germany.

Abstract

Giant cell arteritis is a systemic vasculitis of large vessels, manifesting mainly as temporal arteritis or large vessel vasculitis of the aorta and its branches. Glucocorticoid therapy is essential and so far had to be continued over a period of 1.5-2 years, resulting in relevant morbidity through adverse effects. With the approval of tocilizumab, an effective glucocorticoid sparing option is now available. In two randomized controlled trials, a profound reduction of cumulative glucocorticoid dose, prolonged relapse-free remission and reduced number of adverse events in the treatment groups have been demonstrated. Therefore, tocilizumab constitutes a novel therapeutic option in giant cell arteritis. Its differential role in different subgroups, timing of tocilizumab therapy and optimal treatment duration remain to be determined.

Publication Type

Journal Article. Review.

Year of Publication

2018

<119>

Unique Identifier

29499726

Title

Varicella-zoster-virus vaccination in immunosuppressed children with rheumatic diseases using a pre-vaccination check list.

Source

Pediatric Rheumatology Online Journal. 16(1):15, 2018 Mar 02.

VI 1

Status

MEDLINE

Authors

Speth F; Hinze CH; Andel S; Mertens T; Haas JP.

Authors Full Name

Speth, Fabian; Hinze, Claas H; Andel, Susanne; Mertens, Thomas; Haas, Johannes-Peter.

Institution

Speth, Fabian. German Center for Pediatric and Adolescent Rheumatology, Gehfeldstr. 24, 82467, Garmisch-Partenkirchen, Germany.

Hinze, Claas H. Department of Pediatric Rheumatology and Immunology, University Hospital Munster, Albert-Schweitzer-Campus I, Building W30, 48149, Munster, Germany. claas.hinze@ukmuenster.de.

Andel, Susanne. German Center for Pediatric and Adolescent Rheumatology, Gehfeldstr. 24, 82467, Garmisch-Partenkirchen, Germany.

Mertens, Thomas. Institute of Virology, Ulm University Medical Center, Albert-Einstein-Allee 11, 89081, Ulm, Germany.

Haas, Johannes-Peter. German Center for Pediatric and Adolescent Rheumatology, Gehfeldstr. 24, 82467, Garmisch-Partenkirchen, Germany.

Abstract

BACKGROUND: The goal of this study was to apply the varicella zoster virus (VZV) vaccine to patients with pediatric rheumatic diseases (PRD) at risk for severe chickenpox, without interrupting their current immunosuppression, including biological agents, using an immunological-based pre-vaccination checklist to assure safety. A pre-vaccination checklist was implemented to ensure adequate immune competence prior to immunization.

METHODS: This prospective study included seronegative patients (VZV-IgG <=200 mIU/ml) and patients who had previously received only a single dose of VZV vaccine. All vaccinees demonstrated clinically inactive PRD. Patients were categorized according to their actual

treatment in low-intensity IS (LIIS) and high-intensity IS (HIIS) including biological therapy. The pre-vaccination checklist defined thresholds for the following basic laboratory tests: white blood cell count >=3000/mm3, lymphocytes >=1200/mm3, serum IgG >=500 mg/dl, IgM >=20 mg/dl, tetanus toxoid antibody >=0.1 IU/ml. In case of HIIS additional specifications included a CD4+ lymphocyte count >=200/mm3 and a positive T-cell function (via analyzable positive control of a standard tuberculosis interferon-gamma-release-assay (TB-IGRA) indicating mitogen-induced T cell proliferation). Patients who met the criteria of the pre-vaccination checklist received the first and/or second VZV vaccination. Immunologic response and side effects were monitored.

RESULTS: Twenty-three patients were recruited of whom nine had already received one VZV immunization before initiating IS. All patients met the pre-vaccination checklist criteria despite ongoing IS. There was no overall difference in VZV-IgG levels when comparing the LIIS (n=9) and HIIS (n=14) groups. In total, 21 patients (91%) showed a positive vaccination response, after the first immunization the median VZV-IgG across all patients was 224 (59-1219) mIU/ml (median (range)), after booster immunization it increased to 882 (30-4685) mIU/ml. Two patients in the HIIS group failed to raise positive VZV-IgG, despite booster immunization. All nine patients receiving only the second immunization on IS reached high titers of VZV-IgG >500 mIU/ml (1117 (513-4685) mIU/ml). There were no cases of rash or other vaccine-induced varicella disease symptoms and no evidence of PRD flare.

CONCLUSIONS: VZV vaccination is safe and largely immunogenic in children with ongoing IS fulfilling an immunological based pre-vaccination checklist. This new approach is based on immunologic function rather than on type of medications.

TRIAL REGISTRATION NUMBER: ISRCRTN trial registration number 21654693 , date of registration February 12, 2018, retrospectively registered.

Publication Type

Clinical Trial. Journal Article.

Year of Publication

2018

<120>

Unique Identifier

29495891

Title

Tocilizumab in the treatment of adult rheumatoid arthritis. [Review]

Source

Immunotherapy. 10(6):447-464, 2018 03 01.

VI 1

Status

MEDLINE

Authors

Sanmarti R; Ruiz-Esquide V; Bastida C; Soy D.

Authors Full Name

Sanmarti, Raimon; Ruiz-Esquide, Virginia; Bastida, Carla; Soy, Dolor.

Institution

Sanmarti, Raimon. Arthritis Unit, Rheumatology Service, Hospital Clinic of Barcelona, Barcelona, Catalonia, Spain.

Ruiz-Esquide, Virginia. Arthritis Unit, Rheumatology Service, Hospital Clinic of Barcelona, Barcelona, Catalonia, Spain.

Bastida, Carla. Pharmacy Service, Hospital Clinic of Barcelona, Barcelona, Catalonia, Spain.

Soy, Dolor. Pharmacy Service, Hospital Clinic of Barcelona, Barcelona, Catalonia, Spain.

Abstract

Rheumatoid arthritis (RA) is the most prevalent immune-mediated chronic rheumatic disease and is associated with joint destruction and disability. Therapeutic strategies, including biological disease-modifying antirheumatic drugs (bDMARDs) have improved the prognosis and quality of life of RA patients. Tocilizumab (TCZ) is a humanized monoclonal antibody against IL-6 receptor licensed in 2009 that has demonstrated clinical efficacy in various adult RA populations. RA management guidelines and recommendations consider TCZ as one of the bDMARDS indicated after methotrexate or other conventional synthetic DMARDs and/or TNF inhibitors failure in adult RA. Of particular interest is the demonstration of its effectiveness in monotherapy in comparison with other bDMARDs. Recent observational studies have shown good results for the safety profile of TCZ with no new alert signals.

Publication Type

Journal Article. Review.

Year of Publication

2018

<121>

Unique Identifier

29489833

Title

Immune checkpoint inhibitor PD-1 pathway is down-regulated in synovium at various stages of rheumatoid arthritis disease progression.

Source

PLoS ONE [Electronic Resource]. 13(2):e0192704, 2018.

VI 1

Status

MEDLINE

Authors

Guo Y; Walsh AM; Canavan M; Wechalekar MD; Cole S; Yin X; Scott B; Loza M; Orr C; McGarry T; Bombardieri M; Humby F; Proudman SM; Pitzalis C; Smith MD; Friedman JR; Anderson I; Madakamutil L; Veale DJ; Fearon U; Nagpal S.

Author NameID

Nagpal, Sunil; ORCID: https://orcid.org/0000-0001-6090-8780

Authors Full Name

Guo, Yanxia; Walsh, Alice M; Canavan, Mary; Wechalekar, Mihir D; Cole, Suzanne; Yin, Xuefeng; Scott, Brittney; Loza, Mathew; Orr, Carl; McGarry, Trudy; Bombardieri, Michele; Humby, Frances; Proudman, Susanna M; Pitzalis, Costantino; Smith, Malcolm D; Friedman, Joshua R; Anderson, Ian; Madakamutil, Loui; Veale, Douglas J; Fearon, Ursula; Nagpal, Sunil.

Institution

Guo, Yanxia. Immunology, Janssen Research, Pennsylvania, United States of America.

Walsh, Alice M. Immunology, Janssen Research, Pennsylvania, United States of America.

Canavan, Mary. Molecular Rheumatology, Trinity Biomedical Sciences Institute, Trinity College, Dublin, Ireland.

Wechalekar, Mihir D. Rheumatology Unit, Repatriation General Hospital and Flinders University, Adelaide, Australia.

Cole, Suzanne. Immunology, Janssen Research, Pennsylvania, United States of America.

Yin, Xuefeng. Immunology, Janssen Research, Pennsylvania, United States of America.

Scott, Brittney. Immunology, Janssen Research, Pennsylvania, United States of America.

Loza, Mathew. Immunology, Janssen Research, Pennsylvania, United States of America.

Orr, Carl. Centre for Arthritis and Rheumatic Diseases, St. Vincent's University Hospital, University College Dublin, Elm Park, Dublin, Ireland.

McGarry, Trudy. Molecular Rheumatology, Trinity Biomedical Sciences Institute, Trinity College, Dublin, Ireland.

Bombardieri, Michele. Queen Mary University of London, Charterhouse Square, London, United Kingdom.

Humby, Frances. Queen Mary University of London, Charterhouse Square, London, United Kingdom.

Proudman, Susanna M. Rheumatology Unit, Royal Adelaide Hospital, Adelaide, Australia and Discipline of Medicine, University of Adelaide, Adelaide, Australia.

Pitzalis, Costantino. Queen Mary University of London, Charterhouse Square, London, United Kingdom.

Smith, Malcolm D. Rheumatology Unit, Repatriation General Hospital and Flinders University, Adelaide, Australia.

Friedman, Joshua R. Immunology, Janssen Research, Pennsylvania, United States of America.

Anderson, Ian. Immunology, Janssen Research, Pennsylvania, United States of America.

Madakamutil, Loui. Immunology, Janssen Research, Pennsylvania, United States of America.

Veale, Douglas J. Centre for Arthritis and Rheumatic Diseases, St. Vincent's University Hospital, University College Dublin, Elm Park, Dublin, Ireland.

Fearon, Ursula. Molecular Rheumatology, Trinity Biomedical Sciences Institute, Trinity College, Dublin, Ireland.

Nagpal, Sunil. Immunology, Janssen Research, Pennsylvania, United States of America.

Abstract

Immune checkpoint blockade with therapeutic anti-cytotoxic T lymphocyte-associated antigen (CTLA)-4 (Ipilimumab) and anti-programmed death (PD)-1 (Nivolumab and Pembrolizumab) antibodies alone or in combination has shown remarkable efficacy in multiple cancer types, concomitant with immune-related adverse events, including arthralgia and inflammatory arthritis (IA) in some patients. Herein, using Nivolumab (anti-PD-1 antagonist)-responsive genes along with transcriptomics of synovial tissue from multiple stages of rheumatoid arthritis (RA) disease progression, we have interrogated the activity status of PD-1 pathway during RA development. We demonstrate that the expression of PD-1 was increased in early and established RA synovial tissue compared to normal and OA synovium, whereas that of its ligands, programmed death ligand-1 (PD-L1) and PD-L2, was increased at all the stages of RA disease progression, namely arthralgia, IA/undifferentiated arthritis, early RA and established RA. Further, we show that RA patients expressed PD-1 on a majority of synovial tissue infiltrating CD4+ and CD8+ T cells. Moreover, enrichment of Nivolumab gene signature was observed in IA and RA, indicating that the PD-1 pathway was downregulated during RA disease progression. Furthermore, serum soluble (s) PD-1 levels were increased in autoantibody positive early RA patients. Interestingly, most of the early RA synovium tissue sections showed negative PD-L1 staining by

immunohistochemistry. Therefore, downregulation in PD-1 inhibitory signaling in RA could be attributed to increased serum sPD-1 and decreased synovial tissue PD-L1 levels. Taken together, these data suggest that agonistic PD1 antibody-based therapeutics may show efficacy in RA treatment and interception.

Publication Type

Journal Article. Research Support, Non-U.S. Gov't.

Year of Publication

2018

<122>

Unique Identifier

29476557

Title

Expression of interleukin-17 in primary Sjogren's syndrome and the correlation with disease severity: A systematic review and meta-analysis. [Review]

Source

Scandinavian Journal of Immunology. 87(4):e12649, 2018 Apr.

VI 1

Status

MEDLINE

Authors

Zhang LW; Zhou PR; Wei P; Cong X; Wu LL; Hua H.

Author NameID

Zhang, L-W; ORCID: http://orcid.org/0000-0003-4762-7989

Authors Full Name

Zhang, L-W; Zhou, P-R; Wei, P; Cong, X; Wu, L-L; Hua, H.

Institution

Zhang, L-W. Department of Oral Medicine, Peking University School and Hospital of Stomatology, Beijing, China.

Zhou, P-R. Department of Oral Medicine, Peking University School and Hospital of Stomatology, Beijing, China.

Wei, P. Department of Oral Medicine, Peking University School and Hospital of Stomatology, Beijing, China.

Cong, X. Department of Physiology and Pathophysiology, Peking University Health Science Center, Key Laboratory of Molecular Cardiovascular Sciences, Ministry of Education, and Beijing Key Laboratory of Cardiovascular Receptors Research, Beijing, China.
Wu, L-L. Department of Physiology and Pathophysiology, Peking University Health Science Center, Key Laboratory of Molecular Cardiovascular Sciences, Ministry of Education, and Beijing Key Laboratory of Cardiovascular Receptors Research, Beijing, China.
Hua, H. Department of Oral Medicine, Peking University School and Hospital of Stomatology, Beijing, China.
Abstract
The aberrant expression of interleukin-17 (IL-17) has been reported in primary Sjogren's syndrome (pSS). Abnormalities in IL-17 can promote the production of pro-inflammatory cytokines and aggravate autoimmune disorders. The aim of this study was to investigate alterations of IL-17 in patients with pSS and explore the correlation between IL-17 and disease severity. Eight databases were searched for original studies reporting the expression of IL-17 in patients with pSS and controls. Eligible reports were included in the pooled analysis, and subgroup evaluations were performed according to different types of controls and IL-17 measurement methods. Newcastle-Ottawa Scale criteria were used to assess the risk of bias of the included studies. In total, 45 articles are included in the meta-analysis. The expression of IL-17 is significantly increased in patients with pSS compared to controls. Furthermore, patients with pSS without immunosuppressive treatment show markedly higher IL-17 levels. In addition, patients with pSS with positive rheumatoid factors tend to express a higher level of IL-17 than patients with negative rheumatoid factors. Negative correlations between IL-17 levels and ocular parameters are also found in patients with pSS. The results are similar after adjustment by "trim and fill" methods. In conclusion, the expression of IL-17 is obviously increased in patients with pSS, especially among those without immunosuppressive treatment. In addition, IL-17 level correlates with the disease severity of pSS. These findings demonstrate the significance of IL-17 overexpression in patients with pSS and may provide insights for the development of therapeutic interventions targeting IL-17 for pSS. Copyright © 2018 The Foundation for the Scandinavian Journal of Immunology.
Publication Type
Journal Article. Meta-Analysis. Review. Systematic Review.
Year of Publication
2018

<123>
Unique Identifier
29465352
Title
Immune response against the coiled coil domain of Sjogren's syndrome associated autoantigen Ro52 induces salivary gland dysfunction.
Source
Clinical & Experimental Rheumatology. 36 Suppl 112(3):41-46, 2018 May-Jun.
VI 1
Status
MEDLINE
Authors
Sroka M; Bagavant H; Biswas I; Ballard A; Deshmukh US.
Authors Full Name
Sroka, Magdalena; Bagavant, Harini; Biswas, Indranil; Ballard, Abigail; Deshmukh, Umesh S.
Institution
Sroka, Magdalena. Arthritis and Clinical Immunology Program, Oklahoma Medical Research Foundation, Oklahoma City, USA.
Bagavant, Harini. Arthritis and Clinical Immunology Program, Oklahoma Medical Research Foundation, Oklahoma City, USA.
Biswas, Indranil. Arthritis and Clinical Immunology Program, Oklahoma Medical Research Foundation, Oklahoma City, USA.
Ballard, Abigail. Arthritis and Clinical Immunology Program, Oklahoma Medical Research Foundation, Oklahoma City, USA.
Deshmukh, Umesh S. Arthritis and Clinical Immunology Program, Oklahoma Medical Research Foundation, Oklahoma City, USA. umesh-deshmukh@omrf.org.
Abstract
OBJECTIVES: The structural domains of Ro52, termed the RING, B-box, coiled coil (CC) and B30.2/SPRY are targets of anti-Ro52 in multiple autoimmune disorders. In Sjogren's syndrome patients, the presence of anti-Ro52 is associated with higher disease severity, and in mice, they induce salivary gland hypofunction. This study was undertaken to investigate whether immune responses against different domains of Ro52, influences salivary gland disease in mice.

METHODS: Female NZM2758 mice were immunised with Ro52 domains expressed as recombinant fusion proteins with maltose binding protein (MBP) [MBP-RING-B-box, MBP-CC, MBP-CC(DELTAC19), MBP-B30.2/SPRY]. Sera from immunised mice were studied for IgG

antibodies to Ro52 by immunoprecipitation, and to salivary gland cells by immunofluorescence. Pilocarpine-induced saliva production was measured to evaluate salivary gland function. Submandibular glands were investigated by histopathology for inflammation and by immune-histochemistry for IgG deposition.

RESULTS: Mice immunised with different Ro52-domains had comparable reactivity to Ro52 and to salivary gland cells. However, only mice immunised with the CC domain and its C-terminal truncated version CC(DELTAC19) showed a significant drop in saliva production. None of the mice developed severe salivary gland inflammation. The salivary gland hypofunction significantly correlated with increased intra-lobar IgG deposits in the submandibular salivary glands.

CONCLUSIONS: Our data demonstrate that epitope specificity of anti-Ro52 antibodies plays a critical role in the induction of glandular dysfunction. Clearly, screening Sjogren's syndrome patients for relative levels of Ro52 domain specific antibodies will be more informative for associating anti-Ro52 with clinical measures of the disorder.

Publication Type

Comparative Study. Journal Article.

Year of Publication

2018

<124>

Unique Identifier

29432051

Title

Immunogenicity and persistence of a prime-boost re-vaccination strategy for pneumococcal vaccines in patients with rheumatoid arthritis.

Source

Human vaccines & Immunotherapeutics. 14(6):1464-1470, 2018 06 03.

VI 1

Status

MEDLINE

Authors

Bahuaud M; Beaudouin-Bazire C; Husson M; Molto A; Launay O; Batteux F; Dougados M.

Author NameID
Bahuaud, Mathilde; ORCID: https://orcid.org/0000-0001-6454-0370
Authors Full Name
Bahuaud, Mathilde; Beaudouin-Bazire, Constance; Husson, Marine; Molto, Anna; Launay, Odile; Batteux, Frederic; Dougados, Maxime.
Institution
Bahuaud, Mathilde. a Plateforme d'Immunomonotoring Vaccinal, Laboratoire d'Immunologie, Groupe hospitalier Cochin-Broca-Hotel Dieu, AP-HP, Paris, France.
Beaudouin-Bazire, Constance. b Service de Rhumatologie B, Groupe hospitalier Cochin-Broca-Hotel Dieu, APHP, Paris, France.
Husson, Marine. a Plateforme d'Immunomonotoring Vaccinal, Laboratoire d'Immunologie, Groupe hospitalier Cochin-Broca-Hotel Dieu, AP-HP, Paris, France.
Molto, Anna. b Service de Rhumatologie B, Groupe hospitalier Cochin-Broca-Hotel Dieu, APHP, Paris, France.
Launay, Odile. c Centre d'Investigation Clinique Cochin-Pasteur (CIC1417), Groupe hospitalier Cochin-Broca-Hotel Dieu, AP-HP, Paris, France.
Batteux, Frederic. a Plateforme d'Immunomonotoring Vaccinal, Laboratoire d'Immunologie, Groupe hospitalier Cochin-Broca-Hotel Dieu, AP-HP, Paris, France.
Dougados, Maxime. b Service de Rhumatologie B, Groupe hospitalier Cochin-Broca-Hotel Dieu, APHP, Paris, France.
Abstract
OBJECTIVES: Patients with rheumatoid arthritis (RA) are at an increased risk of Pneumococcal infections. Immunogenicity and persistence of a prime-boost revaccination strategy using 13-valent/23-valent anti-pneumococcal vaccines was evaluated in patients with RA treated by Methotrexate (MTX) and anti-TNF.

METHOD: Twenty-four patients with RA received one dose of PCV13 (Prevenar13 R; Pfizer) followed two months later by one dose of PPV23 (Pneumovax R, Merck). Concentrations of IgG specific for 7 serotypes common to both vaccines and 3 uncommon serotypes, included only in the PPV23 were measured by ELISA and Opsonophagocytic Assay (OPA) at baseline and after 4, 12 and 24 months post-vaccine.

RESULTS: Similar percentages of protection were found at 4 months (63% vs. 55%), 12 months (54% vs. 50%) and 24 months (52% vs. 55%) for the 7 common and 3 uncommon serotypes when antibody titers were assayed by ELISA. Based on functional antibody measurements by OPA, a decrease of protected patients was observed 24 months after vaccine with only 19% of patients protected compared to 29% at baseline.

CONCLUSION: Although the combined pneumococcal revaccination strategy induces good protection in the short term in RA patients, this protection does not persist beyond two years with levels of functional antibody decreasing below pre-vaccine levels. We did not observe a higher efficacy of the conjugate vaccine compared to the polysaccharide vaccine. Our results clearly question the advantage of the prime-boost strategy as it highlight the possible hyporesponse induced by PPV23 against the immune response elicited by the primo-injection of the PCV13 vaccine.

Publication Type

Journal Article.

Year of Publication

2018

<125>

Unique Identifier

29373844

Title

Citrullinated fibrinogen impairs immunomodulatory function of bone marrow mesenchymal stem cells by triggering toll-like receptor.

Source

Clinical Immunology. 193:38-45, 2018 08.

VI 1

Status

MEDLINE

Authors

Sun Y; Deng W; Yao G; Chen W; Tang X; Feng X; Lu L; Sun L.

Authors Full Name

Sun, Yue; Deng, Wei; Yao, Genhong; Chen, Weiwei; Tang, Xiaojun; Feng, Xuebing; Lu, Liwei; Sun, Lingyun.

Institution

Sun, Yue. Department of Rheumatology and Immunology, The Affiliated Drum Tower Hospital of Nanjing University Medical School, Nanjing 210008, China.

Deng, Wei. Department of Rheumatology and Immunology, The Affiliated Drum Tower Hospital of Nanjing University Medical School, Nanjing 210008, China.
Yao, Genhong. Department of Rheumatology and Immunology, The Affiliated Drum Tower Hospital of Nanjing University Medical School, Nanjing 210008, China.
Chen, Weiwei. Department of Rheumatology and Immunology, The Affiliated Drum Tower Hospital of Nanjing University Medical School, Nanjing 210008, China.
Tang, Xiaojun. Department of Rheumatology and Immunology, The Affiliated Drum Tower Hospital of Nanjing University Medical School, Nanjing 210008, China.
Feng, Xuebing. Department of Rheumatology and Immunology, The Affiliated Drum Tower Hospital of Nanjing University Medical School, Nanjing 210008, China.
Lu, Liwei. Department of Pathology, Center of Infection and Immunology, University of Hong Kong, Hong Kong 999077, China.
Sun, Lingyun. Department of Rheumatology and Immunology, The Affiliated Drum Tower Hospital of Nanjing University Medical School, Nanjing 210008, China. Electronic address: lingyunsun@nju.edu.cn.
Abstract
Bone marrow mesenchymal stem cells (BMSC) have been shown to possess immunomodulatory activities, while its role in rheumatoid arthritis (RA) remains unknown. Citrullinated fibrinogen (cfb) has been considered as a specific autoantigen in RA pathogenesis. Our study aims to determine the role of cfb on immunomodulatory function of BMSC. We demonstrated the specific role of toll-like receptor 4 (TLR4)-NFkappaB pathway in the pro-inflammatory response of BMSC to cfb with increased production of interleukin (IL)-6, IL-8 and chemokine CC motif ligand 2 (CCL2). Moreover, cfb impaired BMSC-mediated suppression of peripheral blood mononuclear cells (PBMC) proliferation and reduced the production of the key immunomodulatory molecule indoleamine 2,3-dioxygenase (IDO) in BMSC. We have uncovered a previously unrecognized role of cfb in interfering BMSC-mediated immunoregulation in RA. Cfb could act as a damage-associated molecule pattern (DAMP) for BMSC and thereby contribute to the propagation of inflammation in RA. Copyright © 2018 Elsevier Inc. All rights reserved.
Publication Type
Journal Article. Research Support, Non-U.S. Gov't.
Year of Publication
2018

<126>

Unique Identifier

29363290

Title

Brief Report: Cancer Immunotherapy in Patients With Preexisting Rheumatic Disease: The Mayo Clinic Experience. Arthritis & Rheumatology. 70(3):356-360, 2018 03.

Authors

Richter MD; Pinkston O; Kottschade LA; Finnes HD; Markovic SN; Thanarajasingam U.

Authors Full Name

Richter, Michael D; Pinkston, Olga; Kottschade, Lisa A; Finnes, Heidi D; Markovic, Svetomir N; Thanarajasingam, Uma.

Institution

Richter, Michael D. Mayo School of Graduate Medical Education, Rochester, Minnesota.

Pinkston, Olga. Mayo Clinic, Jacksonville, Florida.

Kottschade, Lisa A. Mayo Clinic College of Medicine, Rochester, Minnesota.

Finnes, Heidi D. Mayo Clinic, Jacksonville, Florida, and Mayo Clinic College of Medicine, Rochester, Minnesota.

Markovic, Svetomir N. Mayo Clinic College of Medicine, Rochester, Minnesota.

Thanarajasingam, Uma. Mayo Clinic College of Medicine, Rochester, Minnesota.

Abstract

OBJECTIVE: To determine the risk of rheumatic disease flare and adverse effects in patients with preexisting rheumatic disease who were receiving immune checkpoint inhibitor (ICI) therapy.

METHODS: A retrospective medical record review was performed to identify all patients who received ICI therapy at Mayo Clinic in Rochester, Minnesota between 2011 and 2016 (~700 patients). Those with a preexisting rheumatic disease were identified using specific diagnostic codes.

RESULTS: Sixteen patients were identified (81% female, median age 68.5 years). The most common rheumatic diseases were rheumatoid arthritis (n = 5), polymyalgia rheumatica (n = 5), Sjogren's syndrome (n = 2), and systemic lupus erythematosus (n = 2). Seven patients were receiving immunosuppressive therapy or glucocorticoids for their rheumatic disease at the time of initiation of the ICI. The primary malignancies were melanoma (n = 10), pulmonary (n = 4), or hematologic (n = 2). In most cases, ICIs were offered only after failure of several other therapies. Immune-related adverse effects (IRAEs) occurred in 6 patients, and all were treated successfully with glucocorticoids and discontinuation of the ICI therapy. There were no significant differences

in time from cancer diagnosis to immunotherapy, duration of immunotherapy, age, or sex between the patients with and those without IRAEs.

CONCLUSION: To our knowledge, this represents the largest single-center cohort of patients with rheumatic diseases who were exposed to modern cancer immunotherapy. Only a minority of these patients experienced a flare of their preexisting rheumatic disease or any other IRAE.
Copyright © 2017, American College of Rheumatology.
Publication Type
Journal Article.
Year of Publication
2018

<127>
Unique Identifier
29361749
Title
Affinity Purification and Comparative Biosensor Analysis of Citrulline-Peptide-Specific Antibodies in Rheumatoid Arthritis.
Source
International Journal of Molecular Sciences. 19(1), 2018 Jan 22.
VI 1
Status
MEDLINE
Authors
Szarka E; Aradi P; Huber K; Pozsgay J; Vegh L; Magyar A; Gyulai G; Nagy G; Rojkovich B; Kiss E; Hudecz F; Sarmay G.
Author NameID
Szarka, Eszter; ORCID: https://orcid.org/0000-0003-4756-1502
Magyar, Anna; ORCID: https://orcid.org/0000-0003-1390-6496
Sarmay, Gabriella; ORCID: https://orcid.org/0000-0002-3066-7768
Authors Full Name
Szarka, Eszter; Aradi, Petra; Huber, Krisztina; Pozsgay, Judit; Vegh, Lili; Magyar, Anna; Gyulai, Gergo; Nagy, Gyorgy; Rojkovich, Bernadette; Kiss, Eva; Hudecz, Ferenc; Sarmay, Gabriella.

Institution
Szarka, Eszter. Department of Immunology, Eotvos Lorand University, 1117 Budapest, Hungary. szarka.eszter@ttk.elte.hu.
Aradi, Petra. Department of Immunology, Eotvos Lorand University, 1117 Budapest, Hungary. aradi.petra@gmail.com.
Huber, Krisztina. Department of Immunology, Eotvos Lorand University, 1117 Budapest, Hungary. krisztina.huber@gmail.com.
Pozsgay, Judit. Department of Immunology, Eotvos Lorand University, 1117 Budapest, Hungary. pozsgayj@gmail.com.
Vegh, Lili. Department of Immunology, Eotvos Lorand University, 1117 Budapest, Hungary. lilivegh92@gmail.com.
Magyar, Anna. MTA-ELTE Research Group of Peptide Chemistry, Hungarian Academy of Science, Eotvos Lorand University, 1117 Budapest, Hungary. magyar@elte.hu.
Gyulai, Gergo. Laboratory of Interfaces and Nanostructures, Institute of Chemistry, Eotvos Lorand University, 1117 Budapest, Hungary. gyulaigor@gmail.com.
Nagy, Gyorgy. Department of Rheumatology, 3rd Department of Internal Medicine, Semmelweis University, 1125 Budapest, Hungary. gyorgyngy@gmail.com.
Nagy, Gyorgy. Rheumatology, Buda Hospital of the Hospitaller Order of St. John of God, 1023 Budapest, Hungary. gyorgyngy@gmail.com.
Rojkovich, Bernadette. Rheumatology, Buda Hospital of the Hospitaller Order of St. John of God, 1023 Budapest, Hungary. rojkovich.b@gmail.com.
Kiss, Eva. Laboratory of Interfaces and Nanostructures, Institute of Chemistry, Eotvos Lorand University, 1117 Budapest, Hungary.
Hudecz, Ferenc. MTA-ELTE Research Group of Peptide Chemistry, Hungarian Academy of Science, Eotvos Lorand University, 1117 Budapest, Hungary. fhudecz@caesar.elte.hu.
Sarmay, Gabriella. Department of Immunology, Eotvos Lorand University, 1117 Budapest, Hungary. sarmayg@ttk.elte.hu.
Abstract
BACKGROUND: In rheumatoid arthritis (RA), anti-citrullinated protein/peptide antibodies (ACPAs) are responsible for disease onset and progression, however, our knowledge is limited on ligand binding affinities of autoantibodies with different citrulline-peptide specificity.

METHODS: Citrulline-peptide-specific ACPA IgGs were affinity purified and tested by ELISA. Binding affinities of ACPA IgGs and serum antibodies were compared by surface plasmon resonance (SPR) analysis. Bifunctional nanoparticles harboring a multi-epitope citrulline-peptide and a complement-activating peptide were used to induce selective depletion of ACPA-producing B cells.

RESULTS: KD values of affinity-purified ACPA IgGs varied between 10-6 and 10-8 M and inversely correlated with disease activity. Based on their cross-reaction with citrulline-peptides, we designed a novel multi-epitope peptide, containing Cit-Gly and Ala-Cit motifs in two-two copies, separated with a short, neutral spacer. This peptide detected antibodies in RA sera with 66% sensitivity and 98% specificity in ELISA and was recognized by 90% of RA sera, while none of the healthy samples in SPR. When coupled to nanoparticles, the multi-epitope peptide specifically targeted and depleted ACPA-producing B cells ex vivo.

CONCLUSIONS: The unique multi-epitope peptide designed based on ACPA cross-reactivity might be suitable to develop better diagnostics and novel therapies for RA.

Publication Type
Journal Article.
Year of Publication
2018

<128>
Unique Identifier
29341936
Title
Rheumatic manifestations among cancer patients treated with immune checkpoint inhibitors.
Autoimmunity Reviews. 17(3):284-289, 2018 Mar.

Authors
Lidar M; Giat E; Garelick D; Horowitz Y; Amital H; Steinberg-Silman Y; Schachter J; Shapira-Frommer R; Markel G.
Authors Full Name
Lidar, Merav; Giat, Eitan; Garelick, Daniela; Horowitz, Yuval; Amital, Howard; Steinberg-Silman, Yael; Schachter, Jacob; Shapira-Frommer, Ronnie; Markel, Gal.
Institution
Lidar, Merav. Rheumatology Unit, Sheba Medical Center, Israel; Sackler Faculty of Medicine, Tel Aviv University, Tel Aviv, Israel. Electronic address: Merav.Lidar@sheba.health.gov.il.
Giat, Eitan. Rheumatology Unit, Sheba Medical Center, Israel.

Garelick, Daniela. Rheumatology Unit, Sheba Medical Center, Israel.
Horowitz, Yuval. Rheumatology Unit, Sheba Medical Center, Israel.
Amital, Howard. Sackler Faculty of Medicine, Tel Aviv University, Tel Aviv, Israel; Internal Medicine B, Sheba Medical Center, Israel.
Steinberg-Silman, Yael. Ella Lemelbaum Institute for Immuno-Oncology, Sheba Medical Center, Israel.
Schachter, Jacob. Sackler Faculty of Medicine, Tel Aviv University, Tel Aviv, Israel; Ella Lemelbaum Institute for Immuno-Oncology, Sheba Medical Center, Israel.
Shapira-Frommer, Ronnie. Ella Lemelbaum Institute for Immuno-Oncology, Sheba Medical Center, Israel.
Markel, Gal. Sackler Faculty of Medicine, Tel Aviv University, Tel Aviv, Israel; Ella Lemelbaum Institute for Immuno-Oncology, Sheba Medical Center, Israel. Electronic address: Gal.Markel@sheba.health.gov.il.
Abstract
BACKGROUND: The use of immune checkpoint inhibitors (ICI) has grown incessantly since they were first approved in 2014. These monoclonal antibodies inhibit T cell activation, yielding a dramatic tumor response with improved survival. However, immunotherapy is frequently hampered by immune adverse events (iAE) such as hypophysitis, colitis, hepatitis, pneumonitis and rash. Until recently, rheumatic side effects were only infrequently reported.

AIM: To describe the rheumatic manifestations encountered among patients treated with ICIs in a large tertiary cancer center in Israel METHODS: The cancer center's patient registry was screened for patients who had ever been treated with ipilimumab, pembrolizumab and/or nivolumab with relevant data gathered from clinical charts.

RESULTS: Rheumatic manifestations were encountered in 14 of 400 patients (3.5%) who had received immunotherapy between January 1st 2013 and April 30th, 2017. The most common rheumatic manifestation was inflammatory arthritis (85%) for which a third (4/11) had a clear cut predisposing factor such as a personal or family history of psoriasis, a prior episode of uveitis or ACPA positivity. Pulmonary sarcoidosis and biopsy-proven eosinophilic fasciitis were diagnosed in two additional patients. Treatment with NSAIDS was mostly unsuccessful while steroid therapy was beneficial in doses >=20mg/d. Methotrexate enabled steroid tapering without an excess of side effects or tumor progression in the short follow-up available. Overall, rheumatic manifestations tended to occur later in the course of immunotherapy as compared to other iAE.

CONCLUSIONS: Our findings underscore that rheumatic iAE are part of the side effect profile of ICIs and require heightened awareness as these therapies are becoming the standard of care

for various malignancies. We show that these appear later in the course of iAEs and respond preferentially to high dose steroids. MTX appears effective as a steroid sparing agent. Copyright © 2018. Published by Elsevier B.V.
Publication Type
  Journal Article.
Year of Publication
  2018

<129>
Unique Identifier
  29337460
Title
  [Rheumatology. Checkpoint-induced autoimmunity - birth of a new disease]. [French]
Revue Medicale Suisse. 14(588-589):93-96, 2018 Jan 10.

REVIEW
Authors
  Dan D; Tapparel L; Hugle T.
Authors Full Name
  Dan, Diana; Tapparel, Ludovic; Hugle, Thomas.
Institution
  Dan, Diana. Service de rhumatologie, CHUV, 1011 Lausanne.
  Tapparel, Ludovic. Service de rhumatologie, CHUV, 1011 Lausanne.
  Hugle, Thomas. Service de rhumatologie, CHUV, 1011 Lausanne.
Abstract
  Tumor cells express checkpoint proteins in order to prevent an immune reaction by T-cells. Checkpoint inhibitors are successfully used in oncology to unleash a cytotoxic immune response. Unfortunately this treatment increasingly leads to immune-related adverse events which resemble various primary autoimmune disorders known in rheumatology. Potentially, checkpoint dysfunction also underlies rheumatic diseases which would open the way for new treatment options to restore immune tolerance.
Other Abstract

Publisher: En exprimant certaines molecules regulatrices (denommees << checkpoints >>) a leur surface, les tumeurs parviennent a eviter la reconnaissance par le systeme immunitaire. L'inhibition de ces checkpoints peut donc permettre une reponse immunitaire, mediee par les lymphocytes T, contre les cellules tumorales. L'utilisation croissante des << checkpoint inhibitors >> en oncologie a permis d'augmenter considerablement la survie des patients, mais a egalement engendre des effets indesirables autoimmuns ressemblant sur le plan clinique aux maladies primaires que l'on connait en rhumatologie. Des traitements corrigeant une dysfonction des checkpoints pourront probablement dans le futur egalement traiter des maladies autoimmunes et retablir l'immunotolerance.; Language: French

Publication Type

Journal Article.

Year of Publication

2018

<130>

Unique Identifier

29306173

Title

Blocking osteopontin-fibronectin interactions reduce extracellular fibronectin deployment and arthritic immunopathology.

Source

International Immunopharmacology. 55:297-305, 2018 Feb.

VI 1

Status

MEDLINE

Authors

Mehta BB; Sharma S; Vasishta RK; Sen RK; Sharma A; Luthra-Guptasarma M.

Authors Full Name

Mehta, Brij Bhushan; Sharma, Saniya; Vasishta, Rakesh K; Sen, Ramesh K; Sharma, Aman; Luthra-Guptasarma, Manni.

Institution

Mehta, Brij Bhushan. Department of Immunopathology, Postgraduate Institute of Medical Education and Research, Chandigarh 160012, India.

Sharma, Saniya. Department of Histopathology, Postgraduate Institute of Medical Education and Research, Chandigarh 160012, India.
Vasishta, Rakesh K. Department of Histopathology, Postgraduate Institute of Medical Education and Research, Chandigarh 160012, India.
Sen, Ramesh K. Department of Orthopaedics, Postgraduate Institute of Medical Education and Research, Chandigarh 160012, India.
Sharma, Aman. Department of Internal Medicine, Postgraduate Institute of Medical Education and Research, Chandigarh 160012, India.
Luthra-Guptasarma, Manni. Department of Immunopathology, Postgraduate Institute of Medical Education and Research, Chandigarh 160012, India. Electronic address: guptasarma.manni@pgimer.edu.in.
Abstract
Elevated levels of a thrombin-cleaved fragment of osteopontin (OPNT) are seen in synovial fluid (SF) and tissues of rheumatoid arthritis (RA) patients. OPNT binds to integrins on cell surfaces, inducing adhesion, migration and survival of inflammatory cells in the synovial joints, where OPNT binds to fibronectin to link fibroblast-like synoviocytes (FLS) with B cells, stimulating the latter to produce inflammatory cytokines. Our aim was to block OPNT-fibronectin interactions and examine whether this reduces inflammation. A human antibody (phage displayed) library was used to select scFv antibodies cognate to OPNT, and a particular scFv antibody (scFv 31) was evaluated. Adhesion, migration and fibronectin polymerization of FLS cells derived from RA patients were monitored, in cultures incorporating scFv 31. Also, scFv 31 was used in mice with CAIA (collagen antibody-induced arthritis), subjected to clinical and histological assessment, analysis of fibronectin and cartilage damage and induction of pro-inflammatory cytokines. The scFv antibody, scFv 31, appeared to cause significantly reduced migration of synovial fibroblasts, altered cell morphology, changes in actin stress fiber arrangement, and marked reduction in fibronectin. In CAIA mice, scFv 31 appeared to prevent arthritic changes through inhibition of synovial hypertrophy and loss of articular cartilage, decrease in fibronectin polymerization and expression of pro-inflammatory cytokines implicated in arthritis. Osteopontin-fibronectin interaction(s) appear to play a role in the expression of key inflammatory molecules by B cells infiltrating the synovial joint. The scFv antibody, scFv 31, provides a potential therapeutic lead for inhibition of some processes implicated in rheumatoid arthritis. Copyright © 2017. Published by Elsevier B.V.
Publication Type
Journal Article.
Year of Publication
2018

<131>

Unique Identifier

29303710

Title

Vaccinations in adults with rheumatoid arthritis in an era of new disease-modifying anti-rheumatic drugs. [Review]

Source

Clinical & Experimental Rheumatology. 36(2):317-328, 2018 Mar-Apr.

VI 1

Status

MEDLINE

Authors

Meroni PL; Zavaglia D; Girmenia C.

Authors Full Name

Meroni, Pier Luigi; Zavaglia, Daniela; Girmenia, Corrado.

Institution

Meroni, Pier Luigi. Department of Clinical Sciences and Community Health, University of Milan, Department of Rheumatology, ASST-G. Pini, Immunorheumatology research laboratory, IRCCS Istituto Auxologico Italiano, Milan, Italy. pierluigi.meroni@unimi.it.

Zavaglia, Daniela. Medical Department, Pfizer Italia, Rome, Italy.

Girmenia, Corrado. Department of Haematology, Oncology and Dermatology, Azienda Policlinico Umberto I, Sapienza University, Rome, Italy.

Abstract

Patients with rheumatoid arthritis are at greater risk of infectious morbidity and mortality due to disease-related abnormalities and use of immunosuppressive medications. Vaccinations are recommended by international guidelines among infection control strategies, but vaccination rates are reported to be still suboptimal in both America and Europe. Furthermore, with the increasing number of immunomodulatory medications used in RA patients, safety and efficacy of vaccinations in RA patients on such therapies have been questioned. This paper reviews current data about the safety of the most relevant vaccinations for RA adult patients and on the extent to which RA treatment can affect vaccine efficacy. Although it is recognised that immunological and pathological reactions can occur following vaccination, especially in genetically susceptible hosts, early data in RA patients under treatment with bDMARDs or tsDMARDs indicate that vaccines

might be safer in the setting of immunosuppression than previously thought. Reviewing safety and immunogenicity data about influenza, pneumococcal, HZ, HPV, and HBV vaccines, we here try to summarise updated, practical suggestions for rheumatologists. Improving the knowledge of the vaccination practice both in patients and physicians is of crucial importance. In RA patients, vaccination status should be assessed in the initial patients' work-up and vaccination strategies should be planned and then implemented ideally during stable disease, as recommended by international guidelines.

Publication Type

Journal Article. Review.

Year of Publication

2018

<132>

Unique Identifier

29264628

Title

[Perioperative handling of immunosuppressive therapy]. [Review] [German]

Source

Chirurg. 89(2):116-121, 2018 02.

VI 1

Status

MEDLINE

Authors

Wagner J; Luber V; Lock JF; Dietz UA; Lichthardt S; Matthes N; Krajinovic K; Germer CT; Knop S; Wiegering A.

Authors Full Name

Wagner, J; Luber, V; Lock, J F; Dietz, U A; Lichthardt, S; Matthes, N; Krajinovic, K; Germer, C-T; Knop, S; Wiegering, A.

Institution

Wagner, J. Klinik fur Allgemein-, Viszeral-, Gefas- und Kinderchirurgie, Universitatsklinikum Wurzburg, Oberduerrbacherstr. 6, 97080, Wurzburg, Deutschland.

Luber, V. Klinik fur Innere Medizin II, Universitatsklinikum Wurzburg, Wurzburg, Deutschland.

Lock, J F. Klinik fur Allgemein-, Viszeral-, Gefas- und Kinderchirurgie, Universitatsklinikum Wurzburg, Oberduerrbacherstr. 6, 97080, Wurzburg, Deutschland.
Dietz, U A. Klinik fur Allgemein-, Viszeral-, Gefas- und Kinderchirurgie, Universitatsklinikum Wurzburg, Oberduerrbacherstr. 6, 97080, Wurzburg, Deutschland.
Lichthardt, S. Klinik fur Allgemein-, Viszeral-, Gefas- und Kinderchirurgie, Universitatsklinikum Wurzburg, Oberduerrbacherstr. 6, 97080, Wurzburg, Deutschland.
Matthes, N. Klinik fur Allgemein-, Viszeral-, Gefas- und Kinderchirurgie, Universitatsklinikum Wurzburg, Oberduerrbacherstr. 6, 97080, Wurzburg, Deutschland.
Krajinovic, K. Klinik fur Allgemein-, Viszeral-, Gefas- und Kinderchirurgie, Universitatsklinikum Wurzburg, Oberduerrbacherstr. 6, 97080, Wurzburg, Deutschland.
Germer, C-T. Klinik fur Allgemein-, Viszeral-, Gefas- und Kinderchirurgie, Universitatsklinikum Wurzburg, Oberduerrbacherstr. 6, 97080, Wurzburg, Deutschland.
Germer, C-T. Comprehensive Cancer Center Mainfranken, Universitatsklinikum Wurzburg, Wurzburg, Deutschland.
Knop, S. Klinik fur Innere Medizin II, Universitatsklinikum Wurzburg, Wurzburg, Deutschland.
Knop, S. Comprehensive Cancer Center Mainfranken, Universitatsklinikum Wurzburg, Wurzburg, Deutschland.
Wiegering, A. Klinik fur Allgemein-, Viszeral-, Gefas- und Kinderchirurgie, Universitatsklinikum Wurzburg, Oberduerrbacherstr. 6, 97080, Wurzburg, Deutschland. wiegering_a@ukw.de.
Wiegering, A. Institut fur Biochemie und Molekularbiologie, Universitat Wurzburg, Wurzburg, Deutschland. wiegering_a@ukw.de.

Abstract

Every year 16 million operations are performed in Germany. Many patients have an autoimmune disorder, for example rheumatoid arthritis, psoriasis or chronic inflammatory bowel disease, which requires treatment. Immunosuppressants are widely applied. Physicians must make a risk-adapted decision whether the immunosuppressant medication can be continued perioperatively or if certain drugs must be paused and if so, with what risks. The handling of immunosuppressants during the perioperative period is very relevant as many patients, for example with rheumatoid arthritis are in need of a hip or knee replacement or patients with inflammatory bowel disease need an operation due to the chronic illness. The interruption of an immunosuppressant therapy should be discussed in an interdisciplinary board according to the underlying disease, because the continuation of immunosuppressants perioperatively can lead to an increased rate of complications, especially wound healing disorders. If a patient is on a glucocorticoid therapy the following must be considered: during the perioperative period the body has an increased demand for glucocorticoids due to the stress reaction. If glucocorticoids are administered in a dosage of more than 7.5 mg/day equivalent of prednisolone this stress reaction

is inhibited. Thus, in these cases a perioperative substitution with hydrocortisone is recommended.

Publication Type

Journal Article. Review.

Year of Publication

2018

<133>

Unique Identifier

29242008

Title

Immune responses to peptides containing homocitrulline or citrulline in the DR4-transgenic mouse model of rheumatoid arthritis.

Source

Journal of Autoimmunity. 89:75-81, 2018 05.

VI 1

Status

MEDLINE

Authors

Lac P; Saunders S; Tutunea-Fatan E; Barra L; Bell DA; Cairns E.

Authors Full Name

Lac, Patrick; Saunders, Sheri; Tutunea-Fatan, Elena; Barra, Lillian; Bell, David A; Cairns, Ewa.

Institution

Lac, Patrick. Department of Microbiology and Immunology, The University of Western Ontario, London, ON, Canada.

Saunders, Sheri. Department of Microbiology and Immunology, The University of Western Ontario, London, ON, Canada.

Tutunea-Fatan, Elena. Department of Microbiology and Immunology, The University of Western Ontario, London, ON, Canada.

Barra, Lillian. Department of Microbiology and Immunology, The University of Western Ontario, London, ON, Canada; Department of Medicine/Rheumatology, The University of Western Ontario, London, ON, Canada.

Bell, David A. Department of Medicine/Rheumatology, The University of Western Ontario, London, ON, Canada.
Cairns, Ewa. Department of Microbiology and Immunology, The University of Western Ontario, London, ON, Canada; Department of Medicine/Rheumatology, The University of Western Ontario, London, ON, Canada. Electronic address: ewa.cairns@schulich.uwo.ca.
Abstract
Antibodies to proteins/peptides containing citrulline are hallmarks of Rheumatoid Arthritis (RA). These antibodies are strongly associated with the expression of the Shared Epitope (SE). RA patients also generate antibodies to homocitrulline-containing proteins/peptides (also referred to as anti-carbamylated protein antibodies (Anti-CarP)). This study was undertaken to investigate the relationship between homocitrulline and citrulline immune responses using an established mouse model of RA: DR4-transgenic (DR4tg) mice that express the human SE. C57BL/6 (B6) and DR4tg (on a B6 background) mice were immunized subcutaneously with a homocitrullinated peptide (HomoCitJED). Splenic T cell proliferation was evaluated by 3H-thymidine incorporation assay. Antibodies to homocitrullinated and citrullinated antigens were screened by enzyme-linked immunosorbent assay (ELISA). Antibody cross-reactivity was examined by inhibition with HomoCitJED and its citrullinated counterpart peptide, CitJED (the number of homocitrullines in HomoCitJED is equal to the number of citrullines in CitJED). HomoCitJED-immunized DR4tg mice developed early T and B cell responses to HomoCitJED and late responses to CitJED. These mice also developed anti-CCP2 antibodies. In some mice, antibodies to HomoCitJED were also reactive to CitJED. B6 mice immunized with HomoCitJED developed late T and B cell responses to HomoCitJED, but did not generate responses to citrullinated antigens. Unlike DR4tg mice, anti-HomoCitJED antibodies from B6 mice did not react to CitJED. In conclusion, DR4tg mice immunized with HomoCitJED developed immune responses to CitJED, indicating cross-reactivity. CitJED immune responses were dependent on the SE. HomoCitJED responses occurred in the absence of the SE (B6 mice); however, they developed earlier in DR4tg SE-expressing mice. Copyright © 2017 Elsevier Ltd. All rights reserved.
Publication Type
Journal Article. Research Support, Non-U.S. Gov't.
Year of Publication
2018

<134>

Unique Identifier
29232324
Title
Vaccination Recommendations for Adults With Autoimmune Inflammatory Rheumatic Diseases in Latin America.
Source
JCR: Journal of Clinical Rheumatology. 24(3):138-147, 2018 Apr.
VI 1
Status
MEDLINE
Authors
Brenol CV; Azevedo VF; Bonvehi PE; Coral-Alvarado PX; Granados J; Munoz-Louis R; Pineda C; Vizzotti C.
Authors Full Name
Brenol, Claiton Viegas; Azevedo, Valderilio Feijo; Bonvehi, Pablo Eduardo; Coral-Alvarado, Paola Ximena; Granados, Julio; Munoz-Louis, Roberto; Pineda, Carlos; Vizzotti, Carla.
Abstract
BACKGROUND/OBJECTIVE: Patients with autoimmune inflammatory rheumatic diseases (AIRDs) are at increased risk of contracting severe infections and suffering complications, particularly when they are receiving immunomodulating therapy. Vaccination is an important means to prevent many potential infections and thereby reduce the morbidity and mortality associated with AIRD. The purpose of this consensus document is to provide health care professionals with recommendations for the vaccination of AIRD patients who reside in Latin America. The recommendations were developed by an expert committee from the region based on a review of the literature and their clinical experience.

METHODS: The Americas Health Foundation (AHF) used PubMed and EMBASE to identify clinicians and scientists with an academic or hospital affiliation and who had published in the field of adult vaccination and rheumatic diseases since 2010. As a result of this effort, AHF convened an 8-member panel of clinical and scientific experts from Latin America. Both the AHF and panel members conducted a careful literature review to identify relevant publications in the areas of adult vaccination and rheumatology, and the sum of the articles identified was provided to the entire panel. Prior to the conference, panelists were each asked to prepare a written response to a salient issue on the subject, identified by AHF.

RESULTS AND CONCLUSIONS: During the conference, each response was edited by the entire group, through numerous drafts and rounds of discussion until a complete consensus on

vaccination recommendations for adult patients with AIRDs was obtained, including 7 key recommendations.
Publication Type
  Journal Article.
Year of Publication
  2018

<135>
Unique Identifier
  29223453
Title
  HBV reactivation in rheumatic diseases patients under therapy: A meta-analysis. [Review]
Source
  Microbial Pathogenesis. 114:436-443, 2018 Jan.
VI 1
Status
  MEDLINE
Authors
  Moghoofei M; Mostafaei S; Ashraf-Ganjouei A; Kavosi H; Mahmoudi M.
Authors Full Name
  Moghoofei, Mohsen; Mostafaei, Shayan; Ashraf-Ganjouei, Amir; Kavosi, Hoda; Mahmoudi, Mahdi.
Institution
  Moghoofei, Mohsen. Department of Microbiology, Faculty of Medicine, Kermanshah University of Medical Sciences, Kermanshah, Iran.
  Mostafaei, Shayan. Rheumatology Research Center, Tehran University of Medical Sciences, Tehran, Iran.
  Ashraf-Ganjouei, Amir. Rheumatology Research Center, Tehran University of Medical Sciences, Tehran, Iran.
  Kavosi, Hoda. Rheumatology Research Center, Tehran University of Medical Sciences, Tehran, Iran. Electronic address: h-kavosi@tums.ac.ir.
  Mahmoudi, Mahdi. Rheumatology Research Center, Tehran University of Medical Sciences, Tehran, Iran. Electronic address: mahmoudim@tums.ac.ir.

Abstract
BACKGROUND: Hepatitis B is one of the most common infectious diseases worldwide. In patients undergoing immunosuppressive therapy such as rheumatic diseases, reactivation of hepatitis B virus (HBV) is considered clinically important. This systematic review and meta-analysis were performed to determine the prevalence rate of HBV reactivation in rheumatic patients from different parts of the world.

METHODS: The authors performed a systematic literature review from several reliable databases including Scopus, ISI Web of Science and PubMed. Furthermore, the keywords of this research were "Hepatitis B virus", "Rheumatic diseases", "HBV reactivation", "Anti-TNF", "DMARDs" and "Biologic agents".

RESULTS: The authors selected 30 studies out of 983 for the present review. The overall estimation of the prevalence of HBV reactivation was 1.4 (95% confidence interval (CI): 1.3-1.6). Also, the heterogeneity in estimating the pooled prevalence among the studies was shown; Cochran Q test, $P < 0.001$, $I2 = 99.9\%$. It should be noted that max and min reactivation rate of HBV were in Italy and France respectively.

CONCLUSIONS: Rheumatic disease patients with resolved hepatitis B should be tightly monitored for possible HBV reactivation by elevation of liver enzymes and HBV DNA levels.
Copyright © 2017 Elsevier Ltd. All rights reserved.
Publication Type
Journal Article. Review. Systematic Review.
Year of Publication
2018

<136>
Unique Identifier
29191572
Title
Th17 cells in primary Sjogren's syndrome: Pathogenicity and plasticity. [Review]
Source
Journal of Autoimmunity. 87:16-25, 2018 02.

VI 1

Status

MEDLINE

Authors

Verstappen GM; Corneth OBJ; Bootsma H; Kroese FGM.

Authors Full Name

Verstappen, Gwenny M; Corneth, Odilia B J; Bootsma, Hendrika; Kroese, Frans G M.

Institution

Verstappen, Gwenny M. Department of Rheumatology and Clinical Immunology, University of Groningen, University Medical Center Groningen, Hanzeplein 1, 9713 GZ Groningen, The Netherlands. Electronic address: g.m.p.j.verstappen@umcg.nl.

Corneth, Odilia B J. Department of Pulmonary Medicine, Erasmus Medical Center, 's-Gravendijkwal 230, 3015 CE Rotterdam, The Netherlands.

Bootsma, Hendrika. Department of Rheumatology and Clinical Immunology, University of Groningen, University Medical Center Groningen, Hanzeplein 1, 9713 GZ Groningen, The Netherlands.

Kroese, Frans G M. Department of Rheumatology and Clinical Immunology, University of Groningen, University Medical Center Groningen, Hanzeplein 1, 9713 GZ Groningen, The Netherlands.

Abstract

Th17 cells play an important physiological role at mucosal barriers, and are involved in inflammatory responses to pathogens. Th17 cells and their signature cytokine IL-17 are also present in salivary gland lesions of primary Sjogren's syndrome (pSS) patients and can be elevated in their peripheral blood. In pSS patients, clear correlations between increased Th17 cell activity and symptoms of the disease have not been found, but Th17 cells may contribute to disease progression, for example by supporting autoreactive B cell responses. In mouse models of pSS, Th17 cells play an important role in pathogenesis, particularly at disease onset, when there is a disturbed balance between T effector and T regulatory cells. Studying the pathogenicity of Th17 cells in humans is complicated due to the plasticity of this cell subset, allowing them to obtain different effector functions depending on the local environment. Th17 cells can develop towards Th17.1 cells, producing both IL-17 and IFN-gamma, or even towards Th1-like cells producing IFN-gamma in the absence of IL-17. These effector subsets may be more pathogenic than bona fide Th17 cells. Co-expression of IFN-gamma by Th17 cells has been shown to promote chronic inflammation in several autoimmune diseases and may also contribute to pSS pathogenesis. In line with the noticeable role of IL-17 in pSS mouse models, interference with Th17 cell generation, recruitment or effector functions (e.g. IL-17 inhibition) can prevent or ameliorate disease in these models. Therapies targeting Th17 cells or IL-17 have not been tested

so far in pSS patients, although treatment with rituximab seems to lower local and systemic IL-17 protein levels, and to a lesser extent also chemokine receptor-defined Th17 cells. In this review we discuss current knowledge of pathogenicity and plasticity of Th17 cells in human pSS and murine models of pSS. We postulate that plasticity towards Th17.1 cells in pSS may enhance pathogenicity of Th17 cells at the main target sites of the disease, i.e. salivary and lacrimal glands. Copyright © 2017 The Authors. Published by Elsevier Ltd.. All rights reserved.

Publication Type
  Journal Article. Review.

Year of Publication
  2018

<137>

Unique Identifier
  29191375

Title
  Rheumatologic symptoms in oncologic patients on PD-1 inhibitors. Seminars in Arthritis & Rheumatism. 47(6):907-910, 2018 06.

Authors
  Kuswanto WF; MacFarlane LA; Gedmintas L; Mulloy A; Choueiri TK; Bermas BL.

Authors Full Name
  Kuswanto, Wilson F; MacFarlane, Lindsey A; Gedmintas, Lydia; Mulloy, Alexandra; Choueiri, Toni K; Bermas, Bonnie L.

Institution
  Kuswanto, Wilson F. Harvard Medical School, Boston, MA.
   MacFarlane, Lindsey A. Harvard Medical School, Boston, MA; Division of Rheumatology, Immunology and Allergy, Brigham and Women's Hospital, 60 Fenwood Rd, Boston, MA. Electronic address: lmacfarlane@partners.org.
   Gedmintas, Lydia. Harvard Medical School, Boston, MA; Division of Rheumatology, Immunology and Allergy, Brigham and Women's Hospital, 60 Fenwood Rd, Boston, MA.
   Mulloy, Alexandra. Department of Medical Oncology, Dana-Farber Cancer Institute, Harvard Medical School, Boston, MA, USA.
   Choueiri, Toni K. Harvard Medical School, Boston, MA; Department of Medical Oncology, Dana-Farber Cancer Institute, Harvard Medical School, Boston, MA, USA.

Bermas, Bonnie L. Harvard Medical School, Boston, MA; Division of Rheumatology, Immunology and Allergy, Brigham and Women's Hospital, 60 Fenwood Rd, Boston, MA.

Abstract

OBJECTIVE: Immune checkpoint inhibitors are effective cancer therapies that have been associated with immune-related adverse events (irAEs). Recent reports of irAEs describe symptoms resembling classic rheumatologic syndromes, most notably associated with cytotoxic T-lymphocyte-associated protein 4 (CTLA-4) inhibitor blockade. Though cases have been described, there are fewer reports of rheumatologic disease associated with programmed cell death protein-1 (PD-1) inhibitors. Here, we describe a series of four patients presenting to the Brigham and Women's Hospital (BWH) Arthritis Center with de novo polymyalgia rheumatica (PMR)-type conditions and/or peripheral synovitis after treatment with PD-1/PD-Ligand 1 (PD-L1) pathway inhibitors.

METHODS: Patients with metastatic renal cell carcinoma (RCC) who were treated with PD-1/PD-L1 pathway inhibitors and subsequently developed complaints of new joint pain were referred to the BWH Arthritis Center as part of routine care and identified retrospectively. The electronic medical record was reviewed for cancer history and treatment, rheumatologic symptoms, physical exam, laboratory testing, and clinical course.

RESULTS: All four patients developed irAEs consistent with a PMR-type syndrome and/or peripheral synovitis. Symptoms persisted despite discontinuation of the PD-1/PD-L1 pathway inhibitors; however, three of the patients responded well to oral glucocorticoids alone while one patient required the addition of oral methotrexate. All patients had an eventual decline in inflammatory markers.

CONCLUSION: These cases highlight the need for both oncologists and rheumatologists to recognize the development of rheumatologic disease during treatment with immune checkpoint blockade. Further investigation is needed to optimize the management of irAEs, particularly considering the increasing use of checkpoint inhibitors to treat malignancies. Copyright © 2017 Elsevier Inc. All rights reserved.

Publication Type

Case Reports. Journal Article.

Year of Publication

2018

<138>

Unique Identifier

29146737

Title

Rheumatic disorders associated with immune checkpoint inhibitors in patients with cancer-clinical aspects and relationship with tumour response: a single-centre prospective cohort study.

Annals of the Rheumatic Diseases. 77(3):393-398, 2018 03.

Authors

Kostine M; Rouxel L; Barnetche T; Veillon R; Martin F; Dutriaux C; Dousset L; Pham-Ledard A; Prey S; Beylot-Barry M; Daste A; Gross-Goupil M; Lallier J; Ravaud A; Forcade E; Bannwarth B; Truchetet ME; Richez C; Mehsen N; Schaeverbeke T; FHU ACRONIM.

Authors Full Name

Kostine, Marie; Rouxel, Lea; Barnetche, Thomas; Veillon, Remi; Martin, Florent; Dutriaux, Caroline; Dousset, Lea; Pham-Ledard, Anne; Prey, Sorilla; Beylot-Barry, Marie; Daste, Amaury; Gross-Goupil, Marine; Lallier, Julie; Ravaud, Alain; Forcade, Edouard; Bannwarth, Bernard; Truchetet, Marie-Elise; Richez, Christophe; Mehsen, Nadia; Schaeverbeke, Thierry; FHU ACRONIM.

Institution

Kostine, Marie. Rheumatology Department, Centre Hospitalier Universitaire, Bordeaux, Aquitaine, France.

Rouxel, Lea. Rheumatology Department, Centre Hospitalier Universitaire, Bordeaux, Aquitaine, France.

Barnetche, Thomas. Rheumatology Department, Centre Hospitalier Universitaire, Bordeaux, Aquitaine, France.

Veillon, Remi. Pulmonology Department, Centre Hospitalier Universitaire, Bordeaux, Aquitaine, France.

Martin, Florent. Pulmonology Department, Centre Hospitalier Universitaire, Bordeaux, Aquitaine, France.

Dutriaux, Caroline. Dermatology Department, Centre Hospitalier Universitaire, Bordeaux, Aquitaine, France.

Dousset, Lea. Dermatology Department, Centre Hospitalier Universitaire, Bordeaux, Aquitaine, France.

Pham-Ledard, Anne. Dermatology Department, Centre Hospitalier Universitaire, Bordeaux, Aquitaine, France.

Prey, Sorilla. Dermatology Department, Centre Hospitalier Universitaire, Bordeaux, Aquitaine, France.

Beylot-Barry, Marie. Dermatology Department, Centre Hospitalier Universitaire, Bordeaux, Aquitaine, France.

Daste, Amaury. Oncology Department, Centre Hospitalier Universitaire, Bordeaux, Aquitaine, France.

Gross-Goupil, Marine. Oncology Department, Centre Hospitalier Universitaire, Bordeaux, Aquitaine, France.

Lallier, Julie. Oncology Department, Centre Hospitalier Universitaire, Bordeaux, Aquitaine, France.

Ravaud, Alain. Oncology Department, Centre Hospitalier Universitaire, Bordeaux, Aquitaine, France.

Forcade, Edouard. Hematology Department, Centre Hospitalier Universitaire, Bordeaux, Aquitaine, France.

Bannwarth, Bernard. Rheumatology Department, Centre Hospitalier Universitaire, Bordeaux, Aquitaine, France.

Truchetet, Marie-Elise. Rheumatology Department, Centre Hospitalier Universitaire, Bordeaux, Aquitaine, France.

Richez, Christophe. Rheumatology Department, Centre Hospitalier Universitaire, Bordeaux, Aquitaine, France.

Mehsen, Nadia. Rheumatology Department, Centre Hospitalier Universitaire, Bordeaux, Aquitaine, France.

Schaeverbeke, Thierry. Rheumatology Department, Centre Hospitalier Universitaire, Bordeaux, Aquitaine, France.

Comments

Comment in (CIN)

Abstract

OBJECTIVES: To evaluate the prevalence and type of rheumatic immune-related adverse events (irAEs) in patients receiving immune checkpoint inhibitors (ICIs), as well as the correlation with tumour response.

METHODS: This was a single-centre prospective observational study including all cancer patients receiving ICIs. The occurrence of irAEs and tumour response was assessed on a regular basis. Patients who experienced musculoskeletal symptoms were referred to the department of rheumatology for clinical evaluation and management.

RESULTS: From September 2015 to May 2017, 524 patients received ICIs and 35 were referred to the department of rheumatology (6.6%). All but one of the rheumatic irAEs occurred with anti-programmed cell death protein 1(PD-1)/PD-1 ligand 1(PD-L1) antibodies, with a median exposure time of 70 days. There were two distinct clinical presentations: (1) inflammatory arthritis (3.8%) mimicking either rheumatoid arthritis (n=7), polymyalgia rheumatica (n=11) or psoriatic arthritis (n=2) and (2) non-inflammatory musculoskeletal conditions (2.8%; n=15). One patient with rheumatoid arthritis was anti-cyclic citrullinated peptide (anti-CCP) positive. Nineteen patients required glucocorticoids, and methotrexate was started in two patients. Non-inflammatory disorders were managed with non-steroidal anti-inflammatory drugs, analgesics and/or physiotherapy. ICI treatment was pursued in all but one patient. Patients with rheumatic irAEs had a higher tumour response rate compared with patients without irAEs (85.7% vs 35.3%; P<0.0001).

CONCLUSION: Since ICIs are used with increasing frequency, knowledge of rheumatic irAEs and their management is of major interest. All patients were responsive either to low-to-moderate doses of prednisone or symptomatic therapies and did not require ICI discontinuation. Furthermore, tumour response was significantly higher in patients who experienced rheumatic irAEs. Copyright © Article author(s) (or their employer(s) unless otherwise stated in the text of the article) 2018. All rights reserved. No commercial use is permitted unless otherwise expressly granted.

Publication Type

Journal Article. Observational Study.

Year of Publication

2018

<139>

Unique Identifier

29037819

Title

Drug-Induced Paradoxical Vocal Fold Motion. [Review]

Source

The Journal of Allergy & Clinical Immunology in Practice. 6(1):90-94, 2018 Jan - Feb.

VI 1

Status

MEDLINE

Authors

Garcia-Neuer M; Lynch DM; Marquis K; Dowdall J; Castells M; Sloane DE.

Authors Full Name

Garcia-Neuer, Marlene; Lynch, Donna Marie; Marquis, Kathleen; Dowdall, Jayme; Castells, Mariana; Sloane, David Edward.

Institution

Garcia-Neuer, Marlene. Division of Rheumatology, Allergy and Immunology, Brigham and Women's Hospital, Boston, Mass.

Lynch, Donna Marie. Division of Rheumatology, Allergy and Immunology, Brigham and Women's Hospital, Boston, Mass.

Marquis, Kathleen. Pharmacy Services, Brigham and Women's Hospital, Boston, Mass.

Dowdall, Jayme. Division of Otolaryngology, Department of Surgery, Brigham and Women's Hospital, Boston, Mass; Harvard Medical School, Boston, Mass.

Castells, Mariana. Division of Rheumatology, Allergy and Immunology, Brigham and Women's Hospital, Boston, Mass; Harvard Medical School, Boston, Mass.

Sloane, David Edward. Division of Rheumatology, Allergy and Immunology, Brigham and Women's Hospital, Boston, Mass; Harvard Medical School, Boston, Mass. Electronic address: dsloane@bwh.harvard.edu.

Abstract

Vocal cord dysfunction, also known as paradoxical vocal fold motion (PVFM), is a disorder characterized by abnormal vocal cord adduction during inspiration. PVFM is commonly misdiagnosed as asthma because of the similarity of symptoms: cough, wheezing, chest pain, and dyspnea. We present the clinical vignette of a 36-year-old woman with juvenile rheumatoid arthritis and multiple adverse drug reactions who presented with recurrent episodes of unrecognized PVFM during skin testing for drug allergy, omalizumab treatment, and tocilizumab desensitization. Before the diagnosis of PVFM, these episodes were treated as anaphylaxis, including the administration of epinephrine. Once diagnosed and treated for PVFM, the patient did not present any further events and continued treatment for drug allergy. PVFM may be underreported in hypersensitivity reactions because of the similarity to Type 1-mediated respiratory symptoms and comorbid asthma. Copyright © 2017 American Academy of Allergy, Asthma & Immunology. Published by Elsevier Inc. All rights reserved.

Publication Type

Case Reports. Journal Article. Review.

Year of Publication

2018

<140>

Unique Identifier

29023682

Title

Short-term cytokine stimulation reveals regulatory T cells with down-regulated Foxp3 expression in human peripheral blood.

Source

European Journal of Immunology. 48(2):366-379, 2018 02.

VI 1

Status

MEDLINE

Authors

Tabares P; Berr S; Langenhorst D; Sawitzki B; Ten Berge I; Tony HP; Hunig T.

Author NameID

Tabares, Paula; ORCID: https://orcid.org/0000-0003-0401-1711

Authors Full Name

Tabares, Paula; Berr, Susanne; Langenhorst, Daniela; Sawitzki, Birgit; Ten Berge, Ineke; Tony, Hans-Peter; Hunig, Thomas.

Institution

Tabares, Paula. Institute for Virology and Immunobiology, University of Wurzburg, Wurzburg, Germany.

Berr, Susanne. Institute for Virology and Immunobiology, University of Wurzburg, Wurzburg, Germany.

Langenhorst, Daniela. Institute for Virology and Immunobiology, University of Wurzburg, Wurzburg, Germany.

Sawitzki, Birgit. Institute of Medical Immunology, Charite University Medicine, Berlin, Germany.

Ten Berge, Ineke. Department of Internal Medicine, Academic Medical Center, University of Amsterdam, Amsterdam, The Netherlands.

Tony, Hans-Peter. Department of Internal Medicine II, University Hospital of Wurzburg, Wurzburg, Germany.

Hunig, Thomas. Institute for Virology and Immunobiology, University of Wurzburg, Wurzburg, Germany.

Abstract
The identification of regulatory T cells (Treg cells) in human peripheral blood is an important tool in diagnosis, research, and therapeutic intervention. As compared to lymphoid tissues, the frequencies of circulating Treg cells identified as CD4+ CD25+ Foxp3+ are, however, low. We here show that many of these cells remain undetected due to transient down regulation of Foxp3, which rapidly decays in the absence of cytokine-mediated STAT5 signals. Short-term incubation of PBMCs or isolated CD4+ T cells, but not of lymph node cells, with IL-2, -7, or -15 more than doubles the frequency of Foxp3+ CD25+ among CD4+ T cells detectable by flow cytometry. This increase is not due to cell division but to upregulation of both proteins. At the same time, the uncovered Treg cells up-regulate CD25 and down-regulate CD127, making them accessible to viable cell sorting. "Latent" Treg cells have a demethylated FOXP3 TSDR sequence, are enriched in naive, non-cycling cells, and are functional. The confirmation of our findings in RA and SLE patients shows the feasibility of uncovering latent Treg cells for immune monitoring in clinical settings. Finally, our results suggest that unmasking of latent Treg cells contributes to the increase in circulating CD4+ CD25+ Foxp3+ cells reported in IL-2 treated patients. Copyright © 2017 WILEY-VCH Verlag GmbH & Co. KGaA, Weinheim.
Publication Type
Journal Article. Research Support, Non-U.S. Gov't.
Year of Publication
2018

<141>
Unique Identifier
28980305
Title
Ly6Chigh monocytes facilitate transport of Murid herpesvirus 68 into inflamed joints of arthritic mice.
Source
European Journal of Immunology. 48(2):250-257, 2018 02.
VI 1
Status
MEDLINE
Authors

LeBel M; Egarnes B; Brunet A; Lacerte P; Pare A; Lacroix S; Brown JP; Gosselin J.
Author NameID
Gosselin, Jean; ORCID: https://orcid.org/0000-0002-2244-4898
Authors Full Name
LeBel, Manon; Egarnes, Benoit; Brunet, Alexandre; Lacerte, Patricia; Pare, Alexandre; Lacroix, Steve; Brown, Jacques P; Gosselin, Jean.
Institution
LeBel, Manon. Laboratory of Innate Immunology, Centre de recherche du CHU de Quebec - Universite Laval, Quebec, QC, Canada.
Egarnes, Benoit. Laboratory of Innate Immunology, Centre de recherche du CHU de Quebec - Universite Laval, Quebec, QC, Canada.
Brunet, Alexandre. Department of Molecular Medicine, Universite Laval, Quebec, QC, Canada.
Brunet, Alexandre. Centre de recherche du CHU de Quebec - Universite Laval, Quebec, QC, Canada.
Lacerte, Patricia. Laboratory of Innate Immunology, Centre de recherche du CHU de Quebec - Universite Laval, Quebec, QC, Canada.
Pare, Alexandre. Centre de recherche du CHU de Quebec - Universite Laval, Quebec, QC, Canada.
Lacroix, Steve. Department of Molecular Medicine, Universite Laval, Quebec, QC, Canada.
Lacroix, Steve. Centre de recherche du CHU de Quebec - Universite Laval, Quebec, QC, Canada.
Brown, Jacques P. Division of Rheumatology, CHU de Quebec - Universite Laval (CHUL), Infectious and Immune Diseases, Centre de recherche du CHU de Quebec - Universite Laval (CHUL), Quebec, QC, Canada.
Gosselin, Jean. Laboratory of Innate Immunology, Centre de recherche du CHU de Quebec - Universite Laval, Quebec, QC, Canada.
Gosselin, Jean. Department of Molecular Medicine, Universite Laval, Quebec, QC, Canada.
Abstract
Viruses, particularly the Epstein-Barr virus (EBV) has long been suspected to exacerbate acute arthritic symptoms. However, the cell populations that contribute to import viruses into the inflamed tissues remain to be identified. In the present study, we have investigated the role of monocytes in the transport of Murid herpesvirus 68 (MHV-68), a mouse virus closely related to EBV, using the serum transfer-induced arthritis (STIA) model. We found compelling evidence that MHV-68 infection markedly increased disease severity in NR4A1-/- mice, which are deficient for Ly6Clow monocytes. In contrast, the MHV-68-induced enhancement of joint inflammation was lessened in CCR2-/- mice, suggesting the involvement of inflammatory Ly6Chigh monocytes in viral transport. We also observed that following selective depletion of monocyte subsets by

administration of clodronate, MHV-68 transport into the synovium occurs only in the presence of Ly6Chigh monocytes. Tracking of adoptively transferred Ly6Chigh GFP infected monocytes into arthritic CCR2-/- mice by two-photon intravital microscopy showed that this monocyte subset has the capacity to deliver viruses to inflamed AR joints, as confirmed by the detection of viral DNA in inflamed tissues of recipient mice. We thus conclude that Ly6Chigh monocytes import MHV-68 when they are mobilized to the inflamed arthritic joint. Copyright © 2017 WILEY-VCH Verlag GmbH & Co. KGaA, Weinheim.

Publication Type

Journal Article. Research Support, Non-U.S. Gov't.

Year of Publication

2018

<142>

Unique Identifier

28261976

Title

Prevalence of co-morbidities and evaluation of their monitoring in Korean patients with rheumatoid arthritis: comparison with the results of an international, cross-sectional study (COMORA).

Source

International Journal of Rheumatic Diseases. 21(7):1414-1422, 2018 Jul.

VI 1

Status

MEDLINE

Authors

Choi IA; Park SH; Cha HS; Park W; Kim HA; Yoo DH; Baek HJ; Lee SG; Lee YJ; Park YB; Shim SC; Hmamouchi I; Song YW.

Authors Full Name

Choi, In Ah; Park, Sung Hwan; Cha, Hoon-Suk; Park, Won; Kim, Hyun Ah; Yoo, Dae-Hyun; Baek, Han Joo; Lee, Seong Geun; Lee, Yun Jong; Park, Yong Bum; Shim, Seung-Cheol; Hmamouchi, Ihsane; Song, Yeong Wook.

Institution

Choi, In Ah. Division of Rheumatology, Department of Internal Medicine, Seoul National University Hospital, Seoul, Korea.
Park, Sung Hwan. Division of Rheumatology, Department of Internal Medicine, Seoul St. Mary's Hospital, Seoul, Korea.
Cha, Hoon-Suk. Division of Rheumatology, Department of Internal Medicine, Samsung Medical Center, Seoul, Korea.
Park, Won. Division of Rheumatology, Department of Internal Medicine, Inha University Hospital, Incheon, Korea.
Kim, Hyun Ah. Division of Rheumatology, Department of Internal Medicine, Hallym University Sacred Heart Hospital, Anyang, Korea.
Yoo, Dae-Hyun. Division of Rheumatology, Department of Internal Medicine, Hanyang University Medical Center, Seoul, Korea.
Baek, Han Joo. Division of Rheumatology, Department of Internal Medicine, Gachon Medical School Gil Medical Center, Incheon, Korea.
Lee, Seong Geun. Division of Rheumatology, Department of Internal Medicine, Pusan National University Hospital, Busan, Korea.
Lee, Yun Jong. Division of Rheumatology, Department of Internal Medicine, Seoul National University Bundang Hospital, Seongnam, Korea.
Park, Yong Bum. Division of Rheumatology, Department of Internal Medicine, Yonsei University Severance Hospital, Seoul, Korea.
Shim, Seung-Cheol. Division of Rheumatology, Department of Internal Medicine, Eulji University Hospital, Daejeon, Korea.
Hmamouchi, Ihsane. Rheumatology Department, Faculty of Medicine, Laboratory of Clinical Research and Epidemiology, El Ayachi Hospital, Mohammed V University, Rabat, Morocco.
Song, Yeong Wook. Division of Rheumatology, Department of Internal Medicine, Seoul National University Hospital, Seoul, Korea.
Song, Yeong Wook. Department of Molecular Medicine and Biopharmaceutical Sciences, Medical Research Center, Seoul National University, Seoul, Korea.
Abstract
AIM: We designed this study to evaluate the prevalence of comorbidities, their monitoring states and association with treatment medication in Korean rheumatoid arthritis (RA) patients compared with patients from other countries.

METHODS: We analyzed 1050 RA patients from 11 Korean centers and compared them with 3520 patients from 16 other countries using an international, cross-sectional study evaluating comorbidities of RA (COMORA) database.

RESULTS: Annual evaluations of cardiovascular (CV) risk were less frequently performed in Korea (P = 0.0011). The prevalence of CV-associated morbidity was similar between Korean and international RA patients, although the proportions of current smokers, patients with a family history of CV disease, patients with hyperlipidemia, and patients with Framingham score > 20% were significantly lower in Korea (P < 0.0001 for all), and the antiplatelet agents were more optimally used in Korea (P = 0.0004). Prostate cancer screening was less frequently performed compared to other countries (P < 0.0001). Less than 10% of Korean RA patients were given influenza and pneumococcal vaccinations according to current recommendations.

CONCLUSIONS: There are differences in the prevalence of comorbidities and monitoring states of the risk factors between patients in Korea and in other countries. The prevalence of CV morbidity was similar between the two groups although the prevalence of CV risk factors was significantly low in Korea, suggesting that rheumatologists in Korea need to pay more attention to yearly CV risk monitoring, in addition to the screening of malignancy and vaccination of RA patients against infectious diseases. Copyright © 2017 The Authors. International Journal of Rheumatic Diseases published by Asia Pacific League of Associations for Rheumatology and John Wiley & Sons Australia, Ltd.

Publication Type

Comparative Study. Journal Article. Multicenter Study. Observational Study.

Year of Publication

2018

<143>

Unique Identifier

27180974

Title

Pneumocystis jirovecii pneumonia developed in a patient with rheumatoid arthritis after 14 weeks of iguratimod add-on to treatment with methotrexate and etanercept.

Source

Modern Rheumatology. 28(6):1041-1043, 2018 Nov.

VI 1

Status

MEDLINE

Authors

Arita Y; Taguchi H; Kobayashi M; Tono T; Ohsone Y; Okano Y.

Authors Full Name

Arita, Yuki; Taguchi, Hiroaki; Kobayashi, Mari; Tono, Toshihiro; Ohsone, Yasuo; Okano, Yutaka.

Institution

Arita, Yuki. a Department of Internal Medicine, Kawasaki Municipal Kawasaki Hospital, Kawasaki, Japan and.

Taguchi, Hiroaki. b Center for Arthritis and Rheumatic Diseases, Kawasaki Municipal Kawasaki Hospital, Kawasaki, Japan.

Kobayashi, Mari. a Department of Internal Medicine, Kawasaki Municipal Kawasaki Hospital, Kawasaki, Japan and.

Tono, Toshihiro. b Center for Arthritis and Rheumatic Diseases, Kawasaki Municipal Kawasaki Hospital, Kawasaki, Japan.

Ohsone, Yasuo. b Center for Arthritis and Rheumatic Diseases, Kawasaki Municipal Kawasaki Hospital, Kawasaki, Japan.

Okano, Yutaka. b Center for Arthritis and Rheumatic Diseases, Kawasaki Municipal Kawasaki Hospital, Kawasaki, Japan.

Abstract

A 66-year-old woman who had rheumatoid arthritis and underwent a long-term treatment with methotrexate and etanercept developed Pneumocystis jirovecii pneumonia (PCP) 3 months after iguratimod add-on. Although most rheumatologists might have the impression that iguratimod has less toxicity and immunosuppressive effect compared with methotrexate and biologic disease-modifying antirheumatic drugs, this case suggests that iguratimod may increase the risk of PCP, especially in combination with other drugs.

Publication Type

Case Reports. Journal Article.

Year of Publication

2018

<144>

Unique Identifier

29624215

Title

Current information about vaccination practice in pediatric rheumatic diseases and recommendations for future applications. [Review]

Source

Turkish Journal of Pediatrics. 59(4):357-368, 2017.

VI 1

Status

MEDLINE

Authors

Acari C; Unsal E.

Authors Full Name

Acari, Ceyhun; Unsal, Erbil.

Institution

Acari, Ceyhun. Division of Rheumatology, Department of Pediatrics, Dokuz Eylul University Faculty of Medicine, Izmir, Turkey.

Unsal, Erbil. Division of Rheumatology, Department of Pediatrics, Dokuz Eylul University Faculty of Medicine, Izmir, Turkey.

Abstract

Acari C, Unsal E. Current information about vaccination practice in pediatric rheumatic diseases and recommendations for future applications. Turk J Pediatr 2017; 59: 357-368. Pediatric patients with autoinflammatory or rheumatic diseases are at increased risk of infections; therefore, safe and effective immunizations are crucial in the management of these group of patients. Current aggressive treatments involving the early use of immunosuppressive drugs and biological agents have further increased the susceptibility to infections in this group of patients. Therefore, effective and safe vaccination with adequate serological responses is important. In patients with rheumatic diseases, immunogenicity of a vaccine can differ from the healthy population, because of the disease itself or the immunosuppressive treatment received. Moreover, possible effects of vaccination on the underlying disease should be considered. In general, live attenuated vaccines should not be administered when high-dose immunosuppressive drugs are used for immunosuppressed patients. Inactivated vaccine agents have proven to be generally safe in patients with RD. The immune-modulating of biologic agents effects can last for weeks to months after discontinuation, depending on their half lifes. Also, live virus vaccines are contraindicated during therapy and for weeks to months following discontinuation of the biologics. The aim of this review is to cover the current information about vaccination practice in pediatric rheumatologic diseases and to give recommendations for future applications.

Publication Type

Journal Article. Review.

Year of Publication

2017

<145>

Unique Identifier

29248936

Title

Patients with Coexistence of Circulating Hepatitis B Surface Antigen and Its Antibody May Have a Strong Predisposition to Virus Reactivation During Immunosuppressive Therapy: A Hypothesis.

Source

Medical Science Monitor. 23:5980-5985, 2017 Dec 17.

VI 1

Status

MEDLINE

Authors

Chen YL; Mo YQ; Zheng DH; Ma JD; Jing J; Dai L.

Authors Full Name

Chen, Yu-Lan; Mo, Ying-Qian; Zheng, Dong-Hui; Ma, Jian-Da; Jing, Jun; Dai, Lie.

Institution

Chen, Yu-Lan. Department of Rheumatology, Sun Yat-Sen Memorial Hospital, Sun Yat-Sen University, Guangzhou, Guangdong, China (mainland).

Mo, Ying-Qian. Department of Rheumatology, Sun Yat-Sen Memorial Hospital, Sun Yat-Sen University, Guangzhou, Guangdong, China (mainland).

Zheng, Dong-Hui. Department of Rheumatology, Sun Yat-Sen Memorial Hospital, Sun Yat-Sen University, Guangzhou, Guangdong, China (mainland).

Ma, Jian-Da. Department of Rheumatology, Sun Yat-Sen Memorial Hospital, Sun Yat-Sen University, Guangzhou, Guangdong, China (mainland).

Jing, Jun. Department of Rheumatology, Sun Yat-Sen Memorial Hospital, Sun Yat-Sen University, Guangzhou, Guangdong, China (mainland).

Dai, Lie. Department of Rheumatology, Sun Yat-Sen Memorial Hospital, Sun Yat-Sen University, Guangzhou, Guangdong, China (mainland).

Abstract

Hepatitis B virus (HBV) reactivation is a well-recognized complication in patients who undergo immunosuppressive drug therapy. Although the recommendation of antiviral prophylaxis made by

the American Gastroenterological Association in 2015 focuses on the risk stratification of different immunosuppressive drugs, risk factors for HBV reactivation are also worth identifying in clinical practice. Recent studies have shown that the uncommon serological pattern of coexistent circulating HBV surface antigen (HBsAg) and its antibody (anti-HBs) was associated with double mutations (A1762T/G1764A) in the basal core promoter (BCP) region of the HBV genome, which is critical for HBV replication. Here, we depicted rheumatoid arthritis (RA) patients with coexistent HBsAg and anti-HBs in our medical center, who developed HBV reactivation during immunosuppressive drug therapy. DNA sequencing analysis of the HBV genome revealed triple mutations (A1762T, G1764A, and T1753V) in the BCP region, which could further enhance the ability of HBV replication. Hence, a novel hypothesis is advanced for the first time that patients with coexistent HBsAg and anti-HBs may have a strong predisposition to HBV reactivation due to specific BCP mutations. This hypothesis would, if correct, justify the concurrent detection of HBsAg and anti-HBs in HBV screening in patients with rheumatic diseases and quickly recognize patients with high risk of HBV reactivation. Further controlled studies are needed to confirm this hypothesis.

Publication Type

Journal Article.

Year of Publication

2017

<146>

Unique Identifier

29216931

Title

VISTA deficiency attenuates antibody-induced arthritis and alters macrophage gene expression in response to simulated immune complexes.

Source

Arthritis Research & Therapy. 19(1):270, 2017 Dec 08.

VI 1

Status

MEDLINE

Authors

Ceeraz S; Eszterhas SK; Sergent PA; Armstrong DA; Ashare A; Broughton T; Wang L; Pechenick D; Burns CM; Noelle RJ; Vincenti MP; Fava RA.
Authors Full Name
Ceeraz, Sabrina; Eszterhas, Susan K; Sergent, Petra A; Armstrong, David A; Ashare, Alix; Broughton, Thomas; Wang, Li; Pechenick, Dov; Burns, Christopher M; Noelle, Randolph J; Vincenti, Matthew P; Fava, Roy A.
Institution
Ceeraz, Sabrina. Department of Microbiology and Immunology, Geisel School of Medicine at Dartmouth, Lebanon, NH, 03756, USA.
Ceeraz, Sabrina. Norris Cotton Cancer Center, Dartmouth Hitchcock Medical Center, Lebanon, NH, 03756, USA.
Eszterhas, Susan K. Department of Microbiology and Immunology, Geisel School of Medicine at Dartmouth, Lebanon, NH, 03756, USA.
Eszterhas, Susan K. Department of Veterans Affairs, Research Service, White River Junction, VT, 05009, USA.
Sergent, Petra A. Department of Microbiology and Immunology, Geisel School of Medicine at Dartmouth, Lebanon, NH, 03756, USA.
Sergent, Petra A. Norris Cotton Cancer Center, Dartmouth Hitchcock Medical Center, Lebanon, NH, 03756, USA.
Armstrong, David A. Pulmonary and Critical Care Medicine, Dartmouth-Hitchcock Medical Center, Lebanon, NH, USA.
Armstrong, David A. Department of Medicine, Geisel School of Medicine at Dartmouth, Dartmouth Hitchcock Medical Center, Lebanon, NH, 03756, USA.
Ashare, Alix. Pulmonary and Critical Care Medicine, Dartmouth-Hitchcock Medical Center, Lebanon, NH, USA.
Ashare, Alix. Department of Medicine, Geisel School of Medicine at Dartmouth, Dartmouth Hitchcock Medical Center, Lebanon, NH, 03756, USA.
Broughton, Thomas. Department of Microbiology and Immunology, Geisel School of Medicine at Dartmouth, Lebanon, NH, 03756, USA.
Broughton, Thomas. Norris Cotton Cancer Center, Dartmouth Hitchcock Medical Center, Lebanon, NH, 03756, USA.
Wang, Li. Microbiology and Immunology & Cancer Center Medical College of Wisconsin, Milwaukee, WI, USA.
Pechenick, Dov. ImmuNext INC, 1 Medical Center Drive, Lebanon, NH, 03756, USA.
Burns, Christopher M. Department of Medicine, Section of Rheumatology, Geisel School of Medicine at Dartmouth, Dartmouth-Hitchcock Medical Center, 1 Medical Center Drive, Lebanon, NH, 03756, USA.

Noelle, Randolph J. Department of Microbiology and Immunology, Geisel School of Medicine at Dartmouth, Lebanon, NH, 03756, USA.
Noelle, Randolph J. Norris Cotton Cancer Center, Dartmouth Hitchcock Medical Center, Lebanon, NH, 03756, USA.
Noelle, Randolph J. ImmuNext INC, 1 Medical Center Drive, Lebanon, NH, 03756, USA.
Vincenti, Matthew P. Department of Veterans Affairs, Research Service, White River Junction, VT, 05009, USA.
Vincenti, Matthew P. Department of Medicine, Geisel School of Medicine at Dartmouth, Dartmouth Hitchcock Medical Center, Lebanon, NH, 03756, USA.
Fava, Roy A. Department of Veterans Affairs, Research Service, White River Junction, VT, 05009, USA. roy.a.fava@dartmouth.edu.
Fava, Roy A. Department of Medicine, Geisel School of Medicine at Dartmouth, Dartmouth Hitchcock Medical Center, Lebanon, NH, 03756, USA. roy.a.fava@dartmouth.edu.
Abstract
BACKGROUND: In addition to activated T cells, the immune checkpoint inhibitor "V domain-containing Ig suppressor of T-cell activation" (VISTA) is expressed by myeloid cell types, including macrophages and neutrophils. The importance of VISTA expression by myeloid cells to antibody-induced arthritis and its potential for relevance in human disease was evaluated.

METHODS: VISTA was immunolocalized in normal and arthritic human synovial tissue sections and synovial tissue lysates were subjected to western blot analysis. The collagen antibody-induced arthritis model (CAIA) was performed with DBA/1 J mice treated with antibodies against VISTA and with VISTA-deficient mice (V-KO). Total mRNA from arthritic joints, spleens, and cultured macrophages was analyzed with NanoString arrays. Cytokines secreted by splenic inflammatory macrophages were determined. In-vitro chemotaxis and signal transduction assays were performed with cultured macrophages.

RESULTS: VISTA protein was localized to synovial membrane cells, neutrophils, and scattered cells in lymphocyte-rich foci and was detected by western blot analysis in normal synovium and synovium from rheumatoid arthritis patients. Deficiency of VISTA or treatment of mice with anti-VISTA monoclonal antibodies attenuated CAIA. Joint damage and MMP-3 expression were significantly reduced in V-KO mice. Surface expression of C5a receptor was reduced on monocytes, neutrophils, and cultured macrophages from V-KO. Upon Fc receptor engagement in vitro, gene expression by V-KO macrophages was altered profoundly compared to WT, including a significant induction of IL-1 receptor antagonist (IL1rn).

CONCLUSIONS: VISTA expression supports immune-complex inflammation in CAIA and VISTA is expressed in human synovium. VISTA supports optimal responses to C5a and modulates macrophage responses to immune complexes.

Publication Type

Journal Article.

Year of Publication

2017

<147>

Unique Identifier

29127143

Title

SATB1 Conditional Knockout Results in Sjogren's Syndrome in Mice.

Source

Journal of Immunology. 199(12):4016-4022, 2017 12 15.

VI 1

Status

MEDLINE

Authors

Tanaka Y; Sotome T; Inoue A; Mukozu T; Kuwabara T; Mikami T; Kowhi-Shigematsu T; Kondo M.

Author NameID

Tanaka, Yuriko; ORCID: https://orcid.org/0000-0002-9278-7677

Kuwabara, Taku; ORCID: https://orcid.org/0000-0001-5985-8245

Kondo, Motonari; ORCID: https://orcid.org/0000-0002-1565-8804

Authors Full Name

Tanaka, Yuriko; Sotome, Takehiko; Inoue, Akiko; Mukozu, Takanori; Kuwabara, Taku; Mikami, Tetuo; Kowhi-Shigematsu, Terumi; Kondo, Motonari.

Institution

Tanaka, Yuriko. Department of Molecular Immunology, Toho University School of Medicine, Tokyo 143-8540, Japan.

Sotome, Takehiko. Department of Pediatrics, Toho University Omori Medical Center, Tokyo 143-8541, Japan.

Inoue, Akiko. Department of Otorhinolaryngology, Toho University Omori Medical Center, Tokyo 143-8541, Japan.
Inoue, Akiko. Toho University Graduate School of Medicine, Tokyo 143-8540, Japan.
Mukozu, Takanori. Division of Gastroenterology and Hepatology, Department of Internal Medicine, Toho University Omori Medical Center, Tokyo 143-8541, Japan.
Kuwabara, Taku. Department of Molecular Immunology, Toho University School of Medicine, Tokyo 143-8540, Japan.
Mikami, Tetuo. Department of Pathology, Toho University School of Medicine, Tokyo 143-8540, Japan; and.
Kowhi-Shigematsu, Terumi. Department of Orofacial Sciences, University of California San Francisco School of Dentistry, San Francisco, CA 94143.
Kondo, Motonari. Department of Molecular Immunology, Toho University School of Medicine, Tokyo 143-8540, Japan; motonari.kondo@med.toho-u.ac.jp.
Comments
Comment in (CIN)
Abstract
Sjogren's syndrome (SS) is an autoimmune disease in which exocrine tissues are affected by cellular and humoral immunity. As a result, the salivary and lacrimal glands of patients with SS are damaged, leading to xerostomia (dry mouth) and keratoconjunctivitis sicca (dry eyes). Because experimental approaches to investigate SS pathogenesis in human patients are limited, development of a mouse model is indispensable for understanding the disease. In this study, we show that special AT-rich sequence binding protein-1 conditional knockout (SATB1cKO) mice, in which the SATB1 gene is specifically deleted from hematopoietic cells, develop SS by 4 wk of age, soon after weaning. Female mice presented an earlier onset of the disease than males, suggesting that female SATB1cKO mice are more susceptible to SS. T cell-dominant immune cell infiltration was observed in the salivary glands of 4 wk old SATB1cKO mice, and the frequency of B cells gradually increased as the mice aged. Consistently, levels of anti-SSA and anti-SSB Abs were increased around 8 wk of age, after salivary production reached its lowest level in SATB1cKO mice. These results suggest that SATB1cKO mice can be a novel SS model, in which the progression and characteristics of the disease resemble those of human SS. Copyright © 2017 by The American Association of Immunologists, Inc.
Publication Type
Journal Article. Research Support, Non-U.S. Gov't. Research Support, N.I.H., Extramural.
Year of Publication
2017

<148>

Unique Identifier

29109281

Title

Peptidyl arginine deiminase immunization induces anticitrullinated protein antibodies in mice with particular MHC types.

Source

Proceedings of the National Academy of Sciences of the United States of America. 114(47):E10169-E10177, 2017 11 21.

VI 1

Status

MEDLINE

Authors

Arnoux F; Mariot C; Peen E; Lambert NC; Balandraud N; Roudier J; Auger I.

Authors Full Name

Arnoux, Fanny; Mariot, Charlotte; Peen, Elisa; Lambert, Nathalie C; Balandraud, Nathalie; Roudier, Jean; Auger, Isabelle.

Institution

Arnoux, Fanny. INSERM UMR 1097, Aix Marseille University, 13009 Marseille, France.

Mariot, Charlotte. INSERM UMR 1097, Aix Marseille University, 13009 Marseille, France.

Peen, Elisa. INSERM UMR 1097, Aix Marseille University, 13009 Marseille, France.

Lambert, Nathalie C. INSERM UMR 1097, Aix Marseille University, 13009 Marseille, France.

Balandraud, Nathalie. INSERM UMR 1097, Aix Marseille University, 13009 Marseille, France.

Balandraud, Nathalie. Service de Rhumatologie, Hopital Sainte Marguerite, Assistance Publique Hopitaux de Marseille, 13009 Marseille, France.

Roudier, Jean. INSERM UMR 1097, Aix Marseille University, 13009 Marseille, France; jean.roudier@inserm.fr.

Roudier, Jean. Service de Rhumatologie, Hopital Sainte Marguerite, Assistance Publique Hopitaux de Marseille, 13009 Marseille, France.

Auger, Isabelle. INSERM UMR 1097, Aix Marseille University, 13009 Marseille, France.

Abstract

Autoantibodies to citrullinated proteins (ACPAs) are present in two-thirds of patients with rheumatoid arthritis (RA). ACPAs are produced in the absence of identified T cell responses for each citrullinated protein. Peptidyl arginine deiminase 4 (PAD4), which binds proteins and

citrullinates them, is the target of autoantibodies in early RA. This suggests a model for the emergence of ACPAs in the absence of detectable T cells specific for citrullinated antigens: ACPAs could arise because PADs are recognized by T cells, which help the production of autoantibodies to proteins bound by PADs, according to a "hapten/carrier" model. Here, we tested this model in normal mice. C3H are healthy mice whose IEbetak chain is highly homologous to the beta1 chain HLA-DRB1*04:01, the allele most strongly associated with RA in humans. C3H mice immunized with PADs developed antibodies and T cells to PAD and IgG antibodies to citrullinated fibrinogen peptides, in the absence of a T cell response to fibrinogen. To analyze the MHC background effect on hapten/carrier immunization, we immunized DBA/2 mice (whose IEbetad chain is similar to that of HLA-DRB1*04:02, an HLA-DR4 subtype not associated with RA). DBA/2 mice failed to develop antibodies to citrullinated fibrinogen peptides. Thus, T cell immunization to PAD proteins may trigger ACPAs through a hapten/carrier mechanism. This may constitute the basis for a new mouse model of ACPA-positive RA.
Copyright © 2017 the Author(s). Published by PNAS.
Publication Type
  Journal Article. Research Support, Non-U.S. Gov't.
Year of Publication
  2017

<149>
Unique Identifier
  29071482
Title
  [Nontuberculous mycobacterial infections]. [Review] [German]
Source
  Zeitschrift fur Rheumatologie. 76(9):752-760, 2017 Nov.
VI 1
Status
  MEDLINE
Authors
  Nowag A; Platten M; Plum G; Hartmann P.
Authors Full Name
  Nowag, A; Platten, M; Plum, G; Hartmann, P.

Institution
Nowag, A. Klinische Infektiologie, Labor Dr. Wisplinghoff, Horbeller Strase 18-20, 50858, Koln, Deutschland.
Nowag, A. Institut fur Medizinische Mikrobiologie, Immunologie und Hygiene (IMMIH), Uniklinik Koln, Koln, Deutschland.
Platten, M. Klinik I fur Innere Medizin, Uniklinik Koln, Koln, Deutschland.
Platten, M. Deutsches Zentrum fur Infektionsforschung, Standort Bonn-Koln, Bonn-Koln, Deutschland.
Plum, G. Institut fur Medizinische Mikrobiologie, Immunologie und Hygiene (IMMIH), Uniklinik Koln, Koln, Deutschland.
Hartmann, P. Klinische Infektiologie, Labor Dr. Wisplinghoff, Horbeller Strase 18-20, 50858, Koln, Deutschland. p.hartmann@wisplinghoff.de.
Hartmann, P. Institut fur Medizinische Mikrobiologie, Immunologie und Hygiene (IMMIH), Uniklinik Koln, Koln, Deutschland. p.hartmann@wisplinghoff.de.
Hartmann, P. Deutsches Zentrum fur Infektionsforschung, Standort Bonn-Koln, Bonn-Koln, Deutschland. p.hartmann@wisplinghoff.de.
Abstract
Nontuberculous mycobacterial (NTM) are found ubiquitously in the environment and are usually of low pathogenicity. Infection occurs via inhalation of aerosols, and some species may cause severe infections. The incidence of NTM infections is rising worldwide. The risk of developing NTM disease depends on the susceptibility of the host as well as the frequency and duration of exposure. In addition to congenital immune deficiencies and immunosuppressive therapy, structural lung and systemic diseases, including rheumatoid arthritis (RA), are associated with an increased risk for NTM infections. The immune response to NTM is complex and relies on the interplay between professional phagocytes and lymphoid cells. This interplay is concerted by three key cytokines: interleukin-12 (IL-12), tumor necrosis factor-alpha (TNF-alpha), and interferon-gamma (IFN-gamma). Targeted immunotherapies, e. g., treatment with TNF inhibitors, interfere with these essential pathways and increase the risk of NTM infection significantly. This review focuses on the relationship between the immune response to NTM and intrinsic and iatrogenic dispositions for NTM infection, with an emphasis on RA.
Publication Type
Journal Article. Review.
Year of Publication
2017

<150>
Unique Identifier
  29067885
Title
  A review article on biosimilar infliximab SB2 in the treatment of rheumatoid arthritis. [Review]
Source
  Immunotherapy. 9(14):1133-1142, 2017 11.
VI 1
Status
  MEDLINE
Authors
  Bellinvia S; Ashraf M; Polosa R; Edwards C.
Authors Full Name
  Bellinvia, Salvatore; Ashraf, Madiha; Polosa, Riccardo; Edwards, Christopher.
Institution
  Bellinvia, Salvatore. Department of Rheumatology & NIHR Wellcome Trust Clinical Research Facility, University Hospital Southampton NHS Foundation Trust, Southampton, Tremona Road, SO16 6YD, UK.
  Bellinvia, Salvatore. Institute of Internal & Emergency Medicine, Rheumatology Outpatient Clinic, Teaching Hospital "Policlinico - V. Emanuele", University of Catania, Via Santa Sofia 78, 95123, Italy.
  Ashraf, Madiha. Department of Rheumatology & NIHR Wellcome Trust Clinical Research Facility, University Hospital Southampton NHS Foundation Trust, Southampton, Tremona Road, SO16 6YD, UK.
  Polosa, Riccardo. Institute of Internal & Emergency Medicine, Rheumatology Outpatient Clinic, Teaching Hospital "Policlinico - V. Emanuele", University of Catania, Via Santa Sofia 78, 95123, Italy.
  Edwards, Christopher. Department of Rheumatology & NIHR Wellcome Trust Clinical Research Facility, University Hospital Southampton NHS Foundation Trust, Southampton, Tremona Road, SO16 6YD, UK.
Abstract
  TNF inhibition has had a major impact as an approach for treating rheumatoid arthritis and a series of biologic agents directed against TNF have been developed for clinical use. Infliximab, a chimeric monoclonal antibody against soluble and membrane-bound TNF-alpha, was the biopharmaceutical to lead this 'biologics revolution'. However, with expiration of patent protection

of the originator medicinal product, biosimilar versions of infliximab have been developed through biosimilarity studies and randomized controlled trials aiming to assess pharmacokinetic, pharmacodynamic and clinical equivalence to their originator (reference product) in patients with moderate-to-severe disease activity. This review summarizes the clinical development of SB2, a biosimilar of infliximab, in rheumatoid arthritis.
Publication Type
  Journal Article. Review.
Year of Publication
  2017

<151>
Unique Identifier
  28967792
Title
  Abatacept and granulocyte-colony stimulating factor in a patient with rheumatoid arthritis and neutropenia.
Source
  Immunotherapy. 9(13):1055-1059, 2017 10.
VI 1
Status
  MEDLINE
Authors
  Priora M; Parisi S; Scarati M; Borrelli R; Peroni CL; Fusaro E.
Authors Full Name
  Priora, Marta; Parisi, Simone; Scarati, Marco; Borrelli, Richard; Peroni, Clara Lisa; Fusaro, Enrico.
Institution
  Priora, Marta. Rheumatology Department, A.O.U. Citta della Salute e della Scienza, Corso Bramante 88, 10124 Turin.
  Parisi, Simone. Rheumatology Department, A.O.U. Citta della Salute e della Scienza, Corso Bramante 88, 10124 Turin.
  Scarati, Marco. Rheumatology Department, A.O.U. Citta della Salute e della Scienza, Corso Bramante 88, 10124 Turin.

Borrelli, Richard. Rheumatology Department, A.O.U. Citta della Salute e della Scienza, Corso Bramante 88, 10124 Turin.
Peroni, Clara Lisa. Rheumatology Department, A.O.U. Citta della Salute e della Scienza, Corso Bramante 88, 10124 Turin.
Fusaro, Enrico. Rheumatology Department, A.O.U. Citta della Salute e della Scienza, Corso Bramante 88, 10124 Turin.
Abstract
Neutropenia in patients with inflammatory diseases increases the risk of infection due to the disease itself and the related immunosuppressive treatments. We report the case of a 54-year-old female with rheumatoid arthritis and following development of chronic neutropenia. All investigations excluded pathogenic relations with drugs and/or other clinical situations; the gravity of neutropenia required a treatment with G-CSF and the increased articular inflammatory activity justified a biologic-therapy, abatacept (CTLA4 inhibitors). The juxtaposition of immunostimulants and immunosuppressors led to great effectiveness for both hematological and rheumatic issues. To date, while some biologic drugs (TNF, IL6R and CD20 inhibitors) have reported relations with neutropenia, no such relevance subsists for Abatacept. Our case reports the experience of the safe effective use of abatacept and G-CSF for 8 years.
Publication Type
Case Reports. Journal Article.
Year of Publication
2017

<152>
Unique Identifier
28966211
Title
Initial Serological Response after Prime-boost Pneumococcal Vaccination in Rheumatoid Arthritis Patients: Results of a Randomized Controlled Trial.
Source
Journal of Rheumatology. 44(12):1794-1803, 2017 Dec.
VI 1
Status
MEDLINE

Authors
Nguyen MTT; Lindegaard H; Hendricks O; Jorgensen CS; Kantso B; Friis-Moller N.
Authors Full Name
Nguyen, Mai T T; Lindegaard, Hanne; Hendricks, Oliver; Jorgensen, Charlotte Svaerke; Kantso, Bjorn; Friis-Moller, Nina.
Institution
Nguyen, Mai T T. From the Department of Infectious Diseases, and Department of Rheumatology, and Odense Patient data Explorative Network (OPEN), Odense University Hospital; Institute of Clinical Research, University of Southern Denmark; Institute of Regional Research, University of Southern Denmark, Odense; Department of Rheumatology, King Christian Xth Hospital for Rheumatic Disease, Graasten; Department of Microbiology and Virology, Statens Serum Institut, Copenhagen; Unit of Infectious Diseases, Department of Internal Medicine, Zealand University Hospital, Roskilde, Denmark. mai.nguyen@rsyd.dk.
Nguyen, Mai T T. M.T. Nguyen, MD, Department of Infectious Diseases, Odense University Hospital, and Institute of Clinical Research, University of Southern Denmark, and OPEN, Odense University Hospital; H. Lindegaard, MD, PhD, Institute of Clinical Research, University of Southern Denmark, and Department of Rheumatology, Odense University Hospital; O. Hendricks, MD, PhD, Department of Rheumatology, King Christian Xth Hospital for Rheumatic Disease, and Institute of Regional Research, University of Southern Denmark; C.S. Jorgensen, MSc, PhD, Department of Microbiology and Virology, Statens Serum Institut; B. Kantso, MSc, PhD, Department of Microbiology and Virology, Statens Serum Institut; N. Friis-Moller, MD, DMSc, Department of Infectious Diseases, Odense University Hospital, and Institute of Clinical Research, University of Southern Denmark, and Unit of Infectious Diseases, Department of Internal Medicine, Zealand University Hospital. mai.nguyen@rsyd.dk.
Lindegaard, Hanne. From the Department of Infectious Diseases, and Department of Rheumatology, and Odense Patient data Explorative Network (OPEN), Odense University Hospital; Institute of Clinical Research, University of Southern Denmark; Institute of Regional Research, University of Southern Denmark, Odense; Department of Rheumatology, King Christian Xth Hospital for Rheumatic Disease, Graasten; Department of Microbiology and Virology, Statens Serum Institut, Copenhagen; Unit of Infectious Diseases, Department of Internal Medicine, Zealand University Hospital, Roskilde, Denmark.
Lindegaard, Hanne. M.T. Nguyen, MD, Department of Infectious Diseases, Odense University Hospital, and Institute of Clinical Research, University of Southern Denmark, and OPEN, Odense University Hospital; H. Lindegaard, MD, PhD, Institute of Clinical Research, University of Southern Denmark, and Department of Rheumatology, Odense University Hospital; O. Hendricks, MD, PhD, Department of Rheumatology, King Christian Xth Hospital for Rheumatic Disease, and Institute of Regional Research, University of Southern Denmark; C.S. Jorgensen, MSc, PhD,

Department of Microbiology and Virology, Statens Serum Institut; B. Kantso, MSc, PhD, Department of Microbiology and Virology, Statens Serum Institut; N. Friis-Moller, MD, DMSc, Department of Infectious Diseases, Odense University Hospital, and Institute of Clinical Research, University of Southern Denmark, and Unit of Infectious Diseases, Department of Internal Medicine, Zealand University Hospital.

Hendricks, Oliver. From the Department of Infectious Diseases, and Department of Rheumatology, and Odense Patient data Explorative Network (OPEN), Odense University Hospital; Institute of Clinical Research, University of Southern Denmark; Institute of Regional Research, University of Southern Denmark, Odense; Department of Rheumatology, King Christian Xth Hospital for Rheumatic Disease, Graasten; Department of Microbiology and Virology, Statens Serum Institut, Copenhagen; Unit of Infectious Diseases, Department of Internal Medicine, Zealand University Hospital, Roskilde, Denmark.

Hendricks, Oliver. M.T. Nguyen, MD, Department of Infectious Diseases, Odense University Hospital, and Institute of Clinical Research, University of Southern Denmark, and OPEN, Odense University Hospital; H. Lindegaard, MD, PhD, Institute of Clinical Research, University of Southern Denmark, and Department of Rheumatology, Odense University Hospital; O. Hendricks, MD, PhD, Department of Rheumatology, King Christian Xth Hospital for Rheumatic Disease, and Institute of Regional Research, University of Southern Denmark; C.S. Jorgensen, MSc, PhD, Department of Microbiology and Virology, Statens Serum Institut; B. Kantso, MSc, PhD, Department of Microbiology and Virology, Statens Serum Institut; N. Friis-Moller, MD, DMSc, Department of Infectious Diseases, Odense University Hospital, and Institute of Clinical Research, University of Southern Denmark, and Unit of Infectious Diseases, Department of Internal Medicine, Zealand University Hospital.

Jorgensen, Charlotte Svaerke. From the Department of Infectious Diseases, and Department of Rheumatology, and Odense Patient data Explorative Network (OPEN), Odense University Hospital; Institute of Clinical Research, University of Southern Denmark; Institute of Regional Research, University of Southern Denmark, Odense; Department of Rheumatology, King Christian Xth Hospital for Rheumatic Disease, Graasten; Department of Microbiology and Virology, Statens Serum Institut, Copenhagen; Unit of Infectious Diseases, Department of Internal Medicine, Zealand University Hospital, Roskilde, Denmark.

Jorgensen, Charlotte Svaerke. M.T. Nguyen, MD, Department of Infectious Diseases, Odense University Hospital, and Institute of Clinical Research, University of Southern Denmark, and OPEN, Odense University Hospital; H. Lindegaard, MD, PhD, Institute of Clinical Research, University of Southern Denmark, and Department of Rheumatology, Odense University Hospital; O. Hendricks, MD, PhD, Department of Rheumatology, King Christian Xth Hospital for Rheumatic Disease, and Institute of Regional Research, University of Southern Denmark; C.S. Jorgensen, MSc, PhD, Department of Microbiology and Virology, Statens Serum Institut; B. Kantso, MSc,

PhD, Department of Microbiology and Virology, Statens Serum Institut; N. Friis-Moller, MD, DMSc, Department of Infectious Diseases, Odense University Hospital, and Institute of Clinical Research, University of Southern Denmark, and Unit of Infectious Diseases, Department of Internal Medicine, Zealand University Hospital.

Kantso, Bjorn. From the Department of Infectious Diseases, and Department of Rheumatology, and Odense Patient data Explorative Network (OPEN), Odense University Hospital; Institute of Clinical Research, University of Southern Denmark; Institute of Regional Research, University of Southern Denmark, Odense; Department of Rheumatology, King Christian Xth Hospital for Rheumatic Disease, Graasten; Department of Microbiology and Virology, Statens Serum Institut, Copenhagen; Unit of Infectious Diseases, Department of Internal Medicine, Zealand University Hospital, Roskilde, Denmark.

Kantso, Bjorn. M.T. Nguyen, MD, Department of Infectious Diseases, Odense University Hospital, and Institute of Clinical Research, University of Southern Denmark, and OPEN, Odense University Hospital; H. Lindegaard, MD, PhD, Institute of Clinical Research, University of Southern Denmark, and Department of Rheumatology, Odense University Hospital; O. Hendricks, MD, PhD, Department of Rheumatology, King Christian Xth Hospital for Rheumatic Disease, and Institute of Regional Research, University of Southern Denmark; C.S. Jorgensen, MSc, PhD, Department of Microbiology and Virology, Statens Serum Institut; B. Kantso, MSc, PhD, Department of Microbiology and Virology, Statens Serum Institut; N. Friis-Moller, MD, DMSc, Department of Infectious Diseases, Odense University Hospital, and Institute of Clinical Research, University of Southern Denmark, and Unit of Infectious Diseases, Department of Internal Medicine, Zealand University Hospital.

Friis-Moller, Nina. From the Department of Infectious Diseases, and Department of Rheumatology, and Odense Patient data Explorative Network (OPEN), Odense University Hospital; Institute of Clinical Research, University of Southern Denmark; Institute of Regional Research, University of Southern Denmark, Odense; Department of Rheumatology, King Christian Xth Hospital for Rheumatic Disease, Graasten; Department of Microbiology and Virology, Statens Serum Institut, Copenhagen; Unit of Infectious Diseases, Department of Internal Medicine, Zealand University Hospital, Roskilde, Denmark.

Friis-Moller, Nina. M.T. Nguyen, MD, Department of Infectious Diseases, Odense University Hospital, and Institute of Clinical Research, University of Southern Denmark, and OPEN, Odense University Hospital; H. Lindegaard, MD, PhD, Institute of Clinical Research, University of Southern Denmark, and Department of Rheumatology, Odense University Hospital; O. Hendricks, MD, PhD, Department of Rheumatology, King Christian Xth Hospital for Rheumatic Disease, and Institute of Regional Research, University of Southern Denmark; C.S. Jorgensen, MSc, PhD, Department of Microbiology and Virology, Statens Serum Institut; B. Kantso, MSc, PhD, Department of Microbiology and Virology, Statens Serum Institut; N. Friis-Moller, MD, DMSc,

Department of Infectious Diseases, Odense University Hospital, and Institute of Clinical Research, University of Southern Denmark, and Unit of Infectious Diseases, Department of Internal Medicine, Zealand University Hospital.
Abstract

OBJECTIVE: To evaluate the initial serological responses to pneumococcal vaccination with the 13-valent protein-conjugated pneumococcal vaccine (PCV13) followed by the 23-valent polysaccharide pneumococcal vaccine (PPV23) among patients with rheumatoid arthritis (RA) treated with biological disease-modifying antirheumatic drugs (bDMARD) according to dosing and intervals between immunizations.

METHODS: Investigator-initiated clinical trial. Patients with RA receiving bDMARD were randomized (1:1:1) to immunization with single dose PCV13 followed by PPV23 after 16 or 24 weeks, or double dose PCV13 followed by PPV23 after 16 weeks. A comparison group of patients with RA treated with conventional synthetic (cs)DMARD received single dose PCV13 followed by PPV23 16 weeks later. Pneumococcal antibodies were collected before and 4 weeks after each vaccination. The primary endpoint was the proportion of participants responding to >= 6/12 pneumococcal serotypes 4 weeks after both vaccinations.

RESULTS: Sixty-five participants receiving bDMARD and 35 participants receiving csDMARD were included. After PPV23 vaccination, 87% (95% CI 0.76-0.94) and 94% (95% CI 0.77-0.99), respectively, of participants treated with bDMARD and csDMARD had reached the primary endpoint. There was no significant difference in primary endpoint between the 3 randomization arms. The response for rituximab-treated participants was 25% compared to >= 89% in participants treated with bDMARD with other mode of action.

CONCLUSION: The early serological response to prime-boost vaccination with PCV13 followed by PPV23 was very similar among participants receiving bDMARD and csDMARD. However, notable differences in response were observed according to individual bDMARD. It is important to consider the RA treatment when planning pneumococcal vaccination in patients with RA.
Publication Type

Clinical Trial, Phase III. Journal Article. Randomized Controlled Trial.

Year of Publication

2017

<153>

Unique Identifier

28959043

Title

Inflammation in gout: mechanisms and therapeutic targets. [Review]

Source

Nature Reviews Rheumatology. 13(11):639-647, 2017 Nov.

VI 1

Status

MEDLINE

Authors

So AK; Martinon F.

Authors Full Name

So, Alexander K; Martinon, Fabio.

Institution

So, Alexander K. Service of Rheumatology, Centre Hospitalier Universitaire Vaudois and University of Lausanne, Avenue Pierre Decker 4, 1011 Lausanne, Switzerland.

Martinon, Fabio. Department of Biochemistry, University of Lausanne, 155 Chemin des Boveresses, 1066 Epalinges, Switzerland.

Abstract

The acute symptoms of gout are triggered by the inflammatory response to monosodium urate crystals, mediated principally by macrophages and neutrophils. Innate immune pathways are of key importance in the pathogenesis of gout, in particular the activation of the NLRP3 inflammasome, which leads to the release of IL-1beta and other pro-inflammatory cytokines. The orchestration of this pro-inflammatory cascade involves multiple intracellular and extracellular receptors and enzymes interacting with environmental influences that modulate the inflammatory state. Furthermore, the resolution of inflammation in gout is becoming better understood. This Review highlights recent advances in our understanding of both positive and negative regulatory pathways, as well as the genetic and environmental factors that modulate the inflammatory response. Some of these pathways can be manipulated and present novel therapeutic opportunities for the treatment of acute gout attacks.

Publication Type

Journal Article. Review.

Year of Publication

2017

<154>

Unique Identifier

28942223

Title

Baicalin suppresses IL-1beta-induced expression of inflammatory cytokines via blocking NF-kappaB in human osteoarthritis chondrocytes and shows protective effect in mice osteoarthritis models.

Source

International Immunopharmacology. 52:218-226, 2017 Nov.

VI 1

Status

MEDLINE

Authors

Chen C; Zhang C; Cai L; Xie H; Hu W; Wang T; Lu D; Chen H.

Authors Full Name

Chen, Chunhui; Zhang, Chuanxu; Cai, Leyi; Xie, Huanguang; Hu, Wei; Wang, Te; Lu, Di; Chen, Hua.

Institution

Chen, Chunhui. Department of Orthopaedic Surgery, The Second Affiliated Hospital and Yuying Children's Hospital of Wenzhou Medical University, Wenzhou 325000, China.

Zhang, Chuanxu. Department of Orthopaedic Surgery, The Second Affiliated Hospital and Yuying Children's Hospital of Wenzhou Medical University, Wenzhou 325000, China.

Cai, Leyi. Department of Orthopaedic Surgery, The Second Affiliated Hospital and Yuying Children's Hospital of Wenzhou Medical University, Wenzhou 325000, China.

Xie, Huanguang. Department of Orthopaedic Surgery, The Second Affiliated Hospital and Yuying Children's Hospital of Wenzhou Medical University, Wenzhou 325000, China.

Hu, Wei. Department of Orthopaedic Surgery, The Second Affiliated Hospital and Yuying Children's Hospital of Wenzhou Medical University, Wenzhou 325000, China.

Wang, Te. Department of Orthopaedic Surgery, The Second Affiliated Hospital and Yuying Children's Hospital of Wenzhou Medical University, Wenzhou 325000, China.

Lu, Di. Department of Orthopaedic Surgery, The Second Affiliated Hospital and Yuying Children's Hospital of Wenzhou Medical University, Wenzhou 325000, China.

Chen, Hua. Department of Orthopaedic Surgery, The Second Affiliated Hospital and Yuying Children's Hospital of Wenzhou Medical University, Wenzhou 325000, China. Electronic address: chenhuafey@126.com.
Abstract
Osteoarthritis (OA) is a degenerative joint disease with an inflammatory component that drives the degradation of cartilage extracellular matrix. Baicalin, a predominant flavonoid isolated from the dry root of Scutellaria baicalensis Georgi, has been reported to have anti-inflammatory effects. However, the anti-inflammatory effects of baicalin on OA have not been reported. Our study aimed to investigate the effect of baicalin on OA both in vitro and in vivo. In vitro, human OA chondrocytes were pretreated with baicalin (10, 50, 100muM) for 2h and subsequently stimulated with IL-1beta for 24h. Production of NO and PGE2 were evaluated by the Griess reaction and ELISAs. The mRNA expression of COX-2, iNOS, MMP-3, MMP-13, ADAMTS-5, aggrecan and collagen-II were measured by real-time PCR. The protein expression of COX-2, iNOS, MMP-3, MMP-13, ADAMTS-5, p65, p-p65, IkappaBalpha and p-IkappaBalpha was detected by Western blot. The protein expression of collagen-II was evaluated by immunofluorescence. Luciferase activity assay was used to assess the relative activity of NF-kB. In vivo, the severity of OA was determined by histological analysis. We found that baicalin significantly inhibited the IL-1beta-induced production of NO and PGE2, expression of COX-2, iNOS, MMP-3, MMP-13 and ADAMTS-5 and degradation of aggrecan and collagen-II. Furthermore, baicalin dramatically suppressed IL-1beta-stimulated NF-kappaB activation. In vivo, treatment of baicalin not only prevented the destruction of cartilage but also relieved synovitis in mice OA models. Taken together, these results suggest that baicalin may be a potential agent in the treatment of OA. Copyright © 2017 Elsevier B.V. All rights reserved.
Publication Type
Journal Article.
Year of Publication
2017

<155>
Unique Identifier
28915439
Title

Alpha-Mangostin suppresses interleukin-1beta-induced apoptosis in rat chondrocytes by inhibiting the NF-kappaB signaling pathway and delays the progression of osteoarthritis in a rat model.

Source

International Immunopharmacology. 52:156-162, 2017 Nov.

VI 1

Status

MEDLINE

Authors

Pan T; Chen R; Wu D; Cai N; Shi X; Li B; Pan J.

Authors Full Name

Pan, Tianlong; Chen, Rong; Wu, Dengying; Cai, Ningyu; Shi, Xuchao; Li, Bin; Pan, Jun.

Institution

Pan, Tianlong. Department of Orthopaedics, The Second Affiliated Hospital and Yuying Children's Hospital of Wenzhou Medical University, 109 Xue Yuan Xi Road, Wenzhou, Zhejiang 325000, China.

Chen, Rong. Department of Orthopaedics, The Second Affiliated Hospital and Yuying Children's Hospital of Wenzhou Medical University, 109 Xue Yuan Xi Road, Wenzhou, Zhejiang 325000, China.

Wu, Dengying. Department of Orthopaedics, The Second Affiliated Hospital and Yuying Children's Hospital of Wenzhou Medical University, 109 Xue Yuan Xi Road, Wenzhou, Zhejiang 325000, China.

Cai, Ningyu. Department of Orthopaedics, The Second Affiliated Hospital and Yuying Children's Hospital of Wenzhou Medical University, 109 Xue Yuan Xi Road, Wenzhou, Zhejiang 325000, China.

Shi, Xuchao. Department of Orthopaedics, The Second Affiliated Hospital and Yuying Children's Hospital of Wenzhou Medical University, 109 Xue Yuan Xi Road, Wenzhou, Zhejiang 325000, China.

Li, Bin. Department of Orthopaedics, The Second Affiliated Hospital and Yuying Children's Hospital of Wenzhou Medical University, 109 Xue Yuan Xi Road, Wenzhou, Zhejiang 325000, China.

Pan, Jun. Department of Orthopaedics, The Second Affiliated Hospital and Yuying Children's Hospital of Wenzhou Medical University, 109 Xue Yuan Xi Road, Wenzhou, Zhejiang 325000, China. Electronic address: panjun@wmu.edu.cn.

Comments

Erratum in (EIN)

Abstract

Osteoarthritis (OA) is a chronic degenerative joint disease that is characterized by progressive joint dysfunction and pain. Apoptosis and catabolism in chondrocytes play critical roles in the development of OA. Alpha-Mangostin (alpha-MG), one of the main components of the mangosteen, has been reported to have anti-apoptotic, anti-inflammatory and antioxidant effects. We investigated the therapeutic effects of alpha-MG on OA through experiments on rat chondrocytes in vitro and in a rat model of OA induced by destabilization of the medial meniscus (DMM). In vitro, we provided experimental evidence that alpha-MG inhibits the expression of MMP-13 and ADAMTs-5, and promotes the expression of SOX-9 in rat chondrocytes stimulated with interleukin-1beta (IL-1beta). In addition, we also found that alpha-MG can inhibit the expression of pro-apoptotic proteins such as Bax, Cyto-c, and C-caspase3, and increase the expression of the anti-apoptotic protein Bcl-2. These changes may be related to an alpha-MG induced inhibition of the IL-1beta-induced activation of the NF-kB signaling pathway. In vivo, we also found that alpha-MG can limit the development of OA in rat models. The above results show that alpha-MG has a potential therapeutic effect on OA, and that this effect may be achieved by inhibiting the mitochondrial apoptosis of chondrocytes induced by an activation of the NF-kB pathway. Copyright © 2017 Elsevier B.V. All rights reserved.

Publication Type

Journal Article.

Year of Publication

2017

<156>

Unique Identifier

28905860

Title

Rheumatoid arthritis: Reducing the risk of herpes zoster.

Source

Nature Reviews Rheumatology. 13(11):634, 2017 11.

VI 1

Status

MEDLINE

Authors

McHugh J.

Authors Full Name
McHugh, Jessica.
Comments
Comment on (CON)
Comment on (CON)
Publication Type
Journal Article. Comment.
Year of Publication
2017

<157>
Unique Identifier
28877634
Title
Certolizumab, an anti-TNF safe during pregancy? The CRIB Study results: an interview with Professor Xavier Mariette.
Source
Immunotherapy. 9(10):793-795, 2017 09.
VI 1
Status
MEDLINE
Authors
Mariette X.
Authors Full Name
Mariette, Xavier.
Institution
Mariette, Xavier. Universite Paris-Sud, Hopitaux Universitaires Paris-Sud, Assistance Publique-Hopitaux de Paris, Paris, France.
Abstract
Professor Xavier Mariette, MD, PhD, has served as the Head of the Rheumatology Department of Bicetre Hospital, Paris-Sud University since 1999, a role he took following 10 years of practice of clinical immunology. Professor Mariette has initiated a number of clinical research studies on biotherapies in autoimmune diseases. He is the head of the French RATIO (Research Axed on

Tolerance of Biotherapy) observatory, collecting specific rare serious adverse events in patients treated with anti-TNF. He initiated the French AIR (Autoimmunity and Rituximab) and ORA (Orencia and Rheumatoid arthritis) registries of patients with autoimmune diseases treated with rituximab and abatacept. He initiated clinical trials in Sjogren's syndrome with infliximab, hydroxychloroquine and belimumab. Professor Mariette is involved in basic research, leading a group working on pathogeny of Sjogren's syndrome, relationships between innate immunity and B-cell activation in autoimmunity and the relationships between autoimmunity and lymphoma. Professor Mariette is also very interested in new ways of teaching. In 2007, he participated with other European Experts in the creation of the EULAR Web Course of Rheumatology in 2007. Professor Mariette has been the President of the Scientific Committee of the EULAR meeting, which took place in Berlin in 2012 and is in 2016 the elect Chair of the EULAR standing committee on investigative rheumatology. Professor Mariette is co-author of more than 430 publications referenced in PubMed with an H-index of 61.

Publication Type

Autobiography. Historical Article. Interview.

Year of Publication

2017

<158>

Unique Identifier

28845573

Title

Editorial: Herpes Zoster: Fear the Infection, Value the Solution.

Source

Arthritis & Rheumatology. 69(10):1917-1920, 2017 10.

VI 1

Status

MEDLINE

Authors

Cush JJ.

Authors Full Name

Cush, John J.

Institution

Cush, John J. Baylor Scott & White Research Institute and Baylor University Medical Center, Dallas, Texas.
Comments
Comment on (CON)
Publication Type
Editorial. Comment.
Year of Publication
2017

<159>
Unique Identifier
28834574
Title
B cell activating factor (BAFF) and BAFF receptors: fakes and facts. [Review]
Source
Clinical & Experimental Immunology. 190(3):291-292, 2017 12.
VI 1
Status
MEDLINE
Authors
Ferraccioli G; Gremese E.
Author NameID
Ferraccioli, G; ORCID: https://orcid.org/0000-0002-6884-4301
Authors Full Name
Ferraccioli, G; Gremese, E.
Institution
Ferraccioli, G. Institute of Rheumatology, School of Medicine, Catholic University of the Sacred Heart, Rome, Italy.
Gremese, E. Institute of Rheumatology, School of Medicine, Catholic University of the Sacred Heart, Rome, Italy.
Abstract
Analysis of B cell activating factor (BAFF) receptors before and after B cell depletion therapy (BCDT) might offer a clue to the understanding of whether some B cell subsets may represent

useful biomarkers of biological and clinical responses. Among the BAFF receptors in a cohort of rheumatoid arthritis (RA) patients, the AA have shown, by fluorescence activated cell sorter (FACS) analysis of median fluorescence intensity (MFI), that transmembrane activator and calcium-modulating cyclophilin ligand interactor (TACI) and B cell maturation antigen (BCMA) do not change, whereas the most important, BAFF receptor 3 (BR3), appears to be decreased before as well as after BCDT in all B cell subsets but not in plasmablasts, the most important subset, depleted by BCDT. Copyright © 2017 British Society for Immunology.

Publication Type

Journal Article. Review.

Year of Publication

2017

<160>

Unique Identifier

28817445

Title

The safety of pembrolizumab in metastatic melanoma and rheumatoid arthritis.

Source

Melanoma Research. 27(5):519-523, 2017 10.

VI 1

Status

MEDLINE

Authors

Puri A; Homsi J.

Authors Full Name

Puri, Akshjot; Homsi, Jade.

Institution

Puri, Akshjot. aDepartment of Internal Medicine, Banner University Medical Center, Phoenix bDepartment of Medical Oncology, Banner MD Anderson Cancer Center, Gilbert, Arizona.

Abstract

Immunotherapy has been in use for the treatment of melanoma since a very long time, but only recently have the cytotoxic T-lymphocyte antigen-4 (CTLA-4) antibody ipilimumab and programmed cell death-1 inhibitors such as nivolimumab and pembrolizumab been shown to

induce marked improvements in survival in patients with metastatic melanoma. An important concern arises in terms of the safety of the use of these agents in patients with autoimmune diseases, solid organ transplant recipients on immunosuppression, patients with a history of previous hepatitis B or C, and patients with HIV infections as these patients were excluded from pivotal immunotherapy studies. Here, we report on the safety and efficacy of pembrolizumab in a melanoma patient with multiple medical problems including poorly controlled rheumatoid arthritis and we review the available literature on the use of immunotherapy and autoimmune diseases. The weight of evidence suggests that these patients should be offered the opportunity to benefit from immune check point inhibitors, with drugs targeting programmed cell death-1 being preferred. More research is required to study the long-term effects of immunotherapy on patients with autoimmune diseases.

Publication Type

Case Reports. Journal Article.

Year of Publication

2017

<161>

Unique Identifier

28760805

Title

H1N1 vaccination in Sjogren's syndrome triggers polyclonal B cell activation and promotes autoantibody production.

Source

Annals of the Rheumatic Diseases. 76(10):1755-1763, 2017 Oct.

VI 1

Status

MEDLINE

Authors

Brauner S; Folkersen L; Kvarnstrom M; Meisgen S; Petersen S; Franzen-Malmros M; Mofors J; Brokstad KA; Klareskog L; Jonsson R; Westerberg LS; Trollmo C; Malmstrom V; Ambrosi A; Kuchroo VK; Nordmark G; Wahren-Herlenius M.

Authors Full Name

Brauner, Susanna; Folkersen, Lasse; Kvarnstrom, Marika; Meisgen, Sabrina; Petersen, Sven; Franzen-Malmros, Michaela; Mofors, Johannes; Brokstad, Karl A; Klareskog, Lars; Jonsson, Roland; Westerberg, Lisa S; Trollmo, Christina; Malmstrom, Vivianne; Ambrosi, Aurelie; Kuchroo, Vijay K; Nordmark, Gunnel; Wahren-Herlenius, Marie.

Institution

Brauner, Susanna. Department of Medicine, Karolinska University Hosptial, Karolinska Institutet, Stockholm, Sweden.

Folkersen, Lasse. Department of Medicine, Karolinska University Hosptial, Karolinska Institutet, Stockholm, Sweden.

Kvarnstrom, Marika. Department of Medicine, Karolinska University Hosptial, Karolinska Institutet, Stockholm, Sweden.

Meisgen, Sabrina. Department of Medicine, Karolinska University Hosptial, Karolinska Institutet, Stockholm, Sweden.

Petersen, Sven. Department of Medicine, Karolinska University Hosptial, Karolinska Institutet, Stockholm, Sweden.

Franzen-Malmros, Michaela. Department of Medicine, Karolinska University Hosptial, Karolinska Institutet, Stockholm, Sweden.

Mofors, Johannes. Department of Medicine, Karolinska University Hosptial, Karolinska Institutet, Stockholm, Sweden.

Brokstad, Karl A. Broegelmann Research Laboratory, Department of Clinical Science, University of Bergen, Bergen, Norway.

Klareskog, Lars. Department of Medicine, Karolinska University Hosptial, Karolinska Institutet, Stockholm, Sweden.

Jonsson, Roland. Broegelmann Research Laboratory, Department of Clinical Science, University of Bergen, Bergen, Norway.

Westerberg, Lisa S. Department of Microbiology Tumor and Cell biology, Karolinska Institutet, Stockholm, Sweden.

Trollmo, Christina. Department of Medicine, Karolinska University Hosptial, Karolinska Institutet, Stockholm, Sweden.

Malmstrom, Vivianne. Department of Medicine, Karolinska University Hosptial, Karolinska Institutet, Stockholm, Sweden.

Ambrosi, Aurelie. Department of Medicine, Karolinska University Hosptial, Karolinska Institutet, Stockholm, Sweden.

Kuchroo, Vijay K. Rheumatology and Science for Life Laboratory, Department of Medical Sciences, Uppsala University, Uppsala, Sweden.

Nordmark, Gunnel. Evergrande Center for Immunologic Diseases, Harvard Medical School and Brigham and Women's Hospital, Boston, Massachusetts, USA.

Wahren-Herlenius, Marie. Department of Medicine, Karolinska University Hosptial, Karolinska Institutet, Stockholm, Sweden.
Abstract
OBJECTIVES: Vaccination of patients with rheumatic disease has been reported to result in lower antibody titres than in healthy individuals. However, studies primarily include patients on immunosuppressive therapy. Here, we investigated the immune response of treatment-naive patients diagnosed with primary Sjogren's syndrome (pSS) to an H1N1 influenza vaccine.

METHODS: Patients with Sjogren's syndrome without immunomodulatory treatment and age-matched and gender-matched healthy controls were immunised with an H1N1 influenza vaccine and monitored for serological and cellular immune responses. Clinical symptoms were monitored with a standardised form. IgG class switch and plasma cell differentiation were induced in vitro in purified naive B cells of untreated and hydroxychloroquine-treated patients and healthy controls. Gene expression was assessed by NanoString technology.

RESULTS: Surprisingly, treatment-naive patients with Sjogren's syndrome developed higher H1N1 IgG titres of greater avidity than healthy controls on vaccination. Notably, off-target B cells were also triggered resulting in increased anti-EBV and autoantibody titres. Endosomal toll-like receptor activation of naive B cells in vitro revealed a greater propensity of patient-derived cells to differentiate into plasmablasts and higher production of class switched IgG. The amplified plasma cell differentiation and class switch could be induced in cells from healthy donors by preincubation with type 1 interferon, but was abolished in hydroxychloroquine-treated patients and after in vitro exposure of naive B cells to chloroquine.

CONCLUSIONS: This comprehensive analysis of the immune response in autoimmune patients to exogenous stimulation identifies a mechanistic basis for the B cell hyperactivity in Sjogren's syndrome, and suggests that caution is warranted when considering vaccination in non-treated autoimmune patients. Copyright © Article author(s) (or their employer(s) unless otherwise stated in the text of the article) 2017. All rights reserved. No commercial use is permitted unless otherwise expressly granted.
Publication Type
Journal Article.
Year of Publication
2017

<162>

Unique Identifier

28750350

Title

Is macrophage polarization important in rheumatoid arthritis?. [Review]

Source

International Immunopharmacology. 50:345-352, 2017 Sep.

VI 1

Status

MEDLINE

Authors

Wang Y; Han CC; Cui D; Li Y; Ma Y; Wei W.

Authors Full Name

Wang, Yang; Han, Chen-Chen; Cui, Dongqian; Li, Yifan; Ma, Yang; Wei, Wei.

Institution

Wang, Yang. Institute of Clinical Pharmacology, Anhui Medical University, Key Laboratory of Anti-inflammatory and Immune Medicine, Ministry of Education, Anhui Collaborative Innovation Center of Anti-inflammatory and Immune Medicine, Hefei 230032, China.

Han, Chen-Chen. Institute of Clinical Pharmacology, Anhui Medical University, Key Laboratory of Anti-inflammatory and Immune Medicine, Ministry of Education, Anhui Collaborative Innovation Center of Anti-inflammatory and Immune Medicine, Hefei 230032, China.

Cui, Dongqian. Institute of Clinical Pharmacology, Anhui Medical University, Key Laboratory of Anti-inflammatory and Immune Medicine, Ministry of Education, Anhui Collaborative Innovation Center of Anti-inflammatory and Immune Medicine, Hefei 230032, China.

Li, Yifan. Institute of Clinical Pharmacology, Anhui Medical University, Key Laboratory of Anti-inflammatory and Immune Medicine, Ministry of Education, Anhui Collaborative Innovation Center of Anti-inflammatory and Immune Medicine, Hefei 230032, China.

Ma, Yang. Institute of Clinical Pharmacology, Anhui Medical University, Key Laboratory of Anti-inflammatory and Immune Medicine, Ministry of Education, Anhui Collaborative Innovation Center of Anti-inflammatory and Immune Medicine, Hefei 230032, China. Electronic address: mayang_ahmu@126.com.

Wei, Wei. Institute of Clinical Pharmacology, Anhui Medical University, Key Laboratory of Anti-inflammatory and Immune Medicine, Ministry of Education, Anhui Collaborative Innovation Center of Anti-inflammatory and Immune Medicine, Hefei 230032, China. Electronic address: wwei@ahmu.edu.cn.

Abstract
Macrophages are myeloid immune cells which are strategically positioned throughout the body, where they engulf and degrade debris, dead cells, and foreign substances, and coordinating the inflammatory processes. Macrophages can be divided into two extreme subsets, classical activation (M1), and alternatively activation (M2). The symptoms and signs of rheumatoid arthritis (RA) would exacerbate with the increase in pro-inflammatory cytokines, whereas anti-inflammatory cytokines will alleviate the symptoms and signs of RA. This review, mainly discusses the effects of Notch, JNK and ERK signaling pathways on the regulation of macrophage polarization, and the effects of pro-inflammatory factors and/or anti-inflammatory cytokines produced by polarized macrophages in RA. Also, we will make an attempt to find out the importance of macrophage polarization in RA treatment as a drug target. Copyright © 2017. Published by Elsevier B.V.
Publication Type
Journal Article. Review.
Year of Publication
2017

<163>
Unique Identifier
28716825
Title
Efficiency and Safety of CRAC Inhibitors in Human Rheumatoid Arthritis Xenograft Models.
Source
Journal of Immunology. 199(5):1584-1595, 2017 09 01.
VI 1
Status
MEDLINE
Authors
Liu S; Hasegawa H; Takemasa E; Suzuki Y; Oka K; Kiyoi T; Takeda H; Ogasawara T; Sawasaki T; Yasukawa M; Maeyama K.
Author NameID
Suzuki, Yasuyuki; ORCID: https://orcid.org/0000-0002-4871-9685
Takeda, Hiroyuki; ORCID: https://orcid.org/0000-0003-1677-8127

Authors Full Name
Liu, Shuang; Hasegawa, Hitoshi; Takemasa, Erika; Suzuki, Yasuyuki; Oka, Keizou; Kiyoi, Takeshi; Takeda, Hiroyuki; Ogasawara, Tomio; Sawasaki, Tatsuya; Yasukawa, Masaki; Maeyama, Kazutaka.
Institution
Liu, Shuang. Department of Pharmacology, Ehime University Graduate School of Medicine, Shitsugawa, Toon-shi, Ehime 791-0295, Japan; liussmzk@m.ehime-u.ac.jp.
Hasegawa, Hitoshi. Department of Hematology, Clinical Immunology and Infectious Diseases, Ehime University Graduate School of Medicine, Ehime 791-0295, Japan.
Takemasa, Erika. Department of Pharmacology, Ehime University Graduate School of Medicine, Shitsugawa, Toon-shi, Ehime 791-0295, Japan.
Suzuki, Yasuyuki. Department of Pharmacology, Ehime University Graduate School of Medicine, Shitsugawa, Toon-shi, Ehime 791-0295, Japan.
Oka, Keizou. Department of Bioscience, Integrated Center for Sciences, Ehime University, Shitsugawa, Toon-shi, Ehime 791-0295, Japan; and.
Kiyoi, Takeshi. Department of Bioscience, Integrated Center for Sciences, Ehime University, Shitsugawa, Toon-shi, Ehime 791-0295, Japan; and.
Takeda, Hiroyuki. Proteo-Science Center, Ehime University, Matsuyama, Ehime 791-8577, Japan.
Ogasawara, Tomio. Proteo-Science Center, Ehime University, Matsuyama, Ehime 791-8577, Japan.
Sawasaki, Tatsuya. Proteo-Science Center, Ehime University, Matsuyama, Ehime 791-8577, Japan.
Yasukawa, Masaki. Department of Hematology, Clinical Immunology and Infectious Diseases, Ehime University Graduate School of Medicine, Ehime 791-0295, Japan.
Maeyama, Kazutaka. Department of Pharmacology, Ehime University Graduate School of Medicine, Shitsugawa, Toon-shi, Ehime 791-0295, Japan.
Abstract
Store-operated Ca2+ release-activated Ca2+ (CRAC) channels are involved in the pathogenesis of rheumatoid arthritis (RA) and have been studied as therapeutic targets in the management of RA. We investigated the efficacy and safety of CRAC inhibitors, including a neutralizing Ab (hCRACM1-IgG) and YM-58483, in the treatment of RA. Patient-derived T cell and B cell activity was suppressed by hCRACM1-IgG as well as YM-58483. Systemically constant, s.c. infused CRAC inhibitors showed anti-inflammatory activity in a human-NOD/SCID xenograft RA model as well as protective effects against the destruction of cartilage and bone. hCRACM1-IgG appeared to be safe for systemic application, whereas YM-58483 showed hepatic and renal toxicity in xenograft mice. Treatment with both CRAC inhibitors also caused hyperglycemia in xenograft

mice. These results indicate the potential of hCRACM1-IgG and YM-58483 as anti-immunological agents for the treatment of RA. However, some safety issues should be addressed and application methods should be optimized prior to their clinical use. Copyright © 2017 by The American Association of Immunologists, Inc.

Publication Type

Journal Article. Research Support, Non-U.S. Gov't.

Year of Publication

2017

<164>

Unique Identifier

28701761

Title

Antigen-specific immunotherapies in rheumatic diseases. [Review]

Source

Nature Reviews Rheumatology. 13(9):525-537, 2017 Sep.

VI 1

Status

MEDLINE

Authors

Pozsgay J; Szekanecz Z; Sarmay G.

Authors Full Name

Pozsgay, Judit; Szekanecz, Zoltan; Sarmay, Gabriella.

Institution

Pozsgay, Judit. Department of Immunology, Eotvos Lorand University (ELTE), Pazmany Peter Setany 1/C, Budapest 1117, Hungary.

Szekanecz, Zoltan. Division of Rheumatology, Department of Medicine, University of Debrecen Faculty of Medicine, Nagyerdei Korut 98, Debrecen 4032, Hungary.

Sarmay, Gabriella. Department of Immunology, Eotvos Lorand University (ELTE), Pazmany Peter Setany 1/C, Budapest 1117, Hungary.

Abstract

The main goal of antigen-specific immunotherapy (ASI) in autoimmune and rheumatic diseases is to reprogramme or remove autoreactive cells and/or induce immune tolerance to self-antigens.

Current therapies in these diseases either treat symptoms or slow down disease progression but are not yet curative or preventative - disease-specific treatments are urgently needed. In contrast to the nonspecific treatments in current use that induce generalized immune suppression, which is associated with several adverse effects including increased risk of infections, ASIs target a restricted subset of B cells or T cells, and thus do not compromise systemic immunity and host defence. This Review provides a summary of novel approaches for identifying autoepitopes and detecting and targeting autoreactive cells that might help in the development of ASIs. Promising approaches include the use of tolerizing peptides coupled to MHC constructs and/or nanocompounds, tolerizing dendritic cells and antigen-specific vaccines. Following studies in animal models of rheumatoid arthritis and systemic lupus erythematosus, several of these strategies have now entered clinical trials. However, to use these approaches in humans, several important limitations must first be addressed, such as; selecting the proper immunodominant autoantigen; identifying the optimal timing, dosing and route of administration; finding biomarkers for monitoring the therapy; and optimizing methodology.

Publication Type

Journal Article. Review.

Year of Publication

2017

<165>

Unique Identifier

28689441

Title

Update of sarilumb to treat rheumatoid arthritis based on randomized clinical trials: a systematic review. [Review]

Source

Expert Review of Clinical Immunology. 13(8):741-752, 2017 08.

VI 1

Status

MEDLINE

Authors

Aly AM; Furst DE.

Authors Full Name

Aly, Aly M; Furst, Daniel E.
Institution
Aly, Aly M. a Alexandria University Faculty of Medicine, Alexandria, Egypt.
Furst, Daniel E. b Department of Medicine, Division of Rheumatology, University of California Los Angeles, Los Angeles, CA, USA.
Abstract
INTRODUCTION: Sarilumab is a human monoclonal antibody against Interleukin 6 alpha (IL-6alpha) receptor. Compared to tocilizumab, another IL-6 alpha receptor antibody, sarilumab has a different structure and higher affinity. Areas covered: In a systematic literature review, we examined all sarilumab randomized clinical trials (RCTs) in rheumatoid arthritis. The 6 reviewed RCTs included patients who were inadequate MTX, DMARD and/or TNFi responders. Sarilumab 150-200 mg every 2 weeks improved RA signs, symptoms, function and decreased radiological progression up to 52 weeks. The most common adverse events were infections and neutropenia, the latter of which will require careful observation in future trials. Examination of the effect of sero-positivity, disease duration, presence of erosions, use of previous biologic and comparisons to other biologics etc are still needed to complete understanding of this drug's profile. Long term studies, too, will be needed to assess long term tolerability Expert commentary: Results support the use of sarilumab to treat RA patients with inadequate response to MTX, other DMARDs and TNFis, although further studies are needed to fully assess its toxicity and understand the specific place of sarilumab in the RA armamentarium.
Publication Type
Journal Article. Review. Systematic Review.
Year of Publication
2017

<166>
Unique Identifier
28646772
Title
Prevalence of immune-related systemic adverse events in patients treated with anti-Programmed cell Death 1/anti-Programmed cell Death-Ligand 1 agents: A single-centre pharmacovigilance database analysis. European Journal of Cancer. 82:34-44, 2017 09.

Authors
Le Burel S; Champiat S; Mateus C; Marabelle A; Michot JM; Robert C; Belkhir R; Soria JC; Laghouati S; Voisin AL; Fain O; Mekinian A; Coutte L; Szwebel TA; Dunogeant L; Lioger B; Luxembourger C; Mariette X; Lambotte O.
Authors Full Name
Le Burel, Sebastien; Champiat, Stephane; Mateus, Christine; Marabelle, Aurelien; Michot, Jean-Marie; Robert, Caroline; Belkhir, Rakiba; Soria, Jean-Charles; Laghouati, Salim; Voisin, Anne-Laure; Fain, Olivier; Mekinian, Arsene; Coutte, Laetitia; Szwebel, Tali-Anne; Dunogeant, Laetitia; Lioger, Bertrand; Luxembourger, Cecile; Mariette, Xavier; Lambotte, Olivier.
Institution
Le Burel, Sebastien. Assistance Publique - Hopitaux de Paris, Hopital Bicetre, Service de Medecine Interne et Immunologie Clinique, F-94275 Le Kremlin-Bicetre, France.
Champiat, Stephane. Departement D'Innovation Therapeutique et des Essais Precoces (DITEP), Gustave Roussy, Universite Paris Saclay, F-94805 Villejuif, France; INSERM U981, F-94805 Villejuif, France.
Mateus, Christine. Departement de Dermatologie, Institut Gustave Roussy, F-94805 Villejuif, France.
Marabelle, Aurelien. Departement D'Innovation Therapeutique et des Essais Precoces (DITEP), Gustave Roussy, Universite Paris Saclay, F-94805 Villejuif, France; INSERM U981, F-94805 Villejuif, France.
Michot, Jean-Marie. Departement D'Innovation Therapeutique et des Essais Precoces (DITEP), Gustave Roussy, Universite Paris Saclay, F-94805 Villejuif, France.
Robert, Caroline. Departement de Dermatologie, Institut Gustave Roussy, F-94805 Villejuif, France.
Belkhir, Rakiba. Assistance Publique - Hopitaux de Paris, Hopital Bicetre, Service de Rhumatologie, F-94275 Le Kremlin-Bicetre, France.
Soria, Jean-Charles. Departement D'Innovation Therapeutique et des Essais Precoces (DITEP), Gustave Roussy, Universite Paris Saclay, F-94805 Villejuif, France; INSERM U981, F-94805 Villejuif, France.
Laghouati, Salim. Unite Fonctionnelle de Pharmacovigilance, Institut Gustave Roussy, F-94805 Villejuif, France.
Voisin, Anne-Laure. Unite Fonctionnelle de Pharmacovigilance, Institut Gustave Roussy, F-94805 Villejuif, France.
Fain, Olivier. Assistance Publique - Hopitaux de Paris, Hopital Saint-Antoine, Service de Medecine Interne, DHU I2B, Universite Paris 6, F-75012 Paris, France.
Mekinian, Arsene. Assistance Publique - Hopitaux de Paris, Hopital Saint-Antoine, Service de Medecine Interne, DHU I2B, Universite Paris 6, F-75012 Paris, France.

Coutte, Laetitia. Assistance Publique - Hopitaux de Paris, Hopital Cochin, Service de Medecine Interne, F-75014 Paris, France.

Szwebel, Tali-Anne. Assistance Publique - Hopitaux de Paris, Hopital Cochin, Service de Medecine Interne, F-75014 Paris, France.

Dunogeant, Laetitia. Center Hospitalier Du Pays D'Aix, Service de Rhumatologie et Medecine Interne, F-13616 Aix en Provence, France.

Lioger, Bertrand. CHRU de Tours, Hopital Bretonneau, Service de Medecine Interne, F-37044 Tours, France.

Luxembourger, Cecile. CHU de Toulouse, Hopital Pierre-Paul Ricquet, Center de Rhumatologie, F-31059 Toulouse, France.

Mariette, Xavier. Assistance Publique - Hopitaux de Paris, Hopital Bicetre, Service de Rhumatologie, F-94275 Le Kremlin-Bicetre, France; INSERM, U1184, Immunology of Viral Infections and Autoimmune Diseases, F-94276 Le Kremlin-Bicetre, France; Universite Paris Sud, UMR 1184, F-94276 Le Kremlin-Bicetre, France.

Lambotte, Olivier. Assistance Publique - Hopitaux de Paris, Hopital Bicetre, Service de Medecine Interne et Immunologie Clinique, F-94275 Le Kremlin-Bicetre, France; INSERM, U1184, Immunology of Viral Infections and Autoimmune Diseases, F-94276 Le Kremlin-Bicetre, France; Universite Paris Sud, UMR 1184, F-94276 Le Kremlin-Bicetre, France; CEA, DSV/iMETI, IDMIT, F-92265 Fontenay-aux-Roses, France. Electronic address: olivier.lambotte@aphp.fr.

Abstract

AIM: The growing use of immune checkpoint inhibitors (ICIs) is associated with the occurrence of immune-related adverse events (irAEs). Few data are published on systemic, immunohaematological and rheumatic irAEs. In a pharmacovigilance database analysis, we screened for these irAEs and calculated their prevalence.

PATIENTS AND METHODS: Participants were recruited via Registre des Effets Indesirables Severes des Anticorps Monoclonaux Immunomodulateurs en Cancerologie (REISAMIC)1 a French registry of grade >=2 irAEs occurring in ICI-treated patients. The pathologies of interest were systemic autoimmune and inflammatory diseases, rheumatic diseases and immune cytopenia.

RESULTS: Out of 908 patients treated with anti-Programmed cell Death 1 (PD1)/anti-Programmed cell Death-Ligand 1 (PD-L1) agents (together with an anti-cytotoxic T-lymphocyte antigen-4 (CTLA-4) agent in 40 cases) between December 2012 and December 2016 at a single centre, 21 patients experienced systemic irAEs. The types and the prevalence of irAEs were as follows: immune thrombocytopenia (0.2%), Sjogren syndrome (0.3%), rheumatoid arthritis (0.2%), polymyalgia rheumatica (0.2%), psoriatic arthritis (0.2%), seronegative polyarthritis (0.7%) and

sarcoidosis (0.2%). Patients with Sjogren syndrome or seronegative polyarthritis were more likely to have received combination therapy with ipilimumab (2.5% for both). We described these 21 cases, together with nine additional cases from five other centres. Most irAE were moderately severe (grade 2, 63%). The median time to onset was 57degreedays (interquartile range (IQR) 24-117). The ICI was withdrawn in 12 cases, 25 patients (83%) received corticosteroids, and five patients (17%) received immunosuppressant/immunomodulatory agents. The irAEs resolved fully or partially in 28 cases (93%).

CONCLUSION: Although systemic, immunohaematological and rheumatic diseases are rarely associated with ICI use, the prevalence is higher when two ICIs are combined. Corticosteroids are often effective and may enable the continued administration of ICIs. Studies designed to identify at-risk patients are warranted. Copyright © 2017 Elsevier Ltd. All rights reserved.

Publication Type

Journal Article.

Year of Publication

2017

<167>

Unique Identifier

28631705

Title

[Vaccination in rheumatology: Evolution of views on the problem]. [Review] [Russian]

Source

Terapevticheskii Arkhiv. 89(5):83-89, 2017.

VI 1

Status

MEDLINE

Authors

Belov BS; Sergeeva MS; Tarasova GM; Bukhanova DV.

Authors Full Name

Belov, B S; Sergeeva, M S; Tarasova, G M; Bukhanova, D V.

Institution

Belov, B S. V.A. Nasonova Research Institute of Rheumatology, Moscow, Russia.

Sergeeva, M S. V.A. Nasonova Research Institute of Rheumatology, Moscow, Russia.
Tarasova, G M. V.A. Nasonova Research Institute of Rheumatology, Moscow, Russia.
Bukhanova, D V. V.A. Nasonova Research Institute of Rheumatology, Moscow, Russia.
Abstract
The problem of coinfections that are due to both a rheumatic disease (RD) itself and the need to use immunosuppressive drugs deserves apparent attention in modern rheumatology. Coinfections substantially affect morbidity and mortality rates, especially in diffuse connective tissue diseases. The data available in the literature on the above subject matter suggest that vaccination is a powerful method for prevention of infectious diseases that are the most important problem for patients with RD.
Other Abstract
Publisher: HecoMHeHHo o BH MaH B coBpeMeHHo peBMaTo o 3ac y BaeT po eMa co eTaHHblx H ek , opM poBaH e koTopblx o yc oB eHo kak caM M peBMaT eck M 3a o eBaH eM (P), Tak Heo xo MocTb p MeHeH pe apaToB MMyHocy pecc BHo o e cTB . So eTaHHble H ek cy ecTBeHHo B T Ha 3a o eBaeMocTb eTa bHocTb, oco eHHo p y3Hblx opa eH x coe H Te bHo TkaH . aHHble TepaTypbl o yka3aHHo po eMaT ke cB eTe bcTBy T, To Bak Ha pe cTaB eT co o Mo He MeTo pe y pe eH H ek oHHblx 3a o eBaH , koTopble B Tc kpa He Ba Ho po eMo a eHToB c P .; Language: Russian
Publication Type
Journal Article. Review.
Year of Publication
2017

<168>
Unique Identifier
28612747
Title
Pathogenetic insights from the treatment of rheumatoid arthritis. [Review]
Source
Lancet. 389(10086):2328-2337, 2017 06 10.
VI 1
Status
MEDLINE

Authors
McInnes IB; Schett G.
Authors Full Name
McInnes, Iain B; Schett, Georg.
Institution
McInnes, Iain B. Institute of Infection Immunity and Inflammation, University of Glasgow, Glasgow, UK. Electronic address: iain.mcinnes@glasgow.ac.uk.
Schett, Georg. Department of Internal Medicine 3-Rheumatology and Immunology, Friedrich-Alexander-University Erlangen-Nurnberg, Erlangen, Germany; Universitatsklinikum Erlangen, Erlangen, Germany.
Comments
Comment in (CIN)
Abstract
Rheumatoid arthritis is a chronic autoimmune disease that causes progressive articular damage, functional loss, and comorbidity. The development of effective biologics and small-molecule kinase inhibitors in the past two decades has substantially improved clinical outcomes. Just as understanding of pathogenesis has led in large part to the development of drugs, so have mode-of-action studies of these specific immune-targeted agents revealed which immune pathways drive articular inflammation and related comorbidities. Cytokine inhibitors have definitively proven a critical role for tumour necrosis factor alpha and interleukin 6 in disease pathogenesis and possibly also for granulocyte-macrophage colony-stimulating factor. More recently, clinical trials with Janus kinase (JAK) inhibitors have shown that cytokine receptors that signal through the JAK/STAT signalling pathway are important for disease, informing the pathogenetic function of additional cytokines (such as the interferons). Finally, successful use of costimulatory blockade and B-cell depletion in the clinic has revealed that the adaptive immune response and the downstream events initiated by these cells participate directly in synovial inflammation. Taken together, it becomes apparent that understanding the effects of specific immune interventions can elucidate definitive molecular or cellular nodes that are essential to maintain complex inflammatory networks that subserve diseases like rheumatoid arthritis. Copyright © 2017 Elsevier Ltd. All rights reserved.
Publication Type
Journal Article. Review.
Year of Publication
2017

<169>

Unique Identifier

28583168

Title

Mesenchymal stem cells for treating autoimmune dacryoadenitis. [Review]

Source

Stem Cell Research & Therapy. 8(1):126, 2017 06 05.

VI 1

Status

MEDLINE

Authors

Lu X; Wang X; Nian H; Yang D; Wei R.

Authors Full Name

Lu, Xiaoxiao; Wang, Xilian; Nian, Hong; Yang, Dan; Wei, Ruihua.

Institution

Lu, Xiaoxiao. Tianjin Medical University Eye Hospital, Tianjin Medical University Eye Institute & Tianjin Medical University School of Optometry and Ophthalmology, No.251 Fukang Road, Nankai District, Tianjin, 300384, People's Republic of China.

Wang, Xilian. Tianjin Beichen Hospital, No. 7, Beiyi Road, Beichen District, Tianjin, 300400, China.

Nian, Hong. Tianjin Medical University Eye Hospital, Tianjin Medical University Eye Institute & Tianjin Medical University School of Optometry and Ophthalmology, No.251 Fukang Road, Nankai District, Tianjin, 300384, People's Republic of China.

Yang, Dan. Tianjin Medical University Eye Hospital, Tianjin Medical University Eye Institute & Tianjin Medical University School of Optometry and Ophthalmology, No.251 Fukang Road, Nankai District, Tianjin, 300384, People's Republic of China.

Wei, Ruihua. Tianjin Medical University Eye Hospital, Tianjin Medical University Eye Institute & Tianjin Medical University School of Optometry and Ophthalmology, No.251 Fukang Road, Nankai District, Tianjin, 300384, People's Republic of China. braveheart0717@sina.com.

Abstract

Autoimmune dacryoadenitis, such as Sjogren syndrome, comprises multifactorial and complex diseases. Inflammation of the lacrimal gland plays a key role in the pathogenesis of diseases. Unfortunately, current treatment strategies, including artificial tears, anti-inflammatory drugs, punctual occlusion, and immunosuppressive drugs, are only palliative, and long-term administration of these strategies is associated with adverse effects that limit their utility. Hence,

an effective and safe treatment for autoimmune dacryoadenitis is urgently needed. Mesenchymal stem cells (MSCs) have emerged as a promising tool for treating autoimmune dacryoadenitis, owing to their immunosuppressive properties, tissue repair functions, and powerful differentiation capabilities. A large number of studies have focused on the effect of MSCs on autoimmune diseases, such as autoimmune uveitis, inflammatory bowel disease, and collagen-induced arthritis, but few studies have, to date, unequivocally established the efficacy of MSCs for treating autoimmune dacryoadenitis. In this review, we discuss recent advances in MSC treatment for autoimmune dacryoadenitis.

Publication Type

Journal Article. Review.

Year of Publication

2017

<170>

Unique Identifier

28545880

Title

Stepwise preconditioning enhances mesenchymal stem cell-based cartilage regeneration through epigenetic modification.

Source

Osteoarthritis & Cartilage. 25(9):1541-1550, 2017 09.

VI 1

Status

MEDLINE

Authors

Lin S; Lee WYW; Xu L; Wang Y; Chen Y; Ho KKW; Qin L; Jiang X; Cui L; Li G.

Authors Full Name

Lin, S; Lee, W Y W; Xu, L; Wang, Y; Chen, Y; Ho, K K W; Qin, L; Jiang, X; Cui, L; Li, G.

Institution

Lin, S. Department of Orthopaedics & Traumatology, Lui Che Woo Institute of Innovative Medicine, Li Ka Shing Institute of Health Sciences, Faculty of Medicine, The Chinese University of Hong Kong, Prince of Wales Hospital, Hong Kong, China; Department of Pharmacology, Guangdong Key Laboratory for Research and Development of Natural Drugs, Guangdong

Medical University, Zhanjiang, Guangdong, China; The CUHK-ACC Space Medicine Centre on Health Maintenance of Musculoskeletal System, The Chinese University of Hong Kong Shenzhen Research Institute, Shenzhen, China.

Lee, W Y W. Department of Orthopaedics & Traumatology, Lui Che Woo Institute of Innovative Medicine, Li Ka Shing Institute of Health Sciences, Faculty of Medicine, The Chinese University of Hong Kong, Prince of Wales Hospital, Hong Kong, China; The CUHK-ACC Space Medicine Centre on Health Maintenance of Musculoskeletal System, The Chinese University of Hong Kong Shenzhen Research Institute, Shenzhen, China.

Xu, L. Department of Orthopaedics & Traumatology, Lui Che Woo Institute of Innovative Medicine, Li Ka Shing Institute of Health Sciences, Faculty of Medicine, The Chinese University of Hong Kong, Prince of Wales Hospital, Hong Kong, China; The CUHK-ACC Space Medicine Centre on Health Maintenance of Musculoskeletal System, The Chinese University of Hong Kong Shenzhen Research Institute, Shenzhen, China.

Wang, Y. Department of Orthopaedics & Traumatology, Lui Che Woo Institute of Innovative Medicine, Li Ka Shing Institute of Health Sciences, Faculty of Medicine, The Chinese University of Hong Kong, Prince of Wales Hospital, Hong Kong, China.

Chen, Y. Department of Orthopaedics & Traumatology, Lui Che Woo Institute of Innovative Medicine, Li Ka Shing Institute of Health Sciences, Faculty of Medicine, The Chinese University of Hong Kong, Prince of Wales Hospital, Hong Kong, China.

Ho, K K W. Department of Orthopaedics & Traumatology, Lui Che Woo Institute of Innovative Medicine, Li Ka Shing Institute of Health Sciences, Faculty of Medicine, The Chinese University of Hong Kong, Prince of Wales Hospital, Hong Kong, China.

Qin, L. Department of Orthopaedics & Traumatology, Lui Che Woo Institute of Innovative Medicine, Li Ka Shing Institute of Health Sciences, Faculty of Medicine, The Chinese University of Hong Kong, Prince of Wales Hospital, Hong Kong, China; The CUHK-ACC Space Medicine Centre on Health Maintenance of Musculoskeletal System, The Chinese University of Hong Kong Shenzhen Research Institute, Shenzhen, China.

Jiang, X. Key Laboratory for Regenerative Medicine, Ministry of Education, School of Biomedical Sciences, Faculty of Medicine, The Chinese University of Hong Kong, Hong Kong, China.

Cui, L. Department of Pharmacology, Guangdong Key Laboratory for Research and Development of Natural Drugs, Guangdong Medical University, Zhanjiang, Guangdong, China.

Li, G. Department of Orthopaedics & Traumatology, Lui Che Woo Institute of Innovative Medicine, Li Ka Shing Institute of Health Sciences, Faculty of Medicine, The Chinese University of Hong Kong, Prince of Wales Hospital, Hong Kong, China; The CUHK-ACC Space Medicine Centre on Health Maintenance of Musculoskeletal System, The Chinese University of Hong Kong Shenzhen Research Institute, Shenzhen, China; Key Laboratory for Regenerative Medicine,

Ministry of Education, School of Biomedical Sciences, Faculty of Medicine, The Chinese University of Hong Kong, Hong Kong, China. Electronic address: gangli@cuhk.edu.hk.
Abstract

OBJECTIVE: This study is to investigate the functions and underlying mechanisms of mesenchymal stem cells (MSCs) underwent stepwise preconditioning in chondrogenic medium before expansion, then further explore their therapeutic effects in a surgically induced osteoarthritis (OA) model.

METHODS: MSCs isolated from the adult rats expressing Green Fluorescence Protein (GFP) were incubated in basal medium or primed in chondrogenic medium before expansion. The multipotency including cell proliferation, differentiation, and survivability was compared between chondrogenic manipulated MSCs (M-MSCs) and untreated MSCs. Methylation modification of Nanog and Oct4 were detected by bisulfite genomic sequencing. Loss-of-function phenotype in M-MSCs induced by shNanog was also observed. Then the therapeutic effect of the cells was evaluated in a surgically induced OA rat model by single intraarticular injection. The injected GFP-labeled cells in the joints were monitored in vivo. These rats were sacrificed and subjected to histological examinations and microstructural analysis after 4 weeks.

RESULTS: We found that cell clonogenicity, proliferation, survivability, and chondrogenic property were enhanced after stepwise preconditioning. We then further found that the expression level of Nanog and Oct4 was temporarily increased in the M-MSCs. Results of epigenetic analysis revealed that demethylation happened in Nanog and Oct4 after the stepwise preconditioning. Results of in vivo imaging showed more GFP-labeled cells in the M-MSCs-injected group. And results of histology and micro-CT analysis also indicated a superior therapeutic effect of M-MSCs on the surgically induced-OA.

CONCLUSION: These findings indicated a feasible method to obtain a cell population with high survivability and chondrogenic commitment for the treatment of OA. Copyright © 2017. Published by Elsevier Ltd.
Publication Type

Journal Article.

Year of Publication

2017

<171>

Unique Identifier

28540751

Title

Treatment of adult-onset still's disease: up to date. [Review]

Source

Expert Review of Clinical Immunology. 13(9):849-866, 2017 09.

VI 1

Status

MEDLINE

Authors

Yoo DH.

Author NameID

Yoo, Dae Hyun; ORCID: https://orcid.org/0000-0002-0643-4008

Authors Full Name

Yoo, Dae Hyun.

Institution

Yoo, Dae Hyun. a Department of Rheumatology, College of Medicine, Hanyang University Hospital for Rheumatic Diseases, Seoul, Korea.

Abstract

INTRODUCTION: Adult onset Still's disease (AOSD) is a systemic inflammatory disorder of unknown etiology, and approximately 60-70% of patients may develop a chronic polyphasic form of the disease or a chronic polyarthritis. Due to rarity of disease, treatment of AOSD is not based on controlled study, but on case based experiences. Areas covered: Recently, the application of anti-cytokine therapy based on pathophysiology has resulted in significant progress in the treatment of AOSD. Here, we review current knowledge of the pathogenesis, disease progression, currently available biomarkers of disease activity, standard therapeutic agents, utility of biologic agents, future perspectives for treatment and treatment of macrophage activation syndrome. Expert commentary: Accumulated clinical data suggest that chronic disease can be classified into two subsets: dominant systemic disease, and the arthritis subgroup. IL-1 inhibitors may be more efficient for systemic manifestations and IL-6 inhibitor for both joint involvement and systemic manifestations. TNF inhibitors must be reserved for patients with purely chronic articular manifestations. For ideal management of patients, it is very important to measure disease activity accurately during follow up, but no single biomarker has been classified as ideal. New therapeutic agents and composite biomarkers are needed to improve the outcome of patients with AOSD by identifying disease activity properly.

Publication Type

Journal Article. Review.

Year of Publication

2017

<172>

Unique Identifier

28516435

Title

Management Considerations in Cancer Patients With Rheumatoid Arthritis. [Review]

Source

Oncology (Williston Park). 31(5):374-80, 2017 05 15.

VI 1

Status

MEDLINE

Authors

Zogala RJ; Goutsouliak K; Suarez-Almazor ME.

Authors Full Name

Zogala, Richard J; Goutsouliak, Kristina; Suarez-Almazor, Maria E.

Abstract

Rheumatoid arthritis is a common inflammatory disease that requires treatment with immunosuppressants to control symptoms and avoid joint destruction. Managing cancer in patients with concomitant rheumatoid arthritis poses special challenges that require close coordination of care between oncologists and rheumatologists. Potential clinical issues needing special consideration include: 1) perioperative management in patients undergoing cancer surgery, which often requires discontinuation of antirrheumatic therapy; 2) use of immunosuppressant therapies for rheumatoid arthritis, especially biologic agents that inhibit cytokine and immune pathways, which conceivably could affect immune-mediated antitumor responses (the issues are different in patients with active cancer vs those with a past history of cancer and no recurrences); 3) management in the palliative care setting; and 4) use of cancer immunotherapy, such as checkpoint inhibitor agents, in patients with pre-existing rheumatoid arthritis. We explore these clinical issues in case-based scenarios. In all cases, clinical decision making must include a careful weighing of risks and benefits of both cancer treatments and

antirrheumatic therapies, with attention given to prognosis and life expectancy, quality of life, and patient preferences.

Publication Type

Journal Article. Review.

Year of Publication

2017

<173>

Unique Identifier

28516380

Title

Polymyalgia rheumatica in a melanoma patient due to nivolumab treatment.

Journal of Cancer Research & Clinical Oncology. 143(7):1357-1358, 2017 07.

Authors

Nakamagoe K; Moriyama T; Maruyama H; Yokosawa M; Hara T; Tanaka S; Fujimoto M; Tamaoka A.

Author NameID

Nakamagoe, Kiyotaka; ORCID: https://orcid.org/0000-0003-4025-5780

Authors Full Name

Nakamagoe, Kiyotaka; Moriyama, Tetuya; Maruyama, Hiroshi; Yokosawa, Masahiro; Hara, Tadashi; Tanaka, Shinya; Fujimoto, Manabu; Tamaoka, Akira.

Institution

Nakamagoe, Kiyotaka. Division of Clinical Medicine, Department of Neurology, Faculty of Medicine, University of Tsukuba, Tsukuba, Ibaraki, 305-8575, Japan. Nakamagoek@md.tsukuba.ac.jp.

Moriyama, Tetuya. Division of Clinical Medicine, Department of Neurology, Faculty of Medicine, University of Tsukuba, Tsukuba, Ibaraki, 305-8575, Japan.

Maruyama, Hiroshi. Division of Clinical Medicine, Department of Dermatology, Faculty of Medicine, University of Tsukuba, Tsukuba, Ibaraki, Japan.

Yokosawa, Masahiro. Division of Clinical Medicine, Department of Rheumatology, Faculty of Medicine, University of Tsukuba, Tsukuba, Ibaraki, Japan.

Hara, Tadashi. Division of Clinical Medicine, Department of Radiology, Faculty of Medicine, University of Tsukuba, Tsukuba, Ibaraki, Japan.
Tanaka, Shinya. Division of Clinical Medicine, Department of Neurology, Faculty of Medicine, University of Tsukuba, Tsukuba, Ibaraki, 305-8575, Japan.
Fujimoto, Manabu. Division of Clinical Medicine, Department of Dermatology, Faculty of Medicine, University of Tsukuba, Tsukuba, Ibaraki, Japan.
Tamaoka, Akira. Division of Clinical Medicine, Department of Neurology, Faculty of Medicine, University of Tsukuba, Tsukuba, Ibaraki, 305-8575, Japan.
Publication Type
Case Reports. Letter.
Year of Publication
2017

<174>
Unique Identifier
28494214
Title
The role and therapeutic targeting of IL-6 in rheumatoid arthritis. [Review]
Source
Expert Review of Clinical Immunology. 13(6):535-551, 2017 06.
VI 1
Status
MEDLINE
Authors
Narazaki M; Tanaka T; Kishimoto T.
Authors Full Name
Narazaki, Masashi; Tanaka, Toshio; Kishimoto, Tadamitsu.
Institution
Narazaki, Masashi. a Department of Respiratory Medicine, Allergy and Rheumatic Diseases, Osaka University Graduate School of Medicine, Osaka University, Osaka, Japan.
Narazaki, Masashi. b Department of Immunopathology, World Premier International Immunology Frontier Research Center, Osaka University, Osaka, Japan.

Tanaka, Toshio. b Department of Immunopathology, World Premier International Immunology Frontier Research Center, Osaka University, Osaka, Japan.
Tanaka, Toshio. c Department of Clinical Application of Biologics, Osaka University Graduate School of Medicine, Osaka University, Osaka, Japan.
Kishimoto, Tadamitsu. d Laboratory of Immune Regulation, World Premier International Immunology Frontier Research Center, Osaka University, Osaka, Japan.
Abstract
INTRODUCTION: Rheumatoid arthritis (RA) is an autoimmune chronic disease with joint and systemic inflammation and it has been found that interleukin-6 (IL-6) plays a key role in RA. Indeed, various clinical studies have proved that the first-in-class IL-6 inhibitor, tocilizumab, a humanized anti-IL-6 receptor monoclonal antibody, showed outstanding efficacy in RA. Areas covered: We review here the role of IL-6 in the inflammatory conditions and how IL-6 contributes to pathogenesis of RA, what induces IL-6 and how IL-6 expression is regulated. Furthermore, clinical studies of tocilizumab for RA are summarized, Expert commentary: We review and discuss the prospects for future applications of IL-6 targeting therapy and new therapeutic strategies targeting IL-6. Finally, we discuss relevant issues with regard to the clinical management of IL-6 blockade in RA.
Publication Type
Journal Article. Review.
Year of Publication
2017

<175>
Unique Identifier
28489423
Title
Is rheumatoid arthritis associated with reduced immunogenicity of the influenza vaccination? A systematic review and meta-analysis. [Review]
Source
Current Medical Research & Opinion. 33(10):1901-1908, 2017 10.
VI 1
Status
MEDLINE

Authors

Huang Y; Wang H; Tam WWS.

Authors Full Name

Huang, Yafang; Wang, Huili; Tam, Wilson W S.

Institution

Huang, Yafang. a School of General Practice and Continuing Education, Capital Medical University, Beijing, China.

Wang, Huili. a School of General Practice and Continuing Education, Capital Medical University, Beijing, China.

Tam, Wilson W S. b Alice Lee Centre for Nursing Studies, Yong Loo Lin School of Medicine, National University of Singapore, Singapore.

Abstract

OBJECTIVE: To determine whether immunogenicity and safety of the influenza vaccination in rheumatoid arthritis (RA) patients are significantly different from those in a healthy population.

METHODS: PubMed, MEDLINE, Embase, Cochrane Library and Web of Science were searched on 31 August 2016. Studies were included when they met the inclusion criteria. Two reviewers independently extracted data on study characteristics, methodological quality and outcomes. The primary outcome was seroprotection (SP) rate after immunization.

RESULTS: Thirteen studies were included. The SP rates did not significantly differ between the RA patients and healthy controls for the H3N2 (RR = 0.96, 95% CI, 0.82 to 1.13, p = .64) and B strain (RR = 0.95, 95% CI 0.84 to 1. 08, p = .44). Nevertheless, RA was associated with a significant decrease in SP rate for the H1N1 strain (RR = 0.72, 95% CI 0.60 to 0.86, p < .001). RA patients receiving immunosuppressive chemotherapy, TNF blockers, rituximab and other biologics responded to the H1N1 strain significantly less than healthy controls in SP rate, whereas those receiving steroids did not. Non-adjuvanted vaccination had a significantly lower SP rate than in healthy controls, whereas adjuvanted vaccination did not. RA was associated with an increase in adverse events (RR = 1.77, 95% CI 1.02 to 3.08, p = .04).

CONCLUSIONS: Immunogenicity was significantly different between RA patients and healthy controls for the H1N1 strain, but not for the H3N2 or B strains. Adverse event rates were higher in RA patients. Adjuvant and special kinds of immunosuppressive biologics may play an important role in immunogenicity of inactivated influenza vaccines for RA patients.

Publication Type

Journal Article. Meta-Analysis. Review. Systematic Review.

Year of Publication

2017

<176>

Unique Identifier

28483543

Title

Vaccinations and risk of systemic lupus erythematosus and rheumatoid arthritis: A systematic review and meta-analysis. [Review]

Source

Autoimmunity Reviews. 16(7):756-765, 2017 Jul.

VI 1

Status

MEDLINE

Authors

Wang B; Shao X; Wang D; Xu D; Zhang JA.

Authors Full Name

Wang, Bin; Shao, Xiaoqing; Wang, Dan; Xu, Donghua; Zhang, Jin-An.

Institution

Wang, Bin. Department of Rheumatology and Immunology, Jinshan Hospital of Fudan University, Shanghai 201508, China; Department of Endocrinology, Jinshan Hospital of Fudan University, Shanghai 201508, China.

Shao, Xiaoqing. Department of Rheumatology and Immunology, Jinshan Hospital of Fudan University, Shanghai 201508, China; Department of Endocrinology, Jinshan Hospital of Fudan University, Shanghai 201508, China.

Wang, Dan. Department of Epidemiology and Biostatistics, School of Public Health, Hebei Medical University, Shijiazhuang 050017, China; Department of Library, Hebei Medical University, Shijiazhuang 050017, China.

Xu, Donghua. Department of Rheumatology and Immunology, The Affiliated Hospital of Weifang Medical University, Weifang 261000, China.

Zhang, Jin-An. Department of Rheumatology and Immunology, Jinshan Hospital of Fudan University, Shanghai 201508, China; Department of Endocrinology, Jinshan Hospital of Fudan University, Shanghai 201508, China. Electronic address: zhangjinan@hotmail.com.

Abstract

BACKGROUND: In the past several years, more and more studies proposed some concerns on the possibly increased risk of autoimmune diseases in individuals receiving vaccinations, but published studies on the associations of vaccinations with risks of systemic lupus erythematosus (SLE) and rheumatoid arthritis (RA) reported conflicting findings. A systematic review and meta-analysis was carried out to comprehensively evaluate the relationship between vaccinations and risk of SLE and RA.

METHODS: Pubmed, Web of Science and Embase were searched for observational studies assessing the associations of vaccinations with risks of RA and SLE. Two authors independently extracted data from those eligible studies. The quality of eligible studies was assessed by using the Newcastle-Ottawa Scale (NOS). The pooled relative risk (RR) with 95% confidence intervals (CIs) was used to measure the risk of RA and SLE associated with vaccinations, and was calculated through random-effect meta-analysis.

RESULTS: Sixteen observational studies were finally considered eligible, including 12 studies on the association between vaccinations and SLE risk and 13 studies on the association between vaccinations and RA risk. The pooled findings suggested that vaccinations significantly increased risk of SLE (RR=1.50; 95%CI 1.05-2.12, P=0.02). In addition, there was an obvious association between vaccinations and increased risk of RA (RR=1.32; 95%CI 1.09-1.60, P=0.004). Meta-analysis of studies reporting outcomes of short vaccinated time also suggested that vaccinations could significantly increase risk of SLE (RR=1.93; 95%CI 1.07-3.48, P=0.028) and RA (RR=1.48; 95%CI 1.08-2.03, P=0.015). Sensitivity analyses in studies with low risk of bias also found obvious associations of vaccinations with increased risk of RA and SLE.

CONCLUSION: This study suggests that vaccinations are related to increased risks of SLE and RA. More and larger observational studies are needed to further verify the findings above and to assess the associations of vaccinations with other rheumatic diseases. Copyright © 2017 Elsevier B.V. All rights reserved.

Publication Type

Journal Article. Meta-Analysis. Review. Systematic Review.

Year of Publication

2017

<177>

Unique Identifier

28465508

Title

CD8 T cells contribute to lacrimal gland pathology in the nonobese diabetic mouse model of Sjogren syndrome.

Source

Immunology & Cell Biology. 95(8):684-694, 2017 09.

VI 1

Status

MEDLINE

Authors

Barr JY; Wang X; Meyerholz DK; Lieberman SM.

Authors Full Name

Barr, Jennifer Y; Wang, Xiaofang; Meyerholz, David K; Lieberman, Scott M.

Institution

Barr, Jennifer Y. Stead Family Department of Pediatrics, Carver College of Medicine, University of Iowa, Iowa City, IA, USA.

Wang, Xiaofang. Stead Family Department of Pediatrics, Carver College of Medicine, University of Iowa, Iowa City, IA, USA.

Meyerholz, David K. Department of Pathology, Carver College of Medicine, University of Iowa, Iowa City, IA, USA.

Lieberman, Scott M. Stead Family Department of Pediatrics, Carver College of Medicine, University of Iowa, Iowa City, IA, USA.

Lieberman, Scott M. Interdisciplinary Graduate Program in Immunology, University of Iowa, Iowa City, IA, USA.

Abstract

Sjogren syndrome is an autoimmune disease characterized by targeted destruction of the lacrimal and salivary glands resulting in symptoms of severe ocular and oral dryness. Despite its prevalence, the mechanisms driving autoimmune manifestations are unclear. In patients and in the nonobese diabetic (NOD) mouse model of Sjogren syndrome, lymphocytic infiltrates consist of CD4 and CD8 T cells, although the role of CD8 T cells in disease pathogenesis has been largely unexplored. Here, we evaluated the contribution of CD8 T cells to lacrimal and salivary gland autoimmunity. Within the lacrimal and salivary glands of NOD mice, CD8 T cells were proliferating, expressed an activated phenotype, and produced inflammatory cytokines. Transfer of purified CD8 T cells isolated from the cervical lymph nodes (LNs) of NOD mice into NOD-severe combined immunodeficiency recipients resulted in inflammation of the lacrimal glands, but

was not sufficient to cause inflammation of the salivary glands. Lacrimal gland-infiltrating CD8 T cells displayed a cytotoxic phenotype, and epithelial cell damage in the lacrimal glands was observed in recipients of CD8 T cells regardless of the presence of CD4 T cells. Collectively, our results demonstrate that CD8 T cells have a pathogenic role in lacrimal gland autoimmunity. The gland-specific pathogenicity of CD8 T cells makes them a valuable resource to further understand the mechanisms that discriminate lacrimal versus salivary gland autoimmunity and for the development of new therapeutics that target the early stages of disease.

Publication Type

Comparative Study. Journal Article. Research Support, U.S. Gov't, Non-P.H.S.. Research Support, Non-U.S. Gov't. Research Support, N.I.H., Extramural.

Year of Publication

2017

<178>

Unique Identifier

28460477

Title

Inhibition of lysophosphatidic acid receptor ameliorates Sjogren's syndrome in NOD mice.

Source

Oncotarget. 8(16):27240-27251, 2017 Apr 18.

VI 1

Status

MEDLINE

Authors

Park E; Kim D; Lee SM; Jun HS.

Authors Full Name

Park, Eunhye; Kim, Donghee; Lee, Song Mi; Jun, Hee-Sook.

Institution

Park, Eunhye. Lee Gil Ya Cancer and Diabetes Institute, Gachon University, Incheon, Republic of Korea.

Kim, Donghee. Lee Gil Ya Cancer and Diabetes Institute, Gachon University, Incheon, Republic of Korea.

Lee, Song Mi. Lee Gil Ya Cancer and Diabetes Institute, Gachon University, Incheon, Republic of Korea.
Lee, Song Mi. College of Pharmacy and Gachon Institute of Pharmaceutical Science, Gachon University, Incheon, Republic of Korea.
Jun, Hee-Sook. Lee Gil Ya Cancer and Diabetes Institute, Gachon University, Incheon, Republic of Korea.
Jun, Hee-Sook. College of Pharmacy and Gachon Institute of Pharmaceutical Science, Gachon University, Incheon, Republic of Korea.
Jun, Hee-Sook. Gil Medical Research Institute, Gil Hospital, Incheon, Republic of Korea.
Abstract
Lysophosphatidic acid (LPA), a bioactive lysophospholipid, is involved in the pathogenesis of chronic inflammatory and autoimmune diseases. In this study, we investigated the role of LPA/LPA receptor (LPAR) signaling in the pathogenesis of Sjogren's syndrome (SS). We found that autotaxin, an LPA producing enzyme, and LPAR1 and LPAR3 mRNA, and IL-17 mRNA were highly expressed in the exocrine glands of 20-week-old nonobese diabetic (NOD) mice, which show SS symptoms at this age, as compared with non-symptomatic 8-week-old NOD mice. In an adoptive transfer model using NOD lymphocytes, treatment with Ki16425, an LPAR1/3 antagonist, restored tear and saliva secretion and decreased symptoms of SS compared with the vehicle-treated group. IL-17 levels in serum and lacrimal glands were also significantly reduced by Ki16425 in recipient mice. In addition, Ki16425 treatment of 20-week-old NOD mice, which spontaneously developed SS, restored saliva volume. Treatment of NOD splenocytes with LPA induced the expression of IL-17 in a dose-dependent manner, and Ki16425 inhibited this increase. LPA stimulated the activation of ROCK2 and p38 MAPK; and inhibition of ROCK2 or p38 MAPK suppressed LPA-induced IL-17 expression. Our data suggest that LPAR signaling stimulates SS development by induction of IL-17 production via ROCK and p38 MAPK pathways. Thus, LPAR inhibition could be a possible therapeutic strategy for SS.
Publication Type
Journal Article.
Year of Publication
2017

<179>
Unique Identifier

28451787

Title

Future therapeutic targets in rheumatoid arthritis?. [Review]

Source

Seminars In Immunopathology. 39(4):487-500, 2017 06.

VI 1

Status

MEDLINE

Authors

Cheung TT; McInnes IB.

Authors Full Name

Cheung, Tommy Tsang; McInnes, Iain B.

Institution

Cheung, Tommy Tsang. Department of Medicine, Li Ka Shing Faculty of Medicine, Queen Mary Hospital, The University of Hong Kong, 102 Pokfulam Road, Hong Kong, Hong Kong.

McInnes, Iain B. Institute of Infection, Immunity and Inflammation, University of Glasgow, B4/13, GBRC, 120 University Place, Glasgow, G12 8TA, UK. Iain.McInnes@glasgow.ac.uk.

Abstract

Rheumatoid arthritis (RA) is a chronic inflammatory disease characterized by persistent joint inflammation. Without adequate treatment, patients with RA will develop joint deformity and progressive functional impairment. With the implementation of treat-to-target strategies and availability of biologic therapies, the outcomes for patients with RA have significantly improved. However, the unmet need in the treatment of RA remains high as some patients do not respond sufficiently to the currently available agents, remission is not always achieved and refractory disease is not uncommon. With better understanding of the pathophysiology of RA, new therapeutic approaches are emerging. Apart from more selective Janus kinase inhibition, there is a great interest in the granulocyte macrophage-colony stimulating factor pathway, Bruton's tyrosine kinase pathway, phosphoinositide-3-kinase pathway, neural stimulation and dendritic cell-based therapeutics. In this review, we will discuss the therapeutic potential of these novel approaches.

Publication Type

Journal Article. Review.

Year of Publication

2017

<180>
Unique Identifier
28422472
Title
[Post-vaccination varicella in a patient receiving methotrexate]. [Spanish]
Source
Revista Espanola de Quimioterapia. 30(3):236-238, 2017 Jun.
VI 1
Status
MEDLINE
Authors
Fernandez-Prada M; Brandy-Garcia AM; Rodriguez-Fonseca OD; Alvarez-Arguelles ME; Melon-Garcia S.
Authors Full Name
Fernandez-Prada, M; Brandy-Garcia, A M; Rodriguez-Fonseca, O D; Alvarez-Arguelles, M E; Melon-Garcia, S.
Institution
Fernandez-Prada, M. Maria Fernandez-Prada. Unidad de Vacunas. Servicio de Medicina Preventiva y Salud Publica del Hospital Universitario Central de Asturias. Avda Roma s/n, 33011, Oviedo, Spain. mariafdezprada@gmail.com.
Publication Type
Case Reports. Letter.
Year of Publication
2017

<181>
Unique Identifier
28381829
Title
Fine tuning of immunometabolism for the treatment of rheumatic diseases. [Review]
Source

Nature Reviews Rheumatology. 13(5):313-320, 2017 May.
VI 1
Status
MEDLINE
Authors
Rhoads JP; Major AS; Rathmell JC.
Authors Full Name
Rhoads, Jillian P; Major, Amy S; Rathmell, Jeffrey C.
Institution
Rhoads, Jillian P. Division of Molecular Pathology, Department of Pathology, Microbiology, and Immunology, Vanderbilt University Medical Center, 1161 21st Avenue South, Nashville, Tennessee 37232, USA.
Major, Amy S. Division of Molecular Pathology, Department of Pathology, Microbiology, and Immunology, Vanderbilt University Medical Center; the Division of Rheumatology and Immunology, Department of Medicine, Vanderbilt University Medical Center; and the Vanderbilt Center for Immunobiology, Vanderbilt University School of Medicine, 1161 21st Avenue South, Nashville, Tennessee 37232, USA; and at the Department for Veterans Affairs, Tennessee Valley Healthcare System, Nashville, Tennessee 37232, USA.
Rathmell, Jeffrey C. Division of Molecular Pathology, Department of Pathology, Microbiology, and Immunology, and the Vanderbilt Center for Immunobiology, Vanderbilt University School of Medicine, 1161 21st Avenue South, Vanderbilt University Medical Center, Nashville, Tennessee 37232, USA.
Abstract
All immune cells depend on specific and efficient metabolic pathways to mount an appropriate response. Over the past decade, the field of immunometabolism has expanded our understanding of the various means by which cells modulate metabolism to achieve the effector functions necessary to fight infection or maintain homeostasis. Harnessing these metabolic pathways to manipulate inappropriate immune responses as a therapeutic strategy in cancer and autoimmunity has received increasing scrutiny by the scientific community. Fine tuning immunometabolism to provide the desired response, or prevent a deleterious response, is an attractive alternative to chemotherapy or overt immunosuppression. The various metabolic pathways used by immune cells in rheumatoid arthritis, systemic lupus erythematosus and osteoarthritis offer numerous opportunities for selective targeting of specific immune cell subsets to manipulate cellular metabolism for therapeutic benefit in these rheumatologic diseases.
Publication Type
Journal Article. Review.
Year of Publication

2017

<182>

Unique Identifier

28364628

Title

Human adipose tissue-derived mesenchymal stem cells in rheumatoid arthritis: Regulatory effects on peripheral blood mononuclear cells activation.

Source

International Immunopharmacology. 47:59-69, 2017 Jun.

VI 1

Status

MEDLINE

Authors

Baharlou R; Ahmadi-Vasmehjani A; Faraji F; Atashzar MR; Khoubyari M; Ahi S; Erfanian S; Navabi SS.

Authors Full Name

Baharlou, Rasoul; Ahmadi-Vasmehjani, Abbas; Faraji, Fatemeh; Atashzar, Mohammad Reza; Khoubyari, Mahshid; Ahi, Salma; Erfanian, Saiedeh; Navabi, Shadi-Sadat.

Institution

Baharlou, Rasoul. Department of Immunology and Microbiology, School of Medicine, Jahrom University of Medical Sciences, Jahrom, Iran.

Ahmadi-Vasmehjani, Abbas. Department of Immunology and Microbiology, School of Medicine, Jahrom University of Medical Sciences, Jahrom, Iran. Electronic address: ahvasmehjani@gmail.com.

Faraji, Fatemeh. Department of Immunology and Microbiology, School of Medicine, Jahrom University of Medical Sciences, Jahrom, Iran.

Atashzar, Mohammad Reza. Department of Immunology and Microbiology, School of Medicine, Jahrom University of Medical Sciences, Jahrom, Iran.

Khoubyari, Mahshid. Department of Student Research Committee, School of Medicine, Jahrom University of Medical Sciences, Jahrom, Iran.

Ahi, Salma. Department of Internal Medicine, School of Medicine, Jahrom University of Medical Sciences, Jahrom, Iran.

Erfanian, Saiedeh. Research Center for Non-Communicable Diseases, School of Medicine, Jahrom University of Medical Sciences, Jahrom, Iran.
Navabi, Shadi-Sadat. Department of Immunology and Microbiology, School of Medicine, Jahrom University of Medical Sciences, Jahrom, Iran.
Abstract
BACKGROUND AND OBJECTIVES: Mesenchymal stem cells (MSCs) are multipotent adult stem cells with immunomodulatory properties. The mechanisms by which MSCs inhibit the proliferation of pro-inflammatory T cells have not been fully elucidated yet. It is assumed that pro-inflammatory T-cells play an important role in the development of autoimmune diseases. We investigated the potential therapeutic effects of human adipose tissue derived (Ad)-MSCs on the peripheral blood mononuclear cells (PBMCs) of rheumatoid arthritis (RA) patients and healthy individuals, with a particular focus on Th17-associated cytokines.

MATERIALS AND METHODS: PBMCs from RA patients and healthy donors were co-cultured with Ad-MSCs and HeLa with or without Phytohemagglutinin (PHA). Finally, IL-6, IL-17, IL-21, IL-23 and TGF-beta levels were determined by ELISA and quantitative real-time RT-PCR on co-culture supernatants and PBMCs, respectively.

RESULTS: In co-culture interaction, Ad-MSCs inhibited IL-17 secretion by PBMCs compared to unstimulated PBMCs cultured alone. In addition, IL-21 expressions in PBMCs of the patient group, and IL-17 and IL-21 in healthy group were inhibited by Ad-MSCs compared to PBMCs cultured alone. TGF-beta expression in healthy individuals remarkably increased in both MSC-treated groups with and without PHA in comparison to PHA-stimulated and -unstimulated PBMCs.

CONCLUSIONS: This study demonstrates that human Ad-MSCs act as key regulators of immune tolerance by inhibiting the inflammation. Therefore, they can be attractive candidates for immunomodulatory cell-based therapy in RA. Copyright © 2017. Published by Elsevier B.V.
Publication Type
Journal Article.
Year of Publication
2017

<183>
Unique Identifier
28343998
Title
Carnosic acid (CA) attenuates collagen-induced arthritis in db/db mice via inflammation suppression by regulating ROS-dependent p38 pathway.
Source
Free Radical Biology & Medicine. 108:418-432, 2017 07.
VI 1
Status
MEDLINE
Authors
Xia G; Wang X; Sun H; Qin Y; Fu M.
Authors Full Name
Xia, Guangtao; Wang, Xia; Sun, Hongsheng; Qin, Yuhong; Fu, Min.
Institution
Xia, Guangtao. Department of Rheumatology and Immunology, Shandong Provincial Hospital Affiliated to Shandong University, Jinan, Shandong 250021, PR China.
Wang, Xia. Department of Rheumatology and Immunology, Shandong Provincial Hospital Affiliated to Shandong University, Jinan, Shandong 250021, PR China.
Sun, Hongsheng. Department of Rheumatology and Immunology, Shandong Provincial Hospital Affiliated to Shandong University, Jinan, Shandong 250021, PR China.
Qin, Yuhong. School of Life Sciences, Tsinghua University, Beijing 100000, PR China.
Fu, Min. Department of Rheumatology and Immunology, Shandong Provincial Hospital Affiliated to Shandong University, Jinan, Shandong 250021, PR China. Electronic address: fumingshandong@126.com.
Abstract
Rheumatoid arthritis (RA) is a multifactorial autoimmune disease, characterized by inflammation of synovial joints. Carnosic acid (CA) is a phenolic diterpene isolated from Rosmarinus officinailis, playing a central role in cytoprotective responses to oxidative stress and inflammation response. Our study aimed to investigate the effects of CA on RA progression in diabetic animals. Carnosic acid (CA) was used to treat collagen-induced arthritis (CIA)-induced db/db mice. Blood glucose, oral glucose tolerance test (OGTT) and insulin tolerance test (ITT) were investigated to explore insulin resistance. CA significantly down-regulated fasting blood glucose, glucose level in OGTT and ITT, ameliorated CIA-induced bone loss, and reduced pro-inflammatory cytokines and reactive oxygen species (ROS) in db/db mice with arthritis induced by CIA. In vitro, CA suppressed Receptor Activator for Nuclear Factor-kappa B Ligand (RANKL)- and Macrophage

colony-stimulating factor (M-CSF)-induced osteoclastogenesis. The osteoclastic specific markers were inhibited by CA. Signal transduction studies showed that CA significantly decreased the expression of molecules contributing to ROS and increased anti-oxidants. Additionally, CA inactivated the RANKL- and M-CSF-induced p38 mitogen activated protein kinases (MAPK), inhibited NF-kappaB phosphorylation, causing pro-inflammatory cytokines down-regulation. Together, CA ameliorated osteoclast formation and CIA-induced bone loss in db/db mice through inflammation suppression by regulating ROS-dependent p38 pathway. Copyright © 2017. Published by Elsevier Inc.

Publication Type

Journal Article.

Year of Publication

2017

<184>

Unique Identifier

28320832

Title

G-CSF Receptor Blockade Ameliorates Arthritic Pain and Disease.

Source

Journal of Immunology. 198(9):3565-3575, 2017 05 01.

VI 1

Status

MEDLINE

Authors

Lee MC; McCubbin JA; Christensen AD; Poole DP; Rajasekhar P; Lieu T; Bunnett NW; Garcia-Caraballo S; Erickson A; Brierley SM; Saleh R; Achuthan A; Fleetwood AJ; Anderson RL; Hamilton JA; Cook AD.

Author NameID

Lee, Ming-Chin; ORCID: https://orcid.org/0000-0002-6609-3524
Rajasekhar, Pradeep; ORCID: https://orcid.org/0000-0002-1983-7244
Erickson, Andelain; ORCID: https://orcid.org/0000-0001-8134-7919
Saleh, Reem; ORCID: https://orcid.org/0000-0002-8292-1895
Achuthan, Adrian; ORCID: https://orcid.org/0000-0003-3640-2338

Anderson, Robin L; ORCID: https://orcid.org/0000-0002-6841-7422
Hamilton, John A; ORCID: https://orcid.org/0000-0002-9493-9224
Authors Full Name
Lee, Ming-Chin; McCubbin, James A; Christensen, Anne D; Poole, Daniel P; Rajasekhar, Pradeep; Lieu, TinaMarie; Bunnett, Nigel W; Garcia-Caraballo, Sonia; Erickson, Andelain; Brierley, Stuart M; Saleh, Reem; Achuthan, Adrian; Fleetwood, Andrew J; Anderson, Robin L; Hamilton, John A; Cook, Andrew D.
Institution
Lee, Ming-Chin. Department of Medicine, Royal Melbourne Hospital, University of Melbourne, Parkville, Victoria 3050, Australia.
McCubbin, James A. Department of Medicine, Royal Melbourne Hospital, University of Melbourne, Parkville, Victoria 3050, Australia.
Christensen, Anne D. Department of Medicine, Royal Melbourne Hospital, University of Melbourne, Parkville, Victoria 3050, Australia.
Poole, Daniel P. Monash Institute of Pharmaceutical Sciences, Monash University, Parkville, Victoria 3052, Australia.
Poole, Daniel P. Department of Anatomy and Neuroscience, University of Melbourne, Parkville, Victoria 3010, Australia.
Rajasekhar, Pradeep. Monash Institute of Pharmaceutical Sciences, Monash University, Parkville, Victoria 3052, Australia.
Lieu, TinaMarie. Monash Institute of Pharmaceutical Sciences, Monash University, Parkville, Victoria 3052, Australia.
Bunnett, Nigel W. Monash Institute of Pharmaceutical Sciences, Monash University, Parkville, Victoria 3052, Australia.
Bunnett, Nigel W. Department of Surgery, Columbia University, New York, NY 10032.
Garcia-Caraballo, Sonia. Centre for Nutrition and Gastrointestinal Diseases, Discipline of Medicine, University of Adelaide, Adelaide, South Australia 5000, Australia.
Garcia-Caraballo, Sonia. South Australian Health and Medical Research Institute, Adelaide, South Australia 5000, Australia.
Garcia-Caraballo, Sonia. Visceral Pain Group, Department of Human Physiology, Flinders University, Bedford Park, South Australia 5042, Australia.
Erickson, Andelain. Centre for Nutrition and Gastrointestinal Diseases, Discipline of Medicine, University of Adelaide, Adelaide, South Australia 5000, Australia.
Erickson, Andelain. South Australian Health and Medical Research Institute, Adelaide, South Australia 5000, Australia.
Erickson, Andelain. Visceral Pain Group, Department of Human Physiology, Flinders University, Bedford Park, South Australia 5042, Australia.

Brierley, Stuart M. Centre for Nutrition and Gastrointestinal Diseases, Discipline of Medicine, University of Adelaide, Adelaide, South Australia 5000, Australia.

Brierley, Stuart M. South Australian Health and Medical Research Institute, Adelaide, South Australia 5000, Australia.

Brierley, Stuart M. Visceral Pain Group, Department of Human Physiology, Flinders University, Bedford Park, South Australia 5042, Australia.

Saleh, Reem. Department of Medicine, Royal Melbourne Hospital, University of Melbourne, Parkville, Victoria 3050, Australia.

Achuthan, Adrian. Department of Medicine, Royal Melbourne Hospital, University of Melbourne, Parkville, Victoria 3050, Australia.

Fleetwood, Andrew J. Department of Medicine, Royal Melbourne Hospital, University of Melbourne, Parkville, Victoria 3050, Australia.

Anderson, Robin L. Olivia Newton-John Cancer Research Institute, Heidelberg, Victoria 3084, Australia; and.

Anderson, Robin L. School of Cancer Medicine, La Trobe University, Heidelberg, Victoria 3084, Australia.

Hamilton, John A. Department of Medicine, Royal Melbourne Hospital, University of Melbourne, Parkville, Victoria 3050, Australia; jahami@unimelb.edu.au.

Cook, Andrew D. Department of Medicine, Royal Melbourne Hospital, University of Melbourne, Parkville, Victoria 3050, Australia.

Abstract

G-CSF or CSF-3, originally defined as a regulator of granulocyte lineage development via its cell surface receptor (G-CSFR), can play a role in inflammation, and hence in many pathologies, due to its effects on mature lineage populations. Given this, and because pain is an extremely important arthritis symptom, the efficacy of an anti-G-CSFR mAb for arthritic pain and disease was compared with that of a neutrophil-depleting mAb, anti-Ly6G, in both adaptive and innate immune-mediated murine models. Pain and disease were ameliorated in Ag-induced arthritis, zymosan-induced arthritis, and methylated BSA/IL-1 arthritis by both prophylactic and therapeutic anti-G-CSFR mAb treatment, whereas only prophylactic anti-Ly6G mAb treatment was effective. Efficacy for pain and disease correlated with reduced joint neutrophil numbers and, importantly, benefits were noted without necessarily the concomitant reduction in circulating neutrophils. Anti-G-CSFR mAb also suppressed zymosan-induced inflammatory pain. A new G-CSF-driven (methylated BSA/G-CSF) arthritis model was established enabling us to demonstrate that pain was blocked by a cyclooxygenase-2 inhibitor, suggesting an indirect effect on neurons. Correspondingly, dorsal root ganglion neurons cultured in G-CSF failed to respond to G-CSF in vitro, and Csf3r gene expression could not be detected in dorsal root ganglion neurons by single-cell RT-PCR. These data suggest that G-CSFR/G-CSF targeting may be a safe therapeutic

strategy for arthritis and other inflammatory conditions, particularly those in which pain is important, as well as for inflammatory pain per se. Copyright © 2017 by The American Association of Immunologists, Inc.
Publication Type
Journal Article. Research Support, Non-U.S. Gov't.
Year of Publication
2017

<185>
Unique Identifier
28320444
Title
OX40 signaling is involved in the autoactivation of CD4+CD28- T cells and contributes to the pathogenesis of autoimmune arthritis.
Source
Arthritis Research & Therapy. 19(1):67, 2017 03 21.
VI 1
Status
MEDLINE
Authors
Jiang J; Liu C; Liu M; Shen Y; Hu X; Wang Q; Wu J; Wu M; Fang Q; Zhang X.
Authors Full Name
Jiang, Juean; Liu, Cuiping; Liu, Mi; Shen, Yu; Hu, Xiaohan; Wang, Qin; Wu, Jian; Wu, Min; Fang, Qi; Zhang, Xueguang.
Institution
Jiang, Juean. Jiangsu Institute of Clinical Immunology & Jiangsu Key Laboratory of Clinical Immunology, First Affiliated Hospital of Soochow University, No. 708 Renmin Road, Suzhou, 215006, Jiangsu, China.
Liu, Cuiping. Jiangsu Institute of Clinical Immunology & Jiangsu Key Laboratory of Clinical Immunology, First Affiliated Hospital of Soochow University, No. 708 Renmin Road, Suzhou, 215006, Jiangsu, China.

Liu, Mi. Jiangsu Institute of Clinical Immunology & Jiangsu Key Laboratory of Clinical Immunology, First Affiliated Hospital of Soochow University, No. 708 Renmin Road, Suzhou, 215006, Jiangsu, China.
Shen, Yu. Jiangsu Institute of Clinical Immunology & Jiangsu Key Laboratory of Clinical Immunology, First Affiliated Hospital of Soochow University, No. 708 Renmin Road, Suzhou, 215006, Jiangsu, China.
Hu, Xiaohan. Jiangsu Institute of Clinical Immunology & Jiangsu Key Laboratory of Clinical Immunology, First Affiliated Hospital of Soochow University, No. 708 Renmin Road, Suzhou, 215006, Jiangsu, China.
Wang, Qin. Jiangsu Institute of Clinical Immunology & Jiangsu Key Laboratory of Clinical Immunology, First Affiliated Hospital of Soochow University, No. 708 Renmin Road, Suzhou, 215006, Jiangsu, China.
Wu, Jian. Jiangsu Institute of Clinical Immunology & Jiangsu Key Laboratory of Clinical Immunology, First Affiliated Hospital of Soochow University, No. 708 Renmin Road, Suzhou, 215006, Jiangsu, China.
Wu, Min. Departments of Rheumatology, Third Affiliated Hospital of Soochow University, No. 185 Juqian Road, Changzhou, 213003, Jiangsu, China.
Fang, Qi. Jiangsu Institute of Clinical Immunology & Jiangsu Key Laboratory of Clinical Immunology, First Affiliated Hospital of Soochow University, No. 708 Renmin Road, Suzhou, 215006, Jiangsu, China. fangqi_008@126.com.
Zhang, Xueguang. Jiangsu Institute of Clinical Immunology & Jiangsu Key Laboratory of Clinical Immunology, First Affiliated Hospital of Soochow University, No. 708 Renmin Road, Suzhou, 215006, Jiangsu, China. xueguangzh@126.com.
Abstract

BACKGROUND: CD4+CD28- T cells exhibit autoreactive potential in autoimmune disorders, including rheumatoid arthritis (RA). It is not well known which costimulator functions as an alternative second signal in the activation of this subset after CD28 expression is downregulated. Tumor necrosis factor receptor superfamily member OX40 is a key costimulator in the activation of T cells. The aim of this study was to investigate the costimulatory effects of OX40 on CD4+CD28- T cells in autoimmune arthritis.

METHODS: Clinical samples were collected from patients with RA and control subjects. Collagen-induced arthritis (CIA) was induced with collagen type II (CII) in DBA/1 mice. The CD4+CD28-OX40+ T-cell subset and its cytokine production were detected by flow cytometry. After T-cell purification, adoptive transfer was performed in CIA mice. The regulatory role of OX40 was determined by blocking experiments in vitro and in vivo.

RESULTS: OX40 and OX40L were abnormally expressed in patients with RA and CIA mice. Further analysis showed that CD4+CD28-OX40+ T cells accumulated in patients with RA and in animal models. These cells produced higher levels of proinflammatory cytokines and were closely correlated with the clinicopathological features of the affected individuals. Adoptive transfer of CII-specific CD4+CD28-OX40+ T cells remarkably aggravated arthritic development and joint pathology in CIA mice. Moreover, OX40 blockade significantly reduced the proinflammatory responses and ameliorated arthritis development.

CONCLUSIONS: OX40 acts as an alternative costimulator of CD4+CD28- T cells and plays a pathogenic role in autoimmune arthritic development, suggesting that it is a potential target for immunomodulatory therapy of RA.

Publication Type

Journal Article. Research Support, Non-U.S. Gov't.

Year of Publication

2017

<186>

Unique Identifier

28314223

Title

GV1001 immunotherapy ameliorates joint inflammation in a murine model of rheumatoid arthritis by modifying collagen-specific T-cell responses and downregulating antigen-presenting cells.

Source

International Immunopharmacology. 46:186-193, 2017 May.

VI 1

Status

MEDLINE

Authors

Choi IA; Choi JY; Jung S; Basri F; Park S; Lee EY.

Authors Full Name

Choi, In Ah; Choi, Ji Yong; Jung, Sundo; Basri, Fathihah; Park, Seho; Lee, Eun Young.

Institution

Choi, In Ah. Division of Rheumatology, Department of Internal Medicine, Seoul National University College of Medicine, 101 Daehak-ro, Jongno-gu, Seoul 03080, Republic of Korea. Electronic address: iachoi@cbnu.ac.kr.

Choi, Ji Yong. Division of Rheumatology, Department of Internal Medicine, Seoul National University College of Medicine, 101 Daehak-ro, Jongno-gu, Seoul 03080, Republic of Korea.

Jung, Sundo. Department of Life Sciences, Korea University, Seoul 02841, Republic of Korea.

Basri, Fathihah. Department of Life Sciences, Korea University, Seoul 02841, Republic of Korea.

Park, Seho. Department of Life Sciences, Korea University, Seoul 02841, Republic of Korea. Electronic address: sehopark@korea.ac.kr.

Lee, Eun Young. Division of Rheumatology, Department of Internal Medicine, Seoul National University College of Medicine, 101 Daehak-ro, Jongno-gu, Seoul 03080, Republic of Korea. Electronic address: elee@snu.ac.kr.

Abstract

This study investigated whether GV1001 may be useful for treating rheumatoid arthritis (RA). Two collagen-induced arthritis (CIA) experiments showed that therapeutic, but not preventive, GV1001 treatment reduced the severity of joint inflammation in CIA. The third CIA experiment indicated that, compared to vehicle treatment, therapeutic GV1001 treatment was associated with a significantly smaller area under the curve for the overall clinical joint score over the 98day observation period (p<0.05). GV1001 treatment was also associated with lower Day 98 serum IL-6 levels (p<0.01) and histological joint scores (p<0.05). Moreover, splenocytes harvested from the GV1001-treated mice exhibited lower basal and collagen-stimulated production of IFN-gamma and IL-6 on Days 49 and 98 than the splenocytes from vehicle-treated mice. The fourth and fifth experiments indicated that earlier treatment resulted in a better response. In addition, human (THP-1) and murine (RAW 264.7) macrophages and fibroblast-like synoviocytes (FLS) from RA patients were used for in vitro analyses. GV1001 treatment of lipopolysaccharide-stimulated macrophages derived from THP-1 and RAW 264.7 monocytes significantly reduced TNF-alpha and IL-6 secretion (THP-1: all p<0.05; RAW 264.7: all p<0.01). However, GV1001 treatment did not affect IL-6 expression in TNFalpha-stimulated RA FLS. GV1001 reduced the clinical joint scores, serum IL-6 levels, and histological joint scores of mice with CIA. In addition, GV1001 lowered the collagen-stimulated IFN-gamma and IL-6 production of murine T-cells and reduced the TNF-alpha and IL-6 production of macrophages in vitro. Thus, GV1001 may ameliorate joint inflammation by modifying T-cell reactions to the triggering autoantigen and by reducing macrophage cytokine production. Copyright © 2017 The Author(s). Published by Elsevier B.V. All rights reserved.

Publication Type

Journal Article.

Year of Publication
  2017

<187>
Unique Identifier
  28285683
Title
  Autoimmune paraneoplastic syndromes associated to lung cancer: A systematic review of the literature. [Review]
Source
  Lung Cancer. 106:102-109, 2017 04.
VI 1
Status
  MEDLINE
Authors
  Durieux V; Coureau M; Meert AP; Berghmans T; Sculier JP.
Authors Full Name
  Durieux, Valerie; Coureau, Michelle; Meert, Anne-Pascale; Berghmans, Thierry; Sculier, Jean-Paul.
Institution
  Durieux, Valerie. Bibliotheque des Sciences de la Sante, Universite Libre de Bruxelles (ULB), Laboratoire Facultaire de Medecine Factuelle (ULB), Belgium.
  Coureau, Michelle. Service des Soins Intensifs et Urgences Oncologiques & Oncologie thoracique, Institut Jules Bordet, Centre des Tumeurs de l'Universite Libre de Bruxelles (ULB) et, Laboratoire Facultaire de Medecine Factuelle (ULB), Belgium.
  Meert, Anne-Pascale. Service des Soins Intensifs et Urgences Oncologiques & Oncologie thoracique, Institut Jules Bordet, Centre des Tumeurs de l'Universite Libre de Bruxelles (ULB) et, Laboratoire Facultaire de Medecine Factuelle (ULB), Belgium.
  Berghmans, Thierry. Service des Soins Intensifs et Urgences Oncologiques & Oncologie thoracique, Institut Jules Bordet, Centre des Tumeurs de l'Universite Libre de Bruxelles (ULB) et, Laboratoire Facultaire de Medecine Factuelle (ULB), Belgium.
  Sculier, Jean-Paul. Service des Soins Intensifs et Urgences Oncologiques & Oncologie thoracique, Institut Jules Bordet, Centre des Tumeurs de l'Universite Libre de Bruxelles (ULB) et,

Laboratoire Facultaire de Medecine Factuelle (ULB), Belgium. Electronic address: sculier@bordet.be.
Abstract
The development of new immune treatment in oncology and particularly for lung cancer may induce new complications, particularly activation or reactivation of auto-immune diseases. In this context, a systematic review on the auto-immune paraneoplastic syndromes associated with lung cancer appears useful. This article is the first of a series of five and deals with the methodology applied for the review and with renal and rheumatic syndromes. Copyright © 2017 Elsevier B.V. All rights reserved.
Publication Type
Journal Article. Review. Systematic Review.
Year of Publication
2017

<188>
Unique Identifier
28267648
Title
Expression of soluble CD83 in plasma from early-stage rheumatoid arthritis patients is not modified by anti-TNF-alpha therapy.
Source
Cytokine. 96:1-7, 2017 08.
VI 1
Status
MEDLINE
Authors
Kristensen AM; Stengaard-Pedersen K; Hetland ML; Horslev-Petersen K; Junker P; Ostergaard M; Hollsberg P; Deleuran B; Hvid M.
Authors Full Name
Kristensen, Anne-Mette; Stengaard-Pedersen, Kristian; Hetland, Merete Lund; Horslev-Petersen, Kim; Junker, Peter; Ostergaard, Mikkel; Hollsberg, Per; Deleuran, Bent; Hvid, Malene.
Institution
Kristensen, Anne-Mette. Dept. of Biomedicine, Aarhus University, Denmark.

Stengaard-Pedersen, Kristian. Dept. of Rheumatology, Aarhus University Hospital, Denmark; Dept. of Clinical Medicine, Aarhus University, Denmark.
Hetland, Merete Lund. The DANBIO Registry and Copenhagen Center for Arthritis Research, Center for Rheumatology and Spine Diseases, Rigshospitalet, Denmark; Dept. of Clinical Medicine, Faculty of Health and Medical Sciences, University of Copenhagen, Denmark.
Horslev-Petersen, Kim. King Christian 10th Hospital for the Rheumatic Diseases, and University of Southern Denmark, Denmark.
Junker, Peter. Dept. of Rheumatology, Odense University Hospital, Denmark.
Ostergaard, Mikkel. The DANBIO Registry and Copenhagen Center for Arthritis Research, Center for Rheumatology and Spine Diseases, Rigshospitalet, Denmark; Dept. of Clinical Medicine, Faculty of Health and Medical Sciences, University of Copenhagen, Denmark.
Hollsberg, Per. Dept. of Biomedicine, Aarhus University, Denmark.
Deleuran, Bent. Dept. of Biomedicine, Aarhus University, Denmark; Dept. of Rheumatology, Aarhus University Hospital, Denmark.
Hvid, Malene. Dept. of Biomedicine, Aarhus University, Denmark; Dept. of Clinical Medicine, Aarhus University, Denmark. Electronic address: malene.hvid@biomed.au.dk.
Abstract
Rheumatoid arthritis (RA) is an autoimmune disease which may lead to severe disabilities due to structural joint damage and extraarticular manifestations The dendritic cell marker CD83 belongs to the immunoglobulin superfamily and has previously been associated with autoimmune diseases. In RA the levels of soluble CD83 (sCD83) are elevated in synovial fluid, however little is known about CD83 expression and regulation in RA. Therefore, we studied how CD83 is expressed in RA and further evaluated the effect of anti-TNF-alpha therapy hereon. Early RA patients were randomized to conventional disease modifying anti-rheumatic drugs with or without additional anti-TNF-alpha therapy. Rheumatoid arthritis patients had increased levels of sCD83 in plasma compared with healthy volunteers. The increase in sCD83 plasma levels were unaffected by anti-TNF-alpha therapy. In chronic RA patients the levels of sCD83 were higher in synovial fluid than in plasma, and only a limited amount of membrane bound CD83 expression was detected on the surface of cells from peripheral blood and synovial fluid. Finally, confocal microscopy of RA synovial membranes revealed that CD83 was mainly localized intracellularly in a group of cells with diverse morphology including both antigen-presenting cells and non-antigen-presenting cells. Our findings demonstrate that early-stage RA patients have elevated levels of sCD83 in plasma and that anti-TNF-alpha treatment has no effect on the sCD83 plasma level. This suggest that in RA patients sCD83 regulation is beyond control of TNF-alpha. Copyright © 2017 Elsevier Ltd. All rights reserved.
Publication Type
Clinical Trial. Journal Article. Randomized Controlled Trial.

Year of Publication
2017

<189>
Unique Identifier
28230210
Title
Synergistic suppression of autoimmune arthritis through concurrent treatment with tolerogenic DC and MSC.
Source
Scientific Reports. 7:43188, 2017 02 23.
VI 1
Status
MEDLINE
Authors
Li R; Zhang Y; Zheng X; Peng S; Yuan K; Zhang X; Min W.
Authors Full Name
Li, Rong; Zhang, Yujuan; Zheng, Xiufen; Peng, Shanshan; Yuan, Keng; Zhang, Xusheng; Min, Weiping.
Institution
Li, Rong. College of Basic Medical Sciences and Institute of Immunotherapy of Nanchang University, and Jiangxi Academy of Medical Sciences, Nanchang, China.
Li, Rong. Jiangxi Provincial Key Laboratory of Immunotherapy, Nanchang, China.
Zhang, Yujuan. College of Basic Medical Sciences and Institute of Immunotherapy of Nanchang University, and Jiangxi Academy of Medical Sciences, Nanchang, China.
Zhang, Yujuan. Jiangxi Provincial Key Laboratory of Immunotherapy, Nanchang, China.
Zheng, Xiufen. Departments of Surgery, Pathology, and Oncology, University of Western Ontario, London, Canada.
Peng, Shanshan. College of Basic Medical Sciences and Institute of Immunotherapy of Nanchang University, and Jiangxi Academy of Medical Sciences, Nanchang, China.
Peng, Shanshan. Jiangxi Provincial Key Laboratory of Immunotherapy, Nanchang, China.
Yuan, Keng. College of Basic Medical Sciences and Institute of Immunotherapy of Nanchang University, and Jiangxi Academy of Medical Sciences, Nanchang, China.

Yuan, Keng. Jiangxi Provincial Key Laboratory of Immunotherapy, Nanchang, China.
Zhang, Xusheng. Departments of Surgery, Pathology, and Oncology, University of Western Ontario, London, Canada.
Min, Weiping. College of Basic Medical Sciences and Institute of Immunotherapy of Nanchang University, and Jiangxi Academy of Medical Sciences, Nanchang, China.
Min, Weiping. Jiangxi Provincial Key Laboratory of Immunotherapy, Nanchang, China.
Min, Weiping. Departments of Surgery, Pathology, and Oncology, University of Western Ontario, London, Canada.
Abstract
Rheumatoid arthritis (RA) is an autoimmune disease characterized by progressive immune-mediated joint deterioration. Current treatments are not antigen specific and are associated with various adverse. We have previously demonstrated that tolerogenic dendritic cells (Tol-DC) are potent antigen-specific immune regulators, which hold great promise in immunotherapy of autoimmune diseases. In this study, we aimed to develop new immunotherapy by combining Tol-DC and mesenchymal stem cells (MSC). We demonstrated that RelB gene silencing resulted in generation of Tol-DC that suppressed T cell responses and selectively promoted Treg generation. The combination of MSC synergized the tolerogenic capacity of Tol-DC in inhibition of T cell responses. In murine collagen-induced arthritis (CIA) model, we demonstrated that progression of arthritis was inhibited with administration of RelB gene-silenced Tol-DC or MSC. This therapeutic effect was remarkably enhanced with concurrent treatment of combination Tol-DC and MSC as demonstrated by improved clinical symptoms, decreased clinical scores and attenuated joint damage. These therapeutic effects were associated with suppression of CII-specific T cell responses, polarization of Th and inhibition of proinflammatory cytokines, and reduced cartilage degeneration. This study for the first time demonstrates a new approach to treat autoimmune inflammatory joint disease with concurrent treatment of RelB gene-silenced Tol-DC and MSC.
Publication Type
Journal Article. Research Support, Non-U.S. Gov't.
Year of Publication
2017

<190>
Unique Identifier
28214146

Title

Impact of anti-TNF therapy on NK cells function and on immunosurveillance against B-cell lymphomas.

Source

Journal of Autoimmunity. 80:56-64, 2017 Jun.

VI 1

Status

MEDLINE

Authors

Nocturne G; Boudaoud S; Ly B; Pascaud J; Paoletti A; Mariette X.

Authors Full Name

Nocturne, Gaetane; Boudaoud, Saida; Ly, Bineta; Pascaud, Juliette; Paoletti, Audrey; Mariette, Xavier.

Institution

Nocturne, Gaetane. INSERM U1184, Center for Immunology of Viral Infections and Autoimmune Diseases, Universite Paris-Sud, Le Kremlin-Bicetre, France; Department of Rheumatology, AP-HP, Hopitaux Universitaires Paris-Sud, Le Kremlin-Bicetre, France. Electronic address: gaetane.nocturne@aphp.fr.

Boudaoud, Saida. INSERM U1184, Center for Immunology of Viral Infections and Autoimmune Diseases, Universite Paris-Sud, Le Kremlin-Bicetre, France.

Ly, Bineta. INSERM U1184, Center for Immunology of Viral Infections and Autoimmune Diseases, Universite Paris-Sud, Le Kremlin-Bicetre, France.

Pascaud, Juliette. INSERM U1184, Center for Immunology of Viral Infections and Autoimmune Diseases, Universite Paris-Sud, Le Kremlin-Bicetre, France.

Paoletti, Audrey. INSERM U1184, Center for Immunology of Viral Infections and Autoimmune Diseases, Universite Paris-Sud, Le Kremlin-Bicetre, France.

Mariette, Xavier. INSERM U1184, Center for Immunology of Viral Infections and Autoimmune Diseases, Universite Paris-Sud, Le Kremlin-Bicetre, France; Department of Rheumatology, AP-HP, Hopitaux Universitaires Paris-Sud, Le Kremlin-Bicetre, France.

Abstract

OBJECTIVES: Rheumatoid arthritis (RA) is associated with an increased risk of lymphoma linked to activity of the disease. Immunosuppressive drugs have been suspected to induce an additional risk. Since, NK cells have been recently shown to participate to anti-lymphoma immunosurveillance, we aimed to assess if anti-TNF might impact their anti-lymphoma activity.

METHODS: NK cells have been assessed ex vivo in patients with RA treated with methotrexate (MTX) with or without anti-TNF. Phenotype has been studied by flow cytometry and function has

been assessed after NKp30-cross linking. NK have been cultured 6 days in presence of anti-TNF, TNF-R inhibitors or controls and phenotype has been studied. Then cytotoxicity against 2 B non-Hodgkin lymphoma cell lines [Farage (EBV+) and SU-DHL4 (EBV-)] was assessed.

RESULTS: Exposure to anti-TNF was associated with a decreased activation of NK cells. NK cells exhibited an impaired function in patients treated with anti-TNF compared to patients treated with MTX alone as assessed by the percentage of degranulation (20.9% [18.5-32.9] vs 31.3% [21.5-49.1], p = 0.04) and a decreased IFN-gamma secretion ((17.4% [8.9-25.9] vs to 29.7% [22.5-43.1], p = 0.007). In vitro, exposure to anti-TNF impaired NK cells function and impacted negatively anti-lymphoma activity. These effects may be the consequence of inhibition of TNFR1 signaling.

CONCLUSIONS: Thus, even if meta-analysis of randomized controlled trials and of registries have not demonstrated to date an increased risk of lymphoma with anti-TNF, cautious must be pursued concerning this possible side effect in patients with long-term anti-TNF exposure.
Copyright © 2017 Elsevier Ltd. All rights reserved.
Publication Type
Journal Article.
Year of Publication
2017

<191>
Unique Identifier
28167631
Title
Ex-Th17 (Nonclassical Th1) Cells Are Functionally Distinct from Classical Th1 and Th17 Cells and Are Not Constrained by Regulatory T Cells.
Source
Journal of Immunology. 198(6):2249-2259, 2017 03 15.
VI 1
Status
MEDLINE
Authors

Basdeo SA; Cluxton D; Sulaimani J; Moran B; Canavan M; Orr C; Veale DJ; Fearon U; Fletcher JM.
Author NameID
Basdeo, Sharee A; ORCID: https://orcid.org/0000-0001-5616-6665
Canavan, Mary; ORCID: https://orcid.org/0000-0003-2310-2563
Fletcher, Jean M; ORCID: https://orcid.org/0000-0002-0670-6659
Authors Full Name
Basdeo, Sharee A; Cluxton, Deborah; Sulaimani, Jamal; Moran, Barry; Canavan, Mary; Orr, Carl; Veale, Douglas J; Fearon, Ursula; Fletcher, Jean M.
Institution
Basdeo, Sharee A. School of Biochemistry and Immunology, Trinity Biomedical Sciences Institute, Trinity College Dublin, Dublin 2, Ireland.
Cluxton, Deborah. School of Biochemistry and Immunology, Trinity Biomedical Sciences Institute, Trinity College Dublin, Dublin 2, Ireland.
Sulaimani, Jamal. School of Biochemistry and Immunology, Trinity Biomedical Sciences Institute, Trinity College Dublin, Dublin 2, Ireland.
Moran, Barry. School of Biochemistry and Immunology, Trinity Biomedical Sciences Institute, Trinity College Dublin, Dublin 2, Ireland.
Canavan, Mary. Department of Rheumatology, Dublin Academic Medical Centre, St. Vincent's University Hospital, Dublin 4, Ireland; and.
Orr, Carl. Department of Rheumatology, Dublin Academic Medical Centre, St. Vincent's University Hospital, Dublin 4, Ireland; and.
Veale, Douglas J. Department of Rheumatology, Dublin Academic Medical Centre, St. Vincent's University Hospital, Dublin 4, Ireland; and.
Fearon, Ursula. Department of Rheumatology, Dublin Academic Medical Centre, St. Vincent's University Hospital, Dublin 4, Ireland; and.
Fletcher, Jean M. School of Biochemistry and Immunology, Trinity Biomedical Sciences Institute, Trinity College Dublin, Dublin 2, Ireland; jean.fletcher@tcd.ie.
Fletcher, Jean M. School of Medicine, Trinity Biomedical Sciences Institute, Trinity College Dublin, Dublin 2, Ireland.
Abstract
Th17 cells are an important therapeutic target in autoimmunity. However, it is known that Th17 cells exhibit considerable plasticity, particularly at sites of autoimmune inflammation. Th17 cells can switch to become ex-Th17 cells that no longer produce IL-17 but produce IFN-gamma. These ex-Th17 cells are also called nonclassical Th1 cells because of their ability to produce IFN-gamma, similar to Th1 cells; however, it is unclear whether they resemble Th1 or Th17 cells in terms of their function and regulation, and whether they have a pathogenic role in autoimmunity.

We compared the phenotypic and functional features of human Th17, Th1, and ex-Th17 cell populations. Our data showed that despite their loss of IL-17 expression, ex-Th17 cells were more polyfunctional in terms of cytokine production than either Th1 or bona fide Th17 cells, and produced increased amounts of proinflammatory cytokines. The proliferative brake on Th17 cells appeared to be lifted because ex-Th17 cells proliferated more than Th17 cells after stimulation. In contrast with Th1 and Th17 cells, ex-Th17 cells were highly resistant to suppression of proliferation and cytokines by regulatory T cells. Finally, we showed that ex-Th17 cells accumulated in the joints of rheumatoid arthritis patients. Taken together, these data indicate that human ex-Th17 cells are functionally distinct from Th1 and Th17 cells, and suggest that they may play a pathogenic role at sites of autoimmunity, such as the rheumatoid arthritis joint where they accumulate. These findings have implications for therapeutic strategies that target IL-17, because these may not inhibit pathogenic ex-Th17 cells. Copyright © 2017 by The American Association of Immunologists, Inc.

Publication Type

Journal Article. Research Support, Non-U.S. Gov't.

Year of Publication

2017

<192>

Unique Identifier

28158970

Title

Tumor necrosis factor alpha inhibitors have no effect on a human T-lymphotropic virus type-I (HTLV-I)-infected cell line from patients with HTLV-I-associated myelopathy.

Source

BMC Immunology. 18(1):7, 2017 02 03.

VI 1

Status

MEDLINE

Authors

Fukui S; Nakamura H; Takahashi Y; Iwamoto N; Hasegawa H; Yanagihara K; Nakamura T; Okayama A; Kawakami A.

Author NameID

Fukui, Shoichi; ORCID: https://orcid.org/0000-0001-5487-4692
Authors Full Name
Fukui, Shoichi; Nakamura, Hideki; Takahashi, Yoshiko; Iwamoto, Naoki; Hasegawa, Hiroo; Yanagihara, Katsunori; Nakamura, Tatsufumi; Okayama, Akihiko; Kawakami, Atsushi.
Institution
Fukui, Shoichi. Department of Immunology and Rheumatology, Nagasaki University Graduate School of Biomedical Sciences, Nagasaki, Japan.
Nakamura, Hideki. Department of Immunology and Rheumatology, Nagasaki University Graduate School of Biomedical Sciences, Nagasaki, Japan. nhideki@nagasaki-u.ac.jp.
Takahashi, Yoshiko. Department of Immunology and Rheumatology, Nagasaki University Graduate School of Biomedical Sciences, Nagasaki, Japan.
Iwamoto, Naoki. Department of Immunology and Rheumatology, Nagasaki University Graduate School of Biomedical Sciences, Nagasaki, Japan.
Hasegawa, Hiroo. Department of Laboratory Medicine, Nagasaki University Graduate School of Biomedical Sciences, Nagasaki, Japan.
Yanagihara, Katsunori. Department of Laboratory Medicine, Nagasaki University Graduate School of Biomedical Sciences, Nagasaki, Japan.
Nakamura, Tatsufumi. Department of Human Community, Faculty of Social Welfare, Nagasaki International University, Sasebo, Japan.
Okayama, Akihiko. Department of Rheumatology, Infectious Diseases and Laboratory Medicine, Faculty of Medicine, University of Miyazaki, Miyazaki, Japan.
Kawakami, Atsushi. Department of Immunology and Rheumatology, Nagasaki University Graduate School of Biomedical Sciences, Nagasaki, Japan.
Abstract
BACKGROUND: While tumor necrosis factor alpha (TNF-alpha) inhibitors (TNFi) and other biologics are very effective against autoimmune diseases, they can also cause infectious diseases. Therefore, it is important to clarify whether the TNFi sometimes used to treat patients with rheumatoid arthritis (RA) complicated with human T-lymphotropic virus type-I (HTLV-I) infection have the unintended side effect of promoting HTLV-I proliferation.

METHODS: We used the HTLV-I-infected cell line HCT-5, derived from spinal fluid cells of a patient with HTLV-I associated myelopathy, to evaluate the production of cytokines and chemokines, TNF-alpha receptor (TNFR), the expression of HTLV-I associated genes, the HTLV-I proviral load (PVL), the expression of HTLV-I structural protein, and apoptosis. We used Jurkat cells as a control.

RESULTS: Supernatants of HCT-5 showed time-dependent elevations of IL-6, RANTES and ICAM-1. HCT-5 supernatants treated with infliximab, adalimumab, etanercept (ETN), golimumab and certolizumab pegol showed no significant differences in the levels of these molecules compared to the control. Neither TNFR1 nor TNFR2 expression was altered by any TNFi treatment, relative to phosphate-buffered saline (PBS) treatment, with the exception that TNFR2 was significantly decreased and internalized in HCT-5 cells by ETN treatment. The HTLV-I associated genes Tax and HBZ and the PVL levels were not significantly changed. Immunofluorescence staining of HCT-5 for an HTLV-I-associated protein, GAG, was also not significantly different between any of the TNFi treatments and the PBS treatment. DNA ladders as an index of apoptosis were not detected. Apoptotic cells were not increased by the addition of any TNFi.

CONCLUSIONS: In vitro, TNFi did not affect the cytokine profiles, expression of associated genes and proteins, proviral load or apoptosis of HCT-5 cells. The results suggested that TNFi treatment of RA patients complicated with HTLV-I might have no effect on HTLV-I infection.

Publication Type

Journal Article. Research Support, Non-U.S. Gov't.

Year of Publication

2017

<193>

Unique Identifier

28145151

Title

Factors associated with influenza and pneumococcal vaccine uptake among rheumatoid arthritis patients in Denmark invited to participate in a pneumococcal vaccine trial (Immunovax_RA).

Source

Scandinavian Journal of Rheumatology. 46(6):446-453, 2017 Nov.

VI 1

Status

MEDLINE

Authors

Nguyen M; Lindegaard H; Hendricks O; Friis-Moller N.

Authors Full Name
Nguyen, Mtt; Lindegaard, H; Hendricks, O; Friis-Moller, N.
Institution
Nguyen, Mtt. a Department of Infectious Diseases, Odense University Hospital, Odense, Denmark.
Nguyen, Mtt. b Institute of Clinical Research, University of Southern Denmark, Odense, Denmark.
Nguyen, Mtt. c OPEN, Odense Patient data Explorative Network, Odense University Hospital, Odense, Denmark.
Lindegaard, H. d Department of Rheumatology, Odense University Hospital, Odense, Denmark.
Hendricks, O. e Department of Rheumatology, King Christian 10th Hospital for Rheumatic Diseases, Graasten, Denmark.
Hendricks, O. f Institute of Regional Research, University of Southern Denmark, Odense, Denmark.
Friis-Moller, N. a Department of Infectious Diseases, Odense University Hospital, Odense, Denmark.
Abstract
OBJECTIVE: This study investigates predictors of influenza and pneumococcal vaccine coverage among rheumatoid arthritis (RA) patients, and explores possible differences according to type of RA therapy.

METHOD: RA patients from two clinics in the region of Southern Denmark were informed about the survey during scheduled follow-up visits. The questionnaire included questions concerning previous influenza and pneumococcal vaccine uptake, attitudes about vaccination, and socio-demographic factors. Factors associated with recalled vaccine uptake were assessed by multivariate logistic regression.

RESULTS: A total of 192 RA patients completed the survey, 134 (70%) of whom were women and 90 (47%) were aged >= 65 years. Sixty-seven patients (35%) received conventional disease-modifying anti-rheumatic drugs (cDMARDs) and 125 (65%) combination therapy with biological disease-modifying anti-rheumatic drugs (bDMARDs). Self-reported uptake of vaccination against seasonal influenza ever was 59% overall; 57% among patients receiving cDMARDs and 61% in patients receiving bDMARDs. Self-reported vaccine uptake against pneumococcal diseases was only 6% overall. Older age, educational level, and information and recommendation by a specialist or general physician were positively associated with influenza vaccine uptake, while there was no significant difference in vaccine uptake according to RA treatment type. Reasons for

not being vaccinated included fear of adverse effects, lack of information and recommendation, and perception of good health.

CONCLUSION: We observed a low prevalence of influenza and in particular of pneumococcal vaccinations among RA patients receiving immunosuppressive drugs, with no difference in coverage according to type of RA therapy. More population-specific evidence to support recommendations is required to increase awareness among patients and physicians.
Publication Type
Journal Article.
Year of Publication
2017

<194>
Unique Identifier
28133957
Title
Efficacy and Safety of Vaccination in Pediatric Patients with Systemic Inflammatory Rheumatic Diseases: a systematic review of the literature. [Review]
Source
Acta Reumatologica Portuguesa. 42(1):8-16, 2017 Jan-Mar.
VI 1
Status
MEDLINE
Authors
Sousa S; Duarte AC; Cordeiro I; Ferreira J; Goncalves MJ; Meirinhos T; Rocha TM; Romao VC; Santos MJ.
Authors Full Name
Sousa, Sandra; Duarte, Ana Catarina; Cordeiro, Ines; Ferreira, Joana; Goncalves, Maria Joao; Meirinhos, Tiago; Rocha, Teresa Martins; Romao, Vasco C; Santos, Maria Jose.
Institution
Sousa, Sandra. Hospital Garcia de Orta.
Duarte, Ana Catarina. Hospital Garcia de Orta.
Cordeiro, Ines. Hospital Garcia de Orta.

Ferreira, Joana. Centro Hospitalar e Universitario de Coimbra.
Goncalves, Maria Joao. Hospital de Santa Maria, Centro Hospitalar Lisboa Norte.
Meirinhos, Tiago. Hospital Infante D. Pedro, Centro Hospitalar do Baixo Vouga.
Rocha, Teresa Martins. Hospital de Sao Joao, Centro Hospitalar de Sao Joao.
Romao, Vasco C. Hospital de Santa Maria, Centro Hospitalar Lisboa Norte.
Santos, Maria Jose. Hospital Garcia de Orta.

Abstract

INTRODUCTION: Children and adolescents with systemic rheumatic diseases have an increased risk of infections. Although some infections are vaccine-preventable, immunization among patients with juvenile rheumatic diseases is suboptimal, partly due to some doubts that still persist regarding its efficacy and safety in this patient population.

OBJECTIVES: To review the available evidence regarding the immunological response and the safety of vaccination in children and adolescents with systemic inflammatory rheumatic diseases (SIRD).

METHODS: A systematic review of the current literature until December 2014 using MEDLINE, EMBASE and abstracts from the American College of Rheumatology and European League Against Rheumatism congresses (2011-2014), complemented by hand search was performed. Eligible studies were identified and efficacy (seroprotection and/or seroconversion) and safety (reactions to vaccine and relapse of rheumatic disease) outcomes were extracted and summarized according to the type of vaccine.

RESULTS: Twenty-eight articles concerning vaccination in pediatric patients with SIRDs were found, that included almost 2100 children and adolescents, comprising nearly all standard vaccinations of the recommended immunization schedule. Children with SIRDs generally achieved seroprotection and seroconversion; nevertheless, the antibody levels were often lower when compared with healthy children. Glucocorticoids and conventional disease-modifying anti-rheumatic drugs do not seem to significantly hamper the immune responses, whereas TNF inhibitors may reduce antibody production, particularly in response to pneumococcal conjugate, influenza, meningococcal C and hepatitis A vaccine. There were no serious adverse events, nor evidence of a relevant worsening of the underlying rheumatic disease. Concerning live attenuated vaccines, the evidence is scarce, but no episodes of overt disease were reported, even in patients under biological therapy.

CONCLUSIONS: Existing literature demonstrates that vaccines are generally well tolerated and effective in stable SIRD patients, yet antibody titers are frequently lower than in healthy controls.

There is some evidence that biological therapy could hamper the immune response. Data on safety of live attenuated vaccines is limited. Although the available literature covers most vaccines included in the national immunization plan, there is a need for more information regarding new vaccines and new anti-rheumatic therapies.

Publication Type

Journal Article. Review. Systematic Review.

Year of Publication

2017

<195>

Unique Identifier

28108005

Title

In vitro allogeneic immune cell response to mesenchymal stromal cells derived from human adipose in patients with rheumatoid arthritis.

Source

Cellular Immunology. 314:18-25, 2017 04.

VI 1

Status

MEDLINE

Authors

Usha Shalini P; Vidyasagar JV; Kona LK; Ponnana M; Chelluri LK.

Authors Full Name

Usha Shalini, P; Vidyasagar, J V S; Kona, Lakshmi Kumari; Ponnana, Meenakshi; Chelluri, Lakshmi Kiran.

Institution

Usha Shalini, P. Transplant Biology, Immunology & Stem Cell Unit, Global Hospitals, Hyderabad, India.

Vidyasagar, J V S. Department of Orthopedics, Aware Global Hospitals, Hyderabad, India.

Kona, Lakshmi Kumari. Department of Bariatric Surgery, Global Hospitals, Hyderabad, India.

Ponnana, Meenakshi. Transplant Biology, Immunology & Stem Cell Unit, Global Hospitals, Hyderabad, India.

Chelluri, Lakshmi Kiran. Transplant Biology, Immunology & Stem Cell Unit, Global Hospitals, Hyderabad, India. Electronic address: lkiran@globalhospitalsindia.com.
Abstract
We investigated the regulatory activity of human adipose-derived mesenchymal stromal cells (MSCs) (n=10) towards immune cells in a cohort of 84 rheumatoid arthritis (RA) patients, 36 apparently healthy controls. We co-cultured MSCs with lymphocyte subsets of T, B, and T regulatory cells (Tregs). Levels of the pro- and anti-inflammatory markers (tumor necrosis factor alpha (TNF-alpha), interferon gamma (IFN-gamma), and interleukin-10 (IL-10)) were estimated in serum and co-culture supernatants. The study revealed a two-fold increase in the proportion of Tregs and an increased level of CD4+CD25+FoxP3. MSCs altered T cell, B cell, and Treg cytokine production during an anti-inflammatory immune response. The MSCs inhibited CD3+T cell-mediated TNF-alpha secretion, upregulated IL-10, and suppressed the production of autoantibodies against citrullinated protein antigens produced by B cells. These data offer insight into the interactions between allogeneic MSCs and immune cells, and elucidate the dose-dependent modulation of MSCs. Copyright © 2017 Elsevier Inc. All rights reserved.
Publication Type
Journal Article.
Year of Publication
2017

<196>
Unique Identifier
28101910
Title
A practical approach to vaccination of patients with autoimmune inflammatory rheumatic diseases in Australia. [Review]
Source
Internal Medicine Journal. 47(5):491-500, 2017 May.
VI 1
Status
MEDLINE
Authors

Wong PKK; Bagga H; Barrett C; Hanrahan P; Johnson D; Katrib A; Leder K; Marabani M; Pentony P; Riordan J; White R; Young L.
Authors Full Name
Wong, Peter K K; Bagga, Hanish; Barrett, Claire; Hanrahan, Paddy; Johnson, Doug; Katrib, Amel; Leder, Karin; Marabani, Mona; Pentony, Peta; Riordan, John; White, Ray; Young, Laurel.
Institution
Wong, Peter K K. Mid-North Coast Arthritis Clinic, Coffs Harbour, New South Wales, Australia.
Wong, Peter K K. UNSW Rural Clinical School, Coffs Harbour, New South Wales, Australia.
Bagga, Hanish. Mid-North Coast Arthritis Clinic, Coffs Harbour, New South Wales, Australia.
Barrett, Claire. Department of Rheumatology, Redcliffe Hospital, Brisbane, Queensland, Australia.
Barrett, Claire. Redcliffe Northside Rheumatology, Brisbane, Queensland, Australia.
Hanrahan, Paddy. Private Rheumatology Practice, South Perth, Western Australia, Australia.
Hanrahan, Paddy. Faculty of Medicine, University of Western Australia, South Perth, Western Australia, Australia.
Johnson, Doug. Department of General Medicine and Infectious Diseases, Austin Health, Melbourne, Victoria, Australia.
Katrib, Amel. Department of Rheumatology, Prince of Wales Hospital, Sydney, New South Wales, Australia.
Leder, Karin. School of Public Health and Preventive Medicine, Monash University, Melbourne, Victoria, Australia.
Leder, Karin. Victorian Infectious Disease Service, Royal Melbourne Hospital at the Doherty Institute for Infection and Immunity, Melbourne, Victoria, Australia.
Marabani, Mona. Private Rheumatology Practice, Royal Prince Alfred Hospital, Sydney, New South Wales, Australia.
Pentony, Peta. Mid-North Coast Arthritis Clinic, Coffs Harbour, New South Wales, Australia.
Pentony, Peta. Institute of Rheumatology and Orthopaedics, Royal Prince Alfred Hospital, Sydney, New South Wales, Australia.
Riordan, John. Illawarra Rheumatology and University of Wollongong Graduate School of Medicine, Wollongong, New South Wales, Australia.
White, Ray. Private Rheumatology Practice, Sydney, New South Wales, Australia.
Young, Laurel. Department of Rheumatology, Redcliffe Hospital, Brisbane, Queensland, Australia.
Young, Laurel. Redcliffe Northside Rheumatology, Brisbane, Queensland, Australia.
Comments
Comment in (CIN)
Comment in (CIN)

Abstract

Autoimmune inflammatory rheumatic diseases (AIIRD), such as rheumatoid arthritis, psoriatic arthritis and ankylosing spondylitis are often complicated by infection, which results in significant morbidity and mortality. The increased risk of infection is probably due to a combination of immunosuppressive effects of the AIIRD, comorbidities and the use of immunosuppressive conventional synthetic disease-modifying anti-rheumatic drugs (DMARDs) and more recently, targeted synthetic DMARDs and biologic DMARDs that block specific pro-inflammatory enzymes, cytokines or cell types. The use of these various DMARDs has revolutionised the treatment of AIIRD. This has led to a marked improvement in quality of life for AIIRD patients, who often now travel for prolonged periods. Many infections are preventable with vaccination. However, as protective immune responses induced by vaccination may be impaired by immunosuppression, where possible, vaccination may need to be performed prior to initiation of immunosuppression. Vaccination status should also be reviewed when planning overseas travel. Limited data regarding vaccine efficacy in patients with AIIRD make prescriptive guidelines difficult. However, a vaccination history should be part of the initial work-up in all AIIRD patients. Those caring for AIIRD patients should regularly consider vaccination to prevent infection within the practicalities of routine clinical practice. Copyright © 2017 Royal Australasian College of Physicians.

Publication Type

Journal Article. Review.

Year of Publication

2017

<197>

Unique Identifier

28099084

Title

Mechanisms and New Strategies for Primary Sjogren's Syndrome. [Review]

Source

Annual Review of Medicine. 68:331-343, 2017 01 14.

VI 1

Status

MEDLINE

Authors

Mavragani CP.
Authors Full Name
Mavragani, Clio P.
Institution
Mavragani, Clio P. Department of Physiology, School of Medicine and Joint Academic Rheumatology Program, National and Kapodistrian University of Athens, Athens, Greece; email: kmauragan@med.uoa.gr.
Abstract
Primary Sjogren's syndrome (SS) is a common chronic autoimmune disease characterized by lymphocytic infiltration of exocrine glands, mainly salivary and lacrimal, resulting in oral and ocular dryness, although virtually any organ system can be affected. SS-related systemic manifestations are classified as either related to the presence of periepithelial infiltrates in exocrine and parenchymal organs or resulting from immunocomplex deposition due to B cell hyperactivity with increased risk for B cell lymphoma development. Activation of both innate and adaptive immune pathways contributes to disease pathogenesis, with prominent interferon (IFN) signatures identified in both peripheral blood and affected salivary gland tissues. Recently, LINE-1 genomic repeat elements have been proposed as potential triggers of type I IFN pathway activation in SS through activation of Toll-like receptor-dependent and -independent pathways. In view of the increasingly appreciated variability of SS, elucidation of distinct operating pathways in relation to diverse clinical phenotypes and selection of the optimal therapeutic intervention remain major challenges. Inhibition of cathepsin S molecules, blockade of costimulation through administration of abatacept and inhibitors of B7-related molecules and CD40, blockade of B cell function and B cell survival factors, and disruption of the formation of ectopic germinal centers are considered the main therapeutic targets. Well-controlled multicenter clinical trials are ongoing and data are awaited.
Publication Type
Journal Article. Review.
Year of Publication
2017

<198>

Unique Identifier
28042126

Title

B Cell Depletion Therapy Normalizes Circulating Follicular Th Cells in Primary Sjogren Syndrome.

Source

Journal of Rheumatology. 44(1):49-58, 2017 01.

VI 1

Status

MEDLINE

Authors

Verstappen GM; Kroese FG; Meiners PM; Corneth OB; Huitema MG; Haacke EA; van der Vegt B; Arends S; Vissink A; Bootsma H; Abdulahad WH.

Authors Full Name

Verstappen, Gwenny M; Kroese, Frans G M; Meiners, Petra M; Corneth, Odilia B; Huitema, Minke G; Haacke, Erlin A; van der Vegt, Bert; Arends, Suzanne; Vissink, Arjan; Bootsma, Hendrika; Abdulahad, Wayel H.

Institution

Verstappen, Gwenny M. From the departments of Rheumatology and Clinical Immunology, Oral and Maxillofacial Surgery, and Pathology and Medical Biology, University of Groningen, University Medical Center Groningen, Groningen; the Department of Pulmonary Medicine, Erasmus MC, Rotterdam, the Netherlands. g.m.p.j.verstappen@umcg.nl.

Verstappen, Gwenny M. G.M. Verstappen, MSc, departments of Rheumatology and Clinical Immunology, University of Groningen, University Medical Center Groningen; F.G. Kroese, PhD, departments of Rheumatology and Clinical Immunology, University of Groningen, University Medical Center Groningen; P.M. Meiners, MD, PhD, DMD, Oral and Maxillofacial Surgery, University of Groningen, University Medical Center Groningen; O.B. Corneth, PhD, Department of Pulmonary Medicine, Erasmus MC; M.G. Huitema, BSc, departments of Rheumatology and Clinical Immunology, University of Groningen, University Medical Center Groningen; E.A. Haacke, MD, departments of Rheumatology and Clinical Immunology, and departments of Pathology and Medical Biology, University of Groningen, University Medical Center Groningen; B. van der Vegt, MD, PhD, Department of Pathology and Medical Biology, University of Groningen, University Medical Center Groningen; S. Arends, PhD, departments of Rheumatology and Clinical Immunology, University of Groningen, University Medical Center Groningen; A. Vissink, DMD, MD, PhD, Oral and Maxillofacial Surgery, University of Groningen, University Medical Center Groningen; H. Bootsma, MD, PhD, departments of Rheumatology and Clinical Immunology, University of Groningen, University Medical Center Groningen; W.H. Abdulahad, PhD, departments of Rheumatology and Clinical Immunology, University of Groningen, University Medical Center Groningen. g.m.p.j.verstappen@umcg.nl.

Kroese, Frans G M. From the departments of Rheumatology and Clinical Immunology, Oral and Maxillofacial Surgery, and Pathology and Medical Biology, University of Groningen, University Medical Center Groningen, Groningen; the Department of Pulmonary Medicine, Erasmus MC, Rotterdam, the Netherlands.

Kroese, Frans G M. G.M. Verstappen, MSc, departments of Rheumatology and Clinical Immunology, University of Groningen, University Medical Center Groningen; F.G. Kroese, PhD, departments of Rheumatology and Clinical Immunology, University of Groningen, University Medical Center Groningen; P.M. Meiners, MD, PhD, DMD, Oral and Maxillofacial Surgery, University of Groningen, University Medical Center Groningen; O.B. Corneth, PhD, Department of Pulmonary Medicine, Erasmus MC; M.G. Huitema, BSc, departments of Rheumatology and Clinical Immunology, University of Groningen, University Medical Center Groningen; E.A. Haacke, MD, departments of Rheumatology and Clinical Immunology, and departments of Pathology and Medical Biology, University of Groningen, University Medical Center Groningen; B. van der Vegt, MD, PhD, Department of Pathology and Medical Biology, University of Groningen, University Medical Center Groningen; S. Arends, PhD, departments of Rheumatology and Clinical Immunology, University of Groningen, University Medical Center Groningen; A. Vissink, DMD, MD, PhD, Oral and Maxillofacial Surgery, University of Groningen, University Medical Center Groningen; H. Bootsma, MD, PhD, departments of Rheumatology and Clinical Immunology, University of Groningen, University Medical Center Groningen; W.H. Abdulahad, PhD, departments of Rheumatology and Clinical Immunology, University of Groningen, University Medical Center Groningen.

Meiners, Petra M. From the departments of Rheumatology and Clinical Immunology, Oral and Maxillofacial Surgery, and Pathology and Medical Biology, University of Groningen, University Medical Center Groningen, Groningen; the Department of Pulmonary Medicine, Erasmus MC, Rotterdam, the Netherlands.

Meiners, Petra M. G.M. Verstappen, MSc, departments of Rheumatology and Clinical Immunology, University of Groningen, University Medical Center Groningen; F.G. Kroese, PhD, departments of Rheumatology and Clinical Immunology, University of Groningen, University Medical Center Groningen; P.M. Meiners, MD, PhD, DMD, Oral and Maxillofacial Surgery, University of Groningen, University Medical Center Groningen; O.B. Corneth, PhD, Department of Pulmonary Medicine, Erasmus MC; M.G. Huitema, BSc, departments of Rheumatology and Clinical Immunology, University of Groningen, University Medical Center Groningen; E.A. Haacke, MD, departments of Rheumatology and Clinical Immunology, and departments of Pathology and Medical Biology, University of Groningen, University Medical Center Groningen; B. van der Vegt, MD, PhD, Department of Pathology and Medical Biology, University of Groningen, University Medical Center Groningen; S. Arends, PhD, departments of Rheumatology and Clinical Immunology, University of Groningen, University Medical Center Groningen; A. Vissink, DMD,

MD, PhD, Oral and Maxillofacial Surgery, University of Groningen, University Medical Center Groningen; H. Bootsma, MD, PhD, departments of Rheumatology and Clinical Immunology, University of Groningen, University Medical Center Groningen; W.H. Abdulahad, PhD, departments of Rheumatology and Clinical Immunology, University of Groningen, University Medical Center Groningen.

Corneth, Odilia B. From the departments of Rheumatology and Clinical Immunology, Oral and Maxillofacial Surgery, and Pathology and Medical Biology, University of Groningen, University Medical Center Groningen, Groningen; the Department of Pulmonary Medicine, Erasmus MC, Rotterdam, the Netherlands.

Corneth, Odilia B. G.M. Verstappen, MSc, departments of Rheumatology and Clinical Immunology, University of Groningen, University Medical Center Groningen; F.G. Kroese, PhD, departments of Rheumatology and Clinical Immunology, University of Groningen, University Medical Center Groningen; P.M. Meiners, MD, PhD, DMD, Oral and Maxillofacial Surgery, University of Groningen, University Medical Center Groningen; O.B. Corneth, PhD, Department of Pulmonary Medicine, Erasmus MC; M.G. Huitema, BSc, departments of Rheumatology and Clinical Immunology, University of Groningen, University Medical Center Groningen; E.A. Haacke, MD, departments of Rheumatology and Clinical Immunology, and departments of Pathology and Medical Biology, University of Groningen, University Medical Center Groningen; B. van der Vegt, MD, PhD, Department of Pathology and Medical Biology, University of Groningen, University Medical Center Groningen; S. Arends, PhD, departments of Rheumatology and Clinical Immunology, University of Groningen, University Medical Center Groningen; A. Vissink, DMD, MD, PhD, Oral and Maxillofacial Surgery, University of Groningen, University Medical Center Groningen; H. Bootsma, MD, PhD, departments of Rheumatology and Clinical Immunology, University of Groningen, University Medical Center Groningen; W.H. Abdulahad, PhD, departments of Rheumatology and Clinical Immunology, University of Groningen, University Medical Center Groningen.

Huitema, Minke G. From the departments of Rheumatology and Clinical Immunology, Oral and Maxillofacial Surgery, and Pathology and Medical Biology, University of Groningen, University Medical Center Groningen, Groningen; the Department of Pulmonary Medicine, Erasmus MC, Rotterdam, the Netherlands.

Huitema, Minke G. G.M. Verstappen, MSc, departments of Rheumatology and Clinical Immunology, University of Groningen, University Medical Center Groningen; F.G. Kroese, PhD, departments of Rheumatology and Clinical Immunology, University of Groningen, University Medical Center Groningen; P.M. Meiners, MD, PhD, DMD, Oral and Maxillofacial Surgery, University of Groningen, University Medical Center Groningen; O.B. Corneth, PhD, Department of Pulmonary Medicine, Erasmus MC; M.G. Huitema, BSc, departments of Rheumatology and Clinical Immunology, University of Groningen, University Medical Center Groningen; E.A.

Haacke, MD, departments of Rheumatology and Clinical Immunology, and departments of Pathology and Medical Biology, University of Groningen, University Medical Center Groningen; B. van der Vegt, MD, PhD, Department of Pathology and Medical Biology, University of Groningen, University Medical Center Groningen; S. Arends, PhD, departments of Rheumatology and Clinical Immunology, University of Groningen, University Medical Center Groningen; A. Vissink, DMD, MD, PhD, Oral and Maxillofacial Surgery, University of Groningen, University Medical Center Groningen; H. Bootsma, MD, PhD, departments of Rheumatology and Clinical Immunology, University of Groningen, University Medical Center Groningen; W.H. Abdulahad, PhD, departments of Rheumatology and Clinical Immunology, University of Groningen, University Medical Center Groningen.

Haacke, Erlin A. From the departments of Rheumatology and Clinical Immunology, Oral and Maxillofacial Surgery, and Pathology and Medical Biology, University of Groningen, University Medical Center Groningen, Groningen; the Department of Pulmonary Medicine, Erasmus MC, Rotterdam, the Netherlands.

Haacke, Erlin A. G.M. Verstappen, MSc, departments of Rheumatology and Clinical Immunology, University of Groningen, University Medical Center Groningen; F.G. Kroese, PhD, departments of Rheumatology and Clinical Immunology, University of Groningen, University Medical Center Groningen; P.M. Meiners, MD, PhD, DMD, Oral and Maxillofacial Surgery, University of Groningen, University Medical Center Groningen; O.B. Corneth, PhD, Department of Pulmonary Medicine, Erasmus MC; M.G. Huitema, BSc, departments of Rheumatology and Clinical Immunology, University of Groningen, University Medical Center Groningen; E.A. Haacke, MD, departments of Rheumatology and Clinical Immunology, and departments of Pathology and Medical Biology, University of Groningen, University Medical Center Groningen; B. van der Vegt, MD, PhD, Department of Pathology and Medical Biology, University of Groningen, University Medical Center Groningen; S. Arends, PhD, departments of Rheumatology and Clinical Immunology, University of Groningen, University Medical Center Groningen; A. Vissink, DMD, MD, PhD, Oral and Maxillofacial Surgery, University of Groningen, University Medical Center Groningen; H. Bootsma, MD, PhD, departments of Rheumatology and Clinical Immunology, University of Groningen, University Medical Center Groningen; W.H. Abdulahad, PhD, departments of Rheumatology and Clinical Immunology, University of Groningen, University Medical Center Groningen.

van der Vegt, Bert. From the departments of Rheumatology and Clinical Immunology, Oral and Maxillofacial Surgery, and Pathology and Medical Biology, University of Groningen, University Medical Center Groningen, Groningen; the Department of Pulmonary Medicine, Erasmus MC, Rotterdam, the Netherlands.

van der Vegt, Bert. G.M. Verstappen, MSc, departments of Rheumatology and Clinical Immunology, University of Groningen, University Medical Center Groningen; F.G. Kroese, PhD,

departments of Rheumatology and Clinical Immunology, University of Groningen, University Medical Center Groningen; P.M. Meiners, MD, PhD, DMD, Oral and Maxillofacial Surgery, University of Groningen, University Medical Center Groningen; O.B. Corneth, PhD, Department of Pulmonary Medicine, Erasmus MC; M.G. Huitema, BSc, departments of Rheumatology and Clinical Immunology, University of Groningen, University Medical Center Groningen; E.A. Haacke, MD, departments of Rheumatology and Clinical Immunology, and departments of Pathology and Medical Biology, University of Groningen, University Medical Center Groningen; B. van der Vegt, MD, PhD, Department of Pathology and Medical Biology, University of Groningen, University Medical Center Groningen; S. Arends, PhD, departments of Rheumatology and Clinical Immunology, University of Groningen, University Medical Center Groningen; A. Vissink, DMD, MD, PhD, Oral and Maxillofacial Surgery, University of Groningen, University Medical Center Groningen; H. Bootsma, MD, PhD, departments of Rheumatology and Clinical Immunology, University of Groningen, University Medical Center Groningen; W.H. Abdulahad, PhD, departments of Rheumatology and Clinical Immunology, University of Groningen, University Medical Center Groningen.

Arends, Suzanne. From the departments of Rheumatology and Clinical Immunology, Oral and Maxillofacial Surgery, and Pathology and Medical Biology, University of Groningen, University Medical Center Groningen, Groningen; the Department of Pulmonary Medicine, Erasmus MC, Rotterdam, the Netherlands.

Arends, Suzanne. G.M. Verstappen, MSc, departments of Rheumatology and Clinical Immunology, University of Groningen, University Medical Center Groningen; F.G. Kroese, PhD, departments of Rheumatology and Clinical Immunology, University of Groningen, University Medical Center Groningen; P.M. Meiners, MD, PhD, DMD, Oral and Maxillofacial Surgery, University of Groningen, University Medical Center Groningen; O.B. Corneth, PhD, Department of Pulmonary Medicine, Erasmus MC; M.G. Huitema, BSc, departments of Rheumatology and Clinical Immunology, University of Groningen, University Medical Center Groningen; E.A. Haacke, MD, departments of Rheumatology and Clinical Immunology, and departments of Pathology and Medical Biology, University of Groningen, University Medical Center Groningen; B. van der Vegt, MD, PhD, Department of Pathology and Medical Biology, University of Groningen, University Medical Center Groningen; S. Arends, PhD, departments of Rheumatology and Clinical Immunology, University of Groningen, University Medical Center Groningen; A. Vissink, DMD, MD, PhD, Oral and Maxillofacial Surgery, University of Groningen, University Medical Center Groningen; H. Bootsma, MD, PhD, departments of Rheumatology and Clinical Immunology, University of Groningen, University Medical Center Groningen; W.H. Abdulahad, PhD, departments of Rheumatology and Clinical Immunology, University of Groningen, University Medical Center Groningen.

Vissink, Arjan. From the departments of Rheumatology and Clinical Immunology, Oral and Maxillofacial Surgery, and Pathology and Medical Biology, University of Groningen, University Medical Center Groningen, Groningen; the Department of Pulmonary Medicine, Erasmus MC, Rotterdam, the Netherlands.

Vissink, Arjan. G.M. Verstappen, MSc, departments of Rheumatology and Clinical Immunology, University of Groningen, University Medical Center Groningen; F.G. Kroese, PhD, departments of Rheumatology and Clinical Immunology, University of Groningen, University Medical Center Groningen; P.M. Meiners, MD, PhD, DMD, Oral and Maxillofacial Surgery, University of Groningen, University Medical Center Groningen; O.B. Corneth, PhD, Department of Pulmonary Medicine, Erasmus MC; M.G. Huitema, BSc, departments of Rheumatology and Clinical Immunology, University of Groningen, University Medical Center Groningen; E.A. Haacke, MD, departments of Rheumatology and Clinical Immunology, and departments of Pathology and Medical Biology, University of Groningen, University Medical Center Groningen; B. van der Vegt, MD, PhD, Department of Pathology and Medical Biology, University of Groningen, University Medical Center Groningen; S. Arends, PhD, departments of Rheumatology and Clinical Immunology, University of Groningen, University Medical Center Groningen; A. Vissink, DMD, MD, PhD, Oral and Maxillofacial Surgery, University of Groningen, University Medical Center Groningen; H. Bootsma, MD, PhD, departments of Rheumatology and Clinical Immunology, University of Groningen, University Medical Center Groningen; W.H. Abdulahad, PhD, departments of Rheumatology and Clinical Immunology, University of Groningen, University Medical Center Groningen.

Bootsma, Hendrika. From the departments of Rheumatology and Clinical Immunology, Oral and Maxillofacial Surgery, and Pathology and Medical Biology, University of Groningen, University Medical Center Groningen, Groningen; the Department of Pulmonary Medicine, Erasmus MC, Rotterdam, the Netherlands.

Bootsma, Hendrika. G.M. Verstappen, MSc, departments of Rheumatology and Clinical Immunology, University of Groningen, University Medical Center Groningen; F.G. Kroese, PhD, departments of Rheumatology and Clinical Immunology, University of Groningen, University Medical Center Groningen; P.M. Meiners, MD, PhD, DMD, Oral and Maxillofacial Surgery, University of Groningen, University Medical Center Groningen; O.B. Corneth, PhD, Department of Pulmonary Medicine, Erasmus MC; M.G. Huitema, BSc, departments of Rheumatology and Clinical Immunology, University of Groningen, University Medical Center Groningen; E.A. Haacke, MD, departments of Rheumatology and Clinical Immunology, and departments of Pathology and Medical Biology, University of Groningen, University Medical Center Groningen; B. van der Vegt, MD, PhD, Department of Pathology and Medical Biology, University of Groningen, University Medical Center Groningen; S. Arends, PhD, departments of Rheumatology and Clinical Immunology, University of Groningen, University Medical Center Groningen; A. Vissink, DMD,

MD, PhD, Oral and Maxillofacial Surgery, University of Groningen, University Medical Center Groningen; H. Bootsma, MD, PhD, departments of Rheumatology and Clinical Immunology, University of Groningen, University Medical Center Groningen; W.H. Abdulahad, PhD, departments of Rheumatology and Clinical Immunology, University of Groningen, University Medical Center Groningen.

Abdulahad, Wayel H. From the departments of Rheumatology and Clinical Immunology, Oral and Maxillofacial Surgery, and Pathology and Medical Biology, University of Groningen, University Medical Center Groningen, Groningen; the Department of Pulmonary Medicine, Erasmus MC, Rotterdam, the Netherlands.

Abdulahad, Wayel H. G.M. Verstappen, MSc, departments of Rheumatology and Clinical Immunology, University of Groningen, University Medical Center Groningen; F.G. Kroese, PhD, departments of Rheumatology and Clinical Immunology, University of Groningen, University Medical Center Groningen; P.M. Meiners, MD, PhD, DMD, Oral and Maxillofacial Surgery, University of Groningen, University Medical Center Groningen; O.B. Corneth, PhD, Department of Pulmonary Medicine, Erasmus MC; M.G. Huitema, BSc, departments of Rheumatology and Clinical Immunology, University of Groningen, University Medical Center Groningen; E.A. Haacke, MD, departments of Rheumatology and Clinical Immunology, and departments of Pathology and Medical Biology, University of Groningen, University Medical Center Groningen; B. van der Vegt, MD, PhD, Department of Pathology and Medical Biology, University of Groningen, University Medical Center Groningen; S. Arends, PhD, departments of Rheumatology and Clinical Immunology, University of Groningen, University Medical Center Groningen; A. Vissink, DMD, MD, PhD, Oral and Maxillofacial Surgery, University of Groningen, University Medical Center Groningen; H. Bootsma, MD, PhD, departments of Rheumatology and Clinical Immunology, University of Groningen, University Medical Center Groningen; W.H. Abdulahad, PhD, departments of Rheumatology and Clinical Immunology, University of Groningen, University Medical Center Groningen.

Abstract

OBJECTIVE: To assess the effect of B cell depletion therapy on effector CD4+ T cell homeostasis and its relation to objective measures of disease activity in patients with primary Sjogren syndrome (pSS).

METHODS: Twenty-four patients with pSS treated with rituximab (RTX) and 24 healthy controls (HC) were included. Frequencies of circulating effector CD4+ T cell subsets were examined by flow cytometry at baseline and 16, 24, 36, and 48 weeks after the first RTX infusion. Th1, Th2, follicular Th (TFH), and Th17 cells were discerned based on surface marker expression patterns. Additionally, intracellular cytokine staining was performed for interferon-gamma, interleukin (IL)-4, IL-21, and IL-17 and serum levels of these cytokines were analyzed.

RESULTS: In patients with pSS, frequencies of circulating TFH cells and Th17 cells were increased at baseline compared with HC, whereas frequencies of Th1 and Th2 cells were unchanged. B cell depletion therapy resulted in a pronounced decrease in circulating TFH cells, whereas Th17 cells were only slightly lowered. Frequencies of IL-21-producing and IL-17-producing CD4+ T cells and serum levels of IL-21 and IL-17 were also reduced. Importantly, the decrease in circulating TFH cells was associated with lower systemic disease activity over time, as measured by the European League Against Rheumatism Sjogren's Syndrome Disease Activity Index scores and serum IgG levels.

CONCLUSION: B cell depletion therapy in patients with pSS results in normalization of the elevated levels of circulating TFH cells. This reduction is associated with improved objective clinical disease activity measures. Our observations illustrate the pivotal role of the crosstalk between B cells and TFH cells in the pathogenesis of pSS.

Publication Type

Journal Article. Research Support, Non-U.S. Gov't.

Year of Publication

2017

<199>

Unique Identifier

28042124

Title

Improvement in Herpes Zoster Vaccination in Patients with Rheumatoid Arthritis: A Quality Improvement Project.

Source

Journal of Rheumatology. 44(1):11-17, 2017 01.

VI 1

Status

MEDLINE

Authors

Sheth H; Moreland L; Peterson H; Aggarwal R.

Authors Full Name

Sheth, Heena; Moreland, Larry; Peterson, Hilary; Aggarwal, Rohit.
Institution
Sheth, Heena. From the Department of Medicine, University of Pittsburgh School of Medicine, and Rheumatology Division, Department of Medicine, University of Pittsburgh Medical Center, Pittsburgh, Pennsylvania, USA.
Sheth, Heena. H. Sheth, MD, MPH, Department of Medicine, University of Pittsburgh School of Medicine; L. Moreland, MD, Rheumatology Division, Department of Medicine, University of Pittsburgh Medical Center; H. Peterson, BA, Rheumatology Division, Department of Medicine, University of Pittsburgh Medical Center; R. Aggarwal, MD, MS, Rheumatology Division, Department of Medicine, University of Pittsburgh Medical Center.
Moreland, Larry. From the Department of Medicine, University of Pittsburgh School of Medicine, and Rheumatology Division, Department of Medicine, University of Pittsburgh Medical Center, Pittsburgh, Pennsylvania, USA.
Moreland, Larry. H. Sheth, MD, MPH, Department of Medicine, University of Pittsburgh School of Medicine; L. Moreland, MD, Rheumatology Division, Department of Medicine, University of Pittsburgh Medical Center; H. Peterson, BA, Rheumatology Division, Department of Medicine, University of Pittsburgh Medical Center; R. Aggarwal, MD, MS, Rheumatology Division, Department of Medicine, University of Pittsburgh Medical Center.
Peterson, Hilary. From the Department of Medicine, University of Pittsburgh School of Medicine, and Rheumatology Division, Department of Medicine, University of Pittsburgh Medical Center, Pittsburgh, Pennsylvania, USA.
Peterson, Hilary. H. Sheth, MD, MPH, Department of Medicine, University of Pittsburgh School of Medicine; L. Moreland, MD, Rheumatology Division, Department of Medicine, University of Pittsburgh Medical Center; H. Peterson, BA, Rheumatology Division, Department of Medicine, University of Pittsburgh Medical Center; R. Aggarwal, MD, MS, Rheumatology Division, Department of Medicine, University of Pittsburgh Medical Center.
Aggarwal, Rohit. From the Department of Medicine, University of Pittsburgh School of Medicine, and Rheumatology Division, Department of Medicine, University of Pittsburgh Medical Center, Pittsburgh, Pennsylvania, USA. aggarwalr@upmc.edu.
Aggarwal, Rohit. H. Sheth, MD, MPH, Department of Medicine, University of Pittsburgh School of Medicine; L. Moreland, MD, Rheumatology Division, Department of Medicine, University of Pittsburgh Medical Center; H. Peterson, BA, Rheumatology Division, Department of Medicine, University of Pittsburgh Medical Center; R. Aggarwal, MD, MS, Rheumatology Division, Department of Medicine, University of Pittsburgh Medical Center. aggarwalr@upmc.edu.
Comments
Comment in (CIN)
Comment in (CIN)

Abstract
OBJECTIVE: To improve herpes zoster (HZ) vaccination rates in high-risk patients with rheumatoid arthritis (RA) being treated with immunosuppressive therapy.

METHODS: This quality improvement project was based on the pre- and post-intervention design. The project targeted all patients with RA over the age of 60 years while being treated with immunosuppressive therapy (not with biologics) seen in 13 rheumatology outpatient clinics. The study period was from July 2012 to June 2013 for the pre-intervention and February 2014 to January 2015 for the post-intervention phase. The electronic best practice alert (BPA) for HZ vaccination was developed; it appeared on electronic medical records during registration and medication reconciliation of the eligible patient by the medical assistant. The BPA was designed to electronically identify patient eligibility and to enable the physician to order the vaccine or to document refusal or deferral reason. Education regarding vaccine guidelines, BPA, vaccination process, and feedback were crucial components of the project interventions. The vaccination rates were compared using the chi-square test.

RESULTS: We evaluated 1823 and 1554 eligible patients with RA during the pre-intervention and post-intervention phases, respectively. The HZ vaccination rates, reported as patients vaccinated among all eligible patients, improved significantly from the pre-intervention period of 10.1% (184/1823) to 51.7% (804/1554) during the intervention phase ($p < 0.0001$). The documentation rates (vaccine received, vaccine ordered, patient refusal, and deferral reasons) increased from 28% (510/1823) to 72.9% (1133/1554; $p < 0.0001$). The HZ infection rates decreased significantly from 2% to 0.3% ($p = 0.002$).

CONCLUSION: Electronic identification of vaccine eligibility and BPA significantly improved HZ vaccination rates. The process required minimal modification of clinic work flow and did not burden the physician's time, and has the potential for self-sustainability and generalizability.
Publication Type
Journal Article. Research Support, Non-U.S. Gov't.
Year of Publication
2017

<200>

Unique Identifier

27998041

Title

Rheumatic and Musculoskeletal Immune-Related Adverse Events Due to Immune Checkpoint Inhibitors: A Systematic Review of the Literature. [**REVIEW**]

Source

Arthritis care & research. 69(11):1751-1763, 2017 11.

VI 1

Status

MEDLINE

Authors

Cappelli LC; Gutierrez AK; Bingham CO 3rd; Shah AA.

Authors Full Name

Cappelli, Laura C; Gutierrez, Anna Kristina; Bingham, Clifton O 3rd; Shah, Ami A.

Institution

Cappelli, Laura C. Johns Hopkins University, Baltimore, Maryland.

Gutierrez, Anna Kristina. Johns Hopkins University, Baltimore, Maryland.

Bingham, Clifton O 3rd. Johns Hopkins University, Baltimore, Maryland.

Shah, Ami A. Johns Hopkins University, Baltimore, Maryland.

Comments

Comment in (CIN)

Abstract

OBJECTIVE: Immune checkpoint inhibitors (ICIs) are improving prognoses in advanced stage cancers, but they also lead to immune-related adverse events (IRAEs). IRAEs targeting many organ systems have been reported, but musculoskeletal and rheumatic IRAEs have not been well-characterized. We systematically reviewed published literature on musculoskeletal and rheumatic IRAEs to better understand prevalence and clinical characteristics.

METHODS: Medline and CENTRAL databases were searched for articles reporting rheumatic and musculoskeletal IRAEs secondary to ICI treatment. After screening abstracts and full texts in duplicate, clinical features, prevalence, and treatment data were extracted and summarized.

RESULTS: A total of 1,725 unique abstracts were screened; 231 contained original data and were about ICIs and went to full-text screening. Fifty-two of these contained information about musculoskeletal or rheumatic IRAEs or about treatment with ICIs in preexisting autoimmune disease. Of these, 33 were clinical trials, 3 were observational studies, and 16 were case reports or series. Arthralgia prevalence in clinical trials ranged 1-43%, and myalgia was reported in 2-

20%. Arthritis was reported in 5 of 33 clinical trials, and vasculitis was reported in only 2. One observational study and 3 case reports described patients with preexisting autoimmune disease treated with ICIs. Case reports included development of inflammatory arthritis, vasculitis, myositis, and lupus nephritis.

CONCLUSION: Arthralgia and myalgia have been reported commonly in patients treated with ICIs. The prevalence of rheumatic IRAEs such as inflammatory arthritis, vasculitis, and sicca syndrome is less clear from current evidence. There is limited observational and case-level evidence describing ICI use in patients with preexisting autoimmune disease. Copyright © 2016, American College of Rheumatology.

Publication Type

Journal Article. Review. Systematic Review.

Year of Publication

2017

<201>

Unique Identifier

27890174

Title

Immune-Related Adverse Effects of Cancer Immunotherapy- Implications for Rheumatology. [Review]

Source

Rheumatic Diseases Clinics of North America. 43(1):65-78, 2017 02.

VI 1

Status

MEDLINE

Authors

Cappelli LC; Shah AA; Bingham CO 3rd.

Authors Full Name

Cappelli, Laura C; Shah, Ami A; Bingham, Clifton O 3rd.

Institution

Cappelli, Laura C. Division of Rheumatology, Johns Hopkins School of Medicine, 5501 Hopkins Bayview Circle, Suite 1.B1, Baltimore, MD, USA. Electronic address: lcappel1@jhmi.edu.

Shah, Ami A. Division of Rheumatology, Johns Hopkins School of Medicine, 5501 Hopkins Bayview Circle, Suite 1.B1, Baltimore, MD, USA.
Bingham, Clifton O 3rd. Division of Rheumatology, Johns Hopkins School of Medicine, 5501 Hopkins Bayview Circle, Suite 1.B1, Baltimore, MD, USA.
Abstract
Immune checkpoint inhibitors (ICIs) are increasingly studied and used as therapy for a growing number of malignancies. ICIs work by blocking inhibitory pathways of T-cell activation, leading to an immune response directed against tumors. Such nonspecific immunologic activation can lead to immune-related adverse events (IRAEs). Some IRAEs, including inflammatory arthritis, sicca syndrome, myositis, and vasculitis, are of special interest to rheumatologists. As use of ICIs increases, recognition of these IRAEs and developing treatment strategies will become important. In this review, the current literature on rheumatic and musculoskeletal IRAEs is summarized. The incidence, clinical presentations, and treatment considerations are highlighted. Copyright A© 2016 Elsevier Inc. All rights reserved.
Publication Type
Journal Article. Review. Research Support, Non-U.S. Gov't.
Year of Publication
2017

<202>
Unique Identifier
27878345
Title
Low vaccination rates among patients with rheumatoid arthritis in a German outpatient clinic.
Source
Rheumatology International. 37(2):229-237, 2017 Feb.
VI 1
Status
MEDLINE
Authors
Krasselt M; Ivanov JP; Baerwald C; Seifert O.
Author NameID
Krasselt, Marco; ORCID: http://orcid.org/0000-0002-1842-0754

Authors Full Name
Krasselt, Marco; Ivanov, Jean-Philipp; Baerwald, Christoph; Seifert, Olga.
Institution
Krasselt, Marco. Rheumatology Unit, Clinic for Gastroenterology and Rheumatology, Department of Internal Medicine, Neurology and Dermatology, University of Leipzig, Liebigstr. 20, 04103, Leipzig, Germany. marco.krasselt@medizin.uni-leipzig.de.
Ivanov, Jean-Philipp. Rheumatology Unit, Clinic for Gastroenterology and Rheumatology, Department of Internal Medicine, Neurology and Dermatology, University of Leipzig, Liebigstr. 20, 04103, Leipzig, Germany.
Baerwald, Christoph. Rheumatology Unit, Clinic for Gastroenterology and Rheumatology, Department of Internal Medicine, Neurology and Dermatology, University of Leipzig, Liebigstr. 20, 04103, Leipzig, Germany.
Seifert, Olga. Rheumatology Unit, Clinic for Gastroenterology and Rheumatology, Department of Internal Medicine, Neurology and Dermatology, University of Leipzig, Liebigstr. 20, 04103, Leipzig, Germany.
Abstract
Patients with rheumatoid arthritis (RA) are at an increased risk of acquiring infections due to two reasons: the disease itself and the immunosuppressive therapy. Vaccinations against preventable diseases are therefore of utmost importance for these group of patients. To estimate vaccination frequencies among patients with rheumatoid arthritis, we studied patients in a survey and calculated vaccination rates based on their vaccination documents. Patients have been recruited from our outpatient clinic during one of their routine visits. For the statistical analysis, they have been divided by age (>=60 vs <60 years) and medication (DMARD, Biologics, TNF inhibitors) for further subgroup analysis. Among the studied patients (n = 331), we found rather low vaccination rates, in particular for the strongly recommended vaccines against Pneumococcus and Influenza (33 and 53%, respectively). Furthermore, protection rates for important basic vaccinations, e.g. against Pertussis, were found to be very low with 12% only. Beside these findings, we saw age-dependent differences for a variety of vaccines: while Pneumococcus and Influenza vaccines were more often given to patients >=60 years, MMR, Pertussis, Diphtheria and Hepatitis were significantly more often applied to younger patients. Vaccination rates have to be improved among RA patients, in particular for vaccines protecting from respiratory tract infections such as Pneumococcus.
Publication Type
Journal Article.
Year of Publication
2017

<203>

Unique Identifier

27841031

Title

Improving B-cell depletion in systemic lupus erythematosus and rheumatoid arthritis. [Review]

Source

Expert Review of Clinical Immunology. 13(7):667-676, 2017 07.

VI 1

Status

MEDLINE

Authors

Mota P; Reddy V; Isenberg D.

Authors Full Name

Mota, Pedro; Reddy, Venkat; Isenberg, David.

Institution

Mota, Pedro. a Department of Internal Medicine, Hospital da Luz, Lisbon, Portugal.
Reddy, Venkat. b Centre for Rheumatology, Division of Medicine, University College London, London, UK.
Isenberg, David. b Centre for Rheumatology, Division of Medicine, University College London, London, UK.

Abstract

INTRODUCTION: Rituximab-based B-cell depletion (BCD) therapy is effective in refractory rheumatoid arthritis (RA) and although used to treat patients with refractory systemic lupus erythematosus (SLE) in routine clinical practice, rituximab failed to meet the primary endpoints in two large randomised controlled trials (RCTs) of non-renal (EXPLORER) and renal (LUNAR) SLE. Areas covered: We review how BCD could be improved to achieve better clinical responses in RA and SLE. Insights into the variability in clinical response to BCD in RA and SLE may help develop new therapeutic strategies. To this end, a literature search was performed using the following terms: rheumatoid arthritis, systemic erythematosus lupus, rituximab and B-cell depletion. Expert commentary: Poor trial design may have, at least partly, contributed to the apparent lack of response to BCD in the two RCTs of patients with SLE. Enhanced B-cell depletion and/or sequential therapy with belimumab may improve clinical response at least in some patients with SLE.

Publication Type
  Journal Article. Review. Research Support, Non-U.S. Gov't.
Year of Publication
  2017

<204>
Unique Identifier
  27687304
Title
  Anti-PD-1 therapy in patients with advanced melanoma and preexisting autoimmune disorders or major toxicity with ipilimumab. Annals of Oncology. 28(2):368-376, 2017 02 01.

Authors
  Menzies AM; Johnson DB; Ramanujam S; Atkinson VG; Wong ANM; Park JJ; McQuade JL; Shoushtari AN; Tsai KK; Eroglu Z; Klein O; Hassel JC; Sosman JA; Guminski A; Sullivan RJ; Ribas A; Carlino MS; Davies MA; Sandhu SK; Long GV.
Authors Full Name
  Menzies, A M; Johnson, D B; Ramanujam, S; Atkinson, V G; Wong, A N M; Park, J J; McQuade, J L; Shoushtari, A N; Tsai, K K; Eroglu, Z; Klein, O; Hassel, J C; Sosman, J A; Guminski, A; Sullivan, R J; Ribas, A; Carlino, M S; Davies, M A; Sandhu, S K; Long, G V.
Institution
  Menzies, A M. Melanoma Institute Australia and The University of Sydney, Sydney, Australia.
  Menzies, A M. Royal North Shore and Mater Hospitals, Sydney, Australia.
  Johnson, D B. Vanderbilt University Medical Center, Nashville, USA.
  Ramanujam, S. Melanoma Institute Australia and The University of Sydney, Sydney, Australia.
  Atkinson, V G. Princess Alexandra Hospital, Greenslopes Hospital and University of Queensland, Brisbane, Australia.
  Wong, A N M. Peter MacCallum Cancer Centre, Melbourne, Australia.
  Park, J J. Crown Princess Mary Cancer Centre Westmead, Sydney, Australia.
  McQuade, J L. The University of Texas MD Anderson Cancer Center, Houston, USA.
  Shoushtari, A N. Memorial Sloan Kettering Cancer Center, New York, USA.
  Tsai, K K. Department of Medical Oncology, University of California San Francisco, San Francisco, USA.

Eroglu, Z. Department of Medical Oncology, Moffitt Cancer Centre, Tampa, USA.
Klein, O. Department of Medical Oncology, Olivia Newton-John Cancer Centre & Cancer Research Institute, Austin Health, Melbourne, Australia.
Hassel, J C. Department of Dermatology, Heidelberg University, Heidelberg, Germany.
Sosman, J A. Vanderbilt University Medical Center, Nashville, USA.
Guminski, A. Melanoma Institute Australia and The University of Sydney, Sydney, Australia.
Guminski, A. Royal North Shore and Mater Hospitals, Sydney, Australia.
Sullivan, R J. Massachusetts General Hospital Cancer Center, Boston, USA.
Ribas, A. Division of Hematology-Oncology, University of California Los Angeles, Los Angeles, USA.
Carlino, M S. Melanoma Institute Australia and The University of Sydney, Sydney, Australia.
Carlino, M S. Crown Princess Mary Cancer Centre Westmead, Sydney, Australia.
Davies, M A. The University of Texas MD Anderson Cancer Center, Houston, USA.
Sandhu, S K. Peter MacCallum Cancer Centre, Melbourne, Australia.
Long, G V. Melanoma Institute Australia and The University of Sydney, Sydney, Australia.
Long, G V. Royal North Shore and Mater Hospitals, Sydney, Australia.

Comments

Comment in (CIN)

Abstract

Background: Anti-PD-1 antibodies (anti-PD-1) have clinical activity in a number of malignancies. All clinical trials have excluded patients with significant preexisting autoimmune disorders (ADs) and only one has included patients with immune-related adverse events (irAEs) with ipilimumab. We sought to explore the safety and efficacy of anti-PD-1 in such patients.

Patients and methods: Patients with advanced melanoma and preexisting ADs and/or major immune-related adverse events (irAEs) with ipilimumab (requiring systemic immunosuppression) that were treated with anti-PD-1 between 1 July 2012 and 30 September 2015 were retrospectively identified.

Results: One hundred and nineteen patients from 13 academic tertiary referral centers were treated with anti-PD-1. In patients with preexisting AD (N = 52), the response rate was 33%. 20 (38%) patients had a flare of AD requiring immunosuppression, including 7/13 with rheumatoid arthritis, 3/3 with polymyalgia rheumatica, 2/2 with Sjogren's syndrome, 2/2 with immune thrombocytopaenic purpura and 3/8 with psoriasis. No patients with gastrointestinal (N = 6) or neurological disorders (N = 5) flared. Only 2 (4%) patients discontinued treatment due to flare, but 15 (29%) developed other irAEs and 4 (8%) discontinued treatment. In patients with prior ipilimumab irAEs requiring immunosuppression (N = 67) the response rate was 40%. Two (3%)

patients had a recurrence of the same ipilimumab irAEs, but 23 (34%) developed new irAEs (14, 21% grade 3-4) and 8 (12%) discontinued treatment. There were no treatment-related deaths.

Conclusions: In melanoma patients with preexisting ADs or major irAEs with ipilimumab, anti-PD-1 induced relatively frequent immune toxicities, but these were often mild, easily managed and did not necessitate discontinuation of therapy, and a significant proportion of patients achieved clinical responses. The results support that anti-PD-1 can be administered safely and can achieve clinical benefit in patients with preexisting ADs or prior major irAEs with ipilimumab.
Copyright © The Author 2016. Published by Oxford University Press on behalf of the European Society for Medical Oncology. All rights reserved. For permissions, please email: journals.permissions@oup.com.
Publication Type
Journal Article.
Year of Publication
2017

<205>
Unique Identifier
27667787
Title
Tolerogenic dendritic cells generated with dexamethasone and vitamin D3 regulate rheumatoid arthritis CD4+ T cells partly via transforming growth factor-beta1.
Source
Clinical & Experimental Immunology. 187(1):113-123, 2017 Jan.
VI 1
Status
MEDLINE
Authors
Anderson AE; Swan DJ; Wong OY; Buck M; Eltherington O; Harry RA; Patterson AM; Pratt AG; Reynolds G; Doran JP; Kirby JA; Isaacs JD; Hilkens CM.
Authors Full Name
Anderson, A E; Swan, D J; Wong, O Y; Buck, M; Eltherington, O; Harry, R A; Patterson, A M; Pratt, A G; Reynolds, G; Doran, J-P; Kirby, J A; Isaacs, J D; Hilkens, C M U.

Institution
Anderson, A E. Musculoskeletal Research Group.
Anderson, A E. Arthritis Research UK Rheumatoid Arthritis Centre of Excellence (RACE).
Swan, D J. Musculoskeletal Research Group.
Wong, O Y. Musculoskeletal Research Group.
Buck, M. Musculoskeletal Research Group.
Eltherington, O. Musculoskeletal Research Group.
Eltherington, O. Arthritis Research UK Rheumatoid Arthritis Centre of Excellence (RACE).
Harry, R A. Musculoskeletal Research Group.
Harry, R A. Arthritis Research UK Rheumatoid Arthritis Centre of Excellence (RACE).
Patterson, A M. Musculoskeletal Research Group.
Pratt, A G. Musculoskeletal Research Group.
Pratt, A G. Arthritis Research UK Rheumatoid Arthritis Centre of Excellence (RACE).
Reynolds, G. Musculoskeletal Research Group.
Reynolds, G. Arthritis Research UK Rheumatoid Arthritis Centre of Excellence (RACE).
Doran, J-P. Musculoskeletal Research Group.
Kirby, J A. Applied Immunobiology and Transplantation Research Group, Institute of Cellular Medicine at the Newcastle NIHR Biomedical Research Centre, Newcastle University and Newcastle upon Tyne NHS Trust, Newcastle upon Tyne, UK.
Isaacs, J D. Musculoskeletal Research Group.
Isaacs, J D. Arthritis Research UK Rheumatoid Arthritis Centre of Excellence (RACE).
Hilkens, C M U. Musculoskeletal Research Group.
Hilkens, C M U. Arthritis Research UK Rheumatoid Arthritis Centre of Excellence (RACE).
Abstract
Tolerogenic dendritic cells (tolDC) are a new immunotherapeutic tool for the treatment of rheumatoid arthritis (RA) and other autoimmune disorders. We have established a method to generate stable tolDC by pharmacological modulation of human monocyte-derived DC. These tolDC exert potent pro-tolerogenic actions on CD4+ T cells. Lack of interleukin (IL)-12p70 production is a key immunoregulatory attribute of tolDC but does not explain their action fully. Here we show that tolDC express transforming growth factor (TGF)-beta1 at both mRNA and protein levels, and that expression of this immunoregulatory cytokine is significantly higher in tolDC than in mature monocyte-derived DC. By inhibiting TGF-beta1 signalling we demonstrate that tolDC regulate CD4+ T cell responses in a manner that is at least partly dependent upon this cytokine. Crucially, we also show that while there is no significant difference in expression of TGF-betaRII on CD4+ T cells from RA patients and healthy controls, RA patient CD4+ T cells are measurably less responsive to TGF-beta1 than healthy control CD4+ T cells [reduced TGF-beta-induced mothers against decapentaplegic homologue (Smad)2/3 phosphorylation, forkhead box

protein 3 (FoxP3) expression and suppression of (IFN)-gamma secretion]. However, CD4+ T cells from RA patients can, nonetheless, be regulated efficiently by tolDC in a TGF-beta1-dependent manner. This work is important for the design and development of future studies investigating the potential use of tolDC as a novel immunotherapy for the treatment of RA.
Copyright © 2016 British Society for Immunology.
Publication Type
Journal Article.
Year of Publication
2017

<206>
Unique Identifier
27643385
Title
RORgammat antagonist suppresses M3 muscarinic acetylcholine receptor-induced Sjogren's syndrome-like sialadenitis.
Source
Clinical & Experimental Immunology. 187(2):213-224, 2017 Feb.
VI 1
Status
MEDLINE
Authors
Tahara M; Tsuboi H; Segawa S; Asashima H; Iizuka-Koga M; Hirota T; Takahashi H; Kondo Y; Matsui M; Matsumoto I; Sumida T.
Authors Full Name
Tahara, M; Tsuboi, H; Segawa, S; Asashima, H; Iizuka-Koga, M; Hirota, T; Takahashi, H; Kondo, Y; Matsui, M; Matsumoto, I; Sumida, T.
Institution
Tahara, M. Department of Internal Medicine, Faculty of Medicine, University of Tsukuba, Tsukuba, Ibaraki.
Tsuboi, H. Department of Internal Medicine, Faculty of Medicine, University of Tsukuba, Tsukuba, Ibaraki.

Segawa, S. Department of Internal Medicine, Faculty of Medicine, University of Tsukuba, Tsukuba, Ibaraki.
Asashima, H. Department of Internal Medicine, Faculty of Medicine, University of Tsukuba, Tsukuba, Ibaraki.
Iizuka-Koga, M. Department of Internal Medicine, Faculty of Medicine, University of Tsukuba, Tsukuba, Ibaraki.
Hirota, T. Department of Internal Medicine, Faculty of Medicine, University of Tsukuba, Tsukuba, Ibaraki.
Takahashi, H. Department of Internal Medicine, Faculty of Medicine, University of Tsukuba, Tsukuba, Ibaraki.
Kondo, Y. Department of Internal Medicine, Faculty of Medicine, University of Tsukuba, Tsukuba, Ibaraki.
Matsui, M. Department of Internal Medicine, Fureai Higashitotsuka Hospital, Yokohama, Kanagawa, Japan.
Matsumoto, I. Department of Internal Medicine, Faculty of Medicine, University of Tsukuba, Tsukuba, Ibaraki.
Sumida, T. Department of Internal Medicine, Faculty of Medicine, University of Tsukuba, Tsukuba, Ibaraki.
Abstract
We showed recently that M3 muscarinic acetylcholine receptor (M3R)-reactive CD3+ T cells play a pathogenic role in the development of murine autoimmune sialadenitis (MIS), which mimics Sjogren's syndrome (SS). The aim of this study was to determine the effectiveness and mechanism of action of retinoic acid-related orphan receptor-gamma t (RORgammat) antagonist (A213) in MIS. Splenocytes from M3R knockout (M3R-/-) mice immunized with murine M3R peptide mixture were inoculated into recombination-activating gene 1 knockout (Rag-1-/-) mice (M3R-/- ->Rag-1-/-) with MIS. Immunized M3R-/- mice (pretransfer treatment) and M3R-/- ->Rag-1-/- mice (post-transfer treatment) were treated with A213 every 3 days. Salivary volume, severity of sialadenitis and cytokine production from M3R peptide-stimulated splenocytes and lymph node cells were examined. Effects of A213 on cytokine production were analysed by enzyme-linked immunosorbent assay (ELISA) and on T helper type 1 (Th1), Th17 and Th2 differentiation from CD4+ T cells by flow cytometry. Pretransfer A213 treatment maintained salivary volume, improved MIS and reduced interferon (IFN)-gamma and interleukin (IL)-17 production significantly compared with phosphate-buffered saline (PBS) ($P < 0.05$). These suppressive effects involved CD4+ T cells rather than CD11c+ cells. Post-transfer treatment with A213 increased salivary volume ($P < 0.05$), suppressed MIS ($P < 0.005$) and reduced IFN-gamma and IL-17 production ($P < 0.05$). In vitro, A213 suppressed IFN-gamma and IL-17 production from M3R-stimulated splenocytes and CD4+ T cells of immunized M3R-/- mice ($P < 0.05$). In contrast with M3R specific

responses, A213 suppressed only IL-17 production from Th17 differentiated CD4+ T cells without any effect on Th1 and Th2 differentiation in vitro. Our findings suggested that RORgammat antagonism is potentially suitable treatment strategy for SS-like sialadenitis through suppression of IL-17 and IFN-gamma production by M3R-specific T cells. Copyright © 2016 British Society for Immunology.

Publication Type

Journal Article.

Year of Publication

2017

<207>

Unique Identifier

27566797

Title

Checkpoint immunotherapy: good for cancer therapy, bad for rheumatic diseases.

Source

Annals of the Rheumatic Diseases. 76(1):1-3, 2017 01.

REVIEW

Authors

Calabrese L; Velcheti V.

Authors Full Name

Calabrese, Leonard; Velcheti, Vamsidhar.

Institution

Calabrese, Leonard. Department of Rheumatology/Immunology, Cleveland Clinic, Cleveland, Ohio, USA.

Velcheti, Vamsidhar. Department of Hematology and Oncology, Cleveland Clinic, Cleveland, Ohio, USA.

Comments

Comment on (CON)

Publication Type

Editorial. Comment.

Year of Publication

2017

<208>

Unique Identifier

27550301

Title

Monotherapy with biologic disease-modifying anti-rheumatic drugs in rheumatoid arthritis. [Review]

Source

Rheumatology. 56(5):689-697, 2017 05 01.

VI 1

Status

MEDLINE

Authors

Choy E; Aletaha D; Behrens F; Finckh A; Gomez-Reino J; Gottenberg JE; Schuch F; Rubbert-Roth A.

Authors Full Name

Choy, Ernest; Aletaha, Daniel; Behrens, Frank; Finckh, Axel; Gomez-Reino, Juan; Gottenberg, Jacques-Eric; Schuch, Florian; Rubbert-Roth, Andrea.

Institution

Choy, Ernest. CREATE Centre, Institute of Infection & Immunity, Cardiff University, Cardiff, UK.

Aletaha, Daniel. Department of Rheumatology, Medical University Vienna, Vienna, Austria.

Behrens, Frank. CIRI/Division of Rheumatology, Johann Wolfgang Goethe-University, Frankfurt, Germany.

Finckh, Axel. Division of Rheumatology, University Hospital of Geneva, Geneva, Switzerland.

Gomez-Reino, Juan. Hospital Clinico, University of Santiago de Compostela, Santiago de Compostela, Spain.

Gottenberg, Jacques-Eric. Department of Rheumatology, Strasbourg University Hospitals, Strasbourg, France.

Schuch, Florian. Rheumatologic Community Practice, Erlangen.

Rubbert-Roth, Andrea. Department of Internal Medicine, University of Cologne, Cologne, Germany.

Abstract

Current EULAR guidelines state that biologic DMARD (bDMARD) therapy should be administered in combination with MTX or other conventional synthetic (cs) DMARD in RA. Nonetheless, a third of patients for whom a bDMARD agent is prescribed take it in the absence of concurrent csDMARD therapy. While the reasons underlying the low uptake of bDMARD-csDMARD combination therapy in clinical practice have not been well delineated, they may include poor adherence, contraindication to csDMARD therapy and adverse effects, as well as csDMARD withdrawal following remission. The challenges surrounding bDMARD therapy and the benefit/risk ratio of biologic monotherapy when compared with combination with a csDMARD will be discussed. We will provide insights into these important issues, as well as reviewing the evidence base differentiating biologic agents and exploring therapeutic options for patients with rheumatoid arthritis for whom csDMARD therapy is contraindicated or discontinued. Copyright © The Author 2016. Published by Oxford University Press on behalf of the British Society for Rheumatology. All rights reserved. For Permissions, please email: journals.permissions@oup.com.

Publication Type

Journal Article. Review. Research Support, Non-U.S. Gov't.

Year of Publication

2017

<209>

Unique Identifier

27342458

Title

Cardiac Impairment in Rheumatoid Arthritis and Influence of Anti-TNFalpha Treatment. [Review]

Source

Clinical Reviews in Allergy & Immunology. 52(3):323-332, 2017 Jun.

VI 1

Status

MEDLINE

Authors

Lazurova I; Tomas L.

Author NameID

Tomas, Lubomir; ORCID: http://orcid.org/0000-0002-3774-2820

Authors Full Name
Lazurova, Ivica; Tomas, Lubomir.
Institution
Lazurova, Ivica. 1st Department od Internal Medicine, Faculty of Medicine, Pavol Jozef Safarik University in Kosice, Trieda SNP 1, 040 11, Kosice, Slovakia. lubomir.tomas@upjs.sk.
Tomas, Lubomir. 1st Department od Internal Medicine, Faculty of Medicine, Pavol Jozef Safarik University in Kosice, Trieda SNP 1, 040 11, Kosice, Slovakia.
Abstract
There is evidence that rheumatoid arthritis (RA) is associated with higher overall and cardiovascular (CV) morbidity and mortality as compared with general population. Increased prevalence of traditional risk factors and chronic inflammation, that has been recognized as independent CV risk factor, may play an important role in atherosclerosis and subsequently ischemic heart disease development. However, myocardial dysfunction as a result of chronic inflammation and secondarily myocardial fibrosis markedly participates on heart failure development. Proinflammatory cytokines, such as C-reactive protein, tumor necrosis factor alpha (TNFalpha), interleukins 1 and 6, that are markedly increased in RA, play a role in the acceleration of atherosclerosis as well as myocardial fibrosis development. Several studies documented that increased CV risk was associated with seropositivity, disease activity score, citrullination, and duration of RA. Early detection of heart dysfunction is based on echocardiographic detection of diastolic dysfunction resulting from myocardial inflammation and fibrosis. Some studies showed also higher prevalence of left ventricular systolic dysfunction and increased prevalence of cardiac arrhythmias as compared to non-RA population. There are still controversies on the impact of NT-proBNP in predicting cardiac impairment in RA patients. Some authors consider it to be a sensitive noninvasive predictor of subclinical CV disease in these patients and also a predictor of all-cause mortality independently on traditional CV risk factors. However, the correlation with parameters of cardiac function was confirmed only in a few studies. The impact of biological treatment on progression of atherosclerosis and heart failure is still controversial and seems to be not harmful in young patients with normal left ventricular function. The effect of biologics, especially anti-TNFalpha drugs, is probably related to the cardiac function before treatment. Larger prospective clinical, echocardiographic, and magnetic resonance studies are needed.
Publication Type
Journal Article. Review.
Year of Publication
2017

<210>

Unique Identifier

27307501

Title

Inflammatory arthritis and sicca syndrome induced by nivolumab and ipilimumab.

Source

Annals of the Rheumatic Diseases. 76(1):43-50, 2017 Jan.

VI 1

Status

MEDLINE

Authors

Cappelli LC; Gutierrez AK; Baer AN; Albayda J; Manno RL; Haque U; Lipson EJ; Bleich KB; Shah AA; Naidoo J; Brahmer JR; Le D; Bingham CO 3rd.

Authors Full Name

Cappelli, Laura C; Gutierrez, Anna Kristina; Baer, Alan N; Albayda, Jemima; Manno, Rebecca L; Haque, Uzma; Lipson, Evan J; Bleich, Karen B; Shah, Ami A; Naidoo, Jarushka; Brahmer, Julie R; Le, Dung; Bingham, Clifton O 3rd.

Institution

Cappelli, Laura C. Division of Rheumatology, Department of Medicine, Johns Hopkins University, Baltimore, Maryland, USA.

Gutierrez, Anna Kristina. Division of Rheumatology, Department of Medicine, Johns Hopkins University, Baltimore, Maryland, USA.

Baer, Alan N. Division of Rheumatology, Department of Medicine, Johns Hopkins University, Baltimore, Maryland, USA.

Albayda, Jemima. Division of Rheumatology, Department of Medicine, Johns Hopkins University, Baltimore, Maryland, USA.

Manno, Rebecca L. Division of Rheumatology, Department of Medicine, Johns Hopkins University, Baltimore, Maryland, USA.

Haque, Uzma. Division of Rheumatology, Department of Medicine, Johns Hopkins University, Baltimore, Maryland, USA.

Lipson, Evan J. Department of Oncology, Johns Hopkins University, Baltimore, Maryland, USA.

Bleich, Karen B. Department of Radiology, Johns Hopkins University, Baltimore, Maryland, USA.

Shah, Ami A. Division of Rheumatology, Department of Medicine, Johns Hopkins University, Baltimore, Maryland, USA.
Naidoo, Jarushka. Department of Oncology, Johns Hopkins University, Baltimore, Maryland, USA.
Brahmer, Julie R. Department of Oncology, Johns Hopkins University, Baltimore, Maryland, USA.
Le, Dung. Department of Oncology, Johns Hopkins University, Baltimore, Maryland, USA.
Bingham, Clifton O 3rd. Division of Rheumatology, Department of Medicine, Johns Hopkins University, Baltimore, Maryland, USA.
Comments
Comment in (CIN)
Abstract
OBJECTIVES: Immune checkpoint inhibitors (ICIs) targeting the cytotoxic T-lymphocyte-associated protein 4 (CTLA-4) and programmed cell death protein 1 (PD-1) pathways have demonstrated survival improvements in multiple advanced cancers, but also cause immune-related adverse events (IRAEs). IRAEs with clinical features similar to rheumatic diseases have not been well described. We report patients with inflammatory arthritis and sicca syndrome secondary to ICIs.

METHODS: We report patients evaluated in the Johns Hopkins Rheumatology clinics from 2012 to 2016 identified as having new rheumatological symptoms in the context of treatment with ipilimumab (anti-CTLA-4) and/or nivolumab (anti-PD-1) for solid tumours.

RESULTS: We identified 13 patients who received ICIs and developed rheumatological IRAEs. Mean age was 58.7 years. Cancer types included melanoma, non-small cell lung cancer, small cell lung cancer and renal cell carcinoma. ICI regimens included nivolumab or ipilimumab as monotherapy (n=5), or combination nivolumab and ipilimumab (n=8). Nine of 13 patients developed an inflammatory arthritis, 4 with synovitis confirmed on imaging (3 ultrasound, 1 MRI) and 4 with inflammatory synovial fluid. Four patients developed sicca syndrome with severe salivary hypofunction. Other IRAEs included: pneumonitis, colitis, interstitial nephritis and thyroiditis. Antinuclear antibodies were positive in 5 out of 13 patients. All 13 patients were treated with corticosteroids with varying response. Two patients were treated with methotrexate and antitumor necrosis factor therapy for inflammatory arthritis.

CONCLUSIONS: As ICIs are increasingly used for a range of malignancies, new cases of rheumatic IRAEs are likely to emerge. Further research is required to understand mechanisms, determine risk factors and develop management algorithms for rheumatic IRAEs. Copyright

Published by the BMJ Publishing Group Limited. For permission to use (where not already granted under a licence) please go to http://www.bmj.com/company/products-services/rights-and-licensing/.
Publication Type
Journal Article.
Year of Publication
2017

<211>
Unique Identifier
27245419
Title
Herpes zoster: Risk and prevention during immunomodulating therapy. [Review]
Source
Joint, Bone, Spine: Revue du Rhumatisme. 84(1):21-27, 2017 Jan.
VI 1
Status
MEDLINE
Authors
Tran CT; Ducancelle A; Masson C; Lunel-Fabiani F.
Authors Full Name
Tran, Cong Tri; Ducancelle, Alexandra; Masson, Charles; Lunel-Fabiani, Francoise.
Institution
Tran, Cong Tri. UPRES EA 3859, laboratoire de virologie, CHU d'Angers, 4, rue Larrey, 49933 Angers cedex 9, France.
Ducancelle, Alexandra. UPRES EA 3859, laboratoire de virologie, CHU d'Angers, 4, rue Larrey, 49933 Angers cedex 9, France.
Masson, Charles. Service de rhumatologie, CHU Angers, 4, rue Larrey, 49933 Angers cedex 09, France. Electronic address: ChMasson@chu-angers.fr.
Lunel-Fabiani, Francoise. UPRES EA 3859, laboratoire de virologie, CHU d'Angers, 4, rue Larrey, 49933 Angers cedex 9, France.
Abstract

Herpes zoster can be serious or incapacitating, particularly in patients whose immune system is compromised by a disease or treatment. Immunomodulating drugs can increase the risk of infection. Well-established risk factors include advanced age and glucocorticoid therapy. The data are somewhat conflicting for medications such as methotrexate, tofacitinib, TNFalpha antagonists (infliximab, adalimumab, etanercept, certolizumab, and golimumab), abatacept, tocilizumab, and rituximab. Nevertheless, the risk of herpes zoster is increased in patients taking biological agents, because of the underlying diseases and/or effects of the drugs. A live attenuated herpes zoster vaccine has been proven effective and safe in immunocompetent individuals. At present, however, it is not recommended for patients with immunodeficiencies, including those taking biological drugs, as no studies have assessed its risk/benefit ratio in this population. This situation may change in the near future, as recent data support the effectiveness and safety of the herpes zoster vaccine in patients who take biotherapies or have other causes of immunodeficiency. Alternative approaches designed to protect these patients from herpes zoster and its complications are also under evaluation. There is a need to define the indications of the herpes zoster vaccine in terms of the target population, timing, modalities, and frequency, according to the underlying chronic systemic disease, age group, varicella-zoster virus status, and exposure to therapeutic agents. Copyright © 2016 Societe francaise de rhumatologie. Published by Elsevier SAS. All rights reserved.

Publication Type

Journal Article. Review.

Year of Publication

2017

<212>

Unique Identifier

27216429

Title

CD20-Mimotope Peptide Active Immunotherapy in Systemic Lupus Erythematosus and a Reappraisal of Vaccination Strategies in Rheumatic Diseases. [Review]

Source

Clinical Reviews in Allergy & Immunology. 52(2):217-233, 2017 Apr.

VI 1

Status

MEDLINE

Authors

Favoino E; Prete M; Marzullo A; Millo E; Shoenfeld Y; Perosa F.

Authors Full Name

Favoino, Elvira; Prete, Marcella; Marzullo, Andrea; Millo, Enrico; Shoenfeld, Yehuda; Perosa, Federico.

Institution

Favoino, Elvira. Department of Biomedical Sciences and Oncology (DIMO), Systemic Rheumatic and Autoimmune Diseases, University of Bari Medical School, Piazza G. Cesare 11, 70124, Bari, Italy.

Prete, Marcella. Department of Biomedical Sciences and Oncology (DIMO), Systemic Rheumatic and Autoimmune Diseases, University of Bari Medical School, Piazza G. Cesare 11, 70124, Bari, Italy.

Marzullo, Andrea. Department of Emergency and Organ Transplantation (DETO), Section of Anatomic Pathology, University of Bari Medical School, Piazza G. Cesare 11, 70124, Bari, Italy.

Millo, Enrico. Department of Experimental Medicine-Biochemistry section and Center of Excellence for Biomedical Research University of Genoa, Viale Benedetto XV 9, 16132, Genoa, Italy.

Shoenfeld, Yehuda. Zabludowicz Center for Autoimmune Diseases, Sheba Medical Center, Incumbent of the Laura Schwarz-Kipp Chair for Research of Autoimmune Diseases, Tel Aviv University, Tel Hashomer, 52621, Israel.

Perosa, Federico. Department of Biomedical Sciences and Oncology (DIMO), Systemic Rheumatic and Autoimmune Diseases, University of Bari Medical School, Piazza G. Cesare 11, 70124, Bari, Italy. federico.perosa@uniba.it.

Abstract

Systemic lupus erythematosus (SLE) is a chronic autoimmune disease in which any organs can be potential targets of autoimmune aggression. Although the pathogenic auto-antibodies have been well characterized, the role of B cells goes far beyond that of antibodies production, and B cell-targeted therapy may be an interesting therapeutic approach. The anti-CD20 monoclonal antibody rituximab has been successfully used to control the most severe form of SLE, and even if two controlled clinical trials failed to demonstrate its superiority compared to conventional immunosuppressants, off-label use of rituximab is still commonly adopted in clinical practice in SLE nephritis resistant to immunosuppressants. Different protocols have stipulated heterogeneous dosages but all of them included repeated injections of the drug, exposing the patient to the risk of adverse reactions and to tachyphylaxis (loss of the therapeutic effect). Stimulation of the host's immune system to develop a CD20 antigen-specific immune response by means of CD20-mimotope molecules may offer an approach that can overcome these

drawbacks. This study provides a critical overview of vaccination therapy in rheumatic diseases and reports the design of a vaccination strategy in (New Zealand Black/New Zealand White) F1 SLE-prone mice using CD20-mimotope peptides. By week 47, this vaccine induces a B- cell depletion by 74 % (cell number, mean +/- SD, 0.57 +/- 0.38) as compared to week 29 (2.19 +/- 0.55) (p = 0.005) and prolongs survival in peptide-treated mice (median, 46.71 weeks; 95 % CI, 39.78-53.64) as compared to the control group (median 39.85; 95 % CI, 37.41-42.30) (Kaplan-Meier p = 0.002), although no differences between the peptide group and control group were detected in terms of proteinuria and auto-antibodies titers. These data indicate the feasibility of this approach, and the mouse model described here may be useful to optimize vaccination protocol and to define the mechanism(s) underlying B- cell depletion.
Publication Type
  Journal Article. Review.
Year of Publication
  2017

<213>
Unique Identifier
  29429506
Title
  [What's new in internal medecine?]. [Review] [French]
Source
  Annales de Dermatologie et de Venereologie. 143 Suppl 3:S23-S28, 2016 Dec.
VI 1
Status
  MEDLINE
Authors
  Jachiet M; Rybojad M; Bouaziz JD.
Authors Full Name
  Jachiet, M; Rybojad, M; Bouaziz, J-D.
Institution
  Jachiet, M. Service de dermatologie, hopital Saint-Louis, INSERM U976, universite Paris-Diderot-Paris VII, Sorbonne-Paris-Cite, Paris, France.

Rybojad, M. Service de dermatologie, hopital Saint-Louis, INSERM U976, universite Paris-Diderot-Paris VII, Sorbonne-Paris-Cite, Paris, France.
Bouaziz, J-D. Service de dermatologie, hopital Saint-Louis, INSERM U976, universite Paris-Diderot-Paris VII, Sorbonne-Paris-Cite, Paris, France. Electronic address: jean-david.bouaziz@aphp.fr.
Abstract
Answering the question << what's new in internal medecine in 2016? >> is very challenging. We used 3 methods of article selection to reduce the selection bias: 3 authors, a systematic review of the articles discussed in the weekly bibliographic meeting of our unit (Dermatology department, Saint-Louis Hospital, Paris, France) and a selection of the best articles by several internal medecine practitioners in Paris. Eleven << hot topics >> were analyzed: i/lowering cholesterol level but not blood blessure has a significant impact on cardiovascular morbi-mortality in cardiovascular intermediate risk patients; ii/the << treat to treat target >> is efficient in psoriatic arthritis; iii/ a genotype/ phenotype correlation favors the separation of ileal Crohn's disease, colonic Crohn's disease and ulcerative colitis; iv/ tocilizumab treatment (anti-IL-6 monoclonal antibody) is very efficient in giant cell arteritis and slightly efficient in systemic sclerosis; v/ combination therapy using methotrexate plus steroids compared with steroids alone becomes the << gold standard >> treatment for juvenile dermatomyositis; vi/ dupilumab treatment (antibody blocking IL-4 and IL-13 receptors) is not only efficient in atopic dermatitis but also in asthma; vii/ think of eosinophilic oesophagitis in a patient with atopic dermatitis and dypshagia or food impaction; viii/ genetic A2 protein dysfunction induces NF-kB hyperactivation and an autoinflammatory disorder with features similar to Behcet's disease; ix/ no new biotherapies have shown high efficacy in systemic lupus erythematosus; x/ nanoparticles loaded with autoantigens induce Tregs and Bregs and may be a promising therapeutic option to treat auto-immune disease in the future; xi/ ipilimumab treatment (anti-CTLA4 antibody, immune checkpoint inhibitor) may induce complete remission in acute myeloid leukemia patients relapsing after haematological stem cell transplantation. Year 2016 is full of great discoveries in internal medicine keeping the dermatologist brain fully open minded. Copyright © 2016 Elsevier Masson SAS. Tous droits reserves.
Publication Type
Journal Article. Review. Systematic Review.
Year of Publication
2016

<214>

Unique Identifier

29235966

Title

The characteristics of antibodies of mice immunized by human unconventional myosin 1c.

Source

Ukrainian Biochemical Journal. 88(6):63-9, 2016 Nov-Dec.

VI 1

Status

MEDLINE

Authors

Myronovskij SL; Boiko NM; Chumak VV; Shorobura MS; Lootsyk MD; Stoika RS; Kit YY.

Authors Full Name

Myronovskij, S L; Boiko, N M; Chumak, V V; Shorobura, M S; Lootsyk, M D; Stoika, R S; Kit, Yu Ya.

Abstract

Specific antibodies produced against a protein of interest are invaluable tools for monitoring the protein structure, intracellular location and biological activity. Inoculation of murine lymphoma cells into the peritoneal cavity of immunized mice provides generation of ascitic fluid containing a significant amount of antibody with desired antigen specificity. Here we demonstrated that the intraperitoneal administration of murine lymphoma NK/Ly cells in mice immunized with 48 kDa isoform of human blood serum unconventional myosin 1c leads to generation of ascitic fluid that contained specific IgG-antibodies. These antibodies were capable of binding of the unconventional myosin 1c isolated from blood serum of patients with multiple sclerosis, rheumatoid arthritis and systemic lupus erythematosis, and could be used for diagnostics of several autoimmune diseases, the multiple sclerosis in particular.

Publication Type

Journal Article.

Year of Publication

2016

<215>

Unique Identifier

28074079

Title

Immunotherapeutic Targeting in Autoimmune Diseases.

Source

Mediators of Inflammation. 2016:1432702, 2016.

VI 1

Status

MEDLINE

Authors

Janikashvili N; Samson M; Magen E; Chikovani T.

Author NameID

Janikashvili, Nona; ORCID: https://orcid.org/0000-0003-1015-361X

Chikovani, Tinatin; ORCID: https://orcid.org/0000-0003-1125-4025

Authors Full Name

Janikashvili, Nona; Samson, Maxime; Magen, Eli; Chikovani, Tinatin.

Institution

Janikashvili, Nona. Tbilisi State Medical University, Tbilisi, Georgia.

Samson, Maxime. University Hospital of Dijon, Universite Bourgogne Franche-Comte, Dijon, France.

Magen, Eli. Barzilai University Medical Center, Ben-Gurion University of the Negev, Ashkelon, Israel.

Chikovani, Tinatin. Tbilisi State Medical University, Tbilisi, Georgia.

Publication Type

Editorial. Introductory Journal Article.

Year of Publication

2016

<216>

Unique Identifier

27993172

Title

Immune reconstitution 20 years after treatment with alemtuzumab in a rheumatoid arthritis cohort: implications for lymphocyte depleting therapies.

Source

Arthritis Research & Therapy. 18(1):302, 2016 12 20.

VI 1

Status

MEDLINE

Authors

Cooles FA; Anderson AE; Drayton T; Harry RA; Diboll J; Munro L; Thalayasingham N; Ostor AJ; Isaacs JD.

Authors Full Name

Cooles, Faye A H; Anderson, Amy E; Drayton, Tracey; Harry, Rachel A; Diboll, Julie; Munro, Lee; Thalayasingham, Nishanthi; Ostor, Andrew J K; Isaacs, John D.

Institution

Cooles, Faye A H. Institute of Cellular Medicine, Newcastle University and National Institute for Health Research Newcastle Biomedical Research Centre at Newcastle upon Tyne Hospitals NHS Foundation Trust and Newcastle University, Newcastle upon Tyne, UK.

Anderson, Amy E. Institute of Cellular Medicine, Newcastle University and National Institute for Health Research Newcastle Biomedical Research Centre at Newcastle upon Tyne Hospitals NHS Foundation Trust and Newcastle University, Newcastle upon Tyne, UK.

Drayton, Tracey. Addenbrooke's NHS trust, Cambridge, UK.

Harry, Rachel A. Institute of Cellular Medicine, Newcastle University and National Institute for Health Research Newcastle Biomedical Research Centre at Newcastle upon Tyne Hospitals NHS Foundation Trust and Newcastle University, Newcastle upon Tyne, UK.

Diboll, Julie. Institute of Cellular Medicine, Newcastle University and National Institute for Health Research Newcastle Biomedical Research Centre at Newcastle upon Tyne Hospitals NHS Foundation Trust and Newcastle University, Newcastle upon Tyne, UK.

Munro, Lee. Institute of Cellular Medicine, Newcastle University and National Institute for Health Research Newcastle Biomedical Research Centre at Newcastle upon Tyne Hospitals NHS Foundation Trust and Newcastle University, Newcastle upon Tyne, UK.

Thalayasingham, Nishanthi. Institute of Cellular Medicine, Newcastle University and National Institute for Health Research Newcastle Biomedical Research Centre at Newcastle upon Tyne Hospitals NHS Foundation Trust and Newcastle University, Newcastle upon Tyne, UK.

Ostor, Andrew J K. Addenbrooke's NHS trust, Cambridge, UK.

Isaacs, John D. Institute of Cellular Medicine, Newcastle University and National Institute for Health Research Newcastle Biomedical Research Centre at Newcastle upon Tyne Hospitals NHS

Foundation Trust and Newcastle University, Newcastle upon Tyne, UK.
john.isaacs@newcastle.ac.uk.

Abstract

BACKGROUND: Alemtuzumab, an anti-CD52 monoclonal antibody, was administered to patients with RA between 1991 and 1994. We have followed a cohort of recipients since that time and previously reported significant delays in immune reconstitution. Here we report >20 years of follow-up data from this unique cohort.

METHOD: Surviving alemtuzumab recipients were age, sex and disease duration matched with RA controls. Updated mortality and morbidity data were collected for alemtuzumab recipients. For both groups antigenic responses were assessed following influenza, Pneumovax II and combined diphtheria/tetanus/poliovirus vaccines. Circulating cytokines and lymphocyte subsets were also quantified.

RESULTS: Of 16 surviving alemtuzumab recipients, 13 were recruited: 9 recipients underwent a full clinical assessment and 4 had case notes review only. Since our last review 10 patients had died from causes of death consistent with long-standing RA, and no suggestion of compromised immune function. Compared with controls the alemtuzumab cohort had significantly reduced CD4+ and CD8+ central memory T-cells, CD5+ B cells, naive B cells and CD19+CD24hiCD38hi transitional (putative regulatory) B cells. Nonetheless vaccine responses were comparable between groups. There were significantly higher serum IL-15 and IFN-gamma levels in the alemtuzumab cohort. IL-15 levels were inversely associated with CD4+ total memory and central memory T cells.

CONCLUSION: After 20 years the immune system of alemtuzumab recipients continues to show differences from disease controls. Nonetheless mortality and morbidity data, alongside vaccination responses, do not suggest clinical immune compromise. As lymphodepleting therapies, including alemtuzumab, continue to be administered this work is important with regard to long-term immune monitoring and stages of immune recovery.

Publication Type
Journal Article. Research Support, Non-U.S. Gov't.
Year of Publication
2016

<217>

Unique Identifier

27992901

Title

Nivolumab-Induced Recurrence of Rheumatoid Arthritis in a Patient With Advanced Non-Small Cell Lung Cancer: A Case Report.

Source

Annals of Internal Medicine. 165(12):894-895, 2016 Dec 20.

VI 1

Status

MEDLINE

Authors

Syrigos K; Tsagouli S; Grapsa D.

Authors Full Name

Syrigos, Konstantinos; Tsagouli, Sofia; Grapsa, Dimitra.

Institution

Syrigos, Konstantinos. From "Sotiria" Athens General Hospital, University of Athens School of Medicine, Athens, Greece.

Tsagouli, Sofia. From "Sotiria" Athens General Hospital, University of Athens School of Medicine, Athens, Greece.

Grapsa, Dimitra. From "Sotiria" Athens General Hospital, University of Athens School of Medicine, Athens, Greece.

Publication Type

Case Reports. Letter.

Year of Publication

2016

<218>

Unique Identifier

27964798

Title

Immunization in patients with inflammatory rheumatic diseases. [Review]

Source
  Best Practice & Research in Clinical Rheumatology. 30(5):946-963, 2016 10.
VI 1
Status
  MEDLINE
Authors
  Thomas K; Vassilopoulos D.
Authors Full Name
  Thomas, Konstantinos; Vassilopoulos, Dimitrios.
Institution
  Thomas, Konstantinos. Joint Rheumatology Program, Clinical Immunology-Rheumatology Unit, 2nd Department of Medicine and Laboratory, National and Kapodistrian University of Athens School of Medicine, Hippokration General Hospital, 114 Vass. Sophias Ave., 115 27, Athens, Greece.
  Vassilopoulos, Dimitrios. Joint Rheumatology Program, Clinical Immunology-Rheumatology Unit, 2nd Department of Medicine and Laboratory, National and Kapodistrian University of Athens School of Medicine, Hippokration General Hospital, 114 Vass. Sophias Ave., 115 27, Athens, Greece. Electronic address: dvassilop@med.uoa.gr.
Abstract
  Immunization represents the most efficient and simplest intervention to prevent certain viral and bacterial infections in the general population as well as in the vulnerable population of patients with inflammatory rheumatic diseases treated with immunosuppressives. Here, we present an updated review of literature data regarding the safety and efficacy of immunizations against different pathogens in rheumatic patients treated with conventional immunosuppressives or the newer biologic agents while at the same time we provide practical guidance for the appropriate vaccine administration in this patient population. Copyright © 2016 Elsevier Ltd. All rights reserved.
Publication Type
  Journal Article. Review.
Year of Publication
  2016

<219>

Unique Identifier

27959350

Title

Progressive multifocal leukoencephalopathy associated with infliximab.

Source

Journal of the Royal College of Physicians of Edinburgh. 46(3):163-165, 2016 Sep.

VI 1

Status

MEDLINE

Authors

Sammut L; Wallis D; Holroyd C.

Authors Full Name

Sammut, L; Wallis, D; Holroyd, C.

Institution

Sammut, L. L Sammut, Rheumatology Department, University Hospital Southampton, NHS Foundation Trust, Tremona Road, Southampton SO16 6YD, UK, E-mail lukesammut@doctors.org.uk.

Abstract

A 69-year-old female with seropositive rheumatoid arthritis presented with progressive cognitive decline following treatment with infliximab and methotrexate. Cranial MRI showed non-enhancing white matter signal abnormality consistent with demyelination was seen in the antero-inferior left frontal lobe extending into the frontal opercular white matter and into the left temporal lobe white matter. Similar appearances were seen in the inferomedial right frontal lobe. Brain biopsy showed histological changes consistent with progressive multifocal leukoencephalopathy. The cerebrospinal fluid polymerase chain reaction was negative but brain tissue polymerase chain reaction was positive for JC virus. This case highlights the association of infliximab with progressive multifocal leukoencephalopathy in a patient with known seropositive rheumatoid arthritis.

Publication Type

Case Reports. Journal Article.

Year of Publication

2016

<220>

Unique Identifier

27909139

Title

Safety and Efficacy of SBI-087, a Subcutaneous Agent for B Cell Depletion, in Patients with Active Rheumatoid Arthritis: Results from a Phase II Randomized, Double-blind, Placebo-controlled Study.

Source

Journal of Rheumatology. 43(12):2094-2100, 2016 12.

VI 1

Status

MEDLINE

Authors

Damjanov N; Tlustochowicz M; Aelion J; Greenwald M; Diehl A; Bhattacharya I; Peeva E; Menon S; Gourley I.

Authors Full Name

Damjanov, Nemanja; Tlustochowicz, Malgorzata; Aelion, Jacob; Greenwald, Maria; Diehl, Annette; Bhattacharya, Indranil; Peeva, Elena; Menon, Sandeep; Gourley, Ian.

Institution

Damjanov, Nemanja. From Belgrade University School of Medicine, Belgrade, Serbia; Wojskowy Instytut Medyczny, Warsaw, Poland; Arthritis Clinic and West Tennessee Research Institute, Jackson, Tennessee; Desert Medical Advances, Palm Desert, California; Pfizer, Collegeville, Pennsylvania; Pfizer, Cambridge, Massachusetts; Janssen, Spring House, Pennsylvania, USA. nemanjadamjanov@yahoo.com.

Damjanov, Nemanja. N. Damjanov, MD, PhD, University of Belgrade School of Medicine; M. Tlustochowicz, MD, PhD, Wojskowy Instytut Medyczny; J. Aelion, MD, Arthritis Clinic and West Tennessee Research Institute; M. Greenwald, MD, Desert Medical Advances; A. Diehl, MPH, MBA, Pfizer; I. Bhattacharya, PhD, Pfizer; E. Peeva, MD, Pfizer; S. Menon, PhD, Pfizer; I. Gourley, MD, Janssen. nemanjadamjanov@yahoo.com.

Tlustochowicz, Malgorzata. From Belgrade University School of Medicine, Belgrade, Serbia; Wojskowy Instytut Medyczny, Warsaw, Poland; Arthritis Clinic and West Tennessee Research Institute, Jackson, Tennessee; Desert Medical Advances, Palm Desert, California; Pfizer, Collegeville, Pennsylvania; Pfizer, Cambridge, Massachusetts; Janssen, Spring House, Pennsylvania, USA.

Tlustochowicz, Malgorzata. N. Damjanov, MD, PhD, University of Belgrade School of Medicine; M. Tlustochowicz, MD, PhD, Wojskowy Instytut Medyczny; J. Aelion, MD, Arthritis Clinic and West Tennessee Research Institute; M. Greenwald, MD, Desert Medical Advances; A. Diehl,

MPH, MBA, Pfizer; I. Bhattacharya, PhD, Pfizer; E. Peeva, MD, Pfizer; S. Menon, PhD, Pfizer; I. Gourley, MD, Janssen.

Aelion, Jacob. From Belgrade University School of Medicine, Belgrade, Serbia; Wojskowy Instytut Medyczny, Warsaw, Poland; Arthritis Clinic and West Tennessee Research Institute, Jackson, Tennessee; Desert Medical Advances, Palm Desert, California; Pfizer, Collegeville, Pennsylvania; Pfizer, Cambridge, Massachusetts; Janssen, Spring House, Pennsylvania, USA.

Aelion, Jacob. N. Damjanov, MD, PhD, University of Belgrade School of Medicine; M. Tlustochowicz, MD, PhD, Wojskowy Instytut Medyczny; J. Aelion, MD, Arthritis Clinic and West Tennessee Research Institute; M. Greenwald, MD, Desert Medical Advances; A. Diehl, MPH, MBA, Pfizer; I. Bhattacharya, PhD, Pfizer; E. Peeva, MD, Pfizer; S. Menon, PhD, Pfizer; I. Gourley, MD, Janssen.

Greenwald, Maria. From Belgrade University School of Medicine, Belgrade, Serbia; Wojskowy Instytut Medyczny, Warsaw, Poland; Arthritis Clinic and West Tennessee Research Institute, Jackson, Tennessee; Desert Medical Advances, Palm Desert, California; Pfizer, Collegeville, Pennsylvania; Pfizer, Cambridge, Massachusetts; Janssen, Spring House, Pennsylvania, USA.

Greenwald, Maria. N. Damjanov, MD, PhD, University of Belgrade School of Medicine; M. Tlustochowicz, MD, PhD, Wojskowy Instytut Medyczny; J. Aelion, MD, Arthritis Clinic and West Tennessee Research Institute; M. Greenwald, MD, Desert Medical Advances; A. Diehl, MPH, MBA, Pfizer; I. Bhattacharya, PhD, Pfizer; E. Peeva, MD, Pfizer; S. Menon, PhD, Pfizer; I. Gourley, MD, Janssen.

Diehl, Annette. From Belgrade University School of Medicine, Belgrade, Serbia; Wojskowy Instytut Medyczny, Warsaw, Poland; Arthritis Clinic and West Tennessee Research Institute, Jackson, Tennessee; Desert Medical Advances, Palm Desert, California; Pfizer, Collegeville, Pennsylvania; Pfizer, Cambridge, Massachusetts; Janssen, Spring House, Pennsylvania, USA.

Diehl, Annette. N. Damjanov, MD, PhD, University of Belgrade School of Medicine; M. Tlustochowicz, MD, PhD, Wojskowy Instytut Medyczny; J. Aelion, MD, Arthritis Clinic and West Tennessee Research Institute; M. Greenwald, MD, Desert Medical Advances; A. Diehl, MPH, MBA, Pfizer; I. Bhattacharya, PhD, Pfizer; E. Peeva, MD, Pfizer; S. Menon, PhD, Pfizer; I. Gourley, MD, Janssen.

Bhattacharya, Indranil. From Belgrade University School of Medicine, Belgrade, Serbia; Wojskowy Instytut Medyczny, Warsaw, Poland; Arthritis Clinic and West Tennessee Research Institute, Jackson, Tennessee; Desert Medical Advances, Palm Desert, California; Pfizer, Collegeville, Pennsylvania; Pfizer, Cambridge, Massachusetts; Janssen, Spring House, Pennsylvania, USA.

Bhattacharya, Indranil. N. Damjanov, MD, PhD, University of Belgrade School of Medicine; M. Tlustochowicz, MD, PhD, Wojskowy Instytut Medyczny; J. Aelion, MD, Arthritis Clinic and West Tennessee Research Institute; M. Greenwald, MD, Desert Medical Advances; A. Diehl, MPH,

MBA, Pfizer; I. Bhattacharya, PhD, Pfizer; E. Peeva, MD, Pfizer; S. Menon, PhD, Pfizer; I. Gourley, MD, Janssen.

Peeva, Elena. From Belgrade University School of Medicine, Belgrade, Serbia; Wojskowy Instytut Medyczny, Warsaw, Poland; Arthritis Clinic and West Tennessee Research Institute, Jackson, Tennessee; Desert Medical Advances, Palm Desert, California; Pfizer, Collegeville, Pennsylvania; Pfizer, Cambridge, Massachusetts; Janssen, Spring House, Pennsylvania, USA.

Peeva, Elena. N. Damjanov, MD, PhD, University of Belgrade School of Medicine; M. Tlustochowicz, MD, PhD, Wojskowy Instytut Medyczny; J. Aelion, MD, Arthritis Clinic and West Tennessee Research Institute; M. Greenwald, MD, Desert Medical Advances; A. Diehl, MPH, MBA, Pfizer; I. Bhattacharya, PhD, Pfizer; E. Peeva, MD, Pfizer; S. Menon, PhD, Pfizer; I. Gourley, MD, Janssen.

Menon, Sandeep. From Belgrade University School of Medicine, Belgrade, Serbia; Wojskowy Instytut Medyczny, Warsaw, Poland; Arthritis Clinic and West Tennessee Research Institute, Jackson, Tennessee; Desert Medical Advances, Palm Desert, California; Pfizer, Collegeville, Pennsylvania; Pfizer, Cambridge, Massachusetts; Janssen, Spring House, Pennsylvania, USA.

Menon, Sandeep. N. Damjanov, MD, PhD, University of Belgrade School of Medicine; M. Tlustochowicz, MD, PhD, Wojskowy Instytut Medyczny; J. Aelion, MD, Arthritis Clinic and West Tennessee Research Institute; M. Greenwald, MD, Desert Medical Advances; A. Diehl, MPH, MBA, Pfizer; I. Bhattacharya, PhD, Pfizer; E. Peeva, MD, Pfizer; S. Menon, PhD, Pfizer; I. Gourley, MD, Janssen.

Gourley, Ian. From Belgrade University School of Medicine, Belgrade, Serbia; Wojskowy Instytut Medyczny, Warsaw, Poland; Arthritis Clinic and West Tennessee Research Institute, Jackson, Tennessee; Desert Medical Advances, Palm Desert, California; Pfizer, Collegeville, Pennsylvania; Pfizer, Cambridge, Massachusetts; Janssen, Spring House, Pennsylvania, USA.

Gourley, Ian. N. Damjanov, MD, PhD, University of Belgrade School of Medicine; M. Tlustochowicz, MD, PhD, Wojskowy Instytut Medyczny; J. Aelion, MD, Arthritis Clinic and West Tennessee Research Institute; M. Greenwald, MD, Desert Medical Advances; A. Diehl, MPH, MBA, Pfizer; I. Bhattacharya, PhD, Pfizer; E. Peeva, MD, Pfizer; S. Menon, PhD, Pfizer; I. Gourley, MD, Janssen.

Abstract

OBJECTIVE: To evaluate subcutaneous SBI-087 to treat rheumatoid arthritis (RA).

METHODS: A total of 210 adult patients with active RA were randomized to receive either 200 mg SBI-087 or placebo (Pbo), according to one of these patterns: SBI/Pbo/Pbo (SBI on Day 1), SBI/SBI/Pbo (SBI days 1 and 15), SBI/Pbo/SBI (SBI days 1 and 84), SBI/SBI/SBI (SBI days 1, 15, and 84), or Pbo/Pbo/Pbo (Pbo all 3 days). All patients were seropositive and taking background methotrexate. The primary endpoint was proportion of patients achieving 20%

improvement from baseline at Week 16 by American College of Rheumatology criteria (ACR20). Other outcomes included 28-joint Disease Activity Score (DAS28)-C-reactive protein (CRP), physician's and patient's global assessments of disease activity (PGA and PtGA, respectively) and Health Assessment Questionnaire-Disability Index (HAQ-DI). Peripheral CD19+ B cells were measured by high-sensitivity flow cytometer. Statistical significance was set at 2-sided alpha 0.10 level.

RESULTS: The SBI/SBI/SBI group demonstrated significant improvement in ACR20 and DAS28-CRP from Week 8 onward, sustained improvement in CRP levels from Week 12 onward, and significant improvements in PGA and PtGA in weeks 16 through 24, and in HAQ-DI at Week 24. The SBI/Pbo/Pbo and SBI/SBI/Pbo groups did not meet the primary endpoint but demonstrated improvements in several secondary endpoints. All treatment groups exhibited depletion of peripheral CD19+ B cells throughout the study. Overall, 61.5% of patients receiving SBI-087 and 55.0% of patients receiving Pbo reported adverse events.

CONCLUSION: SBI-087 effectively depleted peripheral CD20 B cells and was well tolerated. Improvements were consistently observed in the SBI/SBI/SBI group for the majority of efficacy and quality-of-life outcomes.

Publication Type

Clinical Trial, Phase II. Journal Article. Multicenter Study. Randomized Controlled Trial. Research Support, Non-U.S. Gov't.

Year of Publication

2016

<221>

Unique Identifier

27908288

Title

Granulocyte macrophage colony-stimulating factor receptor alpha expression and its targeting in antigen-induced arthritis and inflammation.

Source

Arthritis Research & Therapy. 18(1):287, 2016 12 01.

VI 1

Status

MEDLINE

Authors

Cook AD; Louis C; Robinson MJ; Saleh R; Sleeman MA; Hamilton JA.

Authors Full Name

Cook, Andrew D; Louis, Cynthia; Robinson, Matthew J; Saleh, Reem; Sleeman, Matthew A; Hamilton, John A.

Institution

Cook, Andrew D. Department of Medicine, Royal Melbourne Hospital, University of Melbourne, Parkville, Victoria, 3050, Australia. adcook@unimelb.edu.au.

Louis, Cynthia. Department of Medicine, Royal Melbourne Hospital, University of Melbourne, Parkville, Victoria, 3050, Australia.

Robinson, Matthew J. Department of Respiratory, Inflammation and Autoimmunity, MedImmune Ltd, Granta Park, Cambridge, CB21 6GH, UK.

Saleh, Reem. Department of Medicine, Royal Melbourne Hospital, University of Melbourne, Parkville, Victoria, 3050, Australia.

Sleeman, Matthew A. Department of Respiratory, Inflammation and Autoimmunity, MedImmune Ltd, Granta Park, Cambridge, CB21 6GH, UK.

Hamilton, John A. Department of Medicine, Royal Melbourne Hospital, University of Melbourne, Parkville, Victoria, 3050, Australia.

Abstract

BACKGROUND: Blockade of granulocyte macrophage colony-stimulating factor (GM-CSF) and its receptor (GM-CSFRalpha) is being successfully tested in trials in rheumatoid arthritis (RA) with clinical results equivalent to those found with neutralization of the current therapeutic targets, TNF and IL-6. To explore further the role of GM-CSF as a pro-inflammatory cytokine, we examined the effect of anti-GM-CSFRalpha neutralization on myeloid cell populations in antigen-driven arthritis and inflammation models and also compared its effect with that of anti-TNF and anti-IL-6.

METHODS: Cell population changes upon neutralization by monoclonal antibodies (mAbs) in the antigen-induced arthritis (AIA) and antigen-induced peritonitis (AIP) models were monitored by flow cytometry and microarray. Adoptive transfer of monocytes into the AIP cavity was used to assess the GM-CSF dependence of the development of macrophages and monocyte-derived dendritic cells (Mo-DCs) at a site of inflammation.

RESULTS: Therapeutic administration of a neutralizing anti-GM-CSF mAb, but not of an anti-colony-stimulating factor (anti-CSF)-1 or an anti-CSF-1R mAb, ameliorated AIA disease. Using the anti-GM-CSFRalpha mAb, the relative surface expression of different inflammatory myeloid

populations was found to be similar in the inflamed tissues in both the AIA and AIP models; however, the GM-CSFRalpha mAb, but not neutralizing anti-TNF and anti-IL-6 mAbs, preferentially depleted Mo-DCs from these sites. In addition, we were able to show that locally acting GM-CSF upregulated macrophage/Mo-DC numbers via GM-CSFR signalling in donor monocytes.

CONCLUSIONS: Our findings suggest that GM-CSF blockade modulates inflammatory responses differently to TNF and IL-6 blockade and may provide additional insight into how targeting the GM-CSF/GM-CSFRalpha system is providing efficacy in RA.

Publication Type

Journal Article. Research Support, Non-U.S. Gov't.

Year of Publication

2016

<222>

Unique Identifier

27906921

Title

Rheumatologists fail to advise people with RA to get immunised, which matters if you are under 65: An audit in a New Zealand rheumatology service.

Source

New Zealand Medical Journal. 129(1446):72-78, 2016 Dec 02.

VI 1

Status

MEDLINE

Authors

Ng B; McBain L; Grainger R.

Authors Full Name

Ng, Brendan; McBain, Lynn; Grainger, Rebecca.

Institution

Ng, Brendan. Registrar, Hutt Valley DHB.

McBain, Lynn. General Practitioner, University of Otago, Wellington.

Grainger, Rebecca. Rheumatologist, Wellington Regional Rheumatology Unit and University of Otago, Wellington.

Abstract

AIM: To assess if yearly-influenza and five-yearly pneumococcal vaccines are recommended to people with rheumatoid arthritis (RA) in a New Zealand rheumatology service in accordance with guidelines and determine patient immunisation status for these respiratory pathogens.

METHODS: Retrospective review of electronic health records of all outpatients with RA attending a regional rheumatology centre in New Zealand over a one-month period immediately after the release of the 2015 influenza vaccination.

RESULTS: The 232 people with RA in the sample had a mean age of 60.4 years with 59% having RA for more than five years. Documented advice was infrequent (<5%) at the index visit and other clinically relevant time points. Despite this, many patients were immunised. People with RA over 65 years of age were more likely to receive influenza vaccination, however, the vaccination rate was similar to the general population over 65 years of age.

CONCLUSIONS: People with RA receive recommended respiratory vaccinations despite infrequent advice for immunisation from rheumatology specialist services. However, immunisation rate in people with RA, particularly those under 65 years, remains suboptimal and multi-level interventions are required to improve this.

Publication Type

Journal Article.

Year of Publication

2016

<223>

Unique Identifier

27902999

Title

Fibromyalgia Syndrome, A Geriatric Challenge.

Source

Rhode Island Medicine. 99(12):41-44, 2016 Dec 01.

VI 1
Status
MEDLINE
Authors
Defillo-Draiby JC; Page JS.
Authors Full Name
Defillo-Draiby, Julio C; Page, John S.
Institution
Defillo-Draiby, Julio C. Assistant Professor of Medicine, Division of Geriatrics and Palliative Medicine, Alpert Medical School of Brown University; Lead Geriatrician for the Total Joint Center at The Miriam Hospital, Providence, RI.
Page, John S. Pre-Medical Student.
Publication Type
Journal Article.
Year of Publication
2016

<224>
Unique Identifier
27840083
Title
Cobrotoxin extracted from Naja atra venom relieves arthritis symptoms through anti-inflammation and immunosuppression effects in rat arthritis model.
Source
Journal of Ethnopharmacology. 194:1087-1095, 2016 Dec 24.
VI 1
Status
MEDLINE
Authors
Zhu Q; Huang J; Wang SZ; Qin ZH; Lin F.
Authors Full Name
Zhu, Qi; Huang, Jun; Wang, Shu-Zhi; Qin, Zheng-Hong; Lin, Fang.
Institution

Zhu, Qi. Department of Pharmacology, Laboratory of Aging and Nervous Diseases (SZS0703), Soochow University School of Pharmaceutical Science, Su Zhou 215123, China.. Electronic address: imzhuqi@163.com.

Huang, Jun. Department of Pharmacology, Laboratory of Aging and Nervous Diseases (SZS0703), Soochow University School of Pharmaceutical Science, Su Zhou 215123, China.. Electronic address: 947062336@qq.com.

Wang, Shu-Zhi. Department of Pharmacology, Laboratory of Aging and Nervous Diseases (SZS0703), Soochow University School of Pharmaceutical Science, Su Zhou 215123, China.. Electronic address: wangshuzhi727@126.com.

Qin, Zheng-Hong. Department of Pharmacology, Laboratory of Aging and Nervous Diseases (SZS0703), Soochow University School of Pharmaceutical Science, Su Zhou 215123, China.. Electronic address: qinzhenhong@suda.edu.cn.

Lin, Fang. Department of Pharmacology, Laboratory of Aging and Nervous Diseases (SZS0703), Soochow University School of Pharmaceutical Science, Su Zhou 215123, China.. Electronic address: bluestonelin@hotmail.com.

Abstract

ETHNOPHARMACOLOGICAL RELEVANCE: The Naja atra (Chinese cobra), primarily distributing in the low or medium altitude areas of southern China and Taiwan, was considered as a medicine in traditional Chinese medicine and used to treat pain, inflammation and arthritis.

AIM OF THE STUDY: To study the anti-inflammatory and anti-arthritic activities of cobrotoxin (CTX), an active component of the venom from Naja atra.

MATERIALS AND METHODS: Adjuvant-induced arthritis (AA) rats were used as the animal model of rheumatoid arthritis. The anti-arthritic effects of CTX were evaluated through the arthritis score, paw edema and histopathology changes of joints. The anti-inflammation effects were assayed by the level of IL-6, TNF-alpha, IL-1beta and the number of inflammatory cells in peripheral blood, as well as the proliferation of fibroblast-like synoviocytes (FLS). The immune level was valued by the proliferation of T cells and the level of CD4 and CD8.

RESULTS: CTX alleviated the disease development of AA rats according to the ameliorating arthritis score, paw edema and histopathology character. At the meanwhile, CTX decreased the levels of IL-6, TNF-alpha, IL-1beta and the numbers of inflammatory cells in peripheral blood. CTX also suppressed the abnormal increasing of CD4+ T cells/ CD8+ T cells ratio, and could significantly inhibit T cell proliferation. Consistent with its effects on inhibiting granuloma's formation, CTX inhibited the proliferation of the cultured FLSs. Further studies on inflammatory signaling in FLSs revealed that CTX could inhibit the NF-kappaB signaling pathway.

CONCLUSIONS: CTX has beneficial effects on rheumatoid arthritis by its immune regulation effects and anti-inflammation effects. The inhibition of NF-kappaB pathway partly contributes to the anti-inflammatory properties of CTX. Copyright © 2016 Elsevier Ireland Ltd. All rights reserved.

Publication Type

Journal Article.

Year of Publication

2016

<225>

Unique Identifier

27694038

Title

Targeting mast cells: Uncovering prolific therapeutic role in myriad diseases. [Review]

Source

International Immunopharmacology. 40:362-384, 2016 Nov.

VI 1

Status

MEDLINE

Authors

Singh J; Shah R; Singh D.

Authors Full Name

Singh, Jatinder; Shah, Ramanpreet; Singh, Dhandeep.

Institution

Singh, Jatinder. Department of Pharmaceutical Sciences and Drug Research, Punjabi University, Patiala 147002, Punjab, India.

Shah, Ramanpreet. Department of Pharmaceutical Sciences and Drug Research, Punjabi University, Patiala 147002, Punjab, India.

Singh, Dhandeep. Department of Pharmaceutical Sciences and Drug Research, Punjabi University, Patiala 147002, Punjab, India. Electronic address: ddd300@gmail.com.

Abstract

The mast cells are integral part of immune system and they have pleiotropic physiological functions in our body. Any type of abnormal stimuli causes the mast cells receptors to spur the otherwise innocuous mast cells to degranulate and release inflammatory mediators like histamine, cytokines, chemokines and prostaglandins. These mediators are involved in various diseases like allergy, asthma, mastocytosis, cardiovascular disorders, etc. Herein, we describe the receptors involved in degranulation of mast cells and are broadly divided into four categories: G-protein coupled receptors, ligand gated ion channels, immunoreceptors and pattern recognition receptors. Although, activation of pattern recognition receptors do not cause mast cell degranulation, but result in cytokines production. Degranulation itself is a complex process involving cascade of events like membrane fusion events and various proteins like VAMP, Syntaxins, DOCK5, SNAP-23, MARCKS. Furthermore, we described these mast cell receptors antagonists or agonists useful in treatment of myriad diseases. Like, omalizumab anti-IgE antibody is highly effective in asthma, allergic disorders treatment and recently mechanistic insight of IgE uncovered; matrix mettaloprotease inhibitor marimistat is under phase III trial for inflammation, muscular dystrophy diseases; ZPL-389 (H4 receptor antagonist) is in Phase 2a Clinical Trial for atopic dermatitis and psoriasis; JNJ3851868 an oral H4 receptor antagonist is in phase II clinical development for asthma, rheumatoid arthritis. Therefore, research is still in inchoate stage to uncover mast cell biology, mast cell receptors, their therapeutic role in myriad diseases. Copyright © 2016 Elsevier B.V. All rights reserved.

Publication Type

Journal Article. Review.

Year of Publication

2016

<226>

Unique Identifier

27671511

Title

Effect of TNF-alpha Blockade in Gingival Crevicular Fluid on Periodontal Condition of Patients with Rheumatoid Arthritis.

Source

Iranian Journal Of Immunology: IJI. 13(3):197-203, 2016 Sep.

VI 1

Status

MEDLINE

Authors

Kadkhoda Z; Amirzargar A; Esmaili Z; Vojdanian M; Akbari S.

Authors Full Name

Kadkhoda, Zeinab; Amirzargar, Aliakbar; Esmaili, Zahra; Vojdanian, Mahdi; Akbari, Solmaz.

Institution

Kadkhoda, Zeinab. Department of Periodontology, School of Dentistry, Tehran University of Medical Science, Tehran, Iran.

Abstract

BACKGROUND: Periodontitis and rheumatoid arthritis (RA) share a number of clinical and pathologic features, one of which is the presence of the tumor necrosis factor alpha (TNF-alpha)-induced bone resorption that is involved in the pathogenesis of both.

OBJECTIVES: To investigate the effect of TNF-alpha blockade on periodontal conditions in patients with active RA.

METHOD: The periodontal statuses of 36 patients (26 females, 10 males) diagnosed with active RA were evaluated both before and after anti-TNF-alpha therapy. Gingival index, bleeding on probing (BOP), probing pocket depth (PPD), oral hygiene index (OHI), and levels of TNF-alpha in gingival crevicular fluid (GCF) were measured at the baseline and 6 weeks after the treatment. Wilcoxon signed ranked test was used for statistical analyses.

RESULTS: Based on OHI (p=0.860), the level of plaque control did not change during the study period, but there was a significant reduction in gingival inflammation based on the mean BOP (p=0.049) and GI (p=0.036) before and after 6 weeks of anti-TNF-alpha therapy. The mean PPD index did not significantly differ at the baseline and 6 weeks after treatment (p=0.126).

CONCLUSION: Anti-TNF-alpha therapy might have a desirable effect on periodontal conditions and might reduce TNF-alpha level in GCF of patients with RA.

Publication Type

Journal Article.

Year of Publication

2016

<227>

Unique Identifier

27619991

Title

A Broad Blockade of Signaling from the IL-20 Family of Cytokines Potently Attenuates Collagen-Induced Arthritis.

Source

Journal of Immunology. 197(8):3029-3037, 2016 10 15.

VI 1

Status

MEDLINE

Authors

Liu X; Zhou H; Huang X; Cui J; Long T; Xu Y; Liu H; Yu R; Zhao R; Luo G; Huang A; Liang JG; Liang P.

Author NameID

Zhou, Hong; ORCID: https://orcid.org/0000-0001-7212-457X

Yu, Ruoxuan; ORCID: https://orcid.org/0000-0002-0851-1556

Zhao, Rongchuan; ORCID: https://orcid.org/0000-0001-6198-8612

Luo, Guangping; ORCID: https://orcid.org/0000-0002-7348-5526

Authors Full Name

Liu, Xinyu; Zhou, Hong; Huang, Xueqin; Cui, Jingjing; Long, Tianzhen; Xu, Yang; Liu, Haipeng; Yu, Ruoxuan; Zhao, Rongchuan; Luo, Guangping; Huang, Anliang; Liang, Joshua G; Liang, Peng.

Institution

Liu, Xinyu. Department of Biochemistry and Molecular Biology, School of Life Sciences, Sichuan University, Chengdu 610000, China.

Zhou, Hong. Department of Biochemistry and Molecular Biology, School of Life Sciences, Sichuan University, Chengdu 610000, China.

Huang, Xueqin. Department of Biochemistry and Molecular Biology, School of Life Sciences, Sichuan University, Chengdu 610000, China.

Cui, Jingjing. Department of Biochemistry and Molecular Biology, School of Life Sciences, Sichuan University, Chengdu 610000, China.

Long, Tianzhen. Department of Biochemistry and Molecular Biology, School of Life Sciences, Sichuan University, Chengdu 610000, China.

Xu, Yang. Department of Biochemistry and Molecular Biology, School of Life Sciences, Sichuan University, Chengdu 610000, China.
Liu, Haipeng. Department of Biochemistry and Molecular Biology, School of Life Sciences, Sichuan University, Chengdu 610000, China.
Yu, Ruoxuan. Department of Biochemistry and Molecular Biology, School of Life Sciences, Sichuan University, Chengdu 610000, China.
Zhao, Rongchuan. Department of Biochemistry and Molecular Biology, School of Life Sciences, Sichuan University, Chengdu 610000, China.
Luo, Guangping. Laboratory for Gene and Cell Therapy, Sichuan University, Chengdu 610041, China.
Huang, Anliang. Laboratory for Gene and Cell Therapy, Sichuan University, Chengdu 610041, China.
Liang, Joshua G. Clover Biopharmaceuticals, Chengdu 610041, China; and.
Liang, Peng. Department of Biochemistry and Molecular Biology, School of Life Sciences, Sichuan University, Chengdu 610000, China; liangpeng@scu.edu.cn liang.peng@cloverbiopharma.com.
Liang, Peng. Laboratory for Gene and Cell Therapy, Sichuan University, Chengdu 610041, China.
Liang, Peng. Clover Biopharmaceuticals, Chengdu 610041, China; and.
Liang, Peng. GenHunter Corporation, Nashville, TN 37211.
Abstract
Two heterodimeric receptors consisting of either IL-20R1 or IL-22R1 in complex with a common beta receptor subunit IL-20R2 are shared by three of the IL-20 family of cytokines: IL-19, IL-20, and IL-24. These proinflammatory cytokines have been implicated in the pathogenesis of some autoimmune diseases, including rheumatoid arthritis (RA), psoriasis, and atopic dermatitis. Although mAbs against IL-19 and IL-20 have each been shown to modulate disease severity of collagen-induced arthritis in animal models, and anti-IL-20 therapeutic Ab has exhibited some efficacy in the treatment of RA in clinical trials, benefits for a complete blockade of these functionally redundant cytokines remain to be explored. In this report, we show that recombinant human soluble IL-20R2-Fc fusion protein binds to IL-19, IL-20, and IL-24 with similar high affinity and blocks their signaling in vitro. In DBA/1 mouse collagen-induced arthritis model, recombinant human IL-20R2-Fc exhibits comparable efficacy as TNF blocker etanercept in the treatment of established arthritis, whereas the combined use of both biologics manifests little synergistic therapeutic effects. In situ ligand-receptor functional binding analysis shows that a large amount of immune infiltrates expressing high levels of TNFR and IL-20 subfamily cytokines congregate within the inflamed disease tissues. Colocalization experiments reveal that signals from IL-20R2 and TNF transduction pathways seem to converge in macrophages and function in tandem in

orchestrating the pathogenesis of RA. Elucidation of this interaction provides a better understanding of cytokine cross-talk in RA and a rationale for more effective biologic therapies that target IL-20R2 instead of individual cytokines from IL-20 family. Copyright © 2016 by The American Association of Immunologists, Inc.

Publication Type

Journal Article.

Year of Publication

2016

<228>

Unique Identifier

27606471

Title

Recommendations for Vaccination in Adult Patients with Systemic Inflammatory Rheumatic Diseases from the Portuguese Society of Rheumatology.

Source

Acta Reumatologica Portuguesa. 41(2):112-30, 2016 Apr-Jun.

VI 1

Status

MEDLINE

Authors

Cordeiro I; Duarte AC; Ferreira JF; Goncalves MJ; Meirinhos T; Rocha TM; Romao VC; Sousa S; Guedes M; Conde M; Abreu C; Aleixo MJ; Santos MJ.

Authors Full Name

Cordeiro, I; Duarte, A C; Ferreira, J F; Goncalves, M J; Meirinhos, T; Rocha, T M; Romao, V C; Sousa, S; Guedes, M; Conde, M; Abreu, C; Aleixo, M J; Santos, M J.

Abstract

BACKGROUND: Serious infections are a major cause of morbidity and mortality in systemic inflammatory rheumatic disease (SIRD) patients. Although vaccination may prevent numerous infections, vaccination uptake rates are low in this group of patients.

OBJECTIVES: To develop evidence-based recommendations for vaccination in SIRD patients.

METHODS: We searched MEDLINE (until 31 October 2014) and EMBASE (until 14 December 2014) databases, as well as the ACR and EULAR congress abstracts (2011-2014). Patients with any systemic inflammatory rheumatic disease were included and all vaccines were considered. Any safety and efficacy outcomes were admitted. Search results were submitted to title and abstract selection, followed by detailed review of suitable studies. Data were subsequently pooled according to the type of vaccine and the SIRD considered. Results were presented and discussed by a multidisciplinary panel and systematic literature review (SLR)-derived recommendations were voted according to the Delphi method. The level of agreement among rheumatologists was assessed using an online survey.

RESULTS: Eight general and seven vaccine-specific recommendations were formulated. Briefly, immunization status should routinely be assessed in all SIRD patients. The National Vaccination Program should be followed and some additional vaccines are recommended. To maximize the efficacy of vaccination, vaccines should preferably be administered 4 weeks before starting immunosuppression or, if possible when disease activity is controlled. Non-live vaccines are safe in SIRD, including immunosuppressed patients. The safety of live attenuated vaccines in immunosuppressed patients deserves further ascertainment, but might be considered in particular situations.

DISCUSSION: The present recommendations combine scientific evidence with the multidisciplinary expertise of our taskforce panel and attained desirable agreement among Portuguese rheumatologists. Vaccination recommendations need to be updated on a regular basis, as more scientific data regarding vaccination efficacy and safety, emergent infectious threats, new vaccines as well as new immunomodulatory therapies become available.

Publication Type

Journal Article. Practice Guideline.

Year of Publication

2016

<229>

Unique Identifier

27550090

Title

Functions of interleukin-34 and its emerging association with rheumatoid arthritis. [Review]
Source
Immunology. 149(4):362-373, 2016 Dec.
VI 1
Status
MEDLINE
Authors
Zhou RP; Wu XS; Xie YY; Dai BB; Hu W; Ge JF; Chen FH.
Authors Full Name
Zhou, Ren-Peng; Wu, Xiao-Shan; Xie, Ya-Ya; Dai, Bei-Bei; Hu, Wei; Ge, Jin-Fang; Chen, Fei-Hu.
Institution
Zhou, Ren-Peng. Anhui Key Laboratory of Bioactivity of Natural Products, School of Pharmacy, Anhui Medical University, Hefei, China.
Zhou, Ren-Peng. The Key Laboratory of Anti-inflammatory and Immune Medicine, Ministry of Education, Anhui Medical University, Hefei, China.
Wu, Xiao-Shan. Anhui Key Laboratory of Bioactivity of Natural Products, School of Pharmacy, Anhui Medical University, Hefei, China.
Wu, Xiao-Shan. The Key Laboratory of Anti-inflammatory and Immune Medicine, Ministry of Education, Anhui Medical University, Hefei, China.
Xie, Ya-Ya. Anhui Key Laboratory of Bioactivity of Natural Products, School of Pharmacy, Anhui Medical University, Hefei, China.
Xie, Ya-Ya. The Key Laboratory of Anti-inflammatory and Immune Medicine, Ministry of Education, Anhui Medical University, Hefei, China.
Dai, Bei-Bei. Anhui Key Laboratory of Bioactivity of Natural Products, School of Pharmacy, Anhui Medical University, Hefei, China.
Dai, Bei-Bei. The Key Laboratory of Anti-inflammatory and Immune Medicine, Ministry of Education, Anhui Medical University, Hefei, China.
Hu, Wei. Anhui Key Laboratory of Bioactivity of Natural Products, School of Pharmacy, Anhui Medical University, Hefei, China.
Hu, Wei. The Key Laboratory of Anti-inflammatory and Immune Medicine, Ministry of Education, Anhui Medical University, Hefei, China.
Ge, Jin-Fang. Anhui Key Laboratory of Bioactivity of Natural Products, School of Pharmacy, Anhui Medical University, Hefei, China.
Ge, Jin-Fang. The Key Laboratory of Anti-inflammatory and Immune Medicine, Ministry of Education, Anhui Medical University, Hefei, China.

Chen, Fei-Hu. Anhui Key Laboratory of Bioactivity of Natural Products, School of Pharmacy, Anhui Medical University, Hefei, China. cfhchina@sohu.com, zrpahmu@sina.com.
Chen, Fei-Hu. The Key Laboratory of Anti-inflammatory and Immune Medicine, Ministry of Education, Anhui Medical University, Hefei, China. cfhchina@sohu.com, zrpahmu@sina.com.
Abstract
Rheumatoid arthritis (RA) is a systemic autoimmune disease characterized by chronic, synovial inflammation affecting multiple joints, finally leading to extra-articular lesions for which limited effective treatment options are currently available. Interleukin-34 (IL-34), recently discovered as the second colony-stimulating factor-1 receptor (CSF-1R) ligand, is a newly discovered cytokine. Accumulating evidence has disclosed crucial roles of IL-34 in the proliferation and differentiation of mononuclear phagocyte lineage cells, osteoclastogenesis and inflammation. Recently, IL-34 was detected at high levels in patients with active RA and in experimental models of inflammatory arthritis. Blockade of functional IL-34 with a specific monoclonal antibody can reduce the severity of inflammatory arthritis, suggesting that targeting IL-34 or its receptors may constitute a novel therapeutic strategy for autoimmune diseases such as RA. Here, we have comprehensively discussed the structure and biological functions of IL-34, and reviewed recent advances in our understanding of the emerging role of IL-34 in the development of RA as well as its potential utility as a therapeutic target. Copyright © 2016 John Wiley & Sons Ltd.
Publication Type
Journal Article. Review.
Year of Publication
2016

<230>
Unique Identifier
27539666
Title
Immune checkpoints and rheumatic diseases: what can cancer immunotherapy teach us?.
[REVIEW]
Source
Nature Reviews Rheumatology. 12(10):593-604, 2016 10.
Status
MEDLINE

Authors
van der Vlist M; Kuball J; Radstake TR; Meyaard L.
Authors Full Name
van der Vlist, Michiel; Kuball, Jurgen; Radstake, Timothy R D; Meyaard, Linde.
Institution
van der Vlist, Michiel. Laboratory of Translational Immunology, University Medical Center Utrecht, 3584 CX Utrecht, The Netherlands.
van der Vlist, Michiel. Department of Immunology, University Medical Center Utrecht, 3584 CX Utrecht, The Netherlands.
Kuball, Jurgen. Laboratory of Translational Immunology, University Medical Center Utrecht, 3584 CX Utrecht, The Netherlands.
Kuball, Jurgen. Department of Hematology, University Medical Center Utrecht, 3584 CX Utrecht, The Netherlands.
Radstake, Timothy R D. Laboratory of Translational Immunology, University Medical Center Utrecht, 3584 CX Utrecht, The Netherlands.
Radstake, Timothy R D. Department of Rheumatology and Clinical Immunology, University Medical Center Utrecht, 3584 CX Utrecht, The Netherlands.
Meyaard, Linde. Laboratory of Translational Immunology, University Medical Center Utrecht, 3584 CX Utrecht, The Netherlands.
Meyaard, Linde. Department of Immunology, University Medical Center Utrecht, 3584 CX Utrecht, The Netherlands.
Abstract
The recent success of immune checkpoint blockade in cancer therapy illustrates the importance of the inhibitory receptors cytotoxic T-lymphocyte-associated antigen 4 (CTLA4) and programmed cell death protein 1 (PD1) in the regulation of antitumour immune responses. However, blocking signalling by these inhibitory immune checkpoint receptors is also associated with substantial inflammatory effects that can resemble autoimmune responses, which is consistent with the role of these receptors in protecting the host from excessive inflammation. The human genome encodes over 300 inhibitory receptors, which represent as many opportunities to modulate inflammation in a disease-specific and tissue-specific manner. We argue that rheumatologists and oncologists should join forces to study these inhibitory immune molecules. An improved understanding of these immune checkpoints will enable both fields to make progress in exploiting inhibitory immune receptors therapeutically. In this Review, we discuss data from studies reporting the adverse inflammatory effects of cancer therapies that target immune checkpoints. We discuss the potential implications of these findings on the biological understanding of autoimmune rheumatic diseases and highlight therapeutic strategies that could be used to target inhibitory receptors for the treatment of these conditions.

Publication Type
 Journal Article. Review. Research Support, Non-U.S. Gov't.
Year of Publication
 2016

<231>
Unique Identifier
 27485081
Title
 Perspectives of ofatumumab as CD20 targeted therapy in rheumatoid arthritis and other autoimmune diseases. [Review]
Source
 Immunotherapy. 8(9):1091-6, 2016 09.
VI 1
Status
 MEDLINE
Authors
 Pers YM; Jorgensen C.
Authors Full Name
 Pers, Yves Marie; Jorgensen, Christian.
Institution
 Pers, Yves Marie. Inserm, U1183, Hopital Saint-Eloi, Montpellier, F-34295 France.
 Pers, Yves Marie. Universite MONTPELLIER, UFR de Medecine, Montpellier, F-34000 France.
 Pers, Yves Marie. Service d'immuno-Rhumatologie, CHU Lapeyronie, Montpellier, F-34295 France.
 Jorgensen, Christian. Inserm, U1183, Hopital Saint-Eloi, Montpellier, F-34295 France.
 Jorgensen, Christian. Universite MONTPELLIER, UFR de Medecine, Montpellier, F-34000 France.
 Jorgensen, Christian. Service d'immuno-Rhumatologie, CHU Lapeyronie, Montpellier, F-34295 France.
Abstract
 Rheumatoid arthritis (RA) is a chronic inflammatory autoimmune condition viewed as a severe destructive disease. The treatment strategies include anti-CD20 monoclonal antibody (mAb)-

targeting B cells. Ofatumumab specifically targets a membrane-proximal epitope on the CD20 molecule distinct from other anti-CD20 antibodies including rituximab and ocrelizumab, and bind the epitope located on the large loop of CD20. This explains a more durable B-cell depletion and a different pharmacodynamic. We review the pharmacodynamic of B-cell depletion and analyze the results in RA and other B-cell-mediated autoimmune diseases. The randomized trial in RA showed clinical efficacy comparable to rituximab at week 24. However, structural impact has not been demonstrated. Studies including RA patients refractory to rituximab would be useful to define the optimal strategy of ofatumumab therapy.

Publication Type

Journal Article. Review.

Year of Publication

2016

<232>

Unique Identifier

27472273

Title

Adverse Events Associated with Immune Checkpoint Blockade in Patients with Cancer: A Systematic Review of Case Reports. [REVIEW]

Source

PLoS ONE [Electronic Resource]. 11(7):e0160221, 2016.

VI 1

Status

MEDLINE

Authors

Abdel-Wahab N; Shah M; Suarez-Almazor ME.

Authors Full Name

Abdel-Wahab, Noha; Shah, Mohsin; Suarez-Almazor, Maria E.

Institution

Abdel-Wahab, Noha. Section of Rheumatology and Clinical Immunology, Department of General Internal Medicine, University of Texas MD Anderson Cancer Center, Houston, Texas, United States of America.

Abdel-Wahab, Noha. Rheumatology and Rehabilitation Department, Assiut University Hospitals, Assiut, Egypt.
Shah, Mohsin. Section of Rheumatology and Clinical Immunology, Department of General Internal Medicine, University of Texas MD Anderson Cancer Center, Houston, Texas, United States of America.
Suarez-Almazor, Maria E. Section of Rheumatology and Clinical Immunology, Department of General Internal Medicine, University of Texas MD Anderson Cancer Center, Houston, Texas, United States of America.
Abstract
BACKGROUND: Three checkpoint inhibitor drugs have been approved by the US Food and Drug Administration for use in specific types of cancers. While the results are promising, severe immunotherapy-related adverse events (irAEs) have been reported.

OBJECTIVES: To conduct a systematic review of case reports describing the occurrence of irAEs in patients with cancer following checkpoint blockade therapy, primarily to identify potentially unrecognized or unusual clinical findings and toxicity.

DATA SOURCES: We searched Medline, EMBASE, Web of Science, PubMed ePubs, and Cochrane CENTRAL with no restriction through August 2015.

STUDY SELECTION: Studies reporting cases of cancer develop irAEs following treatment with anti CTLA-4 (ipilimumab) or anti PD-1 (nivolumab or pembrolizumab) antibodies were included.

DATA EXTRACTION: We extracted data on patient characteristics, irAEs characteristics, how irAEs were managed, and their outcomes.

DATA SYNTHESIS: 191 publications met inclusion criteria, reporting on 251 cases. Most patients had metastatic melanoma (95.6%), and the majority were treated with ipilimumab (93.2%). Autoimmune colitis, hepatitis, endocrinopathies, and cutaneous irAEs were the most frequently reported irAEs in ipilimumab treated patients. A broad spectrum of toxicities were reported for almost every body system. Moreover, well-defined diseases such as sarcoidosis, polyarthritis, polymyalgia rheumatica/arteritis, lupus, celiac disease, dermatomyositis, and Vogt-Koyanagi-like syndrome were reported. The most frequent irAEs reported with anti-PD1 agents were dermatitis for pembrolizumab, and thyroid disease and pneumonitis for nivolumab. Complete resolution of adverse events occurred in most cases. However, persistent irAEs and death were reported, mainly in patients treated with ipilimumab.

LIMITATIONS: Our study is limited by information available in the original reports.

CONCLUSIONS: Evidence from case reports shows that cancer patients develop irAEs following checkpoint blockade therapy, and can occasionally develop clearly defined autoimmune systemic diseases. While discontinuation of therapy and/or treatment can result in resolution of irAEs, long-term sequelae and death have been reported.

Publication Type

Journal Article. Review. Systematic Review.

Year of Publication

2016

<233>

Unique Identifier

27457285

Title

Therapeutic potential of cysteine-rich protein 61 in rheumatoid arthritis. [Review]

Source

Gene. 592(1):179-185, 2016 Oct 30.

VI 1

Status

MEDLINE

Authors

Xu T; He YH; Wang MQ; Yao HW; Ni MM; Zhang L; Meng XM; Huang C; Ge YX; Li J.

Authors Full Name

Xu, Tao; He, Ying-Hua; Wang, Ming-Quan; Yao, Hong-Wei; Ni, Ming-Ming; Zhang, Lei; Meng, Xiao-Ming; Huang, Cheng; Ge, Yun-Xuan; Li, Jun.

Institution

Xu, Tao. School of Pharmacy, Anhui Key Laboratory of Bioactivity of Natural Products, Anhui Medical University, Hefei 230032, China.

He, Ying-Hua. School of Pharmacy, Anhui Key Laboratory of Bioactivity of Natural Products, Anhui Medical University, Hefei 230032, China.

Wang, Ming-Quan. Department of Radiology, The First Affiliated Hospital of Anhui Medical University, Hefei, Anhui 230031, China.

Yao, Hong-Wei. School of Pharmacy, Anhui Key Laboratory of Bioactivity of Natural Products, Anhui Medical University, Hefei 230032, China.
Ni, Ming-Ming. Nanjing Municipal Hospital of T.C.M., The Third Affiliated Hospital of Nanjing University of T.C.M., Nanjing 210001, China.
Zhang, Lei. School of Pharmacy, Anhui Key Laboratory of Bioactivity of Natural Products, Anhui Medical University, Hefei 230032, China.
Meng, Xiao-Ming. School of Pharmacy, Anhui Key Laboratory of Bioactivity of Natural Products, Anhui Medical University, Hefei 230032, China.
Huang, Cheng. School of Pharmacy, Anhui Key Laboratory of Bioactivity of Natural Products, Anhui Medical University, Hefei 230032, China.
Ge, Yun-Xuan. Beijing Institute of Radiation Medicine, Academy of Military Medical Sciences, Beijing 100850, China.
Li, Jun. School of Pharmacy, Anhui Key Laboratory of Bioactivity of Natural Products, Anhui Medical University, Hefei 230032, China. Electronic address: lj@ahmu.edu.cn.
Abstract
Cysteine-rich protein 61 (Cyr61)/CCN1, a product of an immediate early gene, can directly accommodate cell adhesion and migratory processes whilst simultaneously regulating the production of other cytokines and chemokines through paracrine and autocrine feedback loops. This intricate functionality of Cyr61 indicate its important role in targeting components of the infectious or chronic inflammatory disease processes including rheumatoid arthritis (RA). Recent work has focused on the role of Cyr61 in RA. For example, Cyr61 induced proIL-1beta production in FLS via the AKT-dependent NF-kappaB signaling pathway. Moreover, Cyr61-siRNA decreased the levels of matrix metalloproteinase (MMP)-3 and MMP-13, and induced apoptosis in RA-FLS cells. These results indicated that Cyr61 may represent a novel target for the treatment of RA. In this article we will introduce the molecular properties of Cyr61, discuss the function of Cyr61, and the therapeutic potential of modulating the Cyr61 in RA. Copyright © 2016 Elsevier B.V. All rights reserved.
Publication Type
Journal Article. Review.
Year of Publication
2016

<234>

Unique Identifier

27427418

Title

Reactive Oxygen Species Regulate Innate But Not Adaptive Inflammation in ZAP70-Mutated SKG Arthritic Mice.

Source

American Journal of Pathology. 186(9):2353-63, 2016 09.

VI 1

Status

MEDLINE

Authors

Guerard S; Holmdahl R; Wing K.

Authors Full Name

Guerard, Simon; Holmdahl, Rikard; Wing, Kajsa.

Institution

Guerard, Simon. Division of Medical Inflammation Research, Department of Medical Biochemistry and Biophysics, Karolinska Institutet, Stockholm, Sweden.

Holmdahl, Rikard. Division of Medical Inflammation Research, Department of Medical Biochemistry and Biophysics, Karolinska Institutet, Stockholm, Sweden.

Wing, Kajsa. Division of Medical Inflammation Research, Department of Medical Biochemistry and Biophysics, Karolinska Institutet, Stockholm, Sweden. Electronic address: kajsa.wing@ki.se.

Abstract

Polysaccharides from Saccharomyces cerevisiae can induce arthritis, ileitis, and interstitial pneumonitis in BALB/c ZAP70 (W163C)-mutant (SKG) mice via T helper 17-cell-dependent pathways. However, little is known regarding the factors influencing disease severity. We investigated mannan-induced arthritis in SKG mice and how NADPH oxidase 2-derived reactive oxygen species (ROS) regulate disease. SKG mice were highly susceptible to both IL-17-mediated T-cell-driven arthritis and T-cell-independent acute psoriasis-like dermatitis. In vivo imaging revealed more ROS in joints of arthritic SKG mice compared to wild-type mice, which links ROS and joint inflammation. Still, ROS deficiency in SKG.Ncf1(m1j/m1j) mice greatly increased severity of arthritis and dermatitis, a difference that could not be attributed to increased T-cell activation, thymic selection, or antibody production. However, when ROS production was restored in CD68(+) macrophages, inflammation reverted to baseline, demonstrating a regulatory role of macrophage-derived ROS in autoimmunity. Thus, arthritis in SKG mice is a useful model to study the role of ROS in innate-driven chronic inflammation independently of adaptive immunity. Copyright © 2016 American Society for Investigative Pathology. Published by Elsevier Inc. All rights reserved.

Publication Type

Journal Article. Research Support, Non-U.S. Gov't.

Year of Publication

2016

<235>

Unique Identifier

27427404

Title

Avenues to autoimmune arthritis triggered by diverse remote inflammatory challenges.

Source

Journal of Autoimmunity. 73:120-9, 2016 09.

VI 1

Status

MEDLINE

Authors

Chevalier N; Tan JK; Mason LJ; Robert R; McKenzie CI; Lim F; Wong CH; Macia L; Thorburn AN; Russ BE; Masters SL; Mackay CR.

Authors Full Name

Chevalier, Nina; Tan, Jian K; Mason, Linda J; Robert, Remy; McKenzie, Craig I; Lim, Florence; Wong, Connie H; Macia, Laurence; Thorburn, Alison N; Russ, Brendan E; Masters, Seth L; Mackay, Charles R.

Institution

Chevalier, Nina. Department of Rheumatology and Clinical Immunology, Medical Center, University of Freiburg, Germany, 79106 Freiburg, Germany; Infection and Immunity Program, Monash Biomedicine Discovery Institute, Monash University, 3800 VIC, Australia; Immunology Department, Garvan Institute of Medical Research, 2010 Sydney, Australia. Electronic address: nina.chevalier@uniklinik-freiburg.de.

Tan, Jian K. Infection and Immunity Program, Monash Biomedicine Discovery Institute, Monash University, 3800 VIC, Australia.

Mason, Linda J. Infection and Immunity Program, Monash Biomedicine Discovery Institute, Monash University, 3800 VIC, Australia.

Robert, Remy. Infection and Immunity Program, Monash Biomedicine Discovery Institute, Monash University, 3800 VIC, Australia.
McKenzie, Craig I. Infection and Immunity Program, Monash Biomedicine Discovery Institute, Monash University, 3800 VIC, Australia.
Lim, Florence. Infection and Immunity Program, Monash Biomedicine Discovery Institute, Monash University, 3800 VIC, Australia.
Wong, Connie H. Center for Inflammatory Disease, Monash University, 3800 Melbourne, Australia.
Macia, Laurence. Infection and Immunity Program, Monash Biomedicine Discovery Institute, Monash University, 3800 VIC, Australia; School of Medical Sciences, The University of Sydney, 2006 Sydney, Australia; Department of Physiology, School of Medical Sciences, Charles Perkins Centre, The University of Sydney, 2006 Sydney, Australia.
Thorburn, Alison N. Infection and Immunity Program, Monash Biomedicine Discovery Institute, Monash University, 3800 VIC, Australia.
Russ, Brendan E. Department of Microbiology and Immunology, The University of Melbourne, 3010 Melbourne, Australia.
Masters, Seth L. Division of Inflammation, The Walter and Eliza Hall Institute of Medical Research, 3052 Melbourne, Australia.
Mackay, Charles R. Infection and Immunity Program, Monash Biomedicine Discovery Institute, Monash University, 3800 VIC, Australia. Electronic address: charles.mackay@monash.edu.
Abstract
Environmental factors contribute to development of autoimmune diseases. For instance, human autoimmune arthritis can associate with intestinal inflammation, cigarette smoking, periodontal disease, and various infections. The cellular and, molecular pathways whereby such remote challenges might precipitate arthritis or flares remain unclear. Here, we used a transfer model of self-reactive arthritis-inducing CD4(+) cells from KRNtg mice that, upon transfer, induce a very mild form of autoinflammatory arthritis in recipient animals. This model enabled us to identify external factors that greatly aggravated disease. We show that several distinct challenges precipitated full-blown arthritis, including intestinal inflammation through DSS-induced colitis, and bronchial stress through Influenza infection. Both triggers induced strong IL-17 expression primarily in self-reactive CD4(+) cells in lymph nodes draining the site of inflammation. Moreover, treatment of mice with IL-1beta greatly exacerbated arthritis, while transfer of KRNtg CD4(+) cells lacking IL-1R significantly reduced disease and IL-17 expression. Thus, IL-1beta enhances the autoaggressive potential of self-reactive CD4(+) cells, through increased Th17 differentiation, and this influences inflammatory events in the joints. We propose that diverse challenges that cause remote inflammation (lung infection or colitis, etc.) result in IL-1beta-driven Th17 differentiation, and this precipitates arthritis in genetically susceptible individuals. Thus the etiology of

autoimmune inflammatory arthritis likely relates to diverse triggers that converge to a common pathway involving IL-1beta production and Th17 cell distribution. Copyright © 2016 Elsevier Ltd. All rights reserved.

Publication Type

Journal Article. Research Support, Non-U.S. Gov't.

Year of Publication

2016

<236>

Unique Identifier

27418053

Title

[Inflammasome and gout]. [Review] [German]

Source

Zeitschrift fur Rheumatologie. 75(6):537-41, 2016 Aug.

VI 1

Status

MEDLINE

Authors

Aringer M; Hedrich C; Tausche AK.

Authors Full Name

Aringer, M; Hedrich, C; Tausche, A-K.

Institution

Aringer, M. Rheumatologie, Medizinische Klinik und Poliklinik III, Universitatsklinikum und Medizinische Fakultat Carl Gustav Carus an der Technischen Universitat Dresden, Fetscherstrase 74, 01307, Dresden, Deutschland. martin.aringer@uniklinikum-dresden.de.

Hedrich, C. Klinik und Poliklinik fur Kinder- und Jugendmedizin, Universitatsklinikum und Medizinische Fakultat Carl Gustav Carus an der Technischen Universitat Dresden, Dresden, Deutschland.

Tausche, A-K. Rheumatologie, Medizinische Klinik und Poliklinik III, Universitatsklinikum und Medizinische Fakultat Carl Gustav Carus an der Technischen Universitat Dresden, Fetscherstrase 74, 01307, Dresden, Deutschland.

Publication Type

Journal Article. Review.
Year of Publication
2016

<237>
Unique Identifier
27379738
Title
[Clinical immunology in rheumatology : State of the art]. [Review] [German]
Source
Zeitschrift fur Rheumatologie. 75(6):526-30, 2016 Aug.
VI 1
Status
MEDLINE
Authors
Peter HH.
Authors Full Name
Peter, H H.
Institution
Peter, H H. Centrum fur chronische Immundefizienz (CCI) im ZTZ, Breisacherstr. 115, 79106, Freiburg, Deutschland. hans-hartmut.peter@uniklinik-freiburg.de.
Publication Type
Journal Article. Review.
Year of Publication
2016

<238>
Unique Identifier
27379736

Title

[Therapy-resistant cells of the B cell line]. [Review] [German]

Source

Zeitschrift fur Rheumatologie. 75(6):556-9, 2016 Aug.

VI 1

Status

MEDLINE

Authors

Hoyer BF; Dorner T.

Authors Full Name

Hoyer, B F; Dorner, T.

Institution

Hoyer, B F. Medizinische Klinik mit Schwerpunkt Rheumatologie und klinische Immunologie, Charite - Universitatsmedizin Berlin, Chariteplatz 1, 10117, Berlin, Deutschland. hoyer@drfz.de.

Dorner, T. Medizinische Klinik mit Schwerpunkt Rheumatologie und klinische Immunologie, Charite - Universitatsmedizin Berlin, Chariteplatz 1, 10117, Berlin, Deutschland.

Publication Type

Journal Article. Review.

Year of Publication

2016

<239>

Unique Identifier

27372078

Title

Influenza and pneumococcal vaccination in patients with rheumatoid arthritis in comparison with age- and sex-matched controls: results of a claims data analysis.

Source

Rheumatology International. 36(9):1255-63, 2016 Sep.

VI 1

Status

MEDLINE

Authors

Luque Ramos A; Hoffmann F; Callhoff J; Zink A; Albrecht K.
Author NameID
Luque Ramos, A; ORCID: http://orcid.org/0000-0002-5625-649X
Authors Full Name
Luque Ramos, A; Hoffmann, F; Callhoff, J; Zink, A; Albrecht, K.
Institution
Luque Ramos, A. Department of Health Services Research, Carl von Ossietzky University, Oldenburg, Germany. andres.luque.ramos@uni-oldenburg.de.
Hoffmann, F. Department of Health Services Research, Carl von Ossietzky University, Oldenburg, Germany.
Callhoff, J. Epidemiology, German Rheumatism Research Centre, Berlin, Germany.
Zink, A. Epidemiology, German Rheumatism Research Centre, Berlin, Germany.
Zink, A. Department of Rheumatology and Clinical Immunology, Charite University Hospital, Berlin, Germany.
Albrecht, K. Epidemiology, German Rheumatism Research Centre, Berlin, Germany.
Abstract
The aim of this study was to assess the vaccination status for influenza and pneumonia and the prevalence of hospitalised pneumonia in rheumatoid arthritis (RA) patients and population controls in Germany. Members of a large statutory health insurance fund in Germany who were continuously insured between 2009 and 2013 and had a diagnosis of RA in 2013 were age and sex matched 1:5 to members without RA. Pneumococcal and influenza vaccinations were evaluated with regard to age, sex and region of residence. Logistic regression models were used to determine predictors for influenza vaccination in RA patients. Prevalences of pneumonia that required hospitalisation were compared to regional vaccination rates. The data of 111,482 RA patients and 557,410 matched controls were available for analysis. Compared to controls, RA patients were vaccinated more frequently against influenza (40.8 vs. 32.2 %) and pneumonia (15.0 vs. 10.0 %). Vaccination rates increased with older age and differed between the federal states (highest in East Germany, lowest in South Germany). The region of residence, comorbidities, rheumatologic care and biologic treatment was associated with a higher probability of an influenza vaccination. Prevalences of pneumonia that required hospitalisation were 2-3 times higher in patients compared to controls and tended to be higher in regions with low vaccination rates. The increased pneumonia prevalence in RA patients confirms their status as a risk group. RA patients are vaccinated more frequently than controls, but vaccination rates are still low. The lower pneumonia prevalence in East Germany indicates that vaccination may help to reduce pneumonia in RA.
Publication Type
Journal Article.

Year of Publication
  2016

<240>
Unique Identifier
  27301320
Title
  Collagen epitope expression on B cells is sufficient to confer tolerance to collagen-induced arthritis.
Source
  Arthritis Research & Therapy. 18(1):140, 2016 06 14.
VI 1
Status
  MEDLINE
Authors
  Andersson SE; Eneljung T; Tengvall S; Jirholt P; Stern A; Henningsson L; Liang B; Thorarinsdottir K; Kihlberg J; Holmdahl R; Martensson IL; Gustafsson K; Gjertsson I.
Authors Full Name
  Andersson, Sofia E M; Eneljung, Tove; Tengvall, Sara; Jirholt, Pernilla; Stern, Anna; Henningsson, Louise; Liang, Bibo; Thorarinsdottir, Katrin; Kihlberg, Jan; Holmdahl, Rikard; Martensson, Inga-Lill; Gustafsson, Kenth; Gjertsson, Inger.
Institution
  Andersson, Sofia E M. Department of Rheumatology and Inflammation Research, Institute for Medicine, Sahlgrenska Academy, University of Gothenburg, Box 480, SE 405 30, Gothenburg, Sweden.
  Eneljung, Tove. Department of Rheumatology and Inflammation Research, Institute for Medicine, Sahlgrenska Academy, University of Gothenburg, Box 480, SE 405 30, Gothenburg, Sweden.
  Eneljung, Tove. Sahlgrenska University Hospital, Gothenburg, Sweden.
  Tengvall, Sara. Department of Rheumatology and Inflammation Research, Institute for Medicine, Sahlgrenska Academy, University of Gothenburg, Box 480, SE 405 30, Gothenburg, Sweden.
  Tengvall, Sara. Sahlgrenska University Hospital, Gothenburg, Sweden.

Jirholt, Pernilla. Department of Rheumatology and Inflammation Research, Institute for Medicine, Sahlgrenska Academy, University of Gothenburg, Box 480, SE 405 30, Gothenburg, Sweden.
Stern, Anna. Department of Rheumatology and Inflammation Research, Institute for Medicine, Sahlgrenska Academy, University of Gothenburg, Box 480, SE 405 30, Gothenburg, Sweden.
Henningsson, Louise. Department of Rheumatology and Inflammation Research, Institute for Medicine, Sahlgrenska Academy, University of Gothenburg, Box 480, SE 405 30, Gothenburg, Sweden.
Liang, Bibo. Medical Inflammation Research, Department of Medical Biochemistry and Biophysics, Karolinska Institutet, Solna, Sweden.
Liang, Bibo. Southern Medical University, Guangzhou, People's Republic China.
Thorarinsdottir, Katrin. Department of Rheumatology and Inflammation Research, Institute for Medicine, Sahlgrenska Academy, University of Gothenburg, Box 480, SE 405 30, Gothenburg, Sweden.
Thorarinsdottir, Katrin. Sahlgrenska University Hospital, Gothenburg, Sweden.
Kihlberg, Jan. Department of Chemistry, BMC, Uppsala University, Uppsala, Sweden.
Holmdahl, Rikard. Medical Inflammation Research, Department of Medical Biochemistry and Biophysics, Karolinska Institutet, Solna, Sweden.
Holmdahl, Rikard. Southern Medical University, Guangzhou, People's Republic China.
Martensson, Inga-Lill. Department of Rheumatology and Inflammation Research, Institute for Medicine, Sahlgrenska Academy, University of Gothenburg, Box 480, SE 405 30, Gothenburg, Sweden.
Gustafsson, Kenth. Molecular Immunology Unit, UCL Institute of Child Health, London, UK.
Gjertsson, Inger. Department of Rheumatology and Inflammation Research, Institute for Medicine, Sahlgrenska Academy, University of Gothenburg, Box 480, SE 405 30, Gothenburg, Sweden. inger.gjertsson@rheuma.gu.se.
Gjertsson, Inger. Sahlgrenska University Hospital, Gothenburg, Sweden. inger.gjertsson@rheuma.gu.se.
Abstract
BACKGROUND: The mechanisms underlying tolerance induction and maintenance in autoimmune arthritis remain elusive. In a mouse model of rheumatoid arthritis, collagen type II (CII)-induced arthritis, we explore the contribution of B cells to antigen-specific tolerance.

METHODS: To generate expression of the CII-peptide specifically on B-cell major histocompatibility complex type II, lentiviral-based gene therapy including a B-cell-specific Igk promoter was used.

RESULTS: Presentation of the CII-peptide on B cells significantly reduced the frequency and severity of arthritis as well as the serum levels of CII -specific IgG antibodies. Further, both frequency and suppressive function of regulatory T cells were increased in tolerized mice. Adoptive transfer of regulatory T cells from tolerized mice to naive mice ameliorated the development of CII-induced arthritis.

CONCLUSION: Our data suggest that endogenous presentation of the CII-peptide on B cells is one of the key contributors to arthritis tolerance induction and maintenance.

Publication Type

Journal Article. Research Support, Non-U.S. Gov't.

Year of Publication

2016

<241>

Unique Identifier

27268027

Title

Vaccination with endosomal unknown epitopes produces therapeutic response in rheumatoid arthritis patients and modulates adjuvant arthritis of rats.

Source

Journal of Translational Medicine. 14(1):162, 2016 06 07.

VI 1

Status

MEDLINE

Authors

Caruso I; Santandrea S; Gismondo M; Lombardi A; Montrone F; Caruso EM; Sarzi Puttini P.

Authors Full Name

Caruso, Innocenzo; Santandrea, Salvatore; Gismondo, Mariarita; Lombardi, Alessandra; Montrone, Franco; Caruso, Enzo Massimo; Sarzi Puttini, Piercarlo.

Institution

Caruso, Innocenzo. Past Chief Rheumatology Unit, L. Sacco Hospital Milano Italy, Corso Italia 11, 20122, Milan, Italy. innocenzo.caruso@tiscali.it.

Santandrea, Salvatore. Rheumatology UOC, L. Sacco Hospital Milano Italy, Milan, Italy.

Gismondo, Mariarita. Director of microbiology chair, University of Milan, L. Sacco Hospital Milano Italy, Milan, Italy.
Lombardi, Alessandra. Microbiology and Virology UO, L. Sacco Hospital milano Italy, Milan, Italy.
Montrone, Franco. Associazione Italiana Contro l'Artrite (AICA), Milan, Italy.
Caruso, Enzo Massimo. Orthopedic Unit, Istituto Clinico San Siro, Milan, Italy.
Sarzi Puttini, Piercarlo. Chief of rheumatology Unit. L. Sacco Hospital Milano Italy, Milan, Italy.
Abstract
BACKGROUND: Our previous results showed that intrasynovial Rifamycin SV caused the lysis of synoviocites and freed the autoantigens which in turn stimulated the immunoregulatory rather than autoreactive T cell response in rheumatoid patients. Here, we hypothesize that disruption in vitro of peripheral blood mononuclear cells, by freeze/thawing or by lytic action of Rifamycin SV, would induce the release of endosomal pathogenic autoantigens from APCs present in the circulation, which could then be isolated from degrading enzymes by ultrafiltration.

METHODS: The preparation of the ultrafiltrates are based on the rupture of PBMCs (5 x 10(6) cells/mL) by the addition of Rifamycin SV in culture (250 mug/mL), which causes the lysis of 90 % of the cells in 3 h, or by three cycles of freeze/thawing of the PBMC, from -80 degreeC to room temperature. The lysate and the fragmented cells were then centrifuged and ultrafiltered by passage through a filtration device with a cut-off of 10 kDa. Also the synovial fluid was subjected to ultrafiltration.

RESULTS AND CONCLUSIONS: At clinical monitoring of the 30th day, 22/58 (38 %) patients subcutaneously treated with the autologous ultrafiltrate prepared by the freeze/thawing of PBMCs reached an ACR20. Comparable results were obtained with the other two ultrafiltrates. Cell cultures The addition of ultrafiltrates to rheumatoid PBMCs cultures led to the upregulation of a marker for T-regulatory cells, and downregulation of a cell proliferation marker; changes that together have the meaning of a global immunomodulatory response and that only a specific antigen (ultrafiltrate UF-f/t) might induce in the rheumatoid patient, probably by activating pre-existing protective network. Experimental arthritis All the ultrafiltrates except that prepared by Rifamycin SV were able to modulate the adjuvant arthritis in rats. In particular, longlasting synovial fluid induced a significant reduction of the severity of subsequent arthritis ($p < 0.01$) while SF from recent RA effusion (5-10 days after a previous complete extraction) and knee osteoarthrosis were ineffective. It is reasonable to assume there are at least two unknown endosomal immunoactive epitopes; one developing its immunotherapeutic property in RA, and the other, related to the molecule of HSP60, reduces the severity of oncoming arthritis. Both

epitopes are present in humans, have a molecular weight of <=10 kDa and do not appear to be bystander antigens. Please see Additional file 1 for the abstract in Italian.

Publication Type

Journal Article.

Year of Publication

2016

<242>

Unique Identifier

27229685

Title

Antibody response to pneumococcal and influenza vaccination in patients with rheumatoid arthritis receiving abatacept.

Source

BMC Musculoskeletal Disorders. 17:231, 2016 05 26.

VI 1

Status

MEDLINE

Authors

Alten R; Bingham CO 3rd; Cohen SB; Curtis JR; Kelly S; Wong D; Genovese MC.

Authors Full Name

Alten, Rieke; Bingham, Clifton O 3rd; Cohen, Stanley B; Curtis, Jeffrey R; Kelly, Sheila; Wong, Dennis; Genovese, Mark C.

Institution

Alten, Rieke. Schlosspark-Klinik University Medicine, Berlin, Germany. rieke.alten@schlosspark-klinik.de.

Alten, Rieke. University Medicine Berlin, Berlin, 14059, Germany. rieke.alten@schlosspark-klinik.de.

Bingham, Clifton O 3rd. Johns Hopkins University, Baltimore, MD, USA.

Cohen, Stanley B. Metroplex Clinical Research Center, Dallas, TX, USA.

Curtis, Jeffrey R. University of Alabama at Birmingham, Birmingham, AL, USA.

Kelly, Sheila. Bristol-Myers Squibb, Princeton, NJ, USA.

Wong, Dennis. Bristol-Myers Squibb, Princeton, NJ, USA.

Genovese, Mark C. Stanford University, Palo Alto, CA, USA.

Abstract

BACKGROUND: Patients with rheumatoid arthritis (RA), including those treated with biologics, are at increased risk of some vaccine-preventable infections. We evaluated the antibody response to standard 23-valent pneumococcal polysaccharide vaccine (PPSV23) and the 2011-2012 trivalent seasonal influenza vaccine in adults with RA receiving subcutaneous (SC) abatacept and background disease-modifying anti-rheumatic drugs (DMARDs).

METHODS: Two multicenter, open-label sub-studies enrolled patients from the ACQUIRE (pneumococcal and influenza) and ATTUNE (pneumococcal) studies at any point during their SC abatacept treatment cycle following completion of >=3 months' SC abatacept. All patients received fixed-dose abatacept 125 mg/week with background DMARDs. A pre-vaccination blood sample was taken, and after 28 +/- 3 days a final post-vaccination sample was collected. The primary endpoint was the proportion of patients achieving an immunologic response to the vaccine at Day 28 among patients without a protective antibody level to the vaccine antigens at baseline (pneumococcal: defined as >=2-fold increase in post-vaccination titers to >=3 of 5 antigens and protective antibody level of >=1.6 mug/mL to >=3 of 5 antigens; influenza: defined as >=4-fold increase in post-vaccination titers to >=2 of 3 antigens and protective antibody level of >=1:40 to >=2 of 3 antigens). Safety and tolerability were evaluated throughout the sub-studies.

RESULTS: Pre- and post-vaccination titers were available for 113/125 and 186/191 enrolled patients receiving the PPSV23 and influenza vaccine, respectively. Among vaccinated patients, 47/113 pneumococcal and 121/186 influenza patients were without protective antibody levels at baseline. Among patients with available data, 73.9 % (34/46) and 61.3 % (73/119) met the primary endpoint and achieved an immunologic response to PPSV23 or influenza vaccine, respectively. In patients with pre- and post-vaccination data available, 83.9 % in the pneumococcal study demonstrated protective antibody levels with PPSV23 (titer >=1.6 mug/mL to >=3 of 5 antigens), and 81.2 % in the influenza study achieved protective antibody levels (titer >=1:40 to >=2 of 3 antigens) at Day 28 post-vaccination. Vaccines were well tolerated with SC abatacept with background DMARDs.

CONCLUSIONS: In these sub-studies, patients with RA receiving SC abatacept and background DMARDs were able to mount an appropriate immune response to pneumococcal and influenza vaccines.

TRIAL REGISTRATION: NCT00559585 (registered 15 November 2007) and NCT00663702 (registered 18 April 2008).

Publication Type

Clinical Trial, Phase III. Journal Article. Multicenter Study. Randomized Controlled Trial. Research Support, Non-U.S. Gov't.

Year of Publication

2016

<243>

Unique Identifier

27228631

Title

Autoimmune/Inflammatory Syndrome Induced by Adjuvants and Sjogren's Syndrome.

Source

Israel Medical Association Journal: Imaj. 18(3-4):150-3, 2016 Mar-Apr.

VI 1

Status

MEDLINE

Authors

Colafrancesco S; Perricone C; Shoenfeld Y.

Authors Full Name

Colafrancesco, Serena; Perricone, Carlo; Shoenfeld, Yehuda.

Abstract

Sjogren's syndrome (SS), a chronic systemic autoimmune inflammatory condition involving the exocrine glands, has been suggested to be part of the spectrum of the Autoimmune/ inflammatory Syndrome Induced by Adjuvants (ASIA). ASIA incorporates an umbrella of clinical conditions including siliconosis, macrophage myofasciitis syndrome, and post-vaccination phenomena that occur after the exposure to a substance, namely the adjuvant. Interestingly, SS and ASIA share several common features. Firstly, a shared pathogenic mechanism involving a disruption of the immune system balance, with B cell proliferation, cytokine production and tissue infiltration, has been proposed. Patients with ASIA often present clinical features resembling those of SS; dry mouth and dry eyes have also been included in the proposed classification criteria for ASIA. Finally, several case reports have suggested that both vaccines and silicone may trigger the

development of SS. Unveiling these common pathways will contribute considerably to our understanding and management of both conditions.

Publication Type

Journal Article.

Year of Publication

2016

<244>

Unique Identifier

27206918

Title

Optimization of Folate-Targeted Immunotherapy for the Treatment of Experimental Arthritis.

Source

Inflammation. 39(4):1345-53, 2016 Aug.

VI 1

Status

MEDLINE

Authors

Varghese B; Paulos C; Low PS.

Authors Full Name

Varghese, Bindu; Paulos, Chrystal; Low, Philip S.

Institution

Varghese, Bindu. Department of Chemistry, Purdue University, 720 Clinic Drive, West Lafayette, IN, 47907-2084, USA.

Paulos, Chrystal. Department of Chemistry, Purdue University, 720 Clinic Drive, West Lafayette, IN, 47907-2084, USA.

Low, Philip S. Department of Chemistry, Purdue University, 720 Clinic Drive, West Lafayette, IN, 47907-2084, USA. plow@purdue.edu.

Abstract

Folate-targeted immunotherapy constitutes a powerful method for the treatment of established arthritis in multiple animal models of the disease. The therapy involves immunization of the animal against a hapten to induce anti-hapten antibodies, followed by injection with a folate-hapten conjugate to decorate the surface of folate receptor-positive (activated) macrophages with

the antigenic hapten. The hapten-marked macrophages are then recognized by the anti-hapten antibodies and eliminated by immune mechanisms, leading to attenuation of disease symptoms. In the following paper, we optimize the therapy for elimination of inflammatory macrophages and suppression of rheumatoid arthritis symptoms. We also demonstrate a tight correlation between folate receptor-positive macrophage abundance in the liver and inflammation of affected joints. The results suggest that therapies that reduce folate receptor-positive macrophage populations in the body should constitute effective treatments for rheumatoid arthritis.

Publication Type

Journal Article.

Year of Publication

2016

<245>

Unique Identifier

27192568

Title

Co-stimulatory and Co-inhibitory Pathways in Autoimmunity. [Review]

Source

Immunity. 44(5):1034-51, 2016 05 17.

VI 1

Status

MEDLINE

Authors

Zhang Q; Vignali DA.

Authors Full Name

Zhang, Qianxia; Vignali, Dario A A.

Institution

Zhang, Qianxia. Department of Immunology, University of Pittsburgh School of Medicine, Pittsburgh, PA 15261, USA.

Vignali, Dario A A. Department of Immunology, University of Pittsburgh School of Medicine, Pittsburgh, PA 15261, USA; Tumor Microenvironment Center, University of Pittsburgh Cancer Institute, Pittsburgh, PA 15232, USA. Electronic address: dvignali@pitt.edu.

Abstract

The immune system is guided by a series of checks and balances, a major component of which is a large array of co-stimulatory and co-inhibitory pathways that modulate the host response. Although co-stimulation is essential for boosting and shaping the initial response following signaling through the antigen receptor, inhibitory pathways are also critical for modulating the immune response. Excessive co-stimulation and/or insufficient co-inhibition can lead to a breakdown of self-tolerance and thus to autoimmunity. In this review, we will focus on the role of co-stimulatory and co-inhibitory pathways in two systemic (systemic lupus erythematosus and rheumatoid arthritis) and two organ-specific (multiple sclerosis and type 1 diabetes) emblematic autoimmune diseases. We will also discuss how mechanistic analysis of these pathways has led to the identification of potential therapeutic targets and initiation of clinical trials for autoimmune diseases, as well as outline some of the challenges that lie ahead. Copyright © 2016 Elsevier Inc. All rights reserved.

Publication Type

Journal Article. Review. Research Support, N.I.H., Extramural.

Year of Publication

2016

<246>

Unique Identifier

27170517

Title

Treatment of rheumatoid arthritis during pregnancy: present and future. [Review]

Source

Expert Review of Clinical Immunology. 12(9):937-44, 2016 Sep.

VI 1

Status

MEDLINE

Authors

Forger F; Villiger PM.

Authors Full Name

Forger, Frauke; Villiger, Peter M.

Institution

Forger, Frauke. a Department of Rheumatology, Immunology and Allergology, University Hospital, and University of Bern, Bern, Switzerland.
Villiger, Peter M. a Department of Rheumatology, Immunology and Allergology, University Hospital, and University of Bern, Bern, Switzerland.
Abstract
INTRODUCTION: For the management of rheumatoid arthritis patients who plan to become pregnant, both disease activity and therapeutic regimens have to be taken into consideration. In the case of stable inactive disease, pregnancy can be planned and therapy can be adjusted with drugs compatible with pregnancy.

AREAS COVERED: Drugs to be discontinued before pregnancy are methotrexate, leflunomide, tocilizumab, rituximab, abatacept and tofacitinib. Pregnancy compatible disease modifying drugs are antimalarial drugs and sulfasalazine. TNF-inhibitors can be continued during the first half of pregnancy, yet if indicated during the third trimester TNF-inhibitors with a low rate of transplacental passage should be used. Glucocorticoids may be considered at the lowest effective dose throughout pregnancy. Non-selective COX-inhibitors can be continued until gestational week 32. Expert commentary: Together, a tailored treatment throughout pregnancy is possible with reasonable safety. Controlling disease activity during pregnancy is important for both, maternal and fetal health.
Publication Type
Journal Article. Review.
Year of Publication
2016

<247>
Unique Identifier
27119134
Title
[Multicenter study. Early summer meningoencephalitis: an added dose for patients with rheumatoid arthritis?]. [German]
Source
MMW Fortschritte der Medizin. 158(5):16, 2016 Mar 17.
VI 1

Status

MEDLINE

Authors

Anonymous.

Publication Type

Journal Article.

Year of Publication

2016

<248>

Unique Identifier

27100817

Title

Autoantibodies and pain. [Review]

Source

Current Opinion in Supportive & Palliative Care. 10(2):137-42, 2016 06.

VI 1

Status

MEDLINE

Authors

Dawes JM; Vincent A.

Authors Full Name

Dawes, John M; Vincent, Angela.

Institution

Dawes, John M. Nuffield Department of Clinical Neurosciences, John Radcliffe Hospital, Oxford, UK.

Abstract

PURPOSE OF REVIEW: Over the last 20 years, several neurological conditions have been identified which appear to be caused directly by autoantibodies targeting receptors, ion channels and related proteins on neuronal or glial cells. Neuroimmune interactions are now accepted contributors to chronic pain conditions. Autoantibodies might be one such cause and here we highlight their potential role in pathological pain.

RECENT FINDINGS: Recent studies have given more weight to the idea that autoantibodies can be directly related to pain; this is suggested by the success of immunotherapy in patients and preclinical studies in animal models. For example, in complex regional pain syndrome, plasma exchange or intravenous immunoglobulins have been successful in reducing pain scores. Similarly, immunotherapies reduce autoantibody levels and pain in neuromyelitis optica and voltage-gated potassium channel complex antibody positive patients. Furthermore, animal studies show that IgG autoantibodies from patients with rheumatoid arthritis or complex regional pain syndrome can recapitulate pain phenotypes in mice.

SUMMARY: There is growing evidence that some pain syndromes may be caused by autoantibodies to proteins that modify or exacerbate pain sensation. This has potentially direct therapeutic advantages for these patients and possible wider implications for sufferers of chronic pain more generally.

Publication Type

Journal Article. Review. Research Support, Non-U.S. Gov't.

Year of Publication

2016

<249>

Unique Identifier

27098309

Title

Ureido group-specific antibodies are induced in rabbits immunized with citrulline- or homocitrulline-containing antigens.

Source

Autoimmunity. 49(7):459-465, 2016 11.

VI 1

Status

MEDLINE

Authors

Turunen S; Koivula MK; Risteli L; Risteli J.

Authors Full Name

Turunen, Sanna; Koivula, Marja-Kaisa; Risteli, Leila; Risteli, Juha.

Institution
Turunen, Sanna. a Department of Clinical Chemistry, Cancer and Translational Medicine Research Unit, Medical Research Center, University of Oulu, Oulu, Finland and.
Koivula, Marja-Kaisa. a Department of Clinical Chemistry, Cancer and Translational Medicine Research Unit, Medical Research Center, University of Oulu, Oulu, Finland and.
Koivula, Marja-Kaisa. b Northern Finland Laboratory Centre NordLab, Oulu University Hospital, Oulu, Finland.
Risteli, Leila. a Department of Clinical Chemistry, Cancer and Translational Medicine Research Unit, Medical Research Center, University of Oulu, Oulu, Finland and.
Risteli, Leila. b Northern Finland Laboratory Centre NordLab, Oulu University Hospital, Oulu, Finland.
Risteli, Juha. a Department of Clinical Chemistry, Cancer and Translational Medicine Research Unit, Medical Research Center, University of Oulu, Oulu, Finland and.
Risteli, Juha. b Northern Finland Laboratory Centre NordLab, Oulu University Hospital, Oulu, Finland.
Abstract
The specificities and cross-reactions of antibodies induced by citrulline- and homocitrulline-containing proteins may give implications on the role of citrulline- and homocitrulline-binding antibodies in the pathogenesis and progression of rheumatoid arthritis (RA). Here we use rabbits as an experimental model of antibody development in RA. Thirty-two animals were immunized with peptide antigens containing either homocitrulline or citrulline. The sera were tested for binding to CCP and MCV antigens and to peptide sequences related to carboxyterminal telopeptides of type I and II collagens and containing arginine, citrulline, or homocitrulline. The binding of CCP and MCV antigens to antisera against homocitrulline-containing immunogens could be inhibited by human serum albumin containing homocitrulline, whereas similar binding to sera against citrulline-containing immunogens was not inhibited. The antisera induced with citrulline-containing collagen telopeptides recognized type I collagen-related antigens in a sequence-specific manner, as antibody binding to both citrulline- and homocitrulline-containing peptides was inhibited by corresponding citrullinated and native peptides. In contrast, type II collagen-related peptides were recognized by the antisera in a ureido group-specific manner, as their binding to homocitrulline-containing peptide was inhibited by both citrulline- and homocitrulline-containing, but not native peptide. Binding of the citrullinated type II collagen peptide could only be inhibited by the similarly citrullinated peptide. In conclusion, antibodies induced with citrulline or homocitrulline-containing antigens bound antigens in a ureido group-specific manner, recognizing citrulline and homocitrulline also in other sequences than those used in the original immunization. In competitive situations the amino acid present in the immunization antigen was favored.

Publication Type
  Journal Article. Research Support, Non-U.S. Gov't.
Year of Publication
  2016

<250>
Unique Identifier
  27096429
Title
  Influenza and Pneumococcal Vaccination Uptake in Patients with Rheumatoid Arthritis Treated with Immunosuppressive Therapy in the UK: A Retrospective Cohort Study Using Data from the Clinical Practice Research Datalink.
Source
  PLoS ONE [Electronic Resource]. 11(4):e0153848, 2016.
VI 1
Status
  MEDLINE
Authors
  Costello R; Winthrop KL; Pye SR; Brown B; Dixon WG.
Author NameID
  Costello, Ruth; ORCID: http://orcid.org/0000-0003-2709-6666
Authors Full Name
  Costello, Ruth; Winthrop, Kevin L; Pye, Stephen R; Brown, Benjamin; Dixon, William G.
Institution
  Costello, Ruth. Arthritis Research UK Centre for Epidemiology, Centre for Musculoskeletal Research, Institute for Inflammation and Repair, Manchester Academic Health Science Centre, The University of Manchester, Manchester, United Kingdom.
  Winthrop, Kevin L. Division of Infectious Diseases, Oregon Health and Science University, Portland, Oregon, United States of America.
  Pye, Stephen R. Arthritis Research UK Centre for Epidemiology, Centre for Musculoskeletal Research, Institute for Inflammation and Repair, Manchester Academic Health Science Centre, The University of Manchester, Manchester, United Kingdom.

Brown, Benjamin. Health eResearch Centre, Manchester Academic Health Science Centre, The University of Manchester, Manchester, United Kingdom.
Dixon, William G. Arthritis Research UK Centre for Epidemiology, Centre for Musculoskeletal Research, Institute for Inflammation and Repair, Manchester Academic Health Science Centre, The University of Manchester, Manchester, United Kingdom.
Dixon, William G. Health eResearch Centre, Manchester Academic Health Science Centre, The University of Manchester, Manchester, United Kingdom.
Abstract
INTRODUCTION: Guidelines for the management of rheumatoid arthritis (RA) recommend using influenza and pneumococcal vaccinations to mitigate infection risk. The level of adherence to these guidelines is not well known in the UK. The aims of this study were to describe the uptake of influenza and pneumococcal vaccinations in patients with RA in the UK, to compare the characteristics of those vaccinated to those not vaccinated and to compare vaccination rates across regions of the UK.

METHODS: A retrospective cohort study of adults diagnosed with incident RA and treated with non-biologic immunosuppressive therapy, using data from a large primary care database. For the influenza vaccination, patients were considered unvaccinated on 1st September each year and upon vaccination their status changed to vaccinated. For pneumococcal vaccination, patients were considered vaccinated after their first vaccination until the end of follow-up. Patients were stratified by age 65 at the start of follow-up, given differences in vaccination guidelines for the general population.

RESULTS: Overall (N=15,724), 80% patients received at least one influenza vaccination, and 50% patients received a pneumococcal vaccination, during follow-up (mean 5.3 years). Of those aged below 65 years (N=9,969), 73% patients had received at least one influenza vaccination, and 43% patients received at least one pneumococcal vaccination. Of those aged over 65 years (N=5,755), 91% patients received at least one influenza vaccination, and 61% patients had received at least one pneumococcal vaccination. Those vaccinated were older, had more comorbidity and visited the GP more often. Regional differences in vaccination rates were seen with the highest rates in Northern Ireland, and the lowest rates in London.

CONCLUSIONS: One in five patients received no influenza vaccinations and one in two patients received no pneumonia vaccine over five years of follow-up. There remains significant scope to improve uptake of vaccinations in patients with RA.
Publication Type
Journal Article. Research Support, Non-U.S. Gov't.

Year of Publication
  2016

<251>
Unique Identifier
  27084914
Title
  A Multifaceted Intervention to Improve Influenza, Pneumococcal, and Herpes Zoster Vaccination among Patients with Rheumatoid Arthritis.
Source
  Journal of Rheumatology. 43(6):1030-7, 2016 06.
VI 1
Status
  MEDLINE
Authors
  Baker DW; Brown T; Lee JY; Ozanich A; Liss DT; Sandler DS; Ruderman EM.
Authors Full Name
  Baker, David W; Brown, Tiffany; Lee, Ji Young; Ozanich, Amanda; Liss, David T; Sandler, Diana S; Ruderman, Eric M.
Institution
  Baker, David W. From the Division of General Internal Medicine and Geriatrics, and Division of Rheumatology, Northwestern University Feinberg School of Medicine, Chicago, Illinois, USA.D.W. Baker, MD, MPH, Division of General Internal Medicine and Geriatrics, Northwestern University Feinberg School of Medicine; T. Brown, MPH, Division of General Internal Medicine and Geriatrics, Northwestern University Feinberg School of Medicine; J.Y. Lee, MS, Division of General Internal Medicine and Geriatrics, Northwestern University Feinberg School of Medicine; A. Ozanich, PA, Division of Rheumatology, Northwestern University Feinberg School of Medicine; D.T. Liss, PhD, Division of General Internal Medicine and Geriatrics, Northwestern University Feinberg School of Medicine; D.S. Sandler, MD, Division of Rheumatology, Northwestern University Feinberg School of Medicine; E.M. Ruderman, MD, Professor of Medicine, Division of Rheumatology, Northwestern University Feinberg School of Medicine.
   Brown, Tiffany. From the Division of General Internal Medicine and Geriatrics, and Division of Rheumatology, Northwestern University Feinberg School of Medicine, Chicago, Illinois,

USA.D.W. Baker, MD, MPH, Division of General Internal Medicine and Geriatrics, Northwestern University Feinberg School of Medicine; T. Brown, MPH, Division of General Internal Medicine and Geriatrics, Northwestern University Feinberg School of Medicine; J.Y. Lee, MS, Division of General Internal Medicine and Geriatrics, Northwestern University Feinberg School of Medicine; A. Ozanich, PA, Division of Rheumatology, Northwestern University Feinberg School of Medicine; D.T. Liss, PhD, Division of General Internal Medicine and Geriatrics, Northwestern University Feinberg School of Medicine; D.S. Sandler, MD, Division of Rheumatology, Northwestern University Feinberg School of Medicine; E.M. Ruderman, MD, Professor of Medicine, Division of Rheumatology, Northwestern University Feinberg School of Medicine.

Lee, Ji Young. From the Division of General Internal Medicine and Geriatrics, and Division of Rheumatology, Northwestern University Feinberg School of Medicine, Chicago, Illinois, USA.D.W. Baker, MD, MPH, Division of General Internal Medicine and Geriatrics, Northwestern University Feinberg School of Medicine; T. Brown, MPH, Division of General Internal Medicine and Geriatrics, Northwestern University Feinberg School of Medicine; J.Y. Lee, MS, Division of General Internal Medicine and Geriatrics, Northwestern University Feinberg School of Medicine; A. Ozanich, PA, Division of Rheumatology, Northwestern University Feinberg School of Medicine; D.T. Liss, PhD, Division of General Internal Medicine and Geriatrics, Northwestern University Feinberg School of Medicine; D.S. Sandler, MD, Division of Rheumatology, Northwestern University Feinberg School of Medicine; E.M. Ruderman, MD, Professor of Medicine, Division of Rheumatology, Northwestern University Feinberg School of Medicine.

Ozanich, Amanda. From the Division of General Internal Medicine and Geriatrics, and Division of Rheumatology, Northwestern University Feinberg School of Medicine, Chicago, Illinois, USA.D.W. Baker, MD, MPH, Division of General Internal Medicine and Geriatrics, Northwestern University Feinberg School of Medicine; T. Brown, MPH, Division of General Internal Medicine and Geriatrics, Northwestern University Feinberg School of Medicine; J.Y. Lee, MS, Division of General Internal Medicine and Geriatrics, Northwestern University Feinberg School of Medicine; A. Ozanich, PA, Division of Rheumatology, Northwestern University Feinberg School of Medicine; D.T. Liss, PhD, Division of General Internal Medicine and Geriatrics, Northwestern University Feinberg School of Medicine; D.S. Sandler, MD, Division of Rheumatology, Northwestern University Feinberg School of Medicine; E.M. Ruderman, MD, Professor of Medicine, Division of Rheumatology, Northwestern University Feinberg School of Medicine.

Liss, David T. From the Division of General Internal Medicine and Geriatrics, and Division of Rheumatology, Northwestern University Feinberg School of Medicine, Chicago, Illinois, USA.D.W. Baker, MD, MPH, Division of General Internal Medicine and Geriatrics, Northwestern University Feinberg School of Medicine; T. Brown, MPH, Division of General Internal Medicine and Geriatrics, Northwestern University Feinberg School of Medicine; J.Y. Lee, MS, Division of General Internal Medicine and Geriatrics, Northwestern University Feinberg School of Medicine;

A. Ozanich, PA, Division of Rheumatology, Northwestern University Feinberg School of Medicine; D.T. Liss, PhD, Division of General Internal Medicine and Geriatrics, Northwestern University Feinberg School of Medicine; D.S. Sandler, MD, Division of Rheumatology, Northwestern University Feinberg School of Medicine; E.M. Ruderman, MD, Professor of Medicine, Division of Rheumatology, Northwestern University Feinberg School of Medicine.

Sandler, Diana S. From the Division of General Internal Medicine and Geriatrics, and Division of Rheumatology, Northwestern University Feinberg School of Medicine, Chicago, Illinois, USA.D.W. Baker, MD, MPH, Division of General Internal Medicine and Geriatrics, Northwestern University Feinberg School of Medicine; T. Brown, MPH, Division of General Internal Medicine and Geriatrics, Northwestern University Feinberg School of Medicine; J.Y. Lee, MS, Division of General Internal Medicine and Geriatrics, Northwestern University Feinberg School of Medicine; A. Ozanich, PA, Division of Rheumatology, Northwestern University Feinberg School of Medicine; D.T. Liss, PhD, Division of General Internal Medicine and Geriatrics, Northwestern University Feinberg School of Medicine; D.S. Sandler, MD, Division of Rheumatology, Northwestern University Feinberg School of Medicine; E.M. Ruderman, MD, Professor of Medicine, Division of Rheumatology, Northwestern University Feinberg School of Medicine.

Ruderman, Eric M. From the Division of General Internal Medicine and Geriatrics, and Division of Rheumatology, Northwestern University Feinberg School of Medicine, Chicago, Illinois, USA.D.W. Baker, MD, MPH, Division of General Internal Medicine and Geriatrics, Northwestern University Feinberg School of Medicine; T. Brown, MPH, Division of General Internal Medicine and Geriatrics, Northwestern University Feinberg School of Medicine; J.Y. Lee, MS, Division of General Internal Medicine and Geriatrics, Northwestern University Feinberg School of Medicine; A. Ozanich, PA, Division of Rheumatology, Northwestern University Feinberg School of Medicine; D.T. Liss, PhD, Division of General Internal Medicine and Geriatrics, Northwestern University Feinberg School of Medicine; D.S. Sandler, MD, Division of Rheumatology, Northwestern University Feinberg School of Medicine; E.M. Ruderman, MD, Professor of Medicine, Division of Rheumatology, Northwestern University Feinberg School of Medicine. e-ruderman@northwestern.edu.

Abstract

OBJECTIVE: Vaccination rates for influenza, pneumococcus, and zoster in patients with rheumatoid arthritis (RA) have remained low. Simple electronic or paper reminders have produced only small increases in vaccination rates. We sought to identify a more effective approach to improve vaccination rates.

METHODS: We conducted a system-level intervention at an academic rheumatology clinic that included electronic reminders with linked order sets, physician auditing and feedback, patient outreach, and optional printed prescriptions for zoster vaccination at an outside pharmacy.

RESULTS: We targeted 1255 eligible patients with RA. There was no change in patients' self-reported influenza vaccination rates, although the baseline self-reported rate was already high and much higher than that documented in the electronic health record. Pneumococcal vaccination rates increased from 28.7% to 45.8%; in regression analysis, the rate of change in pneumococcal vaccination increased by 9.4% per year above baseline trends (95% CI 3.9-15.5, p = 0.002). The rate of zoster vaccination increased from 2.5% to 4.5% overall (p = 0.01) and from 3.0% to 6.6% among patients not receiving biologic therapy that precluded zoster vaccination.

CONCLUSION: Although the intervention improved pneumococcal and zoster vaccination rates, the improvement in pneumococcal vaccination rate was less than expected, and the zoster vaccination rate remained low even for ideal candidates. Likely barriers include lack of familiarity and difficulty using electronic reminders and order sets, uncertainty about the value and safety of recommended vaccines, and uncertainty about patients' insurance coverage and prior vaccination history. Future interventions should include strategies to address these.

Publication Type

Journal Article. Research Support, N.I.H., Extramural. Research Support, Non-U.S. Gov't.

Year of Publication

2016

<252>

Unique Identifier

27079756

Title

Recent advances in the diagnosis and treatment of polymyalgia rheumatica. [Review]

Source

Expert Review of Clinical Immunology. 12(10):1037-45, 2016 Oct.

VI 1

Status

MEDLINE

Authors

Muratore F; Pazzola G; Pipitone N; Salvarani C.

Authors Full Name

Muratore, Francesco; Pazzola, Giulia; Pipitone, Nicolo; Salvarani, Carlo.
Institution
Muratore, Francesco. a Rheumatology Unit, Department of Internal Medicine, Azienda Ospedaliera ASMN, Istituto di Ricovero e Cura a Carattere Scientifico, Reggio Emilia, Italy.
Pazzola, Giulia. a Rheumatology Unit, Department of Internal Medicine, Azienda Ospedaliera ASMN, Istituto di Ricovero e Cura a Carattere Scientifico, Reggio Emilia, Italy.
Pipitone, Nicolo. a Rheumatology Unit, Department of Internal Medicine, Azienda Ospedaliera ASMN, Istituto di Ricovero e Cura a Carattere Scientifico, Reggio Emilia, Italy.
Salvarani, Carlo. a Rheumatology Unit, Department of Internal Medicine, Azienda Ospedaliera ASMN, Istituto di Ricovero e Cura a Carattere Scientifico, Reggio Emilia, Italy.
Abstract
INTRODUCTION: Polymyalgia rheumatica is one of the most common rheumatic inflammatory disorders in people older than 50 years characterized by aching and prolonged morning stiffness in the shoulder and pelvic girdle and neck..

AREAS COVERED: In this review, we will focus on recent advances on the diagnosis and management of PMR. Expert commentary: Controversy exist whether PMR represent a single entity disease or is an umbrella term that comprises a clinical presentation common to a range of related conditions (polymyalgic syndrome). To date there are no specific diagnostic tests, and the diagnosis remains clinical, although ultrasonography, positron emission tomography scan and the recent ACR/EULAR classification criteria may help to confirm the clinical diagnosis. A step-wise process for the diagnosis of PMR has been proposed. Low-dose steroids are highly effective in the majority of patients and remain the mainstay of treatment, but relapses occur in about 50% of patients and glucocorticoid related adverse event are common. The steroid sparing effects of the immunosuppressive treatment evaluated to date are unclear.
Publication Type
Journal Article. Review.
Year of Publication
2016

<253>
Unique Identifier
27055777

Title

B cell depletion with rituximab in patients with rheumatoid arthritis: Multiplex bead array reveals the kinetics of IgG and IgA antibodies to citrullinated antigens.

Source

Journal of Autoimmunity. 70:22-30, 2016 06.

VI 1

Status

MEDLINE

Authors

Cambridge G; Leandro MJ; Lahey LJ; Fairhead T; Robinson WH; Sokolove J.

Authors Full Name

Cambridge, Geraldine; Leandro, Maria J; Lahey, Lauren J; Fairhead, Thomas; Robinson, William H; Sokolove, Jeremy.

Institution

Cambridge, Geraldine. Department of Rheumatology, University College London, UK. Electronic address: g.cambridge@ucl.ac.uk.

Leandro, Maria J. Department of Rheumatology, University College London, UK.

Lahey, Lauren J. VA Palo Alto Healthcare System and Stanford University, USA.

Fairhead, Thomas. Department of Rheumatology, University College London, UK.

Robinson, William H. VA Palo Alto Healthcare System and Stanford University, USA.

Sokolove, Jeremy. VA Palo Alto Healthcare System and Stanford University, USA.

Abstract

The serology of patients with Rheumatoid arthritis (RA) is characterized by persistently raised levels of autoantibodies: Rheumatoid Factors (RhF) against Fc of IgG, and to citrullinated (Cit) protein/peptide sequences: ACPA, recognizing multiple Cit-sequences. B cell depletion therapy based on rituximab delivers good clinical responses in RA patients, particularly in the seropositive group, with responses sometimes lasting beyond the phase of B cell reconstitution. In general, ACPA levels fall following rituximab, but fluctuations with respect to predicting relapse have proved disappointing. In order to identify possible immunodominant specificities within either IgG- or IgA-ACPA we used a Multiplex bead-based array consisting of 30 Cit-peptides/proteins and 22 corresponding native sequences. The kinetics of the serum ACPA response to individual specificities was measured at key points (Baseline, B cell depletion phase, Relapse) within an initial cycle of rituximab therapy in 16 consecutive patients with severe, active RA. All had achieved significant decreases in Disease Activity Scores-28 and maintained B cell depletion in the peripheral blood (<5 CD19+cells/mul) for at least 3 months. At Baseline, mean fluorescence intensity shown by individual IgG- and IgA-ACPA were strongly correlated ($R(2) = 0.75$; $p < 0.0001$) but IgA-ACPA were approximately 10-fold lower. Data were Z-normalised in order to

compare serial results and antibody classes. At Baseline, a total of 68 IgG- and 51 IgA-ACPA had Z-scores >= 1 (above population mean) were identified, with at least one Cit-antigen identified in each serum. ACPA to individual specificities subsequently fluctuated with 3 different patterns. Most 51/68 (75%) IgG- and 48/51 IgA-ACPA (94%) fell between Baseline and Depletion, of which 57% IgG- and 65% IgA-ACPA rebounded pre-Relapse. Interestingly, 17/68 IgG-ACPA (25%) and some IgA-ACPA (3/51; 6%) transiently increased from Baseline, subsequently falling pre-Relapse. Individual responses to particular Cit-epitopes were not linked to particular patterns of fluctuation, but IgG- and IgA-ACPA to individual Cit-antigens often followed similar courses. Some new IgG- and IgA-ACPA, generally to different Cit-antigens however, arose at Relapse in 4 patients. The complexities of the ACPA response after rituximab may therefore reflect its ability to deplete or modify the function of parent B cell clones, which varies between patients. Although relapse following rituximab invariably follows naive B cell exit from the bone marrow, these studies show that interactions between both 'new' and residual autoreactive memory B cells may be key to resumption of symptoms. The lack of identification of any immunodominant specificity suggests that the process of citrullination, rather than any particular Cit-antigen drives the autoimmune response in RA patients. Copyright © 2016 Elsevier Ltd. All rights reserved.

Publication Type

Journal Article. Research Support, N.I.H., Extramural. Research Support, Non-U.S. Gov't.

Year of Publication

2016

<254>

Unique Identifier

27029006

Title

A Personalized Approach to Biological Therapy Using Prediction of Clinical Response Based on MRP8/14 Serum Complex Levels in Rheumatoid Arthritis Patients.

Source

PLoS ONE [Electronic Resource]. 11(3):e0152362, 2016.

VI 1

Status

MEDLINE

Authors

Nair SC; Welsing PM; Choi IY; Roth J; Holzinger D; Bijlsma JW; van Laar JM; Gerlag DM; Lafeber FP; Tak PP.
Authors Full Name
Nair, S C; Welsing, P M J; Choi, I Y K; Roth, J; Holzinger, D; Bijlsma, J W J; van Laar, J M; Gerlag, D M; Lafeber, F P J G; Tak, P P.
Institution
Nair, S C. Department of Rheumatology and Clinical Immunology, University Medical Center, Utrecht, The Netherlands.
Welsing, P M J. Department of Rheumatology and Clinical Immunology, University Medical Center, Utrecht, The Netherlands.
Choi, I Y K. Division of Clinical Immunology and Rheumatology, Academic Medical Center/University of Amsterdam, Amsterdam, the Netherlands.
Roth, J. Institute of Immunology, University Hospital Muenster, Muenster, Germany.
Holzinger, D. Department of Pediatric Rheumatology and Immunology, University Children's Hospital Muenster, Muenster, Germany.
Bijlsma, J W J. Department of Rheumatology and Clinical Immunology, University Medical Center, Utrecht, The Netherlands.
van Laar, J M. Department of Rheumatology and Clinical Immunology, University Medical Center, Utrecht, The Netherlands.
Gerlag, D M. Division of Clinical Immunology and Rheumatology, Academic Medical Center/University of Amsterdam, Amsterdam, the Netherlands.
Lafeber, F P J G. Department of Rheumatology and Clinical Immunology, University Medical Center, Utrecht, The Netherlands.
Tak, P P. Division of Clinical Immunology and Rheumatology, Academic Medical Center/University of Amsterdam, Amsterdam, the Netherlands.
Abstract
OBJECTIVES: Measurement of MRP8/14 serum levels has shown potential in predicting clinical response to different biological agents in rheumatoid arthritis (RA). We aimed to develop a treatment algorithm based on a prediction score using MRP8/14 measurements and clinical parameters predictive for response to different biological agents.

METHODS: Baseline serum levels of MRP8/14 were measured in 170 patients starting treatment with infliximab, adalimumab or rituximab. We used logistic regression analysis to develop a predictive score for clinical response at 16 weeks. MRP8/14 levels along with clinical variables at baseline were investigated. We also investigated how the predictive effect of MRP8/14 was modified by drug type. A treatment algorithm was developed based on categorizing the expected response per drug type as high, intermediate or low for each patient and optimal

treatment was defined. Finally, we present the utility of using this treatment algorithm in clinical practice.

RESULTS: The probability of response increased with higher baseline MRP8/14 complex levels (OR = 1.39), differentially between the TNF-blockers and rituximab (OR of interaction term = 0.78), and also increased with higher DAS28 at baseline (OR = 1.28). Rheumatoid factor positivity, functional disability (a higher HAQ), and previous use of a TNF-inhibitor decreased the probability of response. Based on the treatment algorithm 80 patients would have been recommended for anti-TNF treatment, 8 for rituximab, 13 for another biological treatment (other than TNFi or rituximab) and for 69 no recommendation was made. The predicted response rates matched the observed response in the cohort well. On group level the predicted response based on the algorithm resulted in a modest 10% higher response rate in our cohort with much higher differences in response probability in individual patients treated contrary to treatment recommendation.

CONCLUSIONS: Prediction of response using MRP8/14 levels along with clinical predictors has potential in personalizing treatment for RA patients starting biological anti-rheumatic treatment, and might increase cost-effectiveness.

Publication Type

Journal Article. Research Support, Non-U.S. Gov't.

Year of Publication

2016

<255>

Unique Identifier

27027813

Title

A new window of opportunity in rheumatoid arthritis: targeting at-risk individuals. [Review]

Source

Current Opinion in Rheumatology. 28(3):260-6, 2016 May.

VI 1

Status

MEDLINE

Authors

Mankia K; Emery P.

Authors Full Name

Mankia, Kulveer; Emery, Paul.

Institution

Mankia, Kulveer. aLeeds Institute of Rheumatic and Musculoskeletal MedicinebLeeds Musculoskeletal Biomedical Research Unit, Leeds, UK.

Abstract

PURPOSE OF REVIEW: Progress in our understanding of the preclinical events in rheumatoid arthritis (RA) has provided important insights into disease pathogenesis. Studying prospective cohorts of individuals at risk for RA development offers the opportunity to accurately characterize the sequence of events in preclinical disease as well as quantify the risk of different preclinical phenotypes. These data may provide the basis for preventive strategies in RA.

RECENT FINDINGS: RA-related systemic autoimmunity and inflammation occur long before clinical arthritis. There is growing evidence that initiating events may occur at mucosal surfaces including the periodontium, lung and gut and may be influenced by the local microbiome. For potential preventive strategies to be feasible, it is important that individuals at high risk for RA development can be readily identified from the general population. To this end, studying multiple biomarkers in prospective cohorts of at-risk individuals enables risk prediction in different at-risk phenotypes. RA prevention using immunomodulation is currently being investigated in individuals at high risk of RA development.

SUMMARY: The prospective study of at-risk individuals can provide invaluable aetiological insights as well as facilitating accurate risk prediction data. In this way, high-risk individuals may be identified for preventive interventions.

Publication Type

Journal Article. Review.

Year of Publication

2016

<256>

Unique Identifier

27023021

Title

Understanding vaccination rates and attitudes among patients with rheumatoid arthritis.

Source

American Journal of Managed Care. 22(3):161-7, 2016 Mar.

VI 1

Status

MEDLINE

Authors

Sandler DS; Ruderman EM; Brown T; Lee JY; Mixon A; Liss DT; Baker DW.

Authors Full Name

Sandler, Diana S; Ruderman, Eric M; Brown, Tiffany; Lee, Ji Young; Mixon, Amanda; Liss, David T; Baker, David W.

Institution

Ruderman, Eric M. Division of Rheumatology, Northwestern University Feinberg School of Medicine, 240 E Huron M300, Chicago, IL 60611. E-mail: e-ruderman@northwestern.edu.

Abstract

OBJECTIVES: Appropriate vaccinations are important for patients with rheumatoid arthritis (RA), who are often treated with highly immunosuppressive therapies that increase their risk of infection. However, rates of vaccination among patients with RA are below optimal levels.

STUDY DESIGN: We conducted a patient survey to assess self-reported vaccination status and to compare that status with electronic health record (EHR) data.

METHODS: We recruited randomly selected patients with RA in an academic practice in 2013. Eligible participants had a diagnosis of RA, at least 1 visit to a rheumatology clinic in each of the previous 2 years, were 18 years or older, and had English listed as their preferred language. The survey included the following domains: a) patient self-reported receipt of influenza, pneumococcal (PNVX), and herpes zoster (HZVX) vaccinations; b) attitudes about these vaccines, including reasons for unvaccinated status, if applicable; and c) provider recommendations about these vaccines.

RESULTS: Based on participants' self-report, we found a high vaccination rate for influenza during the previous season (79.4%), a moderate rate of any previous vaccination for pneumococcus (53.9%), and a very low rate of any previous vaccination for herpes zoster (7.8%). If we assume that all self-reports are accurate and we include vaccinations recorded in the EHR

that were not reported by patients, the vaccination rates were approximately 8% to 9% higher for PNVX and HZVX.

CONCLUSIONS: Vaccination rates are low among patients with RA based on self-report data. Further research is needed to investigate system-level barriers to vaccination and the impact of evidence-based, provider-level interventions on vaccination rates.

Publication Type

Comparative Study. Journal Article. Research Support, N.I.H., Extramural. Research Support, Non-U.S. Gov't.

Year of Publication

2016

<257>

Unique Identifier

27001055

Title

[Current therapy of polyarticular forms of juvenile idiopathic arthritis]. [German]

Source

Zeitschrift fur Rheumatologie. 75(3):284-91, 2016 Apr.

VI 1

Status

MEDLINE

Authors

Hospach A; Ruhlmann JM; Weller-Heinemann F.

Authors Full Name

Hospach, A; Ruhlmann, J M; Weller-Heinemann, F.

Institution

Hospach, A. Zentrum fur Padiatrische Rheumatologie, Klinikum Stuttgart, Olgahospital, Kriegsbergstr. 62, 70174, Stuttgart, Deutschland. a.hospach@klinikum-stuttgart.de.

Ruhlmann, J M. Praxis fur Kinderheilkunde und Jugendmedizin/Kinderrheumatologie, Gottingen, Deutschland.

Weller-Heinemann, F. Prof.-Hess-Kinderklinik, Zentrum fur Kinderheilkunde und Jugendmedizin, Klinikum Bremen - Mitte, Gesundheit Nord Klinikverbund Bremen, Bremen, Deutschland.

Abstract

Juvenile idiopathic arthritis (JIA) is the most common rheumatic disease in infancy and childhood. Approximately 20 % of patients with JIA suffer from the polyarticular form of the disease, which causes a substantial disease burden and long-term sequelae. Therapeutic approaches have used steroids and conventional disease modifying antirheumatic drugs (DMARD) but over the last decade new drugs have become available for the treatment of JIA, in particular biologic DMARD. This article summarizes the current therapy options for polyarticular JIA.

Publication Type

Journal Article.

Year of Publication

2016

<258>

Unique Identifier

26999417

Title

IL-1beta at the crossroad between rheumatoid arthritis and type 2 diabetes: may we kill two birds with one stone?. [Review]

Source

Expert Review of Clinical Immunology. 12(8):849-55, 2016 Aug.

VI 1

Status

MEDLINE

Authors

Giacomelli R; Ruscitti P; Alvaro S; Ciccia F; Liakouli V; Di Benedetto P; Guggino G; Berardicurti O; Carubbi F; Triolo G; Cipriani P.

Authors Full Name

Giacomelli, Roberto; Ruscitti, Piero; Alvaro, Saverio; Ciccia, Francesco; Liakouli, Vasiliki; Di Benedetto, Paola; Guggino, Giuliana; Berardicurti, Onorina; Carubbi, Francesco; Triolo, Giovanni; Cipriani, Paola.
Institution
Giacomelli, Roberto. a Division of Rheumatology, Department of Biotechnological and Applied Clinical Science, School of Medicine, University of L'Aquila, L'Aquila, Italy.
Ruscitti, Piero. a Division of Rheumatology, Department of Biotechnological and Applied Clinical Science, School of Medicine, University of L'Aquila, L'Aquila, Italy.
Alvaro, Saverio. a Division of Rheumatology, Department of Biotechnological and Applied Clinical Science, School of Medicine, University of L'Aquila, L'Aquila, Italy.
Ciccia, Francesco. b Division of Rheumatology, Department of Internal Medicine, University of Palermo, Palermo, Italy.
Liakouli, Vasiliki. a Division of Rheumatology, Department of Biotechnological and Applied Clinical Science, School of Medicine, University of L'Aquila, L'Aquila, Italy.
Di Benedetto, Paola. a Division of Rheumatology, Department of Biotechnological and Applied Clinical Science, School of Medicine, University of L'Aquila, L'Aquila, Italy.
Guggino, Giuliana. b Division of Rheumatology, Department of Internal Medicine, University of Palermo, Palermo, Italy.
Berardicurti, Onorina. a Division of Rheumatology, Department of Biotechnological and Applied Clinical Science, School of Medicine, University of L'Aquila, L'Aquila, Italy.
Carubbi, Francesco. a Division of Rheumatology, Department of Biotechnological and Applied Clinical Science, School of Medicine, University of L'Aquila, L'Aquila, Italy.
Triolo, Giovanni. b Division of Rheumatology, Department of Internal Medicine, University of Palermo, Palermo, Italy.
Cipriani, Paola. a Division of Rheumatology, Department of Biotechnological and Applied Clinical Science, School of Medicine, University of L'Aquila, L'Aquila, Italy.
Abstract
Although in the past the prevention of joint destruction in rheumatoid arthritis (RA) was strongly emphasized, now a great interest is focused on associated comorbidities in these patients. Multiple data suggest that a large percentage of RA patients are affected by Type 2 Diabetes (T2D), whose incidence has reached epidemic levels in recent years, thus increasing the health care costs. A better knowledge about the pathogenesis of these diseases as well as the mechanisms of action of drugs may allow both policy designers and physicians to choose the most effective treatments, thus lowering the costs. This review will focus on the role of Interleukin (IL)-1beta in the pathogenesis of both the diseases, the efficacy of IL-1 blocking molecules in controlling these diseases, and will provide information suggesting that targeting IL-1beta, in patients affected by both RA and T2D, may be a promising therapeutic choice.

Publication Type
  Journal Article. Review.
Year of Publication
  2016

<259>
Unique Identifier
  26995488
Title
  Evaluation of the immunogenicity of the 13-valent conjugated pneumococcal vaccine in rheumatoid arthritis patients treated with etanercept.
Source
  Joint, Bone, Spine: Revue du Rhumatisme. 83(6):675-679, 2016 Dec.
VI 1
Status
  MEDLINE
Authors
  Rakoczi E; Perge B; Vegh E; Csomor P; Pusztai A; Szamosi S; Bodnar N; Szanto S; Szucs G; Szekanecz Z.
Authors Full Name
  Rakoczi, Eva; Perge, Bianka; Vegh, Edit; Csomor, Peter; Pusztai, Anita; Szamosi, Szilvia; Bodnar, Nora; Szanto, Sandor; Szucs, Gabriella; Szekanecz, Zoltan.
Institution
  Rakoczi, Eva. Institute of Clinical Pharmacology, Infectious Diseases and Allergology, Kenezy Gyula Hospital, 2-6, Bartok B street, 4034 Debrecen, Hungary.
  Perge, Bianka. Department of Rheumatology, Medical and Health Sciences Center, Faculty of Medicine, University of Debrecen, 98, Nagyerdei street, 4032 Debrecen, Hungary.
  Vegh, Edit. Department of Rheumatology, Medical and Health Sciences Center, Faculty of Medicine, University of Debrecen, 98, Nagyerdei street, 4032 Debrecen, Hungary.
  Csomor, Peter. Department of Rheumatology, Medical and Health Sciences Center, Faculty of Medicine, University of Debrecen, 98, Nagyerdei street, 4032 Debrecen, Hungary.
  Pusztai, Anita. Department of Rheumatology, Medical and Health Sciences Center, Faculty of Medicine, University of Debrecen, 98, Nagyerdei street, 4032 Debrecen, Hungary.

Szamosi, Szilvia. Department of Rheumatology, Medical and Health Sciences Center, Faculty of Medicine, University of Debrecen, 98, Nagyerdei street, 4032 Debrecen, Hungary.
Bodnar, Nora. Department of Rheumatology, Medical and Health Sciences Center, Faculty of Medicine, University of Debrecen, 98, Nagyerdei street, 4032 Debrecen, Hungary.
Szanto, Sandor. Department of Rheumatology, Medical and Health Sciences Center, Faculty of Medicine, University of Debrecen, 98, Nagyerdei street, 4032 Debrecen, Hungary.
Szucs, Gabriella. Department of Rheumatology, Medical and Health Sciences Center, Faculty of Medicine, University of Debrecen, 98, Nagyerdei street, 4032 Debrecen, Hungary.
Szekanecz, Zoltan. Department of Rheumatology, Medical and Health Sciences Center, Faculty of Medicine, University of Debrecen, 98, Nagyerdei street, 4032 Debrecen, Hungary. Electronic address: szekanecz.zoltan@med.unideb.hu.
Abstract
OBJECTIVES: To prospectively evaluate the immunogenicity of a 13-valent conjugated pneumococcal vaccine (PCV13) in rheumatoid arthritis (RA) patients undergoing etanercept therapy.

METHODS: Twenty-two RA patients treated with etanercept (ETA) in combination with methotrexate (MTX) (n=15) or monotherapy (n=7) for at least one year were included. Altogether 24 osteoarthritis patients not receiving biological or MTX therapy, treating only NSAIDs or analgesics served as controls. All subjects were vaccinated with a single dose (0.5ml) of the PCV13. Pneumococcal antibody levels at baseline, 4 and 8weeks were assessed by a VaccZyme TM Anti-PCP IgG Enzyme Immunoassay Kit. Based on recommendations of the American Academy of Allergy, Asthma & Immunology, an at least two-fold increase in antibody level, as the protective antibody response (pAR) was an indicator of responsiveness (i.e., ratio of postvaccination and prevaccination antibody levels). The antibody levels and their ratios were analysed in a variety of different ways, vaccine safety parameters (fever, infections, changes in regular antirheumatic treatments) were assessed at baseline, 4 and 8weeks after vaccination.

RESULTS: Four weeks after vaccination, the anti-pneumococcal antibody levels significantly increased in both groups. At week 8, antibody levels somewhat decreased in both groups, however, still remained significantly higher compared to baseline. Compared with postvaccination levels at 4 and 8weeks between two groups, the mean protective antibody levels were higher in control group (1st month P=0.016; 2nd month: P=0.039). Possible predictors of pAR were analysed by logistic regression model. In RA, increases of antibody levels at week 8 compared to baseline exerted a negative correlation with age, (Spearman's R=-0,431; P=0.045). There were no clinically significant side effects or reaction after administration of vaccine observed in any of these patients after the 2-month follow-up period, all patients medical condition were stable.

CONCLUSIONS: In RA patients treated with ETA, vaccination with PCV13 is effective and safe, resulting in pAR one and two months after vaccination. Higher age at vaccination was identified as predictors of impaired pAR. The efficacy of vaccination may be more pronounced in younger RA patients. The vaccine is safe in RA patients on ETA. Copyright © 2016 Societe francaise de rhumatologie. Published by Elsevier SAS. All rights reserved.

Publication Type

Evaluation Study. Journal Article.

Year of Publication

2016

<260>

Unique Identifier

26986246

Title

Vaccinations for rheumatoid arthritis. [Review]

Source

Current Opinion in Rheumatology. 28(3):330-6, 2016 May.

VI 1

Status

MEDLINE

Authors

Friedman MA; Winthrop K.

Authors Full Name

Friedman, Marcia A; Winthrop, Kevin.

Institution

Friedman, Marcia A. aDepartment of Rheumatology bDepartment of Infectious Diseases, Oregon Health and Science University, Portland, Oregon, USA.

Abstract

PURPOSE OF REVIEW: Rheumatoid arthritis (RA) patients experience increased infectious disease-related morbidity and mortality, and vaccinations represent an important element in their care. However, vaccine immunogenicity can be affected by disease-modifying antirheumatic drug

(DMARD) therapy, such that vaccine choice and timing can be clinically challenging. We review the indications, safety, and immunogenicity of vaccines in the setting of RA.

RECENT FINDINGS: Recent recommendations highlight the use of influenza, pneumococcal, and shingles vaccines in RA patients. Studies suggest influenza and pneumococcal vaccines are underutilized, but well tolerated in RA patients and generally immunogenic during DMARD use with the exception of rituximab. Though data for other nonlive vaccines are more limited, hepatitis B virus and human papilloma virus vaccines also appear well tolerated and immunogenic in this population. Live vaccines for shingles and yellow fever remain contraindicated in some RA patients; however, limited data suggest they might be well tolerated in certain individuals.

SUMMARY: The review updates rheumatologists on the optimal use and timing of routine vaccinations in the care of RA.
Publication Type
Journal Article. Review.
Year of Publication
2016

<261>
Unique Identifier
26971876
Title
Evaluation of Clinical Metrology Instrument in Dogs with Osteoarthritis.
Source
Journal of Veterinary Internal Medicine. 30(3):836-46, 2016 May.
VI 1
Status
MEDLINE
Authors
Muller C; Gaines B; Gruen M; Case B; Arrufat K; Innes J; Lascelles BD.
Authors Full Name
Muller, C; Gaines, B; Gruen, M; Case, B; Arrufat, K; Innes, J; Lascelles, B D X.
Institution

Muller, C. Comparative Pain Research Laboratory, Department of Clinical Sciences, College of Veterinary Medicine, North Carolina State University, Raleigh, NC.

Gaines, B. Department of Statistics, North Carolina State University, Raleigh, NC.

Gruen, M. Comparative Pain Research Laboratory, Department of Clinical Sciences, College of Veterinary Medicine, North Carolina State University, Raleigh, NC.

Gruen, M. Center for Comparative Medicine and Translational Research, College of Veterinary Medicine, North Carolina State University, Raleigh, NC.

Case, B. Comparative Pain Research Laboratory, Department of Clinical Sciences, College of Veterinary Medicine, North Carolina State University, Raleigh, NC.

Arrufat, K. Comparative Pain Research Laboratory, Department of Clinical Sciences, College of Veterinary Medicine, North Carolina State University, Raleigh, NC.

Innes, J. Chester Gates Referral Hospital, Chester, UK.

Lascelles, B D X. Comparative Pain Research Laboratory, Department of Clinical Sciences, College of Veterinary Medicine, North Carolina State University, Raleigh, NC.

Lascelles, B D X. Center for Comparative Medicine and Translational Research, College of Veterinary Medicine, North Carolina State University, Raleigh, NC.

Lascelles, B D X. Center for Pain Research and Innovation, UNC School of Dentistry, Chapel Hill, NC.

Abstract

BACKGROUND: In veterinary clinical pain studies, there is a paucity of data on test-retest variability in Clinical Metrology Instruments (CMIs), and it is unknown whether CMIs should be administered using independent (respondents not permitted to see previous answers) or dependent (respondents shown previous answers) interviewing.

OBJECTIVES: To compare baseline variability in CMIs designed to assess pain in dogs with osteoarthritis, and compare CMI scores using independent (InD) and dependent interviewing (DI) for the Canine Brief Pain Inventory (CBPI) and the Client-Specific Outcome Measures (CSOM).

ANIMALS: Fifty-one client-owned dogs with radiographic evidence of osteoarthritis and associated pain.

METHODS: Clinical Metrology Instruments data were collected during 2 randomized, double-masked, placebo-controlled, proof of principle pilot studies with parallel treatment groups. Enrolled dogs received either placebo or antinerve growth factor antibody (NV-01).

RESULTS: Agreement between baseline CMI scores was good (CBPI Pain P = .29, CBPI Interference P = .32, CSOM P = .036, LOAD P = .67, HCPI P = .27), being best for the LOAD

(ICC = 0.89). CMI responses collected during independent and dependent interviewing were not statistically different (CBPI Pain P = .33, CBPI Interference P = .28, CSOM P = .42) and showed good agreement. Additionally, dependent interviewing resulted in increased treatment effect sizes.

CONCLUSIONS AND CLINICAL IMPORTANCE: There is little difference between independent and dependent interviewing, however, dependent interviewing resulted in increased treatment effect sizes. By using dependent interviewing, investigators could increase clinical trial power through minimal change to study design. Further research is warranted to investigate the use of dependent interviewing. Copyright © 2016 The Authors. Journal of Veterinary Internal Medicine published by Wiley Periodicals, Inc. on behalf of the American College of Veterinary Internal Medicine.

Publication Type
Journal Article. Randomized Controlled Trial.
Year of Publication
2016

<262>
Unique Identifier
26945335
Title
Retinal vasculitis. [Review]
Source
Current Opinion in Rheumatology. 28(3):228-35, 2016 May.
VI 1
Status
MEDLINE
Authors
Rosenbaum JT; Sibley CH; Lin P.
Authors Full Name
Rosenbaum, James T; Sibley, Cailin H; Lin, Phoebe.
Institution

Rosenbaum, James T. aDepartment of Ophthalmology, Casey Eye Institute, Oregon Health & Sciences University bDepartment of Medicine, Oregon Health & Sciences University cDepartment of Cell Biology, Oregon Health & Sciences University, Portland, Oregon, USA dLegacy Devers Eye Institute, Legacy Health System, Portland, Oregon, USA.
Abstract
PURPOSE OF REVIEW: Ophthalmologists and rheumatologists frequently have a miscommunication among themselves, and as a result differ in their opinion for patients consulting them with retinal vasculitis. This report seeks to establish a common understanding of the term, retinal vasculitis, and to review recent studies on this diagnosis.

RECENT FINDINGS: The genetic basis of some rare forms of retinal vascular disease has recently been described. Identified genes include CAPN5, TREX1, and TNFAIP3; Behcet's disease is a systemic illness that is very commonly associated with occlusive retinal vasculitis; retinal imaging, including fluorescein angiography and other newer imaging modalities, has proven crucial to the identification and characterization of retinal vasculitis and its complications; although monoclonal antibodies to interleukin-17A or interleukin-1 beta failed in trials for Behcet's disease, antibodies to TNF-alpha, either infliximab or adalimumab, have demonstrated consistent benefit in managing this disease. Interferon treatment and B-cell depletion therapy via rituximab may be beneficial in certain types of retinal vasculitis.

SUMMARY: Retinal vasculitis is an important entity for rheumatologists to understand. Retinal vasculitis associated with Behcet's disease responds to monoclonal antibodies that neutralize TNF, but the many other forms of noninfectious retinal vasculitis may require alternate therapeutic management.
Publication Type
Journal Article. Research Support, N.I.H., Extramural. Research Support, Non-U.S. Gov't. Review.
Year of Publication
2016

<263>
Unique Identifier
26927443

Title

Infection, malignancy, switching, biosimilars, antibody formation, drug survival and withdrawal, and dose reduction: what have we learned over the last year about tumor necrosis factor inhibitors in rheumatoid arthritis?. [Review]

Source

Current Opinion in Rheumatology. 28(3):303-9, 2016 May.

VI 1

Status

MEDLINE

Authors

Ianculescu I; Weisman MH.

Authors Full Name

Ianculescu, Irina; Weisman, Michael H.

Institution

Ianculescu, Irina. Division of Rheumatology, Cedars-Sinai Medical Center, Los Angeles, California, USA.

Abstract

PURPOSE OF REVIEW: This article reviews the most current studies investigating the use of tumor necrosis factor inhibitors (TNFis) in the treatment of rheumatoid arthritis.

RECENT FINDINGS: Studies over the past year have clarified that suppressing TNF with monoclonal antibodies does increase infection risk, yet coupled with reduction in disease activity and less use of corticosteroids as a consequence, the overall risk to the population is balanced. With caution (provided by some recent studies) TNFi agents can be reduced (dosage intervals lengthened) and maintain benefit. Biosimilars, not surprisingly, are going to be therapeutically identical to the innovator, and not more of a risk for causing antibodies to interfere with benefit. Uncertainty remains about when and how to make the switch.

SUMMARY: TNFi agents have made their powerful impact on management of patients with rheumatoid arthritis, but questions remain: what is their true infection and malignancy risk in the evolving populations using these drugs today; are we able to maintain their benefit with a reduced schedule (and presumed less cost) and yet recapture their benefit if we guess wrong; are biosimilars just as good, or even better with less cost; are there data to inform us about how to achieve successful switching among different mechanism of action TNFi agents? Finally, are we going to face the specter of cost containment causing change from innovator to biosimilars over which we have no control?

Publication Type

Journal Article. Review.
Year of Publication
2016

<264>
Unique Identifier
26924502
Title
The inextricable axis of targeted diagnostic imaging and therapy: An immunological natural history approach. [Review]
Source
Nuclear Medicine & Biology. 43(3):215-25, 2016 03.
VI 1
Status
MEDLINE
Authors
Cope FO; Abbruzzese B; Sanders J; Metz W; Sturms K; Ralph D; Blue M; Zhang J; Bracci P; Bshara W; Behr S; Maurer T; Williams K; Walker J; Beverly A; Blay B; Damughatla A; Larsen M; Mountain C; Neylon E; Parcel K; Raghuraman K; Ricks K; Rose L; Sivakumar A; Streck N; Wang B; Wasco C; Schlesinger LS; Azad A; Rajaram MVS; Jarjour W; Young N; Rosol T; Williams A; McGrath M.
Authors Full Name
Cope, Frederick O; Abbruzzese, Bonnie; Sanders, James; Metz, Wendy; Sturms, Kristyn; Ralph, David; Blue, Michael; Zhang, Jane; Bracci, Paige; Bshara, Wiam; Behr, Spencer; Maurer, Toby; Williams, Kenneth; Walker, Joshua; Beverly, Allison; Blay, Brooke; Damughatla, Anirudh; Larsen, Mark; Mountain, Courtney; Neylon, Erin; Parcel, Kaeli; Raghuraman, Kapil; Ricks, Kevin; Rose, Lucas; Sivakumar, Akhilesh; Streck, Nicholas; Wang, Bryan; Wasco, Christopher; Schlesinger, Larry S; Azad, Abul; Rajaram, Murugesan V S; Jarjour, Wael; Young, Nicholas; Rosol, Thomas; Williams, Amifred; McGrath, Michael.
Institution
Cope, Frederick O. Navidea Biopharmaceuticals, Drug Development, 5600 Blazer Parkway, Dublin, OH 43017. Electronic address: fcope@navidea.com.

Abbruzzese, Bonnie. Navidea Biopharmaceuticals, Drug Development, 5600 Blazer Parkway, Dublin, OH 43017.

Sanders, James. Navidea Biopharmaceuticals, Drug Development, 5600 Blazer Parkway, Dublin, OH 43017.

Metz, Wendy. Navidea Biopharmaceuticals, Drug Development, 5600 Blazer Parkway, Dublin, OH 43017.

Sturms, Kristyn. Navidea Biopharmaceuticals, Drug Development, 5600 Blazer Parkway, Dublin, OH 43017.

Ralph, David. Navidea Biopharmaceuticals, Drug Development, 5600 Blazer Parkway, Dublin, OH 43017.

Blue, Michael. Navidea Biopharmaceuticals, Drug Development, 5600 Blazer Parkway, Dublin, OH 43017.

Zhang, Jane. The University of California San Francisco and the San Francisco General Hospital, AIDS and Cancer Specimen Resource Center, The Department of Pathology, 1001 Potrero Ave, Bldg. 3, Rm 207 San Francisco, CA 94110.

Bracci, Paige. The University of California San Francisco and the San Francisco General Hospital, AIDS and Cancer Specimen Resource Center, The Department of Pathology, 1001 Potrero Ave, Bldg. 3, Rm 207 San Francisco, CA 94110.

Bshara, Wiam. Roswell Park Cancer Institute, Elm and Carlton Streets, Buffalo, NY 14263.

Behr, Spencer. Roswell Park Cancer Institute, Elm and Carlton Streets, Buffalo, NY 14263.

Maurer, Toby. Roswell Park Cancer Institute, Elm and Carlton Streets, Buffalo, NY 14263.

Williams, Kenneth. Boston College, Department of Biology, 14 Commonwealth Ave, Chestnut Hill, Massachusetts 02467.

Walker, Joshua. Boston College, Department of Biology, 14 Commonwealth Ave, Chestnut Hill, Massachusetts 02467.

Beverly, Allison. Navidea Biopharmaceuticals Drug Development Internship Program, 5600 Blazer Parkway, Dublin, OH 43017.

Blay, Brooke. Navidea Biopharmaceuticals Drug Development Internship Program, 5600 Blazer Parkway, Dublin, OH 43017.

Damughatla, Anirudh. Navidea Biopharmaceuticals Drug Development Internship Program, 5600 Blazer Parkway, Dublin, OH 43017.

Larsen, Mark. Navidea Biopharmaceuticals Drug Development Internship Program, 5600 Blazer Parkway, Dublin, OH 43017.

Mountain, Courtney. Navidea Biopharmaceuticals Drug Development Internship Program, 5600 Blazer Parkway, Dublin, OH 43017.

Neylon, Erin. Navidea Biopharmaceuticals Drug Development Internship Program, 5600 Blazer Parkway, Dublin, OH 43017.

Parcel, Kaeli. Navidea Biopharmaceuticals Drug Development Internship Program, 5600 Blazer Parkway, Dublin, OH 43017.
Raghuraman, Kapil. Navidea Biopharmaceuticals Drug Development Internship Program, 5600 Blazer Parkway, Dublin, OH 43017.
Ricks, Kevin. Navidea Biopharmaceuticals Drug Development Internship Program, 5600 Blazer Parkway, Dublin, OH 43017.
Rose, Lucas. Navidea Biopharmaceuticals Drug Development Internship Program, 5600 Blazer Parkway, Dublin, OH 43017.
Sivakumar, Akhilesh. Navidea Biopharmaceuticals Drug Development Internship Program, 5600 Blazer Parkway, Dublin, OH 43017.
Streck, Nicholas. Navidea Biopharmaceuticals Drug Development Internship Program, 5600 Blazer Parkway, Dublin, OH 43017.
Wang, Bryan. Navidea Biopharmaceuticals Drug Development Internship Program, 5600 Blazer Parkway, Dublin, OH 43017.
Wasco, Christopher. Navidea Biopharmaceuticals Drug Development Internship Program, 5600 Blazer Parkway, Dublin, OH 43017.
Williams, Amifred. Navidea Biopharmaceuticals Drug Development Internship Program, 5600 Blazer Parkway, Dublin, OH 43017.
McGrath, Michael. The University of California San Francisco and the San Francisco General Hospital, AIDS and Cancer Specimen Resource Center, The Department of Pathology, 1001 Potrero Ave, Bldg. 3, Rm 207 San Francisco, CA 94110.
Comments
Erratum in (EIN)
Abstract
In considering the challenges of approaches to clinical imaging, we are faced with choices that sometimes are impacted by rather dogmatic notions about what is a better or worse technology to achieve the most useful diagnostic image for the patient. For example, is PET or SPECT most useful in imaging any particular disease dissemination? The dictatorial approach would be to choose PET, all other matters being equal. But is such a totalitarian attitude toward imaging selection still valid? In the face of new receptor targeted SPECT agents one must consider the remarkable specificity and sensitivity of these agents. (99m)Tc-Tilmanocept is one of the newest of these agents, now approved for guiding sentinel node biopsy (SLNB) in several solid tumors. Tilmanocept has a Kd of 3x10(-11)M, and it specificity for the CD206 receptor is unlike any other agent to date. This coupled with a number of facts, that specific disease-associated macrophages express this receptor (100 to 150 thousand receptors), that the receptor has multiple binding sites for tilmanocept (>2 sites per receptor) and that these receptors are recycled every 15 min to bind more tilmanocept (acting as intracellular "drug compilers" of tilmanocept into non-degraded

vesicles), gives serious pause as to how we select our approaches to diagnostic imaging. Clinically, the size of SLNs varies greatly, some, anatomically, below the machine resolution of SPECT. Yet, with tilmanocept targeting, the SLNs are highly visible with macrophages stably accruing adequate (99m)Tc-tilmanocept counting statistics, as high target-to-background ratios can compensate for spatial resolution blurring. Importantly, it may be targeted imaging agents per se, again such as tilmanocept, which may significantly shrink any perceived chasm between the imaging technologies and anchor the diagnostic considerations in the targeting and specificity of the agent rather than any lingering dogma about the hardware as the basis for imaging approaches. Beyond the elements of imaging applications of these agents is their evolution to therapeutic agents as well, and even in the neo-logical realm of theranostics. Characteristics of agents such as tilmanocept that exploit the natural history of diseases with remarkably high specificity are the expectations for the future of patient- and disease-centered diagnosis and therapy. Copyright © 2015 The Authors. Published by Elsevier Inc. All rights reserved.

Publication Type

Journal Article. Review.

Year of Publication

2016

<265>

Unique Identifier

26903194

Title

IL-1 receptor antagonist (IL-1Ra)-Fc ameliorate autoimmune arthritis by regulation of the Th17 cells/Treg balance and arthrogenic cytokine activation.

Source

Immunology Letters. 172:56-66, 2016 04.

VI 1

Status

MEDLINE

Authors

Lee SY; Min HK; Lee SH; Shin HJ; Lee WY; Cho YG; Kwok SK; Ju JH; Cho ML; Park SH.

Authors Full Name

Lee, Seon-Yeong; Min, Hong Ki; Lee, Seung Hoon; Shin, Hye Jeong; Lee, Woon Young; Cho, Young-Gyu; Kwok, Seung-Ki; Ju, Ji Hyeon; Cho, Mi-La; Park, Sung-Hwan.
Institution
Lee, Seon-Yeong. Rheumatism Research Center, Catholic Research Institute of Medical Science, The Catholic University of Korea, Seoul 137-040, South Korea. Electronic address: seonyeong@catholic.ac.kr.
Min, Hong Ki. Rheumatism Research Center, Catholic Research Institute of Medical Science, The Catholic University of Korea, Seoul 137-040, South Korea; Division of Rheumatology, Department of internal Medicine, School of Medicine, The Catholic University of Korea, Seoul 137-070, South Korea. Electronic address: alsghdrl1921@naver.com.
Lee, Seung Hoon. Rheumatism Research Center, Catholic Research Institute of Medical Science, The Catholic University of Korea, Seoul 137-040, South Korea. Electronic address: redcap817@catholic.ac.kr.
Shin, Hye Jeong. HANDOK Inc. 132, Theheran street, Gangnam-gu, Seoul 135-755, South Korea. Electronic address: hyejeong.shin@handok.com.
Lee, Woon Young. HANDOK Inc. 132, Theheran street, Gangnam-gu, Seoul 135-755, South Korea. Electronic address: woonyoung.lee@handok.com.
Cho, Young-Gyu. HANDOK Inc. 132, Theheran street, Gangnam-gu, Seoul 135-755, South Korea. Electronic address: younggyu.cho@handok.com.
Kwok, Seung-Ki. Rheumatism Research Center, Catholic Research Institute of Medical Science, The Catholic University of Korea, Seoul 137-040, South Korea; Division of Rheumatology, Department of internal Medicine, School of Medicine, The Catholic University of Korea, Seoul 137-070, South Korea. Electronic address: seungki73@catholic.ac.kr.
Ju, Ji Hyeon. Rheumatism Research Center, Catholic Research Institute of Medical Science, The Catholic University of Korea, Seoul 137-040, South Korea; Division of Rheumatology, Department of internal Medicine, School of Medicine, The Catholic University of Korea, Seoul 137-070, South Korea. Electronic address: juji@catholic.ac.kr.
Cho, Mi-La. Rheumatism Research Center, Catholic Research Institute of Medical Science, The Catholic University of Korea, Seoul 137-040, South Korea. Electronic address: iammila@catholic.ac.kr.
Park, Sung-Hwan. Rheumatism Research Center, Catholic Research Institute of Medical Science, The Catholic University of Korea, Seoul 137-040, South Korea; Division of Rheumatology, Department of internal Medicine, School of Medicine, The Catholic University of Korea, Seoul 137-070, South Korea. Electronic address: rapark@catholic.ac.kr.
Abstract
INTRODUCTION: IL-1beta signalling has a critical role in the pathogenesis of various types of inflammatory arthritis including rheumatoid arthritis (RA). We aimed to investigate the therapeutic

effects of human IL-1 receptor antagonist with Fc fragment (hIL-1Ra-Fc) on autoimmune arthritis and to identify the possible mechanisms by which hIL-1RA-Fc exerts anti-arthritic effects in a murine model of RA and patients with rheumatoid arthritis.

METHODS: Collagen-induced arthritis (CIA) murine model was established in DBA/1J mice. The levels of various cytokines were determined by using enzyme-linked immunosorbent assay. The mouse joints were assessed for clinical arthritis score and histologic features. Th17 cells and Treg cells were stained by using antibodies specific for CD4, IL-17, CD25, and FoxP3. Osteoclastogenesis was determined by TRAP staining and real-time PCR.

RESULTS: hIL-1RA-Fc reduced the arthritis incidence, histological inflammation and cartilage score in the CIA model. The expression of proinflammatory cytokines, VEGF and RANK, was reduced in the affected joint of hIL-1Ra-Fc-treated mice. hIL-1Ra-Fc-treated mice showed decreased number of Th17 cells with increased number of Treg cells in spleens. hIL-1Ra-Fc reduced Th17 cell differentiation by inactivation of STAT3 signalling, and reciprocally induced Treg cell differentiation through STAT5 signalling. In addition, the expression of IL-17, TNF-alpha, RANKL, and VEGF was decreased, while Foxp3 gene expression was increased in PBMCs of RA patients after administration of hIL-1Ra-Fc.

CONCLUSION: The anti-arthritis effects of hIL-1RA-Fc are associated with regulation of balance between Th17 cells and Treg cells and suppression of osteoclastogenesis and angiogenesis in the affected joints. Copyright © 2016 European Federation of Immunological Societies. Published by Elsevier B.V. All rights reserved.
Publication Type
  Case Reports. Journal Article. Research Support, Non-U.S. Gov't.
Year of Publication
  2016

<266>
Unique Identifier
  26875450
Title
  Novel Immunotherapeutic Avenues for Rheumatoid Arthritis. [Review]

Source
Trends in Molecular Medicine. 22(3):214-229, 2016 Mar.
VI 1
Status
MEDLINE
Authors
Semerano L; Minichiello E; Bessis N; Boissier MC.
Authors Full Name
Semerano, Luca; Minichiello, Emeline; Bessis, Natacha; Boissier, Marie-Christophe.
Institution
Semerano, Luca. Inserm UMR 1125, 74, rue Marcel Cachin, 93017, Bobigny, France; Sorbonne Paris Cite - Universite Paris 13, 74 rue Marcel Cachin, 93017, Bobigny, France; Assistance Publique - Hopitaux de Paris (AP-HP) Groupe hospitalier Avicenne - Jean Verdier - Rene Muret, Service de Rhumatologie, 125 rue de Stalingrad, 93017, Bobigny, France.
Minichiello, Emeline. Sorbonne Paris Cite - Universite Paris 13, 74 rue Marcel Cachin, 93017, Bobigny, France; Assistance Publique - Hopitaux de Paris (AP-HP) Groupe hospitalier Avicenne - Jean Verdier - Rene Muret, Service de Rhumatologie, 125 rue de Stalingrad, 93017, Bobigny, France.
Bessis, Natacha. Inserm UMR 1125, 74, rue Marcel Cachin, 93017, Bobigny, France; Sorbonne Paris Cite - Universite Paris 13, 74 rue Marcel Cachin, 93017, Bobigny, France.
Boissier, Marie-Christophe. Inserm UMR 1125, 74, rue Marcel Cachin, 93017, Bobigny, France; Sorbonne Paris Cite - Universite Paris 13, 74 rue Marcel Cachin, 93017, Bobigny, France; Assistance Publique - Hopitaux de Paris (AP-HP) Groupe hospitalier Avicenne - Jean Verdier - Rene Muret, Service de Rhumatologie, 125 rue de Stalingrad, 93017, Bobigny, France. Electronic address: marie-christophe.boissier@aphp.fr.
Abstract
Rheumatoid arthritis (RA) is the most common inflammatory rheumatic disease. It leads to irreversible joint damage, physical handicap, and reduced life expectancy. The past two decades have seen considerable therapeutic advances with the development of biologic treatments to block proinflammatory cytokines or modulate lymphocyte function, followed by the development of small molecules to target intracellular signaling. Nevertheless, only a minority of patients can achieve disease remission, especially long term, warranting further investigation into newer therapeutic options. Targeting single proinflammatory pathways may not be sufficient, as suggested by variable results with T helper (Th)-17-related cytokine blockade. Multilevel information from 'omics' techniques along with data from mechanistic studies might facilitate the identification of pivotal checkpoints in RA disease pathogenesis and the subsequent development of new effective treatments. Copyright © 2016. Published by Elsevier Ltd.

Publication Type
  Journal Article. Review.
Year of Publication
  2016

<267>
Unique Identifier
  26838521
Title
  [Lung cancer and rheumatoid arthritis. An interdisciplinary challenge]. [Review] [German]
Source
  Zeitschrift fur Rheumatologie. 75(1):47-53, 2016 Feb.
VI 1
Status
  MEDLINE
Authors
  Rubbert-Roth A; Zander T; Kneitz C; Baerwald C; Wirtz H; Witt C.
Authors Full Name
  Rubbert-Roth, A; Zander, T; Kneitz, C; Baerwald, C; Wirtz, H; Witt, C.
Institution
  Rubbert-Roth, A. Klinik I fur Innere Medizin, Universitatsklinik Koln, Kerpenerstr. 62, 50924, Koln, Deutschland. andrea.rubbert@uk-koeln.de.
  Zander, T. Klinik I fur Innere Medizin, Universitatsklinik Koln, Kerpenerstr. 62, 50924, Koln, Deutschland.
  Kneitz, C. Sudstadtklinikum Rostock, Rostock, Deutschland.
  Baerwald, C. Sektion Rheumatologie, Universitatsklinikum Leipzig, Leipzig, Deutschland.
  Wirtz, H. Abteilung Pneumologie, Universitatsklinikum Leipzig, Leipzig, Deutschland.
  Witt, C. Arbeitsbereich Pneumologische Onkologie, Charite-Universitatsmedizin Berlin, Berlin, Deutschland.
Abstract
  Lung cancer is a frequently occurring disease, particularly in the elderly; however, within the last 10 years the pharmaceutical treatment of lung cancer has been significantly improved. Due to a better understanding of the pathophysiological events and the identification of molecular

subgroups of lung tumors, new therapeutic drugs have been developed that significantly prolong survival of patients with the respective molecular pattern. In particular immunotherapeutic agents, such as programmed death-ligand 1 (PD-L1) and programmed death 1 (PD1) antibodies have shown promising clinical results in a subgroup of lung cancer patients. Due to the high incidence of both lung cancer and rheumatic diseases they often occur together, which necessitates an interdisciplinary management. The success of improved therapy of lung cancer has led to a greater focus on the treatment of comorbidities; however, interventions into the immune system by immune checkpoint inhibitors can lead to new challenges when an autoimmune disease is simultaneously present. The possibility of an effective screening for lung cancer in the future also presents the prospect of an improvement in mortality, which raises the question of the optimal monitoring of patients with rheumatoid arthritis (RA) under immunosuppressive therapy. The aim of this review is to discuss the interaction between lung cancer and RA with respect to the currently available data.

Publication Type

Journal Article. Meta-Analysis. Review.

Year of Publication

2016

<268>

Unique Identifier

26831226

Title

Cytomegalovirus Pneumonia in Patients with Rheumatic Diseases After Immunosuppressive Therapy: A Single Center Study in China.

Source

Chinese Medical Journal. 129(3):267-73, 2016 Feb 05.

VI 1

Status

MEDLINE

Authors

Xue Y; Jiang L; Wan WG; Chen YM; Zhang J; Zhang ZC.

Authors Full Name

Xue, Yu; Jiang, Li; Wan, Wei-Guo; Chen, Yu-Ming; Zhang, Jiong; Zhang, Zhen-Chun.

Institution
Zhang, Zhen-Chun. Institute of Rheumatology, Immunology and Allergy, Fudan University, Shanghai 200040; Department of Rheumatology, Linyi People's Hospital, Linyi, Shandong 276000, China.
Abstract
BACKGROUND: Rheumatic diseases involve multiple organs that are affected by immunological mechanisms. Treatment with corticosteroids and immunosuppressive agents may also increase the frequency of infection. Cytomegalovirus (CMV) is a widespread herpes virus and a well-recognized pathogen, which causes an opportunistic and potentially fatal infection in immunocompromised patients. This retrospective study aimed to investigate the clinical and laboratory characteristics of CMV pneumonia in patients with rheumatic diseases after immunosuppressive therapy in a single center in Shanghai, China.

METHODS: Eight hundred and thirty-four patients with rheumatic diseases who had undergone CMV-DNA viral load tests were included, and the medical records of 142 patients who were positive for CMV-DNA in plasma samples were evaluated. GraphPad Prism version 5.013 (San Diego, CA, USA) was used to conduct statistical analysis. The correlation between CMV-DNA viral loads and lymphocyte counts was assessed using the Spearman rank correlation coefficient test. Significance between qualitative data was analyzed using Pearson's Chi-squared test. The cut-off thresholds for CMV-DNA viral load and lymphocyte count were determined by receiver operating characteristic (ROC) curve analysis.

RESULTS: One hundred and forty-two patients had positive CMV viral load tests. Of these 142 patients, 73 patients with CMV pneumonia were regarded as symptomatic, and the other 69 were asymptomatic. The symptomatic group received higher doses of prednisolone (PSL) and more frequently immunosuppressants than the asymptomatic group ($P < 0.01$). The symptomatic group had lower lymphocyte counts, especially CD4+ T-cells, than the asymptomatic group ($P < 0.01$). By ROC curve analysis, when CD4+ T-cell count was <0.39 x 109/L, patients with rheumatic diseases were at high risk for symptomatic CMV infection. The CMV-DNA load was significantly higher in the symptomatic patients than that in asymptomatic patients ($P < 0.01$; threshold viral loads: 1.75 x 104 copies/ml). Seven patients had a fatal outcome, and they had lower peripheral lymphocyte counts ($P < 0.01$), including CD4+ and CD8+ T-cells ($P < 0.01$).

CONCLUSIONS: When CD4+ T-cell count is <0.39 x 109/L, patients are at high risk for pulmonary CMV infection. Patients are prone to be symptomatic with CMV-DNA load >1.75 x 104 copies/ml. Lymphopenia (especially CD4+ T-cells), presence of symptoms, and other infections,

especially fungal infection, are significant risk factors for poor outcome, and a higher PSL dosage combined with immunosuppressants may predict CMV pneumonia.

Publication Type

Journal Article.

Year of Publication

2016

<269>

Unique Identifier

26822477

Title

Depletion of regulatory T cells leads to an exacerbation of delayed-type hypersensitivity arthritis in C57BL/6 mice that can be counteracted by IL-17 blockade.

Source

Disease Models & Mechanisms. 9(4):427-40, 2016 Apr.

VI 1

Status

MEDLINE

Authors

Atkinson SM; Hoffmann U; Hamann A; Bach E; Danneskiold-Samsoe NB; Kristiansen K; Serikawa K; Fox B; Kruse K; Haase C; Skov S; Nansen A.

Authors Full Name

Atkinson, Sara Marie; Hoffmann, Ute; Hamann, Alf; Bach, Emil; Danneskiold-Samsoe, Niels Banhos; Kristiansen, Karsten; Serikawa, Kyle; Fox, Brian; Kruse, Kim; Haase, Claus; Skov, Soren; Nansen, Anneline.

Institution

Atkinson, Sara Marie. Department of Diabetes Complications Research, Global Research, Novo Nordisk A/S, Maaloev 2760, Denmark Department of Veterinary Disease Biology, University of Copenhagen, Frederiksberg 1870, Denmark sara.marie.atkinson@gmail.com.

Hoffmann, Ute. Experimentelle Rheumatologie, Deutsches Rheuma-Forschungszentrum and Charite-Universitatsmedizin Berlin, Berlin 10117, Germany.

Hamann, Alf. Experimentelle Rheumatologie, Deutsches Rheuma-Forschungszentrum and Charite-Universitatsmedizin Berlin, Berlin 10117, Germany.

Bach, Emil. Laboratory of Genomics and Molecular Biomedicine, Department of Biology, University of Copenhagen, Copenhagen 2100, Denmark.

Danneskiold-Samsoe, Niels Banhos. Laboratory of Genomics and Molecular Biomedicine, Department of Biology, University of Copenhagen, Copenhagen 2100, Denmark.

Kristiansen, Karsten. Laboratory of Genomics and Molecular Biomedicine, Department of Biology, University of Copenhagen, Copenhagen 2100, Denmark.

Serikawa, Kyle. Benaroya Research Institute, Seattle, WA 98101, USA.

Fox, Brian. Immunexpress, Seattle, WA 98109, USA.

Kruse, Kim. Department of Diabetes Complications Research, Global Research, Novo Nordisk A/S, Maaloev 2760, Denmark.

Haase, Claus. Department of Diabetes Complications Research, Global Research, Novo Nordisk A/S, Maaloev 2760, Denmark.

Skov, Soren. Department of Veterinary Disease Biology, University of Copenhagen, Frederiksberg 1870, Denmark.

Nansen, Anneline. Department of Pharmacology, Zealand Pharma, Glostrup 2600, Denmark.

Abstract

Rodent models of arthritis have been extensively used in the elucidation of rheumatoid arthritis (RA) pathogenesis and are instrumental in the development of therapeutic strategies. Here we utilise delayed-type hypersensitivity arthritis (DTHA), a model in C57BL/6 mice affecting one paw with synchronised onset, 100% penetrance and low variation. We investigate the role of regulatory T cells (Tregs) in DTHA through selective depletion of Tregsand the role of IL-17 in connection with Tregdepletion. Given the relevance of Tregsin RA, and the possibility of developing Treg-directed therapies, this approach could be relevant for advancing the understanding of Tregsin inflammatory arthritis. Selective depletion of Tregswas achieved using aFoxp3-DTR-eGFPmouse, which expresses the diphtheria toxin receptor (DTR) and enhanced green fluorescent protein (eGFP) under control of theFoxp3gene. Anti-IL-17 monoclonal antibody (mAb) was used for IL-17 blockade. Numbers and activation of Tregsincreased in the paw and its draining lymph node in DTHA, and depletion of Tregsresulted in exacerbation of disease as shown by increased paw swelling, increased infiltration of inflammatory cells, increased bone remodelling and increased production of inflammatory mediators, as well as increased production of anti-citrullinated protein antibodies. Anti-IL-17 mAb treatment demonstrated that IL-17 is important for disease severity in both the presence and absence of Tregs, and that IL-17 blockade is able to rescue mice from the exacerbated disease caused by Tregdepletion and caused a reduction in RANKL, IL-6 and the number of neutrophils. We show that Tregsare important for the containment of inflammation and bone remodelling in DTHA. To our knowledge, this is the first study using theFoxp3-DTR-eGFPmouse on a C57BL/6 background for Tregdepletion in an arthritis model, and we here demonstrate the usefulness of the approach to

study the role of Tregsand IL-17 in arthritis. Copyright © 2016. Published by The Company of Biologists Ltd.
Publication Type
Journal Article. Research Support, Non-U.S. Gov't.
Year of Publication
2016

<270>
Unique Identifier
26805457
Title
The immunosuppressive effect of domain-deleted dimer of HLA-G2 isoform in collagen-induced arthritis mice.
Source
Human Immunology. 77(9):754-9, 2016 Sep.
VI 1
Status
MEDLINE
Authors
Takahashi A; Kuroki K; Okabe Y; Kasai Y; Matsumoto N; Yamada C; Takai T; Ose T; Kon S; Matsuda T; Maenaka K.
Authors Full Name
Takahashi, Ami; Kuroki, Kimiko; Okabe, Yuki; Kasai, Yoshiyuki; Matsumoto, Naoki; Yamada, Chisato; Takai, Toshiyuki; Ose, Toyoyuki; Kon, Shigeyuki; Matsuda, Tadashi; Maenaka, Katsumi.
Institution
Takahashi, Ami. Laboratory of Biomolecular Science, Faculty of Pharmaceutical Sciences, Hokkaido University, Sapporo, Japan.
Kuroki, Kimiko. Laboratory of Biomolecular Science, Faculty of Pharmaceutical Sciences, Hokkaido University, Sapporo, Japan. Electronic address: k-kimiko@pharm.hokudai.ac.jp.
Okabe, Yuki. Laboratory of Biomolecular Science, Faculty of Pharmaceutical Sciences, Hokkaido University, Sapporo, Japan.
Kasai, Yoshiyuki. Laboratory of Biomolecular Science, Faculty of Pharmaceutical Sciences, Hokkaido University, Sapporo, Japan.

Matsumoto, Naoki. Department of Immunology, Faculty of Pharmaceutical Sciences, Hokkaido University, Sapporo, Japan.
Yamada, Chisato. Laboratory of Biomolecular Science, Faculty of Pharmaceutical Sciences, Hokkaido University, Sapporo, Japan.
Takai, Toshiyuki. Department of Experimental Immunology, IDAC, Tohoku University, Sendai, Japan.
Ose, Toyoyuki. Laboratory of Biomolecular Science, Faculty of Pharmaceutical Sciences, Hokkaido University, Sapporo, Japan.
Kon, Shigeyuki. Department of Immunology, Faculty of Pharmaceutical Sciences, Hokkaido University, Sapporo, Japan.
Matsuda, Tadashi. Department of Immunology, Faculty of Pharmaceutical Sciences, Hokkaido University, Sapporo, Japan.
Maenaka, Katsumi. Laboratory of Biomolecular Science, Faculty of Pharmaceutical Sciences, Hokkaido University, Sapporo, Japan; Center for Research and Education on Drug Discovery, Faculty of Pharmaceutical Sciences, Hokkaido University, Sapporo, Japan. Electronic address: maenaka@pharm.hokudai.ac.jp.
Abstract
HLA-G is involved in maternal-fetal immune tolerance and is reported to be a natural tolerogenic molecule. Seven-spliced isoforms including dimeric and beta2m-free forms have been identified. The major isoform, HLA-G1 (and its soluble type HLA-G5), binds to the inhibitory immune receptors, leukocyte immunoglobulin (Ig)-like receptor (LILR) B1 and LILRB2. We previously reported that HLA-G1 also binds to paired Ig-like receptor (PIR)-B, a mouse homolog of LILRBs, and had a significant immunosuppressive effect in collagen-induced arthritis (CIA) mice. Although HLA-G2 and its soluble form HLA-G6 bind specifically to LILRB2, its functional characteristics are largely unknown. In this study, we report the significant immunosuppressive effect of HLA-G2 dimer in CIA mice. Surface plasmon resonance analysis revealed a specific interaction of HLA-G2 with PIR-B. CIA mice were administered HLA-G2 protein subcutaneously once in the left footpad and clinical severity was evaluated in a double-blind study. A single administration of HLA-G2 maintained a suppressive effect for over 1month. These results suggested that the HLA-G2 protein might be a useful biopharmaceutical for the treatment of rheumatoid arthritis by binding to inhibitory PIR-B. Copyright © 2016 American Society for Histocompatibility and Immunogenetics. Published by Elsevier Inc. All rights reserved.
Publication Type
Journal Article.
Year of Publication
2016

<271>

Unique Identifier

26799279

Title

Colonic Diverticulitis in a 19-Year-Old Boy With Juvenile Idiopathic Arthritis: Surgical Implications of Chronic Immunosuppression.

Source

Journal of Pediatric Gastroenterology & Nutrition. 62(2):e15-7, 2016 Feb.

VI 1

Status

MEDLINE

Authors

Cameron DB; Scherl A; Fitzgerald T; Ozgediz D.

Authors Full Name

Cameron, Danielle B; Scherl, Alexis; Fitzgerald, Tamara; Ozgediz, Doruk.

Institution

Cameron, Danielle B. *Department of Surgery Department of Pathology, Yale University School of Medicine, Yale New Haven Hospital, New Haven, CT++Department of Surgery, Texas Tech University Health Sciences Center at El Paso Paul L. Foster School of Medicine, El Paso, TX.

Publication Type

Case Reports. Journal Article.

Year of Publication

2016

<272>

Unique Identifier

26794912

Title

Mimotope mimicking epidermal growth factor receptor alleviates mononuclear cell infiltration in exocrine glands induced by muscarinic acetylcholine 3 receptor.

Source

Clinical Immunology. 163:111-9, 2016 Feb.

VI 1

Status

MEDLINE

Authors

Yang L; Wang Y; Xing R; Bai L; Li C; Li Z; Liu X.

Authors Full Name

Yang, Lin; Wang, Yongfu; Xing, Rui; Bai, Li; Li, Changhong; Li, Zhengqian; Liu, Xiangyuan.

Institution

Yang, Lin. Department of Rheumatology and Immunology, Peking University Third Hospital, No.49, HuaYuan (North) Road, Beijing 100191, PR China.

Wang, Yongfu. Department of Rheumatology, The First Affiliated Hospital of Baotou Medical College, No.41, LinYin Road, Baotou 014010, PR China.

Xing, Rui. Department of Rheumatology and Immunology, Peking University Third Hospital, No.49, HuaYuan (North) Road, Beijing 100191, PR China.

Bai, Li. Department of Rheumatology, The First Affiliated Hospital of Baotou Medical College, No.41, LinYin Road, Baotou 014010, PR China.

Li, Changhong. Department of Rheumatology and Immunology, Peking University Third Hospital, No.49, HuaYuan (North) Road, Beijing 100191, PR China.

Li, Zhengqian. Department of Anesthesiology, Peking University Third Hospital, No.49, HuaYuan (North) Road, Beijing 100191, PR China.

Liu, Xiangyuan. Department of Rheumatology and Immunology, Peking University Third Hospital, No.49, HuaYuan (North) Road, Beijing 100191, PR China. Electronic address: liuxiangyuan2014@hotmail.com.

Abstract

The muscarinic type 3 receptor (M3R) plays a pivotal role in the pathogenesis of Sjogren's syndrome (SS). Characterization of the crosstalk between M3R and EGFR has been investigated in some human malignancies. In the current study, we sought to investigate whether EGFR mimic immunization could alleviate the abnormal immune responses in an experimental SS-like model triggered by M3R peptides. After immunization with the combination of mimotope and M3R peptide, the active immunization targeting EGFR induced by the mimotope could reduce the marked infiltration of mononuclear cells, the high titer of antibodies against M3R and the accumulation of crucial pro-inflammatory cytokines in mice immunized with M3R peptide. Mechanistic analysis showed that mimotope immunization could alleviate the autoimmune

response through inhibiting mitochondrion-mediated anti-apoptosis and up-regulating the FAS apoptosis pathway. These results may help to clarify the role of M3R in the pathogenesis of SS and suggested that transactivation of the EGFR signaling pathway may help M3R activate the autoimmune response in the pathogenesis of SS. Copyright © 2016 Elsevier Inc. All rights reserved.

Publication Type

Journal Article. Research Support, Non-U.S. Gov't.

Year of Publication

2016

<273>

Unique Identifier

26773117

Title

Longterm Efficacy of an Antipneumococcal Polysaccharide Vaccine among Patients with Autoimmune Inflammatory Rheumatic Diseases.

Source

Journal of Rheumatology. 43(2):267-72, 2016 Feb.

VI 1

Status

MEDLINE

Authors

Broyde A; Arad U; Madar-Balakirski N; Paran D; Kaufman I; Levartovsky D; Wigler I; Caspi D; Elkayam O.

Authors Full Name

Broyde, Adi; Arad, Uri; Madar-Balakirski, Noa; Paran, Daphna; Kaufman, Ilana; Levartovsky, David; Wigler, Irena; Caspi, Dan; Elkayam, Ori.

Institution

Broyde, Adi. From the Department of Rheumatology, Tel Aviv Medical Center, Sackler School of Medicine, Tel Aviv University, Tel Aviv, Israel.A. Broyde, MD, Department of Rheumatology, Tel Aviv Medical Center, Sackler School of Medicine, Tel Aviv University; U. Arad, MD, PhD, Department of Rheumatology, Tel Aviv Medical Center, Sackler School of Medicine, Tel Aviv University; N. Madar-Balakirski, PhD, Department of Rheumatology, Tel Aviv Medical Center,

Sackler School of Medicine, Tel Aviv University; D. Paran, MD, Department of Rheumatology, Tel Aviv Medical Center, Sackler School of Medicine, Tel Aviv University; I. Kaufman, MD, Department of Rheumatology, Tel Aviv Medical Center, Sackler School of Medicine, Tel Aviv University; D. Levartovsky, MD, Department of Rheumatology, Tel Aviv Medical Center, Sackler School of Medicine, Tel Aviv University; I. Wigler, MD, Department of Rheumatology, Tel Aviv Medical Center, Sackler School of Medicine, Tel Aviv University; D. Caspi, MD, Department of Rheumatology, Tel Aviv Medical Center, Sackler School of Medicine, Tel Aviv University; O. Elkayam, M.D, Department of Rheumatology, Tel Aviv Medical Center, Sackler School of Medicine, Tel Aviv University.

Arad, Uri. From the Department of Rheumatology, Tel Aviv Medical Center, Sackler School of Medicine, Tel Aviv University, Tel Aviv, Israel.A. Broyde, MD, Department of Rheumatology, Tel Aviv Medical Center, Sackler School of Medicine, Tel Aviv University; U. Arad, MD, PhD, Department of Rheumatology, Tel Aviv Medical Center, Sackler School of Medicine, Tel Aviv University; N. Madar-Balakirski, PhD, Department of Rheumatology, Tel Aviv Medical Center, Sackler School of Medicine, Tel Aviv University; D. Paran, MD, Department of Rheumatology, Tel Aviv Medical Center, Sackler School of Medicine, Tel Aviv University; I. Kaufman, MD, Department of Rheumatology, Tel Aviv Medical Center, Sackler School of Medicine, Tel Aviv University; D. Levartovsky, MD, Department of Rheumatology, Tel Aviv Medical Center, Sackler School of Medicine, Tel Aviv University; I. Wigler, MD, Department of Rheumatology, Tel Aviv Medical Center, Sackler School of Medicine, Tel Aviv University; D. Caspi, MD, Department of Rheumatology, Tel Aviv Medical Center, Sackler School of Medicine, Tel Aviv University; O. Elkayam, M.D, Department of Rheumatology, Tel Aviv Medical Center, Sackler School of Medicine, Tel Aviv University.

Madar-Balakirski, Noa. From the Department of Rheumatology, Tel Aviv Medical Center, Sackler School of Medicine, Tel Aviv University, Tel Aviv, Israel.A. Broyde, MD, Department of Rheumatology, Tel Aviv Medical Center, Sackler School of Medicine, Tel Aviv University; U. Arad, MD, PhD, Department of Rheumatology, Tel Aviv Medical Center, Sackler School of Medicine, Tel Aviv University; N. Madar-Balakirski, PhD, Department of Rheumatology, Tel Aviv Medical Center, Sackler School of Medicine, Tel Aviv University; D. Paran, MD, Department of Rheumatology, Tel Aviv Medical Center, Sackler School of Medicine, Tel Aviv University; I. Kaufman, MD, Department of Rheumatology, Tel Aviv Medical Center, Sackler School of Medicine, Tel Aviv University; D. Levartovsky, MD, Department of Rheumatology, Tel Aviv Medical Center, Sackler School of Medicine, Tel Aviv University; I. Wigler, MD, Department of Rheumatology, Tel Aviv Medical Center, Sackler School of Medicine, Tel Aviv University; D. Caspi, MD, Department of Rheumatology, Tel Aviv Medical Center, Sackler School of Medicine, Tel Aviv University; O. Elkayam, M.D, Department of Rheumatology, Tel Aviv Medical Center, Sackler School of Medicine, Tel Aviv University.

Paran, Daphna. From the Department of Rheumatology, Tel Aviv Medical Center, Sackler School of Medicine, Tel Aviv University, Tel Aviv, Israel.A. Broyde, MD, Department of Rheumatology, Tel Aviv Medical Center, Sackler School of Medicine, Tel Aviv University; U. Arad, MD, PhD, Department of Rheumatology, Tel Aviv Medical Center, Sackler School of Medicine, Tel Aviv University; N. Madar-Balakirski, PhD, Department of Rheumatology, Tel Aviv Medical Center, Sackler School of Medicine, Tel Aviv University; D. Paran, MD, Department of Rheumatology, Tel Aviv Medical Center, Sackler School of Medicine, Tel Aviv University; I. Kaufman, MD, Department of Rheumatology, Tel Aviv Medical Center, Sackler School of Medicine, Tel Aviv University; D. Levartovsky, MD, Department of Rheumatology, Tel Aviv Medical Center, Sackler School of Medicine, Tel Aviv University; I. Wigler, MD, Department of Rheumatology, Tel Aviv Medical Center, Sackler School of Medicine, Tel Aviv University; D. Caspi, MD, Department of Rheumatology, Tel Aviv Medical Center, Sackler School of Medicine, Tel Aviv University; O. Elkayam, M.D, Department of Rheumatology, Tel Aviv Medical Center, Sackler School of Medicine, Tel Aviv University.

Kaufman, Ilana. From the Department of Rheumatology, Tel Aviv Medical Center, Sackler School of Medicine, Tel Aviv University, Tel Aviv, Israel.A. Broyde, MD, Department of Rheumatology, Tel Aviv Medical Center, Sackler School of Medicine, Tel Aviv University; U. Arad, MD, PhD, Department of Rheumatology, Tel Aviv Medical Center, Sackler School of Medicine, Tel Aviv University; N. Madar-Balakirski, PhD, Department of Rheumatology, Tel Aviv Medical Center, Sackler School of Medicine, Tel Aviv University; D. Paran, MD, Department of Rheumatology, Tel Aviv Medical Center, Sackler School of Medicine, Tel Aviv University; I. Kaufman, MD, Department of Rheumatology, Tel Aviv Medical Center, Sackler School of Medicine, Tel Aviv University; D. Levartovsky, MD, Department of Rheumatology, Tel Aviv Medical Center, Sackler School of Medicine, Tel Aviv University; I. Wigler, MD, Department of Rheumatology, Tel Aviv Medical Center, Sackler School of Medicine, Tel Aviv University; D. Caspi, MD, Department of Rheumatology, Tel Aviv Medical Center, Sackler School of Medicine, Tel Aviv University; O. Elkayam, M.D, Department of Rheumatology, Tel Aviv Medical Center, Sackler School of Medicine, Tel Aviv University.

Levartovsky, David. From the Department of Rheumatology, Tel Aviv Medical Center, Sackler School of Medicine, Tel Aviv University, Tel Aviv, Israel.A. Broyde, MD, Department of Rheumatology, Tel Aviv Medical Center, Sackler School of Medicine, Tel Aviv University; U. Arad, MD, PhD, Department of Rheumatology, Tel Aviv Medical Center, Sackler School of Medicine, Tel Aviv University; N. Madar-Balakirski, PhD, Department of Rheumatology, Tel Aviv Medical Center, Sackler School of Medicine, Tel Aviv University; D. Paran, MD, Department of Rheumatology, Tel Aviv Medical Center, Sackler School of Medicine, Tel Aviv University; I. Kaufman, MD, Department of Rheumatology, Tel Aviv Medical Center, Sackler School of Medicine, Tel Aviv University; D. Levartovsky, MD, Department of Rheumatology, Tel Aviv

Medical Center, Sackler School of Medicine, Tel Aviv University; I. Wigler, MD, Department of Rheumatology, Tel Aviv Medical Center, Sackler School of Medicine, Tel Aviv University; D. Caspi, MD, Department of Rheumatology, Tel Aviv Medical Center, Sackler School of Medicine, Tel Aviv University; O. Elkayam, M.D, Department of Rheumatology, Tel Aviv Medical Center, Sackler School of Medicine, Tel Aviv University.

Wigler, Irena. From the Department of Rheumatology, Tel Aviv Medical Center, Sackler School of Medicine, Tel Aviv University, Tel Aviv, Israel.A. Broyde, MD, Department of Rheumatology, Tel Aviv Medical Center, Sackler School of Medicine, Tel Aviv University; U. Arad, MD, PhD, Department of Rheumatology, Tel Aviv Medical Center, Sackler School of Medicine, Tel Aviv University; N. Madar-Balakirski, PhD, Department of Rheumatology, Tel Aviv Medical Center, Sackler School of Medicine, Tel Aviv University; D. Paran, MD, Department of Rheumatology, Tel Aviv Medical Center, Sackler School of Medicine, Tel Aviv University; I. Kaufman, MD, Department of Rheumatology, Tel Aviv Medical Center, Sackler School of Medicine, Tel Aviv University; D. Levartovsky, MD, Department of Rheumatology, Tel Aviv Medical Center, Sackler School of Medicine, Tel Aviv University; I. Wigler, MD, Department of Rheumatology, Tel Aviv Medical Center, Sackler School of Medicine, Tel Aviv University; D. Caspi, MD, Department of Rheumatology, Tel Aviv Medical Center, Sackler School of Medicine, Tel Aviv University; O. Elkayam, M.D, Department of Rheumatology, Tel Aviv Medical Center, Sackler School of Medicine, Tel Aviv University.

Caspi, Dan. From the Department of Rheumatology, Tel Aviv Medical Center, Sackler School of Medicine, Tel Aviv University, Tel Aviv, Israel.A. Broyde, MD, Department of Rheumatology, Tel Aviv Medical Center, Sackler School of Medicine, Tel Aviv University; U. Arad, MD, PhD, Department of Rheumatology, Tel Aviv Medical Center, Sackler School of Medicine, Tel Aviv University; N. Madar-Balakirski, PhD, Department of Rheumatology, Tel Aviv Medical Center, Sackler School of Medicine, Tel Aviv University; D. Paran, MD, Department of Rheumatology, Tel Aviv Medical Center, Sackler School of Medicine, Tel Aviv University; I. Kaufman, MD, Department of Rheumatology, Tel Aviv Medical Center, Sackler School of Medicine, Tel Aviv University; D. Levartovsky, MD, Department of Rheumatology, Tel Aviv Medical Center, Sackler School of Medicine, Tel Aviv University; I. Wigler, MD, Department of Rheumatology, Tel Aviv Medical Center, Sackler School of Medicine, Tel Aviv University; D. Caspi, MD, Department of Rheumatology, Tel Aviv Medical Center, Sackler School of Medicine, Tel Aviv University; O. Elkayam, M.D, Department of Rheumatology, Tel Aviv Medical Center, Sackler School of Medicine, Tel Aviv University.

Elkayam, Ori. From the Department of Rheumatology, Tel Aviv Medical Center, Sackler School of Medicine, Tel Aviv University, Tel Aviv, Israel.A. Broyde, MD, Department of Rheumatology, Tel Aviv Medical Center, Sackler School of Medicine, Tel Aviv University; U. Arad, MD, PhD, Department of Rheumatology, Tel Aviv Medical Center, Sackler School of Medicine, Tel Aviv

University; N. Madar-Balakirski, PhD, Department of Rheumatology, Tel Aviv Medical Center, Sackler School of Medicine, Tel Aviv University; D. Paran, MD, Department of Rheumatology, Tel Aviv Medical Center, Sackler School of Medicine, Tel Aviv University; I. Kaufman, MD, Department of Rheumatology, Tel Aviv Medical Center, Sackler School of Medicine, Tel Aviv University; D. Levartovsky, MD, Department of Rheumatology, Tel Aviv Medical Center, Sackler School of Medicine, Tel Aviv University; I. Wigler, MD, Department of Rheumatology, Tel Aviv Medical Center, Sackler School of Medicine, Tel Aviv University; D. Caspi, MD, Department of Rheumatology, Tel Aviv Medical Center, Sackler School of Medicine, Tel Aviv University; O. Elkayam, M.D, Department of Rheumatology, Tel Aviv Medical Center, Sackler School of Medicine, Tel Aviv University. oribe14@netvision.net.il.

Comments

Comment in (CIN)

Abstract

OBJECTIVE: To estimate the longterm humoral response of an antipneumococcal polysaccharide vaccine (PPSV23) in patients with rheumatoid arthritis (RA), psoriatic arthritis (PsA), ankylosing spondylitis (AS), or inflammatory bowel disease (IBD)-associated spondyloarthropathy (SpA), and the effect of demographic and clinical factors and treatment on the longterm efficacy of the vaccine.

METHODS: A total of 145 consecutive patients treated with biologics [tumor necrosis factor-alpha (TNF-alpha) or interleukin 6 (IL-6) receptor inhibitors] or methotrexate (MTX) participated in this study. Fifteen were excluded because of absent information regarding their vaccination status (n = 9) or because of technical problems in obtaining their serum sample (n = 6). They were diagnosed with RA (n = 63, 48.5%), PsA (n = 29, 22.3%), AS (n = 28, 21.5%), or IBD-associated SpA (n = 3, 2.3%). Their mean age was 54.6 years, and 61.5% were women. Data were collected on the timing of vaccination, demographic and clinical characteristics, and treatment, and patients' serum antipneumococcal antibody levels were tested.

RESULTS: Two-thirds of the patients (67.7%) had received PPSV23 45 months (mean) earlier. Treatment included TNF-alpha inhibitors (73.9%), IL-6 receptor inhibitors (13.1%), or MTX without a biological treatment (13%). The uptake of vaccination was significantly higher in the older population (> 65 yrs). Vaccinated patients had significantly higher antibody levels compared with vaccine-naive patients. The antibody levels had been preserved after 10 years. MTX use, but not biologics, was associated with significantly lower antibody levels.

CONCLUSION: The longterm efficacy of the PPSV23 vaccination seems to be preserved among patients with RA, PsA, AS, and IBD-associated SpA for at least 10 years. Efficacy is

slightly impaired by MTX, but it is not affected by biologics. These findings suggest that revaccination after 5 years might not be needed for all, and testing the antibody titers should be considered to identify those who may benefit from revaccination.

Publication Type

Journal Article.

Year of Publication

2016

<274>

Unique Identifier

26761790

Title

Endothelial Dysfunction in Rheumatoid Arthritis: Mechanistic Insights and Correlation with Circulating Markers of Systemic Inflammation.

Source

PLoS ONE [Electronic Resource]. 11(1):e0146744, 2016.

VI 1

Status

MEDLINE

Authors

Totoson P; Maguin-Gate K; Nappey M; Wendling D; Demougeot C.

Authors Full Name

Totoson, Perle; Maguin-Gate, Katy; Nappey, Maude; Wendling, Daniel; Demougeot, Celine.

Institution

Totoson, Perle. EA 4267 FDE, FHU INCREASE, Univ. Bourgogne Franche-Comte, Besancon, France.

Maguin-Gate, Katy. EA 4267 FDE, FHU INCREASE, Univ. Bourgogne Franche-Comte, Besancon, France.

Nappey, Maude. EA 4267 FDE, FHU INCREASE, Univ. Bourgogne Franche-Comte, Besancon, France.

Wendling, Daniel. Service de Rhumatologie, CHRU Besancon, Besancon, France.

Wendling, Daniel. EA 4266, Univ. Bourgogne Franche-Comte, Besancon, France.

Demougeot, Celine. EA 4267 FDE, FHU INCREASE, Univ. Bourgogne Franche-Comte, Besancon, France.
Comments
Erratum in (EIN)
Abstract
OBJECTIVES: To determine mechanisms involved in endothelial dysfunction (ED) during the course of arthritis and to investigate the link between cytokines, chemokines and osteoprotegerin.

APPROACH AND RESULTS: Experiments were conducted on aortic rings at day 4 (preclinical), day 11 (onset of disease), day 33 (acute disease) and day 90 (chronic disease) after adjuvant-induced arthritis (AIA) in Lewis rats. At day 4, the unique vascular abnormality was a reduced norepinephrine-induced constriction. At day 11, endothelial function assessed by the relaxation to acetylcholine was normal despite increased cyclo-oxygenase-2 activity (COX-2) and overproduction of superoxide anions that was compensated by increased nitric oxide synthase (NOS) activity. At day 33, ED apparition coincides with the normalization of NOS activity. At day 90, ED was only observed in rats with a persisting imbalance between endothelial NOS and COX-2 pathways and higher plasma levels of IL-1beta and TNFalpha. Plasma levels of IL-1beta, TNFalpha and MIP-1alpha negatively correlated with Ach-induced relaxation throughout the course of AIA.

CONCLUSIONS: Our data identified increased endothelial NOS activity as an important compensatory response that opposes the ED in the early arthritis. Thereafter, a cross-talk between endothelial COX-2/NOS pathways appears as an important element for the occurrence of ED. Our results encourage determining the clinical value of IL-1beta, TNFalpha and MIP-1alpha as biomarkers of ED in RA.
Publication Type
Journal Article. Research Support, Non-U.S. Gov't.
Year of Publication
2016

<275>
Unique Identifier
26721805

Title

Disentangling the effects of tocilizumab on neutrophil survival and function.

Source

Immunologic Research. 64(3):665-76, 2016 06.

VI 1

Status

MEDLINE

Authors

Gaber T; Hahne M; Strehl C; Hoff P; Dorffel Y; Feist E; Burmester GR; Buttgereit F.

Author NameID

Gaber, Timo; ORCID: https://orcid.org/0000-0002-6675-3895

Authors Full Name

Gaber, Timo; Hahne, Martin; Strehl, Cindy; Hoff, Paula; Dorffel, Yvonne; Feist, Eugen; Burmester, Gerd-Rudiger; Buttgereit, Frank.

Institution

Gaber, Timo. Department of Rheumatology and Clinical Immunology, Charite University Hospital, Charite University Medicine, Chariteplatz 1, 10117, Berlin, Germany. timo.gaber@charite.de.

Gaber, Timo. German Rheumatism Research Center (DRFZ), Chariteplatz 1, 10117, Berlin, Germany. timo.gaber@charite.de.

Gaber, Timo. Berlin-Brandenburg Center of Regenerative Therapies (BCRT), Augustenburger Platz 1, 13353, Berlin, Germany. timo.gaber@charite.de.

Hahne, Martin. Department of Rheumatology and Clinical Immunology, Charite University Hospital, Charite University Medicine, Chariteplatz 1, 10117, Berlin, Germany.

Hahne, Martin. German Rheumatism Research Center (DRFZ), Chariteplatz 1, 10117, Berlin, Germany.

Hahne, Martin. Berlin-Brandenburg School of Regenerative Therapies (BSRT), Augustenburger Platz 1, 13353, Berlin, Germany.

Strehl, Cindy. Department of Rheumatology and Clinical Immunology, Charite University Hospital, Charite University Medicine, Chariteplatz 1, 10117, Berlin, Germany.

Strehl, Cindy. German Rheumatism Research Center (DRFZ), Chariteplatz 1, 10117, Berlin, Germany.

Hoff, Paula. Department of Rheumatology and Clinical Immunology, Charite University Hospital, Charite University Medicine, Chariteplatz 1, 10117, Berlin, Germany.

Hoff, Paula. German Rheumatism Research Center (DRFZ), Chariteplatz 1, 10117, Berlin, Germany.

Dorffel, Yvonne. Department of Gastroenterology and Hepatology, Charite University Medicine, Chariteplatz 1, 10117, Berlin, Germany.
Feist, Eugen. Department of Rheumatology and Clinical Immunology, Charite University Hospital, Charite University Medicine, Chariteplatz 1, 10117, Berlin, Germany.
Burmester, Gerd-Rudiger. Department of Rheumatology and Clinical Immunology, Charite University Hospital, Charite University Medicine, Chariteplatz 1, 10117, Berlin, Germany.
Buttgereit, Frank. Department of Rheumatology and Clinical Immunology, Charite University Hospital, Charite University Medicine, Chariteplatz 1, 10117, Berlin, Germany.
Buttgereit, Frank. German Rheumatism Research Center (DRFZ), Chariteplatz 1, 10117, Berlin, Germany.
Buttgereit, Frank. Berlin-Brandenburg Center of Regenerative Therapies (BCRT), Augustenburger Platz 1, 13353, Berlin, Germany.
Abstract
The synovial tissue in rheumatoid arthritis (RA) represents a hypoxic environment with up-regulated pro-inflammatory cytokines and cellular infiltrates including neutrophils. Although inhibition of the interleukin (IL)6 receptor pathway by tocilizumab is a potent treatment option for RA, it may also cause adverse effects such as an occasionally high-grade neutropenia. We analysed the impact of tocilizumab on survival, mediator secretion, oxidative burst, phagocytosis and energy availability of high-dose toll-like receptor (TLR)2/4-stimulated neutrophils (to mimic an arthritis flare) under normoxic versus hypoxic conditions. Human neutrophils were purified, pre-treated with varying doses of tocilizumab, dexamethasone or human IgG1 and high-dose-stimulated with lipopolysaccharide (LPS) alone-triggering TLR2/4-, LPS plus IL6, or left unstimulated. Cells were then incubated under normoxic (18 % O2) or hypoxic (1 % O2) conditions and subsequently analysed. Neutrophil survival and energy availability were significantly decreased by tocilizumab in a dose-dependent manner in high-dose TLR2/4-stimulated cells, but to a greater extent under normoxia as compared to hypoxia. We also found high-dose LPS-stimulated oxidative burst and phagocytosis of neutrophils to be higher under hypoxic versus normoxic conditions, but this difference was reduced by tocilizumab. Finally, we observed that tocilizumab affected neutrophil mediator secretion as a function of oxygen availability. Tocilizumab is known for both beneficial effects and a higher incidence of neutropenia when treating RA patients. Our results suggest that both effects can at least in part be explained by a reduction in neutrophil survival, a dose-dependent inhibition of hypoxia-induced NADPH oxidase-mediated oxidative burst and phagocytosis of infiltrating hypoxic neutrophils and an alteration of mediator secretion.
Publication Type
Journal Article. Research Support, Non-U.S. Gov't.
Year of Publication

2016

<276>

Unique Identifier

26718689

Title

Tick-borne encephalitis (TBE) vaccine to medically immunosuppressed patients with rheumatoid arthritis: A prospective, open-label, multi-centre study.

Source

Vaccine. 34(5):650-655, 2016 Jan 27.

VI 1

Status

MEDLINE

Authors

Hertzell KB; Pauksens K; Rombo L; Knight A; Vene S; Askling HH.

Authors Full Name

Hertzell, Katarina Brodin; Pauksens, Karlis; Rombo, Lars; Knight, Ann; Vene, Sirkka; Askling, Helena H.

Institution

Hertzell, Katarina Brodin. Karolinska Institutet, Dept. of Medicine/Solna, Unit for Infectious Diseases, SE 17176 Stockholm, Sweden. Electronic address: katarina.brodin-hertzell@capiostgoran.se.

Pauksens, Karlis. Department of Medical Sciences, Section of Infectious Diseases, Uppsala University, SE 751 85 Uppsala, Sweden.

Rombo, Lars. Karolinska Institutet, Dept. of Medicine/Solna, Unit for Infectious Diseases, SE 17176 Stockholm, Sweden; Centre for Clinical Research, Sormland, Uppsala University, SE 631 88 Eskilstuna, Sweden. Electronic address: lars.rombo@gmail.com.

Knight, Ann. Dept. of Rheumatology, Inst. of Medical Sciences, Uppsala University, SE 751 85 Uppsala, Sweden. Electronic address: ann.knight@akademiska.se.

Vene, Sirkka. Public Health Agency of Sweden, Nobels vag 18, SE 17182 Solna, Sweden. Electronic address: sirkka@vene.se.

Askling, Helena H. Karolinska Institutet, Dept. of Medicine/Solna, Unit for Infectious Diseases, SE 17176 Stockholm, Sweden; Dept. of Communicable Diseases Control and Prevention, Box 17533, SE-11891 Stockholm, Sweden. Electronic address: helena.hervius-askling@ki.se.
Abstract
BACKGROUND: Tick-borne Encephalitis (TBE) is endemic in south-eastern Sweden as well as in the Baltic regions, Central Europe and Russia. Ageing and immunosuppressed individuals are more prone to severe disease and neurological complications. We assessed the immunogenicity of TBE-vaccine in rheumatoid arthritis (RA) patients treated with tumor necrosis factor-inhibitors (TNFi) and/or methotrexate (MTX).

METHODS: TBE vaccine, FSME-Immune(R) or Encepur(R), was administered to non-immune RA patients as well as age and gender matched healthy controls. Individuals <60 years of age were given three doses at month 0, 1, 12. Individuals >= 60 years old were given an additional priming dose at month 3, i.e. a total of four doses. Tick-borne encephalitis neutralizing antibodies were assessed by a rapid fluorescent focus inhibition test.

RESULTS: The study population consisted of 66 patients and 56 age and gender matched healthy controls. Median age was 58.5 years. The patients were either treated with TNFi (n=16), TNFi+MTX (n=36) or MTX (n=14). After the last TBE-vaccine dose, given one year after the first, 39% of the patients compared to 79% of the healthy controls had seroprotective levels (p=<0.05).

CONCLUSIONS: Standard TBE-vaccine schedule does not confer enough immunogenicity in this group of immunosuppressed patients, who should be carefully informed about a higher risk for vaccination failure and risk of infection when exposed in high-endemic areas. Copyright © 2015 Elsevier Ltd. All rights reserved.
Publication Type
Journal Article. Multicenter Study.
Year of Publication
2016

<277>
Unique Identifier
26688003

Title

Blood and salivary-gland BAFF-driven B-cell hyperactivity is associated to rituximab inefficacy in primary Sjogren's syndrome.

Source

Journal of Autoimmunity. 67:102-110, 2016 Feb.

VI 1

Status

MEDLINE

Authors

Cornec D; Costa S; Devauchelle-Pensec V; Jousse-Joulin S; Marcorelles P; Berthelot JM; Chiche L; Hachulla E; Hatron PY; Goeb V; Vittecoq O; Saraux A; Pers JO.

Authors Full Name

Cornec, Divi; Costa, Sebastian; Devauchelle-Pensec, Valerie; Jousse-Joulin, Sandrine; Marcorelles, Pascale; Berthelot, Jean-Marie; Chiche, Laurent; Hachulla, Eric; Hatron, Pierre-Yves; Goeb, Vincent; Vittecoq, Olivier; Saraux, Alain; Pers, Jacques-Olivier.

Institution

Cornec, Divi. Service de Rhumatologie, Hopital de la Cavale Blanche, CHRU Brest, Boulevard Tanguy Prigent, Brest 29609, France; EA2216, INSERM ESPRI, ERI29, Laboratoire d'Immunotherapies et Pathologies lymphocytaires B, Universite de Brest, and Labex "IGO", Brest, France.

Costa, Sebastian. Laboratoire d'Anatomie Pathologique et Cytologie, Hopital Morvan, CHRU Brest, Avenue Foch, Brest 29609, France.

Devauchelle-Pensec, Valerie. Service de Rhumatologie, Hopital de la Cavale Blanche, CHRU Brest, Boulevard Tanguy Prigent, Brest 29609, France; EA2216, INSERM ESPRI, ERI29, Laboratoire d'Immunotherapies et Pathologies lymphocytaires B, Universite de Brest, and Labex "IGO", Brest, France.

Jousse-Joulin, Sandrine. Service de Rhumatologie, Hopital de la Cavale Blanche, CHRU Brest, Boulevard Tanguy Prigent, Brest 29609, France; EA2216, INSERM ESPRI, ERI29, Laboratoire d'Immunotherapies et Pathologies lymphocytaires B, Universite de Brest, and Labex "IGO", Brest, France.

Marcorelles, Pascale. Laboratoire d'Anatomie Pathologique et Cytologie, Hopital Morvan, CHRU Brest, Avenue Foch, Brest 29609, France.

Berthelot, Jean-Marie. Service de Rhumatologie, Hotel-Dieu, CHU Nantes, Nantes Cedex 01 44093, France.

Chiche, Laurent. Service de Medecine Interne, Hopital Europeen, Marseille, France.

Hachulla, Eric. Service de Medecine Interne, Claude Huriez Hospital, Universite Lille Nord-de-France, Lille Cedex 59037, France.

Hatron, Pierre-Yves. Service de Medecine Interne, Claude Huriez Hospital, Universite Lille Nord-de-France, Lille Cedex 59037, France.
Goeb, Vincent. Service de Rhumatologie, Hopital Nord, CHU Amiens, Amiens 80054, France.
Vittecoq, Olivier. Service de Rhumatologie, CHRU de Rouen, Bois-Guillaume 76230, France.
Saraux, Alain. Service de Rhumatologie, Hopital de la Cavale Blanche, CHRU Brest, Boulevard Tanguy Prigent, Brest 29609, France; EA2216, INSERM ESPRI, ERI29, Laboratoire d'Immunotherapies et Pathologies lymphocytaires B, Universite de Brest, and Labex "IGO", Brest, France.
Pers, Jacques-Olivier. EA2216, INSERM ESPRI, ERI29, Laboratoire d'Immunotherapies et Pathologies lymphocytaires B, Universite de Brest, and Labex "IGO", Brest, France. Electronic address: pers@univ-brest.fr.
Abstract
OBJECTIVES: To determine whether B-cell markers (blood and minor salivary gland [SG] B-cell depletion [BCD], autoantibodies, B-cell-activating factor [BAFF]) are associated with clinical response to rituximab in patients with primary Sjogren's syndrome (pSS).

METHODS: 45 patients with pSS were included: in group I, 14 received low-dose rituximab (two 375-mg/m(2) infusions) in an open-labelled study; in group II, 17 received full-dose rituximab (two 1000-mg infusions) and 14 received a placebo in a randomized, controlled study. The proportion of SG B cells was assessed using pixel-based software analyses of digitized double-immunostained (CD3/CD20) whole SGs. Response was defined at week-24 according to the Sjogren's Syndrome Responder Index (SSRI)-30.

RESULTS: Response rate was 50% in both groups of rituximab-treated patients. Duration of blood BCD was similar in both groups despite the difference in rituximab dosage, and was highly correlated with residual serum-rituximab levels at week-16. SG B-cell dynamics mirrored blood B-cell levels, with a drastic decrease in SG B-cells at week-12 (group I), but an increase in ~ 50% of patients in group II by week-24, in whom blood B cells had already returned. Duration of BCD was not associated with the clinical response, but responders had lower baseline proportions of SG B cells. Baseline serum BAFF level was correlated with the proportion of SG B-cells and other B-cell-activation markers, and was associated with the clinical response with higher levels in non-responders.

CONCLUSIONS: In pSS, half of the patients display an intense BAFF-driven B-cell activation and do not respond to a single course of rituximab. Copyright © 2015 Elsevier Ltd. All rights reserved.
Publication Type

Journal Article. Research Support, Non-U.S. Gov't.
Year of Publication
2016

<278>
Unique Identifier
26679631
Title
CTLA-4 blockade in the treatment of rheumatoid arthritis: an update. [Review]
Source
Expert Review of Clinical Immunology. 12(4):417-25, 2016.
VI 1
Status
MEDLINE
Authors
Cutolo M; Sulli A; Paolino S; Pizzorni C.
Authors Full Name
Cutolo, Maurizio; Sulli, Alberto; Paolino, Sabrina; Pizzorni, Carmen.
Institution
Cutolo, Maurizio. a Research Laboratories and Academic Division of Clinical Rheumatology, Postgraduate School of Rheumatology, Department of Internal Medicine, University of Genova, Genova, Italy.
Sulli, Alberto. a Research Laboratories and Academic Division of Clinical Rheumatology, Postgraduate School of Rheumatology, Department of Internal Medicine, University of Genova, Genova, Italy.
Paolino, Sabrina. a Research Laboratories and Academic Division of Clinical Rheumatology, Postgraduate School of Rheumatology, Department of Internal Medicine, University of Genova, Genova, Italy.
Pizzorni, Carmen. a Research Laboratories and Academic Division of Clinical Rheumatology, Postgraduate School of Rheumatology, Department of Internal Medicine, University of Genova, Genova, Italy.
Abstract

Rheumatoid arthritis (RA) is characterized by chronic joint inflammation as well as by extra-articular involvement. The immunopathology of RA is polygenic and involves different cell populations. Patients with an inadequate response to non-biologic disease- modifying antirheumatic drugs (DMARDs) should integrate their therapy with biologic DMARDs. Biologic DMARDs can target several inflammatory cytokines, or CD20+ B cells, or can modulate T-cell co-stimulation and activation. The cytotoxic T lymphocyte-associated antigen 4 immunoglobulin fusion protein (CTLA-4-Ig: abatacept) that selectively modulates the CD28:CD80/86 co-stimulation signal appears a biologic DMARD interacting with T cells but also with other cell populations involved in RA pathophysiology. Activated B lymphocytes, macrophages, osteoclasts and endothelial cells express the costimulatory molecules (CD80/86) and are downregulated by CTLA-4 blockade. The relatively low frequency and severity of safety issues related to CTLA-4-Ig treatment seems further to confirm the targeted downregulatory action exerted by the fusion protein, which is mainly focussed on activated immune/inflammatory cells.

Publication Type

Journal Article. Review.

Year of Publication

2016

<279>

Unique Identifier

26642378

Title

IL-6 blockers in systemic onset juvenile idiopathic arthritis. [Review]

Source

Immunotherapy. 8(1):79-87, 2016.

VI 1

Status

MEDLINE

Authors

Barone P; Pignataro R; Garozzo MT; Leonardi S.

Authors Full Name

Barone, Patrizia; Pignataro, Rossana; Garozzo, Maria Teresa; Leonardi, Salvatore.

Institution

Barone, Patrizia. Department of Clinical & Experimental Medicine, University of Catania, Italy.
Pignataro, Rossana. Department of Clinical & Experimental Medicine, University of Catania, Italy.
Garozzo, Maria Teresa. Department of Clinical & Experimental Medicine, University of Catania, Italy.
Leonardi, Salvatore. Department of Clinical & Experimental Medicine, University of Catania, Italy.
Abstract
IL-6 has a key role in the pathogenesis, clinical manifestations and activity of Systemic Onset Idiopathic Arthritis (sJIA). Tocilizumab (TCZ), the first humanized antihuman IL-6 receptor antibody, inhibits the activity of IL-6. In this review, we summarize the main studies performed, to date, about the use of TCZ in children affected by sJIA refractory to conventional treatment. Nowadays TCZ can be used, alone or in association with Metotrexate, in children older than 2 years. Its use in children younger than 2 years is being investigated. Further study about its use in sJIA and other type of idiopathic arthritis should be done.
Publication Type
Journal Article. Review.
Year of Publication
2016

<280>
Unique Identifier
26558442
Title
Interleukin 17 inhibits progenitor cells in rheumatoid arthritis cartilage.
Source
European Journal of Immunology. 46(2):440-5, 2016 Feb.
VI 1
Status
MEDLINE
Authors
Schminke B; Trautmann S; Mai B; Miosge N; Blaschke S.
Authors Full Name

Schminke, Boris; Trautmann, Sandra; Mai, Burkhard; Miosge, Nicolai; Blaschke, Sabine.
Institution
Schminke, Boris. Department of Prosthodontics, Tissue Regeneration Work Group, University Medical Center, Goettingen, Germany.
Trautmann, Sandra. Department of Nephrology and Rheumatology, University Medical Center, Goettingen, Germany.
Mai, Burkhard. VitosOrthopaedic Clinic, Kassel, Germany.
Miosge, Nicolai. Department of Prosthodontics, Tissue Regeneration Work Group, University Medical Center, Goettingen, Germany.
Blaschke, Sabine. Department of Nephrology and Rheumatology, University Medical Center, Goettingen, Germany.
Abstract
Mesenchymal stem cells are known to exert immunomodulatory effects in inflammatory diseases. Immuneregulatory cells lead to progressive joint destruction in rheumatoid arthritis (RA). Proinflammatory cytokines, such as tumour necrosis factor alpha (TNF-alpha) and interleukins (ILs) are the main players. Here, we studied progenitor cells from RA cartilage (RA-CPCs) that are positive for IL-17 receptors to determinate the effects of inflammation on their chondrogenic potenial. IL-17A/F reduced the chondrogenic potential of these cells via the upregulation of RUNX2 protein and enhanced IL-6 protein and MMP3 mRNA levels. Blocking antibodies against IL-17 positively influenced their repair potential. Furthermore, treating the RA-CPCs with the anti-human IL-17 antibody secukinumab or the anti-TNF-alpha antibody adalimumab reduced the proinflammatory IL-6 protein level and positively influenced the secretion of anti-inflammatory IL-10 protein. Additionally, adalimumab and secukinumab in particular reduced RUNX2 protein to promote chondrogenesis. The amelioration of inflammation, particularly via IL-17 antagonism, might be a new therapeutic approach for enhancing intrinsic cartilage repair mechanisms in RA patients. Copyright © 2015 The Authors. European Journal of Immunology published by WILEY-VCH Verlag GmbH & Co. KGaA, Weinheim.
Publication Type
Journal Article. Research Support, Non-U.S. Gov't.
Year of Publication
2016

<281>

Unique Identifier
  26510559
Title
  Immunotherapies for Neurological Manifestations in the Context of Systemic Autoimmunity.
[Review]
Source
  Neurotherapeutics. 13(1):163-78, 2016 Jan.
VI 1
Status
  MEDLINE
Authors
  Kampylafka EI; Alexopoulos H; Dalakas MC; Tzioufas AG.
Authors Full Name
  Kampylafka, Eleni I; Alexopoulos, Harry; Dalakas, Marinos C; Tzioufas, Athanasios G.
Institution
  Kampylafka, Eleni I. Department of Pathophysiology, Faculty of Medicine, National and Kapodistrian University of Athens, Athens, 11527, Greece.
  Alexopoulos, Harry. Department of Pathophysiology, Faculty of Medicine, National and Kapodistrian University of Athens, Athens, 11527, Greece.
  Dalakas, Marinos C. Department of Pathophysiology, Faculty of Medicine, National and Kapodistrian University of Athens, Athens, 11527, Greece.
  Tzioufas, Athanasios G. Department of Pathophysiology, Faculty of Medicine, National and Kapodistrian University of Athens, Athens, 11527, Greece. agtzi@med.uoa.gr.
Abstract
  Neurological involvement is relatively common in the majority of systemic autoimmune diseases and may lead to severe morbidity and mortality, if not promptly treated. Treatment options vary greatly, depending on the underlying systemic pathophysiology and the associated neurological symptoms. Selecting the appropriate therapeutic scheme is further complicated by the lack of definite therapeutic guidelines, the necessity to differentiate primary neurological syndromes from those related to the underlying systemic disease, and to sort out adverse neurological manifestations caused by immunosuppressants or the biological agents used to treat the primary disease. Immunotherapy is a sine qua non for treating most, if not all, neurological conditions presenting in the context of systemic autoimmunity. Specific agents include classical immune modulators such as corticosteroids, cyclophosphamide, intravenous immunoglobulin, and plasma exchange, as well as numerous biological therapies, for example anti-tumor necrosis factor agents and monoclonal antibodies that target various immune pathways such as B cells, cytokines, and co-stimulatory molecules. However, experience regarding the use of these agents

in neurological complications of systemic diseases is mainly empirical or based on small uncontrolled studies and case series. The aim of this review is to present the state-of-the-art therapies applied in various neurological manifestations encountered in the context of systemic autoimmune diseases; evaluate all treatment options on the basis of existing guidelines; and compliment these data with our personal experience derived from a large number of patients.

Publication Type

Journal Article. Review.

Year of Publication

2016

<282>

Unique Identifier

26471922

Title

The safety and effectiveness of HBV vaccination in patients with juvenile idiopathic arthritis controlled by treatment.

Source

Modern Rheumatology. 26(3):368-71, 2016.

VI 1

Status

MEDLINE

Authors

Nerome Y; Akaike H; Nonaka Y; Takezaki T; Kubota T; Yamato T; Yamasaki Y; Imanaka H; Kawano Y; Takei S.

Authors Full Name

Nerome, Yasuhito; Akaike, Harumi; Nonaka, Yukiko; Takezaki, Tomoko; Kubota, Tomohiro; Yamato, Tsuyoshi; Yamasaki, Yuichi; Imanaka, Hiroyuki; Kawano, Yoshifumi; Takei, Syuji.

Institution

Nerome, Yasuhito. a Department of Pediatrics, Kagoshima University Graduate School of Medical and Dental Sciences, Kagoshima University, Kagoshima, Japan .

Nerome, Yasuhito. b Education Center for Doctors on Remote Islands and in Rural Areas, Kagoshima University Graduate School of Medical and Dental Sciences, Kagoshima, Japan, and.

Akaike, Harumi. a Department of Pediatrics, Kagoshima University Graduate School of Medical and Dental Sciences, Kagoshima University, Kagoshima, Japan .

Nonaka, Yukiko. a Department of Pediatrics, Kagoshima University Graduate School of Medical and Dental Sciences, Kagoshima University, Kagoshima, Japan .

Takezaki, Tomoko. a Department of Pediatrics, Kagoshima University Graduate School of Medical and Dental Sciences, Kagoshima University, Kagoshima, Japan .

Kubota, Tomohiro. a Department of Pediatrics, Kagoshima University Graduate School of Medical and Dental Sciences, Kagoshima University, Kagoshima, Japan .

Yamato, Tsuyoshi. a Department of Pediatrics, Kagoshima University Graduate School of Medical and Dental Sciences, Kagoshima University, Kagoshima, Japan .

Yamasaki, Yuichi. a Department of Pediatrics, Kagoshima University Graduate School of Medical and Dental Sciences, Kagoshima University, Kagoshima, Japan .

Imanaka, Hiroyuki. a Department of Pediatrics, Kagoshima University Graduate School of Medical and Dental Sciences, Kagoshima University, Kagoshima, Japan .

Kawano, Yoshifumi. a Department of Pediatrics, Kagoshima University Graduate School of Medical and Dental Sciences, Kagoshima University, Kagoshima, Japan .

Takei, Syuji. a Department of Pediatrics, Kagoshima University Graduate School of Medical and Dental Sciences, Kagoshima University, Kagoshima, Japan .

Takei, Syuji. c Faculty of Medicine, School of Health Sciences, Kagoshima University, Kagoshima, Japan.

Abstract

OBJECTIVES: To evaluate the safety and effectiveness of hepatitis B virus (HBV) vaccination in patients with juvenile idiopathic arthritis (JIA) controlled by treatment.

METHODS: Among 49 patients with juvenile idiopathic arthritis (JIA) at the outpatient clinic of Kagoshima University Hospital, we enrolled 25 who were controlled by treatment. All children were unimmunized and were vaccinated against HBV according to the schedule. Their responses to the vaccine and vaccine adverse events were examined during their visits.

RESULTS: Nineteen of the 25 patients with JIA controlled by treatment developed effective antibody responses (76%). All eight patients with JIA below 10 years of age achieved seroconversion. The seroconversion was not influenced by biologics. Five adverse events were observed (6.7%). The rate of all adverse events did not surpass that of a previous report, and all adverse events were immediately resolved. None of the patients with JIA experienced a flare-up or clinical deterioration related to the vaccination.

CONCLUSIONS: HBV vaccination is safe and effective. Pediatric rheumatologists should consider HBV vaccination for unimmunized patients with JIA, because the response to HBV vaccine might be influenced by age, and children have a higher risk for potential HBV infection than adults.

Publication Type

Journal Article.

Year of Publication

2016

<283>

Unique Identifier

26453110

Title

Seasonal influenza vaccine coverage of patients on biotherapy for inflammatory joint disease in Normandy, France.

Source

Joint, Bone, Spine: Revue du Rhumatisme. 83(4):465-7, 2016 Jul.

VI 1

Status

MEDLINE

Authors

Michel M; Vincent FB; Rio S; Leon N; Marcelli C.

Authors Full Name

Michel, Murielle; Vincent, Fabien B; Rio, Simon; Leon, Nathalie; Marcelli, Christian.

Institution

Michel, Murielle. Service de rhumatologie, CHU de Caen, avenue de la Cote-de-Nacre, 14033 Caen cedex 9, France. Electronic address: michel-m@chu-caen.fr.

Vincent, Fabien B. Department of Immunology, Monash University, Central Clinical School, Alfred Medical Research and Education Precinct (AMREP), 89, Commercial Road, Melbourne, 3004 Victoria, Australia.

Rio, Simon. Service de rhumatologie, CHU de Caen, avenue de la Cote-de-Nacre, 14033 Caen cedex 9, France.

Leon, Nathalie. Service de rhumatologie, CHU de Caen, avenue de la Cote-de-Nacre, 14033 Caen cedex 9, France.
Marcelli, Christian. Service de rhumatologie, CHU de Caen, avenue de la Cote-de-Nacre, 14033 Caen cedex 9, France.
Publication Type
Letter.
Year of Publication
2016

<284>
Unique Identifier
26453106
Title
Vaccinations in adults with chronic inflammatory joint disease: Immunization schedule and recommendations for patients taking synthetic or biological disease-modifying antirheumatic drugs. [Review]
Source
Joint, Bone, Spine: Revue du Rhumatisme. 83(2):135-41, 2016 Mar.
VI 1
Status
MEDLINE
Authors
Morel J; Czitrom SG; Mallick A; Sellam J; Sibilia J.
Authors Full Name
Morel, Jacques; Czitrom, Severine Guillaume; Mallick, Auriane; Sellam, Jeremie; Sibilia, Jean.
Institution
Morel, Jacques. Departement de rhumatologie, CHRU, universite de Montpellier, 371, avenue du Doyen-Gaston-Giraud, 34295 Montpellier cedex 5, France. Electronic address: j-morel@chu-montpellier.fr.
Czitrom, Severine Guillaume. Service de medecine des adolescents, CHU Bicetre, AP-HP, 78, rue du General-Leclerc, 94270 Le Kremlin-Bicetre, France.
Mallick, Auriane. Service de rhumatologie, UMR_S1109, FMTS, universite de Strasbourg, 4, rue Blaise-Pascal, 67081 Strasbourg, France.

Sellam, Jeremie. Service de rhumatologie, hopital Saint-Antoine, AP-HP, Inserm UMRS_938, universite Paris 6, DHU i2B, 184, rue du Faubourg-Saint-Antoine, 75012 Paris, France.
Sibilia, Jean. Service de rhumatologie, UMR_S1109, FMTS, universite de Strasbourg, 4, rue Blaise-Pascal, 67081 Strasbourg, France.
Abstract
The risk of infection associated with autoimmune diseases is further increased by the use of biotherapies. Recommendations to minimize this risk include administering the full complement of vaccines on the standard immunization schedule, as well as the pneumococcal and influenza vaccines. Adults with chronic inflammatory joint disease (IJD) may receive a 13-valent pneumococcal conjugate vaccine, as well as a live attenuated vaccine against recurrent herpes zoster, recently licensed by European regulatory authorities. Live attenuated vaccines can be given only after an interval without immunosuppressant and/or glucocorticoid therapy. The effectiveness of vaccines, as assessed based on titers of protective antibodies, varies across vaccine types and disease-modifying antirheumatic drugs (DMARDs). Thus, methotrexate and rituximab are usually associated with decreased vaccine responses. The risks associated with vaccines are often considerably exaggerated by the media, which serve lobbies opposed to immunizations and make some patients reluctant to accept immunizations. Increasing immunization coverage may diminish the risk of treatment-related infections. A physician visit dedicated specifically to detecting comorbidities in patients with chronic IJD may result in improved immunization coverage. In this review, we discuss immunizations for adults with chronic IJD based on the treatments used, as well as immunization coverage. Many questions remain unanswered and warrant investigation by studies coordinated by the French networks IREIVAC (Innovative clinical research network in vaccinology) and IMIDIATE (Immune-Mediated Inflammatory Disease Alliance for Translational and Clinical Research). Copyright © 2015 Societe francaise de rhumatologie. Published by Elsevier SAS. All rights reserved.
Publication Type
Journal Article. Review.
Year of Publication
2016

<285>
Unique Identifier
26392009

Title

Spontaneous reports of vasculitis as an adverse event following immunization: A descriptive analysis across three international databases.

Source

Vaccine. 34(51):6634-6640, 2016 12 12.

VI 1

Status

MEDLINE

Authors

Felicetti P; Trotta F; Bonetto C; Santuccio C; Brauchli Pernus Y; Burgner D; Chandler R; Girolomoni G; Hadden RD; Kochhar S; Kucuku M; Monaco G; Ozen S; Pahud B; Phuong L; Bachtiar NS; Teeba A; Top K; Varricchio F; Wise RP; Zanoni G; Zivkovic S; Bonhoeffer J; Brighton Collaboration Vasculitis Working Group.

Authors Full Name

Felicetti, Patrizia; Trotta, Francesco; Bonetto, Caterina; Santuccio, Carmela; Brauchli Pernus, Yolanda; Burgner, David; Chandler, Rebecca; Girolomoni, Giampiero; Hadden, Robert D M; Kochhar, Sonali; Kucuku, Merita; Monaco, Giuseppe; Ozen, Seza; Pahud, Barbara; Phuong, Linny; Bachtiar, Novilia Sjafri; Teeba, Amina; Top, Karina; Varricchio, Frederick; Wise, Robert P; Zanoni, Giovanna; Zivkovic, Sasa; Bonhoeffer, Jan; Brighton Collaboration Vasculitis Working Group.

Institution

Felicetti, Patrizia. Italian Medicines Agency, Rome, Italy. Electronic address: contact@brightoncollaboration.org.

Trotta, Francesco. Italian Medicines Agency, Rome, Italy.

Bonetto, Caterina. Italian Medicines Agency, Rome, Italy.

Santuccio, Carmela. Italian Medicines Agency, Rome, Italy.

Brauchli Pernus, Yolanda. Brighton Collaboration Foundation, Basel, Switzerland.

Burgner, David. Monash Children's Hospital-Clayton, Melbourne, Australia; Murdoch Children's Research Institute (MCRI) - Department of Paediatrics, Melbourne University, Australia.

Chandler, Rebecca. Uppsala Monitoring Centre, Uppsala, Sweden.

Girolomoni, Giampiero. University of Verona, Department of Medicine, Section of Dermatology and Venereology, Verona, Italy.

Hadden, Robert D M. King's College Hospital, London, UK.

Kochhar, Sonali. USAID, Deliver Project, JSIPL, New Delhi, India.

Kucuku, Merita. Department of Vaccines Control, National Agency for Medicine & Medical Devices, Tirana, Albania.

Monaco, Giuseppe. Centre for Pharmacovigilance, The Lombardy Region, Milan, Italy.

Ozen, Seza. Hacettepe University, Department of Pediatric Rheumatology, Ankara, Turkey.
Pahud, Barbara. Children's Mercy Hospital, Kansas City, MO, USA.
Phuong, Linny. Monash Children's and Royal Children's Hospitals, Melbourne, Australia.
Bachtiar, Novilia Sjafri. Bio Farma Vaccine Institute, Bandung, West Java, Indonesia.
Teeba, Amina. Centre National Anti Poison et de Pharmacovigilance, Rabat, Morocco.
Top, Karina. Department of Pediatrics, Dalhousie University, Halifax, NS, Canada.
Varricchio, Frederick. Independent Consultant Vaccinologist, Wakefield, RI, USA.
Wise, Robert P. MedImmune/AstraZeneca, Gaithersburg, MD, USA.
Zanoni, Giovanna. Immunology Unit, Azienda Ospedaliera Universitaria Integrata Verona, Verona, Italy.
Zivkovic, Sasa. University of Pittsburgh Medical Center and Neurology service, MSL, VA Pittsburgh Healthcare System, Pittsburgh, PA, USA.
Bonhoeffer, Jan. University of Basel Children's Hospital, Basel, Switzerland; Brighton Collaboration Foundation, Basel, Switzerland.
Abstract
BACKGROUND: Vasculitides have been reported as adverse events following immunization (AEFI) following various vaccines. We describe reports of vasculitis to three international spontaneous reporting systems.

METHODS: All spontaneous reports of vasculitis following immunization between January 2003 and June 2014 were retrieved from Eudravigilance (EV), the Vaccine Adverse Event Reporting System (VAERS), and VigiBase R. A Standard MedDRA Query (SMQ) for vasculitis was used and vaccine types were categorized using the Anatomical Therapeutic Chemical classification system. We performed a descriptive analysis by source, sex, age, country, time to onset, vaccine, and type of vasculitis.

RESULTS: We retrieved 1797 reports of vasculitis in EV, 1171 in VAERS, and 2606 in VigiBase R. Vasculitis was predominantly reported in children aged 1-17 years, and less frequently in the elderly (>65 years). The generic term "vasculitis" was the most frequently reported AEFI in this category across the three databases (range 21.9% to 27.5% of all reported vasculitis for vaccines). For the more specific terms, Henoch-Schoenlein Purpura (HSP) was most frequently reported, (19.1% on average), followed by Kawasaki disease (KD) (16.1% on average) and polymyalgia rheumatica (PMR) (9.2% on average). Less frequently reported subtypes were cutaneous vasculitis (CuV), vasculitis of the central nervous system (CNS-V), and Behcet's syndrome (BS). HSP, PMR and CuV were more frequently reported with influenza vaccines: on average in 29.3% for HSP reports, 61.5% for PMR reports and in 39.2% for CuV reports. KD was reported with pneumococcal vaccines in 32.0% of KD reports and with rotavirus vaccines in more

than 20% of KD reports. BS was most frequently reported after hepatitis and HPV vaccines and CNS-V after HPV vaccines.

CONCLUSION: Similar reporting patterns of vasculitides were observed in different databases. Implementation of standardized case definitions for specific vasculitides could improve overall data quality and comparability of reports. Copyright Published by Elsevier Ltd.

Publication Type

Journal Article. Research Support, Non-U.S. Gov't.

Year of Publication

2016

<286>

Unique Identifier

26190453

Title

Influenza vaccination status in rheumatoid arthritis and spondyloarthritis patients receiving biologic DMARDs.

Source

Joint, Bone, Spine: Revue du Rhumatisme. 83(2):237-8, 2016 Mar.

VI 1

Status

MEDLINE

Authors

Michel M; Vincent FB; Rio S; Leon N; Marcelli C.

Authors Full Name

Michel, Murielle; Vincent, Fabien B; Rio, Simon; Leon, Nathalie; Marcelli, Christian.

Institution

Michel, Murielle. Department of Rheumatology, University Hospital Centre of Caen, avenue de la Cote-de-Nacre, 14033 Caen cedex 9, France. Electronic address: michel-m@chu-caen.fr.

Vincent, Fabien B. Department of Immunology, Monash University, Central Clinical School, Alfred Medical Research and Education Precinct (AMREP), 89 Commercial Road, Melbourne, Victoria 3004, Australia.

Rio, Simon. Department of Rheumatology, University Hospital Centre of Caen, avenue de la Cote-de-Nacre, 14033 Caen cedex 9, France.
Leon, Nathalie. Department of Rheumatology, University Hospital Centre of Caen, avenue de la Cote-de-Nacre, 14033 Caen cedex 9, France.
Marcelli, Christian. Department of Rheumatology, University Hospital Centre of Caen, avenue de la Cote-de-Nacre, 14033 Caen cedex 9, France.
Publication Type
Letter.
Year of Publication
2016

<287>
Unique Identifier
25906316
Title
Interaction between innate immunity and Ro52-induced antibody causes Sjogren's syndrome-like disorder in mice.
Source
Annals of the Rheumatic Diseases. 75(3):617-22, 2016 Mar.
VI 1
Status
MEDLINE
Authors
Szczerba BM; Kaplonek P; Wolska N; Podsiadlowska A; Rybakowska PD; Dey P; Rasmussen A; Grundahl K; Hefner KS; Stone DU; Young S; Lewis DM; Radfar L; Scofield RH; Sivils KL; Bagavant H; Deshmukh US.
Authors Full Name
Szczerba, Barbara M; Kaplonek, Paulina; Wolska, Nina; Podsiadlowska, Anna; Rybakowska, Paulina D; Dey, Paromita; Rasmussen, Astrid; Grundahl, Kiely; Hefner, Kimberly S; Stone, Donald U; Young, Stephen; Lewis, David M; Radfar, Lida; Scofield, R Hal; Sivils, Kathy L; Bagavant, Harini; Deshmukh, Umesh S.
Institution

Szczerba, Barbara M. Division of Nephrology, University of Virginia, Charlottesville, Virginia, USA University of Basel, Basel, Switzerland.

Kaplonek, Paulina. Arthritis and Clinical Immunology Program, Oklahoma Medical Research Foundation, Oklahoma City, Oklahoma, USA.

Wolska, Nina. Arthritis and Clinical Immunology Program, Oklahoma Medical Research Foundation, Oklahoma City, Oklahoma, USA.

Podsiadlowska, Anna. Arthritis and Clinical Immunology Program, Oklahoma Medical Research Foundation, Oklahoma City, Oklahoma, USA.

Rybakowska, Paulina D. Division of Nephrology, University of Virginia, Charlottesville, Virginia, USA Arthritis and Clinical Immunology Program, Oklahoma Medical Research Foundation, Oklahoma City, Oklahoma, USA.

Dey, Paromita. Division of Nephrology, University of Virginia, Charlottesville, Virginia, USA.

Rasmussen, Astrid. Arthritis and Clinical Immunology Program, Oklahoma Medical Research Foundation, Oklahoma City, Oklahoma, USA.

Grundahl, Kiely. Arthritis and Clinical Immunology Program, Oklahoma Medical Research Foundation, Oklahoma City, Oklahoma, USA.

Hefner, Kimberly S. Hefner Eye Care and Optical Center, Oklahoma City, Oklahoma, USA.

Stone, Donald U. Department of Ophthalmology, Dean McGee Eye Institute, University of Oklahoma Health Sciences Center, Oklahoma City, Oklahoma, USA.

Young, Stephen. University of Oklahoma College of Dentistry, Oklahoma City, Oklahoma, USA.

Lewis, David M. Department of Oral Diagnosis and Radiology, University of Oklahoma College of Dentistry, Oklahoma City, Oklahoma, USA.

Radfar, Lida. Department of Oral Diagnosis and Radiology, University of Oklahoma College of Dentistry, Oklahoma City, Oklahoma, USA.

Scofield, R Hal. Arthritis and Clinical Immunology Program, Oklahoma Medical Research Foundation, Oklahoma City, Oklahoma, USA Department of Medicine, University of Oklahoma Health Sciences Center, Oklahoma City, Oklahoma, USA Department of Veterans, Affairs Medical Center, Oklahoma City, Oklahoma, USA.

Sivils, Kathy L. Arthritis and Clinical Immunology Program, Oklahoma Medical Research Foundation, Oklahoma City, Oklahoma, USA.

Bagavant, Harini. Division of Nephrology, University of Virginia, Charlottesville, Virginia, USA Arthritis and Clinical Immunology Program, Oklahoma Medical Research Foundation, Oklahoma City, Oklahoma, USA.

Deshmukh, Umesh S. Division of Nephrology, University of Virginia, Charlottesville, Virginia, USA Arthritis and Clinical Immunology Program, Oklahoma Medical Research Foundation, Oklahoma City, Oklahoma, USA.

Abstract

OBJECTIVES: Autoantibodies reactive with Ro52 are often found in sera of patients with Sjogren's syndrome (SS). This study was undertaken to investigate the role of Ro52-induced immune responses in pathogenesis of SS.

METHODS: New Zealand Mixed (NZM) 2758 mice were immunised with Ro52 in alum adjuvant. Control mice were immunised either with maltose-binding protein or injected with alum alone. Mice were monitored for anti-Ro52 antibody, sialoadenitis and pilocarpine-induced salivation. Antibody binding to salivary gland (SG) cells was analysed in vivo and in vitro by immunofluorescence. Sera from immunised mice were passively transferred into untreated or alum injected NZM2758 mice.

RESULTS: By day 30 post-immunisation, Ro52 immunised mice generated immunoprecipitating anti-Ro52 antibodies and they had the maximum drop in saliva production. Both Ro52 immunised and control mice showed evidence of mild sialoadenitis. However, only Ro52 immunised mice had antibody deposition in their SG. Passive transfer of Ro52-immune sera induced SG dysfunction in recipient mice, only if the recipients were primed with alum. In vitro, antibodies from Ro52-immune sera were internalised by a SG cell line and this uptake was inhibited by cytochalasin D treatment.

CONCLUSIONS: Our data show for the first time that antibodies induced by Ro52 are capable of inducing SG dysfunction, and that this phenomenon is dependent on the activation of innate immunity. The mouse model described in this study implies that autoantibody deposition in the SG might be an important step in the induction of xerostomia and pathogenesis of SS. Copyright Published by the BMJ Publishing Group Limited. For permission to use (where not already granted under a licence) please go to http://www.bmj.com/company/products-services/rights-and-licensing/

Publication Type

Journal Article. Research Support, N.I.H., Extramural. Research Support, Non-U.S. Gov't.

Year of Publication

2016

<288>

Unique Identifier

25376466

Title

Screening of latent tuberculosis infection by interferon-gamma release assays in rheumatic patients: a systemic review and meta-analysis. [Review]

Source

Clinical Rheumatology. 35(2):417-25, 2016 Feb.

VI 1

Status

MEDLINE

Authors

Ruan Q; Zhang S; Ai J; Shao L; Zhang W.

Authors Full Name

Ruan, Qiaoling; Zhang, Shu; Ai, Jingwen; Shao, Lingyun; Zhang, Wenhong.

Institution

Ruan, Qiaoling. Department of Infectious Diseases, Huashan Hospital, Fudan University, 12 Wulumuqi Zhong Road, 200040, Shanghai, China.

Zhang, Shu. Department of Infectious Diseases, Huashan Hospital, Fudan University, 12 Wulumuqi Zhong Road, 200040, Shanghai, China.

Ai, Jingwen. Department of Infectious Diseases, Huashan Hospital, Fudan University, 12 Wulumuqi Zhong Road, 200040, Shanghai, China.

Shao, Lingyun. Department of Infectious Diseases, Huashan Hospital, Fudan University, 12 Wulumuqi Zhong Road, 200040, Shanghai, China.

Zhang, Wenhong. Department of Infectious Diseases, Huashan Hospital, Fudan University, 12 Wulumuqi Zhong Road, 200040, Shanghai, China. zhangwenhong@fudan.edu.cn.

Abstract

The aim of this study is to assess the diagnostic value of interferon-gamma release assays (IGRAs) for latent tuberculosis infection (LTBI) in patients with rheumatic disease before receiving biologic agents. MEDLINE and EMBASE databases were used for searching studies concerning the evaluation on the performance of IGRAs [QuantiFERON-TB Gold (QFT-G), QuantiFERON-TB Gold In-Tube (QFT-GIT) and T-SPOT.TB] in rheumatic patients before biological therapy. After assessing the quality of all studies included in the review, we summarized the results in subgroups using forest plots and calculated pooled estimates if applicable. The search identified 11 studies with a total sample size of 1940 individuals. Compared with the tuberculin skin test (TST), the pooled agreements in QFT-G/GIT and T-SPOT.TB were 72 % (95 % confidence interval (CI) 65, 78 %) and 75 % (95 % CI 67, 83 %), respectively. BCG vaccination was positively correlated with positive rates of TST (pooled odds ratio (OR) 1.64, 95 % CI 1.06, 2.53). Compared with TST, IGRAs were better associated with the presentence of one or more

tuberculosis (TB) risk factors. Neither steroid nor disease-modifying anti-rheumatic drugs (DMARDs) significantly affect positive IGRA results. In contrast, TST positivity was significantly impacted by the use of steroid (pooled OR 0.45, 95 % CI 0.30, 0.69), but less significantly by the use of DMARDs (pooled OR 0.78, 95 % CI 0.50, 1.21). In conclusion, in rheumatic patients with previous BCG vaccination or currently on steroid therapy, IGRAs would be the better choice to identify LTBI by decreasing the false-positivity and false-negativity rate compared with conventional TST.

Publication Type

Journal Article. Meta-Analysis. Research Support, Non-U.S. Gov't. Review.

Year of Publication

2016

<289>

Unique Identifier

31168414

Title

Polymyalgia rheumatica-like syndrome from checkpoint inhibitor therapy: case series and systematic review of the literature. RMD Open. 5(1):e000906, 2019.

Authors

Calabrese C; Cappelli LC; Kostine M; Kirchner E; Braaten T; Calabrese L.

Author NameID

Calabrese, Cassandra; ORCID: https://orcid.org/0000-0002-8773-7739

Authors Full Name

Calabrese, Cassandra; Cappelli, Laura C; Kostine, Marie; Kirchner, Elizabeth; Braaten, Tawnie; Calabrese, Leonard.

Institution

Calabrese, Cassandra. Rheumatic & Immunologic Disease, Cleveland Clinic, Cleveland, Ohio, USA.

Cappelli, Laura C. Division of Rheumatology, Johns Hopkins School of Medicine, Baltimore, Maryland, USA.

Kostine, Marie. Rheumatology Department, Centre Hospitalier Universitaire de Bordeaux, Bordeaux, France.

Kirchner, Elizabeth. Rheumatic & Immunologic Disease, Cleveland Clinic, Cleveland, Ohio, USA.
Braaten, Tawnie. Division of Rheumatology, Johns Hopkins School of Medicine, Baltimore, Maryland, USA.
Calabrese, Leonard. Rheumatic & Immunologic Disease, Cleveland Clinic, Cleveland, Ohio, USA.
Abstract
Objective: To assess whether the polymyalgia rheumatica (PMR)-like syndrome reported as an immune related adverse event (irAE) from checkpoint inhibitor therapy is consistent with the 2012 European League Against Rheumatism (EULAR)/American College of Rheumatology (ACR) provisional criteria for PMR.

Methods: The cases were derived from two sources. Group 1 represents reported cases from three contributing centres. Group 2 was derived from a systematic review of the literature searching for all cases reported as PMR or PMR-like illness associated with checkpoint inhibitor therapy. Cases were assessed for the quality of reporting and then analysed to determine whether they fulfilled the 2012 EULAR/ACR provisional criteria for PMR.

Results: A total of 49 patients were included for analysis. Among the entire group, 37 (75%) were designated 'complete' indicating that they had sufficient data to reliably apply the 2012 EULAR/ACR criteria. 28 (75%) cases fulfilled complete criteria for PMR. A number of cases also demonstrated some clinical features unusual for idiopathic PMR.

Conclusion: This study suggests a high proportion of reported cases of checkpoint inhibitor-related PMR fulfil preliminary criteria for PMR, yet in one quarter clinical details were incomplete making verification problematic. Furthermore, in the absence of a gold standard for the diagnosis of PMR, the relationship of checkpoint inhibitor-related PMR to the idiopathic form remains unclear.
Publication Type
Research Support, N.I.H., Extramural. Journal Article.
Year of Publication
2019

<290>

Unique Identifier

30453170

Title

Myositis and neuromuscular side-effects induced by immune checkpoint inhibitors.

European Journal of Cancer. 106:12-23, 2019 Jan.

IN-DATA-REVIEW

Authors

Moreira A; Loquai C; Pfohler C; Kahler KC; Knauss S; Heppt MV; Gutzmer R; Dimitriou F; Meier F; Mitzel-Rink H; Schuler G; Terheyden P; Thoms KM; Turk M; Dummer R; Zimmer L; Schroder R; Heinzerling L.

Authors Full Name

Moreira, Alvaro; Loquai, Carmen; Pfohler, Claudia; Kahler, Katharina C; Knauss, Samuel; Heppt, Markus V; Gutzmer, Ralf; Dimitriou, Florentia; Meier, Friedegund; Mitzel-Rink, Heidrun; Schuler, Gerold; Terheyden, Patrick; Thoms, Kai-Martin; Turk, Matthias; Dummer, Reinhard; Zimmer, Lisa; Schroder, Rolf; Heinzerling, Lucie.

Institution

Moreira, Alvaro. Department of Dermatology, University Hospital Erlangen, Friedrich-Alexander-University Erlangen-Nurnberg (FAU), Germany.

Loquai, Carmen. Department of Dermatology, University Hospital of Mainz, Mainz, Germany.

Pfohler, Claudia. Department of Dermatology, Saarland University Medical School, Homburg, Germany.

Kahler, Katharina C. Department of Dermatology, Skin Cancer Center, University Hospital Schleswig-Holstein (UKSH), Campus Kiel, Kiel, Germany.

Knauss, Samuel. Department of Experimental Neurology, Charite Universitatsmedizin Berlin, Germany.

Heppt, Markus V. Department of Dermatology and Allergy, University Hospital, LMU Munich, Frauenlobstr. 9-11, 80337 Munich, Germany.

Gutzmer, Ralf. Skin Cancer Center Hannover, Department of Dermatology and Allergy, Hannover Medical School, Germany.

Dimitriou, Florentia. Department of Dermatology, University Hospital Zurich, Switzerland.

Meier, Friedegund. Department of Dermatology, University Hospital Dresden, Germany.

Mitzel-Rink, Heidrun. Department of Dermatology, University Hospital of Mainz, Mainz, Germany.

Schuler, Gerold. Department of Dermatology, University Hospital Erlangen, Friedrich-Alexander-University Erlangen-Nurnberg (FAU), Germany.

Terheyden, Patrick. Department of Dermatology, University of Lubeck, Lubeck, Germany.

Thoms, Kai-Martin. Department of Dermatology, University Hospital Gottingen, Germany.
Turk, Matthias. Department of Neuropathology, University Hospital Erlangen, Friedrich-Alexander-University Erlangen-Nurnberg (FAU), Germany.
Dummer, Reinhard. Department of Dermatology, University Hospital Zurich, Switzerland.
Zimmer, Lisa. Department of Dermatology, University Duisburg-Essen, Germany.
Schroder, Rolf. Department of Neuropathology, University Hospital Erlangen, Friedrich-Alexander-University Erlangen-Nurnberg (FAU), Germany.
Heinzerling, Lucie. Department of Dermatology, University Hospital Erlangen, Friedrich-Alexander-University Erlangen-Nurnberg (FAU), Germany. Electronic address: Lucie.Heinzerling@uk-erlangen.de.
Abstract
AIM: To characterise clinical presentation, laboratory and histopathologic characteristics and assess the treatment and outcome of neuromuscular side-effects of checkpoint therapy.

METHODS: The side-effect registry and the institutional database from ten skin cancer centres were queried for reports on myositis and neuromuscular side-effects induced by checkpoint inhibitors. In total, 38 patients treated with ipilimumab, tremelimumab, nivolumab and pembrolizumab for metastatic skin cancer were evaluated and characterised.

RESULTS: Myositis was the most frequent neuromuscular adverse event. In 32% of cases, myositis was complicated by concomitant myocarditis. Furthermore, cases of isolated myocarditis, myasthenia gravis, polymyalgia rheumatica, radiculoneuropathy and asymptomatic creatine kinase elevation were reported. The onset of side-effects ranged from the first week of treatment to 115 weeks after the start of therapy. Most of the cases were severe (49% grade III-IV Common Terminology Criteria for Adverse Events), and there were two fatalities (5%) due to myositis and myositis with concomitant myocarditis. Only half of the cases (50%) completely resolved, whereas the rest was either ongoing or had sequelae. Steroids were given in 80% of the resolved cases and in 40% of the unresolved cases.

CONCLUSION: Immune-mediated neuromuscular side-effects of checkpoint inhibitors greatly vary in presentation and differ from their idiopathic counterparts. These side-effects can be life threatening and may result in permanent sequelae. Occurrence of these side-effects must be taken into consideration for patient information, especially when considering adjuvant immunotherapy with anti-programmed cell-death protein 1 (PD-1) antibodies and monitoring, which should include regular surveillance of creatine kinase. Copyright © 2018. Published by Elsevier Ltd.
Publication Type

Journal Article.
Year of Publication
2019

<291>
Unique Identifier
29950326
Title
Checkpoint inhibitors and arthritis: seeking balance between victories and defeats.
Source
Annals of the Rheumatic Diseases. 78(9):e91, 2019 Sep.
VI 1
Status
IN-DATA-REVIEW
Authors
Moura CA; Moura CG.
Authors Full Name
Moura, Carlos Antonio; Moura, Carlos Geraldo.
Institution
Moura, Carlos Antonio. Clinica Medica, Hospital Santo Antonio, Salvador, Bahia, Brazil.
Moura, Carlos Antonio. Clinica Medica, Escola Bahiana de Medicina e Saude Publica, Salvador, Bahia, Brazil.
Moura, Carlos Antonio. Rheumatology, Universidade Salvador (UNIFACS), Salvador, Bahia, Brazil.
Moura, Carlos Geraldo. Clinica Medica, Hospital Santo Antonio, Salvador, Bahia, Brazil.
Moura, Carlos Geraldo. Clinica Medica, Escola Bahiana de Medicina e Saude Publica, Salvador, Bahia, Brazil.
Publication Type
Letter.
Year of Publication
2019

<292>

Unique Identifier

30167328

Title

Characteristics and treatment of new-onset arthritis after checkpoint inhibitor therapy.

Source

RMD Open. 4(2):e000714, 2018.

VI 1

Status

PubMed-not-MEDLINE

Authors

Leipe J; Christ LA; Arnoldi AP; Mille E; Berger F; Heppt M; Goldscheider I; Kauffmann-Guerrero D; Huber RM; Dechant C; Berking C; Schulze-Koops H; Skapenko A.

Authors Full Name

Leipe, Jan; Christ, Lisa A; Arnoldi, Andreas P; Mille, Erik; Berger, Frank; Heppt, Markus; Goldscheider, Ilana; Kauffmann-Guerrero, Diego; Huber, Rudolf M; Dechant, Claudia; Berking, Carola; Schulze-Koops, Hendrik; Skapenko, Alla.

Institution

Leipe, Jan. Division of Rheumatology and Clinical Immunology, Department of Internal Medicine IV, Ludwig-Maximilians-University Munich, Munich, Germany.

Christ, Lisa A. Division of Rheumatology and Clinical Immunology, Department of Internal Medicine IV, Ludwig-Maximilians-University Munich, Munich, Germany.

Arnoldi, Andreas P. Department of Radiology, Ludwig-Maximilians-University Munich, Munich, Germany.

Mille, Erik. Department of Nuclear Medicine, Ludwig-Maximilians-University Munich, Munich, Germany.

Berger, Frank. Department of Radiology, Ludwig-Maximilians-University Munich, Munich, Germany.

Heppt, Markus. Department of Dermatology and Allergology, Ludwig-Maximilians-University Munich, Munich, Germany.

Goldscheider, Ilana. Department of Dermatology and Allergology, Ludwig-Maximilians-University Munich, Munich, Germany.

Kauffmann-Guerrero, Diego. Division of Respiratory Medicine and Thoracic Oncology, Department of Internal Medicine V, Ludwig-Maximilians-University Munich, Munich, Germany.

Huber, Rudolf M. Division of Respiratory Medicine and Thoracic Oncology, Department of Internal Medicine V, Ludwig-Maximilians-University Munich, Munich, Germany.
Dechant, Claudia. Division of Rheumatology and Clinical Immunology, Department of Internal Medicine IV, Ludwig-Maximilians-University Munich, Munich, Germany.
Berking, Carola. Department of Dermatology and Allergology, Ludwig-Maximilians-University Munich, Munich, Germany.
Schulze-Koops, Hendrik. Division of Rheumatology and Clinical Immunology, Department of Internal Medicine IV, Ludwig-Maximilians-University Munich, Munich, Germany.
Skapenko, Alla. Division of Rheumatology and Clinical Immunology, Department of Internal Medicine IV, Ludwig-Maximilians-University Munich, Munich, Germany.
Abstract
Immune checkpoint inhibitors (ICIs) may cause immune-related adverse events (IRAEs). Characterisation and data on treatment of musculoskeletal IRAEs are scarce. In this cohort study, patients receiving ICI therapy who experienced arthralgia were evaluated for the presence of synovitis. Data on demographics, ICI regime, time of onset, imaging and response to therapy of synovitis were prospectively collected. Arthritis was demonstrated in 14 of 16 patients of whom 7 showed monarthritis, 5 had oligoarthritis and 2 had polyarthritis. Patients with ICI-induced arthritis were predominantly male (57%) and seronegative (69%). Regarding the detection of synovitis in staging imaging, moderate sensitivity for contrast-enhanced CT with PET-CT as reference was observed. Disease burden at baseline was high and was significantly reduced after anti-inflammatory treatment. Nine patients were treated with systemic and eight patients with intra-articular glucocorticoids. Six patients who flared on glucocorticoid treatment on tapering were given methotrexate resulting in long-term remission. Patients with synovitis were more likely to have good tumour response. Patients with ICI-induced arthritis were predominantly male and seronegative showing different patterns of arthritis with high disease burden. Good efficacy and safety was observed for methotrexate, particularly for ICI-induced polyarthritis.
Publication Type
Journal Article.
Year of Publication
2018

<293>
Unique Identifier

28405474

Title

Rheumatic immune-related adverse events of checkpoint therapy for cancer: case series of a new nosological entity. RMD Open. 3(1):e000412, 2017.

Authors

Calabrese C; Kirchner E; Kontzias A; Velcheti V; Calabrese LH.

Authors Full Name

Calabrese, C; Kirchner, E; Kontzias, A; Velcheti, V; Calabrese, L H.

Institution

Calabrese, C. Cleveland Clinic Foundation, Cleveland, Ohio, USA; Departmentof Rheumatology and Immunology, Cleveland Clinic, Cleveland, Ohio, USA.

Kirchner, E. Cleveland Clinic Foundation, Cleveland, Ohio, USA; Departmentof Rheumatology and Immunology, Cleveland Clinic, Cleveland, Ohio, USA.

Kontzias, A. Cleveland Clinic Foundation, Cleveland, Ohio, USA; Departmentof Rheumatology and Immunology, Cleveland Clinic, Cleveland, Ohio, USA.

Velcheti, V. Cleveland Clinic Foundation, Cleveland, Ohio, USA; Department of Hematology and Oncology, Cleveland Clinic, Cleveland, Ohio, USA.

Calabrese, L H. Cleveland Clinic Foundation, Cleveland, Ohio, USA; Departmentof Rheumatology and Immunology, Cleveland Clinic, Cleveland, Ohio, USA.

Comments

Erratum in (EIN)

Abstract

Immunotherapy of cancer with checkpoint inhibitors has been associated with a spectrum of autoimmune and systemic inflammatory reactions known as immune-related adverse events (irAEs). Rheumatic irAEs are infrequently reported and extensively described. Here, we report our experience over an 18-month period with 15 patients evaluated in the rheumatology department for rheumatic irAEs. We identified 13 patients without pre-existing autoimmune disease (AID) who subsequently developed rheumatic irAEs, and two with established AID referred pre-emptively. irAEs encountered included: inflammatory arthritis, sicca syndrome, polymyalgia rheumatica-like symptoms and myositis. All cases required glucocorticoids, and three required a biological agent. Rheumatic irAEs led to temporary or permanent cessation of immunotherapy in all but five patients. One patient with pre-existing AID experienced a flare after starting immunotherapy. Our findings underscore that rheumatic irAEs are complex, at times require additional immunosuppressive therapy, and may influence ongoing immunotherapy regimens for the primary disease. Similar irAEs will be increasingly seen as checkpoint inhibitors adopted as standard of care in the community.

Publication Type
  Journal Article.
Year of Publication
  2017

<294>
Unique Identifier
  27259225
Title
  [Autoimmune connective tissue diseases and vaccination]. [Review] [Polish]
Source
  Postepy Higieny i Medycyny do Swiadczalnej (Online). 69:1530-8, 2015 Dec 31.
VI 1
Status
  MEDLINE
Authors
  Wiesik-Szewczyk E; Jahnz-Rozyk K.
Authors Full Name
  Wiesik-Szewczyk, Ewa; Jahnz-Rozyk, Karina.
Institution
  Wiesik-Szewczyk, Ewa. Zaklad Immunologii i Alergologii Klinicznej, Wojskowy Instytut Medyczny w Warszawie.
  Jahnz-Rozyk, Karina. Zaklad Immunologii i Alergologii Klinicznej, Wojskowy Instytut Medyczny w Warszawie.
Abstract
  The idea that infectious agents can induce autoimmune diseases in genetically susceptible subjects has been a matter of discussion for years. Moreover, increased incidence of autoimmune diseases and introduction of prophylactic vaccinations from early childhood suggest that these two trends are linked. In the medical literature and even non-professional media, case reports or events temporally related to vaccination are reported. It raises the issue of vaccination safety. In everyday practice medical professionals, physicians, rheumatologists and other specialists will be asked their opinion of vaccination safety. The decision should be made according to evidence-based medicine and the current state of knowledge. The purpose of this

paper is to discuss a potential mechanism which links infections, vaccinations and autoimmunity. We present an overview of published case reports, especially of systemic connective tissue diseases temporally related to vaccination and results from case-nested studies. As yet, no conclusive evidence supports a causal relationship between vaccination and autoimmune diseases. It has to be determined whether the performed studies are sufficiently sensitive to detect the link. The debate is ongoing, and new data may be required to explain the pathogenesis of autoimmunity. We would like to underscore the need for prophylactic vaccination in patients with autoimmune rheumatic diseases and to break down the myth that the vaccines are contraindicated in this target group.

Publication Type

Journal Article. Review.

Year of Publication

2015

<295>

Unique Identifier

26621128

Title

BAFF inhibition does not significantly impair immunization responses in patients with rheumatoid arthritis.

Source

Arthritis Research & Therapy. 17:347, 2015 Nov 30.

VI 1

Status

MEDLINE

Authors

Bingham CO 3rd; Winthrop KL; Yang L; Lee C; Komocsar WJ.

Authors Full Name

Bingham, Clifton O 3rd; Winthrop, Kevin L; Yang, Lili; Lee, Chin; Komocsar, Wendy J.

Institution

Bingham, Clifton O 3rd. Division of Rheumatology, Johns Hopkins University School of Medicine, 5200 Eastern Avenue, Mason F. Lord Center Tower Room 404, Baltimore, MD, 21224, USA. cbingha2@jhmi.edu.

Winthrop, Kevin L. Divisions of Infectious Diseases, Public Health, and Preventive Medicine, Oregon Health & Science University, 3375 SW Terwilliger Blvd, Portland, OR, 97239, USA.
Yang, Lili. Eli Lilly and Company, Indianapolis, IN, 46285, USA.
Lee, Chin. Eli Lilly and Company, Indianapolis, IN, 46285, USA.
Komocsar, Wendy J. Eli Lilly and Company, Indianapolis, IN, 46285, USA.
Publication Type
Letter. Randomized Controlled Trial.
Year of Publication
2015

<296>
Unique Identifier
26492963
Title
Influenza vaccination is safe and effective in patients suffering from fibromyalgia syndrome.
Source
Reumatismo. 67(2):57-61, 2015 Sep 16.
VI 1
Status
MEDLINE
Authors
Ablin JN; Aloush V; Brill A; Berman M; Barzilai M; Caspi D; Mandelboim M; Levartovsky D; Polachek A; Wolman Y; Paran D; Barkagan M; Elkayam O.
Authors Full Name
Ablin, J N; Aloush, V; Brill, A; Berman, M; Barzilai, M; Caspi, D; Mandelboim, M; Levartovsky, D; Polachek, A; Wolman, Y; Paran, D; Barkagan, M; Elkayam, O.
Institution
Ablin, J N. Institute of Rheumatology, Tel Aviv Sourasky Medical Center, & Sackler School of Medicine, Tel Aviv University, Tel Aviv. ajacob@post.tau.ac.il.
Abstract
The fibromyalgia syndrome (FMS) is considered to result from the exposure of a genetically susceptible individual to various triggers, such as physical trauma, stress, viral infections etc. A possible role of vaccination in FMS etiology has been suspected. Our objective was to evaluate

the efficacy and safety of influenza vaccination in FMS patients. Nineteen FMS patients underwent physical and dolorimetric examinations and answered the fibromyalgia impact questionnaire (FIQ), the widespread pain index (WPI) checklist and the symptoms severity scale (SSS), which are part of the 2010 diagnostic criteria. Thirty-eight healthy subjects were recruited as controls. All participants were vaccinated with the inactivated split virion influenza vaccine. Serum was collected for antibody titration. Six weeks after vaccination, sera were tested by hemagglutination (HI) against A/California (H1N1), A/Perth (H3N2) and B/Brisbane. Humoral response was defined as either a fourfold or greater increase in titer, or an increase from a non-protective baseline level of <1/40 to a level of 1/40. No severe vaccination reactions were observed. No significant change was observed between WPI, SSS and FIQ values before and after vaccination, indicating no worsening of FMS symptoms. Vaccine immunogenicity: Six weeks after vaccination, FMS patients showed a significant increase in geometric mean titers of HI antibody. The rates of sero-protection increased from 22.9% for H1N1 to 89.5% post-vaccination. A significant increase in HI antibody titers was also demonstrated among healthy controls. Influenza vaccination was both safe and effective in FMS patients. In view of these results, FMS patients should be encouraged to undergo influenza vaccination according to the standard WHO recommendations.

Publication Type

Clinical Trial. Journal Article.

Year of Publication

2015

<297>

Unique Identifier

26394271

Title

Varicella-zoster virus infection in rheumatoid arthritis patients in the anti-tumour necrosis factor era. [Review]

Source

Clinical & Experimental Rheumatology. 33(6):917-23, 2015 Nov-Dec.

VI 1

Status

MEDLINE

Authors

Cacciapaglia F; Zuccaro C; Iannone F.

Authors Full Name

Cacciapaglia, F; Zuccaro, C; Iannone, F.

Institution

Cacciapaglia, F. Internal Medicine Unit, Rheumatology Outpatient Clinic Ninetto Melli Hospital, San Pietro Vernotico, Italy. f.cacciapaglia@unicampus.it.

Zuccaro, C. Geriatric Unit, Rheumatology Outpatient Clinic, Antonio Perrino Hospital, Brindisi, Italy.

Iannone, F. Department Interdisciplinary of Medicine, Rheumatology Unit, University of Bari, Italy.

Abstract

Patients with rheumatoid arthritis are increasingly being treated with different drugs (both non-biologic and biologic disease-modifying anti-rheumatic drugs - DMARDs) that may have immunomodulatory, cytotoxic, or immunosuppressive effects; in particular, anti-tumour necrosis factor (TNF) agents are raising major concern as regards safety issues. An increased risk of infections has been extensively reported during anti-TNF treatment, owing to the primary role of TNF in host defense and immune responses. Although in clinical practice cases of reactivation of varicella zoster virus (VZV) infections during therapy with TNF inhibitors commonly occur, the knowledge on this topic deriving from randomised clinical trials is limited. In this narrative review we focus on the pathophysiology of VZV infection and the role of TNF, and report the available data about VZV outbreaks recorded on Registries of rheumatic patients treated with anti-TNF agents. Finally, we discuss screening strategies and promising preventive measures against VZV infection.

Publication Type

Journal Article. Review.

Year of Publication

2015

<298>

Unique Identifier

26362746

Title

Role of vaccinations and prophylaxis in rheumatic diseases. [Review]

Source

Best Practice & Research in Clinical Rheumatology. 29(2):306-18, 2015 Apr.

VI 1

Status

MEDLINE

Authors

Papadopoulou D; Tsoulas C; Tragiannidis A; Sipsas NV.

Authors Full Name

Papadopoulou, Despoina; Tsoulas, Christos; Tragiannidis, Athanassios; Sipsas, Nikolaos V.

Institution

Papadopoulou, Despoina. Pain and Palliative Care Unit, Aretaieion Hospital, National and Kapodistrian University of Athens, Athens, Greece. Electronic address: papadopould@med.uoa.gr.

Tsoulas, Christos. Institute for Continuing Medical Education of Ioannina, Ioannina, Greece. Electronic address: christsoulas@gmail.com.

Tragiannidis, Athanassios. Hematology and Oncology Unit, Second Department of Pediatrics, AHEPA Hospital, Aristotle University of Thessaloniki, Thessaloniki, Greece. Electronic address: atragian@auth.gr.

Sipsas, Nikolaos V. Infectious Disease Unit, Pathophysiology Department, Laikon General Hospital and Medical School, National and Kapodistrian University of Athens, Athens, Greece. Electronic address: nsipsas@med.uoa.gr.

Abstract

Targeted strategies for reducing the increased risk of infection in patients with autoimmune rheumatic diseases include vaccinations as well as antibiotic prophylaxis in selected patients. However, there are still issues under debate: Is vaccination in patients with rheumatic diseases immunogenic? Is it safe? What is the impact of immunosuppressive drugs on vaccine immunogenicity and safety? Does vaccination cause disease flares? In which cases is prophylaxis against Pneumocystis jirovecii required? This review addresses these important questions to which clinicians and researchers still do not have definite answers. The first part includes immunization recommendations and reviews current data on vaccine efficacy and safety in patients with rheumatic diseases. The second part discusses prophylaxis for Pneumocystis pneumonia. Copyright © 2015 Elsevier Ltd. All rights reserved.

Publication Type

Journal Article. Research Support, Non-U.S. Gov't. Review.

Year of Publication

2015

<299>

Unique Identifier

26337719

Title

Immune related adverse events associated with anti-CTLA-4 antibodies: systematic review and meta-analysis. [REVIEW]

BMC Medicine. 13:211, **2015** Sep 04.

Authors

Bertrand A; Kostine M; Barnetche T; Truchetet ME; Schaeverbeke T.

Authors Full Name

Bertrand, Anne; Kostine, Marie; Barnetche, Thomas; Truchetet, Marie-Elise; Schaeverbeke, Thierry.

Institution

Bertrand, Anne. Departement de Rhumatologie, Hopital Pellegrin, CHU de Bordeaux, Bordeaux, France. anne.bertrand87@gmail.com.

Kostine, Marie. Departement de Rhumatologie, Hopital Pellegrin, CHU de Bordeaux, Bordeaux, France. mariekostine@hotmail.fr.

Barnetche, Thomas. Departement de Rhumatologie, Hopital Pellegrin, CHU de Bordeaux, Bordeaux, France. thomas.barnetche@chu-bordeaux.fr.

Truchetet, Marie-Elise. Departement de Rhumatologie, Hopital Pellegrin, CHU de Bordeaux, Bordeaux, France. marie-elise.truchetet@chu-bordeaux.fr.

Truchetet, Marie-Elise. Laboratoire d'Immunologie, UMR-CNRS 5164, Universite de Bordeaux, Bordeaux, France. marie-elise.truchetet@chu-bordeaux.fr.

Schaeverbeke, Thierry. Departement de Rhumatologie, Hopital Pellegrin, CHU de Bordeaux, Bordeaux, France. thierry.schaeverbeke@chu-bordeaux.fr.

Schaeverbeke, Thierry. Unite sous Contrat, Infections a Mycoplasmes et a Chlamydia chez l'Homme, Universite de Bordeaux, Bordeaux, France. thierry.schaeverbeke@chu-bordeaux.fr.

Abstract

BACKGROUND: Targeting CTLA-4 is a recent strategic approach in cancer control: blocking CTLA-4 enhances an antitumor immunity by promoting T-cell activation and cytotoxic T-lymphocyte proliferation. This induction of a tolerance break against the tumor may be responsible for immune-related adverse events (irAEs). Our objective was to assess the

incidence and nature of irAEs in oncologic patients receiving anti-CTLA-4 antibodies (ipilimumab and tremelimumab).

METHODS: A systematic search of literature up to February 2014 was performed in MEDLINE, EMBASE, and Cochrane databases to identify relevant articles. Paired reviewers independently selected articles for inclusion and extracted data. Pooled incidence was calculated using R(©), package meta.

RESULTS: Overall, 81 articles were included in the study, with a total of 1265 patients from 22 clinical trials included in the meta-analysis. Described irAEs consisted of skin lesions (rash, pruritus, and vitiligo), colitis, and less frequently hepatitis, hypophysitis, thyroiditis, and some rare events such as sarcoidosis, uveitis, Guillain-Barre syndrome, immune-mediated cytopenia and polymyalgia rheumatic/Horton. The overall incidence of all-grade irAEs was 72 % (95 % CI, 65-79 %). The overall incidence of high-grade irAEs was 24 % (95 % CI, 18-30 %). The risk of developing irAEs was dependent of dosage, with incidence of all-grade irAEs being evaluated to 61 % (95 % CI, 56-66 %) for ipilimumab 3 mg/kg and 79 % (95 % CI, 69-89 %) for ipilimumab 10 mg/kg. Death due to irAEs occurred in 0.86 % of patients. The median time of onset of irAEs was about 10 weeks (IQR, 6-12) after the onset of treatment, corresponding with the first three cycles but varied according to the organ system involved. Such immune activation could also be indicative for tumor-specific T-cell activation and irAE occurrence was associated with clinical response to CTLA-4 blocking in 60 % of patients.

CONCLUSION: The price of potential long-term survival to metastatic tumors is an atypical immune toxicity, reflecting the mechanism of action of anti-CTLA-4 antibodies. A better knowledge of these irAEs and its management in a multidisciplinary approach will help to reduce morbidity and therapy interruptions.

Publication Type

Journal Article. Meta-Analysis. Review. Systematic Review.

Year of Publication

2015

<300>

Unique Identifier

26293116

Title

Peptidylarginine deiminase type 4 deficiency reduced arthritis severity in a glucose-6-phosphate isomerase-induced arthritis model.

Source

Scientific Reports. 5:13041, 2015 Aug 21.

VI 1

Status

MEDLINE

Authors

Seri Y; Shoda H; Suzuki A; Matsumoto I; Sumida T; Fujio K; Yamamoto K.

Authors Full Name

Seri, Yu; Shoda, Hirofumi; Suzuki, Akari; Matsumoto, Isao; Sumida, Takayuki; Fujio, Keishi; Yamamoto, Kazuhiko.

Institution

Seri, Yu. Department of Allergy and Rheumatology, Graduate School of Medicine, the University of Tokyo, Bunkyo-ku, Tokyo, 113-8655, Japan.

Shoda, Hirofumi. Department of Allergy and Rheumatology, Graduate School of Medicine, the University of Tokyo, Bunkyo-ku, Tokyo, 113-8655, Japan.

Suzuki, Akari. Laboratory for Rheumatic Diseases, SNP Research Center, The Institute of Physical and Chemical Research (RIKEN), 1-7-22 Suehirocho, Tsurumi-ku, Yokohama City, Kanagawa 230-0045, Japan.

Matsumoto, Isao. Division of Clinical Immunology, Major of Advanced Biomedical Applications, Graduate School of Comprehensive Human Sciences, Tsukuba University, Tsukuba, Ibaraki, 305-8575, Japan.

Sumida, Takayuki. Division of Clinical Immunology, Major of Advanced Biomedical Applications, Graduate School of Comprehensive Human Sciences, Tsukuba University, Tsukuba, Ibaraki, 305-8575, Japan.

Fujio, Keishi. Department of Allergy and Rheumatology, Graduate School of Medicine, the University of Tokyo, Bunkyo-ku, Tokyo, 113-8655, Japan.

Yamamoto, Kazuhiko. Department of Allergy and Rheumatology, Graduate School of Medicine, the University of Tokyo, Bunkyo-ku, Tokyo, 113-8655, Japan.

Yamamoto, Kazuhiko. Laboratory for Rheumatic Diseases, SNP Research Center, The Institute of Physical and Chemical Research (RIKEN), 1-7-22 Suehirocho, Tsurumi-ku, Yokohama City, Kanagawa 230-0045, Japan.

Abstract

Peptidyl arginine deiminase 4 (PAD4) is an enzyme that is involved in protein citrullination, and is a target for anti-citrullinated peptide antibodies (ACPAs) in rheumatoid arthritis (RA). Genetic polymorphisms in the PADI4 gene encoding PAD4 are associated with RA susceptibility. We herein analyzed the roles of PADI4 in inflammatory arthritis using a glucose-6-phosphate isomerase (GPI)-induced arthritis (GIA) model in Padi4 knockout (KO) mice. Arthritis severity, serum anti-GPI antibody titers, and IL-6 concentrations were significantly reduced in Padi4 KO mice. The frequency of Th17 cells was decreased in GPI-immunized Padi4 KO mice, whereas WT and Padi4-deficient naive CD4(+) T cells displayed the same efficiencies for Th17 cell differentiation in vitro. In addition, the numbers of myeloid lineage cells were reduced with the increased expression of pro-apoptotic genes in GPI-immunized Padi4 KO mice. Furthermore, the survival of Padi4-deficient neutrophils was impaired in vitro. Our results suggest that PADI4 exacerbates arthritis with diverse immunological modifications.

Publication Type

Journal Article. Research Support, Non-U.S. Gov't.

Year of Publication

2015

<301>

Unique Identifier

26290110

Title

B Cell Therapies, Approved and Emerging: a Review of Infectious Risk and Prevention During Use. [Review]

Source

Current Rheumatology Reports. 17(10):65, 2015 Oct.

VI 1

Status

MEDLINE

Authors

Md Yusof MY; Vital EM; Buch MH.

Authors Full Name

Md Yusof, Md Yuzaiful; Vital, Edward M; Buch, Maya H.

Institution

Md Yusof, Md Yuzaiful. Leeds Institute of Rheumatic and Musculoskeletal Medicine, University of Leeds, Chapel Allerton Hospital, Chapeltown Road, Leeds, LS7 4SA, UK.
Abstract
The development of B cell-targeted biologics represents a major advance in the treatment of autoimmune rheumatic diseases. As with other immunosuppressive agents, risk of infection is a key clinical concern. This review summarises safety data from 15 years of experience of rituximab in autoimmune diseases with a particular focus on opportunistic infection and class-specific complications and infection risk. Rarely, cases of progressive multifocal leucoencephalopathy in rituximab-treated patients (5/100 000) have accumulated over time although no proven causal association has yet been shown. With repeat cycles of therapy, hypogammaglobulinaemia has been observed in a larger proportion of patients and is associated with increased risk of serious infections. The infection profile of the newer B cell-targeted agent, belimumab, in patients with active systemic lupus erythematosus is also discussed. Data from registries are needed to extend insights further and also to evaluate for any impact with the difference in mode of action of belimumab and infection risk in this population.
Publication Type
Journal Article. Review.
Year of Publication
2015

<302>
Unique Identifier
26218860
Title
Vaccination recommendations for adult patients with autoimmune inflammatory rheumatic diseases.
Source
Swiss Medical Weekly. 145:w14159, 2015.
VI 1
Status
MEDLINE
Authors
Buhler S; Eperon G; Ribi C; Kyburz D; van Gompel F; Visser LG; Siegrist CA; Hatz C.

Authors Full Name
Buhler, Silja; Eperon, Gilles; Ribi, Camillo; Kyburz, Diego; van Gompel, Fons; Visser, Leo G; Siegrist, Claire-Anne; Hatz, Christoph.
Institution
Buhler, Silja. Department of Public Health, Division of Infectious Disease / Travel Clinic, Epidemiology, Biostatistics and Prevention Institute, University of Zurich, Zurich, Switzerland.
Eperon, Gilles. Service de Medecine Tropicale et Humanitaire, Geneva University Hospitals, Geneva, Switzerland.
Ribi, Camillo. Division of Immunology and Allergy, Centre Hospitalier Universitaire Vaudois, Lausanne, Switzerland.
Kyburz, Diego. Department of Rheumatology, Basel University Hospital, Basel, Switzerland.
van Gompel, Fons. Department of Tropical Medicine, Institute for Tropical Medicine, Antwerp, Belgium.
Visser, Leo G. Department of Infectious Diseases, Leiden University Medical Centre, Leiden, The Netherlands.
Siegrist, Claire-Anne. Centre for Vaccinology, University Hospital and Faculty of Medicine, Geneva, Switzerland.
Hatz, Christoph. Department of Public Health, Division of Infectious Disease / Travel Clinic, Epidemiology, Biostatistics and Prevention Institute, University of Zurich, Zurich, Switzerland, and Swiss Tropical and Public Health Institute, Department of Medicine and Diagno.
Abstract
BACKGROUND: The number of individuals with autoimmune inflammatory rheumatic diseases (AIIRDs) treated with immunosuppressive drugs is increasing steadily. The variety of immunosuppressive drugs and, in particular, biological therapies is also rising. The immunosuppressants, as well as the AIIRD itself, increase the risk of infection in this population. Thus, preventing infections by means of vaccination is of utmost importance. New Swiss vaccination recommendations for AIIRD patients were initiated by the Swiss Federal Office of Public Health and prepared by a working group of the Federal Commission for Vaccination Issues as well as by consultation of international experts.

METHODS: A literature search was performed in electronic databases (Cochrane, Medline, PubMed, Embase). In addition, unpublished literature was identified through a targeted website search of relevant organisations and international conferences dealing with vaccination, infectious diseases and rheumatology.

RESULTS: Although data are scarce, the following main points were retrieved from the literature. Inactivated vaccines are safe, but their immunogenicity may be reduced in AIIRD

patients, especially if they are under immunosuppressive therapy. Rituximab and abatacept appear to reduce significantly immune responses after vaccination. Live vaccines are generally contraindicated under immunosuppressive therapy owing to safety concerns. Specific exceptions, as well as time intervals for the administration of live vaccines after interruption of an immunosuppressive therapy, have been formulated in this article.

CONCLUSION: More evidence regarding the immunogenicity and safety of vaccinations in AIIRD patients under various therapies is needed. Vaccination recommendations should be updated on a regular basis, as more scientific data will become available.

Publication Type

Journal Article.

Year of Publication

2015

<303>

Unique Identifier

26205081

Title

Anti-B lymphocyte immunotherapy is associated with improvement of periodontal status in subjects with rheumatoid arthritis.

Source

Journal of Clinical Periodontology. 42(9):817-823, 2015 Sep.

VI 1

Status

MEDLINE

Authors

Coat J; Demoersman J; Beuzit S; Cornec D; Devauchelle-Pensec V; Saraux A; Pers JO.

Authors Full Name

Coat, Juliette; Demoersman, Julien; Beuzit, Sebastien; Cornec, Divi; Devauchelle-Pensec, Valerie; Saraux, Alain; Pers, Jacques-Olivier.

Institution

Coat, Juliette. EA2216, INSERM ESPRI, ERI29, Universite de Brest, Brest, France.

Coat, Juliette. LabEx IGO, Brest, France.

Demoersman, Julien. EA2216, INSERM ESPRI, ERI29, Universite de Brest, Brest, France.
Demoersman, Julien. LabEx IGO, Brest, France.
Beuzit, Sebastien. Department of Dentistry, Brest University Medical School Hospital, Brest, France.
Cornec, Divi. EA2216, INSERM ESPRI, ERI29, Universite de Brest, Brest, France.
Cornec, Divi. LabEx IGO, Brest, France.
Cornec, Divi. Department of Rheumatology, Brest University Medical School Hospital, Brest, France.
Devauchelle-Pensec, Valerie. EA2216, INSERM ESPRI, ERI29, Universite de Brest, Brest, France.
Devauchelle-Pensec, Valerie. LabEx IGO, Brest, France.
Devauchelle-Pensec, Valerie. Department of Rheumatology, Brest University Medical School Hospital, Brest, France.
Saraux, Alain. EA2216, INSERM ESPRI, ERI29, Universite de Brest, Brest, France.
Saraux, Alain. LabEx IGO, Brest, France.
Saraux, Alain. Department of Rheumatology, Brest University Medical School Hospital, Brest, France.
Pers, Jacques-Olivier. EA2216, INSERM ESPRI, ERI29, Universite de Brest, Brest, France.
Pers, Jacques-Olivier. LabEx IGO, Brest, France.
Pers, Jacques-Olivier. Department of Dentistry, Brest University Medical School Hospital, Brest, France.
Abstract
AIM: Rheumatoid arthritis (RA) and periodontitis present many similar features. The benefits of anti-B lymphocyte therapy (rituximab) on reducing tissue resorption in RA prompted us to assess its potential efficacy on the periodontal status of patients with RA treated with rituximab.

MATERIALS AND METHODS: Periodontal status was assessed in 21 subjects with RA, divided into two groups: Group I consisted in 11 subjects assessed before their first infusion of rituximab and again 6 months later. Five of them were also assessed for up to 4 years after their first rituximab infusion. The 10 subjects in group II had received more than two courses of two rituximab infusions at the time of periodontal assessment.

RESULTS: Pocket depth and attachment loss were significantly decreased 6 months after treatment with rituximab in group I. The periodontal status of the five subjects from group I followed for up to 48 months after rituximab treatment was improved irrespective of the clinical parameter observed. Patients from group II had a better periodontal status than patients from group I before treatment with rituximab.

CONCLUSIONS: Anti-B lymphocyte therapy could be beneficial to improve periodontitis suggesting a major role of B cells in this disease. Copyright © 2015 John Wiley & Sons A/S. Published by John Wiley & Sons Ltd.

Publication Type

Journal Article. Research Support, Non-U.S. Gov't.

Year of Publication

2015

<304>

Unique Identifier

26184541

Title

Uveitis in adults: What do rheumatologists need to know?. [Review]

Source

Joint, Bone, Spine: Revue du Rhumatisme. 82(5):308-14, 2015 Oct.

VI 1

Status

MEDLINE

Authors

Seve P; Kodjikian L; Adelaide L; Jamilloux Y.

Authors Full Name

Seve, Pascal; Kodjikian, Laurent; Adelaide, Leopold; Jamilloux, Yvan.

Institution

Seve, Pascal. Service de medecine interne, Hospices Civils de Lyon, Hopital de la Croix-Rousse, 103, Grande Rue de la Croix-Rousse, 69317 Lyon cedex 04, France; Universite de Lyon, 69361 Lyon cedex 07, France. Electronic address: pascal.seve@chu-lyon.fr.

Kodjikian, Laurent. Universite de Lyon, 69361 Lyon cedex 07, France; Service d'ophtalmologie, Hospices Civils de Lyon, Hopital de la Croix-Rousse, 103, Grande Rue de la Croix-Rousse, 69317 Lyon cedex 04, France.

Adelaide, Leopold. Service de medecine interne, Hospices Civils de Lyon, Hopital de la Croix-Rousse, 103, Grande Rue de la Croix-Rousse, 69317 Lyon cedex 04, France; Universite de Lyon, 69361 Lyon cedex 07, France.

Jamilloux, Yvan. Service de medecine interne, Hospices Civils de Lyon, Hopital de la Croix-Rousse, 103, Grande Rue de la Croix-Rousse, 69317 Lyon cedex 04, France; Universite de Lyon, 69361 Lyon cedex 07, France.
Abstract
Rheumatologists may need to establish the etiological diagnosis and handle the therapeutic management of adults with uveitis. To date, no diagnostic strategy for uveitis has been validated by prospective studies. Investigations are selected based on the clinical features and on the anatomic location of the ocular abnormalities. Infections such as syphilis, Lyme disease, tuberculosis, and Whipple's disease may cause uveitis, with concomitant joint inflammation in a few cases. In patients with a known history of chronic inflammatory joint disease, causes of uveitis include bisphosphonate therapy and immunodepression-related infections (e.g., due to Toxoplasma or a herpes virus). Sarcoidosis is an underestimated cause of uveitis, which occurs in 15% of cases, with a predilection for middle-aged women. In spondyloarthritis, uveitis is almost always acute, unilateral, and anterior. Among patients with uveitis and spondyloarthritis, about two thirds have their joint disease diagnosed during an evaluation for uveitis. Therefore, patients with inflammatory or noninflammatory back pain should be routinely evaluated for spondyloarthritis, which is the leading cause of uveitis in western countries. The risk of blindness is extremely low, and the main complication is recurrent uveitis, seen in 50% to 60% of cases. Sulfasalazine decreases the frequency, duration, and severity of uveitis and can be used prophylactically. Copyright © 2015. Published by Elsevier SAS.
Publication Type
Journal Article. Review.
Year of Publication
2015

<305>
Unique Identifier
26165113
Title
[Focusing on the use of biologics in rheumatoid arthritis]. [French]
Source
Revue du Praticien. 65(5):687-8, 2015 May.
VI 1

Status

MEDLINE

Authors

Devauchelle-Pensec V; Guellec D.

Authors Full Name

Devauchelle-Pensec, Valerie; Guellec, Dewi.

Publication Type

Journal Article.

Year of Publication

2015

<306>

Unique Identifier

26164595

Title

New insights into B cell biology in systemic lupus erythematosus and Sjogren's syndrome.

[Review]

Source

Current Opinion in Rheumatology. 27(5):461-7, 2015 Sep.

VI 1

Status

MEDLINE

Authors

Bird AK; Meednu N; Anolik JH.

Authors Full Name

Bird, Anna K; Meednu, Nida; Anolik, Jennifer H.

Institution

Bird, Anna K. Department of Medicine, Division of Allergy, Immunology, and Rheumatology, University of Rochester Medical Center, Rochester, New York, USA.

Abstract

PURPOSE OF REVIEW: Our understanding of the physiological and pathogenic functions of B cells in systemic lupus erythematosus (SLE) and Primary Sjogren's syndrome (pSS) continues to

expand. In this review, we discuss novel insights published in the last 18 months into the roles of B cells in systemic autoimmunity.

RECENT FINDINGS: Data have continued to expand regarding the diverse mechanisms by which innate immune signals including Toll-like receptors (TLRs) regulate the B cell compartment. Localized B cells and long-lived plasma cells have been identified as playing an important role in target tissue including the development of ectopic lymphoid structures in kidney and salivary gland. In addition to pathogenic roles for B cells, there is mounting evidence for regulatory B cell subsets that play a protective role and new insights into the signals that regulate their development.

SUMMARY: The past few years have provided insights into the multiple paths by which innate immune signals can lead to B cell activation in SLE and pSS and the increasingly diverse ways in which B cells contribute to disease expression. Further understanding the imbalance between protective and pathogenic functions for B cells in disease including in understudied target tissue should yield new treatment approaches.

Publication Type

Journal Article. Research Support, N.I.H., Extramural. Research Support, Non-U.S. Gov't. Review.

Year of Publication

2015

<307>

Unique Identifier

26140604

Title

Stem Cell Therapies in Clinical Trials: Progress and Challenges. [Review]

Source

Cell Stem Cell. 17(1):11-22, 2015 Jul 02.

VI 1

Status

MEDLINE

Authors

Trounson A; McDonald C.
Authors Full Name
Trounson, Alan; McDonald, Courtney.
Institution
Trounson, Alan. Hudson Institute for Medical Research, 27-31 Wright Street, Clayton, VIC 3168, Australia. Electronic address: alan.trounson@hudson.org.au.
McDonald, Courtney. Hudson Institute for Medical Research, 27-31 Wright Street, Clayton, VIC 3168, Australia.
Abstract
Clinical investigations using stem cell products in regenerative medicine are addressing a wide spectrum of conditions using a variety of stem cell types. To date, there have been few reports of safety issues arising from autologous or allogeneic transplants. Many cells administered show transient presence for a few days with trophic influences on immune or inflammatory responses. Limbal stem cells have been registered as a product for eye burns in Europe and mesenchymal stem cells have been approved for pediatric graft versus host disease in Canada and New Zealand. Many other applications are progressing in trials, some with early benefits to patients.
Copyright © 2015 Elsevier Inc. All rights reserved.
Publication Type
Journal Article. Review.
Year of Publication
2015

<308>
Unique Identifier
26100972
Title
Rheumatoid arthritis: First-in-human phase I trial of DC immunotherapy for early RA.
Source
Nature Reviews Rheumatology. 11(8):443, 2015 Aug.
VI 1
Status
MEDLINE
Authors

Buckland J.

Authors Full Name

Buckland, Jenny.

Comments

Comment on (CON)

Publication Type

Journal Article. Comment.

Year of Publication

2015

<309>

Unique Identifier

26062995

Title

Polyfunctional, Pathogenic CD161+ Th17 Lineage Cells Are Resistant to Regulatory T Cell-Mediated Suppression in the Context of Autoimmunity.

Source

Journal of Immunology. 195(2):528-40, 2015 Jul 15.

VI 1

Status

MEDLINE

Authors

Basdeo SA; Moran B; Cluxton D; Canavan M; McCormick J; Connolly M; Orr C; Mills KH; Veale DJ; Fearon U; Fletcher JM.

Authors Full Name

Basdeo, Sharee A; Moran, Barry; Cluxton, Deborah; Canavan, Mary; McCormick, Jennifer; Connolly, Mary; Orr, Carl; Mills, Kingston H G; Veale, Douglas J; Fearon, Ursula; Fletcher, Jean M.

Institution

Basdeo, Sharee A. School of Biochemistry and Immunology, Trinity Biomedical Sciences Institute, Trinity College Dublin, Dublin 2, Ireland;

Moran, Barry. School of Biochemistry and Immunology, Trinity Biomedical Sciences Institute, Trinity College Dublin, Dublin 2, Ireland;

Cluxton, Deborah. School of Biochemistry and Immunology, Trinity Biomedical Sciences Institute, Trinity College Dublin, Dublin 2, Ireland;
Canavan, Mary. Department of Rheumatology, Dublin Academic Medical Centre, St. Vincent's University Hospital, Dublin 4, Ireland; and.
McCormick, Jennifer. Department of Rheumatology, Dublin Academic Medical Centre, St. Vincent's University Hospital, Dublin 4, Ireland; and.
Connolly, Mary. Department of Rheumatology, Dublin Academic Medical Centre, St. Vincent's University Hospital, Dublin 4, Ireland; and.
Orr, Carl. Department of Rheumatology, Dublin Academic Medical Centre, St. Vincent's University Hospital, Dublin 4, Ireland; and.
Mills, Kingston H G. School of Biochemistry and Immunology, Trinity Biomedical Sciences Institute, Trinity College Dublin, Dublin 2, Ireland;
Veale, Douglas J. Department of Rheumatology, Dublin Academic Medical Centre, St. Vincent's University Hospital, Dublin 4, Ireland; and.
Fearon, Ursula. Department of Rheumatology, Dublin Academic Medical Centre, St. Vincent's University Hospital, Dublin 4, Ireland; and.
Fletcher, Jean M. School of Biochemistry and Immunology, Trinity Biomedical Sciences Institute, Trinity College Dublin, Dublin 2, Ireland; School of Medicine, Trinity Biomedical Sciences Institute, Trinity College Dublin, Dublin 2, Ireland jean.fletcher@tcd.ie.
Abstract
In autoimmune diseases such as rheumatoid arthritis (RA), regulatory T cells (Tregs) fail to constrain autoimmune inflammation; however, the reasons for this are unclear. We investigated T cell regulation in the RA joint. Tregs from RA synovial fluid suppressed autologous responder T cells; however, when compared with Tregs from healthy control peripheral blood, they were significantly less suppressive. Despite their reduced suppressive activity, Tregs in the RA joint were highly proliferative and expressed FOXP3, CD39, and CTLA-4, which are markers of functional Tregs. This suggested that the reduced suppression is due to resistance of RA synovial fluid responder T cells to Treg inhibition. CD161(+) Th17 lineage cells were significantly enriched in the RA joint; we therefore investigated their relative susceptibility to Treg-mediated suppression. Peripheral blood CD161(+) Th cells from healthy controls were significantly more resistant to Treg-mediated suppression, when compared with CD161(-) Th cells, and this was mediated through a STAT3-dependant mechanism. Furthermore, depletion of CD161(+) Th cells from the responder T cell population in RA synovial fluid restored Treg-mediated suppression. In addition, CD161(+) Th cells exhibited pathogenic features, including polyfunctional proinflammatory cytokine production, an ability to activate synovial fibroblasts, and to survive and persist in the inflamed and hypoxic joint. Because CD161(+) Th cells are known to be enriched at sites of autoinflammation, our finding that they are highly proinflammatory and resistant to Treg-

mediated suppression suggests an important pathogenic role in RA and other autoimmune diseases. Copyright © 2015 by The American Association of Immunologists, Inc.
Publication Type
Journal Article. Research Support, Non-U.S. Gov't.
Year of Publication
2015

<310>
Unique Identifier
26057192
Title
The role of autoreactive T cell in the pathogenesis of rheumatoid arthritis and implications for T cell targeted vaccine therapy. [Review]
Source
Minerva Medica. 106(3):157-67, 2015 Jun.
VI 1
Status
MEDLINE
Authors
Wang D; Li Y; Liu Y; Shi G.
Authors Full Name
Wang, D; Li, Y; Liu, Y; Shi, G.
Institution
Wang, D. Molecular Biology Research Center, Shandong Medical College, Linyi, Shandong, China - gshi@xmu.edu.cn.
Abstract
Rheumatoid arthritis (RA) is a systemic autoimmune disease characterised by chronic inflammation of joint synovial tissue and subsequent destruction of associated bone, cartilage and soft tissues. RA is commonly treated with non-steroidal anti-inflammatory drugs (NSAIDs), traditional disease-modifying antirheumatic drugs (DMARDs), glucocorticoids and biologic inhibitors of TNF, IL-1, IL-6, T cells and B cells. The use of these drugs especially biological agents has greatly improved the treatment of RA. Although the pathogenesis of RA remains unclear, T-cell mediated immune response is considered as a critical contributor in RA initiation

and progression. It has been hypothesized that arthritogenic T cells (autoreactive T cells) escaping negative selection can recognize arthritogenic antigens and lead to autoimmunity and tissue destruction. Due to the important role of autoreactive T cells in the mechanisms of RA, they might be a novel therapeutic target. Many vaccines targeting autoreactive T cells which can establish immunological self tolerance have been developed. The efficacy of these vaccines has been justified in experimental models of RA and clinical trials. Inhibition of autoreactive T cell response by vaccination might provide a new treatment opinion in RA.
Publication Type
  Journal Article. Research Support, Non-U.S. Gov't. Review.
Year of Publication
  2015

<311>
Unique Identifier
  26047509
Title
  Expansion of Activated Peripheral Blood Memory B Cells in Rheumatoid Arthritis, Impact of B Cell Depletion Therapy, and Biomarkers of Response.
Source
  PLoS ONE [Electronic Resource]. 10(6):e0128269, 2015.
VI 1
Status
  MEDLINE
Authors
  Adlowitz DG; Barnard J; Biear JN; Cistrone C; Owen T; Wang W; Palanichamy A; Ezealah E; Campbell D; Wei C; Looney RJ; Sanz I; Anolik JH.
Authors Full Name
  Adlowitz, Diana G; Barnard, Jennifer; Biear, Jamie N; Cistrone, Christopher; Owen, Teresa; Wang, Wensheng; Palanichamy, Arumugam; Ezealah, Ezinma; Campbell, Debbie; Wei, Chungwen; Looney, R John; Sanz, Inaki; Anolik, Jennifer H.
Institution
  Adlowitz, Diana G. Department of Medicine, Division of Allergy, Immunology and Rheumatology, University of Rochester Medical Center, Rochester, New York, 14642, United States of America.

Barnard, Jennifer. Department of Medicine, Division of Allergy, Immunology and Rheumatology, University of Rochester Medical Center, Rochester, New York, 14642, United States of America.

Biear, Jamie N. Department of Medicine, Division of Allergy, Immunology and Rheumatology, University of Rochester Medical Center, Rochester, New York, 14642, United States of America.

Cistrone, Christopher. Department of Medicine, Division of Allergy, Immunology and Rheumatology, University of Rochester Medical Center, Rochester, New York, 14642, United States of America.

Owen, Teresa. Department of Medicine, Division of Allergy, Immunology and Rheumatology, University of Rochester Medical Center, Rochester, New York, 14642, United States of America.

Wang, Wensheng. Department of Medicine, Division of Allergy, Immunology and Rheumatology, University of Rochester Medical Center, Rochester, New York, 14642, United States of America.

Palanichamy, Arumugam. Department of Medicine, Division of Allergy, Immunology and Rheumatology, University of Rochester Medical Center, Rochester, New York, 14642, United States of America.

Ezealah, Ezinma. Department of Medicine, Division of Allergy, Immunology and Rheumatology, University of Rochester Medical Center, Rochester, New York, 14642, United States of America.

Campbell, Debbie. Department of Medicine, Division of Allergy, Immunology and Rheumatology, University of Rochester Medical Center, Rochester, New York, 14642, United States of America.

Wei, Chungwen. Department of Medicine, Emory University, Atlanta, Georgia, 30332, United States of America.

Looney, R John. Department of Medicine, Division of Allergy, Immunology and Rheumatology, University of Rochester Medical Center, Rochester, New York, 14642, United States of America.

Sanz, Inaki. Department of Medicine, Emory University, Atlanta, Georgia, 30332, United States of America.

Anolik, Jennifer H. Department of Medicine, Division of Allergy, Immunology and Rheumatology, University of Rochester Medical Center, Rochester, New York, 14642, United States of America.

Abstract

Although B cell depletion therapy (BCDT) is effective in a subset of rheumatoid arthritis (RA) patients, both mechanisms and biomarkers of response are poorly defined. Here we characterized abnormalities in B cell populations in RA and the impact of BCDT in order to elucidate B cell roles in the disease and response biomarkers. In active RA patients both CD27+IgD- switched memory (SM) and CD27-IgD- double negative memory (DN) peripheral blood B cells contained significantly higher fractions of CD95+ and CD21- activated cells compared to healthy controls. After BCD the predominant B cell populations were memory, and

residual memory B cells displayed a high fraction of CD21- and CD95+ compared to pre-depletion indicating some resistance of these activated populations to anti-CD20. The residual memory populations also expressed more Ki-67 compared to pre-treatment, suggesting homeostatic proliferation in the B cell depleted state. Biomarkers of clinical response included lower CD95+ activated memory B cells at depletion time points and a higher ratio of transitional B cells to memory at reconstitution. B cell function in terms of cytokine secretion was dependent on B cell subset and changed with BCD. Thus, SM B cells produced pro-inflammatory (TNF) over regulatory (IL10) cytokines as compared to naive/transitional. Notably, B cell TNF production decreased after BCDT and reconstitution compared to untreated RA. Our results support the hypothesis that the clinical and immunological outcome of BCDT depends on the relative balance of protective and pathogenic B cell subsets established after B cell depletion and repopulation.
Publication Type
Journal Article. Research Support, N.I.H., Extramural.
Year of Publication
2015

<312>
Unique Identifier
26041704
Title
Citrullinated peptide dendritic cell immunotherapy in HLA risk genotype-positive rheumatoid arthritis patients.
Source
Science Translational Medicine. 7(290):290ra87, 2015 Jun 03.
VI 1
Status
MEDLINE
Authors
Benham H; Nel HJ; Law SC; Mehdi AM; Street S; Ramnoruth N; Pahau H; Lee BT; Ng J; Brunck ME; Hyde C; Trouw LA; Dudek NL; Purcell AW; O'Sullivan BJ; Connolly JE; Paul SK; Le Cao KA; Thomas R.
Authors Full Name

Benham, Helen; Nel, Hendrik J; Law, Soi Cheng; Mehdi, Ahmed M; Street, Shayna; Ramnoruth, Nishta; Pahau, Helen; Lee, Bernett T; Ng, Jennifer; Brunck, Marion E G; Hyde, Claire; Trouw, Leendert A; Dudek, Nadine L; Purcell, Anthony W; O'Sullivan, Brendan J; Connolly, John E; Paul, Sanjoy K; Le Cao, Kim-Anh; Thomas, Ranjeny.

Institution

Benham, Helen. University of Queensland Diamantina Institute, Translational Research Institute, Princess Alexandra Hospital, Woolloongabba, Queensland 4102, Australia. University of Queensland School of Medicine, Brisbane, Queensland 4102, Australia.

Nel, Hendrik J. University of Queensland Diamantina Institute, Translational Research Institute, Princess Alexandra Hospital, Woolloongabba, Queensland 4102, Australia.

Law, Soi Cheng. University of Queensland Diamantina Institute, Translational Research Institute, Princess Alexandra Hospital, Woolloongabba, Queensland 4102, Australia.

Mehdi, Ahmed M. University of Queensland Diamantina Institute, Translational Research Institute, Princess Alexandra Hospital, Woolloongabba, Queensland 4102, Australia.

Street, Shayna. University of Queensland Diamantina Institute, Translational Research Institute, Princess Alexandra Hospital, Woolloongabba, Queensland 4102, Australia.

Ramnoruth, Nishta. University of Queensland Diamantina Institute, Translational Research Institute, Princess Alexandra Hospital, Woolloongabba, Queensland 4102, Australia.

Pahau, Helen. University of Queensland Diamantina Institute, Translational Research Institute, Princess Alexandra Hospital, Woolloongabba, Queensland 4102, Australia.

Lee, Bernett T. Singapore Immunology Network, Agency for Science, Technology and Research, 8A Biomedical Grove, Immunos Building, Level 3, Biopolis, 138673 Singapore, Singapore.

Ng, Jennifer. University of Queensland Diamantina Institute, Translational Research Institute, Princess Alexandra Hospital, Woolloongabba, Queensland 4102, Australia.

Brunck, Marion E G. University of Queensland Diamantina Institute, Translational Research Institute, Princess Alexandra Hospital, Woolloongabba, Queensland 4102, Australia.

Hyde, Claire. University of Queensland Diamantina Institute, Translational Research Institute, Princess Alexandra Hospital, Woolloongabba, Queensland 4102, Australia.

Trouw, Leendert A. Department of Rheumatology, Leiden University Medical Center, Leiden 2333, Netherlands.

Dudek, Nadine L. Department of Biochemistry and Molecular Biology, School of Biomedical Sciences, Monash University, Clayton, Victoria 3800, Australia.

Purcell, Anthony W. Department of Biochemistry and Molecular Biology, School of Biomedical Sciences, Monash University, Clayton, Victoria 3800, Australia.

O'Sullivan, Brendan J. University of Queensland Diamantina Institute, Translational Research Institute, Princess Alexandra Hospital, Woolloongabba, Queensland 4102, Australia.

Connolly, John E. Singapore Immunology Network, Agency for Science, Technology and Research, 8A Biomedical Grove, Immunos Building, Level 3, Biopolis, 138673 Singapore, Singapore.
Paul, Sanjoy K. Queensland Clinical Trials & Biostatistics Centre, School of Population Health, The University of Queensland, Brisbane, Queensland 4006, Australia.
Le Cao, Kim-Anh. University of Queensland Diamantina Institute, Translational Research Institute, Princess Alexandra Hospital, Woolloongabba, Queensland 4102, Australia.
Thomas, Ranjeny. University of Queensland Diamantina Institute, Translational Research Institute, Princess Alexandra Hospital, Woolloongabba, Queensland 4102, Australia. ranjeny.thomas@uq.edu.au.
Comments
Comment in (CIN)
Abstract
In animals, immunomodulatory dendritic cells (DCs) exposed to autoantigen can suppress experimental arthritis in an antigen-specific manner. In rheumatoid arthritis (RA), disease-specific anti-citrullinated peptide autoantibodies (ACPA or anti-CCP) are found in the serum of about 70% of RA patients and are strongly associated with HLA-DRB1 risk alleles. This study aimed to explore the safety and biological and clinical effects of autologous DCs modified with a nuclear factor kappaB (NF-kappaB) inhibitor exposed to four citrullinated peptide antigens, designated "Rheumavax," in a single-center, open-labeled, first-in-human phase 1 trial. Rheumavax was administered once intradermally at two progressive dose levels to 18 human leukocyte antigen (HLA) risk genotype-positive RA patients with citrullinated peptide-specific autoimmunity. Sixteen RA patients served as controls. Rheumavax was well tolerated: adverse events were grade 1 (of 4) severity. At 1 month after treatment, we observed a reduction in effector T cells and an increased ratio of regulatory to effector T cells; a reduction in serum interleukin-15 (IL-15), IL-29, CX3CL1, and CXCL11; and reduced T cell IL-6 responses to vimentin(447-455)-Cit450 relative to controls. Rheumavax did not induce disease flares in patients recruited with minimal disease activity, and DAS28 decreased within 1 month in Rheumavax-treated patients with active disease. This exploratory study demonstrates safety and biological activity of a single intradermal injection of autologous modified DCs exposed to citrullinated peptides, and provides rationale for further studies to assess clinical efficacy and antigen-specific effects of autoantigen immunomodulatory therapy in RA. Copyright © 2015, American Association for the Advancement of Science.
Publication Type
Journal Article. Research Support, Non-U.S. Gov't.
Year of Publication
2015

<313>
Unique Identifier
26025339
Title
Vaccinations in paediatric rheumatology: an update on current developments. [Review]
Source
Current Rheumatology Reports. 17(7):46, 2015 Jul.
VI 1
Status
MEDLINE
Authors
Groot N; Heijstek MW; Wulffraat NM.
Authors Full Name
Groot, Noortje; Heijstek, Marloes W; Wulffraat, Nico M.
Institution
Groot, Noortje. Department of Pediatrics, Wilhemina Children's Hospital/University Medical Centre Utrecht (UMCU), PO-box 85090, 3508 AB, Utrecht, The Netherlands, n.groot@erasmusmc.nl.
Abstract
In 2011, the European League Against Rheumatism (EULAR) published recommendations regarding the vaccination of children with rheumatic diseases. These recommendations were based on a systematic literature review published in that same year. Since then, the evidence body on this topic has grown substantially. This review provides an update of the systematic literature study of 2011, summarizing all the available evidence on the safety and immunogenicity of vaccination in paediatric patients with rheumatic diseases. The current search yielded 21 articles, in addition to the 27 articles described in the 2011 review. In general, vaccines are immunogenic and safe in this patient population. The effect of immunosuppressive drugs on the immunogenicity of vaccines was not detrimental for glucocorticosteroids and methotrexate. Biologicals could accelerate a waning of antibody levels over time, although most patients were initially protected adequately. Overall, persistence of immunological memory may be reduced in children with rheumatic diseases, which shows the need for (booster) vaccination. This update of the 2011 systematic literature review strengthens the evidence base for the EULAR

recommendations, and it must be concluded that vaccinations in patients with rheumatic diseases should be advocated.

Publication Type

Journal Article. Review. Systematic Review.

Year of Publication

2015

<314>

Unique Identifier

25986458

Title

The association between antibody levels before and after 7-valent pneumococcal conjugate vaccine immunization and subsequent pneumococcal infection in chronic arthritis patients.

Source

Arthritis Research & Therapy. 17:124, 2015 May 19.

VI 1

Status

MEDLINE

Authors

Nagel J; Geborek P; Saxne T; Jonsson G; Englund M; Petersson IF; Nilsson JA; Truedsson L; Kapetanovic MC.

Authors Full Name

Nagel, Johanna; Geborek, Pierre; Saxne, Tore; Jonsson, Goran; Englund, Martin; Petersson, Ingemar F; Nilsson, Jan-Ake; Truedsson, Lennart; Kapetanovic, Meliha C.

Institution

Nagel, Johanna. Department of Clinical Sciences, Lund, Section of Rheumatology, Lund University, and Skane University Hospital, Kioskgatan 3, SE-221 85, Lund, Sweden. johanna.bengtsson.nagel@gmail.com.

Geborek, Pierre. Department of Clinical Sciences, Lund, Section of Rheumatology, Lund University, and Skane University Hospital, Kioskgatan 3, SE-221 85, Lund, Sweden. pierre.geborek@med.lu.se.

Saxne, Tore. Department of Clinical Sciences, Lund, Section of Rheumatology, Lund University, and Skane University Hospital, Kioskgatan 3, SE-221 85, Lund, Sweden. tore.saxne@med.lu.se.

Jonsson, Goran. Department of Clinical Sciences Lund, Section of Infectious Diseases, Lund University and Skane University Hospital, Kioskgatan 3, SE-221 85, Lund, Sweden. goran.b.jonsson@skane.se.

Englund, Martin. Epidemiology and Register Centre South, Skane University Hospital, Lund, and Orthopaedics, Department of Clinical Sciences Lund, Lund University, Barngatan 2B, Lund, SE-221 85, Sweden. martin.englund@med.lu.se.

Englund, Martin. Clinical Epidemiology Research & Training Unit, Boston University School of Medicine, 650 Albany Street, Boston, MA, 02118, USA. martin.englund@med.lu.se.

Petersson, Ingemar F. Epidemiology and Register Centre South, Skane University Hospital, Lund, and Orthopaedics, Department of Clinical Sciences Lund, Lund University, Barngatan 2B, Lund, SE-221 85, Sweden. ingemar.petersson@med.lu.se.

Nilsson, Jan-Ake. Department of Clinical Sciences, Lund, Section of Rheumatology, Lund University, and Skane University Hospital, Kioskgatan 3, SE-221 85, Lund, Sweden. jan-ake.nilsson@med.lu.se.

Truedsson, Lennart. Department of Laboratory Medicine, Section of Microbiology and Immunology, Lund University, Solvegatan 23, Lund, SE-223 62, Sweden. lennart.truedsson@med.lu.se.

Kapetanovic, Meliha C. Department of Clinical Sciences, Lund, Section of Rheumatology, Lund University, and Skane University Hospital, Kioskgatan 3, SE-221 85, Lund, Sweden. meliha.c_kapetanovic@med.lu.se.

Abstract

INTRODUCTION: The aim of present study is to inverstigate the association between antibody levels after vaccination with 7-valent pneumococcal conjugate vaccine (PCV7) and subsequent serious pneumococcal infections in rheumatoid arthritis (RA) and spondylarthropathy (SpA) patients.

METHODS: A cohort of 497 patients (RA=248 and SpA=249) received a single dose of PCV7. At vaccination, patients were treated with methotrexate (MTX; n=85), anti-tumour necrosis factor (anti-TNF) + MTX (n=169), anti-TNF monotherapy (n=158) and non-steroidal anti-inflammatory drugs (NSAIDs)/analgesics (n=85). Antibody levels of serotypes 6B and 23B were analyzed before and 4 to 6 weeks after vaccination using standard enzyme-linked immunosorbent assay (ELISA). Serious pneumococcal infections (pneumonia/lower respiratory tract infection, meningitis, sepsis, septic arthritis) occurring within 4.5 years after vaccination were identified in the Skane Healthcare Register using the International Classification of Diseases, tenth revision

(ICD-10) codes. The association between post-vaccination antibody levels and protection against infections and determination of protective cutoff levels was explored using receiver operating characteristic (ROC) curves. Predictors of infection were studied using regression analyses.

RESULTS: Eighteen infections were registered in 15 patients before vaccination and 27 infections in 23 patients after vaccination. Patients with serious infections after vaccination had significantly lower post-vaccination antibody titres for both 6B (P=0.04) and 23 F (P=0.04). Post-vaccination antibody levels of at least 1.29 mg/L and 1.01 mg/L for 6B and 23, respectively, were associated with better protection from serious infections. Higher age, concomitant prednisolone but not MTX or anti-TNF were associated with such infections.

CONCLUSIONS: Patients with more robust antibody responses after vaccination with pneumococcal conjugate vaccine were less likely to suffer from serious infections. High age and prednisolone at vaccination were associated with putative serious pneumococcal infections in this cohort.

TRIAL REGISTRATION NUMBER: EudraCT EU 2007-006539-29 and NCT00828997 . Registered 23 January 2009.

Publication Type

Journal Article. Randomized Controlled Trial. Research Support, Non-U.S. Gov't.

Year of Publication

2015

<315>

Unique Identifier

25962385

Title

Transplantation of human amnion mesenchymal cells attenuates the disease development in rats with collagen-induced arthritis.

Source

Clinical & Experimental Rheumatology. 33(4):484-90, 2015 Jul-Aug.

VI 1

Status

MEDLINE

Authors

Shu J; Pan L; Huang X; Wang P; Li H; He X; Cai Z.

Authors Full Name

Shu, Jun; Pan, Lin; Huang, Xiaojie; Wang, Ping; Li, Hong; He, Xiaojuan; Cai, Zhe.

Institution

Shu, Jun. Institute of Clinical Medical Science, China-Japan Friendship Hospital, Beijing, China.

Pan, Lin. Institute of Clinical Medical Science, China-Japan Friendship Hospital, Beijing, China.

Huang, Xiaojie. Institute of Clinical Medical Science, China-Japan Friendship Hospital, Beijing, China.

Wang, Ping. Institute of Clinical Medical Science, China-Japan Friendship Hospital, Beijing, China.

Li, Hong. Institute of Clinical Medical Science, China-Japan Friendship Hospital, Beijing, China.

He, Xiaojuan. Institute of Basic Research in Clinical Medicine, China Academy of Chinese Medical Sciences, Beijing, China.

Cai, Zhe. Institute of Clinical Medical Science, China-Japan Friendship Hospital, Beijing, China.

Abstract

OBJECTIVES: Human amnion mesenchymal cells (hAMCs), isolated from the amniotic membrane of human placenta, are a unique population of mesenchymal stem cells (MSCs). Recent studies indicated that hAMCs had immunosuppressive functions and might be used in treatment of some autoimmune diseases. The aim of this study is to explore the feasibility of using hAMCs for treatment rats with collagen-induced arthritis (CIA), a classic animal model for human rheumatoid arthritis.

METHODS: SD rats were immunised with type II collagen and Freund's incomplete adjuvant. hAMCs were injected intraperitoneal when arthritis had become established. The arthritis was evaluated macroscopically and microscopically. Serum levels of IFN-gamma, TNF-alpha, SOD, MDA, GSH-Px and T-AOC were detected by commercially assay kits. CD4+/CD8+ T-cell ratio in peripheral blood was examined by flow cytometry. Proliferation of splenocytes was evaluated using MTT assay.

RESULTS: The results demonstrated that application of hAMCs significantly ameliorated severity of arthritis and decreased the histopathological changes in CIA rats. Consistently, production of proinflammatory cytokines such as IFN-gamma and TNF-alpha was dramatically inhibited. Moreover, hAMCs exerted anti-oxidative capacity by significantly raising the levels of SOD, GSH-Px, T-AOC and lowering the level of MDA. In addition, hAMCs also remarkably

restored CD4+/CD8+ T-cell ratio and induced hyporesponsiveness of T lymphocytes by inhibiting their active proliferation. Finally, hAMCs had no obvious side effect on CIA rats.

CONCLUSIONS: In conclusion, our results indicated that hAMCs could attenuate the disease development in rats with CIA, which might be a promising cell source for therapy of rheumatoid arthritis.

Publication Type

Journal Article.

Year of Publication

2015

<316>

Unique Identifier

25959608

Title

TNF signals are dispensable for the generation of CD23+ CD21/35-high CD1d-high B cells in inflamed lymph nodes.

Source

Cellular Immunology. 296(2):133-7, 2015 Aug.

VI 1

Status

MEDLINE

Authors

Kuzin II; Bouta EM; Schwarz EM; Bottaro A.

Authors Full Name

Kuzin, Igor I; Bouta, Echoe M; Schwarz, Edward M; Bottaro, Andrea.

Institution

Kuzin, Igor I. Dept. of Biomedical Sciences, Cooper Medical School of Rowan University, Camden, NJ, USA.

Bouta, Echoe M. Center for Musculoskeletal Research, Dept. of Biomedical Engineering, and Dept. of Orthopaedics, University of Rochester School of Medicine and Dentistry, Rochester, NY, USA.

Schwarz, Edward M. Center for Musculoskeletal Research, Dept. of Biomedical Engineering, and Dept. of Orthopaedics, University of Rochester School of Medicine and Dentistry, Rochester, NY, USA.
Bottaro, Andrea. Dept. of Biomedical Sciences, Cooper Medical School of Rowan University, Camden, NJ, USA. Electronic address: bottaro@rowan.edu.
Abstract
Tumor necrosis factor (TNF) is a key cytokine in rheumatoid arthritis (RA) pathogenesis, as underscored by the clinical effectiveness of TNF antagonists. While several of TNF's key targets in RA are well understood, its many pleiotropic effects remain to be elucidated. TNF-transgenic mice develop inflammatory-erosive arthritis associated with disruption of draining lymph node histology and function, and accumulation of B cells with unique phenotypic and functional features consistent with contribution to pathogenesis (B cells in inflamed nodes, Bin). Bin cell induction depends on the inflamed microenvironment, but the specific signals are unknown. Using anti-TNF treatment and TNF-receptor-deficient mice, here we show that Bin cells are induced and maintained independently of B cell-intrinsic TNF signals. Copyright © 2015 Elsevier Inc. All rights reserved.
Publication Type
Journal Article. Research Support, N.I.H., Extramural.
Year of Publication
2015

<317>
Unique Identifier
25926287
Title
A canine-specific anti-nerve growth factor antibody alleviates pain and improves mobility and function in dogs with degenerative joint disease-associated pain.
Source
BMC Veterinary Research [Electronic Resource]. 11:101, 2015 Apr 30.
VI 1
Status
MEDLINE
Authors

Lascelles BD; Knazovicky D; Case B; Freire M; Innes JF; Drew AC; Gearing DP.
Authors Full Name
Lascelles, B Duncan X; Knazovicky, David; Case, Beth; Freire, Mila; Innes, John F; Drew, Alexander C; Gearing, David P.
Institution
Lascelles, B Duncan X. Comparative Pain Research Laboratory, Department of Clinical Sciences, College of Veterinary Medicine, North Carolina State University, Raleigh, NC, USA. duncan_lascelles@ncsu.edu.
Lascelles, B Duncan X. Center for Comparative Medicine and Translational Research, College of Veterinary Medicine, North Carolina State University, Raleigh, NC, USA. duncan_lascelles@ncsu.edu.
Lascelles, B Duncan X. Center for Pain Research and Innovation, UNC School of Dentistry, Chapel Hill, NC, USA. duncan_lascelles@ncsu.edu.
Knazovicky, David. Comparative Pain Research Laboratory, Department of Clinical Sciences, College of Veterinary Medicine, North Carolina State University, Raleigh, NC, USA. david_knazovicky@ncsu.edu.
Case, Beth. Comparative Pain Research Laboratory, Department of Clinical Sciences, College of Veterinary Medicine, North Carolina State University, Raleigh, NC, USA. bcc@ncsu.edu.
Freire, Mila. Faculte de medecine veterinaire, Universite de Montreal, Saint-Hyacinthe, QC, Canada. m.freire.gonzalez@umontreal.ca.
Innes, John F. ChesterGates Referral Hospital, Chester, UK. john.innes@cvsvets.com.
Drew, Alexander C. Nexvet Australia Pty Ltd, Melbourne, VIC, Australia. alec.drew@nexvet.com.
Gearing, David P. Nexvet Australia Pty Ltd, Melbourne, VIC, Australia. dave.gearing@nexvet.com.
Abstract
BACKGROUND: There is a critical need for proven drugs other than non-steroidal anti-inflammatory drugs for treatment of degenerative joint disease (DJD) pain in dogs. Antibodies against nerve growth factor (NGF) are analgesic in rodent models and in humans with DJD. This pilot study aimed to evaluate the efficacy of a novel caninised anti-NGF antibody (NV-01) for the treatment of DJD pain in dogs. In a randomized, parallel group, stratified, double masked, placebo controlled, proof of principle clinical pilot study design, 26 dogs with DJD received NV-01 (200 mcg/kg IV) or placebo on day 0 (D0). In addition to objective accelerometry measures, owners completed clinical metrology instruments (Client-Specific Outcome Measures [CSOM], Canine Brief Pain Inventory [CBPI] and Liverpool Osteoarthritis in Dogs Index [LOAD]) on D0, D14 and D28. CBPI subscales (pain severity [PS] and pain interference [PI]), CSOM and LOAD

scores were evaluated within and between groups for change over time. Recognized success/failure criteria were applied and success compared between groups.

RESULTS: CBPI PS and PI scores significantly improved in the NV-01 group (PS: D0-14, P = 0.012 and D0-28, P = 0.019; PI: D0-14, P = 0.012 and D0-28, P = 0.032) but not in the placebo group. CSOM scores showed similar patterns with a significant difference between within-group changes at D14 and D28 (P = 0.038 and P = 0.009, respectively), and significantly more successes at D28 (P = 0.047). LOAD scores significantly improved in the NV-01 group (D0-14, P = 0.004 and D0-28, P = 0.002) but not in the placebo group. There were significant differences between the groups for change in LOAD score at D14 (P = 0.014) and D28 (P = 0.033). No side effects were noted. Activity in the NV-01 group increased over the study period compared to placebo (P = 0.063) and the difference between the groups for change in activity over the time period 9am-5pm (8 hours) was significant (P = 0.006).

CONCLUSIONS: These pilot data demonstrate a positive analgesic effect of anti-NGF antibody in dogs suffering from chronic pain. The magnitude of the effect appeared identical to that expected with an NSAID.

Publication Type

Journal Article. Randomized Controlled Trial. Research Support, Non-U.S. Gov't.

Year of Publication

2015

<318>

Unique Identifier

25917627

Title

Investigating the potential side effects of anti-TNF therapy for rheumatoid arthritis: cause for concern?. [Review]

Source

Immunotherapy. 7(4):353-61, 2015.

VI 1

Status

MEDLINE

Authors
  Atzeni F; Gianturco L; Talotta R; Varisco V; Ditto MC; Turiel M; Sarzi-Puttini P.
Authors Full Name
  Atzeni, Fabiola; Gianturco, Luigi; Talotta, Rossella; Varisco, Valentina; Ditto, Maria Chiara; Turiel, Maurizio; Sarzi-Puttini, Piercarlo.
Institution
  Atzeni, Fabiola. IRCCS Galeazzi Orthopedic Institute, Milan, Italy.
Abstract
  There are now five anti-TNF drugs available for clinical use, and it will not be long before they are joined by biosimilar drugs. Some patients treated with selective TNF drugs may develop adverse events such as infections, malignancies, acute infusion and injection reactions, autoimmunity and cardiovascular effects. Registry data consistently show that, particularly during the first 6 months, anti-TNF drugs slightly increase the risk of serious infections of the skin, soft tissues and joints, but it does not seem to increase the risk of cancer other than nonmelanoma skin cancers. A number of studies have shown that the administration of biological agents can lead to the formation of neutralizing and nonneutralizing antibodies. Lipid levels increase, but the atherogenic index remains stable and qualitative changes to lipid particles may reduce the risk of cardiovascular diseases. Patients treated with anti-TNF drugs therefore need to be monitored regularly.
Publication Type
  Journal Article. Review.
Year of Publication
  2015

<319>
Unique Identifier
  25887268
Title
  SND-117, a sinomenine bivalent alleviates type II collagen-induced arthritis in mice.
Source
  International Immunopharmacology. 26(2):423-31, 2015 Jun.
VI 1
Status

MEDLINE

Authors

Zhou YR; Zhao Y; Bao BH; Li JX.

Authors Full Name

Zhou, Yu-Ren; Zhao, Yang; Bao, Bei-Hua; Li, Jian-Xin.

Institution

Zhou, Yu-Ren. Key Lab of Analytical Chemistry for Life Science, School of Chemistry and Chemical Engineering, Nanjing University, Nanjing 210093, PR China.

Zhao, Yang. Key Lab of Analytical Chemistry for Life Science, School of Chemistry and Chemical Engineering, Nanjing University, Nanjing 210093, PR China.

Bao, Bei-Hua. Key Lab of Analytical Chemistry for Life Science, School of Chemistry and Chemical Engineering, Nanjing University, Nanjing 210093, PR China.

Li, Jian-Xin. Key Lab of Analytical Chemistry for Life Science, School of Chemistry and Chemical Engineering, Nanjing University, Nanjing 210093, PR China. Electronic address: lijxnju@nju.edu.cn.

Abstract

Rheumatoid arthritis (RA) is a chronic, systemic inflammatory disorder that affects about 1% of the population worldwide. RA is mainly manifested by persistent synovitis and progressive joint destruction. The aim of the present study was to examine the anti-arthritis effects of SND-117, a sinomenine bivalent that is obtained from the structure modification of a clinically available anti-RA drug, sinomenine. The arthritis model (CIA) was established by immunizing DBA/1 mice with type II collagen, and the arthritis scores including inflammation, joint destruction and bone erosion were assessed after booster immunization for 3weeks. The levels of cytokines such as IL-1beta, IL-6 and TNF-alpha were analyzed by quantitative PCR and ELISA. The TNF-alpha induced NF-kappaB activation in fibroblast-like synovial cells (FLSCs) was analyzed by Western blot. SND-117 significantly relieved the inflammatory symptoms of collagen-induced arthritis, reduced bone erosion and joint destruction in CIA mice. The serum levels of IL-1beta, IL-6 and TNF-alpha of CIA mice were markedly decreased by SND-117. SND-117 also strongly inhibited the phosphorylation and nuclear translocation of NF-kappaB p65 in FLSCs upon TNF-alpha stimulation. These data demonstrated that SND-117 could effectively block the pathogenesis of collagen-induced arthritis in CIA mice via inhibition of NF-kappaB signaling, and might provide potential clinic benefits in rheumatoid arthritis management. Copyright © 2015 Elsevier B.V. All rights reserved.

Publication Type

Journal Article. Research Support, Non-U.S. Gov't.

Year of Publication

2015

<320>

Unique Identifier

25886694

Title

Extracorporeal photopheresis for the treatment of autoimmune diseases. [Review]

Source

Transfusion & Apheresis Science. 52(2):171-82, 2015 Apr.

VI 1

Status

MEDLINE

Authors

Adamski J; Kinard T; Ipe T; Cooling L.

Authors Full Name

Adamski, Jill; Kinard, Theresa; Ipe, Tina; Cooling, Laura.

Institution

Adamski, Jill. Department of Laboratory Medicine and Pathology, Mayo Clinic Arizona, Phoenix, AZ, USA. Electronic address: Adamski.Jill@mayo.edu.

Kinard, Theresa. Department of Laboratory Medicine and Pathology, Mayo Clinic Arizona, Phoenix, AZ, USA.

Ipe, Tina. Department of Pathology and Genomic Medicine, Houston Methodist Hospital, Houston, TX, USA.

Cooling, Laura. Department of Pathology, University of Michigan, Ann Arbor, MI, USA.

Abstract

The immune system is tasked with the unique challenge of recognizing foreign pathogens and damaged cells while at the same time preserving and protecting the integrity of "self". When this process fails, severe consequences including cancer and autoimmunity are the end result. Current therapies aimed at treating autoimmune disorders result in generalized immunosuppression and place the patient at increased risk for infection and malignancy. ECP is a potential therapeutic intervention that recapitulates natural physiologic processes of tolerance induction to restore immune homeostasis. Several clinical trials suggest that ECP may be used to treat a broad spectrum of autoimmune diseases. Copyright © 2015 Elsevier Ltd. All rights reserved.

Publication Type
  Journal Article. Review.
Year of Publication
  2015

<321>
Unique Identifier
  25879435
Title
  CXCL13 antibody for the treatment of autoimmune disorders.
Source
  BMC Immunology. 16:6, 2015 Feb 12.
VI 1
Status
  MEDLINE
Authors
  Klimatcheva E; Pandina T; Reilly C; Torno S; Bussler H; Scrivens M; Jonason A; Mallow C; Doherty M; Paris M; Smith ES; Zauderer M.
Authors Full Name
  Klimatcheva, Ekaterina; Pandina, Tracy; Reilly, Christine; Torno, Sebold; Bussler, Holm; Scrivens, Maria; Jonason, Alan; Mallow, Crystal; Doherty, Michael; Paris, Mark; Smith, Ernest S; Zauderer, Maurice.
Institution
  Klimatcheva, Ekaterina. Vaccinex, Inc, 1895 Mt. Hope Avenue, Rochester, NY, 14620, USA. kklimatcheva@vaccinex.com.
   Pandina, Tracy. Vaccinex, Inc, 1895 Mt. Hope Avenue, Rochester, NY, 14620, USA. tpandina@vaccinex.com.
   Reilly, Christine. Vaccinex, Inc, 1895 Mt. Hope Avenue, Rochester, NY, 14620, USA. creilly@vaccinex.com.
   Torno, Sebold. Vaccinex, Inc, 1895 Mt. Hope Avenue, Rochester, NY, 14620, USA. storno@vaccinex.com.
   Bussler, Holm. Vaccinex, Inc, 1895 Mt. Hope Avenue, Rochester, NY, 14620, USA. hbussler@vaccinex.com.

Scrivens, Maria. Vaccinex, Inc, 1895 Mt. Hope Avenue, Rochester, NY, 14620, USA. mscrivens@vaccinex.com.

Jonason, Alan. Vaccinex, Inc, 1895 Mt. Hope Avenue, Rochester, NY, 14620, USA. ajonason@vaccinex.com.

Mallow, Crystal. Vaccinex, Inc, 1895 Mt. Hope Avenue, Rochester, NY, 14620, USA. cmallow@vaccinex.com.

Doherty, Michael. Vaccinex, Inc, 1895 Mt. Hope Avenue, Rochester, NY, 14620, USA. mdoherty@vaccinex.com.

Paris, Mark. Vaccinex, Inc, 1895 Mt. Hope Avenue, Rochester, NY, 14620, USA. mparis@vaccinex.com.

Smith, Ernest S. Vaccinex, Inc, 1895 Mt. Hope Avenue, Rochester, NY, 14620, USA. esmith@vaccinex.com.

Zauderer, Maurice. Vaccinex, Inc, 1895 Mt. Hope Avenue, Rochester, NY, 14620, USA. mzauderer@vaccinex.com.

Abstract

BACKGROUND: Homeostatic B Cell-Attracting chemokine 1 (BCA-1) otherwise known as CXCL13 is constitutively expressed in secondary lymphoid organs by follicular dendritic cells (FDC) and macrophages. It is the only known ligand for the CXCR5 receptor, which is expressed on mature B cells, follicular helper T cells (Tfh), Th17 cells and regulatory T (Treg) cells. Aberrant expression of CXCL13 within ectopic germinal centers has been linked to the development of autoimmune disorders (e.g. Rheumatoid Arthritis, Multiple Sclerosis, Systemic Lupus Erythematosis). We, therefore, hypothesized that antibody-mediated disruption of the CXCL13 signaling pathway would interfere with the formation of ectopic lymphoid follicles in the target organs and inhibit autoimmune disease progression. This work describes pre-clinical development of human anti-CXCL13 antibody MAb 5261 and includes therapeutic efficacy data of its mouse counterpart in murine models of autoimmunity.

RESULTS: We developed a human IgG1 monoclonal antibody, MAb 5261 that specifically binds to human, rodent and primate CXCL13 with an affinity of approximately 5 nM and is capable of neutralizing the activity of CXCL13 from these various species in in vitro functional assays. For in vivo studies we have engineered a chimeric antibody to contain the same human heavy and light chain variable genes along with mouse constant regions. Treatment with this antibody led to a reduction in the number of germinal centers in mice immunized with 4-Hydroxy-3-nitrophenylacetyl hapten conjugated to Keyhole Limpet Hemocyanin (NP-KLH) and, in adoptive transfer studies, interfered with the trafficking of B cells to the B cell areas of mouse spleen. Furthermore, this mouse anti-CXCL13 antibody demonstrated efficacy in a mouse model of

Rheumatoid arthritis (Collagen-Induced Arthritis (CIA)) and Th17-mediated murine model of Multiple Sclerosis (passively-induced Experimental Autoimmune Encephalomyelitis (EAE)).

CONCLUSIONS: We developed a novel therapeutic antibody targeting CXCL13-mediated signaling pathway for the treatment of autoimmune disorders.
Publication Type
Journal Article.
Year of Publication
2015

<322>
Unique Identifier
25875802
Title
A literature review on the patients with autoimmune diseases following vaccination against infections. [Review]
Source
Human vaccines & Immunotherapeutics. 11(9):2274-80, 2015.
VI 1
Status
MEDLINE
Authors
Liang Y; Meng FY; Pan HF; Ye DQ.
Authors Full Name
Liang, Yan; Meng, Fan-Ya; Pan, Hai-Feng; Ye, Dong-Qing.
Institution
Liang, Yan. a Department of Epidemiology and Biostatistics ; School of Public Health; Anhui Medical University ; Hefei, China.
Abstract
Due to immune abnormalities and the use of steroids and immunosuppressant treatment, patients with rheumatic diseases are susceptible to infections. Vaccination is one of the most important prevention tools in modern medicine. A discussion on risk-benefit or cost-benefit analysis, and advisory on individual vaccines or vaccination programs falls outside the scope of

this review. In particularly, this review summarizes the knowledge about the effectiveness and safety vaccinations in patients with autoimmune inflammatory rheumatic diseases (AIIRD) treated with biologics. Finally, we aim to provide vaccination plans basis for clinical management of rheumatic patients depending upon prevaccination antibody titers, drug treatments and immunological potential.

Publication Type

Journal Article. Review.

Year of Publication

2015

<323>

Unique Identifier

25819273

Title

Cell source-dependent in vivo immunosuppressive properties of mesenchymal stem cells derived from the bone marrow and synovial fluid of minipigs.

Source

Experimental Cell Research. 333(2):273-88, 2015 May 01.

VI 1

Status

MEDLINE

Authors

Lee WJ; Hah YS; Ock SA; Lee JH; Jeon RH; Park JS; Lee SI; Rho NY; Rho GJ; Lee SL.

Authors Full Name

Lee, Won-Jae; Hah, Young-Sool; Ock, Sun-A; Lee, Jae-Hoon; Jeon, Ryong-Hoon; Park, Ji-Sung; Lee, Sang-Il; Rho, Na-Young; Rho, Gyu-Jin; Lee, Sung-Lim.

Institution

Lee, Won-Jae. College of Veterinary Medicine, Gyeongsang National University, Jinju 660-701, Gyeongnam, Republic of Korea.

Hah, Young-Sool. Biomedical Research Institute, Gyeongsang National University Hospital, Jinju, Republic of Korea.

Ock, Sun-A. Animal Biotechnology Division, National Institute of Animal Science, RDA, Suwon 441-706, Gyeonggi, Republic of Korea.

Lee, Jae-Hoon. College of Veterinary Medicine, Gyeongsang National University, Jinju 660-701, Gyeongnam, Republic of Korea.
Jeon, Ryong-Hoon. College of Veterinary Medicine, Gyeongsang National University, Jinju 660-701, Gyeongnam, Republic of Korea.
Park, Ji-Sung. College of Veterinary Medicine, Gyeongsang National University, Jinju 660-701, Gyeongnam, Republic of Korea.
Lee, Sang-Il. Department of Internal Medicine and Institute of Health Sciences, Gyeongsang National University, Jinju, Republic of Korea.
Rho, Na-Young. Department of Biomedical Sciences, Ontario Veterinary College, University of Guelph, Guelph, ON, Canada N1G 4S7.
Rho, Gyu-Jin. College of Veterinary Medicine, Gyeongsang National University, Jinju 660-701, Gyeongnam, Republic of Korea; Research Institute of Life Sciences, Gyeongsang National University, Jinju 660-701, Gyeongnam, Republic of Korea.
Lee, Sung-Lim. College of Veterinary Medicine, Gyeongsang National University, Jinju 660-701, Gyeongnam, Republic of Korea; Research Institute of Life Sciences, Gyeongsang National University, Jinju 660-701, Gyeongnam, Republic of Korea. Electronic address: sllee@gnu.ac.kr.
Abstract
The in vitro differentiation and immunosuppressive capacity of mesenchymal stem cells (MSCs) derived from synovial fluid (SF-MSCs) and bone marrow extract (BM-MSCs) in an isogenic background of minipigs were comparatively analyzed in a collagen-induced arthritis (CIA) mouse model of rheumatoid arthritis (RA). The proliferation capacity and expression of pluripotent transcription factors (Oct3/4 and Sox2) were significantly ($P<0.05$) higher in SF-MSCs than in BM-MSCs. The differentiation capacity of SF-MSCs into adipocytes, osteocytes and neurocytes was significantly ($P<0.05$) lower than that of BM-MSCs, and the differentiation capacity of SF-MSCs into chondrocytes was significantly ($P<0.05$) higher than that of BM-MSCs. Systemic injection of BM- and SF-MSCs significantly ($P<0.05$) ameliorated the clinical symptoms of CIA mice, with SF-MSCs having significantly ($P<0.05$) higher clinical and histopathological recovery scores than BM-MSCs. Furthermore, the immunosuppressive properties of SF-MSCs in CIA mice were associated with increased levels of the anti-inflammatory cytokine interleukin (IL)-10, and decreased levels of the pro-inflammatory cytokine IL-1beta and osteoclast-related sRANKL. In conclusion, SF-MSCs exhibited eminent pluripotency and differentiation capacity into chondrocytes, addition to substantial in vivo immunosuppressive capacity by elevating IL-10 and reducing IL-1beta levels in CIA mice. Copyright © 2015 The Authors. Published by Elsevier Inc. All rights reserved.
Publication Type
Journal Article. Research Support, Non-U.S. Gov't.
Year of Publication

2015

<324>

Unique Identifier

25787143

Title

Rheumatoid arthritis vaccine therapies: perspectives and lessons from therapeutic ligand epitope antigen presentation system vaccines for models of rheumatoid arthritis. [Review]

Source

Expert Review of Vaccines. 14(6):891-908, 2015 Jun.

VI 1

Status

MEDLINE

Authors

Rosenthal KS; Mikecz K; Steiner HL 3rd; Glant TT; Finnegan A; Carambula RE; Zimmerman DH.

Authors Full Name

Rosenthal, Kenneth S; Mikecz, Katalin; Steiner, Harold L 3rd; Glant, Tibor T; Finnegan, Alison; Carambula, Roy E; Zimmerman, Daniel H.

Institution

Rosenthal, Kenneth S. Roseman University of Health Sciences, Las Vegas, NV, USA.

Abstract

The current status of therapeutic vaccines for autoimmune diseases is reviewed with rheumatoid arthritis as the focus. Therapeutic vaccines for autoimmune diseases must regulate or subdue responses to common self-antigens. Ideally, such a vaccine would initiate an antigen-specific modulation of the T-cell immune response that drives the inflammatory disease. Appropriate animal models and types of T helper cells and signature cytokine responses that drive autoimmune disease are also discussed. Interpretation of these animal models must be done cautiously because the means of initiation, autoantigens, and even the signature cytokine and T helper cell (Th1 or Th17) responses that are involved in the disease may differ significantly from those in humans. We describe ligand epitope antigen presentation system vaccine modulation of T-cell autoimmune responses as a strategy for the design of therapeutic vaccines for rheumatoid arthritis, which may also be effective in other autoimmune conditions.

Publication Type

Journal Article. Research Support, N.I.H., Extramural. Review.

Year of Publication

2015

<325>

Unique Identifier

25758914

Title

Desensitization to febuxostat: report of two cases.

Source

The Journal of Allergy & Clinical Immunology in Practice. 3(4):633-6, 2015 Jul-Aug.

VI 1

Status

MEDLINE

Authors

Di Paolo C; Minetti S; Mineni M; Inverardi S; Rizzini FL; Cinquini M; Tosoni C.

Authors Full Name

Di Paolo, Camilla; Minetti, Stefano; Mineni, Michela; Inverardi, Silvia; Rizzini, Fabio Lodi; Cinquini, Massimo; Tosoni, Cinzia.

Institution

Di Paolo, Camilla. Struttura Semplice Valenza Dipartimentale Allergologia, Spedali Civili di Brescia, Brescia, Italy.

Minetti, Stefano. Struttura Semplice Valenza Dipartimentale Allergologia, Universita del studi di Brescia, Brescia, Italy. Electronic address: Steminetti@libero.it.

Mineni, Michela. Struttura Semplice Valenza Dipartimentale Allergologia, Universita del studi di Brescia, Brescia, Italy.

Inverardi, Silvia. Struttura Semplice Valenza Dipartimentale Allergologia, Universita del studi di Brescia, Brescia, Italy.

Rizzini, Fabio Lodi. Struttura Semplice Valenza Dipartimentale Allergologia, Universita del studi di Brescia, Brescia, Italy.

Cinquini, Massimo. Struttura Semplice Valenza Dipartimentale Allergologia, Spedali Civili di Brescia, Brescia, Italy.

Tosoni, Cinzia. Struttura Semplice Valenza Dipartimentale Allergologia, Spedali Civili di Brescia, Brescia, Italy.
Publication Type
Case Reports. Letter.
Year of Publication
2015

<326>
Unique Identifier
25731770
Title
Immunoregulatory role of IL-35 in T cells of patients with rheumatoid arthritis.
Source
Rheumatology. 54(8):1498-506, 2015 Aug.
VI 1
Status
MEDLINE
Authors
Nakano S; Morimoto S; Suzuki S; Tsushima H; Yamanaka K; Sekigawa I; Takasaki Y.
Authors Full Name
Nakano, Souichiro; Morimoto, Shinji; Suzuki, Satoshi; Tsushima, Hiroshi; Yamanaka, Kenjiro; Sekigawa, Iwao; Takasaki, Yoshinari.
Institution
Nakano, Souichiro. Department of Rheumatology and Internal Medicine, Juntendo University School of Medicine, Tokyo, soubey@juntendo.ac.jp.
Morimoto, Shinji. Institute for Environment and Gender Specific Medicine, Juntendo University Graduate School of Medicine, Chiba and.
Suzuki, Satoshi. Department of Rheumatology and Internal Medicine, Juntendo University School of Medicine, Tokyo.
Tsushima, Hiroshi. Department of Rheumatology and Internal Medicine, Juntendo University School of Medicine, Tokyo.
Yamanaka, Kenjiro. Department of Rheumatology and Internal Medicine, Sasaki Foundation, Tokyo, Japan.

Sekigawa, Iwao. Institute for Environment and Gender Specific Medicine, Juntendo University Graduate School of Medicine, Chiba and.
Takasaki, Yoshinari. Department of Rheumatology and Internal Medicine, Juntendo University School of Medicine, Tokyo.
Abstract
OBJECTIVE: IL-35 is the most recently identified member of the IL-12 family. It consists of EBV-induced gene 3 (EBI3) and IL-12alpha chain p35. We investigated whether IL-35 enhances the in vitro immunosuppressive function of peripheral blood isolated from patients with RA.

METHODS: Peripheral blood was harvested from 17 active and 10 inactive RA patients and IL-35 concentrations were quantified using an ELISA. An expression vector containing IL-35 with a FLAG tag at the carboxyl-terminus was constructed by covalently linking EBI3 and IL-12alpha (p35). The function of IL-35 was then evaluated in a suppression assay using T cells isolated from human RA patients with CD2, CD3 and CD28 antibodies.

RESULTS: Serum IL-35 levels and the number of Treg were decreased significantly in patients with active RA. There was a significant correlation between serum IL-35 and the 28-joint DAS with ESR (DAS28-ESR) in patients with active RA. IL-35 treatment enhanced the regulatory function, suppressing the levels of inflammatory cytokines such as IL-17 and IFN-gamma and the cellular growth of effector T cells stimulated by conjugation with CD2, CD3 and CD28.

CONCLUSION: These data revealed that IL-35 might suppress T cell activation during the peripheral immune responses of RA. Therefore our data suggest that IL-35 might have multiple therapeutic targets. Copyright © The Author 2015. Published by Oxford University Press on behalf of the British Society for Rheumatology. All rights reserved. For Permissions, please email: journals.permissions@oup.com.
Publication Type
Journal Article. Research Support, Non-U.S. Gov't.
Year of Publication
2015

<327>
Unique Identifier

25727209

Title

Pathogenic functions of B cells in autoimmune diseases: IFN-gamma production joins the criminal gang.

Source

European Journal of Immunology. 45(4):966-70, 2015 Apr.

VI 1

Status

MEDLINE

Authors

Fillatreau S.

Authors Full Name

Fillatreau, Simon.

Institution

Fillatreau, Simon. Deutsches Rheuma-Forschungszentrum, a Leibniz Institute, Berlin, Germany.

Abstract

B-cell depletion therapy has emerged as a powerful strategy to intercept the progression of T-cell-mediated autoimmune diseases such as rheumatoid arthritis, type 1 diabetes, or relapsing remitting multiple sclerosis. However, its mode of action remains incompletely defined, reflecting our incomplete understanding of the pathogenic functions of B cells in such pathologies. B cells can contribute to immune responses through the production of antibodies, presentation of antigen to T cells, and production of cytokines. In this issue of the European Journal of Immunology [Eur. J. Immunol. 2015. 45: 988-998], Olalekan et al. demonstrate that IFN-gamma production by B cells is essential for the development of arthritis in mice. Lack of IFN-gamma expression in B cells results in reduced autoimmune T-cell responses and autoantibody levels, impacting the arthritogenic reaction akin to that in B-cell depletion therapy. Together with other reports, the article by Olalekan et al. emphasizes the importance of cytokine-producing B cells in the pathogenesis of autoimmune diseases. In this commentary, I discuss how these findings shed new light on the roles of B cells as drivers of autoimmune pathogenesis, and how they more generally contribute to our understanding of the role of B cells in immunity. Copyright © 2015 WILEY-VCH Verlag GmbH & Co. KGaA, Weinheim.

Publication Type

Journal Article. Research Support, Non-U.S. Gov't.

Year of Publication

2015

<328>

Unique Identifier

25690878

Title

Risk of zoster in patients on immunosuppressant therapy: evaluation of current data.

Source

American Journal of Infection Control. 43(4):420-1, 2015 Apr 01.

VI 1

Status

MEDLINE

Authors

Tafuri SS; Chironna MM; Quarto MM; Germinario CC.

Authors Full Name

Tafuri, Silvio S T; Chironna, Maria M C; Quarto, Michele M Q; Germinario, Cinzia C G.

Institution

Tafuri, Silvio S T. Department of Biomedical Science and Human Oncology, Aldo Moro University of Bari, Bari, Italy. Electronic address: silvio.tafuri@uniba.it.

Chironna, Maria M C. Department of Biomedical Science and Human Oncology, Aldo Moro University of Bari, Bari, Italy.

Quarto, Michele M Q. Department of Biomedical Science and Human Oncology, Aldo Moro University of Bari, Bari, Italy.

Germinario, Cinzia C G. Department of Biomedical Science and Human Oncology, Aldo Moro University of Bari, Bari, Italy.

Publication Type

Letter.

Year of Publication

2015

<329>

Unique Identifier

25680967

Title

Myeloid-derived suppressor cells are proinflammatory and regulate collagen-induced arthritis through manipulating Th17 cell differentiation.

Source

Clinical Immunology. 157(2):175-86, 2015 Apr.

VI 1

Status

MEDLINE

Authors

Zhang H; Wang S; Huang Y; Wang H; Zhao J; Gaskin F; Yang N; Fu SM.

Authors Full Name

Zhang, Hui; Wang, Shuang; Huang, Yuefang; Wang, Hongyue; Zhao, Jijun; Gaskin, Felicia; Yang, Niansheng; Fu, Shu Man.

Institution

Zhang, Hui. Department of Rheumatology, First Affiliated Hospital, Sun Yat-sen University, 58 Zhongshan Road II, Guangzhou 510080, China.

Wang, Shuang. Department of Rheumatology, First Affiliated Hospital, Sun Yat-sen University, 58 Zhongshan Road II, Guangzhou 510080, China.

Huang, Yuefang. Department of Pediatrics, First Affiliated Hospital, Sun Yat-sen University, 58 Zhongshan Road II, Guangzhou 510080, PR China.

Wang, Hongyue. Department of Rheumatology, First Affiliated Hospital, Sun Yat-sen University, 58 Zhongshan Road II, Guangzhou 510080, China.

Zhao, Jijun. Department of Rheumatology, First Affiliated Hospital, Sun Yat-sen University, 58 Zhongshan Road II, Guangzhou 510080, China.

Gaskin, Felicia. Department of Psychiatry and Neurobehavioral Sciences, University of Virginia, Charlottesville, VA 22908-0133, USA.

Yang, Niansheng. Department of Rheumatology, First Affiliated Hospital, Sun Yat-sen University, 58 Zhongshan Road II, Guangzhou 510080, China. Electronic address: zsuyns@163.com.

Fu, Shu Man. Division of Rheumatology and Center of inflammation, Immunology and Regenerative Medicine, Department of Medicine and Department of Microbiology, Immunology and Cancer Biology, University of Virginia, Charlottesville, VA 22908-0133, USA.

Abstract

Myeloid-derived suppressor cells (MDSC) and Th17 cells were found to expand in collagen-induced arthritis (CIA) significantly. Two subsets of MDSC, polymorphonuclear (PMN) and mononuclear (MO), were detected and their ratios varied during the development of CIA. The

depletion of MDSC in vivo resulted in suppression of T-cell proliferation and decreased IL-17A and IL-1beta production. The adoptive transfer of MDSC restored the severity of arthritis and Th17 cell differentiation. The depletion of MDSCs on day 35 resulted in arthritis amelioration without reaching a significant difference. Furthermore, MDSCs from CIA mice had higher production of IL-1beta and promoted Th17 cell differentiation. The expansion of MDSCs in the peripheral blood of rheumatoid arthritis (RA) patients was in correlation with increased Th17 cells and disease activity DAS28. These results support the hypothesis that MDSC may play a significant proinflammatory role in the pathogenesis of CIA and RA by inducing Th17 development in an IL-1beta-dependent manner. Copyright © 2015 Elsevier Inc. All rights reserved.

Publication Type

Journal Article. Research Support, N.I.H., Extramural. Research Support, Non-U.S. Gov't.

Year of Publication

2015

<330>

Unique Identifier

25659279

Title

Low rate of influenza and pneumococcal vaccine coverage in rheumatoid arthritis: data from the international COMORA cohort.

Source

Vaccine. 33(12):1446-52, 2015 Mar 17.

VI 1

Status

MEDLINE

Authors

Hmamouchi I; Winthrop K; Launay O; Dougados M.

Authors Full Name

Hmamouchi, Ihsane; Winthrop, Kevin; Launay, Odile; Dougados, Maxime.

Institution

Hmamouchi, Ihsane. Paris Descartes University, APHP Cochin Hospital, Rheumatology Department, Paris, France; Mohammed V University, Faculty of Medicine, Laboratory of

Biostatistics, Clinical Research and Epidemiology, El Ayachi Hospital, Rheumatology Department, Rabat, Morocco. Electronic address: ihsane.hmamouchi@gmail.com.
Winthrop, Kevin. Division of Infectious Diseases, Public Health and Preventive Medicine, Oregon Health and Science University, Portland, OR, USA. Electronic address: winthrop@ohsu.edu.
Launay, Odile. Paris Descartes University, AP-HP Cochin Hospital, CIC Cochin Pasteur, Inserm, CIC 1417, Paris, France. Electronic address: odile.launay@cch.aphp.fr.
Dougados, Maxime. Paris Descartes University, APHP Cochin Hospital, Rheumatology Department, Paris, France. Electronic address: maxime.dougados@cch.aphp.fr.
Abstract
BACKGROUND: Rheumatoid Arthritis (RA) patients are at increased risk of suffering from respiratory infections than the general public. Vaccinations against Streptococcus pneumococcus and influenza are recommended, but not often used in RA. Our objectives were: (1) to describe pneumococcal and influenza vaccine coverage in RA patients across various countries and (2) to identify factors associated with their usage.

METHODS: Using data from the COMORA cohort, 3920 RA patients were enrolled across 17 countries. We collected patient demographic and disease characteristics, and reported vaccine use over a six month time period. We used logistic regression to evaluate factors related to pneumococcal and influenza vaccine coverage.

RESULTS: Overall vaccination coverage within the recommendations was low with huge disparities between countries: 17.2% (95%CI: 16.0-18.4) for pneumococcal vaccination (from 0% in Morocco to 56.5% in France) and 25.3% (95%CI: 23.8-26.5) for influenza vaccination (less than 1% in Morocco and Egypt to 66.2% in Japan). In countries where immunization was more frequent, we found that predictive factors of vaccination were older age, lower disease activity, higher educational level, use of biotherapy, absence of corticosteroid therapy, and presence of comorbidities.

CONCLUSION: Despite international recommendations for influenza and pneumococcal vaccination, we observed a low prevalence of these vaccinations among RA patients, with huge disparity between countries. Efforts are needed to better inform patients and physicians regarding the need for vaccinations. Copyright © 2015 Elsevier Ltd. All rights reserved.
Publication Type
Journal Article. Multicenter Study. Observational Study. Research Support, Non-U.S. Gov't.
Year of Publication
2015

<331>

Unique Identifier

25645456

Title

B cells expressing IFN-gamma suppress Treg-cell differentiation and promote autoimmune experimental arthritis.

Source

European Journal of Immunology. 45(4):988-98, 2015 Apr.

VI 1

Status

MEDLINE

Authors

Olalekan SA; Cao Y; Hamel KM; Finnegan A.

Authors Full Name

Olalekan, Susan A; Cao, Yanxia; Hamel, Keith M; Finnegan, Alison.

Institution

Olalekan, Susan A. Department of Immunology/Microbiology, Rush University Medical Center, Cohn Research Building, Chicago, IL, USA.

Abstract

Clinical efficacy in the treatment of rheumatoid arthritis with anti-CD20 (Rituximab)-mediated B-cell depletion has garnered interest in the mechanisms by which B cells contribute to autoimmunity. We have reported that B-cell depletion in a murine model of proteoglycan-induced arthritis (PGIA) leads to an increase in Treg cells that correlate with decreased autoreactivity. Here, we demonstrate that the increase in Treg cells after B-cell depletion is due to an increase in the differentiation of naive CD4(+) T cells into Treg cells. Since the development of PGIA is dependent on IFN-gamma and B cells are reported to produce IFN-gamma, we hypothesized that B-cell-specific IFN-gamma plays a role in the development of PGIA. Accordingly, mice with B-cell-specific IFN-gamma deficiency were as resistant to the induction of PGIA as mice that were completely IFN-gamma deficient. Importantly, despite a normal frequency of IFN-gamma-producing CD4(+) T cells, B-cell-specific IFN-gamma-deficient mice exhibited a higher percentage of Treg cells compared with that in WT mice. These data indicate that B-cell IFN-gamma production inhibits Treg-cell differentiation and exacerbates arthritis. Thus, we have

established that IFN-gamma, specifically derived from B cells, uniquely contributes to the pathogenesis of autoimmunity through prevention of immunoregulatory mechanisms. Copyright © 2015 WILEY-VCH Verlag GmbH & Co. KGaA, Weinheim.

Publication Type

Journal Article. Research Support, N.I.H., Extramural. Research Support, Non-U.S. Gov't.

Year of Publication

2015

<332>

Unique Identifier

25608826

Title

Thrombocytopenia due to low-dose colchicine therapy: A possible drug interaction with nivolumab and implications for supportive care.

Source

Acta Oncologica. 54(8):1235-7, 2015.

VI 1

Status

MEDLINE

Authors

Solomon LR.

Authors Full Name

Solomon, Lawrence R.

Institution

Solomon, Lawrence R. a Section of Palliative Care, Department of Medicine, Yale University School of Medicine and Smilow Cancer Hospital, New Haven, CT, USA.

Publication Type

Case Reports. Letter.

Year of Publication

2015

<333>

Unique Identifier

25601498

Title

Fibroblast growth factor 21 (FGF21) ameliorates collagen-induced arthritis through modulating oxidative stress and suppressing nuclear factor-kappa B pathway.

Source

International Immunopharmacology. 25(1):74-82, 2015 Mar.

VI 1

Status

MEDLINE

Authors

Yu Y; Li S; Liu Y; Tian G; Yuan Q; Bai F; Wang W; Zhang Z; Ren G; Zhang Y; Li D.

Authors Full Name

Yu, Yinhang; Li, Siming; Liu, Yaonan; Tian, Guiyou; Yuan, Qingyan; Bai, Fuliang; Wang, Wenfei; Zhang, Zhiyi; Ren, Guiping; Zhang, Yu; Li, Deshan.

Institution

Yu, Yinhang. Bio-pharmaceutical Lab, Life Science College, Northeast Agricultural University, Harbin 150030, China.

Li, Siming. Bio-pharmaceutical Lab, Life Science College, Northeast Agricultural University, Harbin 150030, China; Harbin University of Commerce, Harbin 150028, China.

Liu, Yaonan. Bio-pharmaceutical Lab, Life Science College, Northeast Agricultural University, Harbin 150030, China.

Tian, Guiyou. Bio-pharmaceutical Lab, Life Science College, Northeast Agricultural University, Harbin 150030, China.

Yuan, Qingyan. Bio-pharmaceutical Lab, Life Science College, Northeast Agricultural University, Harbin 150030, China.

Bai, Fuliang. Bio-pharmaceutical Lab, Life Science College, Northeast Agricultural University, Harbin 150030, China.

Wang, Wenfei. Bio-pharmaceutical Lab, Life Science College, Northeast Agricultural University, Harbin 150030, China; Key Laboratory of Agricultural Biological Functional Gene, Harbin 150030, China.

Zhang, Zhiyi. Harbin Medical University, The First Affiliated Hospital, Nangang District, 150001 Harbin, China.

Ren, Guiping. Bio-pharmaceutical Lab, Life Science College, Northeast Agricultural University, Harbin 150030, China; Key Laboratory of Agricultural Biological Functional Gene, Harbin 150030, China. Electronic address: renguiping@126.com.
Zhang, Yu. Division of Avian Infectious Diseases, State Key Laboratory of Veterinary Biotechnology, Harbin Veterinary Research Institute, The Chinese Academy of Agricultural Sciences, Harbin 150030, China.
Li, Deshan. Bio-pharmaceutical Lab, Life Science College, Northeast Agricultural University, Harbin 150030, China; Key Laboratory of Agricultural Biological Functional Gene, Harbin 150030, China. Electronic address: deshanli@163.com.
Abstract
It has been demonstrated that circulating FGF21 levels are elevated in the serum and synovial fluid of patients with rheumatoid arthritis (RA). The aim of this study is to investigate efficacy of FGF21 for treatment of RA and the molecular mechanisms of the therapeutic effect on collagen-induced arthritis (CIA). Mice with CIA were subcutaneously administered with FGF21 (5, 2 or 1mg.kg(-1). d(-1)), IL-1beta antibody (5mg.kg(-1). d(-1)), IL-17A antibody (5mg.kg(-1). d(-1)) and dexamethasone (DEX) (1mg.kg(-1). d(-1)), respectively. The effects of treatment were determined by arthritis severity score, histological damage and cytokine production. The activation of NF-kappaB was analyzed by Western blotting. We also detected the levels of oxidative stress parameters. Our results showed that FGF21 had beneficial effects on clinical symptom and histological lesion of CIA mice. Similar to antibody and DEX, FGF21 treatment alleviated the severity of arthritis by reducing humoral and cellular immune responses and down-regulating the expression of pro-inflammatory cytokines. FGF21 treatment also reduced the expression of TNF-alpha, IL-1beta, IL-6, IFN-gamma and MMP-3 and increased level of IL-10 in the spleen tissue or the plasma of CIA mice in a dose-dependent manner. Furthermore, FGF21 inhibited IkappaBalpha degradation and NF-kappaB p65 nuclear translocation and induced significant changes of oxidative stress parameters (MDA, SOD, CAT, GSH-PX and GSH) in the plasma. FGF21 exerts therapeutic efficacy for RA through antioxidant reaction and inhibiting NF-kappaB inflammatory pathway. This study provides evidence that FGF21 may be a promising therapeutic agent for RA patients. Copyright © 2015 Elsevier B.V. All rights reserved.
Publication Type
Comparative Study. Journal Article. Research Support, Non-U.S. Gov't.
Year of Publication
2015

<334>

Unique Identifier

25581706

Title

Reversible lacrimal gland-protective regulatory T-cell dysfunction underlies male-specific autoimmune dacryoadenitis in the non-obese diabetic mouse model of Sjogren syndrome.

Source

Immunology. 145(2):232-41, 2015 Jun.

VI 1

Status

MEDLINE

Authors

Lieberman SM; Kreiger PA; Koretzky GA.

Authors Full Name

Lieberman, Scott M; Kreiger, Portia A; Koretzky, Gary A.

Institution

Lieberman, Scott M. Stead Family Department of Pediatrics, Carver College of Medicine, University of Iowa, Iowa City, IA, USA.

Abstract

CD4(+) CD25(+) Foxp3(+) regulatory T (Treg) cells are required to maintain immunological tolerance; however, defects in specific organ-protective Treg cell functions have not been demonstrated in organ-specific autoimmunity. Non-obese diabetic (NOD) mice spontaneously develop lacrimal and salivary gland autoimmunity and are a well-characterized model of Sjogren syndrome. Lacrimal gland disease in NOD mice is male-specific, but the role of Treg cells in this sex-specificity is not known. This study aimed to determine if male-specific autoimmune dacryoadenitis in the NOD mouse model of Sjogren syndrome is the result of lacrimal gland-protective Treg cell dysfunction. An adoptive transfer model of Sjogren syndrome was developed by transferring cells from the lacrimal gland-draining cervical lymph nodes of NOD mice to lymphocyte-deficient NOD-SCID mice. Transfer of bulk cervical lymph node cells modelled the male-specific dacryoadenitis that spontaneously develops in NOD mice. Female to female transfers resulted in dacryoadenitis if the CD4(+) CD25(+) Treg-enriched population was depleted before transfer; however, male to male transfers resulted in comparable dacryoadenitis regardless of the presence or absence of Treg cells within the donor cell population. Hormone manipulation studies suggested that this Treg cell dysfunction was mediated at least in part by androgens. Surprisingly, male Treg cells were capable of preventing the transfer of dacryoadenitis to female recipients. These data suggest that male-specific factors promote

reversible dysfunction of lacrimal gland-protective Treg cells and, to our knowledge, form the first evidence for reversible organ-protective Treg cell dysfunction in organ-specific autoimmunity.
Copyright © 2015 John Wiley & Sons Ltd.
Publication Type
Journal Article. Research Support, N.I.H., Extramural. Research Support, Non-U.S. Gov't.
Year of Publication
2015

<335>
Unique Identifier
25550277
Title
Introduction: Antibody-targeted therapy special issue.
Source
International Immunology. 27(1):1-2, 2015 Jan.
VI 1
Status
MEDLINE
Authors
Kishimoto T.
Authors Full Name
Kishimoto, Tadamitsu.
Institution
Kishimoto, Tadamitsu. Editor-in-Chief.
Publication Type
Editorial. Introductory Journal Article.
Year of Publication
2015

<336>

Unique Identifier

25486980

Title

Vaccination of patients with autoimmune inflammatory rheumatic diseases. [Review]

Source

Nature Reviews Rheumatology. 11(3):135-45, 2015 Mar.

VI 1

Status

MEDLINE

Authors

Westra J; Rondaan C; van Assen S; Bijl M.

Authors Full Name

Westra, Johanna; Rondaan, Christien; van Assen, Sander; Bijl, Marc.

Institution

Westra, Johanna. Department of Rheumatology and Clinical Immunology, University Medical Center Groningen, University of Groningen, PO Box 30.001, 9700 RB Groningen, Netherlands.

Rondaan, Christien. Department of Rheumatology and Clinical Immunology, University Medical Center Groningen, University of Groningen, PO Box 30.001, 9700 RB Groningen, Netherlands.

van Assen, Sander. Department of Internal Medicine, Division of Infectious Diseases, University Medical Center Groningen, University of Groningen, PO Box 30.001, 9700 RB Groningen, Netherlands.

Bijl, Marc. Department of Internal Medicine and Rheumatology, Martini Hospital, Van Swietenplein 1, PO Box 30.033, 9700 RM, Groningen, Netherlands.

Abstract

Patients with autoimmune inflammatory rheumatic diseases (AIRDs) are at increased risk of infections. This risk has been further increased by the introduction of biologic agents over the past two decades. One of the most effective strategies to prevent infection is vaccination. However, patients with an AIRD have a compromised immune system, which is further impaired by medication. Another important issue is the possibility of triggering a broad nonspecific response by vaccination, potentially resulting in increased activity of the underlying autoimmune disease. In this Review, we provide an analysis of data on vaccination of patients with an AIRD. Both the efficacy and the safety of vaccination are addressed, together with the epidemiology of vaccine-preventable infectious diseases in different subgroups of adults with AIRDs. Special attention is given to vaccination of patients who are treated with biologic agents.

Publication Type

Journal Article. Review.

Year of Publication
  2015

<337>
Unique Identifier
  25466295
Title
  The role of the Th17 cytokines IL-17 and IL-22 in Rheumatoid Arthritis pathogenesis and developments in cytokine immunotherapy. [Review]
Source
  Cytokine. 74(1):101-7, 2015 Jul.
VI 1
Status
  MEDLINE
Authors
  Roeleveld DM; Koenders MI.
Authors Full Name
  Roeleveld, Debbie M; Koenders, Marije I.
Institution
  Roeleveld, Debbie M. Radboud University Medical Center, Experimental Rheumatology, Department of Rheumatology, Geert Grooteplein 26-28, PO Box 9101, 6500 HB Nijmegen, The Netherlands. Electronic address: Debbie.Roeleveld@radboudumc.nl.
   Koenders, Marije I. Radboud University Medical Center, Experimental Rheumatology, Department of Rheumatology, Geert Grooteplein 26-28, PO Box 9101, 6500 HB Nijmegen, The Netherlands. Electronic address: Marije.Koenders@radboudumc.nl.
Abstract
  Over the past few years, the importance of Interleukin (IL)-17 and T helper (Th)17 cells in the pathology of Rheumatoid Arthritis (RA) has become apparent. RA is a systemic autoimmune disease that affects up to 1% of the population worldwide. It is characterized by an inflamed, hyperplastic synovium with pannus formation, leading to bone and cartilage destruction in the joints. By the production of effector cytokines like IL-17 and IL-22, the T helper 17 subset protects the host against bacterial and fungal infections, but it can also promote the development of various autoimmune diseases like RA. Hence, the Th17 pathway recently became a very

interesting target in RA treatment. Up to now, several therapies targeting the Th17 cells or its effector cytokines have been tested, or are currently under investigation. This review clarifies the role of Th17 cells and its cytokines in the pathogenesis of RA, and provides an overview of the clinical trials using immunotherapy to target this particular T helper subset or the two main effector cytokines by which the Th17 cells exert their function, IL-17 and IL-22. Copyright © 2014 Elsevier Ltd. All rights reserved.
Publication Type
  Journal Article. Research Support, Non-U.S. Gov't. Review.
Year of Publication
  2015

<338>
Unique Identifier
  25418753
Title
  Seroconversion in patients with rheumatic diseases treated with immunomodulators or immunosuppressants, who were inadvertently revaccinated against yellow fever.
Source
  Arthritis & Rheumatology. 67(2):582-3, 2015 Feb.
VI 1
Status
  MEDLINE
Authors
  Oliveira AC; Mota LM; Santos-Neto LL; Simoes M; Martins-Filho OA; Tauil PL.
Authors Full Name
  Oliveira, Ana C V; Mota, Licia M H; Santos-Neto, Leopoldo L; Simoes, Marisol; Martins-Filho, Olindo A; Tauil, Pedro L.
Institution
  Oliveira, Ana C V. University of Brasilia, Brasilia, Brazil.
Publication Type
  Journal Article. Research Support, Non-U.S. Gov't.
Year of Publication
  2015

<339>

Unique Identifier

25411043

Title

Anti-TNF therapy: past, present and future. [Review]

Source

International Immunology. 27(1):55-62, 2015 Jan.

VI 1

Status

MEDLINE

Authors

Monaco C; Nanchahal J; Taylor P; Feldmann M.

Authors Full Name

Monaco, Claudia; Nanchahal, Jagdeep; Taylor, Peter; Feldmann, Marc.

Institution

Monaco, Claudia. Kennedy Institute of Rheumatology, Nuffield Department of Orthopaedics, Rheumatology and Musculoskeletal Sciences, University of Oxford, Roosevelt Drive, Headington, Oxford OX3 7FY, UK.

Nanchahal, Jagdeep. Kennedy Institute of Rheumatology, Nuffield Department of Orthopaedics, Rheumatology and Musculoskeletal Sciences, University of Oxford, Roosevelt Drive, Headington, Oxford OX3 7FY, UK.

Taylor, Peter. Kennedy Institute of Rheumatology, Nuffield Department of Orthopaedics, Rheumatology and Musculoskeletal Sciences, University of Oxford, Roosevelt Drive, Headington, Oxford OX3 7FY, UK.

Feldmann, Marc. Kennedy Institute of Rheumatology, Nuffield Department of Orthopaedics, Rheumatology and Musculoskeletal Sciences, University of Oxford, Roosevelt Drive, Headington, Oxford OX3 7FY, UK marc.feldmann@kennedy.ox.ac.uk.

Abstract

While for a century therapeutics has been dominated by small molecules, i.e. organic chemicals of ~400Da absorbable via the gut, this is no longer the case. There are now a plethora of important medicines which are proteins and injectable, which have dramatically improved the therapy of many inflammatory diseases and of cancer. Most of these are monoclonal antibodies,

some are receptor Ig Fc fusion proteins, others are cytokines or enzymes. The key to this new aspect of therapeutics has been the filling of unmet needs, and the consequent commercial success, which promoted further research and development. The first 'biologic' for a common disease, rheumatoid arthritis (RA), was a monoclonal antibody, infliximab, to human tumour necrosis factor (TNF). This was based on our work, which is described in this review, summarizing how TNF was defined as a good target in RA, how it was developed is described here, as well as future indications for anti-TNF and related agents. Biologics are now the fastest growing sector of therapeutics. Copyright © The Japanese Society for Immunology. 2014. All rights reserved. For permissions, please e-mail: journals.permissions@oup.com.

Publication Type

Journal Article. Research Support, Non-U.S. Gov't. Review.

Year of Publication

2015

<340>

Unique Identifier

25381726

Title

Pediatric Rheumatology Association of Japan recommendation for vaccination in pediatric rheumatic diseases. [Review]

Source

Modern Rheumatology. 25(3):335-43, 2015 May.

VI 1

Status

MEDLINE

Authors

Kobayashi I; Mori M; Yamaguchi K; Ito S; Iwata N; Masunaga K; Shimojo N; Ariga T; Okada K; Takei S.

Authors Full Name

Kobayashi, Ichiro; Mori, Masaaki; Yamaguchi, Ken-Ichi; Ito, Shuichi; Iwata, Naomi; Masunaga, Kenji; Shimojo, Naoki; Ariga, Tadashi; Okada, Kenji; Takei, Shuji.

Institution

Kobayashi, Ichiro. Department of Pediatrics, Hokkaido University Graduate School of Medicine, Sapporo, Japan.
Abstract
Pediatric Rheumatology Association of Japan has developed evidence-based guideline of vaccination in pediatric rheumatic diseases (PRDs) as a part of Guideline of Vaccination for Pediatric Immunocompromised Hosts. Available articles on vaccination in both adult rheumatic diseases and PRDs were analyzed. Non-live vaccines are generally safe and effective in patients with PRDs on corticosteroid, immunosuppressant, and/or biologics, although efficacy may be attenuated under high dose of the drugs. On the other hand, efficacy and safety of live-attenuated vaccine for the patients on such medication have not been established. Thus, live-attenuated vaccines should be withheld and, if indicated, may be considered as a clinical trial under the approval by Institutional Review Board. All patients with PRDs anticipating treatment with immunosuppressants or biologics should be screened for infection of hepatitis B and C and tuberculosis before the commencement of medication. Varicella vaccine should be considered in sensitive patients ideally 3 weeks or longer before the commencement of immunosuppressants, corticosteroids, or biologics. Bacille Calmette-Guerin should be withheld at least for 6 months after birth, if their mothers have received anti-tumor necrosis factor-alpha antibodies during the second or third trimester of pregnancy.
Publication Type
Journal Article. Practice Guideline. Review.
Year of Publication
2015

<341>
Unique Identifier
25370437
Title
Usefulness of monitoring of B cell depletion in rituximab-treated rheumatoid arthritis patients in order to predict clinical relapse: a prospective observational study.
Source
Clinical & Experimental Immunology. 180(1):11-8, 2015 Apr.
VI 1
Status

MEDLINE

Authors

Trouvin AP; Jacquot S; Grigioni S; Curis E; Dedreux I; Roucheux A; Boulard H; Vittecoq O; Le Loet X; Boyer O; Goeb V.

Authors Full Name

Trouvin, A-P; Jacquot, S; Grigioni, S; Curis, E; Dedreux, I; Roucheux, A; Boulard, H; Vittecoq, O; Le Loet, X; Boyer, O; Goeb, V.

Institution

Trouvin, A-P. Rouen University Hospital, Department of Rheumatology, France.

Abstract

Our objective was to evaluate the contribution of monitoring B cell subset depletion after rituximab in patients with rheumatoid arthritis (RA) in order to guide reintroduction to forestall relapse. This prospective, monocentre study included all RA patients receiving two 1-g rituximab infusions at a 15-day interval. The patients were followed clinically and biologically every 2 months until rituximab reintroduction. The physician was blinded to lymphocyte-typing results to diagnose relapse and, hence, retreatment. Among the 39 patients included between March 2010 and December 2011 and followed until April 2013, seven received two rituximab cycles, yielding a total of 46 cycles for analysis. After the two rituximab cycles, the total number of CD19(+) B cells decreased significantly (0.155 versus 0.0002 G/l, $P < 0.0001$), with complete depletions in all patients of CD19(+) CD38(++) CD24(++) (transitional) ($P < 0.0001$) and CD19(+) CD27(+) (memory) B lymphocytes. A significant majority of patients relapsed within the 4 months following repopulation of total B ($P = 0.036$), B transitional ($P = 0.007$) and B memory ($P = 0.01$) lymphocytes. CD19(+) B lymphocyte repopulation preceded clinical RA relapse and enabled its prediction 4 months in advance. Hence, monitoring of CD19(+) B lymphocytes could serve as a tool to predict those relapses. Copyright © 2014 British Society for Immunology.

Publication Type

Journal Article.

Year of Publication

2015

<342>

Unique Identifier

25370295

Title

Passive transfer of antibodies to the linear epitope 60 kD Ro 273-289 induces features of Sjogren's syndrome in naive mice.

Source

Clinical & Experimental Immunology. 180(1):19-27, 2015 Apr.

VI 1

Status

MEDLINE

Authors

Maier-Moore JS; Kurien BT; D'Souza A; Bockus L; Asfa S; Dorri Y; Hubbell S; Yeliosof O; Obeso D; Schoeb TR; Jonsson R; Scofield RH.

Authors Full Name

Maier-Moore, J S; Kurien, B T; D'Souza, A; Bockus, L; Asfa, S; Dorri, Y; Hubbell, S; Yeliosof, O; Obeso, D; Schoeb, T R; Jonsson, R; Scofield, R H.

Institution

Maier-Moore, J S. The Arthritis and Immunology Program, Oklahoma Medical Research Foundation, Oklahoma City, OK, USA; The Department of Medicine, University of Oklahoma Health Sciences Center, Oklahoma City, OK, USA.

Abstract

Sjogren's syndrome (SS) is an autoimmune inflammatory disease that primarily affects the lacrimal and salivary glands causing dry eyes and mouth. Antibodies to Ro60 are observed frequently in patients with SS; however, the role of these antibodies in SS initiation and progression remains unclear. The sequence Ro60 273-289 (Ro274) is a known B cell epitope of Ro60 and antibodies to this epitope have been observed in a subset of SS patients and in animals immunized with Ro60 protein. Animals immunized with Ro274 linear peptide develop a Sjogren's-like illness. We hypothesized that passive transfer of anti-Ro274-specific immunoglobulin (Ig)G would induce a Sjogren's-like phenotype. To evaluate this hypothesis, we adoptively transferred affinity-purified Ro274 antibodies into naive BALB/c animals, then evaluated salivary gland histology, function and IgG localization 4 days post-transfer. At this time-point, there was no demonstrable mononuclear cell infiltration and salivary glands were histologically normal, but we observed a functional deficit in stimulated salivary flow of animals receiving Ro274 antibodies compared to animals receiving control IgG. Cellular fractionation and enzyme-linked immunosorbent assay revealed Ro274-specific antibodies in the nucleus and cytoplasmic fractions of isolated parotid salivary gland cells that was confirmed by immunohistochemistry. These data support the hypothesis that antibodies to Ro274 deposit in salivary glands can enter intact salivary gland cells and are involved in the dysregulation of salivary flow in SS. Copyright © 2014 British Society for Immunology.

Publication Type
  Journal Article.
Year of Publication
  2015

<343>
Unique Identifier
  25142313
Title
  Therapeutic uses of anti-interleukin-6 receptor antibody. [Review]
Source
  International Immunology. 27(1):21-9, 2015 Jan.
VI 1
Status
  MEDLINE
Authors
  Kang S; Tanaka T; Kishimoto T.
Authors Full Name
  Kang, Sujin; Tanaka, Toshio; Kishimoto, Tadamitsu.
Institution
Kang, Sujin. Department of Clinical Application of Biologics, Osaka University Graduate School of Medicine, 2-2 Yamada-oka, Suita City, Osaka 565-0871, Japan Department of Immunopathology, World Premier International Immunology Frontier Research Center, Osaka University, 3-1 Yamada-oka, Suita City, Osaka 565-0871, Japan.

Tanaka, Toshio. Department of Clinical Application of Biologics, Osaka University Graduate School of Medicine, 2-2 Yamada-oka, Suita City, Osaka 565-0871, Japan Department of Immunopathology, World Premier International Immunology Frontier Research Center, Osaka University, 3-1 Yamada-oka, Suita City, Osaka 565-0871, Japan.

Kishimoto, Tadamitsu. Laboratory of Immune Regulation, World Premier International Immunology Frontier Research Center, Osaka University, 3-1 Yamada-oka, Suita City, Osaka 565-0871, Japan kishimoto@ifrec.osaka-u.ac.jp.
Abstract

Cytokine-targeted therapy has generated a paradigm shift in the treatment of several immune-mediated diseases. Interleukin-6 (IL-6), which was initially identified as B-cell stimulatory factor 2, is a prototypical cytokine with wide-ranging biological effects on immune cells such as B and T cells, on hepatocytes, hematopoietic cells, vascular endothelial cells and on many others. IL-6 is thus crucially involved in the regulation of immune responses, hematopoiesis and inflammation. When infections and tissue injuries occur, IL-6 is promptly synthesized and performs a protective role in host defense against such stresses and traumas. However, excessive production of IL-6 during this emergent process induces potentially fatal complications, including systemic inflammatory response syndrome (SIRS), and dysregulated, persistently high expression of IL-6 causes the onset or development of various chronic immune-mediated disorders. For these reasons, IL-6 blockade was expected to become a novel therapeutic strategy for various diseases characterized by IL-6 overproduction. Indeed, worldwide clinical trials of tocilizumab, a humanized anti-IL-6 receptor monoclonal antibody, have successfully proved its outstanding efficacy against rheumatoid arthritis, juvenile idiopathic arthritis and Castleman disease, leading to the approval of tocilizumab for the treatment of these diseases. Moreover, various reports regarding off-label use of tocilizumab strongly suggest that it will be widely applicable for acute, severe complications such as SIRS and cytokine-release syndrome and other refractory chronic immune-mediated diseases. Copyright © The Japanese Society for Immunology. 2014. All rights reserved. For permissions, please e-mail: journals.permissions@oup.com.

Publication Type

Journal Article. Research Support, Non-U.S. Gov't. Review.

Year of Publication

2015

<344>

Unique Identifier

25099958

Title

Tocilizumab, a humanized anti-IL-6R antibody, as an emerging therapeutic option for rheumatoid arthritis: molecular and cellular mechanistic insights. [Review]

Source

International Reviews of Immunology. 34(3):265-79, 2015 May.

VI 1

Status

MEDLINE

Authors

Hashizume M; Tan SL; Takano J; Ohsawa K; Hasada I; Hanasaki A; Ito I; Mihara M; Nishida K.

Authors Full Name

Hashizume, Misato; Tan, Seng-Lai; Takano, Junichi; Ohsawa, Kazunori; Hasada, Ikuo; Hanasaki, Akira; Ito, Ichiro; Mihara, Masahiko; Nishida, Keiichiro.

Institution

Hashizume, Misato. Chugai Pharmaceutical Co., Ltd., Fuji-Gotemba Research Laboratories, Gotemba, Japan.

Abstract

Pro-inflammatory cytokines play a major role in the initiation and maintenance of joint inflammation and destruction in rheumatoid arthritis (RA). The therapeutic success of biologics targeting tumour necrosis factor-alpha (TNF-alpha), interleukin-1 (IL-1) and interleukin (IL)-6 receptor (IL-6R) has broadened the treatment options for RA. These agents have potential overlapping and discriminating biologic effects, as well as different pharmacological features. Tocilizumab (TCZ) is a humanized monoclonal antibody that binds and neutralizes IL-6R, resulting in the inhibition of various IL-6-mediated biological activities, including inflammation-related, immunomodulatory and tissue/matrix remodelling effects. Randomized, double-blind, controlled phase III studies and a number of early clinical observational studies have shown that treatment with TCZ results in rapid and sustained improvement in the signs and symptoms of RA among different patient populations. These studies have established the efficacy and safety of TCZ. Here, we review the pleiotropic functions of IL-6 and how it impinges on many aspects of RA pathogenesis, and highlight the clinical experience to date with TCZ as an emerging new treatment option for RA.

Publication Type

Journal Article. Review.

Year of Publication

2015

<345>

Unique Identifier

25081063

Title

Ocular Involvement in Juvenile Idiopathic Arthritis: Classification and Treatment. [Review]

Source

Clinical Reviews in Allergy & Immunology. 49(3):271-7, 2015 Dec.

VI 1

Status

MEDLINE

Authors

Foeldvari I.

Authors Full Name

Foeldvari, Ivan.

Institution

Foeldvari, Ivan. Hamburger Zentrum fur Kinder- und Jugendrheumatologie am Klinikum Eilbek, Dehnhaide 120, 22081, Hamburg, Germany. sprechstunde@kinderrheumatologie.de.

Abstract

Juvenile idiopathic arthritis (JIA) is one of the most common rheumatic diseases in childhood with a prevalence of 4 in 1,000 children. Anterior uveitis is a well-known threatening comorbid condition of JIA and affects around 10 % of the patients depending on JIA subtype. A large proportion of children with JIA develop uveitis in the first year of disease and 73 to 90 % after 4 years. Uveitis can progress into adulthood and usually occurs as 'white uveitis', while in the JIA related to the enthesitis subtype that is symptomatic. Current studies reinforced the previous observations that early age of JIA onset, oligoarticular subtype and ANA reactivity are the main risk factors for the development of uveitis. Factors associated to worse prognosis are as follows: findings of 1+ or more vitreous cells at presentation and initial visual acuity of 20/200 or worse. The Standardization of Uveitis Nomenclature (SUN) Group took the first step to define outcome measures for uveitis, but it was established for adults. The Multinational Interdisciplinary Working Group for Uveitis in Childhood (MIWGUC) proposed outcome measures for JIA-associated uveitis incorporating the SUN criteria in 2011. The current suggested management recommends to start early a steroid-sparing effective immunomodulatory systemic treatment. Methylprednisolone intravenous pulse therapy, rituximab, tocilizumab and abatacept are promising agents. Because JIA-associated uveitis is a potentially threatening comorbidity, it is important to recognize and treat it early to prevent any visual damage that could impair visual acuity.

Publication Type

Journal Article. Review.

Year of Publication

2015

<346>
Unique Identifier
24573745
Title
Th17 cells play a critical role in the development of experimental Sjogren's syndrome.
Source
Annals of the Rheumatic Diseases. 74(6):1302-10, 2015 Jun.
VI 1
Status
MEDLINE
Authors
Lin X; Rui K; Deng J; Tian J; Wang X; Wang S; Ko KH; Jiao Z; Chan VS; Lau CS; Cao X; Lu L.
Authors Full Name
Lin, Xiang; Rui, Ke; Deng, Jun; Tian, Jie; Wang, Xiaohui; Wang, Shengjun; Ko, King-Hung; Jiao, Zhijun; Chan, Vera Sau-Fong; Lau, Chak Sing; Cao, Xuetao; Lu, Liwei.
Institution
Lin, Xiang. Department of Pathology and Center of Infection and Immunology, Shenzhen Institute of Research and Innovation, The University of Hong Kong, Hong Kong.
Rui, Ke. Department of Immunology, School of Medical Science and Laboratory Medicine, Jiangsu University, Zhenjiang, China.
Deng, Jun. Department of Pathology and Center of Infection and Immunology, Shenzhen Institute of Research and Innovation, The University of Hong Kong, Hong Kong.
Tian, Jie. Department of Immunology, School of Medical Science and Laboratory Medicine, Jiangsu University, Zhenjiang, China.
Wang, Xiaohui. Department of Pathology and Center of Infection and Immunology, Shenzhen Institute of Research and Innovation, The University of Hong Kong, Hong Kong.
Wang, Shengjun. Department of Immunology, School of Medical Science and Laboratory Medicine, Jiangsu University, Zhenjiang, China.
Ko, King-Hung. Department of Pathology and Center of Infection and Immunology, Shenzhen Institute of Research and Innovation, The University of Hong Kong, Hong Kong.
Jiao, Zhijun. Zhenjiang Key Laboratory of Medical Immunology, Department of Laboratory Medicine, Affiliated Hospital of Jiangsu University, Zhenjiang, China.
Chan, Vera Sau-Fong. Department of Medicine, The University of Hong Kong, Hong Kong.

Lau, Chak Sing. Department of Medicine, The University of Hong Kong, Hong Kong.
Cao, Xuetao. National Key Laboratory of Medical Immunology & Institute of Immunology, Second Military Medical University, Shanghai, China.
Lu, Liwei. Department of Pathology and Center of Infection and Immunology, Shenzhen Institute of Research and Innovation, The University of Hong Kong, Hong Kong Department of Immunology, School of Medical Science and Laboratory Medicine, Jiangsu University, Zhenjiang, China.
Abstract
OBJECTIVE: Although Th17 cells have been increasingly recognised as an important effector in various autoimmune diseases, their function in the pathogenesis of Sjogren's syndrome (SS) remains largely uncharacterised. This study aims to determine the role of Th17 cells in the development of experimental SS (ESS).

METHODS: The ESS was induced in wildtype and IL-17A knockout (IL-17 KO) C57BL/6 mice immunised with salivary glands (SG) proteins. Phenotypic analysis of immune cells in the draining cervical lymph nodes (CLN) and SG was performed by flow cytometry and immunofluorescence microscopy. To determine the role of Th17 cells in ESS, immunised IL-17 KO mice were adoptively transferred with in vitro-generated Th17 cells and monitored for SS development. The salivary flow rate was measured, whereas inflammatory infiltration and tissue destruction in SG were assessed by histopathology.

RESULTS: SG protein-immunised mice developed overt SS symptoms with increased Th17 cells detected in CLN and within lymphocytic foci in inflamed SG. Notably, immunised IL-17 KO mice were completely resistant for SS induction, showing no evidence of disease symptoms and histopathological changes in SG. Adoptive transfer of Th17 cells rapidly induced the onset of ESS in immunised IL-17 KO mice with markedly reduced saliva secretion, elevated autoantibody production and pronounced inflammation and tissue damage in SG.

CONCLUSIONS: Our findings have defined a critical role of Th17 cells in the pathogenesis of ESS. Further studies may validate Th17 cell as a potential target for treating SS. Copyright Published by the BMJ Publishing Group Limited. For permission to use (where not already granted under a licence) please go to http://group.bmj.com/group/rights-licensing/permissions.
Publication Type
Journal Article. Research Support, Non-U.S. Gov't.
Year of Publication
2015

<347>

Unique Identifier

25627297

Title

[Vaccination in patients from Brasilia cohort with early rheumatoid arthritis]. [Portuguese]

Source

Revista Brasileira de Reumatologia. 54(5):349-55, 2014 Sep-Oct.

VI 1

Status

MEDLINE

Authors

Muniz LF; Silva CR; Costa TF; da Mota LM.

Authors Full Name

Muniz, Luciana Feitosa; Silva, Carolina Rocha; Costa, Thais Ferreira; da Mota, Licia Maria Henrique.

Institution

Muniz, Luciana Feitosa. Hospital Universitario de Brasilia, Universidade de Brasilia, Brasilia, DF, Brasil. Electronic address: luciana_muniz@yahoo.com.br.

Silva, Carolina Rocha. Hospital Universitario de Brasilia, Universidade de Brasilia, Brasilia, DF, Brasil.

Costa, Thais Ferreira. Hospital Universitario de Brasilia, Universidade de Brasilia, Brasilia, DF, Brasil.

da Mota, Licia Maria Henrique. Hospital Universitario de Brasilia, Universidade de Brasilia, Brasilia, DF, Brasil.

Abstract

INTRODUCTION: Patients with a diagnosis of rheumatoid arthritis (RA) are at increased risk of infections. Vaccination is a recommended preventive measure. There are no studies evaluating the practice of vaccination in patients with early RA.

OBJECTIVES: To evaluate the frequency of vaccination and the orientation (by the doctor) about vaccines among patients with early RA diagnosis.

METHODS: Cross-sectional study including patients from the early RA Brasilia cohort. Demographic data, disease activity index (Disease Activity Score 28 - DAS28), functional disability (Health Assessment Questionnaire - HAQ), and data on treatment and vaccination after diagnosis of RA were analyzed.

RESULTS: 68 patients were evaluated, 94.1% women, mean age 50.7+/-13.2 years. DAS28 was 3.65+/-1.64, and HAQ was 0.70. Most patients (63%) had vaccination card. Only five patients (7.3%) were briefed by the doctor about the use of vaccines. Patients were vaccinated for MMR (8.8%), tetanus (44%), yellow fever (44%), hepatitis B (22%), influenza (42%), H1N1 (61.76%), pneumonia (1.4%), meningitis (1.4%), and chickenpox (1.4%). All patients vaccinated with live attenuated virus were undergoing immunosuppressive therapy, and were vaccinated inadvertently, without medical supervision. There was no association between the use of any vaccine and disease activity, functional disability, years of education, lifestyle, and comorbidities.

CONCLUSION: Patients were infrequently briefed by the physician regarding use of vaccines, with high frequency of inadvertent vaccination with live attenuated component, while immunization with killed virus was below the recommended level. Copyright © 2014 Elsevier Editora Ltda. All rights reserved.

Publication Type

Journal Article.

Year of Publication

2014

<348>

Unique Identifier

25546788

Title

7th International Immunoglobulin Conference: Immunomodulation.

Source

Clinical & Experimental Immunology. 178 Suppl 1:123, 2014 Dec.

VI 1

Status

MEDLINE

Authors
  Danieli MG; Shoenfeld Y.
Authors Full Name
  Danieli, M G; Shoenfeld, Y.
Institution
  Danieli, M G. Universita Politecnica delle Marche, Ancona, Italy.
Abstract
  Rheumatoid arthritis (RA) is a debilitating autoimmune disease that is usually treated aggressively to slow the rate of joint destruction. The therapeutic strategy used at the French centre, described here, is to use the non-biological disease-modifying drug, methotrexate, as first-line therapy and to add biological agents as second-line treatment. The two other autoimmune diseases discussed in this session were immunobullous skin diseases, and secondary recurrent miscarriage (RM). In the former conditions, low levels of pathogenic autoantibodies can be achieved with adjuvant intravenous immunoglobulin (IVIg) therapy, usually in combination with an immunosuppressant. Secondary RM has an autoimmune basis, as shown by high tumour necrosis factor (TNF)-alpha levels and specific human leucocyte antigen (HLA) polymorphisms. Although the mechanism is not yet known, IVIg may also be an effective treatment, despite the generally low doses used in published studies. Copyright © 2014 British Society for Immunology.
Publication Type
  Journal Article.
Year of Publication
  2014

<349>
Unique Identifier
  25517733
Title
  Therapeutic vaccination with TNF-Kinoid in TNF antagonist-resistant rheumatoid arthritis: a phase II randomized, controlled clinical trial.
Source
  PLoS ONE [Electronic Resource]. 9(12):e113465, 2014.
VI 1
Status

MEDLINE

Authors

Durez P; Vandepapeliere P; Miranda P; Toncheva A; Berman A; Kehler T; Mociran E; Fautrel B; Mariette X; Dhellin O; Fanget B; Ouary S; Grouard-Vogel G; Boissier MC.

Authors Full Name

Durez, Patrick; Vandepapeliere, Pierre; Miranda, Pedro; Toncheva, Antoaneta; Berman, Alberto; Kehler, Tatjana; Mociran, Eugenia; Fautrel, Bruno; Mariette, Xavier; Dhellin, Olivier; Fanget, Bernard; Ouary, Stephane; Grouard-Vogel, Geraldine; Boissier, Marie-Christophe.

Institution

Durez, Patrick. Service et Pole de Rhumatologie, Cliniques Universitaires Saint-Luc, Brussels, Belgium; Institut de Recherche Experimentale et Clinique, Universite catholique de Louvain, Rheumatology, Brussels, Belgium.

Vandepapeliere, Pierre. Neovacs S.A., Paris, France.

Miranda, Pedro. Centro de Estudios Reumatologicos, Santiago de Chile, Chile.

Toncheva, Antoaneta. National Multiprofile Transport Hospital "Tzar Boris III", Sofia, Bulgaria.

Berman, Alberto. Centro Medico Privado de Reumatologia, San Miguel de Tucuman, Argentina.

Kehler, Tatjana. Thalassotherapia Opatija, Opatija, Croatia.

Mociran, Eugenia. Emergency County Hospital "Dr Constantin Opris", Baia Mare, Romania.

Fautrel, Bruno. UPMC, GRC 08 (EEMOIS), Institut Pierre Louis d'Epidemiologie et Sante Publique, Paris, France; AP-HP, Pitie Salpetriere University Hospital, Dept of Rheumatology, Paris, France.

Mariette, Xavier. Universite Paris-Sud, Hopitaux Universitaires Paris-Sud, AP-HP, INSERM U1012, Le Kremlin-Bicetre, France.

Dhellin, Olivier. Neovacs S.A., Paris, France.

Fanget, Bernard. Neovacs S.A., Paris, France.

Ouary, Stephane. Neovacs S.A., Paris, France.

Grouard-Vogel, Geraldine. Neovacs S.A., Paris, France.

Boissier, Marie-Christophe. INSERM U1125, Bobigny, France; Universite Paris 13, Sorbonne Paris Cite, Bobigny, France; AP-HP, Avicenne University Hospital, Department of Rheumatology, Bobigny, France.

Abstract

OBJECTIVES: Active immunization, or vaccination, with tumor necrosis factor (TNF)-Kinoid (TNF-K) is a novel approach to induce polyclonal anti-TNF antibodies in immune-mediated inflammatory diseases. This study was performed to transfer the proof of concept obtained in mice model of rheumatoid arthritis (RA) into human. We designed a pilot study to demonstrate the feasibility of therapeutic vaccination in RA.

METHODS: This was a phase IIa, placebo-controlled, multicenter study in adults with RA who previously experienced secondary failure of TNF antagonists. Patients were immunized intramuscularly with 2 or 3 doses of placebo (n = 10) or 90 (n = 6), 180 (n = 12), or 360 micro g TNF-K (n = 12). The primary objective was to identify the best dose and schedule based on anti-TNF antibody titers. Clinical symptoms and safety were assessed during 12 months and solicited reactions for 7 days after each injection.

RESULTS: The highest anti-TNF antibody response was detected in patients immunized with 360 micro g TNF-K and with 3 injections, although this difference was not significant with all other groups. Similar proportions of patients receiving TNF-K and placebo reported adverse events up to month 12. Serious adverse events were reported by 4 patients treated with TNF-K (13.3%) and 3 treated with placebo (30.0%), all unrelated to treatment. At month 12, DAS28-CRP, tender and swollen joint counts, and HAQ scores decreased significantly more in patients who exhibited anti-TNF antibody response than in patients who did not.

CONCLUSIONS: TNF-K therapeutic vaccination induced dose- and schedule-dependent anti-TNF antibodies in RA patients and was well tolerated. Patients who developed anti-TNF antibodies showed a trend toward clinical improvement. Although the most aggressive dose and schedule, i.e. 360 mg dose administered 3 times, did show a strong trend of higher antibody response, further studies are warranted to examine even higher and more frequent doses in order to establish the best conditions for clinical improvement.

TRIAL REGISTRATION: ClinicalTrials.gov NCT01040715.

Publication Type

Clinical Trial, Phase II. Journal Article. Multicenter Study. Randomized Controlled Trial. Research Support, Non-U.S. Gov't.

Year of Publication

2014

<350>

Unique Identifier

25479932

Title

[Immunization in children and adolescents with rheumatic and musculoskeletal diseases].
[German]
Source
Zeitschrift fur Rheumatologie. 73(10):878-89, 2014 Dec.
VI 1
Status
MEDLINE
Authors
Minden K; Speth F; Huppertz HI; Borte M.
Authors Full Name
Minden, K; Speth, F; Huppertz, H-I; Borte, M.
Institution
Minden, K. Universitatskinderklinik Charite, Campus Virchow, Otto-Heubner-Centrum, SPZ, Deutsches Rheuma-Forschungszentrum Berlin, Augustenburger Platz 1, 13353, Berlin, Deutschland, minden@drfz.de.
Abstract
BACKGROUND: Children and adolescents with inflammatory rheumatic diseases have a disease and treatment-related increased risk of infections. This risk includes vaccine-preventable diseases; therefore, vaccinations represent an important preventive measure against infection in these patients. However, approximately one in three patients with a juvenile rheumatic disease is nowadays still inadequately vaccinated, mostly due to uncertainty regarding the efficacy and safety of vaccination in these patients.

OBJECTIVES: This paper summarizes the available evidence regarding the efficacy and safety of vaccinations in children and adolescents with rheumatic diseases and gives recommendations for the clinical practice.

RESULTS AND PERSPECTIVES: Almost 2000 children and adolescents with rheumatic diseases were examined in the more than 30 previously published vaccination studies, comprising nearly all standard vaccinations in the immunization schedule. The immunogenicity was usually sufficient and there was no evidence of a relevant aggravation of the underlying disease. Recommendations for the clinical practice are given also considering data beyond pediatric rheumatology; however, a final benefit-risk assessment is not yet possible.
Publication Type
English Abstract. Journal Article. Meta-Analysis.
Year of Publication
2014

<351>

Unique Identifier

25427994

Title

Chronic fatigue syndrome and fibromyalgia following immunization with the hepatitis B vaccine: another angle of the 'autoimmune (auto-inflammatory) syndrome induced by adjuvants' (ASIA).

Source

Immunologic Research. 60(2-3):376-83, 2014 Dec.

VI 1

Status

MEDLINE

Authors

Agmon-Levin N; Zafrir Y; Kivity S; Balofsky A; Amital H; Shoenfeld Y.

Authors Full Name

Agmon-Levin, Nancy; Zafrir, Yaron; Kivity, Shaye; Balofsky, Ari; Amital, Howard; Shoenfeld, Yehuda.

Institution

Agmon-Levin, Nancy. The Zabludowicz Center for Autoimmune Diseases, Chaim Sheba Medical Center, 52621, Tel-Hashomer, Israel.

Abstract

The objectives of this study were to gather information regarding demographic and clinical characteristics of patients diagnosed with either fibromyalgia (FM) or chronic fatigue (CFS) following hepatitis B vaccination (HBVv) and furthermore to apply the recently suggested criteria of autoimmune (auto-inflammatory) syndromes induced by adjuvants (ASIA), in the aim of identifying common characteristics that may suggest an association between fibromyalgia, chronic fatigue and HBV vaccination. Medical records of 19 patients with CFS and/or fibromyalgia following HBVv immunization were analyzed. All of which were immunized during 1990-2008 in different centers in the USA. All medical records were evaluated for demographics, medical history, the number of vaccine doses, as well as immediate and long term post-immunization adverse events and clinical manifestations. In addition, available blood tests, imaging results, treatments and outcomes were analyzed. ASIA criteria were applied to all patients. The mean age of patients was 28.6 +/- 11 years, of which 68.4 % were females. 21.05 % had either

personal or familial background of autoimmune disease. The mean latency period from the last dose of HBVv to onset of symptoms was 38.6 +/- 79.4 days, ranging from days to a year. Eight (42.1 %) patients continued with the immunization program despite experiencing adverse events. Manifestations that were commonly reported included neurological manifestations (84.2 %), musculoskeletal (78.9 %), psychiatric (63.1 %), fatigue (63.1 %), gastrointestinal complains (58 %) and mucocutaneous manifestations (36.8 %). Autoantibodies were detected in 71 % of patients tested. All patients fulfilled the ASIA criteria. This study suggests that in some cases CFS and FM can be temporally related to immunization, as part of ASIA syndrome. The appearance of adverse event during immunization, the presence of autoimmune susceptibility and higher titers of autoantibodies all can be suggested as risk factors. ASIA criteria were fulfilled in all patients eluding the plausible link between ASIA and CFS/FM.

Publication Type

Journal Article.

Year of Publication

2014

<352>

Unique Identifier

25369029

Title

Suppression of proteoglycan-induced autoimmune arthritis by myeloid-derived suppressor cells generated in vitro from murine bone marrow.

Source

PLoS ONE [Electronic Resource]. 9(11):e111815, 2014.

VI 1

Status

MEDLINE

Authors

Kurko J; Vida A; Ocsko T; Tryniszewska B; Rauch TA; Glant TT; Szekanecz Z; Mikecz K.

Authors Full Name

Kurko, Julia; Vida, Andras; Ocsko, Timea; Tryniszewska, Beata; Rauch, Tibor A; Glant, Tibor T; Szekanecz, Zoltan; Mikecz, Katalin.

Institution

Kurko, Julia. Section of Molecular Medicine, Department of Orthopedic Surgery, Rush University Medical Center, Chicago, Illinois, United States of America; Department of Rheumatology, University of Debrecen, Faculty of Medicine, Debrecen, Hungary.

Vida, Andras. Section of Molecular Medicine, Department of Orthopedic Surgery, Rush University Medical Center, Chicago, Illinois, United States of America.

Ocsko, Timea. Section of Molecular Medicine, Department of Orthopedic Surgery, Rush University Medical Center, Chicago, Illinois, United States of America.

Tryniszewska, Beata. Section of Molecular Medicine, Department of Orthopedic Surgery, Rush University Medical Center, Chicago, Illinois, United States of America.

Rauch, Tibor A. Section of Molecular Medicine, Department of Orthopedic Surgery, Rush University Medical Center, Chicago, Illinois, United States of America.

Glant, Tibor T. Section of Molecular Medicine, Department of Orthopedic Surgery, Rush University Medical Center, Chicago, Illinois, United States of America.

Szekanecz, Zoltan. Department of Rheumatology, University of Debrecen, Faculty of Medicine, Debrecen, Hungary.

Mikecz, Katalin. Section of Molecular Medicine, Department of Orthopedic Surgery, Rush University Medical Center, Chicago, Illinois, United States of America.

Abstract

BACKGROUND: Myeloid-derived suppressor cells (MDSCs) are innate immune cells capable of suppressing T-cell responses. We previously reported the presence of MDSCs with a granulocytic phenotype in the synovial fluid (SF) of mice with proteoglycan (PG)-induced arthritis (PGIA), a T cell-dependent autoimmune model of rheumatoid arthritis (RA). However, the limited amount of SF-MDSCs precluded investigations into their therapeutic potential. The goals of this study were to develop an in vitro method for generating MDSCs similar to those found in SF and to reveal the therapeutic effect of such cells in PGIA.

METHODS: Murine bone marrow (BM) cells were cultured for 3 days in the presence of granulocyte macrophage colony-stimulating factor (GM-CSF), interleukin-6 (IL-6), and granulocyte colony-stimulating factor (G-CSF). The phenotype of cultured cells was analyzed using flow cytometry, microscopy, and biochemical methods. The suppressor activity of BM-MDSCs was tested upon co-culture with activated T cells. To investigate the therapeutic potential of BM-MDSCs, the cells were injected into SCID mice at the early stage of adoptively transferred PGIA, and their effects on the clinical course of arthritis and PG-specific immune responses were determined.

RESULTS: BM cells cultured in the presence of GM-CSF, IL-6, and G-CSF became enriched in MDSC-like cells that showed greater phenotypic heterogeneity than MDSCs present in SF. BM-

MDSCs profoundly inhibited both antigen-specific and polyclonal T-cell proliferation primarily via production of nitric oxide. Injection of BM-MDSCs into mice with PGIA ameliorated arthritis and reduced PG-specific T-cell responses and serum antibody levels.

CONCLUSIONS: Our in vitro enrichment strategy provides a SF-like, but controlled microenvironment for converting BM myeloid precursors into MDSCs that potently suppress both T-cell responses and the progression of arthritis in a mouse model of RA. Our results also suggest that enrichment of BM in MDSCs could improve the therapeutic efficacy of BM transplantation in RA.

Publication Type

Journal Article. Research Support, N.I.H., Extramural. Research Support, Non-U.S. Gov't.

Year of Publication

2014

<353>

Unique Identifier

25275771

Title

Fibromyalgia-like illness in 2 girls after human papillomavirus vaccination.

Source

JCR: Journal of Clinical Rheumatology. 20(7):392-3, 2014 Oct.

VI 1

Status

MEDLINE

Authors

Martinez-Lavin M.

Authors Full Name

Martinez-Lavin, Manuel.

Institution

Martinez-Lavin, Manuel. Rheumatology Department Instituto Nacional de Cardiologia Ignacio Chavez Mexico City Mexico drmartinezlavin@gmail.com.

Publication Type

Case Reports. Letter.

Year of Publication
2014

<354>
Unique Identifier
25264193
Title
Substance P ameliorates collagen II-induced arthritis in mice via suppression of the inflammatory response.
Source
Biochemical & Biophysical Research Communications. 453(1):179-84, 2014 Oct 10.
VI 1
Status
MEDLINE
Authors
Hong HS; Son Y.
Authors Full Name
Hong, Hyun Sook; Son, Youngsook.
Institution
Hong, Hyun Sook. College of Medicine, East-West Medical Research Institute, Kyung Hee University, 1, Hoegi-dong, Dongdaemun-gu, Seoul 130-702, Republic of Korea.
Son, Youngsook. Graduate School of Biotechnology & Department of Genetic Engineering, College of Life Science, Kyung Hee University Global Campus, Seochun-dong, Kiheung-ku, Yong In 441-706, Republic of Korea. Electronic address: ysson@khu.ac.kr.
Abstract
Current rheumatoid arthritis (RA) therapies such as biologics inhibiting pathogenic cytokines substantially delay RA progression. However, patient responses to these agents are not always complete and long lasting. This study explored whether substance P (SP), an 11 amino acids long endogenous neuropeptide with the novel ability to mobilize mesenchymal stem cells (MSC) and modulate injury-mediated inflammation, can inhibit RA progression. SP efficacy was evaluated by paw swelling, clinical arthritis scoring, radiological analysis, histological analysis of cartilage destruction, and blood levels of tumor necrosis factor-alpha (TNF-alpha) interleukin (IL)-10, and IL-17 in vivo. SP treatment significantly reduced local inflammatory signs, mean arthritis

scores, degradation of joint cartilage, and invasion of inflammatory cells into the synovial tissues. Moreover, the SP treatment markedly reduced the size of spleens enlarged by excessive inflammation in CIA, increased IL-10 levels, and decreased TNF-alpha and IL-17 levels. Mobilization of stem cells and induction of T(reg) and M2 type macrophages in the circulation were also increased by the SP treatment. These effect of SP might be associated with the suppression of inflammatory responses in RA and, furthermore, blockade of RA progression. Our results propose SP as a potential therapeutic for autoimmune-related inflammatory diseases.
Copyright © 2014. Published by Elsevier Inc.
Publication Type
  Journal Article. Research Support, Non-U.S. Gov't.
Year of Publication
  2014

<355>
Unique Identifier
  25213064
Title
  Successful rapid tocilizumab desensitization in a patient with Still disease.
Source
  The Journal of Allergy & Clinical Immunology in Practice. 2(5):631-2, 2014 Sep-Oct.
VI 1
Status
  MEDLINE
Authors
  Justet A; Neukirch C; Poubeau P; Arrault X; Borie R; Dombret MC; Crestani B.
Authors Full Name
  Justet, Aurelien; Neukirch, Catherine; Poubeau, Patrice; Arrault, Xavier; Borie, Raphael; Dombret, Marie-Christine; Crestani, Bruno.
Institution
  Justet, Aurelien. Service de Pneumologie A, Assistance Publique Hopitaux de Paris, Hopital Bichat, Universite Paris Diderot, Paris, France. Electronic address: aurelienjustet@yahoo.fr.
  Neukirch, Catherine. Service de Pneumologie A, Assistance Publique Hopitaux de Paris, Hopital Bichat, Universite Paris Diderot, Paris, France.

Poubeau, Patrice. Service de Medecine Interne, Centre Hospitalo-universitaire, Sud Reunion, Saint Pierre, France.
Arrault, Xavier. Service de Pharmacie Clinique et des Biomateriaux, Assistance Publique Hopitaux de Paris, Hopital Bichat, Paris, France.
Borie, Raphael. Service de Pneumologie A, Assistance Publique Hopitaux de Paris, Hopital Bichat, Universite Paris Diderot, Paris, France.
Dombret, Marie-Christine. Service de Pneumologie A, Assistance Publique Hopitaux de Paris, Hopital Bichat, Universite Paris Diderot, Paris, France.
Crestani, Bruno. Service de Pneumologie A, Assistance Publique Hopitaux de Paris, Hopital Bichat, Universite Paris Diderot, Paris, France.
Publication Type
Case Reports. Journal Article.
Year of Publication
2014

<356>
Unique Identifier
25166212
Title
The quest for personalized B-cell depletion therapy in rheumatic disease.
Source
Arthritis Research & Therapy. 16(3):116, 2014.
VI 1
Status
MEDLINE
Authors
Nistala K; Mauri C.
Authors Full Name
Nistala, Kiran; Mauri, Claudia.
Abstract
Although B cell depletion therapy (BCDT) is now a well-accepted therapeutic option in autoimmune rheumatic disease, a significant proportion of patients remain resistant to therapy. .19pt?>A more challenging clinical problem is the high rate of relapse after B cell reconstitution,

as well as the difficulty in predicting the exact timing of that relapse. In this article, we consider the immunological mechanisms that may account for the heterogeneity of clinical response to BCDT. Understanding how BCDT alters the balance between different B cell subsets, some pathogenic and some regulatory, may help us correctly target BCDT to the right patients, and thereby improve treatment responses in rheumatic disease.

Publication Type

Journal Article.

Year of Publication

2014

<357>

Unique Identifier

25134101

Title

Quantification of risk factors for herpes zoster: population based case-control study.

Source

BMJ. 348:g2911, 2014 May 13.

VI 1

Status

MEDLINE

Authors

Forbes HJ; Bhaskaran K; Thomas SL; Smeeth L; Clayton T; Langan SM.

Authors Full Name

Forbes, Harriet J; Bhaskaran, Krishnan; Thomas, Sara L; Smeeth, Liam; Clayton, Tim; Langan, Sinead M.

Institution

Forbes, Harriet J. Faculty of Epidemiology and Population Health, London School of Hygiene and Tropical Medicine, London WC1E 7HT, UK harriet.forbes@lshtm.ac.uk.

Bhaskaran, Krishnan. Faculty of Epidemiology and Population Health, London School of Hygiene and Tropical Medicine, London WC1E 7HT, UK.

Thomas, Sara L. Faculty of Epidemiology and Population Health, London School of Hygiene and Tropical Medicine, London WC1E 7HT, UK.

Smeeth, Liam. Faculty of Epidemiology and Population Health, London School of Hygiene and Tropical Medicine, London WC1E 7HT, UK.
Clayton, Tim. Faculty of Epidemiology and Population Health, London School of Hygiene and Tropical Medicine, London WC1E 7HT, UK.
Langan, Sinead M. Faculty of Epidemiology and Population Health, London School of Hygiene and Tropical Medicine, London WC1E 7HT, UK.
Abstract
OBJECTIVES: To quantify the effects of possible risk factors for herpes zoster at different ages.

DESIGN: Case-control study.

SETTING: UK Clinical Practice Research Datalink primary care data.

PARTICIPANTS: 144 959 adults diagnosed with zoster between 2000 and 2011; 549,336 age, sex, and practice matched controls.

MAIN OUTCOME MEASURES: Conditional logistic regression was used to generate adjusted odds ratios to estimate the strength of association of each potential risk factor with zoster and assess effect modification by age.

RESULTS: The median age of the cases and controls was 62 years. Factors associated with increased risk of zoster included rheumatoid arthritis (3111 (2.1%) v 8029 (1.5%); adjusted odds ratio 1.46, 99% confidence interval 1.38 to 1.55), inflammatory bowel disease (1851 (1.3%) v 5118 (0.9%); 1.36, 1.26 to 1.46), chronic obstructive pulmonary disease (6815 (4.7%) v 20 201 (3.7%); 1.32, 1.27 to 1.37), asthma (10 243 (7.1%) v 31 865 (5.8%); 1.21, 1.17 to 1.25), chronic kidney disease (8724 (6.0%) v 29 437 (5.4%); 1.14, 1.09 to 1.18), and depression (6830 (4.7%) v 22 052 (4.0%); 1.15, 1.10 to 1.20). Type 1, but not type 2, diabetes showed some association with zoster (adjusted odds ratio 1.27, 1.07 to 1.50). The relative effects of many assessed risk factors were larger in younger patients. Patients with severely immunosuppressive conditions were at greatest risk of zoster-for example, patients with lymphoma (adjusted odds ratio 3.90, 3.21 to 4.74) and myeloma (2.16, 1.84 to 2.53), who are not eligible for zoster vaccination.

CONCLUSIONS: A range of conditions were associated with increased risk of zoster. In general, the increased risk was proportionally greater in younger age groups. Current vaccines are contraindicated in people at the greatest risk of zoster, highlighting the need for alternative risk reduction strategies in these groups. Copyright © Forbes et al 2014.
Publication Type

Journal Article. Research Support, Non-U.S. Gov't.
Year of Publication
2014

<358>
Unique Identifier
25112605
Title
Adverse reactions to biologic agents and their medical management. [Review]
Source
Nature Reviews Rheumatology. 10(10):612-27, 2014 Oct.
VI 1
Status
MEDLINE
Authors
Boyman O; Comte D; Spertini F.
Authors Full Name
Boyman, Onur; Comte, Denis; Spertini, Francois.
Institution
Boyman, Onur. Department of Immunology, University Hospital Zurich, Gloriastrasse 30, CH-8091 Zurich, Switzerland.
Comte, Denis. Division of Immunology and Allergy, Centre Hospitalier Universitaire Vaudois, Rue du Bugnon 46, CH-1011 Lausanne, Switzerland.
Spertini, Francois. Division of Immunology and Allergy, Centre Hospitalier Universitaire Vaudois, Rue du Bugnon 46, CH-1011 Lausanne, Switzerland.
Abstract
Biologic agents have substantially advanced the treatment of immunological disorders, including chronic inflammatory and autoimmune diseases. However, these drugs are often associated with adverse events (AEs), including allergic, immunological and other unwanted reactions. AEs can affect almost any organ or system in the body and can occur immediately, within minutes to hours, or with a delay of several days or more after initiation of biologic therapy. Although some AEs are a direct consequence of the functional inhibition of biologic-agent-targeted antigens, the pathogenesis of other AEs results from a drug-induced imbalance of the immune system,

intermediary factors and cofactors, a complexity that complicates their prediction. Herein, we review the AEs associated with biologic therapy most relevant to rheumatic and immunological diseases, and discuss their underlying pathogenesis. We also include our recommendations for the medical management of such AEs. Increased understanding and improved risk management of AEs induced by biologic agents will enable better use of these versatile immune-response modifiers.

Publication Type

Journal Article. Research Support, Non-U.S. Gov't. Review.

Year of Publication

2014

<359>

Unique Identifier

25076314

Title

[Infections during antirheumatic treatment]. [Review] [German]

Source

Deutsche Medizinische Wochenschrift. 139(31-32):1593-5, 2014 Aug.

VI 1

Status

MEDLINE

Authors

Kneitz C.

Authors Full Name

Kneitz, C.

Institution

Kneitz, C. Schwerpunkt Rheumatologie und Klinische Immunologie, Klinik fur Innere Medizin II, Klinikum Sudstadt, Rostock.

Publication Type

Journal Article. Review.

Year of Publication

2014

<360>

Unique Identifier

25022358

Title

Update on infections and vaccinations in systemic lupus erythematosus and Sjogren's syndrome. [Review]

Source

Current Opinion in Rheumatology. 26(5):528-37, 2014 Sep.

VI 1

Status

MEDLINE

Authors

Pasoto SG; Ribeiro AC; Bonfa E.

Authors Full Name

Pasoto, Sandra G; Ribeiro, Ana C M; Bonfa, Eloisa.

Institution

Pasoto, Sandra G. Rheumatology Division, Hospital das Clinicas da Faculdade de Medicina da Universidade de Sao Paulo (USP), Sao Paulo, Brazil.

Abstract

PURPOSE OF REVIEW: To provide an update on infections in systemic lupus erythematosus (SLE) and Sjogren's syndrome, particularly addressing their role as triggers of autoimmunity, their impact on mortality, the main microorganisms, the approaches to differential diagnosis with disease flares and recommendations for vaccination.

RECENT FINDINGS: New mechanisms for autoimmunity triggered by Epstein-Barr virus and human commensal microbiota have been described. The increased risk for tuberculosis was recently demonstrated for the first time in Sjogren's syndrome. C-reactive protein was reported to be a more sensitive and specific marker for bacterial infections in SLE than procalcitonin and phagocyte-specific S100A8/A9 protein. Inactivated vaccines are well tolerated and efficacy was demonstrated for influenza vaccine. Immunogenicity is generally reduced but adequate in SLE. Prednisone or immunosuppressants are associated with decreased vaccine serological response, whereas hydroxicloroquine seems to improve vaccine immunogenicity. Other infection-preventive

measures for these diseases include antimalarials and prophylaxis for tuberculosis or Pneumocystis jirovecii.

SUMMARY: Advances in the role of infectious agents as triggers for SLE and Sjogren's syndrome have provided new insights into disease development. Knowledge on vaccine immunogenicity, safety and efficacy has improved with evidence of a generally reduced but adequate response for inactivated vaccines in SLE. Other preventive measures comprise infection prophylaxis and antimalarials.

Publication Type

Journal Article. Research Support, Non-U.S. Gov't. Review.

Year of Publication

2014

<361>

Unique Identifier

25015955

Title

Folate receptor-beta constitutes a marker for human proinflammatory monocytes.

Source

Journal of Leukocyte Biology. 96(4):563-70, 2014 Oct.

VI 1

Status

MEDLINE

Authors

Shen J; Hilgenbrink AR; Xia W; Feng Y; Dimitrov DS; Lockwood MB; Amato RJ; Low PS.

Authors Full Name

Shen, Jiayin; Hilgenbrink, Andrew R; Xia, Wei; Feng, Yang; Dimitrov, Dimiter S; Lockwood, Michael B; Amato, Robert J; Low, Philip S.

Institution

Shen, Jiayin. Department of Chemistry, Purdue University, West Lafayette, Indiana, USA;

Hilgenbrink, Andrew R. Department of Chemistry, Purdue University, West Lafayette, Indiana, USA;

Xia, Wei. Department of Chemistry, Purdue University, West Lafayette, Indiana, USA;

Feng, Yang. Protein Interactions Group, Center for Cancer and Inflammation Program, National Cancer Institute-Frederick, National Institutes of Health, Frederick, Maryland, USA;

Dimitrov, Dimiter S. Protein Interactions Group, Center for Cancer and Inflammation Program, National Cancer Institute-Frederick, National Institutes of Health, Frederick, Maryland, USA;

Lockwood, Michael B. Department of Rheumatology, Clarian Arnett Health, Lafayette, Indiana, USA; and.

Amato, Robert J. Department of Internal Medicine, Memorial Hermann Cancer Center, Division of Oncology, University of Texas Health Science Center at Houston, Medical School, Houston, Texas, USA.

Low, Philip S. Department of Chemistry, Purdue University, West Lafayette, Indiana, USA; plow@purdue.edu.

Abstract

Activated macrophages are commonly involved in the pathogenesis of inflammatory and autoimmune diseases and have been frequently reported to overexpress FR-beta. Although FR-targeted therapies aimed at eliminating activated macrophages have shown promise for treating inflammatory diseases, little work has been performed to evaluate whether other hematopoietic cells might also express FR-beta. Analysis of peripheral blood cells with a mAb to human FR-beta reveals that only monocytes express FR-beta. Molecular characterization of these circulating monocytes further demonstrates that solely the classic/proinflammatory subset (CD14(high)CD16(-)) expresses the FR and that only CD14(high)CD16(-) FR-beta(+) monocytes also display the ability to bind folate-linked molecules. Confirmation that this subset of monocytes indeed constitutes the proinflammatory subpopulation was obtained by demonstrating coexpression of FR-beta with other proinflammatory markers, including CCR2 and HLA-DR. Synovial monocytes from the joints of patients with RA were also shown to express FR-beta. As inhibition of the chemotaxis of proinflammatory monocytes into sites of inflammation has been explored frequently as a means of controlling autoimmune diseases, demonstration that FR-beta is uniquely expressed on this proinflammatory subpopulation offers a new strategy to suppress migration of inflammatory monocytes into sites of inflammation. Copyright © 2014 Society for Leukocyte Biology.

Publication Type

Journal Article. Research Support, Non-U.S. Gov't.

Year of Publication

2014

<362>

Unique Identifier

24961616

Title

The effect of antidrug antibodies on the sustainable efficacy of biologic therapies in rheumatoid arthritis: practical consequences. [Review]

Source

Expert Review of Clinical Immunology. 10(8):1049-57, 2014 Aug.

VI 1

Status

MEDLINE

Authors

Keiserman M; Codreanu C; Handa R; Xibille-Friedmann D; Mysler E; Briceno F; Akar S.

Authors Full Name

Keiserman, Mauro; Codreanu, Catalin; Handa, Rohini; Xibille-Friedmann, Daniel; Mysler, Eduardo; Briceno, Francisco; Akar, Servet.

Institution

Keiserman, Mauro. Rheumatology Section, Pontifical Catholic University RS, School of Medicine, Porto Alegre, Brazil.

Abstract

Biologic therapies, predominantly TNF-alpha inhibitors, have revolutionized the treatment of rheumatoid arthritis (RA). However, their clinical utility can be limited by the development of antidrug antibodies (ADAs). Immunogenicity is a complex phenomenon related to various drug, disease, and patient characteristics, and may be more common with the monoclonal antibodies than with etanercept, a soluble TNF receptor-Fc immunoglobulin fusion protein. Neutralizing antibodies - those that hinder bioactivity by preventing drug molecules from binding to TNF - are correlated with reduced serum drug concentrations, loss of therapeutic response, adverse events, and treatment discontinuation. Cost-effective use of these agents will depend on further research into drug and ADA assays, and how they should guide dose reduction or switching strategies.

Publication Type

Journal Article. Research Support, Non-U.S. Gov't. Review.

Year of Publication

2014

<363>

Unique Identifier

24931640

Title

Acute and probable chronic Q fever during anti-TNFalpha and anti B-cell immunotherapy: a case report.

Source

BMC Infectious Diseases. 14:330, 2014 Jun 15.

VI 1

Status

MEDLINE

Authors

Schoffelen T; den Broeder AA; Nabuurs-Franssen M; van Deuren M; Sprong T.

Authors Full Name

Schoffelen, Teske; den Broeder, Alfons A; Nabuurs-Franssen, Marrigje; van Deuren, Marcel; Sprong, Tom.

Institution

Schoffelen, Teske. Department of Internal Medicine, Radboud university medical center, Nijmegen, The Netherlands. Teske.Schoffelen@radboudumc.nl.

Abstract

BACKGROUND: Q fever is caused by the intracellular bacterium Coxiella burnetii. Initial infection can present as acute Q fever, while a minority of infected individuals develops chronic Q fever endocarditis or vascular infection months to years after initial infection. Serology is an important diagnostic tool for both acute and chronic Q fever. However, since immunosuppressive drugs may hamper the humoral immune response, diagnosis of Q fever might be blurred when these drugs are used.

CASE PRESENTATION: A 71-year-old Caucasian male was diagnosed with symptomatic acute Q fever (based on positive C. burnetii PCR followed by seroconversion) while using anti-tumor necrosis factor-alpha (anti-TNFalpha) drugs for rheumatoid arthritis (RA). He was treated for two weeks with moxifloxacin. After 24 months of follow-up, the diagnosis of probable chronic Q fever was established based on increasing anti-C. burnetii phase I IgG antibody titres in a immunocompromised patient combined with clinical suspicion of endocarditis. At the time of chronic Q fever diagnosis, he had been treated with anti B-cell therapy for 16 months. Antibiotic

therapy consisting of 1.5 years doxycycline and hydroxychloroquine was started and successfully completed and no signs of relapse were seen after more than one year of follow-up.

CONCLUSION: The use of anti-TNFalpha agents for RA in the acute phase of Q fever did not hamper the C. burnetii-specific serological response as measured by immunofluorescence assay. However, in the presented case, an intact humoral response did not prevent progression to probable chronic C. burnetii infection, most likely because essential cellular immune responses were suppressed during the acute phase of the infection. Despite the start of anti-B-cell therapy with rituximab after the acute Q fever episode, an increase in anti-C. burnetii phase I IgG antibodies was observed, supporting the notion that C. burnetii specific CD20-negative memory B-cells are responsible for this rise in antibody titres.

Publication Type

Case Reports. Journal Article. Research Support, Non-U.S. Gov't.

Year of Publication

2014

<364>

Unique Identifier

24925588

Title

Vaccination in paediatric rheumatology. [Review]

Source

Current Rheumatology Reports. 16(8):432, 2014 Aug.

VI 1

Status

MEDLINE

Authors

Akikusa JD; Crawford NW.

Authors Full Name

Akikusa, Jonathan D; Crawford, Nigel W.

Institution

Akikusa, Jonathan D. Rheumatology Unit, Department of General Medicine, Royal Children's Hospital (RCH), 50 Flemington Road, Parkville, 3052, Melbourne, Victoria, Australia, Jonathan.Akikusa@rch.org.au.

Abstract

As awareness of the risk of vaccine-preventable diseases for children with rheumatic diseases has increased, vaccination has become an important clinical consideration and focus of research in paediatric rheumatology. Conflicting reports in the literature and differing advice from national bodies regarding the safety of different vaccines for this patient population have led to confusion in the minds of many rheumatologists as to what is appropriate. This article will provide an overview of crucial aspects of the recently published European League Against Rheumatism recommendations regarding vaccination of paediatric patients with rheumatic disease, and will review advances in this field since their publication.

Publication Type

Journal Article. Review.

Year of Publication

2014

<365>

Unique Identifier

24925587

Title

Vaccinations for rheumatoid arthritis. [Review]

Source

Current Rheumatology Reports. 16(8):431, 2014 Aug.

VI 1

Status

MEDLINE

Authors

Perry LM; Winthrop KL; Curtis JR.

Authors Full Name

Perry, Lisa M; Winthrop, Kevin L; Curtis, Jeffrey R.

Institution

Perry, Lisa M. Division of Arthritis & Rheumatic Diseases, Oregon Health and Science University, 3181 SW Sam Jackson Park Rd, Portland, OR, 97239, USA.
Abstract
Patients with rheumatoid arthritis (RA) suffer an increased burden of infectious disease-related morbidity and mortality and have twice the risk of acquiring a severe infection compared to the general population. This increased risk is not only a result of the autoimmune disease but is also attributed to the immunosuppressive therapies that are commonly used in this patient population. Given the increase in infection-related risks in RA, there is great interest in mitigating such risk. A number of vaccines are available to the rheumatologist, with a handful that are of importance for RA patients in the United States. The goal of this paper is to highlight the most recent literature on the key vaccines and the specific considerations for the rheumatologist and their RA patients, with a particular focus on influenza, pneumococcal, and herpes zoster vaccines. It is important for rheumatologist to understand and be aware of which vaccines are live and what potential contraindications exist for giving vaccines to RA patients.
Publication Type
Journal Article. Research Support, N.I.H., Extramural. Research Support, Non-U.S. Gov't. Review.
Year of Publication
2014

<366>
Unique Identifier
24896630
Title
Specific therapy to regulate inflammation in rheumatoid arthritis: molecular aspects. [Review]
Source
Immunotherapy. 6(5):623-36, 2014.
VI 1
Status
MEDLINE
Authors
Garcia-Hernandez MH; Gonzalez-Amaro R; Portales-Perez DP.
Authors Full Name

Garcia-Hernandez, Mariana H; Gonzalez-Amaro, Roberto; Portales-Perez, Diana Patricia.
Institution
Garcia-Hernandez, Mariana H. Laboratory of Immunology & Cellular & Molecular Biology, Facultad de Ciencias Quimicas, Universidad Autonoma de San Luis Potosi, San Luis Potosi, SLP, Mexico.
Abstract
Rheumatoid arthritis (RA) is a chronic inflammatory disease in which persistent inflammation of synovial tissue results in a progressive functional decline of the joint and premature mortality. TNF inhibitors were the first biological disease-modifying antirheumatic drugs (DMARDs) used to treat RA. Since then, new biological drugs have emerged, such as inhibitors of IL-1, IL-6 and others, with different mechanisms of action that include the depletion of B cells and the inhibition of T-cell costimulation. Recently, RA treatments have incorporated the use of synthetic DMARDs. This review describes the molecular aspects of the mechanisms of action of biological and synthetic DMARDs, discusses the adverse effects and limitations of established therapies and analyses the alternative approaches to RA treatment.
Publication Type
Journal Article. Review.
Year of Publication
2014

<367>
Unique Identifier
24889761
Title
Rituximab impairs immunoglobulin (Ig)M and IgG (subclass) responses after influenza vaccination in rheumatoid arthritis patients.
Source
Clinical & Experimental Immunology. 178(1):40-7, 2014 Oct.
VI 1
Status
MEDLINE
Authors
Westra J; van Assen S; Wilting KR; Land J; Horst G; de Haan A; Bijl M.

Authors Full Name
Westra, J; van Assen, S; Wilting, K R; Land, J; Horst, G; de Haan, A; Bijl, M.
Institution
Westra, J. Department of Rheumatology and Clinical Immunology, University of Groningen, Groningen, the Netherlands.
Abstract
Rituximab (RTX) treatment in rheumatoid arthritis (RA) patients severely hampers humoral response after influenza vaccination as determined by haemagglutination inhibition assay (HI). It is not known whether HI reflects both immunoglobulin (Ig)M and IgG (subclass) influenza response, and whether IgM antibodies contribute to the low rate of influenza infection seen in RA patients. Twenty RA patients on methotrexate (MTX), 23 on RTX and 28 healthy controls (HC) received trivalent influenza subunit vaccination. Before and 28 days after vaccination, H1N1- and H3N2-specific antibodies were measured by HI and by IgM and IgG (subclass) enzyme-linked immunosorbent assay (ELISA). B cell activating factor (BAFF) levels were determined in serum samples before vaccination. Vaccination induced a significant increase of IgM and IgG (IgG1 and IgG3) antibodies against both strains in the HC and MTX groups (all $P < 0.01$), but not in the RTX group. HI correlated significantly in all cases with IgG (IgG1) but not with IgM. In RTX late patients (RTX treatment 6-10 months before vaccination), IgG (IgG1 and IgG3) response to vaccination was restored, but not IgM response. BAFF levels were significantly increased in RA-RTX patients and correlated with total IgG levels. Haemagglutination inhibition assay, used as gold standard, detects primarily IgG (IgG1) responses. IgM- and IgG influenza-specific antibodies increase after vaccination in HC and RA patients except in patients on RTX treatment. BAFF levels are increased in both early and late RTX-treated patients, but do not correlate with an influenza-specific antibody response. Copyright © 2014 British Society for Immunology.
Publication Type
Journal Article. Research Support, Non-U.S. Gov't.
Year of Publication
2014

<368>
Unique Identifier
24887388
Title

Juvenile arthritis after haematopoietic stem cell transplantation.

Source

Bone Marrow Transplantation. 49(9):1244-5, 2014 Sep.

VI 1

Status

MEDLINE

Authors

Tronconi E; Miniaci A; Prete A; Masetti R; Pession A.

Authors Full Name

Tronconi, E; Miniaci, A; Prete, A; Masetti, R; Pession, A.

Institution

Tronconi, E. Department of Pediatrics, University of Bologna Sant'Orsola-Malpighi Hospital, Bologna, Italy.

Miniaci, A. Department of Pediatrics, University of Bologna Sant'Orsola-Malpighi Hospital, Bologna, Italy.

Prete, A. Department of Pediatrics, University of Bologna Sant'Orsola-Malpighi Hospital, Bologna, Italy.

Masetti, R. Department of Pediatrics, University of Bologna Sant'Orsola-Malpighi Hospital, Bologna, Italy.

Pession, A. Department of Pediatrics, University of Bologna Sant'Orsola-Malpighi Hospital, Bologna, Italy.

Publication Type

Case Reports. Letter.

Year of Publication

2014

<369>

Unique Identifier

24886976

Title

Type 1 regulatory T cells specific for collagen type II as an efficient cell-based therapy in arthritis.

Source

Arthritis Research & Therapy. 16(3):R115, 2014 May 22.
VI 1
Status
MEDLINE
Authors
Asnagli H; Martire D; Belmonte N; Quentin J; Bastian H; Boucard-Jourdin M; Fall PB; Mausset-Bonnefont AL; Mantello-Moreau A; Rouquier S; Marchetti I; Jorgensen C; Foussat A; Louis-Plence P.
Authors Full Name
Asnagli, Helene; Martire, Delphine; Belmonte, Nathalie; Quentin, Julie; Bastian, Herve; Boucard-Jourdin, Mathilde; Fall, Papa Babacar; Mausset-Bonnefont, Anne-Laure; Mantello-Moreau, Amelie; Rouquier, Sandrine; Marchetti, Irene; Jorgensen, Christian; Foussat, Arnaud; Louis-Plence, Pascale.
Abstract
INTRODUCTION: Regulatory T (Treg) cells play a crucial role in preventing autoimmune diseases and are an ideal target for the development of therapies designed to suppress inflammation in an antigen-specific manner. Type 1 regulatory T (Tr1) cells are defined by their capacity to produce high levels of interleukin 10 (IL-10), which contributes to their ability to suppress pathological immune responses in several settings. The aim of this study was to evaluate the therapeutic potential of collagen type II-specific Tr1 (Col-Treg) cells in two models of rheumatoid arthritis (RA) in mice.

METHODS: Col-Treg clones were isolated and expanded from collagen-specific TCR transgenic mice. Their cytokine secretion profile and phenotype characterization were studied. The therapeutic potential of Col-Treg cells was evaluated after adoptive transfer in collagen-antibody- and collagen-induced arthritis models. The in vivo suppressive mechanism of Col-Treg clones on effector T-cell proliferation was also investigated.

RESULTS: Col-Treg clones are characterized by their specific cytokine profile (IL-10(high)IL-4(neg)IFN-gamma(int)) and mediate contact-independent immune suppression. They also share with natural Tregs high expression of GITR, CD39 and granzyme B. A single infusion of Col-Treg cells reduced the incidence and clinical symptoms of arthritis in both preventive and curative settings, with a significant impact on collagen type II antibodies. Importantly, injection of antigen-specific Tr1 cells decreased the proliferation of antigen-specific effector T cells in vivo significantly.

CONCLUSIONS: Our results demonstrate the therapeutic potential of Col-Treg cells in two models of RA, providing evidence that Col-Treg could be an efficient cell-based therapy for RA patients whose disease is refractory to current treatments.

Publication Type

Journal Article. Research Support, Non-U.S. Gov't.

Year of Publication

2014

<370>

Unique Identifier

24878740

Title

Increase of hemoglobin levels by anti-IL-6 receptor antibody (tocilizumab) in rheumatoid arthritis.

Source

PLoS ONE [Electronic Resource]. 9(5):e98202, 2014.

VI 1

Status

MEDLINE

Authors

Hashimoto M; Fujii T; Hamaguchi M; Furu M; Ito H; Terao C; Yamamoto K; Yamamoto W; Matsuo T; Mori M; Ohmura K; Kawabata H; Mimori T.

Authors Full Name

Hashimoto, Motomu; Fujii, Takao; Hamaguchi, Masahide; Furu, Moritoshi; Ito, Hiromu; Terao, Chikashi; Yamamoto, Keiichi; Yamamoto, Wataru; Matsuo, Takashi; Mori, Masato; Ohmura, Koichiro; Kawabata, Hiroshi; Mimori, Tsuneyo.

Institution

Hashimoto, Motomu. Department of the Control for Rheumatic Diseases, Graduate School of Medicine, Kyoto University, Kyoto, Japan.

Fujii, Takao. Department of the Control for Rheumatic Diseases, Graduate School of Medicine, Kyoto University, Kyoto, Japan; Department of Rheumatology and Clinical Immunology, Graduate School of Medicine, Kyoto University, Kyoto, Japan.

Hamaguchi, Masahide. Department of Experimental Immunology, Immunology Frontier Research Center, Osaka University, Osaka, Japan.

Furu, Moritoshi. Department of the Control for Rheumatic Diseases, Graduate School of Medicine, Kyoto University, Kyoto, Japan.

Ito, Hiromu. Department of the Control for Rheumatic Diseases, Graduate School of Medicine, Kyoto University, Kyoto, Japan; Department of Orthopedic Surgery, Graduate School of Medicine, Kyoto University, Kyoto, Japan.

Terao, Chikashi. Center for Genomic Medicine, Graduate School of Medicine, Kyoto University, Kyoto, Japan.

Yamamoto, Keiichi. Division of Clinical and Epidemiological Database, Department of Information Governance, National Cerebral and Cardiovascular Center, Osaka, Japan.

Yamamoto, Wataru. Department of the Control for Rheumatic Diseases, Graduate School of Medicine, Kyoto University, Kyoto, Japan; Department of Health Information Management, Kurashiki Sweet Hospital, Kurashiki, Japan.

Matsuo, Takashi. Department of Rheumatology and Clinical Immunology, Graduate School of Medicine, Kyoto University, Kyoto, Japan.

Mori, Masato. Department of Rheumatology and Clinical Immunology, Graduate School of Medicine, Kyoto University, Kyoto, Japan.

Ohmura, Koichiro. Department of Rheumatology and Clinical Immunology, Graduate School of Medicine, Kyoto University, Kyoto, Japan.

Kawabata, Hiroshi. Department of Hematology and Oncology, Graduate School of Medicine, Kyoto University, Kyoto, Japan.

Mimori, Tsuneyo. Department of Rheumatology and Clinical Immunology, Graduate School of Medicine, Kyoto University, Kyoto, Japan.

Abstract

OBJECTIVE: To compare the effect of tocilizumab (TCZ) with other biologic therapies in improving anemia of rheumatoid arthritis (RA) patients.

METHODS: We compared the change of hemoglobin (Hb) levels in a cohort of 147 consecutive RA patients who were treated with biologics for more than 12 weeks. Twenty eight patients were treated with TCZ, and 119 patients were treated with biologics other than TCZ (87 with TNF inhibitors and 32 with abatacept). The change of Hb levels from baseline to week 12 was compared between the TCZ and the non-TCZ groups. We performed univariate and multivariate analyses with adjustment of potential confounders such as baseline characteristics, concomitant treatment, and the clinical response to treatment.

RESULTS: Hb levels generally increased after biologic therapies both in the TCZ and the non-TCZ groups. The increase of Hb levels was greater in the TCZ group than in the non-TCZ groups (1.1 g/dL in the TCZ group vs 0.3 g/dL in the non-TCZ group, p = 0.009). Univariate analysis

revealed that increase of Hb levels was also significantly associated with lower Hb, higher Low Hemoglobin Density, and higher CRP levels at baseline and greater reduction in the clinical disease activity index. TCZ therapy was significantly associated with the increase of Hb levels even after adjustment for these factors by multivariate analysis (p<0.001, effect size 0.08-0.12).

CONCLUSION: TCZ therapy is an independent factor associated with the increase of Hb level after biologic therapies in RA patients. It will help in selecting appropriate biologics for RA patients with anemia.

Publication Type

Journal Article.

Year of Publication

2014

<371>

Unique Identifier

24868914

Title

[Proteolytic activity of IgG-antibodies of mice, immunized by calf thymus histones]. [Ukrainian]

Source

Ukrainian Biochemical Journal. 86(2):79-88, 2014 Mar-Apr.

VI 1

Status

MEDLINE

Authors

Kit IuIa; Kornii N; Kril' II; Mahorivs'ka IB; Tkachenko V; Bilyi RO; Stoika RS.

Authors Full Name

Kit, Iu Ia; Kornii, N; Kril', I I; Mahorivs'ka, I B; Tkachenko, V; Bilyi, R O; Stoika, R S.

Abstract

The main goal of the study was to determine the ability of histones to induce production of the proteolytically active IgG-antibodies in BALB/c mice. In order to perform this study 8 mice were immunized with the fraction of total calf thymus histones. IgGs were isolated from the serum of the immunized and not immunized animals by means of precipitation with 33% ammonium sulfate, followed by affinity chromatography on protein G-Sepharose column. Histones, myelin

basic protein (MBP), lysozyme, BSA, ovalbumin, macroglobulin, casein and cytochrome c served as substrates for determining the proteolytic activity. It was found that IgGs from the blood serum of immunized mice are capable of hydrolyzing histone H1, core histone and MBP. On the contrary, the proteolytic activity of IgGs from the blood serum of not immunized mice was not detected. The absence of proteolytical enzymes in the fraction of IgGs was proven by HPLC chromatography. High levels of proteolytic activity toward histones have been also detected in affinity purified IgGs from blood serum of patients with rheumatoid arthritis, but not in healthy donors. These data indicate that eukaryotic histones may induce production of protabzymes in mammals. The possible origin of these protabzymes and their potential biological role in mammalians is discussed.

Publication Type

English Abstract. Journal Article. Research Support, Non-U.S. Gov't.

Year of Publication

2014

<372>

Unique Identifier

24752013

Title

Immunogenicity of anti-tumour necrosis factor therapy in Korean patients with rheumatoid arthritis and ankylosing spondylitis.

Source

International Immunopharmacology. 21(1):20-5, 2014 Jul.

VI 1

Status

MEDLINE

Authors

Jung SM; Kim HS; Kim HR; Kim NY; Lee JH; Kim J; Kwok SK; Park KS; Park SH; Kim HY; Ju JH.

Authors Full Name

Jung, Seung Min; Kim, Hyun-Sook; Kim, Hae-Rim; Kim, Na Young; Lee, Jung-Hwa; Kim, Juryun; Kwok, Seung-Ki; Park, Kyung-Su; Park, Sung-Hwan; Kim, Ho-Youn; Ju, Ji Hyeon.

Institution

Jung, Seung Min. Division of Rheumatology, Department of Internal Medicine, The Catholic University of Korea, Seoul St. Mary's Hospital, Seoul, South Korea.
Kim, Hyun-Sook. Division of Rheumatology, Department of Internal Medicine, Soonchunhyang University, College of Medicine, Seoul, South Korea.
Kim, Hae-Rim. Division of Rheumatology, Department of Internal Medicine, Konkuk University Medical Center, Seoul, South Korea.
Kim, Na Young. Division of Rheumatology, Department of Internal Medicine, The Catholic University of Korea, Seoul St. Mary's Hospital, Seoul, South Korea.
Lee, Jung-Hwa. Division of Rheumatology, Department of Internal Medicine, Konkuk University Medical Center, Seoul, South Korea.
Kim, Juryun. CiSTEM Laboratory, Convergent Research Consortium for Immunologic Disease, The Catholic University of Korea, Seoul St. Mary's Hospital, Seoul, South Korea.
Kwok, Seung-Ki. Division of Rheumatology, Department of Internal Medicine, The Catholic University of Korea, Seoul St. Mary's Hospital, Seoul, South Korea.
Park, Kyung-Su. Division of Rheumatology, Department of Internal Medicine, The Catholic University of Korea, Seoul St. Mary's Hospital, Seoul, South Korea.
Park, Sung-Hwan. Division of Rheumatology, Department of Internal Medicine, The Catholic University of Korea, Seoul St. Mary's Hospital, Seoul, South Korea.
Kim, Ho-Youn. Division of Rheumatology, Department of Internal Medicine, Konkuk University Medical Center, Seoul, South Korea.
Ju, Ji Hyeon. Division of Rheumatology, Department of Internal Medicine, The Catholic University of Korea, Seoul St. Mary's Hospital, Seoul, South Korea; CiSTEM Laboratory, Convergent Research Consortium for Immunologic Disease, The Catholic University of Korea, Seoul St. Mary's Hospital, Seoul, South Korea. Electronic address: juji@catholic.ac.kr.
Abstract
The aim of this study was to investigate the prevalence of antidrug antibodies (ADAs) against tumour necrosis factor (TNF) inhibitors in patients with rheumatoid arthritis (RA) and ankylosing spondylitis (AS). ADAs were detected in 18 (9.8%) patients with RA and in 18 (10.2%) patients with AS of the 360 patients. Development of ADAs was significantly associated with treatment failure in RA patients (P=0.003). When classified by drugs, the prevalence of immunogenicity in descending order was 17 (28.8%) patients treated with infliximab, 17 (10.4%) with adalimumab, and 2 (1.4%) with etanercept. After adjustment for disease and duration of anti-TNF therapy, the odds ratio as a reference of adalimumab-treated patients was 9.159 (95% confidence interval [CI] 2.005-41.845) for infliximab and 0.280 (95% CI 0.128-0.611) for etanercept. The immunogenicity of anti-TNF therapy was highest in the infliximab-treated group and significantly lower in the etanercept-treated group. Copyright © 2014 Elsevier B.V. All rights reserved.
Publication Type

Comparative Study. Journal Article. Research Support, Non-U.S. Gov't.
Year of Publication
2014

<373>
Unique Identifier
24666108
Title
Lessons from helminth infections: ES-62 highlights new interventional approaches in rheumatoid arthritis. [Review]
Source
Clinical & Experimental Immunology. 177(1):13-23, 2014 Jul.
VI 1
Status
MEDLINE
Authors
Pineda MA; Al-Riyami L; Harnett W; Harnett MM.
Authors Full Name
Pineda, M A; Al-Riyami, L; Harnett, W; Harnett, M M.
Institution
Pineda, M A. Institute of Infection, Immunity and Inflammation, University of Glasgow, Glasgow, UK.
Abstract
Parasitic worms are able to survive in their mammalian host for many years due to their ability to manipulate the immune response by secreting immunomodulatory products. It is increasingly clear that, reflecting the anti-inflammatory actions of such worm-derived immunomodulators, there is an inverse correlation between helminth infection and autoimmune diseases in the developing world. As the decrease in helminth infections due to increased sanitation has correlated with an alarming increase in prevalence of such disorders in industrialized countries, this 'hygiene hypothesis' has led to the proposal that worms and their secreted products offer a novel platform for the development of safe and effective strategies for the treatment of autoimmune disorders. In this study we review the anti-inflammatory effects of one such immunomodulator, ES-62 on innate and adaptive immune responses and the mechanisms it

exploits to afford protection in the murine collagen-induced arthritis (CIA) model of rheumatoid arthritis (RA). As its core mechanism involves targeting of interleukin (IL)-17 responses, which despite being pathogenic in RA are important for combating infection, we discuss how its selective targeting of IL-17 production by T helper type 17 (Th17) and gammadelta T cells, while leaving that of CD49b(+) natural killer (NK and NK T) cells intact, reflects the ability of helminths to modulate the immune system without immunocompromising the host. Exploiting helminth immunomodulatory mechanisms therefore offers the potential for safer therapies than current biologicals, such as 'IL-17 blockers', that are not able to discriminate sources of IL-17 and hence present adverse effects that limit their therapeutic potential. Copyright © 2013 The Authors. Clinical & Experimental Immunology published by John Wiley & Sons Ltd on behalf of British Society for Immunology.

Publication Type

Journal Article. Research Support, Non-U.S. Gov't. Review.

Year of Publication

2014

<374>

Unique Identifier

24665841

Title

Rapid-onset clinical and mechanistic effects of anti-C5aR treatment in the mouse collagen-induced arthritis model.

Source

Clinical & Experimental Immunology. 177(1):219-33, 2014 Jul.

VI 1

Status

MEDLINE

Authors

Andersson C; Wenander CS; Usher PA; Hebsgaard JB; Sondergaard BC; Rono B; Mackay C; Friedrichsen B; Chang C; Tang R; Hornum L.

Authors Full Name

Andersson, C; Wenander, C S; Usher, P A; Hebsgaard, J B; Sondergaard, B-C; Rono, B; Mackay, C; Friedrichsen, B; Chang, C; Tang, R; Hornum, L.

Institution

Andersson, C. Inflammatory Arthritis, Immunopharmacology, Novo Nordisk A/S, Malov, Denmark.

Abstract

Preclinical evidence supports targeting the C5a receptor (C5aR) in rheumatoid arthritis (RA). To support ongoing clinical development of an anti-C5aR monoclonal antibody, we have investigated for the first time the mechanism of action and the pharmacodynamics of a blocking anti-murine C5aR (anti-mC5aR) surrogate antibody in mouse collagen-induced arthritis (CIA). First, efficacy was demonstrated in a multiple-dose treatment study. Almost complete inhibition of clinical disease progression was obtained, including reduced bone and cartilage destruction in anti-mC5aR-treated mice. Then, the mechanism of action was examined by looking for early effects of anti-mC5aR treatment in single-dose treatment studies. We found that 48 h after single-dose treatment with anti-mC5aR, the neutrophil and macrophage infiltration into the paws was already reduced. In addition, several inflammatory markers, including tumour necrosis factor (TNF)-alpha, interleukin (IL)-6 and IL-17A were reduced locally in the paws, indicating reduction of local inflammation. Furthermore, dose-setting experiments supported a beneficial clinical effect of dosing above the C5aR saturation level. In conclusion, these preclinical data demonstrated rapid onset effects of antibody blockade of C5aR. The data have translational value in supporting the Novo Nordisk clinical trials of an anti-C5aR antibody in rheumatoid arthritis patients, by identifying potential biomarkers of treatment effects as well as by providing information on pharmacodynamics and novel insights into the mechanism of action of monoclonal antibody blockade of C5aR. Copyright © 2014 British Society for Immunology.

Publication Type

Journal Article.

Year of Publication

2014

<375>

Unique Identifier

24655394

Title

Infection risk in rheumatoid arthritis and spondyloarthropathy patients under treatment with DMARDs, corticosteroids and TNF-alpha antagonists.

Source
  Journal of Translational Medicine. 12:77, 2014 Mar 22.
VI 1
Status
  MEDLINE
Authors
  Germano V; Cattaruzza MS; Osborn J; Tarantino A; Di Rosa R; Salemi S; D'Amelio R.
Authors Full Name
  Germano, Valentina; Cattaruzza, Maria Sofia; Osborn, John; Tarantino, Aurora; Di Rosa, Roberta; Salemi, Simonetta; D'Amelio, Raffaele.
Institution
  D'Amelio, Raffaele. Department of Clinical and Molecular Medicine, "Sapienza" University of Rome, S, Andrea University Hospital, Via di Grottarossa 1039, 00189 Rome, Italy. raffaele.damelio@uniroma1.it.
Abstract
  BACKGROUND: Infections which complicate rheumatic diseases such as Rheumatoid Arthritis (RA) and Spondyloarthropathy (SpA) (Psoriatic Arthritis [PA] and Ankylosing Spondylitis [AS]), may cause significant morbidity and mortality. However, among the studies on the incidence rate (IR) of infections in such patients, very few have involved controls and the results have been controversial, probably due to methodological difficulties.To estimate infection rates in RA and SpA patients under disease-modifying anti-rheumatic drugs (DMARDs), corticosteroids (CS) and tumor necrosis factor (TNF)alpha antagonists, alone or combined, a single-centre retrospective observational cohort study has been performed.

   PATIENTS AND METHODS: Incidence rates/100 patient-years of any infections were evaluated in RA and SpA outpatients observed in the period November 1, 2003 through December 31, 2009 and stratified according to therapy. Infection incidence rate ratios (IRR) were calculated using Poisson regression models which adjusted for demographic/clinical characteristics of the patients.

   RESULTS: Three hundred and thirtyone infections [318 (96.1%) non-serious and 13 (3.9%) serious] have been registered among 176 of the 341 patients (52%). The IR/100 patient-years of all infections was 36.3 ranging from 12.4 (DMARDs + CS) to 62.7 (anti-TNFalpha + CS). The most frequent infection site was respiratory tract, and bacteria were responsible for three quarters of all infections. In the multivariate analysis, adding anti-TNFalpha to DMARDs doubled the IRR compared to DMARDs alone, anti-TNFalpha + CS significantly tripled it, whereas anti-TNFalpha + CS + DMARDs only increased the risk 2.5 times. The degree of disease activity was strongly and

significantly associated with the infection risk (severe or moderate versus mild, IRR = 4). Female sex was significantly associated with increased infection risk, while duration of disease and anti-influenza vaccination were protective, the latter even for cutaneous/soft-tissue (mainly herpetic) infections.

CONCLUSION: The combination anti-TNFalpha with CS was found to be the most pro-infective treatment, whereas DMARDs alone were relatively safe. Physicians, therefore, should be aware that there may be an increased risk of infection when using anti-TNFalpha and CS therapy together. Anti-influenza vaccination appears to provide broad protection, adding evidence to support its use in these patients, and deserves further study.

Publication Type

Journal Article.

Year of Publication

2014

<376>

Unique Identifier

24646084

Title

Immunization with vaccines and Sjogren's syndrome.

Source

Expert Review of Clinical Immunology. 10(4):429-35, 2014 Apr.

VI 1

Status

MEDLINE

Authors

Soriano A; Afeltra A; Shoenfeld Y.

Authors Full Name

Soriano, Alessandra; Afeltra, Antonella; Shoenfeld, Yehuda.

Institution

Soriano, Alessandra. Zabludowicz Center for Autoimmune Diseases, Sheba Medical Center, Tel-Hashomer, Israel.

Abstract

Sjogren's syndrome (SjS) is a systemic autoimmune disease with complex pathogenesis and still unknown etiology. Infections are listed among the main environmental factors triggering the disease in genetically predisposed individuals. Among other environmental factors, the role of immunization with vaccines in the etiopathogenesis of SjS has not yet been elucidated. Although immunization with vaccines is safe for the majority of subjects, in rare cases it can trigger or exacerbate autoimmune and rheumatic inflammatory conditions. In this paper we investigate the possible links between immunization with vaccines and the pathogenesis of SjS. The current scientific evidence about safety and efficacy of vaccines in the course of SjS are also reviewed.

Publication Type

Journal Article.

Year of Publication

2014

<377>

Unique Identifier

24633313

Title

Immune responses induced by T-cell vaccination in patients with rheumatoid arthritis.

Source

Human vaccines & Immunotherapeutics. 10(5):1221-7, 2014.

VI 1

Status

MEDLINE

Authors

Ivanova I; Seledtsova G; Mamaev S; Shishkov A; Seledtsov V.

Authors Full Name

Ivanova, Irina; Seledtsova, Galina; Mamaev, Sergey; Shishkov, Alexey; Seledtsov, Viktor.

Institution

Ivanova, Irina. The Laboratory of Cellular Biotechnologies; State Research Institute for Clinical Immunology; Novosibirsk, Russia.

Seledtsova, Galina. The Laboratory of Cellular Biotechnologies; State Research Institute for Clinical Immunology; Novosibirsk, Russia.

Mamaev, Sergey. Federal State-funded Health Care Institution Clinical Hospital No 85; Russian Federal Medical and Biological Agency; Moscow, Russia.
Shishkov, Alexey. The Laboratory of Cellular Biotechnologies; State Research Institute for Clinical Immunology; Novosibirsk, Russia.
Seledtsov, Viktor. Research and Implementation Centre for Medical Biotechnologies; Immanuel Kant Baltic Federal University; Kaliningrad, Russia.
Abstract
Patients with rheumatoid arthritis (RA) were treated with a cellular vaccine, which consisted of autologous collagen-reactive T-cells. This study showed that antigen-specific proliferative activity of the peripheral blood mononuclear cells was significantly downregulated after T-cell vaccination in RA patients. T-cell vaccination resulted in a statistically significant decrease in plasma IFNgamma levels and a concomitant increase in IL-4 levels in treated patients. Accordingly, following T-cell vaccination the number of IFNgamma-producing CD4(+) and CD8(+) T-cells was decreased by 1.6-1.8-fold, which was paralleled by 1.7-fold increases in IL-4-producing CD4(+) T-cells. In addition, the present study showed 5-7-fold increase in the CD8(+)CD45RO(+)CD62L(-) effector memory T-cells and central memory T-cells (both CD4(+) CD45RO(+)CD62L(+) T-cells and CD8(+)CD45RO(+)CD62L(+) T-cells) in RA patients, as compared with healthy individuals. We observed significant reduction in CD4(+) and CD8(+) central memory T-cells, as well as reduction in CD8(+) effector memory T-cells in vaccinated patients in the course of the treatment. We also demonstrated that CD4(+)CD25(+)FoxP3(+) regulatory T-cell levels were significantly up-regulated in the peripheral blood of RA patients following T-cell vaccination. However, CD4(+)CD25(-)FoxP3(+) T-cell levels did not significantly change during the entire T-cell vaccination course. In conclusion, the T-cell immunotherapy regimen used resulted in the clinical improvement, which was achieved in 87% patients.
Publication Type
Clinical Trial. Journal Article. Research Support, Non-U.S. Gov't.
Year of Publication
2014

<378>
Unique Identifier
24626168
Title

Regulatory T-cell vaccination independent of auto-antigen. [Review]

Source

Experimental & Molecular Medicine. 46:e82, 2014 Mar 14.

VI 1

Status

MEDLINE

Authors

Pascual DW; Yang X; Holderness K; Jun S; Maddaloni M; Kochetkova I.

Authors Full Name

Pascual, David W; Yang, Xinghong; Holderness, Kathryn; Jun, SangMu; Maddaloni, Massimo; Kochetkova, Irina.

Institution

Pascual, David W. Department of Infectious Diseases and Pathology, College of Veterinary Medicine, University of Florida, Gainesville, FL, USA.

Yang, Xinghong. Department of Infectious Diseases and Pathology, College of Veterinary Medicine, University of Florida, Gainesville, FL, USA.

Holderness, Kathryn. Department of Immunology and Infectious Diseases, Montana State University, Bozeman, MT, USA.

Jun, SangMu. Department of Infectious Diseases and Pathology, College of Veterinary Medicine, University of Florida, Gainesville, FL, USA.

Maddaloni, Massimo. Department of Infectious Diseases and Pathology, College of Veterinary Medicine, University of Florida, Gainesville, FL, USA.

Kochetkova, Irina. Department of Immunology and Infectious Diseases, Montana State University, Bozeman, MT, USA.

Abstract

To date, efforts to treat autoimmune diseases have primarily focused on the disease symptoms rather than on the cause of the disease. In large part, this is attributed to not knowing the responsible auto-antigens (auto-Ags) for driving the self-reactivity coupled with the poor success of treating autoimmune diseases using oral tolerance methods. Nonetheless, if tolerogenic approaches or methods that stimulate regulatory T (Treg) cells can be devised, these could subdue autoimmune diseases. To forward such efforts, our approach with colonization factor antigen I (CFA/I) fimbriae is to establish bystander immunity to ultimately drive the development of auto-Ag-specific Treg cells. Using an attenuated Salmonella vaccine expressing CFA/I fimbriae, fimbriae-specific Treg cells were induced without compromising the vaccine's capacity to protect against travelers' diarrhea or salmonellosis. By adapting the vaccine's anti-inflammatory properties, it was found that it could also dampen experimental inflammatory diseases resembling multiple sclerosis (MS) and rheumatoid arthritis. Because of this bystander effect, disease-

specific Treg cells are eventually induced to resolve disease. Interestingly, this same vaccine could elicit the required Treg cell subset for each disease. For MS-like disease, conventional CD25(+) Treg cells are stimulated, but for arthritis CD39(+) Treg cells are induced instead. This review article will examine the potential of treating autoimmune diseases without having previous knowledge of the auto-Ag using an innocuous antigen to stimulate Treg cells via the production of transforming growth factor-beta and interleukin-10.

Publication Type

Journal Article. Research Support, N.I.H., Extramural. Review.

Year of Publication

2014

<379>

Unique Identifier

24624676

Title

[Pain management and Hypnosis]. [French]

Source

Revue Medicale Suisse. 10(415):252-3, 2014 Jan 29.

VI 1

Status

MEDLINE

Authors

Schlegel-Christen S.

Authors Full Name

Schlegel-Christen, Simone.

Publication Type

Case Reports. Journal Article.

Year of Publication

2014

<380>
Unique Identifier
24584918
Title
Vaccine responses in patients with rheumatoid arthritis treated with certolizumab pegol: results from a single-blind randomized phase IV trial.
Source
Journal of Rheumatology. 41(4):648-57, 2014 Apr.
VI 1
Status
MEDLINE
Authors
Kivitz AJ; Schechtman J; Texter M; Fichtner A; de Longueville M; Chartash EK.
Authors Full Name
Kivitz, Alan J; Schechtman, Joy; Texter, Michele; Fichtner, Andreas; de Longueville, Marc; Chartash, Elliot K.
Institution
Kivitz, Alan J. From the Altoona Center for Clinical Research, Duncansville, PA; Sun Valley Arthritis Center, Peoria, AZ; UCB Pharma, Smyrna, GA, USA; UCB Pharma, Monheim, Germany; UCB Pharma, Brussels, Belgium.
Comments
Comment in (CIN)
Abstract
OBJECTIVE: To evaluate the humoral immune response to pneumococcal and influenza vaccination in adults with rheumatoid arthritis (RA) receiving certolizumab pegol (CZP).

METHODS: In this 6-week, single-blind, placebo-controlled trial with optional 6-month open-label extension (NCT00993668), patients were stratified by concomitant methotrexate (MTX) use and randomized to receive CZP 400 mg (loading dose; according to CZP label) or placebo at weeks 0, 2, and 4. Pneumococcal (polysaccharide 23) and influenza vaccines were administered at Week 2. Satisfactory humoral immune response, defined as >=2-fold titer increase in >=3 of 6 pneumococcal antigens and >=4-fold titer increase in >=2 of 3 influenza antigens, were assessed independently 4 weeks after vaccination.

RESULTS: Following pneumococcal vaccination, 62.5% of placebo patients and 54.5% of CZP patients without effective titers at baseline achieved a humoral response (difference in

proportions was -8.0 percentage points; 95% CI -22.5 to 6.6%). Following influenza vaccination, 61.4% of placebo and 53.5% of CZP patients without effective titers at baseline achieved a humoral response (difference in proportions: -8.0 percentage points; 95% CI -22.9 to 7.0%). In all patients, including those with effective titers at baseline, 58.2% of placebo and 53.3% of CZP patients developed satisfactory pneumococcal titers, and 54.1% of placebo and 50.5% of CZP patients developed satisfactory influenza antibody titers. Vaccine responses to pneumococcal and influenza antigens were reduced similarly in both treatment groups with concomitant MTX use.

CONCLUSION: Humoral immune responses to pneumococcal and influenza vaccination are not impaired when given during the loading phase of CZP treatment in patients with RA. (ClinicalTrials.gov NCT00993668).

Publication Type

Clinical Trial, Phase IV. Journal Article. Randomized Controlled Trial. Research Support, Non-U.S. Gov't.

Year of Publication

2014

<381>

Unique Identifier

24581098

Title

Preventing rheumatic fever: M-protein based vaccine. [Review]

Source

Indian Heart Journal. 66(1):64-7, 2014 Jan-Feb.

VI 1

Status

MEDLINE

Authors

Tandon R.

Authors Full Name

Tandon, Rajendra.

Institution

Tandon, Rajendra. Consultant Cardiologist, Sitaram Bhartia Institute of Science & Research, B-16, Mehrauli Institutional Area, New Delhi 110 016, India. Electronic address: dr_rajtandon@yahoo.com.
Abstract
Group A beta hemolytic streptococcus (GAS), the organism which initiates rheumatic fever (RF) continues to be sensitive to penicillin. However, penicillin cannot prevent RF if the preceding sore throat is asymptomatic in more than 70 percent children. Prevention of rheumatic fever (RF) may be possible only with the use of a vaccine. Efforts to design a vaccine based on emm gene identification of GAS, M-protein going on for more than 40 years, is unlikely to succeed. M-protein is strain specific. Infection with one strain does not provide immunity from infection with another strain. Based on the emm gene identification, of 250 or more identified strains of GAS, the distribution is heterogenous and keeps changing. The M-protein gene sequence of the organism tends to mutate. A vaccine prepared from available strains may not be effective against a strain following mutation. Lethal toxic shock syndrome due to GAS infection has been described with organisms without identifiable or functional M-protein. M-protein has been excluded as the antigen responsible for acute glomerulonephritis (GN). Therefore M-protein plays no role in one suppurative (toxic shock syndrome) and one non-suppurative (acute GN) manifestation due to GAS infection. Lastly there is no direct evidence to indicate that M-protein is involved in inducing RF. The role of M-protein and the GAS component resulting in the suppurative manifestations of GAS infections like pyoderma, septic arthritis or necrotizing fasciitis etc is unknown. For a vaccine to be effective, an epitope of the streptococcus which is stable and uniformly present in all strains, needs to be identified and tested for its safety and efficacy. The vaccine if and when available is expected to prevent GAS infection. Preventing GAS infection will prevent all the suppurative as well as non-suppurative manifestations including RF. Copyright © 2013 Cardiological Society of India. Published by Elsevier B.V. All rights reserved.
Publication Type
Journal Article. Review.
Year of Publication
2014

<382>
Unique Identifier
24535556

Title

Emerging immunotherapies for rheumatoid arthritis. [Review]

Source

Human vaccines & Immunotherapeutics. 10(4):822-37, 2014.

VI 1

Status

MEDLINE

Authors

Reynolds G; Cooles FA; Isaacs JD; Hilkens CM.

Authors Full Name

Reynolds, Gary; Cooles, Faye A H; Isaacs, John D; Hilkens, Catharien M U.

Institution

Reynolds, Gary. Institute of Cellular Medicine; Musculoskeletal Research Group; Newcastle University; Newcastle upon Tyne, Tyne and Wear UK.

Cooles, Faye A H. Institute of Cellular Medicine; Musculoskeletal Research Group; Newcastle University; Newcastle upon Tyne, Tyne and Wear UK.

Isaacs, John D. Institute of Cellular Medicine; Musculoskeletal Research Group; Newcastle University; Newcastle upon Tyne, Tyne & Wear UK.

Hilkens, Catharien M U. Institute of Cellular Medicine; Musculoskeletal Research Group; Newcastle University; Newcastle upon Tyne, Tyne & Wear UK.

Abstract

Novel treatments in development for rheumatoid arthritis target 3 broad areas: cytokines, cells, and signaling pathways. Therapies from each domain share common advantages (for example previously demonstrated efficacy, potential long-term immunomodulation, and oral administration respectively) that have stimulated research in each area but also common obstacles to their development. In this review recent progress in each area will be discussed alongside the factors that have impeded their path to clinical use.

Publication Type

Journal Article. Review.

Year of Publication

2014

<383>

Unique Identifier

24445477

Title

Mechanisms of autoimmunity in human diseases: a critical review of current dogma. [Review]

Source

Current Opinion in Rheumatology. 26(2):197-203, 2014 Mar.

VI 1

Status

MEDLINE

Authors

Benson RA; Brewer JM; Platt AM.

Authors Full Name

Benson, Robert A; Brewer, James M; Platt, Andrew M.

Institution

Benson, Robert A. Institute of Infection, Immunity and Inflammation, College of Medical, Veterinary & Life Sciences, Sir Graeme Davies Building, University of Glasgow, Glasgow, UK.

Abstract

PURPOSE OF REVIEW: Autoimmune diseases such as rheumatoid arthritis (RA) pose an increasing, worldwide economic and health burden. Significantly, no cure exists for the majority of autoimmune diseases and consequently treatment is largely aimed at controlling disease symptoms. Therefore, there exists a critical need to develop new approaches that directly address the cause of disease, leading to disease remission and ultimately cure.

RECENT FINDINGS: The organs, cells and molecules involved in the breach of self-tolerance have been partially defined in experimental models of autoimmunity. However, the broad applicability of this dogma in clinical disease is only partially understood. This gap between analyses of established disease and investigating early disease pathogenesis argues for the need for complementary studies in mice and humans.

SUMMARY: Through a combination of clinical and experimental systems, novel autoantigens and neoepitopes involved in RA have been revealed. These have clear utility in predisease diagnosis and offer the possibility of antigen-specific immunotherapy. Ongoing experimental and clinical studies, for example using dendritic cell transfer, will facilitate a clearer understanding of the molecules, cells and organs that should be targeted to reinstate immunological tolerance. Antigen-specific immunotherapy therefore offers disease intervention without broad immunosuppression, and most importantly increases the likelihood of achieving true disease remission and cure.

Publication Type
  Journal Article. Review.
Year of Publication
  2014

<384>
Unique Identifier
  24405551
Title
  Characterization of T cell phenotype and function in a double transgenic (collagen-specific TCR/HLA-DR1) humanized model of arthritis.
Source
  Arthritis Research & Therapy. 16(1):R7, 2014 Jan 10.
VI 1
Status
  MEDLINE
Authors
  Tang B; Kim S; Hammond S; Cullins DL; Brand DD; Rosloniec EF; Stuart JM; Postlethwaite AE; Kang AH; Myers LK.
Authors Full Name
  Tang, Bo; Kim, Seunghyun; Hammond, Sarah; Cullins, David L; Brand, David D; Rosloniec, Edward F; Stuart, John M; Postlethwaite, Arnold E; Kang, Andrew H; Myers, Linda K.
Abstract
  INTRODUCTION: T cells orchestrate joint inflammation in rheumatoid arthritis (RA), yet they are difficult to study due to the small numbers of antigen-specific cells. The goal of this study was to characterize a new humanized model of autoimmune arthritis and to describe the phenotypic and functional changes that occur in autoimmune T cells following the induction of pathological events.

   METHODS: We developed a double transgenic mouse containing both the HLA-DR1 transgene and an HLA-DR1-restricted collagen-specific TCR in order to obtain large numbers of antigen-specific T cells that can be used for immunologic studies.

RESULTS: In vitro, CII-specific T cells from this mouse proliferated vigorously in response to the CII immunodominant peptide A2 and the cells altered their phenotype to become predominately CD62Llow and CD44high "activated" T cells. The response was accompanied by the production of Th1, Th2, and Th17-type cytokines. Following immunization with bovine CII/CFA, these mice develop an accelerated arthritis compared to single transgenic HLA-DR1 mice. On the other hand, when the mice were treated orally with the analog peptide A12, (a suppressive analog of collagen we have previously described), arthritis was significantly suppressed, despite the fact that >90% of the CD4+ T cells express the TCR Tg. In GALT tissues taken from the A12-treated mice, IL-2, IFN-gamma, and IL-17 production to the autoimmune collagen determinant dropped while high levels of IL-10 and IL-4 were produced.

CONCLUSIONS: We have developed a humanized model of autoimmune arthritis that will be useful for the study of T cell directed therapies as well as T cell mediated mechanisms of autoimmune diseases.

Publication Type

Journal Article. Research Support, N.I.H., Extramural. Research Support, U.S. Gov't, Non-P.H.S..

Year of Publication

2014

<385>

Unique Identifier

24405357

Title

Immunological characteristics and T-cell receptor clonal diversity in children with systemic juvenile idiopathic arthritis undergoing T-cell-depleted autologous stem cell transplantation.

Source

Immunology. 142(2):227-36, 2014 Jun.

VI 1

Status

MEDLINE

Authors

Wu Q; Pesenacker AM; Stansfield A; King D; Barge D; Foster HE; Abinun M; Wedderburn LR.

Authors Full Name

Wu, Qiong; Pesenacker, Anne M; Stansfield, Alka; King, Douglas; Barge, Dawn; Foster, Helen E; Abinun, Mario; Wedderburn, Lucy R.

Institution

Wu, Qiong. Rheumatology Unit, UCL Institute of Child Health, University College London, London, UK.

Abstract

Children with systemic Juvenile Idiopathic Arthritis (sJIA), the most severe subtype of JIA, are at risk from destructive polyarthritis and growth failure, and corticosteroids as part of conventional treatment can result in osteoporosis and growth delay. In children where there is failure or toxicity from drug therapies, disease has been successfully controlled by T-cell-depleted autologous stem cell transplantation (ASCT). At present, the immunological basis underlying remission after ASCT is unknown. Immune reconstitution of T cells, B cells, natural killer cells, natural killer T cells and monocytes, in parallel with T-cell receptor (TCR) diversity by analysis of the beta variable region (TCRVb) complementarity determining region-3 (CDR3) using spectratyping and sequencing, were studied in five children with sJIA before and after ASCT. At time of follow up (mean 11.5 years), four patients remain in complete remission, while one child relapsed within 1 month of transplant. The CD8(+) TCRVb repertoire was highly oligoclonal early in immune reconstitution and re-emergence of pre-transplant TCRVb CDR3 dominant peaks was observed after transplant in certain TCRVb families. Further, re-emergence of pre-ASCT clonal sequences in addition to new sequences was identified after transplant. These results suggest that a chimeric TCR repertoire, comprising T-cell clones developed before and after transplant, can be associated with clinical remission from severe arthritis. Copyright © 2014 The Authors. Immunology published by John Wiley & Sons Ltd.

Publication Type

Journal Article. Research Support, Non-U.S. Gov't.

Year of Publication

2014

<386>

Unique Identifier

24397962

Title

The role of M3 muscarinic acetylcholine receptor reactive T cells in Sjogren's syndrome: a critical review. [Review]

Source

Journal of Autoimmunity. 51:44-50, 2014 Jun.

VI 1

Status

MEDLINE

Authors

Sumida T; Tsuboi H; Iizuka M; Hirota T; Asashima H; Matsumoto I.

Authors Full Name

Sumida, Takayuki; Tsuboi, Hiroto; Iizuka, Mana; Hirota, Tomoya; Asashima, Hiromitsu; Matsumoto, Isao.

Institution

Sumida, Takayuki. Department of Internal Medicine, University of Tsukuba, 1-1-1 Tennodai, Tsukuba-City, Ibaraki 305-8575, Japan. Electronic address: tsumida@md.tsukuba.ac.jp.

Tsuboi, Hiroto. Department of Internal Medicine, University of Tsukuba, 1-1-1 Tennodai, Tsukuba-City, Ibaraki 305-8575, Japan.

Iizuka, Mana. Department of Internal Medicine, University of Tsukuba, 1-1-1 Tennodai, Tsukuba-City, Ibaraki 305-8575, Japan.

Hirota, Tomoya. Department of Internal Medicine, University of Tsukuba, 1-1-1 Tennodai, Tsukuba-City, Ibaraki 305-8575, Japan.

Asashima, Hiromitsu. Department of Internal Medicine, University of Tsukuba, 1-1-1 Tennodai, Tsukuba-City, Ibaraki 305-8575, Japan.

Matsumoto, Isao. Department of Internal Medicine, University of Tsukuba, 1-1-1 Tennodai, Tsukuba-City, Ibaraki 305-8575, Japan.

Abstract

CD4+ T cells constitute the majority of infiltrating cells in salivary glands and lachrymal glands of patients with Sjogren's syndrome (SS). The pathophysiology of SS involves T cell recognition of antigens through the T cell antigen receptor, which triggers cytokine production and chronic inflammation. The M3 muscarinic acetylcholine receptor (M3R) molecule is expressed in exocrine glands, such as salivary glands and lachrymal glands, and plays an important role in exocrine secretion. Previous studies indicated the presence of M3R reactive T cells in peripheral blood of 40% of patients with SS and autoantibodies against M3R in sera of 9-100% of the same patients. Thus, M3R is considered a candidate receptor for autoantigen recognition by T and B cells. The relationship between B cell epitopes and the function of anti-M3R antibodies has been reported, suggesting the pathogenic role of anti-M3R antibodies in xerostomia commonly seen in SS patients. We generated new experimental mouse model, M3R-induced sialadenitis (MIS), using

Rag1(-/-) mice inoculated with splenocytes from M3R(-/-) mice immunized with M3R synthetic peptides. Mice with MIS developed severe SS-like sialadenitis. Cell transfer experiments using M3R(-/-)xIFNgamma(-/-) mice and M3R(-/-)xIL-17(-/-) mice showed that IFNgamma and IL-17 are key cytokines in the pathogenesis of sialadenitis. These findings indicate the crucial roles of M3R-reactive Th1 and Th17 cells in autoimmune sialadenitis, and suggest that these cells, in addition to anti-M3R antibodies, are potential targets in new treatments for SS. Copyright © 2013 Elsevier Ltd. All rights reserved.

Publication Type

Journal Article. Research Support, Non-U.S. Gov't. Review.

Year of Publication

2014

<387>

Unique Identifier

24389864

Title

Dendritic cells as targets or therapeutics in rheumatic autoimmune disease. [Review]

Source

Current Opinion in Rheumatology. 26(2):211-8, 2014 Mar.

VI 1

Status

MEDLINE

Authors

Thomas R.

Authors Full Name

Thomas, Ranjeny.

Institution

Thomas, Ranjeny. The University of Queensland Diamantina Institute, Translational Research Institute, Princess Alexandra Hospital, Woolloongabba, Queensland, Australia.

Abstract

PURPOSE OF REVIEW: Antigen-specific immunotherapy is a major goal for improvement in the treatment of autoimmune rheumatic disease. Dendritic cells are professional antigen-presenting cells, abundant at mucosal surfaces and in tissues. They also play a critical role in self-tolerance.

This review covers recent advances in the field of dendritic cells as targets or therapeutics in rheumatic autoimmune disease.

RECENT FINDINGS: Key themes include the phenotypic and functional characterization, lineage relationships and transcription factors involved in the development of the various dendritic cell subsets. Phenotype and function of mouse and human subsets has now been much better mapped. Progress in the elucidation of targeting ligands and routes for induction of antigen-specific tolerance using either antigen-antibody fusion constructs or particulate conjugates is described. Various inflammatory molecules made by dendritic cells, including type I interferon, are important therapeutic targets in autoimmune rheumatic diseases. Approaches to block this and clinical trials in this area are discussed.

SUMMARY: There are considerable basic science developments in the field of dendritic cells and tolerance that will speed translation to human of the large amount of knowledge generated in mouse in-vivo systems. Various antigen-specific therapy approaches are in the process of translation to the clinic.
Publication Type
Journal Article. Research Support, Non-U.S. Gov't. Review.
Year of Publication
2014

<388>
Unique Identifier
24365380
Title
The effect of B-cell depletion therapy on serological evidence of B-cell and plasmablast activation in patients with rheumatoid arthritis over multiple cycles of rituximab treatment.
Source
Journal of Autoimmunity. 50:67-76, 2014 May.
VI 1
Status
MEDLINE
Authors

Cambridge G; Perry HC; Nogueira L; Serre G; Parsons HM; De La Torre I; Dickson MC; Leandro MJ; Edwards JC.
Authors Full Name
Cambridge, G; Perry, H C; Nogueira, L; Serre, G; Parsons, H M; De La Torre, I; Dickson, M C; Leandro, M J; Edwards, J C W.
Institution
Cambridge, G. Department of Rheumatology, University College London, London, UK. Electronic address: g.cambridge@ucl.ac.uk.
Perry, H C. Quantitative Sciences, GlaxoSmithKline, Stevenage, UK.
Nogueira, L. Laboratory "Epidermis Differentiation and Rheumatoid Autoimmunity", CNRS, INSERM, Universite de Toulouse & Laboratory of Cell Biology and Cytology, CHU de Toulouse, Toulouse, France.
Serre, G. Laboratory "Epidermis Differentiation and Rheumatoid Autoimmunity", CNRS, INSERM, Universite de Toulouse & Laboratory of Cell Biology and Cytology, CHU de Toulouse, Toulouse, France.
Parsons, H M. Department of Rheumatology, University College London, London, UK.
De La Torre, I. Department of Rheumatology, University College London, London, UK.
Dickson, M C. Immunoinflammation TAU, GlaxoSmithKline, Stevenage, UK.
Leandro, M J. Department of Rheumatology, University College London, London, UK.
Edwards, J C W. Department of Rheumatology, University College London, London, UK.
Abstract
B-cell depletion therapy (BCDT) based on rituximab (RTX) induces clinical remission in a majority of seropositive patients with Rheumatoid arthritis (RA). However, all patients eventually relapse. The aim of this study was to determine whether dynamic changes in combinations of serological measures of B-cell activation were associated over up to three cycles of BCDT. We included only RA patients who gave an adequate clinical response, as measured by DAS28. Twenty three patients were studied over 1 cycle, 21 over 2, and 15 over 3 cycles of BCDT. Serum analytes including isotypes of Rheumatoid factors (RhF) and anti-citrullinated protein/peptide antibodies (ACPA), B-cell activating factor (BAFF), serum free light chains (SFLC), soluble CD23 (sCD23), antibodies to tetanus toxoid (TT) and to pneumococcal capsular polysaccharide (PCP) were measured by ELISA at 4 key points in each cycle, namely: Baseline (pre-RTX in each cycle); when B-cell depleted (CD19+B-cells < 5/mul); at B-cell return (CD19+B-cells >= 5/mul); and at clinical relapse (DELTADAS28 > 1.2). SFLC were used as a measure of plasmablast activity. As sCD23 is cleaved from naive B-cells coincident with attaining CD27 expression, levels were used as a novel measure of maturation of B-cells to CD27+. The most consistent changes between baseline and B-cell depletion within all 3 cycles were in SFLC, sCD23 and IgM-RhF which fell and in BAFF levels which rose. After 3 complete cycles of BCDT, both IgM

autoantibodies and IgG-CCP had decreased, BAFF levels were higher (all p < 0.05); other analytes remained unchanged compared with baseline. Dynamic changes in lambdaSFLC, sCD23, IgM-RhF and BAFF were also consistently associated with relapse in patients with longer clinical responses after B-cell return. Incremental rises in sCD23 levels in cycles 2 and 3 were correlated with time to relapse. Repopulation of the periphery after BCDT is initiated by naive B-cells and precedes relapse. Our study showed that differentiation into plasmablasts, attended by sCD23 and SFLC production and IgM-RhF specificity may be required to precipitate relapse in patients experiencing longer responses after RTX. These studies also provide novel information related to the resumption of autoimmune responses and their association with B-cell kinetics following BCDT. Copyright © 2013 Elsevier Ltd. All rights reserved.
Publication Type
Journal Article.
Year of Publication
2014

<389>
Unique Identifier
24322451
Title
Comparison of national clinical practice guidelines and recommendations on vaccination of adult patients with autoimmune rheumatic diseases. [Review]
Source
Rheumatology International. 34(2):151-63, 2014 Feb.
VI 1
Status
MEDLINE
Authors
Papadopoulou D; Sipsas NV.
Authors Full Name
Papadopoulou, Despoina; Sipsas, Nikolaos V.
Institution

Papadopoulou, Despoina. Pain and Palliative Care Clinic, Aretaieion Hospital and Medical School, National and Kapodistrian University of Athens, Vasilisis Sofias 76, 11528, Athens, Greece, papadopould@med.uoa.gr.
Abstract
The aim of the study is to identify and compare national recommendations on vaccination of adult patients with autoimmune rheumatic diseases (ARDs) in Europe, North America, and Australia. We conducted a search for recommended immunizations in adult patients with ARDs in the Medline database and the Web sites of National Rheumatologic Societies, Ministries of Health, National Advisory Committees on Immunization, and other relevant National Scientific Societies. We compared national guidelines and identified points of agreement and differences. Guidelines on vaccination of adult patients with ARDs were identified in 21 countries. Points of agreement include administering influenza and pneumococcal vaccines in addition to inactivated age-appropriate or travel-related vaccines, and avoiding the use of live vaccines in immunocompromised patients with ARDs. The most important differences concern the steroid dose that induces immunosuppression, the time interval between live vaccines and the initiation of immunosuppressive treatment, herpes zoster vaccination, and the preferred pneumococcal vaccine in patients with ARDs. We observed significant differences among national recommendations on immunizations in patients with ARDs, reflecting the lack of evidence-based data.
Publication Type
Comparative Study. Journal Article. Research Support, Non-U.S. Gov't. Review.
Year of Publication
2014

<390>
Unique Identifier
24288046
Title
Survey about tolerance of the AS03-adjuvanted H1N1 influenza vaccine in children with rheumatic diseases.
Source
Clinical Rheumatology. 33(1):137-9, 2014 Jan.
VI 1

Status

MEDLINE

Authors

Sengler C; Niewerth M; Kallinich T; Nimtz-Talaska A; Haller M; Huppertz HI; Minden K.

Authors Full Name

Sengler, C; Niewerth, M; Kallinich, T; Nimtz-Talaska, A; Haller, M; Huppertz, H-I; Minden, K.

Institution

Sengler, C. Epidemiology Unit, German Rheumatism Research Centre Berlin, Leibniz Institute, Chariteplatz 1, 10117, Berlin, Germany, sengler@drfz.de.

Abstract

The objective of this study is to evaluate complications and changes in health status (disease activity and flare) in response to the AS03-adjuvanted H1N1 vaccine in children with rheumatic diseases. We conducted a nationwide survey addressing paediatric rheumatology sites who participated in the national paediatric rheumatology database. Ninety patients were documented-38 % under treatment with biologicals-of whom 18 % suffered from complications (10 % local and 8 % systemic) with no relevant changes in median disease activity or flare rate during 4 weeks following the vaccination. The adjuvanted H1N1 influenza vaccine seems to be adequately tolerated in children with rheumatic diseases.

Publication Type

Journal Article.

Year of Publication

2014

<391>

Unique Identifier

24264477

Title

Myeloid-derived suppressor cells protect mouse models from autoimmune arthritis via controlling inflammatory response.

Source

Inflammation. 37(3):670-7, 2014 Jun.

VI 1

Status

MEDLINE

Authors

Zhang L; Zhang Z; Zhang H; Wu M; Wang Y.

Authors Full Name

Zhang, Lei; Zhang, Zhengmei; Zhang, Huailiang; Wu, Min; Wang, Yanxia.

Institution

Zhang, Lei. Department of Nephrology, Jinan Military General Hospital, 25 Shifan Road, Jinan, 250031, Shandong, China.

Abstract

Myeloid-derived suppressor cells (MDSCs) have been reported to participate in immune suppression and autoimmune disorders. However, its role in autoimmune arthritis remains to be determined. We explored whether adoptive transfer of MDSCs in vivo would block joint inflammation and histological damage using collagen-induced arthritis (CIA) and antigen-induced arthritis (AIA) models. CD11b(+) Gr-1(+) MDSCs were isolated from the single cells from the spleens of CIA mice on day 41 or AIA mice on day 35. MDSCs (2 x 10(6)) were then transferred to AIA and CIA mice via tail vein before arthritis establishment at indicated time points. Phosphate buffered saline (PBS) was injected as control. Arthritis was evaluated by severity score and histology. The levels of TNF-alpha, IL-6, IL-17 and IL-10 in the serum and joints were detected by enzyme-linked immunosorbent assay (ELISA). The number of Th17 cells and macrophages in draining lymph nodes and joint tissues was assessed by flow cytometric analysis. Adoptive transfer of MDSCs significantly reduced the clinical score of arthritis, alleviated joint inflammation and histological damage both in AIA and CIA models compared with PBS-treated control groups. The levels of TNF-alpha, IL-6, IL-17, and IL-10 in the serum and joints were down-regulated by transfer of MDSCs. In addition, adoptive transfer of MDSCs significantly reduced the number of Th17 cells and macrophages in draining lymph nodes and joint tissues. Altogether, we demonstrate that adoptive transfer of MDSCs prevented autoimmune arthritis in mouse models of RA through inhibiting Th17 cells and macrophages. These new findings provide insights into the inhibitory functions of MDSCs and MDSCs may be used as a cell-based biotherapy in RA.

Publication Type

Journal Article.

Year of Publication

2014

<392>

Unique Identifier

24252023

Title

Immune response to influenza vaccine and pneumococcal polysaccharide vaccine under IL-6 signal inhibition therapy with tocilizumab.

Source

Modern Rheumatology. 24(3):511-6, 2014 May.

VI 1

Status

MEDLINE

Authors

Tsuru T; Terao K; Murakami M; Matsutani T; Suzaki M; Amamoto T; Nakashima H; Akiyama A; Nishimoto N.

Authors Full Name

Tsuru, Tomomi; Terao, Kimio; Murakami, Miho; Matsutani, Takaji; Suzaki, Midori; Amamoto, Toshiaki; Nakashima, Hitoshi; Akiyama, Azusa; Nishimoto, Norihiro.

Institution

Tsuru, Tomomi. Medical Co. LTA PS clinic, Fukuoka, Japan.

Abstract

OBJECTIVES: To evaluate humoral immune response to influenza vaccine and polysaccharide pneumococcal vaccine in patients with rheumatoid arthritis (RA) or Castleman's disease (CD) during tocilizumab therapy.

METHODS: Thirty-eight patients (28 RA and 10 CD) receiving tocilizumab and 39 RA patients receiving TNF inhibitors and/or synthetic DMARDs subcutaneously received a single dose of a split-virion inactivated influenza vaccine containing A(New Caledonia (NC):H1N1), A(Hiroshima (HIR):H3N2) and B(Malaysia (MAL)) strains. Twenty-one RA patients using tocilizumab also received 23-valent polysaccharide pneumococcal vaccine. Antibody titers were measured every 4 weeks for a total of 12 weeks after vaccination.

RESULTS: In the tocilizumab group, seroprotective titers (40-fold or more) were obtained in 36/38(95%) for A(NC), 35/38(92%) for A(HIR) and 32/38(84%) for B(MAL). In the patients with baseline antibody titer < 40-fold, 11/11(100%), 7/8(88%) and 18/20(90%) patients showed four-fold or more increase in the titer from baseline to A(NC), A(HIR) and B(MAL), respectively. Patients using TNF inhibitors and/or DMARDs showed similar responses. Pneumococcal

antibody titers increased at least two-fold in more than 9 of 12 serotypes, which continued for longer than 12 weeks in all the patients.

CONCLUSION: Interleukin-6 (IL-6) blocking therapy with tocilizumab did not affect the humoral immune response to both influenza and pneumococcal vaccines.

Publication Type

Journal Article. Research Support, Non-U.S. Gov't.

Year of Publication

2014

<393>

Unique Identifier

24126901

Title

B cells: depletion or functional modulation in rheumatic diseases. [Review]

Source

Current Opinion in Rheumatology. 26(2):228-36, 2014 Mar.

VI 1

Status

MEDLINE

Authors

Dorner T; Lipsky PE.

Authors Full Name

Dorner, Thomas; Lipsky, Peter E.

Institution

Dorner, Thomas. aDepartment of Rheumatology and Clinical Immunology, CC12 Department of Medicine, Deutsches Rheuma-Forschungszentrum Berlin, Charite - Universitatsmedizin Berlin, Berlin, Germany bNIAMS, NIH, Bethesda, Maryland, USA.

Abstract

PURPOSE OF REVIEW: The availability of drugs directly and indirectly targeting the B cells has refocussed attention on the role of B lymphocytes in rheumatic autoimmune/inflammatory diseases (RAIDs), but their distinct therapeutic potential for certain diseases remains to be further assessed.

RECENT FINDINGS: Although additional drugs are currently in clinical development targeting surface molecules (CD19, CD20, CD22, etc.) and cytokines (IL-6, IL-21, BAFF and APRIL) with key effects on B cell/plasma cell survival and differentiation, respectively, recent studies have also provided further insights into the effects of currently available drugs on protective immunity and mechanisms of the initiation and progression of RAIDs (i.e. rituximab, belimumab, mycophenolate and azathioprine). A key aspect of B-cell-directed drugs is their impact on continuous immune activation and chronic maintenance which may differ between individual RAIDs.

SUMMARY: The translational advances in the area of B-cell-depleting therapies and more sophisticated approaches to modulate key B-cell functions, such as blocking B-cell receptor downstream effects, interfering with the differentiation and survival of antigen-experienced memory B and plasma cells are of central interest. Differences in the efficacy and safety profiles of B-cell depletion compared with B-cell-modulating therapies (including antigen-specific tolerance induction) need to be further delineated.

Publication Type

Journal Article. Review.

Year of Publication

2014

<394>

Unique Identifier

24108586

Title

Systemic rheumatoid vasculitis in the era of modern immunosuppressive therapy.

Source

Rheumatology. 53(1):145-52, 2014 Jan.

VI 1

Status

MEDLINE

Authors

Ntatsaki E; Mooney J; Scott DG; Watts RA.

Authors Full Name
Ntatsaki, Eleana; Mooney, Janice; Scott, David G I; Watts, Richard A.
Institution
Ntatsaki, Eleana. Clinical Teaching Fellows Office, UCL Medical School, Room GF/664, Royal Free Hospital, Hampstead, London NW3 2PF, UK. ntatsakie@doctors.org.uk.
Abstract
OBJECTIVES: Systemic rheumatoid vasculitis (SRV) is a rare but potentially serious systemic disease manifestation of rheumatoid arthritis (RA) characterized by the development of necrotizing vasculitis. The incidence of SRV appears to be decreasing possibly reflecting progress in RA treatment. The aims of this study were to review the clinical manifestations of SRV in a stable well-defined population during 2001-10 and to compare with our previous cohort (1988-2000) and also a cohort from 1975 to 1981.

METHODS: Using Norfolk Vasculitis Register, a prospective register of patients with systemic vasculitis since 1988, all patients with a diagnosis of SRV from 1 January 2001 until 31 December 2010 were identified. SRV was defined according to the Scott and Bacon criteria (1984). Clinical features were obtained by retrospective case note review.

RESULTS: Eighteen patients with SRV were identified (10 male), median age at diagnosis was 72 years and average disease duration 15.6 years. The average annual incidence for 2001-10 was 3.9 per million. One-year mortality was 12% and 5-year mortality 60%. The clinical manifestations were similar apart from systemic and cutaneous features which were more common in the earlier cohorts.

CONCLUSION: The incidence of SRV has declined significantly in the last 40 years; but the clinical manifestations remain similar. Systemic symptoms, and cutaneous manifestations such as infarcts and nodules, are slightly less common in the recent cohort. Despite modern immunosuppressive therapy the prognosis remains poor.
Publication Type
Comparative Study. Journal Article.
Year of Publication
2014

<395>

Unique Identifier

24022789

Title

CD8+ cells regulate the T helper-17 response in an experimental murine model of Sjogren syndrome.

Source

Mucosal immunology. 7(2):417-27, 2014 Mar.

VI 1

Status

MEDLINE

Authors

Zhang X; Schaumburg CS; Coursey TG; Siemasko KF; Volpe EA; Gandhi NB; Li DQ; Niederkorn JY; Stern ME; Pflugfelder SC; de Paiva CS.

Authors Full Name

Zhang, X; Schaumburg, C S; Coursey, T G; Siemasko, K F; Volpe, E A; Gandhi, N B; Li, D-Q; Niederkorn, J Y; Stern, M E; Pflugfelder, S C; de Paiva, C S.

Institution

Zhang, X. 1] Ocular Surface Center, Cullen Eye Institute, and the Department of Ophthalmology, Baylor College of Medicine, Houston, Texas, USA [2] Department of Ophthalmology, Shenyang He Eye Hospital, Shenyang, Liaoning Province, China.

Schaumburg, C S. Department of Biological Sciences, Allergan, Irvine, California, USA.

Coursey, T G. Ocular Surface Center, Cullen Eye Institute, and the Department of Ophthalmology, Baylor College of Medicine, Houston, Texas, USA.

Siemasko, K F. Department of Biological Sciences, Allergan, Irvine, California, USA.

Volpe, E A. Ocular Surface Center, Cullen Eye Institute, and the Department of Ophthalmology, Baylor College of Medicine, Houston, Texas, USA.

Gandhi, N B. Ocular Surface Center, Cullen Eye Institute, and the Department of Ophthalmology, Baylor College of Medicine, Houston, Texas, USA.

Li, D-Q. Ocular Surface Center, Cullen Eye Institute, and the Department of Ophthalmology, Baylor College of Medicine, Houston, Texas, USA.

Niederkorn, J Y. Department of Ophthalmology, University of Texas Southwestern Medical Center, Dallas, Texas, USA.

Stern, M E. 1] Ocular Surface Center, Cullen Eye Institute, and the Department of Ophthalmology, Baylor College of Medicine, Houston, Texas, USA [2] Department of Biological Sciences, Allergan, Irvine, California, USA.

Pflugfelder, S C. Ocular Surface Center, Cullen Eye Institute, and the Department of Ophthalmology, Baylor College of Medicine, Houston, Texas, USA.
de Paiva, C S. Ocular Surface Center, Cullen Eye Institute, and the Department of Ophthalmology, Baylor College of Medicine, Houston, Texas, USA.
Abstract
This study investigated the regulatory function of CD8+ cells in T helper-17 (Th17) cell-mediated corneal epithelial barrier disruption that develops in a murine desiccating stress (DS) model that resembles Sjogren syndrome. CD8+ cell depletion promoted generation of interleukin-17A (IL-17A)-producing CD4+ T cells via activation of dendritic cells in both the ocular surface and draining cervical lymph nodes in C57BL/6 mice subjected to DS. T-cell-deficient nude recipient mice receiving adoptively transferred CD4+ T cells from CD8+ cell-depleted donors exposed to DS displayed increased CD4+ T-cell infiltration and elevated IL-17A and CC-chemokine attractant ligand 20 levels in the ocular surface, which was associated with greater corneal barrier disruption. Enhanced DS-specific corneal barrier disruption in CD8-depleted donor mice correlated with a Th17-mediated expression of matrix metalloproteinases (MMP-3 and MMP-9) in the recipient corneal epithelium. Co-transfer of CD8+CD103+ regulatory T cells did not affect the ability of DS-specific pathogenic CD4+ T cells to infiltrate and cause ocular surface disease in the nude recipients, showing that CD8+ cells regulate the efferent arm of DS-induced immune response. In summary, CD8+ regulatory cells suppress generation of a pathogenic Th17 response that has a pivotal role in DS-induced disruption of corneal barrier function.
Publication Type
Journal Article. Research Support, N.I.H., Extramural. Research Support, Non-U.S. Gov't.
Year of Publication
2014

<396>
Unique Identifier
23345026
Title
Novel mechanisms of action of the biologicals in rheumatic diseases. [Review]
Source
Clinical Reviews in Allergy & Immunology. 47(1):6-16, 2014 Aug.
VI 1

Status

MEDLINE

Authors

Chighizola CB; Favalli EG; Meroni PL.

Authors Full Name

Chighizola, Cecilia Beatrice; Favalli, Ennio Giulio; Meroni, Pier Luigi.

Institution

Chighizola, Cecilia Beatrice. Division of Rheumatology, Istituto G Pini, Piazza C. Ferrari, 1, 20122, Milan, Italy.

Abstract

Biological drugs targeting pro-inflammatory or co-stimulatory molecules or depleting lymphocyte subsets made a revolution in rheumatoid arthritis (RA) treatment. Their comparable efficacy in clinical trials raised the point of the heterogeneity of RA pathogenesis, suggesting that we are dealing with a syndrome rather than with a single disease. Several tumor necrosis factor-alpha (TNF-alpha) blockers are available, and a burning question is whether they are biosimilar or not. The evidence of diverse biological effects in vitro is in line with the fact that a lack of efficacy to one TNF-alpha agent does not imply a non-response to another one. As proteins, biologicals are potentially immunogenic. It has been recently raised that anti-drug antibodies (ADA) may affect their bioavailability and eventually the clinical efficacy through local formation of immune complexes and directly by preventing the interaction between the drug and TNF-alpha. Regular monitoring of drug and ADA levels appears the best way to tailor anti-TNF-alpha therapies. Owing to the pleiotropic characteristics of the target, anti-TNF-alpha blockers may affect several mechanisms beyond rheumatoid synovitis. As TNF-alpha plays a pivotal role in the induction of early atherosclerosis, treatment with TNF-inhibitors may modulate cholesterol handling, in particular, cholesterol efflux from macrophages. Side effects are a major issue because of the systemic TNF-alpha blocking action. The efficacy of an anti-C5 monoclonal antibody fused to a peptide targeting inflamed synovia in experimental arthritis opened the way for new strategies: Homing to the synovium of molecules neutralizing TNF would allow to maximize the therapeutic action avoiding the side effects.

Publication Type

Journal Article. Review.

Year of Publication

2014

<397>

Unique Identifier

23275079

Title

TNF-related apoptosis-inducing ligand (TRAIL) in rheumatoid arthritis: what's new?. [Review]

Source

Clinical & Experimental Medicine. 14(2):115-20, 2014 May.

VI 1

Status

MEDLINE

Authors

Neve A; Corrado A; Cantatore FP.

Authors Full Name

Neve, Anna; Corrado, Addolorata; Cantatore, Francesco Paolo.

Institution

Neve, Anna. Department of Medical and Surgical Sciences, Rheumatology Clinic, University of Foggia, Ospedale "Col. D'Avanzo", V.le degli Aviatori 1, 71100, Foggia, Italy.

Abstract

TNF-related apoptosis-inducing ligand (TRAIL) is a type II transmembrane protein of the TNF superfamily that serves as an extracellular signal that triggers programmed cell death in tumor cells, without affecting normal cells. Recently, scientists have turned their attention to the emerging role of TRAIL in immune and autoimmune responses. TRAIL has been shown to down-regulate the self-antigens in autoimmune diseases, such as rheumatoid arthritis (RA) by exerting its apoptotic effect on activated T cells and synoviocytes and by its local anti-inflammatory effect. The impact of TRAIL molecular variants and agonistic monoclonal antibodies in the regulation of TRAIL activity in arthritis animal models strongly supports the idea of testing the role of TRAIL in humans, with the aim of developing new effective therapies that promote apoptosis of synoviocytes and/or infiltrating lymphocytes, by targeting TRAIL. The aim of this review is to summarize recent progress and current knowledge of TRAIL functions in RA.

Publication Type

Journal Article. Review.

Year of Publication

2014

<398>

Unique Identifier

24294966

Title

T regulatory cells in childhood arthritis--novel insights. [Review]

Source

Expert Reviews in Molecular Medicine. 15:e13, 2013 Dec 03.

VI 1

Status

MEDLINE

Authors

Pesenacker AM; Wedderburn LR.

Authors Full Name

Pesenacker, Anne M; Wedderburn, Lucy R.

Institution

Pesenacker, Anne M. Rheumatology Unit, UCL Institute of Child Health, University College London, London, UK.

Abstract

In recent years, there have been many new developments in the field of regulatory T cells (Treg), challenging the consensus on their behaviour, classification and role(s) in disease. The role Treg might play in autoimmune disease appears to be more complex than previously thought. Here, we discuss the current knowledge of regulatory T cells through animal and human research and illustrate the recent developments in childhood autoimmune arthritis (juvenile idiopathic arthritis (JIA)). Furthermore, this review summarises our understanding of the fields and assesses current and future implications for Treg in the treatment of JIA.

Publication Type

Journal Article. Research Support, Non-U.S. Gov't. Review.

Year of Publication

2013

<399>

Unique Identifier

24270030

Title

Are immunizations safe and effective for patients being treated with immunosuppressive agents?.

Source

JAAPA. 26(12):12-3, 2013 Dec.

VI 1

Status

MEDLINE

Authors

Bushardt RL; Winter M.

Authors Full Name

Bushardt, Reamer L; Winter, Mary.

Institution

Bushardt, Reamer L. Reamer L. Bushardt is professor and chair of the Department of Physician Assistant Studies at Wake Forest School of Medicine in Winston-Salem, North Carolina, and editor-in-chief of JAAPA. Mary Winter practices in the Department of Pharmacy at Wake Forest Baptist Medical Center in Winston-Salem, North Carolina. The authors have indicated no relationships to disclose relating to the content of this article.

Abstract

Most immunizations have not been well studied in patients with drug-induced immune suppression. This article reviews strategies for administering vaccines to patients with rheumatoid arthritis who are taking disease-modifying antirheumatic drugs.

Publication Type

Journal Article.

Year of Publication

2013

<400>

Unique Identifier

24217844

Title

Biological therapies in rheumatic diseases. [Review]

Source

Clinica Terapeutica. 164(5):e413-28, 2013.

VI 1

Status

MEDLINE

Authors

Conti F; Ceccarelli F; Massaro L; Cipriano E; Di Franco M; Alessandri C; Spinelli FR; Scrivo R.

Authors Full Name

Conti, F; Ceccarelli, F; Massaro, L; Cipriano, E; Di Franco, M; Alessandri, C; Spinelli, F R; Scrivo, R.

Institution

Conti, F. Department of Internal Medicine and Medical Specialties, Rheumatology, 'Sapienza' University, Rome, Italy.

Abstract

The development of the biological drugs has revolutionized the therapeutic approach of the chronic inflammatory rheumatic diseases, particularly in patients resistant to standard treatment. These drugs are characterized by an innovative mechanism of action, based on the targeted inhibition of specific molecular or cellular targets directly involved in the pathogenesis of the diseases: pro-inflammatory cytokines (tumor necrosis factor, interleukin-1 and 6), CTLA-4, and molecules involved in the activation, differentiation and maturation of B cells. Their use has indeed allowed for a better prognosis in several rheumatic diseases (such as rheumatoid arthritis, psoriatic arthritis, ankylosing spondylitis, systemic lupus erythematosus) and to obtain a clinical remission. In the present review we give an overview of the biological drugs currently available for the treatment of the rheumatic diseases, analyzing the different mechanism of action, the therapeutic indications and efficacy data, and adverse events.

Publication Type

Journal Article. Review.

Year of Publication

2013

<401>

Unique Identifier

24204938

Title

A mouse model of adoptive immunotherapeutic targeting of autoimmune arthritis using allo-tolerogenic dendritic cells.

Source

PLoS ONE [Electronic Resource]. 8(10):e77729, 2013.

VI 1

Status

MEDLINE

Authors

Yang J; Yang Y; Ren Y; Xie R; Zou H; Fan H.

Authors Full Name

Yang, Jie; Yang, Yiming; Ren, Yana; Xie, Rufeng; Zou, Hejian; Fan, Huahua.

Institution

Yang, Jie. Blood Engineering Laboratory, Shanghai Blood Center, Shanghai, China ; Division of Rheumatology, Huashan Hospital, Fudan University, Shanghai, China.

Abstract

OBJECTIVE: Tolerogenic dendritic cells (tDCs) are immunosuppressive cells with potent tolerogenic ability and are promising immunotherapeutic tools for treating rheumatoid arthritis (RA). However, it is currently unknown whether allogeneic tDCs (allo-tDCs) induce tolerance in RA, and whether the numbers of adoptively transferred allo-tDCs, or the requirement for pulsing with relevant auto-antigens are important.

METHODS: tDCs were derived from bone marrow precursors of C57BL/B6 mice, which were induced in vitro by GM-CSF, IL-10 and TGF-beta1. Collagen-induced arthritis (CIA) was modeled in D1 mice by immunization with type II collagen (CII) to test the therapeutic ability of allo-tDCs against CIA. Clinical and histopathologic scores, arthritic incidence, cytokine and anti-CII antibody secretion, and CD4(+)Th subsets were analyzed.

RESULTS: tDCs were characterized in vitro by a stable immature phonotype and a potent immunosuppressive ability. Following adoptive transfer of low doses (5x10(5)) of CII-loaded allo-tDCs, a remarkable anti-arthritic activity, improved clinical scores and histological end-points were found. Serological levels of inflammatory cytokines and anti-CII antibodies were also significantly lower in CIA mice treated with CII-pulsed allo-tDCs as compared with allo-tDCs. Moreover, treatment with allo-tDCs altered the proportion of Treg/Th17 cells.

CONCLUSION: These findings suggested that allo-tDCs, especially following antigen loading, reduced the severity of CIA in a dose-dependent manner. The dampening of CIA was associated with modulated cytokine secretion, Treg/Th17 polarization and inhibition of anti-CII secretion. This study highlights the potential therapeutic utility of allo-tDCs in autoimmune arthritis and should facilitate the future design of allo-tDC immunotherapeutic strategies against RA.

Publication Type

Journal Article. Research Support, Non-U.S. Gov't.

Year of Publication

2013

<402>

Unique Identifier

24076765

Title

Immunogenicity and safety of two doses of a non-adjuvanted influenza A H1N1/2009 vaccine in young autoimmune rheumatic diseases patients.

Source

Lupus. 22(13):1394-8, 2013 Nov.

VI 1

Status

MEDLINE

Authors

Aikawa NE; Trudes G; Campos LM; Pereira RM; Moraes JC; Ribeiro AC; Miraglia J; Timenetsky Mdo C; Bonfa E; Silva C.

Authors Full Name

Aikawa, N E; Trudes, G; Campos, L M A; Pereira, R M R; Moraes, J C B; Ribeiro, A C; Miraglia, J; Timenetsky, M do Carmo S; Bonfa, E; Silva, Ca.

Institution

Aikawa, N E. 1Pediatric Rheumatology Unit.

Abstract

OBJECTIVES: The aim of this study was to evaluate the immunogenicity and safety of the influenza A H1N1/2009 vaccine in children under 9 years old with autoimmune rheumatic diseases (ARD).

METHODS: Thirty-eight ARD patients and 11 healthy children received two doses of non-adjuvanted influenza A/California/7/2009 (H1N1) virus-like vaccine. Subjects were evaluated before and 21 days after vaccination. Seroprotection (SP) and seroconversion (SC) rates, geometric mean titers (GMT) and factor increases (FI) in GMT were calculated.

RESULTS: Mean ages were comparable between patients and controls. Pre-vaccination SP and GMT were similar in patients and controls ($p > 0.05$). Three weeks after immunization, SP (81.6% vs. 81.8%, $p = 1.0$), SC (81.6% vs. 90.9%, $p = 0.66$), GMT (151.5 vs. 282.1, $p = 0.26$) and the FI in GMT (16.7 vs. 36.3, $p = 0.23$) were similar in patients and controls, with both groups achieving an adequate response, according to the European Medicines Agency and Food and Drug Administration standards. Analysis of the possible factors influencing SC showed no difference in demographic data, leukocyte/lymphocyte counts or immunosuppressant use between seroconverted and non-seroconverted patients ($p > 0.05$). The vaccine demonstrated a satisfactory safety profile in this population.

CONCLUSIONS: Two doses of influenza A H1N1/2009 vaccination induced an effective antibody response and caused adverse events in rare instances, suggesting this vaccine is appropriate and can be recommended for this age group.

Publication Type

Journal Article. Research Support, Non-U.S. Gov't.

Year of Publication

2013

<403>

Unique Identifier

24048103

Title

Successful etanercept desensitization in a patient with severe injection site reactions.

Source

JCR: Journal of Clinical Rheumatology. 19(7):407-8, 2013 Oct.

VI 1

Status

MEDLINE
Authors
Hall J; Findeisen J.
Authors Full Name
Hall, Joanna; Findeisen, John.
Institution
Hall, Joanna. From the Alfred Hospital, Melbourne, Victoria, Australia.
Publication Type
Case Reports. Journal Article.
Year of Publication
2013

<404>
Unique Identifier
24036370
Title
1,25-Dihydroxyvitamin D3-3-bromoacetate, a novel vitamin D analog induces immunosuppression through PI3K/Akt/mTOR signaling cascade.
Source
International Immunopharmacology. 17(3):744-51, 2013 Nov.
VI 1
Status
MEDLINE
Authors
Datta-Mitra A; Mitra A; Ray R; Raychaudhuri SP; Kundu-Raychaudhuri S.
Authors Full Name
Datta-Mitra, Ananya; Mitra, Anupam; Ray, Rahul; Raychaudhuri, Siba P; Kundu-Raychaudhuri, Smriti.
Institution
Datta-Mitra, Ananya. Department of Internal Medicine/Rheumatology, Allergy and Clinical Immunology, School of Medicine, University of California Davis, Davis, CA 95616, USA; VA Medical Centre Sacramento, Mather, CA 95655, USA.
Abstract

PURPOSE: The molecular mechanism responsible for the immunomodulatory effect of 1,25-dihydroxyvitamin D3 (Vit-D) is still not well elucidated. Unavoidable systemic toxicity of Vit-D has encouraged to develop more potent and less toxic Vit-D analogs, such as 1,25-dihydroxyvitamin D3-3-bromoacetate (BE). Our aim was to explore the immunosuppressive effect of BE and its molecular mechanism in autoimmune diseases.

METHOD: Magnetically sorted CD3(+) T cells (T cells) from PBMCs of psoriasis and autoimmune arthritis patients were cultured with/without BE and Vit-D followed by proliferation (MTT, CFSE dilution assays) and apoptosis assays (annexin V). Immunoblot was performed to determine the signaling cascade responsible for the antiproliferative effect.

RESULTS: In MTT assay, BE (OD: 0.64+/-0.08) markedly inhibited the anti-CD3/CD28 stimulated proliferation of T cells (OD: 1.8+/-0.30, p<0.001) and at equivalent doses, the inhibitory effect was more than that of Vit-D (OD: 0.91+/-0.11, p<0.05). The antiproliferative effect of BE was extended to activated CD4(+) and CD8(+) memory T cells (CD45RA(-)CD11a(+)) without much effect on the naive T cells. BE induced more apoptosis of T cells (45.01+/-4.27%, p<0.01) compared to untreated cells (3.45+/-1.8%), and the proapoptotic effect was markedly more than that of Vit-D (26.1+/-2.05%, p<0.05). BE effectively inhibited the anti-CD3/CD28-induced phosphorylation of Akt and mTOR and in both, BE showed more potency than Vit-D (p<0.05).

CONCLUSION: Topical Vit-D is being used successfully in psoriasis for years. However, its potency is less compared to topical corticosteroids. The de novo BE showed significantly more immunosuppression than conventional Vit-D and the immunosuppressive effect is PI3K/Akt/mTOR dependent. Our results indicate that BE could be an effective therapeutic agent for psoriasis and other T-cell-mediated autoimmune diseases. Copyright Published by Elsevier B.V.

Publication Type

Journal Article. Research Support, U.S. Gov't, Non-P.H.S..

Year of Publication

2013

<405>

Unique Identifier

23998731

Title

Current immunotherapy in rheumatoid arthritis. [Review]

Source

Immunotherapy. 5(9):955-74, 2013 Sep.

VI 1

Status

MEDLINE

Authors

Meier FM; Frerix M; Hermann W; Muller-Ladner U.

Authors Full Name

Meier, Florian M P; Frerix, Marc; Hermann, Walter; Muller-Ladner, Ulf.

Institution

Meier, Florian M P. Department of Internal Medicine & Rheumatology, Justus-Liebig-University Giessen, Kerckhoff-Klinik, Bad Nauheim, Germany.

Abstract

Rheumatoid arthritis is a common autoimmune disease primarily manifesting as chronic synovitis, subsequently leading to a change in joint integrity. Progressive disability and systemic complications are strongly associated with a decreased quality of life. To maintain function and health in patients with rheumatoid arthritis, early, aggressive and guided immunosuppressive therapy is required to induce clinical remission. Antirheumatic drugs are capable of controlling synovial inflammation and are therefore named 'disease-modifying antirheumatic drugs' (DMARDs). This article aims to bridge the beginning of DMARD therapy with agents such as methotrexate, leflunomide, sulfasalazine, injectable gold and (hydroxy)chloroquine with biological therapies, and with the new era of kinase inhibitors. Mechanisms of action, as well as advantages and disadvantages of DMARDs, are discussed with respect to the current literature and current recommendations.

Publication Type

Journal Article. Review.

Year of Publication

2013

<406>

Unique Identifier

23960240

Title

The cellular source and target of IL-21 in K/BxN autoimmune arthritis.

Source

Journal of Immunology. 191(6):2948-55, 2013 Sep 15.

VI 1

Status

MEDLINE

Authors

Block KE; Huang H.

Authors Full Name

Block, Katharine E; Huang, Haochu.

Institution

Block, Katharine E. Committee on Immunology, University of Chicago, Chicago, IL 60637, USA.

Abstract

IL-21 is a pluripotent cytokine that regulates B cell and plasma cell differentiation and is thought be an autocrine factor for follicular helper T cell (T(FH)) and Th17 differentiation. Although IL-21 has been implicated in autoimmune diseases, its relevant cellular source and target cells have not been well characterized. We investigated this issue in the K/BxN mouse model of autoimmune arthritis. Adoptive transfer of KRN-transgenic CD4+ T cells into appropriate hosts drives germinal center (GC) formation and autoantibody production against glucose-6-phosphate isomerase, leading to joint inflammation and destruction. By comparing transfer of T or B cells deficient in IL-21 or IL-21R, we were able to dissect the contribution of each cell type. T cells deficient in IL-21 did not induce GC formation or autoantibody production, but they went through normal T(FH) differentiation. However, T cells lacking IL-21R induced Ab titers, GC B cell frequency, and arthritis development similar to wild-type T cells, suggesting that IL-21 is not required for T(FH) differentiation and function. IL-21 acts on B cells, because IL-21R expression on B cells was required to induce disease. In contrast, Th17 cells, a T cell subset that also produces IL-21 and can provide help to B cells, are not required for the GC response and arthritis. These data have implications in developing effective therapies for rheumatoid arthritis and other Ab-mediated autoimmune diseases.

Publication Type

Journal Article. Research Support, N.I.H., Extramural.

Year of Publication

2013

<407>

Unique Identifier

23929239

Title

[Vaccination in adult patients with chronic inflammatory rheumatic diseases]. [German]

Source

Zeitschrift fur Rheumatologie. 72(7):690-4, 696-700, 702-4, 2013 Sep.

VI 1

Status

MEDLINE

Authors

Goldacker S; Gause AM; Warnatz K; Kommission Pharmakotherapie der DGRh.

Authors Full Name

Goldacker, S; Gause, A M; Warnatz, K; Kommission Pharmakotherapie der DGRh.

Institution

Goldacker, S. Centrum fur Chronische Immmundefizienz, Universitatsklinikum Freiburg und Universitat Freiburg, Breisacher Str. 117 - 2. OG, 79106, Freiburg, Deutschland. sigune.goldacker@uniklinik-freiburg.de

Abstract

Patients with chronic inflammatory rheumatic diseases often have an intrinsic and therapy associated increased susceptibility to infections which substantially contributes to morbidity and mortality of the patients. A large proportion of these infections are preventable by vaccination. For this reason in 2005 the standing vaccination committee (STIKO) recommended for patients with immunosuppression vaccination against pneumococcus, influenza, Haemophilus influenza b and meningococcus in addition to standard vaccinations, independent of age. Every patient should therefore be informed about a possible increase in susceptibility to infections and the recommended prevention by vaccination before implementation of immunosuppressive therapy.

Publication Type

English Abstract. Journal Article.

Year of Publication

2013

<408>

Unique Identifier

23929238

Title

[Vaccination recommendations of the Commission for Pharmacotherapy of the German Society of Rheumatology]. [German]

Source

Zeitschrift fur Rheumatologie. 72(7):687-9, 2013 Sep.

VI 1

Status

MEDLINE

Authors

Warnatz K; Goldacker S; Gause AM; die Kommission Pharmakotherapie der DGRh.

Authors Full Name

Warnatz, K; Goldacker, S; Gause, A M; die Kommission Pharmakotherapie der DGRh.

Institution

Warnatz, K. Centrum fur Chronische Immmundefizienz, Universitatsklinikum Freiburg, Breisacher Str. 117-2. OG, 79106, Freiburg, Deutschland. klaus.warnatz@uniklinik-freiburg.de

Publication Type

Journal Article.

Year of Publication

2013

<409>

Unique Identifier

23908512

Title

Novel immunotherapies for rheumatoid arthritis. [Review]

Source

Clinical Medicine. 13(4):391-4, 2013 Aug.

VI 1

Status

MEDLINE

Authors

Richardson S; Isaacs J.

Authors Full Name

Richardson, Sarah; Isaacs, John.

Institution

Richardson, Sarah. Newcastle University, UK.

Publication Type

Journal Article. Review.

Year of Publication

2013

<410>

Unique Identifier

23869798

Title

Transforming growth factor (TGF)-beta signalling is increased in rheumatoid synovium but TGF-beta blockade does not modify experimental arthritis.

Source

Clinical & Experimental Immunology. 174(2):245-55, 2013 Nov.

VI 1

Status

MEDLINE

Authors

Gonzalo-Gil E; Criado G; Santiago B; Dotor J; Pablos JL; Galindo M.

Authors Full Name

Gonzalo-Gil, E; Criado, G; Santiago, B; Dotor, J; Pablos, J L; Galindo, M.

Institution

Gonzalo-Gil, E. Hospital 12 de Octubre, 'i+12', Instituto de Investigacion, Madrid, Spain.

Abstract

The aim of this study was to analyse the distribution of regulatory and inhibitory mothers against decapentaplegic homologue (Smad) proteins as markers of active transforming growth factor (TGF)-beta signalling in rheumatoid arthritis (RA) synovial tissue and to investigate the effect of TGF-beta blockade in the development and progression of collagen-induced arthritis. The expression of Smad proteins in synovial tissues from RA, osteoarthritic and healthy controls was analysed by immunohistochemistry. Arthritis was induced in DBA/1 mice by immunization with chicken type-II collagen (CII). TGF-beta was blocked in vivo with the specific peptide p17 starting at the time of immunization or on the day of arthritis onset. T cell population frequencies and specific responses to CII were analysed. The expression of cytokines and transcription factors was quantified in spleen and joint samples. Statistical differences between groups were compared using the Mann-Whitney U-test or one-way analysis of variance (anova) using the Kruskal-Wallis test. p-Smad-2/3 and inhibitory Smad-7 expression were detected in RA and control tissues. In RA, most lymphoid infiltrating cells showed nuclear p-Smad-2/3 without Smad-7 expression. Treatment with TGF-beta antagonist did not affect clinical severity, joint inflammation and cartilage damage in collagen-induced arthritis. Frequency of T cell subsets, mRNA levels of cytokines and transcription factors, specific proliferation to CII, serum interleukin (IL)-6 and anti-CII antibodies were comparable in p17 and phosphate-buffered saline (PBS)-treated groups. The pattern of Smad proteins expression demonstrates active TGF-beta signalling in RA synovium. However, specific TGF-beta blockade does not have a significant effect in the mice model of collagen-induced arthritis. Copyright © 2013 British Society for Immunology.

Publication Type

Journal Article. Research Support, Non-U.S. Gov't.

Year of Publication

2013

<411>

Unique Identifier

23869084

Title

Long-term remission of T-cell large granular lymphocyte leukemia associated with rheumatoid arthritis after rituximab therapy.

Source

Blood. 122(9):1583-6, 2013 Aug 29.
VI 1
Status
MEDLINE
Authors
Cornec D; Devauchelle-Pensec V; Jousse-Joulin S; Marhadour T; Ugo V; Berthou C; Douet-Guilbert N; Saraux A.
Authors Full Name
Cornec, Divi; Devauchelle-Pensec, Valerie; Jousse-Joulin, Sandrine; Marhadour, Thierry; Ugo, Valerie; Berthou, Christian; Douet-Guilbert, Nathalie; Saraux, Alain.
Institution
Cornec, Divi. Service de Rhumatologie, Centre Hospitalier Regional et Universitaire de Brest, Brest, France. divi.cornec@chu-brest.fr
Abstract
T-cell large granular lymphocyte leukemia (LGLL) is a rare clonal disease often associated with rheumatoid arthritis (RA) and manifests chiefly as neutropenia and recurrent infections. Immunosuppressive agents are the mainstay of treatment, but long-term remissions are rare. We report 2 cases of LGLL in patients with RA successfully treated with rituximab, a monoclonal antibody specific of B cells and approved for treating RA. The first patient experienced a complete LGLL remission that was sustained during the 8-year follow-up after the first rituximab infusion. In the second patient, rituximab therapy was followed by immediate neutropenia recovery and then by marked shrinkage of the LGLL clone 1 year later. The paradoxical efficacy of this specific anti-B-cell drug on a monoclonal T-cell disease suggests that some cases of LGLL may be reactive manifestations of chronic autoantigen stimulation rather than true malignancies.
Publication Type
Case Reports. Journal Article.
Year of Publication
2013

<412>
Unique Identifier
23867949
Title

Is there a therapeutic window of opportunity in early inflammatory bowel disease? Early stage inflammatory bowel disease: the actual management. [Review]

Source

Minerva Gastroenterologica e Dietologica. 59(3):299-312, 2013 Sep.

VI 1

Status

MEDLINE

Authors

Kevans D; Van Assche G.

Authors Full Name

Kevans, D; Van Assche, G.

Institution

Kevans, D. Zane Cohen Centre for Digestive Diseases, Mount Sinai Hospital, Toronto, Ontario, Canada - GVanAssche@mtsinai.on.ca.

Abstract

Traditionally therapy for inflammatory bowel disease (IBD) encompassed a sequential approach with subjects treated with 5-ASA products and/or corticosteroids initially, and only where failing such treatment, moving on to immunomodulator or biologic therapy. In the rheumatologic literature the importance of the early introduction of immunosuppressive therapies for inflammatory arthropathies has been increasingly recognized, however this concept remains much debated in IBD with no clear consensus on the optimal therapeutic approach. In this review we discuss how the natural history of IBD provides a rationale for the early introduction of the most effective therapy. We outline how the experience of early immunosuppressive therapy in rheumatoid arthritis informs therapeutic decision making in IBD. We review the evolving treatment strategies in IBD and the current evidence supporting the introduction of immunosuppressive treatment soon after IBD diagnosis. Finally we discuss the importance of selecting appropriate therapeutic endpoints in IBD and review the potential risks and benefits of early immunosuppressive treatment strategies in IBD.

Publication Type

Journal Article. Research Support, Non-U.S. Gov't. Review.

Year of Publication

2013

<413>

Unique Identifier

23864239

Title

Predominantly proinflammatory cytokines decrease after B cell depletion therapy in patients with primary Sjogren's syndrome.

Source

Annals of the Rheumatic Diseases. 72(12):2048-50, 2013 Dec.

VI 1

Status

MEDLINE

Authors

Pollard RP; Abdulahad WH; Bootsma H; Meiners PM; Spijkervet FK; Huitema MG; Burgerhof JG; Vissink A; Kroese FG.

Authors Full Name

Pollard, R P E; Abdulahad, W H; Bootsma, H; Meiners, P M; Spijkervet, F K L; Huitema, M G; Burgerhof, J G M; Vissink, A; Kroese, F G M.

Institution

Pollard, R P E. Department of Oral and Maxillofacial Surgery, University of Groningen, University Medical Center Groningen,, Groningen, The Netherlands.

Publication Type

Journal Article. Randomized Controlled Trial. Research Support, Non-U.S. Gov't.

Year of Publication

2013

<414>

Unique Identifier

23863094

Title

Heat shock proteins can be targets of regulatory T cells for therapeutic intervention in rheumatoid arthritis. [Review]

Source

International Journal of Hyperthermia. 29(5):448-54, 2013 Aug.

VI 1

Status

MEDLINE

Authors

Van Herwijnen MJ; Van Der Zee R; Van Eden W; Broere F.

Authors Full Name

Van Herwijnen, Martijn J C; Van Der Zee, Ruurd; Van Eden, Willem; Broere, Femke.

Institution

Van Herwijnen, Martijn J C. Department of Infectious Diseases and Immunology, Utrecht University, Utrecht, the Netherlands.

Abstract

Rheumatoid arthritis (RA) is a chronic autoimmune disease characterised by excessive immune responses resulting in inflammation of the joints. Although current therapies can be successful in dampening inflammation, a long-lived state of tolerance is seldom achieved. Therefore, novel therapies are needed that restore and maintain tolerance in patients with RA. Targeting regulatory T cells (Tregs) is a successful strategy to achieve tolerance, as was shown in studies performed in animal models and in human clinical trials. The antigen-specificity of Tregs is crucial for their effectiveness and allows for very specific targeting of these cells. However, which antigen is suitable for autoimmune diseases such as RA, for which the autoantigens are largely unknown? Heat shock proteins (HSPs) are ubiquitously expressed and can be up-regulated during inflammation. Additionally, HSPs, or HSP-derived peptides are immunogenic and can be recognised by a variety of immune cells, including Tregs. Therefore, this review highlights the potential of HSP-specific Tregs to control inflammatory immune responses. Targeting HSP-specific Tregs in RA can be achieved via the administration of HSPs (derived peptides), thereby controlling inflammatory responses. This makes HSPs attractive candidates for therapeutic intervention in chronic autoimmune diseases, with the ultimate goal of inducing long-lasting tolerance.

Publication Type

Journal Article. Research Support, Non-U.S. Gov't. Review.

Year of Publication

2013

<415>

Unique Identifier

23845805

Title

Strategies for active TNF-alpha vaccination in rheumatoid arthritis treatment. [Review]

Source

Vaccine. 31(38):4063-8, 2013 Aug 28.

VI 1

Status

MEDLINE

Authors

Jia T; Pan Y; Li J; Wang L.

Authors Full Name

Jia, Tingting; Pan, Yang; Li, Jinming; Wang, Lunan.

Institution

Jia, Tingting. Graduate School, Peking Union Medical College, Chinese Academy of Medical Sciences, Beijing, People's Republic of China.

Abstract

Local overexpression of tumor necrosis factors alpha (TNF-alpha) is critically involved in the inflammatory response and tissue destruction of rheumatoid arthritis (RA). Currently, the blockade of TNF-alpha by passive immunotherapy is indeed efficacious in the treatment of RA, but it still present some disadvantages. Induction of high level of anti-TNF-alpha neutralizing autoantibodies by TNF-alpha autovaccine has been developed to avoid these shortcomings. This review is to briefly introduce several vaccination approaches that have been used to induce a B cell response, including coupled TNF-alpha (entire/peptide) with a carrier protein, modified TNF-alpha with foreign Th cell epitopes, and engineered DNA vaccine. These methods showed remarkable therapeutic efficiency in experimental animals which indicated that active TNF-alpha immunization would be a promising and cost-effective new treatment option for RA. Copyright © 2013 Elsevier Ltd. All rights reserved.

Publication Type

Journal Article. Research Support, Non-U.S. Gov't. Review.

Year of Publication

2013

<416>

Unique Identifier

23820860

Title

Vaccinations in juvenile chronic inflammatory diseases: an update. [Review]

Source

Nature Reviews Rheumatology. 9(9):532-43, 2013 Sep.

VI 1

Status

MEDLINE

Authors

Silva CA; Aikawa NE; Bonfa E.

Authors Full Name

Silva, Clovis A; Aikawa, Nadia E; Bonfa, Eloisa.

Institution

Silva, Clovis A. Pediatric Rheumatology Unit, Instituto da Crianca, Faculty of Medicine, University of Sao Paulo, Avenida Dr. Eneas de Carvalho Aguiar 647, Cerqueira Cesar, Sao Paulo, SP, Brazil. clovis.silva@ hc.fm.usp.br

Abstract

Vaccination is a powerful tool to reduce the burden of infectious diseases in paediatric patients with chronic rheumatic diseases. Live attenuated vaccines are not recommended for profoundly immunosuppressed patients, but nonlive vaccines have adequate safety and efficacy profiles in the few (admittedly underpowered) studies published to date. No severe vaccine-specific or disease-specific adverse events have been observed in patients with juvenile idiopathic arthritis (JIA) or childhood-onset systemic lupus erythematosus (SLE) who have been vaccinated with live or nonlive agents. The immune response to live vaccines is variable in these patients but generally adequate, despite concomitant use of immunosuppressive and biologic agents. The proposal that onset of autoimmune rheumatic diseases could be induced by vaccination is controversial and primarily based on case reports; however, patients with mevalonate kinase deficiency can experience febrile attacks after immunizations. Adequately powered studies of live and nonlive vaccination in patients with paediatric rheumatic diseases are necessary to clarify safety and efficacy issues. This narrative Review discusses vaccination in patients with JIA, childhood-onset SLE, juvenile dermatomyositis, juvenile systemic sclerosis, primary vasculitis and autoinflammatory syndromes. Vaccine safety, short-term and long-term changes in disease parameters, and the immunogenicity and influence of immunosuppressive agents are outlined for each combination of disease and vaccine.

Publication Type

Journal Article. Research Support, Non-U.S. Gov't. Review.
Year of Publication
2013

<417>
Unique Identifier
23806224
Title
Efficient boosting of the antiviral T cell response in B cell-depleted patients with autoimmune rheumatic diseases following influenza vaccination.
Source
Clinical & Experimental Rheumatology. 31(5):723-30, 2013 Sep-Oct.
VI 1
Status
MEDLINE
Authors
Muller RB; Maier R; Hoschler K; Zambon M; Ludewig B; Herrmann M; Schulze-Koops H; von Kempis J.
Authors Full Name
Muller, Rudiger B; Maier, Reinhard; Hoschler, Katja; Zambon, Maria; Ludewig, Burkhard; Herrmann, Martin; Schulze-Koops, Hendrik; von Kempis, Johannes.
Institution
Muller, Rudiger B. Division of Rheumatology, Department of Internal Medicine, Kantonsspital St. Gallen, St. Gallen, Switzerland. ruediger.mueller@kssg.ch.
Abstract
OBJECTIVES: Booster vaccination against 2009 H1N1 influenza virus was recommended for rheumatologic patients under immunosuppressive therapy during the 2009/2010 H1N1 pandemic. In this study we assessed whether B cell depletion with rituximab influences of the antiviral immune response in 2009 H1N1 influenza virus-vaccinated patients.

METHODS: Influenza virus-specific immune responses were analysed after the first and a booster vaccination with pandemrixTM in sixteen consecutive rituximab-treated patients with different rheumatic autoimmune disorders. Antibody titers were determined by a

haemagglutination-inhibition assay and virus-specific T cell responses were evaluated by a flow cytometry-based intracellular cytokine-secretion assay. Patients showing clinical symptoms of influenza infection were excluded from this study.

RESULTS: Two out of seven patients with low (<10%) and four out of nine with normal (>10%) B cells developed significant antibody responses after the first vaccination. Booster vaccination led to an antibody response in one additional patient. After the first vaccination, virus-specific CD4+ and CD8+ T cell responses were significantly lower in patients with low B cells than in those with normal B cells. Of importance, the booster vaccination stimulated the antiviral T cell response only in patients with low B cells.

CONCLUSIONS: In the absence of a significant effect of booster vaccinations against 2009 H1N1 influenza virus on the humoral immune response in B cell-depleted patients with autoimmune rheumatic diseases, enhanced antiviral T cell responses in patients with low B cells indicate that T cells, maybe, compensate for the impaired humoral immunity in these patients.

Publication Type

Journal Article.

Year of Publication

2013

<418>

Unique Identifier

23806191

Title

Markedly decreased antibody titers against hepatitis B in previously immunised children presenting with juvenile idiopathic arthritis.

Source

Clinical & Experimental Rheumatology. 31(6):969-73, 2013 Nov-Dec.

VI 1

Status

MEDLINE

Authors

Maritsi D; Vartzelis G; Soldatou A; Garoufi A; Spyridis N.

Authors Full Name
Maritsi, Despoina; Vartzelis, George; Soldatou, Alex; Garoufi, Anastasia; Spyridis, Nikos.
Institution
Maritsi, Despoina. 2nd Department of Academic Pediatrics, Medical Faculty, University of Athens, Athens, Greece. dmaritsi@yahoo.co.uk.
Abstract
OBJECTIVES: Hepatitis B is a vaccine preventable disease with intermediate endemicity in Greece. Patients with juvenile idiopathic arthritis (JIA) on immunomodulating therapy are prone to infection or reactivation of hepatitis B virus (HBV). The aim of this study is to define the immune status against HBV in children newly-diagnosed with JIA.

METHODS: Case-control prospective study including 89 JIA patients and 89 controls matched for age and gender. Eighty-nine JIA patients were included in the study (22 males), with a mean age of 6.8 years. Sera were tested for hepatitis B surface antigen, hepatitis B core antibody, and anti-HBs. Patients with anti-HBs titers >=10 IU/L were considered immune. Data were analysed with SPSS 18.0 version.

RESULTS: In the JIA group 55% were HBV immune (anti-HBs level >=10 IU/L) while in the control group 92% were immune against HBV (p<0.001). Antibody levels in the patient group were significantly lower compared to the control group. The mean concentration of anti-HBs levels in JIA patients was 18.3 IU/L versus 82.6 IU/L in the control group (p<0.001).

CONCLUSIONS: Antibody titers against HBV in fully vaccinated JIA patients due to start treatment are significantly lower compared to matched healthy children in this study. Diagnosis of JIA and older age were associated with the absence of protective antibodies. Although there is no evidence to support the introduction of a booster HBV dose in healthy children who mount low antibody response following immunisation, further studies are required to address this question in patients with JIA.
Publication Type
Journal Article.
Year of Publication
2013

<419>

Unique Identifier

23800433

Title

IL-33 in rheumatoid arthritis: potential role in pathogenesis and therapy. [Review]

Source

Human Immunology. 74(9):1057-60, 2013 Sep.

VI 1

Status

MEDLINE

Authors

Xu WD; Zhang M; Zhang YJ; Ye DQ.

Authors Full Name

Xu, Wang-Dong; Zhang, Min; Zhang, Yu-Jing; Ye, Dong-Qing.

Institution

Xu, Wang-Dong. Department of Epidemiology and Biostatistics, School of Public Health, Anhui Medical University, 81 Meishan Road, Hefei, Anhui 230032, PR China.

Abstract

Rheumatoid arthritis (RA) is characterized by chronic inflammatory disease, including synovial proliferation and excessive pro-inflammatory cytokines production, leading to cartilage and bone destruction. Cytokine-mediated immunity plays an important role in the pathogenesis of various autoimmune diseases such as RA. Recently, the IL-1 family member IL-33, was recognized to perform as an inflammatory cytokine, exerted profound effects in human RA and experimental inflammatory arthritis. Furthermore, inhibition of IL-33 signaling proposed a potential therapeutic approach. In this review, we summarize recent advances on the pathological roles of IL-33 in RA and discuss the therapeutic significance of these new findings. Copyright © 2013 American Society for Histocompatibility and Immunogenetics. Published by Elsevier Inc. All rights reserved.

Publication Type

Journal Article. Research Support, Non-U.S. Gov't. Review.

Year of Publication

2013

<420>

Unique Identifier
23780457
Title
Effects of the live attenuated measles-mumps-rubella booster vaccination on disease activity in patients with juvenile idiopathic arthritis: a randomized trial.
Source
JAMA. 309(23):2449-56, 2013 Jun 19.
VI 1
Status
MEDLINE
Authors
Heijstek MW; Kamphuis S; Armbrust W; Swart J; Gorter S; de Vries LD; Smits GP; van Gageldonk PG; Berbers GA; Wulffraat NM.
Authors Full Name
Heijstek, Marloes W; Kamphuis, Sylvia; Armbrust, Wineke; Swart, Joost; Gorter, Simone; de Vries, Lara D; Smits, Gaby P; van Gageldonk, Pieter G; Berbers, Guy A M; Wulffraat, Nico M.
Institution
Heijstek, Marloes W. Department of Pediatric Immunology, University Medical Center Utrecht, Wilhelmina Children's Hospital, Room KC03.063.0, PO Box 85090, 3508 AB, Utrecht, The Netherlands. m.w.heijstek@umcutrecht.nl
Abstract
IMPORTANCE: The immunogenicity and the effects of live attenuated measles-mumps-rubella (MMR) vaccination on disease activity in patients with juvenile idiopathic arthritis (JIA) are matters of concern, especially in patients treated with immunocompromising therapies.

OBJECTIVES: To assess whether MMR booster vaccination affects disease activity and to describe MMR booster immunogenicity in patients with JIA.

DESIGN, SETTING, AND PARTICIPANTS: Randomized, multicenter, open-label clinical equivalence trial including 137 patients with JIA aged 4 to 9 years who were recruited from 5 academic hospitals in The Netherlands between May 2008 and July 2011.

INTERVENTION: Patients were randomly assigned to receive MMR booster vaccination (n=68) or no vaccination (control group; n=69). Among patients taking biologics, these treatments were discontinued at 5 times their half-lives prior to vaccination.

MAIN OUTCOMES AND MEASURES: Disease activity as measured by the Juvenile Arthritis Disease Activity Score (JADAS-27), ranging from 0 (no activity) to 57 (high activity). Disease activity in the year following randomization was compared between revaccinated patients and controls using a linear mixed model. A difference in JADAS-27 of 2.0 was the equivalence margin. Primary immunogenicity outcomes were seroprotection rates and MMR-specific antibody concentrations at 3 and 12 months.

RESULTS: Of 137 randomized patients, 131 were analyzed in the modified intention-to-treat analysis, including 60 using methotrexate and 15 using biologics. Disease activity during complete follow-up did not differ between 63 revaccinated patients (JADAS-27, 2.8; 95% CI, 2.1-3.5) and 68 controls (JADAS-27, 2.4; 95% CI, 1.7-3.1), with a difference of 0.4 (95% CI, -0.5 to 1.2), within the equivalence margin of 2.0. At 12 months, seroprotection rates were higher in revaccinated patients vs controls (measles, 100% vs 92% [95% CI, 84%-99%]; mumps, 97% [95% CI, 95%-100%] vs 81% [95% CI, 72%-93%]; and rubella, 100% vs 94% [95% CI, 86%-100%], respectively), as were antibody concentrations against measles (1.63 vs 0.78 IU/mL; P = .03), mumps (168 vs 104 RU/mL; P = .03), and rubella (69 vs 45 IU/mL; P = .01). Methotrexate and biologics did not affect humoral responses, but low patient numbers precluded definite conclusions.

CONCLUSION AND RELEVANCE: Among children with JIA who had undergone primary immunization, MMR booster vaccination compared with no booster did not result in worse JIA disease activity and was immunogenic. Larger studies are needed to assess MMR effects in patients using biologic agents.

TRIAL REGISTRATION: clinicaltrials.gov Identifier: NCT00731965.
Publication Type
Journal Article. Multicenter Study. Randomized Controlled Trial. Research Support, Non-U.S. Gov't.
Year of Publication
2013

<421>
Unique Identifier

23725286

Title

The future of B cell-targeted therapies in Sjogren's syndrome. [Review]

Source

Immunotherapy. 5(6):639-46, 2013 Jun.

VI 1

Status

MEDLINE

Authors

Cornec D; Saraux A; Devauchelle-Pensec V; Clodic C; Pers JO.

Authors Full Name

Cornec, Divi; Saraux, Alain; Devauchelle-Pensec, Valerie; Clodic, Coralie; Pers, Jacques-Olivier.

Institution

Cornec, Divi. Department of Rheumatology, CHRU la Cavale Blanche, Brest, France.

Abstract

Primary Sjogren's syndrome is a systemic autoimmune disease characterized by progressive exocrine gland destruction, resulting clinically in eyes and mouth dryness. To date, no treatment has been proven effective to modify the course of this slow-evolving disease. B cells are now considered to play a central role in the pathogenesis of primary Sjogren's syndrome because their functions are not restrained to antibody production. Thus, several B-cell targeting therapies are under clinical investigation. Rituximab, a monoclonal antibody directed to CD20 and leading to transient blood B-cell depletion, has shown partial improvements in subjective and objective sicca symptoms in small studies. However, the results of two large controlled trials are awaited before considering its use in large populations of patients. Several other therapeutic strategies are being studied, targeting other B-cell surface proteins (epratuzumab and anti-CD22) or major cytokines of B-cell homeostasis (e.g., BAFF, IL-6 and lymphotoxin-beta). Although great hope is generated by the trials of these specific therapies, another challenge for clinical researchers is the development of reliable tools to assess the activity of Sjogren's syndrome and its response to treatment.

Publication Type

Journal Article. Review.

Year of Publication

2013

<422>

Unique Identifier

23681395

Title

Successful desensitization to low-dose methotrexate.

Source

Rheumatology. 52(12):2305-6, 2013 Dec.

VI 1

Status

MEDLINE

Authors

Fernando SL.

Authors Full Name

Fernando, Suran L.

Institution

Fernando, Suran L. Department of Clinical Immunology and Allergy, Royal North Shore Hospital, Reserve Road, St Leonards, NSW 2065, Australia. sfernando@nsccahs.health.nsw.gov.au.

Publication Type

Case Reports. Letter.

Year of Publication

2013

<423>

Unique Identifier

23628802

Title

Regulation of TNF-alpha with a focus on rheumatoid arthritis. [Review]

Source

Immunology & Cell Biology. 91(6):393-401, 2013 Jul.

VI 1

Status

MEDLINE

Authors

Moelants EA; Mortier A; Van Damme J; Proost P.

Authors Full Name

Moelants, Eva A V; Mortier, Anneleen; Van Damme, Jo; Proost, Paul.

Institution

Moelants, Eva A V. Laboratory of Molecular Immunology, Department of Microbiology and Immunology, Rega Institute for Medical Research, KU Leuven, Leuven, Belgium.

Abstract

Cytokines and chemokines represent two important groups of proteins that control the human immune system. Dysregulation of the network in which these immunomodulators function can result in uncontrolled inflammation, leading to various diseases including rheumatoid arthritis (RA), characterized by chronic inflammation and bone erosion. Potential triggers of RA include autoantibodies, cytokines and chemokines. The tight regulation of cytokine and chemokine production, and biological activity is important. Tumor necrosis factor-alpha (TNF-alpha) is abundantly present in RA patients' serum and the arthritic synovium. This review, therefore, discusses first the role and regulation of the major proinflammatory cytokine TNF-alpha, in particular the regulation of TNF-alpha production, post-translational processing and signaling of TNF-alpha and its receptors. Owing to the important role of TNF-alpha in RA, the TNF-alpha-producing cells and the dynamics of its expression, the direct and indirect action of this cytokine and possible biological therapy for RA are described.

Publication Type

Journal Article. Research Support, Non-U.S. Gov't. Review.

Year of Publication

2013

<424>

Unique Identifier

23627915

Title

Janus kinase inhibition with tofacitinib: changing the face of inflammatory bowel disease treatment. [Review]

Source

Current Drug Targets. 14(12):1385-91, 2013 Nov.

VI 1

Status

MEDLINE

Authors

Vuitton L; Koch S; Peyrin-Biroulet L.

Authors Full Name

Vuitton, Lucine; Koch, Stephane; Peyrin-Biroulet, Laurent.

Institution

Vuitton, Lucine. Inserm U954 and Department of Hepato-Gastroenterology, University Hospital of Nancy-Brabois, Universite Henri Poincare 1 Allee du Morvan, 54511 Vandoeuvre-les-Nancy, France. peyrinbiroulet@gmail.com.

Abstract

The advent of anti-Tumor Necrosis Factor (TNF) therapy has changed the way of treating inflammatory bowel disease (IBD). However, primary and secondary failure are relatively frequent with all anti-TNF agents, which are available only as parenteral agents. Tofacitinib is an oral janus kinase (JAK) inhibitor that inhibits JAK family kinase members, in particular JAK1 and JAK3, achieving a broad limitation of inflammation by interfering with several cytokine receptors. It first proved its efficacy as an immunosuppressive regimen after renal transplantation, and was recently approved by the FDA for rheumatoid arthritis. First data in IBD are promising, especially in ulcerative colitis. Ongoing clinical trials in both UC and Crohn's disease (CD) are needed to further explore its efficacy in CD and to better assess its safety profile.

Publication Type

Journal Article. Review.

Year of Publication

2013

<425>

Unique Identifier

23607771

Title

Immunization with 60 kD Ro peptide produces different stages of preclinical autoimmunity in a Sjogren's syndrome model among multiple strains of inbred mice.

Source

Clinical & Experimental Immunology. 173(1):67-75, 2013 Jul.

VI 1

Status

MEDLINE

Authors

Kurien BT; Dsouza A; Igoe A; Lee YJ; Maier-Moore JS; Gordon T; Jackson M; Scofield RH.

Authors Full Name

Kurien, B T; Dsouza, A; Igoe, A; Lee, Y J; Maier-Moore, J S; Gordon, T; Jackson, M; Scofield, R H.

Institution

Kurien, B T. Arthritis & Clinical Immunology Program, Oklahoma Medical Research Foundation, Oklahoma City, OK, USA.

Abstract

Sjogren's syndrome is a chronic illness manifested characteristically by immune injury to the salivary and lacrimal glands, resulting in dry mouth/eyes. Anti-Ro [Sjogren's syndrome antigen A (SSA)] and anti-La [Sjogren's syndrome antigen B (SSB)] autoantibodies are found frequently in Sjogren's subjects as well as in individuals who will go on to develop the disease. Immunization of BALB/c mice with Ro60 peptides results in epitope spreading with anti-Ro and anti-La along with lymphocyte infiltration of salivary glands similar to human Sjogren's. In addition, these animals have poor salivary function/low saliva volume. In this study, we examined whether Ro-peptide immunization produces a Sjogren's-like illness in other strains of mice. BALB/c, DBA-2, PL/J, SJL/J and C57BL/6 mice were immunized with Ro60 peptide-274. Sera from these mice were studied by immunoblot and enzyme-linked immunosorbent assay for autoantibodies. Timed salivary flow was determined after pharmacological stimulation, and salivary glands were examined pathologically. We found that SJL/J mice had no immune response to the peptide from Ro60, while C57BL/6 mice produced antibodies that bound the peptide but had no epitope spreading. PL/J mice had epitope spreading to other structures of Ro60 as well as to La, but like C57BL/6 and SJL/J had no salivary gland lymphocytic infiltration and no decrement of salivary function. DBA-2 and BALB/c mice had infiltration but only BALB/c had decreased salivary function. The immunological processes leading to a Sjogren's-like illness after Ro-peptide immunization were interrupted in a stepwise fashion in these differing mice strains. These data suggest that this is a model of preclinical disease with genetic control for epitope spreading, lymphocytic infiltration and glandular dysfunction. Copyright Published 2013. This article is a U.S. Government work and is in the public domain in the USA.

Publication Type
  Journal Article. Research Support, N.I.H., Extramural. Research Support, Non-U.S. Gov't.
Year of Publication
  2013

<426>
Unique Identifier
  23588512
Title
  2012 Brazilian Society of Rheumatology Consensus on vaccination of patients with rheumatoid arthritis.
Source
  Revista Brasileira de Reumatologia. 53(1):4-23, 2013 Feb.
VI 1
Status
  MEDLINE
Authors
  Brenol CV; da Mota LM; Cruz BA; Pileggi GS; Pereira IA; Rezende LS; Bertolo MB; Freitas MV; Silva NA; Louzada-Junior P; Giorgi RD; Lima RA; Pinheiro Gda R; Brazilian Society of Rheumatology.
Authors Full Name
  Brenol, Claiton Viegas; da Mota, Licia Maria Henrique; Cruz, Boris Afonso; Pileggi, Gecilmara Salviato; Pereira, Ivanio Alves; Rezende, Lucila Stange; Bertolo, Manoel Barros; Freitas, Max Victor Carioca; Silva, Nilzio Antonio da; Louzada-Junior, Paulo; Giorgi, Rina Dalva Neubarth; Lima, Rodrigo Aires Correa; Pinheiro, Geraldo da Rocha Castelar; Brazilian Society of Rheumatology.
Institution
  Brenol, Claiton Viegas. Departamento de Medicina Interna, Universidade Federal do Rio Grande do Sul, Brazil. cbrenol@hcpa.ufrgs.br
Abstract
  OBJECTIVE: To elaborate recommendations to the vaccination of patients with rheumatoid arthritis (RA) in Brazil.

METHOD: Literature review and opinion of expert members of the Brazilian Society of Rheumatology Committee of Rheumatoid Arthritis and of an invited pediatric rheumatologist.

RESULTS AND CONCLUSIONS: The following 12 recommendations were established: 1) Before starting disease-modifying anti-rheumatic drugs, the vaccine card should be reviewed and updated; 2) Vaccines against seasonal influenza and against H1N1 are indicated annually for patients with RA; 3) The pneumococcal vaccine should be indicated for all patients with RA; 4) The vaccine against varicella should be indicated for patients with RA and a negative or dubious history for that disease; 5) The HPV vaccine should be considered for adolescent and young females with RA; 6) The meningococcal vaccine is indicated for patients with RA only in the presence of asplenia or complement deficiency; 7) Asplenic adults with RA should be immunized against Haemophilus influenzae type B; 8) An additional BCG vaccine is not indicated for patients diagnosed with RA; 9) Hepatitis B vaccine is indicated for patients with RA who are negative for antibodies against HBsAg; the combined hepatitis A and B vaccine should be considered; 10) Patients with RA and at high risk for tetanus, who received rituximab in the preceding 24 weeks, should undergo passive immunization with tetanus immunoglobulin in case of exposure; 11) The YF vaccine is contraindicated to patients with RA on immunosuppressive drugs; 12) The above described recommendations should be reviewed over the course of RA.

Publication Type

Consensus Development Conference. Journal Article. Practice Guideline.

Year of Publication

2013

<427>

Unique Identifier

23588511

Title

Vaccination for patients with rheumatoid arthritis: a pressing need.

Source

Revista Brasileira de Reumatologia. 53(1):1-3, 2013 Feb.

VI 1

Status

MEDLINE

Authors
  Brenol CV; Pileggi GS.
Authors Full Name
  Brenol, Claiton Viegas; Pileggi, Gecilmara Salviato.
Publication Type
  Editorial.
Year of Publication
  2013

<428>
Unique Identifier
  23578666
Title
  The role of citrullination of an immunodominant proteoglycan (PG) aggrecan T cell epitope in BALB/c mice with PG-induced arthritis.
Source
  Immunology Letters. 152(1):25-31, 2013 Apr.
VI 1
Status
  MEDLINE
Authors
  Misjak P; Bosze S; Horvati K; Pasztoi M; Paloczi K; Holub MC; Szakacs F; Aradi B; Gyorgy B; Szabo TG; Nagy G; Glant TT; Mikecz K; Falus A; Buzas EI.
Authors Full Name
  Misjak, Petra; Bosze, Szilvia; Horvati, Kata; Pasztoi, Maria; Paloczi, Krisztina; Holub, Marianna C; Szakacs, Ferenc; Aradi, Borbala; Gyorgy, Bence; Szabo, Tamas G; Nagy, Gyorgy; Glant, Tibor T; Mikecz, Katalin; Falus, Andras; Buzas, Edit I.
Institution
  Misjak, Petra. Department of Genetics, Cell- and Immunobiology, Semmelweis University, Budapest, Hungary.
Abstract
  The P70-84 peptide (also called 5/4E8 epitope) of the human cartilage proteoglycan (PG) aggrecan is the dominant/arthritogenic epitope in both humans and arthritis-prone BALB/c mice

(PG-induced arthritis, PGIA). An elevated T cell reactivity was demonstrated to a citrullinated version of the P70-84 epitope in most of the patients with rheumatoid arthritis (RA). The goal of this study was to understand better how a T cell epitope, if citrullinated, may affect antigenicity/arthritogenicity in PGIA, a murine model of RA. T cell reactivity to differentially citrullinated versions of either the human PG aggrecan P70-84 peptide or the corresponding mouse sequence was assessed in peptide or aggrecan-immunized and arthritic BALB/c mice as well as in T cell receptor transgenic mice specific for peptide P70-84 sequence. Peripheral T cell responses were induced by priming BALB/c mice with either the human wild-type or its citrullinated versions. Unexpectedly, priming with the citrullinated self-peptide induced a higher T cell response compared to the wild-type sequence ($p<0.001$), and the citrullination of the human peptide abolished T cell reactivity in PGIA. Our data suggest that T cells reactive to the citrullinated P70-84 peptide escaped thymic selection and are present in the peripheral T cell repertoire. Results of this study provide evidence that citrullination of an immunodominant T cell epitope may substantially alter, either increase or abolish, T cell recognition at the periphery in an experimental model of arthritis. Copyright © 2013 Elsevier B.V. All rights reserved.

Publication Type

Journal Article. Research Support, Non-U.S. Gov't.

Year of Publication

2013

<429>

Unique Identifier

23574312

Title

Tolerogenic dendritic cell therapy for rheumatoid arthritis: where are we now?. [Review]

Source

Clinical & Experimental Immunology. 172(2):148-57, 2013 May.

VI 1

Status

MEDLINE

Authors

Hilkens CM; Isaacs JD.

Authors Full Name

Hilkens, C M U; Isaacs, J D.
Institution
Hilkens, C M U. Institute of Cellular Medicine, Newcastle University, Newcastle upon Tyne, UK. catharien.hilkens@ncl.ac.uk
Abstract
Dendritic cells with tolerogenic function (tolDC) have become a promising immunotherapeutic tool for reinstating immune tolerance in rheumatoid arthritis (RA) and other autoimmune diseases. The concept underpinning tolDC therapy is that it specifically targets the pathogenic autoimmune response while leaving protective immunity intact. Findings from human in-vitro and mouse in-vivo studies have been translated into the development of clinical grade tolDC for the treatment of autoimmune disorders. Recently, two tolDC trials in RA and type I diabetes have been carried out and other trials are in progress or are imminent. In this review, we provide an update on tolDC therapy, in particular in relation to the treatment of RA, and discuss the challenges and the future perspectives of this new experimental immunotherapy. Copyright © 2012 British Society for Immunology.
Publication Type
Journal Article. Research Support, Non-U.S. Gov't. Review.
Year of Publication
2013

<430>
Unique Identifier
23549103
Title
Immune suppression in cynomolgus monkeys by XPro9523: an improved CTLA4-Ig fusion with enhanced binding to CD80, CD86 and neonatal Fc receptor FcRn.
Source
mAbs. 5(3):384-96, 2013 May-Jun.
VI 1
Status
MEDLINE
Authors

Bernett MJ; Chu SY; Leung I; Moore GL; Lee SH; Pong E; Chen H; Phung S; Muchhal US; Horton HM; Lazar GA; Desjarlais JR; Szymkowski DE.
Authors Full Name
Bernett, Matthew J; Chu, Seung Y; Leung, Irene; Moore, Gregory L; Lee, Sung-Hyung; Pong, Erik; Chen, Hsing; Phung, Sheryl; Muchhal, Umesh S; Horton, Holly M; Lazar, Greg A; Desjarlais, John R; Szymkowski, David E.
Institution
Bernett, Matthew J. Xencor, Inc.; Monrovia, CA USA.
Chu, Seung Y. Xencor, Inc.; Monrovia, CA USA.
Leung, Irene. Xencor, Inc.; Monrovia, CA USA.
Moore, Gregory L. Xencor, Inc.; Monrovia, CA USA.
Lee, Sung-Hyung. Xencor, Inc.; Monrovia, CA USA.
Pong, Erik. Xencor, Inc.; Monrovia, CA USA.
Chen, Hsing. Xencor, Inc.; Monrovia, CA USA.
Phung, Sheryl. Xencor, Inc.; Monrovia, CA USA.
Muchhal, Umesh S. Xencor, Inc.; Monrovia, CA USA.
Horton, Holly M. Xencor, Inc.; Monrovia, CA USA.
Lazar, Greg A. Xencor, Inc.; Monrovia, CA USA.
Desjarlais, John R. Xencor, Inc.; Monrovia, CA USA.
Szymkowski, David E. Xencor, Inc.; Monrovia, CA USA.
Abstract
The CTLA4-Ig fusion proteins abatacept and belatacept are clinically proven immunosuppressants used for rheumatoid arthritis and renal transplant, respectively. Given that both biologics are typically administered chronically by infusion, a need exists for a next-generation CTLA4-Ig with more convenient dosing. We used structure-based protein engineering to optimize the affinity of existing CTLA4-Ig therapeutics for the ligands CD80 and CD86, and for the neonatal Fc receptor, FcRn. From a rationally designed library, we identified four substitutions that enhanced binding to human CD80 and CD86. Coupled with two IgG1 Fc substitutions that enhanced binding to human FcRn, these changes comprise the novel CTLA4-Ig fusion protein, XPro9523. Compared with abatacept, XPro9523 demonstrated 5.9-fold, 23-fold, and 12-fold increased binding to CD80, CD86, and FcRn, respectively; compared with belatacept, CD80, CD86, and FcRn binding increased 1.5-fold, 7.7-fold, and 11-fold, respectively. XPro9523 and belatacept suppressed human T cell proliferation and IL-2 production more potently than abatacept. XPro9523 also suppressed inflammation in the mouse collagen-induced arthritis model. In cynomolgus monkeys, XPro9523 saturated CD80 and CD86 more effectively than abatacept and belatacept, potently inhibited IgM and IgG immunization responses, and demonstrated longer half-life. Pharmacokinetic modeling of its increased potency and persistence

suggests that, in humans, XPro9523 may demonstrate superior efficacy and dosing convenience compared with abatacept and belatacept.

Publication Type

Journal Article.

Year of Publication

2013

<431>

Unique Identifier

23547216

Title

Longterm effects of rituximab on B cell counts and autoantibody production in rheumatoid arthritis: use of high-sensitivity flow cytometry for more sensitive assessment of B cell depletion.

Source

Journal of Rheumatology. 40(5):565-71, 2013 May.

VI 1

Status

MEDLINE

Authors

Vancsa A; Szabo Z; Szamosi S; Bodnar N; Vegh E; Gergely L; Szucs G; Szanto S; Szekanecz Z.

Authors Full Name

Vancsa, Andrea; Szabo, Zoltan; Szamosi, Szilvia; Bodnar, Nora; Vegh, Edit; Gergely, Lajos; Szucs, Gabriella; Szanto, Sandor; Szekanecz, Zoltan.

Institution

Vancsa, Andrea. Department of Rheumatology, Division of Hematology, Institute of Medicine, University of Debrecen Medical and Health Sciences Center, Debrecen, Hungary. andrea.vancsa@gmail.com

Abstract

OBJECTIVE: To assess the efficacy and safety of longterm rituximab (RTX) therapy for rheumatoid arthritis (RA) and study correlations among B cell depletion, clinical response, and autoantibody production.

METHODS: Seventy-seven patients with moderate or high RA activity received RTX and were re-treated every 6 months regardless of clinical response. All patients received at least 5 cycles. We assessed 28-joint Disease Activity Score (DAS28), IgM rheumatoid factor (RF), and anticitrullinated protein antibody (ACPA) levels at baseline, after 15 days, and then every 6 months for 24 months. Absolute CD19+ B lymphocyte counts were determined in 50 patients using high-sensitivity flow cytometry (hsFACS) by reading 100,000 events.

RESULTS: After 6, 12, 18, and 24 months, 51.6%, 51.9%, 73.3%, and 83.8% of patients, respectively, showed good European League Against Rheumatism responses. Significant and sustained decreases in IgM RF and ACPA levels were observed as early as 6 months and 12 months, respectively. The baseline mean absolute B cell number was 0.234 g/l. B cell numbers diminished significantly after the very first infusion by Day 15 (0.104 g/l; $p = 0.007$); they further decreased until 24 months (0.0013 g/l; $p < 0.001$). One RTX infusion resulted in incomplete depletion in 76.7% of patients. Upon RTX treatment, changes in CD19+ B cell numbers positively correlated with changes in DAS28 ($r = 0.963$, $p = 0.008$) and IgM RF ($r = 0.859$, $p = 0.028$), but not with changes in ACPA production ($r = 0.726$, $p = 0.102$). The correlations between B cell numbers and DAS28 were observed in both ACPA-seropositive ($r = 0.999$, $p < 0.0001$) and ACPA-negative patient subpopulations ($r = 0.962$, $p = 0.009$). The correlation between CD19+ cell numbers and IgM RF was observed only in the ACPA-positive population ($r = 0.944$, $p = 0.005$) but not in seronegative patients ($r = 0.398$, $p = 0.435$). No safety issues arose.

CONCLUSION: In RA, clinical response to RTX is associated with the extent of B cell depletion and with autoantibody production. Changes in CD19+ B cell numbers correlate with those in disease activity and, in seropositive patients, also with IgM RF, but not with ACPA production. We found that hsFACS may be a useful method to more accurately assess incomplete B cell depletion.

Publication Type

Clinical Trial. Journal Article.

Year of Publication

2013

<432>

Unique Identifier

23510070

Title

Perspectives on epigenetic-based immune intervention for rheumatic diseases. [Review]

Source

Arthritis Research & Therapy. 15(2):207, 2013 Mar 14.

VI 1

Status

MEDLINE

Authors

Gray SG.

Authors Full Name

Gray, Steven G.

Abstract

Rheumatic disease can loosely be described as any painful condition affecting the loco-motor system, including joints, muscles, connective tissues, and soft tissues around the joints and bones. There is a wide spectrum of rheumatic diseases, many of which involve autoimmunity, including systemic lupus erythematosus and rheumatoid arthritis. A significant body of evidence now links aberrant epigenetic regulation of gene expression with rheumatic disease and points toward the use of epigenetic targeting agents as potential new treatment options, particularly for those conditions associated with an autoimmune element. In this perspective, I will briefly cover the current knowledge surrounding this area in the field of rheumatology.

Publication Type

Journal Article. Review.

Year of Publication

2013

<433>

Unique Identifier

23480185

Title

Protection from articular damage by passive or active anti-tumour necrosis factor (TNF)-alpha immunotherapy in human TNF-alpha transgenic mice depends on anti-TNF-alpha antibody levels.

Source

Clinical & Experimental Immunology. 172(1):54-62, 2013 Apr.
VI 1
Status
MEDLINE
Authors
Semerano L; Biton J; Delavallee L; Duvallet E; Assier E; Bessis N; Bernier E; Dhellin O; Grouard-Vogel G; Boissier MC.
Authors Full Name
Semerano, L; Biton, J; Delavallee, L; Duvallet, E; Assier, E; Bessis, N; Bernier, E; Dhellin, O; Grouard-Vogel, G; Boissier, M-C.
Institution
Semerano, L. Universite Paris-13 Sorbonne Paris Cite, EA4222, Paris, France. luca.semerano@avc.aphp.fr
Abstract
Active anti-tumour necrosis factor (TNF)-alpha immunization with the kinoid of TNF-alpha (TNF-K) induces polyclonal anti-TNF-alpha antibodies and ameliorates arthritis in human TNF-alpha (hTNF-alpha) transgenic mice (TTg). We compared the efficacy of TNF-K to that of infliximab (IFX) and of TNF-K and IFX co-administration, and evaluated whether the titres of anti-hTNF-alpha antibodies induced by immunization were a determinant of TNF-K efficacy. Forty-eight TTg mice received one of the following treatments: TNF-K immunization (TNF-K group); weekly IFX throughout the study duration (IFXw0-15); TNF-K plus weekly IFX for 4 weeks (TNF-K + IFX); and weekly IFX for 4 weeks (IFXw0-4); PBS. Animals were killed at week 16. Anti-hTNF-alpha antibody titres and clinical and histological scores were compared. All TNF-K immunized mice (TNF-K and TNF-K + IFX) produced anti-hTNF-alpha antibodies. Titres were higher in TNF-K versus TNF-K + IFX (P < 0.001) and correlated inversely with histological inflammation (R = -0.78; P = 0.0001) and destruction (R = -0.67; P = 0.001). TNF-K + IFX had higher histological inflammation and destruction versus TNF-K (P < 0.05). A receiver operating characteristic (ROC) analysis of anti-hTNF-alpha antibody titres identified the criterion cut-off value to discriminate most effectively between the TNF-K and TNF-K + IFX groups. Mice with high versus low titres had less histological inflammation and destruction (P < 0.05). In a model of TNF-alpha-dependent arthritis, protection from articular damage by TNF-K correlates with the titres of induced anti-hTNF-alpha antibodies. The co-administration of TNF-K and a short course of infliximab does not result in less articular damage versus solely TNF-K, due probably to lower anti-hTNF-alpha antibody production. These results are relevant for future development of active anti-TNF-alpha immunization in human disease. Copyright © 2012 British Society for Immunology.
Publication Type
Journal Article.

Year of Publication

2013

<434>

Unique Identifier

23467774

Title

Septic shock after seasonal influenza vaccination in an HIV-infected patient during treatment with etanercept for rheumatoid arthritis: a case report.

Source

Clinical & Vaccine Immunology: CVI. 20(5):761-4, 2013 May.

VI 1

Status

MEDLINE

Authors

De Nardo P; Bellagamba R; Corpolongo A; Gentilotti E; Taglietti F; Rosati S; Galeazzi M; Sebastiani GD; Quinti I; Nicastri E.

Authors Full Name

De Nardo, Pasquale; Bellagamba, Rita; Corpolongo, Angela; Gentilotti, Elisa; Taglietti, Fabrizio; Rosati, Silvia; Galeazzi, Mauro; Sebastiani, Gian Domenico; Quinti, Isabella; Nicastri, Emanuele.

Institution

De Nardo, Pasquale. National Institute for Infectious Diseases L. Spallanzani, Rome, Italy. dr.pasqualedenardo@gmail.com

Abstract

Anti-tumor necrosis factor alpha (anti-TNF-alpha) is used in the treatment of rheumatic diseases not responsive to first-line regimens. Data on the safety of anti-TNF-alpha in HIV-infected patients are scarce and conflicting. We describe a case of septic shock and multiorgan failure that occurred after etanercept initiation and influenza vaccination in an HIV-infected woman with rheumatoid arthritis.

Publication Type

Case Reports. Journal Article.

Year of Publication

2013

<435>

Unique Identifier

23421370

Title

Elevated serum and synovial fluid levels of interleukin-34 in rheumatoid arthritis: possible association with disease progression via interleukin-17 production.

Source

Journal of Interferon & Cytokine Research. 33(7):398-401, 2013 Jul.

VI 1

Status

MEDLINE

Authors

Tian Y; Shen H; Xia L; Lu J.

Authors Full Name

Tian, Ye; Shen, Hui; Xia, Liping; Lu, Jing.

Institution

Tian, Ye. Department of Rheumatology, 1st Affiliated Hospital of China Medical University, Shen Yang, China.

Abstract

To measure the levels of interleukin-34 (IL-34) in serum and synovial fluid (SF) of patients with rheumatoid arthritis (RA) and to evaluate the effect of recombination human (rh) IL-34 on IL-17 production by peripheral blood mononuclear cells (PBMC) in RA patients, the serum and SF levels of IL-34, and the production of IL-17 by rhIL-34-treated PBMC of RA patients were measured by enzyme-linked immunosorbent assay. We also tested the change of IL-34 level after tumor necrosis factor (TNF)-alpha blockade therapy in 30 RA patients. In contrast to almost no detectable IL-34 in osteoarthritis (OA) and healthy serum, IL-34 could be detected in 93 out of the 125 RA cases (74.4%). Sera IL-34 levels were significantly higher in RA patients compared with the controls and correlated with disease activity. IL-34 levels were higher in SF samples than in sera in 11 RA patients. The level of serum IL-34 decreased after anti-TNF treatment. In the presence of rhIL-34, stimulation of PBMC from RA patients resulted in increased production of IL-17. These findings suggest that IL-34 may play a role in the pathogenesis of RA.

Publication Type

Journal Article. Research Support, Non-U.S. Gov't.
Year of Publication
2013

<436>
Unique Identifier
23374912
Title
Dendritic cells and the promise of antigen-specific therapy in rheumatoid arthritis. [Review]
Source
Arthritis Research & Therapy. 15(1):204, 2013 Feb 04.
VI 1
Status
MEDLINE
Authors
Thomas R.
Authors Full Name
Thomas, Ranjeny.
Abstract
Rheumatoid arthritis (RA) is a systemic inflammatory disease resulting from an autoimmune response to self-antigens, leading to inflammation of synovial tissue of joints and subsequent cartilage and bone erosion. Current disease-modifying anti-rheumatic drugs and biologic inhibitors of TNF, IL-6, T cells and B cells block inflammation nonspecifically, which may lead to adverse effects, including infection. They do not generally induce long-term drug-free remission or restoration of immune tolerance to self-antigens, and lifelong treatment is usual. The development of antigen-specific strategies in RA has so far been limited by insufficient knowledge of autoantigens, of the autoimmune pathogenesis of RA and of the mechanisms of immune tolerance in man. Effective tolerance-inducing antigen-specific immunotherapeutic strategies hold promise of greater specificity, of lower toxicity and of a longer-term solution for controlling or even preventing RA. This paper reviews current understanding of autoantigens and their relationship to immunopathogenesis of RA, and emerging therapeutics that aim to leverage normal tolerance mechanisms for implementation of antigen-specific therapy in RA.
Publication Type

Journal Article. Research Support, Non-U.S. Gov't. Review.
Year of Publication
2013

<437>
Unique Identifier
23370371
Title
Vaccination and auto-immune rheumatic diseases: lessons learnt from the 2009 H1N1 influenza virus vaccination campaign. [Review]
Source
Current Opinion in Rheumatology. 25(2):164-70, 2013 Mar.
VI 1
Status
MEDLINE
Authors
Touma Z; Gladman DD; Urowitz MB.
Authors Full Name
Touma, Zahi; Gladman, Dafna D; Urowitz, Murray B.
Institution
Touma, Zahi. University of Toronto, Toronto Western Research Institute, University of Toronto Lupus Clinic, Centre for Prognosis Studies in the Rheumatic Diseases, Toronto Western Hospital, University of Toronto, Toronto, Ontario, Canada.
Abstract
PURPOSE OF REVIEW: To determine the safety and efficacy of adjuvant and nonadjuvant influenza A/H1NI vaccination in patients with rheumatic diseases.

RECENT FINDINGS: Due to immune abnormalities and the use of steroids and immunosuppressant treatment, patients with rheumatic diseases are susceptible to infections including influenza. Infections continue to be one of the leading causes of morbidity and mortality in rheumatic diseases, partly due to the disease processes and partly due to medications. Viral infections are particularly an issue, so vaccinations would be advisable. However, because of the abnormalities in immune mechanisms in many rheumatic diseases, it is not clear whether

vaccinations are well tolerated and effective. A number of studies confirmed the efficacy and safety of adjuvant and nonadjuvant influenza A/H1NI vaccination in patients with rheumatic diseases. The potential side effects associated with H1N1 vaccines were not different from those observed with seasonal influenza vaccine. The use of steroids and immunosuppressant therapies may alter the efficacy of the vaccines. Adjuvant and nonadjuvant influenza A/H1NI vaccinations have no clinically important effect on production or levels of autoantibodies in patients with rheumatic diseases.

SUMMARY: H1N1 vaccination should be given to patients with rheumatic diseases.

Publication Type

Journal Article. Research Support, Non-U.S. Gov't. Review.

Year of Publication

2013

<438>

Unique Identifier

23355360

Title

A vaccine against Streptococcus pyogenes: the potential to prevent rheumatic fever and rheumatic heart disease. [Review]

Source

American Journal of Cardiovascular Drugs. 13(1):1-4, 2013 Feb.

VI 1

Status

MEDLINE

Authors

Guilherme L; Ferreira FM; Kohler KF; Postol E; Kalil J.

Authors Full Name

Guilherme, Luiza; Ferreira, Frederico Moraes; Kohler, Karen Francine; Postol, Edilberto; Kalil, Jorge.

Institution

Guilherme, Luiza. Heart Institute (InCor), Laboratory of Immunology, University of Sao Paulo, Sao Paulo, Brazil. luizagui@usp.br

Abstract

Streptococcus pyogenes causes severe, invasive infections such as the sequelae associated with acute rheumatic fever, rheumatic heart disease, acute glomerulonephritis, uncomplicated pharyngitis, and pyoderma. Efforts to produce a vaccine against S. pyogenes began several decades ago, and different models have been proposed. We have developed a vaccine candidate peptide, StreptInCor, comprising 55 amino acid residues of the C-terminal portion of the M protein and encompassing both the T- and B-cell protective epitopes. The present article summarizes data from the previous 5 years during which we tested the immunogenicity and safety of StreptInCor in different animal models. We showed that StreptInCor overlapping peptides induced cellular and humoral immune responses of individuals bearing different HLA class II molecules. These results are consistent with peptides that have a universal vaccine epitope. The tridimensional molecular structure of StreptInCor was elucidated by nuclear magnetic resonance spectroscopy, which showed that its structure is composed of two microdomains linked by an 18-residue alpha-helix. Additionally, we comprehensively evaluated the structural stability of the StreptInCor peptide in different physicochemical conditions using circular dichroism. Additional experiments were performed with inbred, outbred, and HLA class II transgenic mice. Analysis of several organs of these mice showed neither deleterious nor autoimmune reactions even after a long period of vaccination, indicating that the StreptInCor candidate peptide could be considered as an immunogenic and safe vaccine.

Publication Type

Journal Article. Research Support, Non-U.S. Gov't. Review.

Year of Publication

2013

<439>

Unique Identifier

23312448

Title

Mesenchymal stromal cells isolated from children with systemic juvenile idiopathic arthritis suppress innate and adaptive immune responses.

Source

Cytotherapy. 15(3):280-91, 2013 Mar.

VI 1

Status

MEDLINE

Authors

Calkoen FG; Brinkman DM; Vervat C; van Ostaijen-Ten Dam MM; Ten Cate R; van Tol MJ; Ball LM.

Authors Full Name

Calkoen, Friso G J; Brinkman, Danielle M C; Vervat, Carly; van Ostaijen-Ten Dam, Monique M; Ten Cate, Rebecca; van Tol, Maarten J D; Ball, Lynne M.

Institution

Calkoen, Friso G J. Department of Paediatrics, Leiden University Medical Centre, Leiden, The Netherlands.

Abstract

BACKGROUND AIMS: Infusion of mesenchymal stromal cells (MSCs) has been reported to be an effective treatment modality for acute graft-versus-host disease, and MSCs have been considered for use in the treatment of patients with autoimmune diseases. Before contemplating clinical studies with MSCs in patients with systemic juvenile idiopathic arthritis (sJIA), the immunomodulatory capacity of MSCs in this setting needs to be explored. A comparative analysis of bone marrow-derived MSCs from children with sJIA and healthy pediatric controls was performed.

METHODS: MSCs were successfully expanded from 11 patients with sJIA and 10 controls. The phenotype, differentiation and immunomodulatory capacity of these MSCs were compared. The effect of immunosuppressive drugs on MSC function was also investigated.

RESULTS: MSCs from patients with sJIA and controls showed no differences in their suppressive effect using control peripheral blood mononuclear cells. Furthermore, the suppression of the response of peripheral blood mononuclear cells from patients with sJIA by autologous sJIA MSCs and allogeneic control MSCs was comparable. The immunosuppressive effect of both groups of MSCs was diminished in the presence of indomethacin ($P < 0.05$). MSCs from patients with sJIA and controls suppressed interleukin-2-induced natural killer cell activation to a similar extent. In addition, MSCs of patients with sJIA and controls inhibited the differentiation of monocytes to dendritic cells.

CONCLUSIONS: This is the first explorative study in a significant cohort of patients with sJIA to evaluate the effect of MSCs on adaptive and innate immune responses. The comparable immunosuppressive characteristics of MSCs derived from patients with sJIA to age-matched

controls support the potential use of patient-derived MSCs in the treatment of sJIA. Copyright © 2013 International Society for Cellular Therapy. Published by Elsevier Inc. All rights reserved.
Publication Type
Journal Article. Research Support, Non-U.S. Gov't.
Year of Publication
2013

<440>
Unique Identifier
23293979
Title
Transient depletion of CD4+ CD25+ regulatory T cells results in multiple autoimmune diseases in wild-type and B-cell-deficient NOD mice.
Source
Immunology. 139(2):179-86, 2013 Jun.
VI 1
Status
MEDLINE
Authors
Ellis JS; Wan X; Braley-Mullen H.
Authors Full Name
Ellis, Jason S; Wan, Xiaoxiao; Braley-Mullen, Helen.
Institution
Ellis, Jason S. Department of Medicine, University of Missouri School of Medicine, Columbia, MO, USA.
Abstract
Approximately 80% of female wild-type non-obese diabetic (WT NOD) mice spontaneously develop diabetes, whereas B-cell-deficient (B(-/-)) NOD mice are resistant to diabetes. B(-/-) mice are also resistant to other spontaneous and experimentally induced autoimmune diseases, including arthritis, systemic lupus erythematosus, Sjogren syndrome and thyroiditis. Under normal conditions, activation of self-reactive T cells in the periphery is limited by CD4(+) CD25(+) natural regulatory T (Treg) cells. B(-/-) NOD.H-2h4 mice, normally resistant to spontaneous autoimmune thyroiditis (SAT), develop SAT when Treg cells are depleted, suggesting that Treg cells are

preferentially activated when autoantigen is initially presented by non-B-cell antigen-presenting cells. To test the hypothesis that increased Treg cell activity in B(-/-) mice contributes to their resistance to other autoimmune diseases, WT and B(-/-) NOD mice were given anti-CD25 to transiently deplete CD4(+) CD25(+) Treg cells. The WT and B(-/-) NOD mice given anti-CD25 developed diabetes much earlier than WT mice given rat IgG, whereas rat IgG-treated B(-/-) mice did not develop diabetes. Treg-cell-depleted mice had increased lymphocyte infiltration of the pancreas, salivary glands and thyroid compared with controls given rat IgG. These results are consistent with the hypothesis that resistance of B-cell-deficient NOD mice to several autoimmune diseases is due to the activity of Treg cells. Copyright © 2013 Blackwell Publishing Ltd.

Publication Type

Journal Article. Research Support, N.I.H., Extramural.

Year of Publication

2013

<441>

Unique Identifier

23286942

Title

Treatment with anti-NAP monoclonal antibody reduces disease severity in murine model of novel angiogenic protein-induced or ovalbumin-induced arthritis.

Source

Clinical & Experimental Immunology. 171(2):155-63, 2013 Feb.

VI 1

Status

MEDLINE

Authors

Nataraj NB; Krishnamurthy J; Salimath BP.

Authors Full Name

Nataraj, N B; Krishnamurthy, J; Salimath, B P.

Institution

Nataraj, N B. Department of Biotechnology, University of Mysore, Karnataka, India.

Abstract

Rheumatoid arthritis (RA) is a polyarticular inflammatory, angiogenic disease. Synovial angiogenesis contributes to inflammation in RA. In this study we have developed an arthritic model in rats using a novel angiogenic protein (NAP), isolated from human synovial fluid of RA patients. We produced anti-NAP monoclonal antibodies (mAbs) and investigated the therapeutic efficacy of the same in adjuvant-induced or NAP-induced arthritis as a model of human RA. The treatment of arthritic rats with anti-NAP mAbs resulted in effective amelioration of paw oedema, radiological arthritic characteristics, serum levels of vascular endothelial growth factor (VEGF) and NAP, compared to that of untreated arthritic animals. Further, profiling of angiogenic markers such as synovial microvessel density, angiogenesis, CD31, VEGF and fms-like tyrosine kinase (Flt1) by immunohistochemistry both in arthritic and anti-NAP mAb-treated animals revealed the efficacy of mAb as an anti-angiogenic functional antibody. Therefore, NAP may be an attractive target to design anti-angiogenic and anti-arthritic therapies to control the pathogenesis of arthritis.
Copyright © 2012 British Society for Immunology.
Publication Type
Journal Article. Research Support, Non-U.S. Gov't.
Year of Publication
2013

<442>
Unique Identifier
23286772
Title
Persistence of antibody response 1.5 years after vaccination using 7-valent pneumococcal conjugate vaccine in patients with arthritis treated with different antirheumatic drugs.
Source
Arthritis Research & Therapy. 15(1):R1, 2013 Jan 04.
VI 1
Status
MEDLINE
Authors
Crnkic Kapetanovic M; Saxne T; Truedsson L; Geborek P.
Authors Full Name
Crnkic Kapetanovic, Meliha; Saxne, Tore; Truedsson, Lennart; Geborek, Pierre.

Abstract

INTRODUCTION: The aim of this study was to explore the persistence of an antibody response 1.5 years after vaccination with 7-valent pneumococcal conjugate vaccine in patients with rheumatoid arthritis (RA) or spondyloarthropathy (SpA) treated with different antirheumatic drugs.

METHODS: Of 505 patients initially recruited, data on current antirheumatic treatment and blood samples were obtained from 398 (79%) subjects after mean (SD, range) 1.4 (0.5; 1 to 2) years. Antibody levels against pneumococcal serotypes 23F and 6B were analyzed by using enzyme-linked immunosorbent assay (ELISA). Original treatment groups were as follows: (a) RA receiving methotrexate (MTX); (b) RA taking anti-TNF monotherapy; (c) RA taking anti-TNF+MTX; (d) SpA with anti-TNF monotherapy; (e) SpA taking anti-TNF+MTX; and (f) SpA taking NSAID/analgesics. Geometric mean levels (GMLs; 95% CI) and proportion (percentage) of patients with putative protective antibody levels>=1 mg/L for both serotypes, calculated in different treatment groups, were compared with results 4 to 6 weeks after vaccination. Patients remaining on initial treatment were included in the analysis. Possible predictors of persistence of protective antibody response were analysed by using logistic regression analysis.

RESULTS: Of 398 patients participating in the 1.5-year follow up, 302 patients (RA, 163, and SpA, 139) had unchanged medication. Compared with postvaccination levels at 1.5 years, GMLs for each serotype were significantly lower in all groups (P between 0.035 and <0.001; paired-sample t test), as were the proportions of patients with protective antibody levels for both serotypes (P<0.001; chi2 test). Higher prevaccination antibody levels for both serotypes 23F and 6B were associated with better persistence of protective antibodies (P<0.001). Compared with patients with protective antibody levels at 1.5 years, those not having protective antibody levels were older, more often women, had longer disease duration and higher HAQ and DAS, and had a lower proportion of initial responders to both serotypes.

CONCLUSIONS: After initial increase, 1.5 years after pneumococcal vaccination with 7-valent conjugate vaccine, postvaccination antibody levels decreased significantly, reaching levels before vaccination in this cohort of patients with established arthritis treated with different antirheumatic drugs. MTX and anti-TNF treatment predicted low persistence of protective immunity among patients with RA. To boost antibody response, early revaccination with conjugate vaccine might be needed in patients receiving potent immunosuppressive remedies.

TRIAL REGISTRATION NUMBER: EudraCT EU 2007-006539-29 and NCT00828997.
Publication Type
Clinical Trial. Journal Article. Research Support, Non-U.S. Gov't.

Year of Publication
  2013

<443>
Unique Identifier
  23280345
Title
  Synergistic effects of interleukin-1beta and interleukin-17A antibodies on collagen-induced arthritis mouse model.
Source
  International Immunopharmacology. 15(2):199-205, 2013 Feb.
VI 1
Status
  MEDLINE
Authors
  Zhang Y; Ren G; Guo M; Ye X; Zhao J; Xu L; Qi J; Kan F; Liu M; Li D.
Authors Full Name
  Zhang, Yu; Ren, Guiping; Guo, Mo; Ye, Xianlong; Zhao, Jingzhuang; Xu, Liming; Qi, Jianying; Kan, Fangming; Liu, Miao; Li, Deshan.
Institution
  Zhang, Yu. Bio-pharmaceutical Lab, Life Science College, Northeast Agricultural University, Harbin, 150030, China.
Abstract
  Rheumatoid arthritis (RA) is a chronic autoimmune inflammatory disease that mainly causes the synovial joint inflammation and cartilage destruction. Both interleukin-1beta (IL-1beta) and Interleukin-17 (IL-17) are important proinflammatory cytokines involved in the pathogenesis of RA. We investigated whether combination therapy with IL-1beta and IL-17A antibodies would generate the potential for synergistic effects on a collagen-induced arthritis (CIA) mouse model. Mice with CIA were subcutaneously injected with humanized IL-1beta antibody, IL-17A antibody, or combination treatment. The effects of treatment were determined by arthritis severity score, histological damage and bone destruction, autoreactive humoral and cellular immune responses and cytokine production. Treatment with IL-1beta antibody or IL-17A antibody alone resulted in beneficial effects on clinical and histological parameters of CIA mice. Compared with the single

antibody treatments, the combination therapy resulted in a more significant effect in alleviating the severity of arthritis by preventing bone damage and cartilage destruction, reducing humoral and cellular immune responses, and down-regulating the expression of IL-1beta, IL-6, IL-17A, IFN-gamma, RANKL and MMP-3 in inflammatory tissue. In conclusion, combination treatment with humanized IL-1beta and IL-17A antibodies demonstrates synergistic beneficial effects for preventing joint inflammation and cartilage destruction and bone damage in CIA mice model. These studies also provide evidence that combination with IL-1beta and IL-17A antibodies may lead to a new combinatorial therapy for RA patients. Copyright © 2012 Elsevier B.V. All rights reserved.
Publication Type
Journal Article.
Year of Publication
2013

<444>
Unique Identifier
23280233
Title
Presence and role of anti-citrullinated protein antibodies in experimental arthritis models.
Source
Arthritis & Rheumatism. 65(4):939-48, 2013 Apr.
VI 1
Status
MEDLINE
Authors
Cantaert T; Teitsma C; Tak PP; Baeten D.
Authors Full Name
Cantaert, Tineke; Teitsma, Christine; Tak, Paul P; Baeten, Dominique.
Institution
Cantaert, Tineke. Academic Medical Center and University of Amsterdam, Amsterdam, The Netherlands.
Abstract

OBJECTIVE: Anti-citrullinated protein antibodies (ACPAs) are the serologic hallmark of rheumatoid arthritis. Functional studies on the role of ACPAs in experimental arthritis have yielded conflicting results, and therefore the present study was undertaken to assess systematically whether citrullinated proteins can really induce ACPAs and modulate arthritis in mice.

METHODS: Balb/c, SJL, and DBA/1 mice were immunized with either native or citrullinated fibrinogen, myelin basic protein (MBP), and type II collagen (CII). ACPAs were detected with a peptide-based enzyme-linked immunosorbent assay (ELISA) and with Western blotting using fibrinogen as substrate. Arthritis was induced in mice by immunization with CII in Freund's complete adjuvant or by injection of anticollagen antibodies.

RESULTS: Analysis of the sera of mice immunized with citrullinated proteins revealed false-positive results with the citrulline peptide-based ELISA. In contrast, Western blot analysis using either citrullinated or native fibrinogen as substrate reliably detected ACPAs in Balb/c mice immunized with citrullinated fibrinogen, MBP, and CII. However, these ACPAs failed to induce or aggravate disease in Balb/c mice in the anticollagen antibody-induced arthritis model. Immunization with citrullinated fibrinogen induced ACPAs but did not lead to arthritis development in SJL and DBA/1 mice. In contrast, immunization with citrullinated CII failed to induce ACPAs or enhance disease in these strains in the collagen-induced arthritis model.

CONCLUSION: Mice can develop genuine ACPAs, but detection of ACPAs is highly dependent on strain, immunogen, immunization protocol, and detection assay. Murine ACPAs are not overtly pathogenic, since neither preexisting ACPAs nor the use of citrullinated collagen as immunogen modulates the clinical course of arthritis. Copyright © 2013 by the American College of Rheumatology.

Publication Type

Journal Article. Research Support, Non-U.S. Gov't.

Year of Publication

2013

<445>

Unique Identifier

23199973

Title

[Whipple disease revealed by anti-TNFalpha therapy]. [French]

Source

Revue de Medecine Interne. 34(2):105-9, 2013 Feb.

VI 1

Status

MEDLINE

Authors

Sparsa L; Fenollar F; Gossec L; Leone J; Pennaforte JL; Dougados M; Roux C.

Authors Full Name

Sparsa, L; Fenollar, F; Gossec, L; Leone, J; Pennaforte, J-L; Dougados, M; Roux, C.

Institution

Sparsa, L. Service de rhumatologie B, universite Paris-Descartes, hopital Cochin, AP-HP, Paris, France. sparsal@ch-mulhouse.fr

Abstract

INTRODUCTION: Whipple disease is a rare infectious disease with protean clinical manifestations. This infection may mimic chronic inflammatory rheumatisms such as rheumatoid arthritis or spondylarthritis. In this context, introduction of a biotherapy after a diagnostic hesitation does not always lead to early complications. Sometimes, the clinical degradation follows an initial improvement, encouraging continuation of the immunosuppressive treatment and leading consequently to a greater diagnostic delay.

CASE REPORTS: We report two cases of Whipple disease diagnosed in the context of an inflammatory disease with anti-TNFalpha failure. The first patient was a 53-year-old man who presented with an axial and peripheral spondylarthritis who was treated with etanercept and adalimumab. The second was a 42-year-old man who received adalimumab and then etanercept for a peripheral spondylarthritis.

CONCLUSION: Whipple disease should be suspected in all patients who present with a chronic inflammatory rheumatism that is partially or not controlled with anti-TNFalpha therapy and who had persisting elevated acute phase reactants. Copyright © 2012 Societe nationale francaise de medecine interne (SNFMI). Published by Elsevier SAS. All rights reserved.

Publication Type

Case Reports. English Abstract. Journal Article.

Year of Publication

2013

<446>

Unique Identifier

23092365

Title

Allopurinol hypersensitivity reactions: desensitization strategies and new therapeutic alternative molecules.

Source

Inflammation & Allergy Drug Targets. 12(1):19-28, 2013 Feb.

VI 1

Status

MEDLINE

Authors

Calogiuri G; Nettis E; Di Leo E; Foti C; Ferrannini A; Butani L.

Authors Full Name

Calogiuri, Gianfranco; Nettis, Eustachio; Di Leo, Elisabetta; Foti, Caterina; Ferrannini, Antonio; Butani, Lavjay.

Institution

Calogiuri, Gianfranco. Pneumology Department - Hospital Ninetto Melli-S. Pietro Vernotico, Brindisi, Italy. gf.calogiuri@libero.it

Abstract

Allopurinol, an analog of hypoxanthine has been worldwide used for the treatment of hyperuricemia and gout for over 40 years. Unfortunately some patients assuming this medication have developed hypersensitivity reactions ranging from mild cutaneous eruption to more severe clinical manifestations such as allopurinol hypersensitivity syndrome or Steven-Johnson syndrome and lethal toxic epidermal necrolysis. Various strategies of slow desensitization have been elaborated to reintroduce allopurinol in a part of these patients, mainly patients affected by mild skin reactions as fixed drug eruption or exanthema. However, several new uricosuric therapies have been recently introduced. Actually drugs as recombinant urate oxidase and febuxostat are under post-marketing surveillance to control potential adverse effects related to their immunogenicity even.

Publication Type

Journal Article.

Year of Publication

2013

<447>

Unique Identifier

23064976

Title

Rituximab-treated patients have a poor response to influenza vaccination.

Source

Journal of Clinical Immunology. 33(2):388-96, 2013 Feb.

VI 1

Status

MEDLINE

Authors

Eisenberg RA; Jawad AF; Boyer J; Maurer K; McDonald K; Prak ET; Sullivan KE.

Authors Full Name

Eisenberg, Robert A; Jawad, Abbas F; Boyer, Jean; Maurer, Kelly; McDonald, Kenyetta; Prak, Eline T Luning; Sullivan, Kathleen E.

Institution

Eisenberg, Robert A. Division of Rheumatology, The Perelman School of Medicine at the University of Pennsylvania, Philadelphia, PA, USA.

Abstract

The efficacy of influenza vaccination in patients treated with rituximab is a clinically important question. Rheumatology clinics are populated with patients receiving rituximab for a broad array of disorders. Although several studies have explored the efficacy of other vaccines in rituximab-treated populations, results have been conflicting. We wished to define influenza vaccine efficacy in a rituximab-treated cohort. We examined 17 evaluable subjects treated with rituximab for rheumatologic conditions. T cell subsets, B cells subsets, T cell function, and B cell function were evaluated at specific time points along with hemagglutinination inhibition titers after receiving the standard inactivated influenza vaccine. T cell subset counts were significantly different than controls but did not change with rituximab. B cells depleted in all patients but were in various stages of recovery at the time of vaccination. Influenza vaccine responsiveness was poor overall, with only 16 % of subjects having a four-fold increase in titer. Pre-existing titers were retained

throughout the study, however. The ability to respond to the influenza vaccine appeared to be related to the degree of B cell recovery at the time of vaccination. This study emphasizes that antibody responses to vaccine are impaired in subjects treated with rituximab and supports the concept that B cell recovery influences influenza vaccine responsiveness.

Publication Type

Journal Article. Research Support, N.I.H., Extramural. Research Support, Non-U.S. Gov't.

Year of Publication

2013

<448>

Unique Identifier

23063342

Title

Injection site reaction to adalimumab: Positive skin test and successful rapid desensitisation.

Source

Allergologia et Immunopathologia. 41(3):204-6, 2013 May-Jun.

VI 1

Status

MEDLINE

Authors

Bavbek S; Ataman S; Bankova L; Castells M.

Authors Full Name

Bavbek, S; Ataman, S; Bankova, L; Castells, M.

Publication Type

Case Reports. Letter.

Year of Publication

2013

<449>

Unique Identifier

23054859

Title

Incomplete Kawasaki disease followed by systemic onset juvenile idiopathic arthritis- the diagnostic dilemma.

Source

Indian Journal of Pediatrics. 80(9):783-5, 2013 Sep.

VI 1

Status

MEDLINE

Authors

Dogra S; Gehlot A; Suri D; Rawat A; Kumar RM; Singh S.

Authors Full Name

Dogra, Shivani; Gehlot, Arushi; Suri, Deepti; Rawat, Amit; Kumar, Rohit Manoj; Singh, Surjit.

Institution

Dogra, Shivani. Department of Pediatrics, Advanced Pediatrics Centre, Post Graduate Institute of Medical Education and Research, Chandigarh, India.

Abstract

The authors report an 18-mo-old girl who presented with features of incomplete Kawasaki disease and was refractory to intravenous immunoglobulin and infliximab treatment. She subsequently responded to pulse intravenous methylprednisolone therapy. The diagnostic dilemma arose after 2 mo when she developed clinical features suggestive of systemic onset juvenile idiopathic arthritis. Since both diseases have overlapping clinical features and no specific diagnostic laboratory tests, it is difficult for the clinicians even in the best of centers to reach a definitive diagnosis as illustrated by the index case.

Publication Type

Case Reports. Journal Article.

Year of Publication

2013

<450>

Unique Identifier

23041839

Title

C57BL/6 mice need MHC class II Aq to develop collagen-induced arthritis dependent on autoreactive T cells.

Source

Annals of the Rheumatic Diseases. 72(7):1225-32, 2013 Jul.

VI 1

Status

MEDLINE

Authors

Backlund J; Li C; Jansson E; Carlsen S; Merky P; Nandakumar KS; Haag S; Ytterberg J; Zubarev RA; Holmdahl R.

Authors Full Name

Backlund, Johan; Li, Cuiqin; Jansson, Erik; Carlsen, Stefan; Merky, Patrick; Nandakumar, Kutty-Selva; Haag, Sabrina; Ytterberg, Jimmy; Zubarev, Roman A; Holmdahl, Rikard.

Institution

Backlund, Johan. Medical Inflammation Research, Department of Biochemistry and Biophysics, Karolinska Institute, Stockholm, Sweden. johan.backlund@ki.se

Abstract

INTRODUCTION: Collagen-induced arthritis (CIA) has traditionally been performed in MHC class II A(q)-expressing mice, whereas most genetically modified mice are on the C57BL/6 background (expressing the b haplotype of the major histocompatibility complex (MHC) class II region). However, C57BL/6 mice develop arthritis after immunisation with chicken-derived collagen type II (CII), but arthritis susceptibility has been variable, and the immune specificity has not been clarified.

OBJECTIVE: To establish a CIA model on the C57BL/6 background with a more predictable and defined immune response to CII.

RESULTS: Both chicken and rat CII were arthritogenic in C57BL/6 mice provided they were introduced with high doses of Mycobacterium tuberculosis adjuvant. However, contaminating pepsin was strongly immunogenic and was essential for arthritis development. H-2(b)-restricted T cell epitopes on chicken or rat CII could not be identified, but expression of A(q) on the C57BL/6 background induced T cell response to the CII260-270 epitope, and also prolonged the arthritis to be more chronic.

CONCLUSIONS: The putative (auto)antigen and its arthritogenic determinants in C57BL/6 mice remains undisclosed, questioning the value of the model for addressing T cell-driven pathological

pathways in arthritis. To circumvent this impediment, we recommend MHC class II congenic C57BL/6N.Q mice, expressing A(q), with which T cell determinants have been thoroughly characterised.

Publication Type

Journal Article. Research Support, Non-U.S. Gov't.

Year of Publication

2013

<451>

Unique Identifier

23002006

Title

Efficacy of B cell depletion therapy for murine joint arthritis flare is associated with increased lymphatic flow.

Source

Arthritis & Rheumatism. 65(1):130-8, 2013 Jan.

VI 1

Status

MEDLINE

Authors

Li J; Ju Y; Bouta EM; Xing L; Wood RW; Kuzin I; Bottaro A; Ritchlin CT; Schwarz EM.

Authors Full Name

Li, Jie; Ju, Yawen; Bouta, Echoe M; Xing, Lianping; Wood, Ronald W; Kuzin, Igor; Bottaro, Andrea; Ritchlin, Christopher T; Schwarz, Edward M.

Institution

Li, Jie. University of Rochester School of Medicine and Dentistry, University of Rochester Medical Center, Rochester, New York 14642, USA.

Abstract

OBJECTIVE: B cell depletion therapy ameliorates rheumatoid arthritis by mechanisms that are incompletely understood. Arthritis flare in tumor necrosis factor (TNF)-transgenic mice is associated with efferent lymph node (LN) "collapse," triggered by B cell translocation into lymphatic spaces and decreased lymphatic drainage. The aim of this study was to examine

whether the efficacy of B cell depletion therapy is associated with restoration of lymphatic drainage due to removal of obstructing nodal B cells.

METHODS: We used contrast-enhanced magnetic resonance imaging, indocyanine green near-infrared imaging, and intravital immunofluorescence imaging to longitudinally assess synovitis, lymphatic flow, and cell migration in lymphatic vessels in TNF-transgenic mice. We conducted tests to determine whether the efficacy of B cell depletion therapy is associated with restoration of lymphatic draining and cell egress from arthritic joints.

RESULTS: Unlike active lymphatics to normal and prearthritic knees, afferent lymphatic vessels to collapsed LNs in inflamed knees do not pulse. Intravital immunofluorescence imaging demonstrated that CD11b+ monocyte/macrophages in lymphatic vessels afferent to expanding LNs travel at high velocity (mean+/-SD 186+/-37 mum/second), while these cells are stationary in lymphatic vessels afferent to collapsed popliteal LNs. B cell depletion therapy for arthritis flares in TNF-transgenic mice significantly decreased knee synovium volume (by 50% from the baseline level) and significantly increased lymphatic clearance compared with placebo (P<0.05). This increased lymphatic drainage restored macrophage egress from inflamed joints without recovery of the lymphatic pulse.

CONCLUSION: These results support a novel mechanism in which B cell depletion therapy for joint arthritis flares lessens inflammation by increasing lymphatic drainage and subsequent migration of cells and cytokines from the synovial space. Copyright © 2013 by the American College of Rheumatology.
Publication Type
Journal Article. Research Support, N.I.H., Extramural.
Year of Publication
2013

<452>
Unique Identifier
22843486
Title
Targeted delivery of cytokine therapy to rheumatoid tissue by a synovial targeting peptide.

Source

Annals of the Rheumatic Diseases. 72(1):129-35, 2013 Jan.

VI 1

Status

MEDLINE

Authors

Wythe SE; DiCara D; Taher TE; Finucane CM; Jones R; Bombardieri M; Man YK; Nissim A; Mather SJ; Chernajovsky Y; Pitzalis C.

Authors Full Name

Wythe, Sarah E; DiCara, Danielle; Taher, Taher E I; Finucane, Ciara M; Jones, Rita; Bombardieri, Michele; Man, Y K Stella; Nissim, Ahuva; Mather, Stephen J; Chernajovsky, Yuti; Pitzalis, Costantino.

Institution

Wythe, Sarah E. William Harvey Research Institute, Barts and The London School of Medicine and Dentistry, London, UK.

Abstract

OBJECTIVES: The synovial endothelium targeting peptide (SyETP) CKSTHDRLC has been identified previously and was shown to preferentially localise to synovial xenografts in the human/severe combined immunodeficient (SCID) mouse chimera model of rheumatoid arthritis (RA). The objective of the current work was to generate SyETP-anti-inflammatory-cytokine fusion proteins that would deliver bioactive cytokines specifically to human synovial tissue.

METHODS: Fusion proteins consisting of human interleukin (IL)-4 linked via a matrix metalloproteinase (MMP)-cleavable sequence to multiple copies of either SyETP or scrambled control peptide were expressed in insect cells, purified by Ni-chelate chromatography and bioactivity tested in vitro. The ability of SyETP to retain bioactive cytokine in synovial but not control skin xenografts in SCID mice was determined by in vivo imaging using nano-single-photon emission computed tomography-computed tomography (nano-SPECT-CT) and measuring signal transducer and activator of transcription 6 (STAT6) phosphorylation in synovial grafts following intravenous administration of the fusion protein.

RESULTS: In vitro assays confirmed that IL-4 and the MMP-cleavable sequence were functional. IL-4-SyETP augmented production of IL-1 receptor antagonist (IL-1ra) by fibroblast-like synoviocytes (FLS) stimulated with IL-1beta in a dose-dependent manner. In vivo imaging showed that IL-4-SyETP was retained in synovial but not in skin tissue grafts and the period of retention was significantly enhanced through increasing the number of SyETP copies from one to

three. Finally, retention correlated with increased bioactivity of the cytokine as quantified by STAT6 phosphorylation in synovial grafts.

CONCLUSIONS: The present work demonstrates that SyETP specifically delivers fused IL-4 to human rheumatoid synovium transplanted into SCID mice, thus providing a proof of concept for peptide-targeted tissue-specific immunotherapy in RA. This technology is potentially applicable to other biological treatments providing enhanced potency to inflammatory sites and reducing systemic toxicity.

Publication Type

Journal Article. Research Support, Non-U.S. Gov't.

Year of Publication

2013

<453>

Unique Identifier

22786533

Title

Prevalence and pharmacological modulation of humoral immunity to AAV vectors in gene transfer to synovial tissue.

Source

Gene Therapy. 20(4):417-24, 2013 Apr.

VI 1

Status

MEDLINE

Authors

Mingozzi F; Chen Y; Edmonson SC; Zhou S; Thurlings RM; Tak PP; High KA; Vervoordeldonk MJ.

Authors Full Name

Mingozzi, F; Chen, Y; Edmonson, S C; Zhou, S; Thurlings, R M; Tak, P P; High, K A; Vervoordeldonk, M J.

Institution

Mingozzi, F. Center of Cellular and Molecular Therapeutics, The Children's Hospital of Philadelphia, Philadelphia, PA 19104, USA. mingozzi@email.chop.edu

Abstract
Antibodies against adeno-associated viral (AAV) vectors are highly prevalent in humans. Both preclinical and clinical studies showed that antibodies against AAV block transduction even at low titers, particularly when the vector is introduced into the bloodstream. Here we measured the neutralizing antibody (NAb) titer against AAV serotypes 2, 5, 6 and 8 in the serum and matched synovial fluid (SF) from rheumatoid arthritis patients. The titer in the SF was lower than that in the matched plasma samples, indicating a difference in distribution of NAb to AAV depending on the body fluid compartment. This difference was more evident for AAV2, against which higher titers were measured. Of all serotypes, anti-AAV5 antibodies were the least prevalent in both the serum and SF. We next evaluated the impact of B-cell depletion on anti-AAV antibodies in rheumatoid arthritis patients who received one or two courses of the anti-CD20 antibody rituximab as part of their disease management. A drop of NAb titer was observed in a subset of those subjects carrying NAb titers <=1:1000; however, only in a minority of subjects titers dropped below 1:5. This work provides insights into strategies to overcome the limitation of pre-existing humoral immunity to AAV vectors.
Publication Type
Journal Article. Research Support, N.I.H., Extramural. Research Support, Non-U.S. Gov't.
Year of Publication
2013

<454>
Unique Identifier
22441967
Title
Vaccine history knowledge of subcutaneous anti-TNF receiving rheumatology patients.
Source
Rheumatology International. 33(7):1907-8, 2013 Jul.
VI 1
Status
MEDLINE
Authors
Nolan TJ; O'Connor MB; Bond U; Swan J; Phelan MJ.
Authors Full Name

Nolan, T J; O'Connor, Mortimer B; Bond, Ursula; Swan, Joan; Phelan, Mark J.
Publication Type
Letter.
Year of Publication
2013

<455>
Unique Identifier
25486725
Title
[Vaccination in chronic autoimmune and inflammatory rheumatic diseases]. [Croatian]
Source
Reumatizam. 59(1):28-35, 2012.
VI 1
Status
MEDLINE
Authors
Krstulovic DM; Kaliterna DM.
Authors Full Name
Krstulovic, Daniela Marasovic; Kaliterna, Dusanka Martinovic.
Abstract
Vaccine preventable infections occur more often in patients with autoimmune and chronic rheumatic diseases when compared to the general population. Most vaccines are immunogenic, efficacious and safe in those patients, even with immunosuppressive treatment, excluding rituximab. Live attenuated vaccines should be avoided in patients receiving long-term immunosuppressive therapy. The occurrence of adverse events and autoimmune phenomena is almost the same in vaccinated patients with rheumatic diseases and in vaccinated healthy individuals. A deeper research is needed regarding safety of vaccination and the influence of new immunomodulating drugs on efficacy of vaccination since the studies that were undertaken so far are retrospective or performed on small cohorts of patients. Evidence-based recommendations for vaccination in adult patients with autoimmune inflammatory rheumatic diseases developed by European League Against Rheumatism (EULAR) will help rheumatologists in making a decision about vaccinating their patients.

Publication Type
  Journal Article.
Year of Publication
  2012

<456>
Unique Identifier
  23431528
Title
  Orthopedic surgery is possible in hemophilic patients with inhibitors. [Review]
Source
  American Journal of Orthopedics (Chatham, Nj). 41(12):570-4, 2012 Dec.
VI 1
Status
  MEDLINE
Authors
  Rodriguez-Merchan EC.
Authors Full Name
  Rodriguez-Merchan, E Carlos.
Institution
  Rodriguez-Merchan, E Carlos. Department of Orthopaedic Surgery, La Paz University Hospital, Madrid, Spain. ecrmerchan@gmx.es
Abstract
  Hemophilia is an inherited recessive sex-linked bleeding disorder. An insufficiency of coagulation factor VIII produces hemophilia A, and lack of factor IX causes hemophilia B. Prevention and management of the disease require intravenous infusion of the deficient factor. Worldwide, hemophilia affects approximately 600,000 people, 20% of whom develop antibodies against the deficient coagulation factor. Hemophilic patients with inhibitors present with multiarticular joint degeneration (hemophilic arthropathy) secondary to recurrent hemarthroses. The availability of activated prothrombin complex concentrates and activated recombinant factor VII allows hemophilic patients with high inhibitor titers to undergo elective orthopedic surgery with a high expectation of success, and thorough individual case analysis by a multidisciplinary team

allows surgeons to obtain satisfactory results. However, the rate of potential complications must not be underestimated.

Publication Type

Journal Article. Review.

Year of Publication

2012

<457>

Unique Identifier

23285480

Title

Association between the response to B cell depletion therapy and the allele*2 of the HS1,2A enhancer in seropositive rheumatoid arthritis patients.

Source

Reumatismo. 64(6):368-73, 2012 Dec 20.

VI 1

Status

MEDLINE

Authors

Canestri S; Totaro MC; Serone E; Tolusso B; Frezza D; Gremese E; Ferraccioli G.

Authors Full Name

Canestri, S; Totaro, M C; Serone, E; Tolusso, B; Frezza, D; Gremese, E; Ferraccioli, G.

Institution

Canestri, S. Division of Rheumatology, Institute of Rheumatology and Affine Sciences, Catholic University of Sacred Heart, Rome, Italy.

Abstract

OBJECTIVE: Several studies underline the relevance of the genetic background for the response to therapy. We evaluated the relationship between the polymorphism of the HS1,2A enhancer, located in the 3' regulatory region of the heavy immunoglobulin chain (IgH), and the response to B cell depletion therapy (BCDT) with Rituximab (RTX).

METHODS: Fifty rheumatoid arthritis (RA) patients (42 women; disease duration 13.9 +/- 10.6 years) treated with RTX, not responsive to previous DMARDs and/or TNFalpha inhibitors

therapies, and 220 healthy subjects were enrolled in the study. Patients were genotyped for HS1,2A enhancer polymorphism, as previously described. Disease activity was assessed every three months according to the European League Against Rheumatism's (EULAR) criteria.

RESULTS: All RA patients were seropositive for at least one of the tested autoantibodies: rheumatoid factor (FR IgA, FR IgM e FR IgG), anti-cyclic citrullinated peptides (anti-CCP IgA, anti-CCP IgM e anti-CCP IgG) and anti-vimentin antibodies. RA patients had an increased frequency of the allele*2 (60.0%) of the HS1,2A enhancer compared to healthy subjects (42.0%; OR(95%ICs): 2.07 (1.33-3.22)). Patients with a good EULAR response at 6 months follow-up visit had an increased frequency of genotype 2/2 (47.1%) compared to poor-responders RA patients (genotype 2/2: 18.2%, OR(95%ICs): 4.00 (1.09-14.68)). All the patients with a good EULAR response had the allele*2, thus showing a possible association with the allele in this population.

CONCLUSIONS: The presence of allele*2 seems to be related to a good response to BCDT with RTX in seropositive RA patients, thus highlighting the role of the HS1,2A enhancer in B cell maturation and class-switch recombination.

Publication Type

Journal Article.

Year of Publication

2012

<458>

Unique Identifier

23232510

Title

[Immunization with 2nd extracellular loop peptide of muscarinic acetylcholine 3 receptor induces the secretion of IL-17 and IFN-gamma in NOD-scid mice]. [Chinese]

Source

Xibao Yu Fenzi Mianyixue Zazhi. 28(12):1233-6, 2012 Dec.

VI 1

Status

MEDLINE

Authors

Yang L; Ju JZ; Lu FF; Zhang W; Pang CY; Wang YF.
Authors Full Name
Yang, Lin; Ju, Jin-zhe; Lu, Feng-feng; Zhang, Wei; Pang, Chun-yan; Wang, Yong-fu.
Institution
Yang, Lin. Inner Mongolia Key Laboratory of Autoimmunity, Institution of Immunology and Rheumatism, Baotou Medical College, Inner Mongolia University of Science and Technology, Baotou 014010, China.
Abstract
AIM: To evaluate whether or not the changes in the secretions of IL-17 and IFN-gamma can be induced by the immunization with 2nd extracellular loop peptide of muscarinic acetylcholine 3 receptor (M3R) in NOD-scid (nonobese diabetic-severe combined immunodeficiency) mice.

METHODS: We synthesized the 2nd extracellular loop peptide of M3R and immunized NOD-scid mice subcutaneously with the 1:1 mixture of the peptide and the incomplete Freund's adjuvant (IFA). At day 1, 7, 14, 21 after immunization, tail blood samples were taken to determine the antibody titer and evaluate the secretions of IL-17 and IFN-gamma in sera. Meanwhile, we recorded the fluid intake amount per mouse every week. At day 21, all of the NOD-scid mice were killed to measure the concentrations of IL-17 and IFN-gamma in cell supernatants. Immunofluorescence staining of lacrimal glands was performed to observe the changes in the secretions of IL-17 and IFN-gamma.

RESULTS: Compared with the control group, the sera titers of anti-2nd extracellular loop peptide antibodies were significantly higher in 2nd extracellular loop peptide immunized NOD-scid mice at day 14 ($P<0.05$). The concentrations of IL-17 and IFN-gamma increased significantly in sera of the 2nd extracellular loop peptide immunized NOD-scid mice at day 7 and 14 ($P<0.01$). The concentration of IL-17 maintained at a certain level in the supernatants of spleen cells co-cultured with 2nd extracellular loop peptide, while it decreased significantly in the control groups ($P<0.01$). Immunofluorescence staining demonstrated that the production of IL-17 and IFN-gamma increased in the lacrimal glands of NOD-scid mice immunized with the 2nd extracellular loop peptide. However, no changes in fluid intake was observed in NOD-scid mice immunized with the 2nd extracellular loop peptide($P>0.05$).

CONCLUSION: Immunization with 2nd extracellular loop peptide of M3R can induce the production of anti-2nd extracellular loop peptide antibodies and the secretions of IL-17 and IFN-gamma in NOD-scid mice.
Publication Type
Journal Article. Research Support, Non-U.S. Gov't.

Year of Publication
  2012

<459>
Unique Identifier
  23194358
Title
  Conference scene: antigen-presenting cells: different approaches to immune induction and tolerance.
Source
  Immunotherapy. 4(11):1095-7, 2012 Nov.
VI 1
Status
  MEDLINE
Authors
  Nicolaou N.
Authors Full Name
  Nicolaou, Nectarios.
Abstract
  APCs are key players in the induction of T-cell responses. Accordingly, they have gained much attention as therapeutic targets for inflammatory diseases and cancer. Understanding their mode of action could serve in revealing novel approaches for the induction and blockade of T-cell immunity. This meeting has focused on recent advances in inducing the tolerogenic and immunostimulatory function of dendritic cells as well as current progress in the understanding of dendritic cell suppression through the CD4(+) Treg cell function.
Publication Type
  Congress. News.
Year of Publication
  2012

<460>

Unique Identifier

23167679

Title

Glucocorticoid effects on skeletal muscle: benefit and risk in patients with autoimmune inflammatory rheumatoid diseases.

Source

Expert Review of Clinical Immunology. 8(8):695-7, 2012 Nov.

VI 1

Status

MEDLINE

Authors

Hanaoka BY; Peterson CA; Crofford LJ.

Authors Full Name

Hanaoka, Beatriz Y; Peterson, Charlotte A; Crofford, Leslie J.

Publication Type

Editorial.

Year of Publication

2012

<461>

Unique Identifier

23137751

Title

Hearing loss in fibromyalgia? Somatic sensory and non-sensory symptoms in patients with fibromyalgia and other rheumatic disorders.

Source

Clinical & Experimental Rheumatology. 30(6 Suppl 74):88-93, 2012 Nov-Dec.

VI 1

Status

MEDLINE

Authors

Wolfe F; Rasker JJ; Hauser W.
Authors Full Name
Wolfe, Frederick; Rasker, Johannes J; Hauser, Winfried.
Institution
Wolfe, Frederick. National Data Bank for Rheumatic Diseases and University of Kansas School of Medicine, Wichita, KS, USA. fwolfe@arthritis-research.org
Abstract
OBJECTIVES: It has been proposed that fibromyalgia can be understood as a disorder of central sensitisation and dysregulation (CD) and that characteristic somatic symptoms are the result of 'central augmentation'. We examined this hypothesis by analysing sensory and non-sensory variables in the context of the updated (2010) American College of Rheumatology definition of fibromyalgia and the fibromyalgianess (polysymptomatic distress) scale.

METHODS: We studied 11,288 patients, including those with fibromyalgia, rheumatoid arthritis (RA) and osteoarthritis (OA). We divided somatic symptoms into sensory (hearing difficulties) and evaluative (easy bruising and hair loss) non-sensory symptoms, and included a non-symptom that was neutral as to psychological content or meaning (influenza vaccination). Data were analysed by logistic regression and adjusted for age and sex.

RESULTS: Fibromyalgia patients reported more sensory and non-sensory symptoms than patients with RA and OA, but not more non-symptoms. At all levels of fibromyalgianess (or fibromyalgia intensity) the probability of sensory and non-sensory symptoms was similar across all rheumatic diseases, and this association occurred in FM criteria (+) and criteria (-) patients. No association was noted with the non-symptom control question.

CONCLUSIONS: While the CD hypothesis is consistent with hearing problems in fibromyalgia, there is no medical explanation for the evaluative symptoms of hair loss and bruising being increased. The associations between fibromyalgia/fibromyalgianess and evaluative (not sensory) symptoms must occur through mechanisms other than central sensitization and augmentation, and are consistent with over-reporting that has a psychological basis. However, augmentation of sensory symptoms does not preclude simultaneous over-reporting.
Publication Type
Comparative Study. Journal Article.
Year of Publication
2012

<462>

Unique Identifier

23137579

Title

Infections and biologic therapy in rheumatoid arthritis: our changing understanding of risk and prevention. [Review]

Source

Rheumatic Diseases Clinics of North America. 38(4):727-45, 2012 Nov.

VI 1

Status

MEDLINE

Authors

Winthrop KL.

Authors Full Name

Winthrop, Kevin L.

Institution

Winthrop, Kevin L. Public Health and Preventive Medicine, Oregon Health & Science University, Portland, OR, USA. winthrop@ohsu.edu

Abstract

Patients with rheumatoid arthritis are at higher risk for serious infections and death from infection than the general public. Prednisone and biologic agents increase this risk, although the risk associated with biologics can be mitigated when such agents act as prednisone-sparing therapies. Some of the important causes of infectious morbidity in this setting are preventable with screening (eg, tuberculosis) or vaccination (eg, herpes zoster). Copyright © 2012 Elsevier Inc. All rights reserved.

Publication Type

Journal Article. Review.

Year of Publication

2012

<463>

Unique Identifier

23129076

Title

Common variable immunodeficiency presenting with persistent parvovirus B19 infection.

Source

Pediatrics. 130(6):e1711-5, 2012 Dec.

VI 1

Status

MEDLINE

Authors

Adams ST; Schmidt KM; Cost KM; Marshall GS.

Authors Full Name

Adams, Sarah T M; Schmidt, Kara M; Cost, Karen M; Marshall, Gary S.

Institution

Adams, Sarah T M. Department of Pediatrics, University of Louisville School of Medicine, Louisville, Kentucky, Louisville, KY 40202, USA.

Abstract

Parvovirus B19 infection in healthy hosts is self-limited, but persistent infection has been described in patients with cellular immune defects. A 6-year-old boy presented with a 6-month history of weight loss and malaise and a 1-month history of fever and polyarticular arthritis. Parvovirus DNA was detected in plasma at 10 300 copies/mL. Levels of immunoglobulin (Ig)G, IgA, IgM, IgG-1, and IgG-2 were low, and antibody responses to vaccine antigens were impaired. HIV antibody and DNA polymerase chain reaction were negative, and the patient had normal immunophenotype, mitogen stimulation response, CD40 ligand and inducible costimulator expression, transmembrane activator and CAML interactor sequencing, genomic analysis, and fluorescent in situ hybridization for deletions at 22q11.2. Common variable immunodeficiency was diagnosed and replacement therapy with immune globulin intravenous was initiated. The parvovirus DNA level declined by half over 3 months and was undetectable at 15 months. Constitutional symptoms improved but arthritis persisted and eosinophilic fasciitis eventually developed. This case demonstrates that persistent parvovirus infection may be a presenting feature of humoral immune deficiency and can mimic juvenile rheumatoid arthritis. The infection may respond to immune globulin intravenous therapy.

Publication Type

Case Reports. Journal Article.

Year of Publication

2012

<464>

Unique Identifier

23124404

Title

Infections in biological agents used in rheumatic disease. [Review]

Source

British Journal of Hospital Medicine. 73(9):517-20, 2012 Sep.

VI 1

Status

MEDLINE

Authors

Byng-Maddick R; Ehrenstein M.

Authors Full Name

Byng-Maddick, Rachel; Ehrenstein, Michael.

Institution

Byng-Maddick, Rachel. Rheumatology Department, University College, London Hospitals NHS Foundation Trust, London, UK. r.byng-maddick@ucl.ac.uk

Abstract

Immunosuppression and biological therapies are being used exponentially to treat inflammatory arthritis and connective tissue diseases. This article discusses the potential infectious complications of these therapies.

Publication Type

Journal Article. Review.

Year of Publication

2012

<465>

Unique Identifier

23094578

Title

[Vaccinations in patients with autoimmune inflammatory rheumatic diseases--EULAR recommendations for pediatric and adult patients]. [Review] [German]

Source

Medizinische Monatsschrift fur Pharmazeuten. 35(10):353-64; quiz 365-6, 2012 Oct.

VI 1

Status

MEDLINE

Authors

Muller-Ladner C; Muller-Ladner U; European League Against Rheumatism.

Authors Full Name

Muller-Ladner, Claudia; Muller-Ladner, Ulf; European League Against Rheumatism.

Institution

Muller-Ladner, Claudia. Justus-Liebig Universitat Giessen, Abt. Rheumatologie und klinische Immunologie, Kerckhoff Klinik Bad Nauheim, Benekestrasse 2, 61231 Bad Nauheim.

Abstract

Since patients with autoimmune inflammatory rheumatic diseases are prone to infectious complications--on one hand due to the rheumatic disease itself, on the other hand due to the immunosuppressive therapy--vaccination is an essential tool to prevent these infectious complications. Although there exist several recommendations for the vaccination of immunocompromised patients, many questions still remain for the distinct clinical situations of patients with autoimmune inflammatory rheumatic diseases. In addition, there are several questions concerning the safety and efficacy of various vaccinations, especially with regard to live-attenuated vaccines. Therefore, EULAR (European League Against Rheumatism) assembled two expert panels to clarify as much of these clinical problems as possible. After extensive literature review and evidence grading, the expert panels published recommendations for the vaccination of adult and pediatric patients, which are outlined in this review article.

Publication Type

English Abstract. Guideline. Journal Article. Review.

Year of Publication

2012

<466>

Unique Identifier

23092552

Title

A bispecific antibody against IL-1beta and IL-17A is beneficial for experimental rheumatoid arthritis.

Source

International Immunopharmacology. 14(4):770-8, 2012 Dec.

VI 1

Status

MEDLINE

Authors

Qi J; Kan F; Ye X; Guo M; Zhang Y; Ren G; Li D.

Authors Full Name

Qi, Jianying; Kan, Fangming; Ye, Xianlong; Guo, Mo; Zhang, Yu; Ren, Guiping; Li, Deshan.

Institution

Qi, Jianying. Northeast Agricultural University, School of Life Science, No 59 Mucai Street, Xiangfang District, 150030 Harbin, Heilongjiang, China.

Abstract

IL-1beta is a pivotal cytokine and plays an important role in rheumatoid arthritis (RA). More recently, the biological therapy targeting this cytokine has been impressively effective for many RA patients, however, it remains insufficient in some patients. One of the reasons for these failures may be due to multiple cytokines involved in the disease process. In the present study, we constructed a single-chain bispecific antibody (scBsAb1/17) against both human IL-1beta and human IL-17A which is the mediator for several key cytokines involved in the RA process such as TNF- and IL-6. A number of in vitro assays demonstrated that scBsAb1/17 simultaneously bound to both targets with a similar antigen-binding affinity as an individual single-chain antibody molecule (anti-IL-1beta scFv or anti-IL-17A scFv). Mice with collagen-induced arthritis (CIA) were administrated with either scBsAb1/17 or individual single chain antibody alone, and we noticed that treatment with scBsAb1/17 significantly ameliorated clinical signs and alleviated histological lesion of CIA mice compared to treatments with anti-IL-1beta scFv or anti-IL-17A scFv alone. Production of CII-specific antibodies in scBsAb1/17-treated CIA mice was substantially lower than that of single-chain antibody-treated CIA mice. In addition, scBsAb1/17 was more potent in the inhibition of collagen-specific proliferation of splenocytes and mRNA expression of TNF-, IL-6, IL-2, IL-1beta and IFN-gamma in the spleens of CIA mice compared to a single-chain antibody alone. These results suggest that scBsAb1/17 appears more beneficial in CIA mice than monovalent single-chain antibody molecules. Copyright © 2012 Elsevier B.V. All rights reserved.

Publication Type
  Journal Article.
Year of Publication
  2012

<467>
Unique Identifier
  23045254
Title
  The multiple facets of glucocorticoid action in rheumatoid arthritis. [Review]
Source
  Nature Reviews Rheumatology. 8(11):645-55, 2012 Nov.
VI 1
Status
  MEDLINE
Authors
  Baschant U; Lane NE; Tuckermann J.
Authors Full Name
  Baschant, Ulrike; Lane, Nancy E; Tuckermann, Jan.
Institution
  Baschant, Ulrike. Leibniz Institute for Age Research, Fritz Lipmann Institute, Beutenbergstrase 11, D-07745 Jena, Germany.
Abstract
  Glucocorticoids have potent anti-inflammatory effects and have been used to treat patients with rheumatoid arthritis for more than 60 years. However, severe adverse effects of glucocorticoid treatment, including loss of bone mass and increased risk of fractures, are common. Data from studies of glucocorticoid-mediated gene regulation, which utilized conditional knockout mice in animal models of arthritis or glucocorticoid-induced osteoporosis, have substantially increased our understanding of the mechanisms by which glucocorticoids act via the glucocorticoid receptor. Following glucocorticoid binding, the receptor regulates gene expression either by interacting with DNA-bound transcription factors as a monomer or by binding directly to DNA as a dimer. In contrast to the old hypothesis that transrepression mechanisms involving monomeric glucocorticoid receptor actions were responsible for the anti-inflammatory effects of

glucocorticoids, whereas dimeric glucocorticoid receptor binding resulted in adverse effects, data from animal models have shown that the anti-inflammatory and adverse effects of glucocorticoids are mediated by both monomeric and dimeric glucocorticoid receptor binding. This improved knowledge of the molecular mechanisms that underlie the beneficial and adverse effects of glucocorticoid therapy might lead to the development of rationales for novel glucocorticoid receptor ligands that could potentially have anti-inflammatory efficacy without adverse effects on bone.

Publication Type

Journal Article. Research Support, N.I.H., Extramural. Research Support, Non-U.S. Gov't. Review.

Year of Publication

2012

<468>

Unique Identifier

23027029

Title

Immunology: Zoster vaccine and biologic agents: time to question a paradigm?.

Source

Nature Reviews Rheumatology. 8(11):636-8, 2012 Nov.

VI 1

Status

MEDLINE

Authors

Bongartz T; Orenstein R.

Authors Full Name

Bongartz, Tim; Orenstein, Robert.

Publication Type

News.

Year of Publication

2012

<469>

Unique Identifier

23023360

Title

Female with rash, acute kidney failure and rheumatoid arthritis.

Source

Journal of Postgraduate Medicine. 58(3):217-20, 2012 Jul-Sep.

VI 1

Status

MEDLINE

Authors

Atlani M; Gandhi P; Gulwani H; Kaur S.

Authors Full Name

Atlani, M; Gandhi, P; Gulwani, H; Kaur, S.

Institution

Atlani, M. Department of Nephrology, Bhopal Memorial Hospital and Research Centre, Bhopal, Madhya Pradesh, India.

Abstract

This case describes a 42-year-old female with longstanding history of rheumatoid arthritis (RA) and Felty syndrome (FS). She presented with acute renal kidney failure, skin rash and hemoptysis. A clinical suspicion of small vessel vasculitis (SVV) was thought, serology was also positive for various markers of SVV. However, these serology markers could be false-positive in a patient of rheumatoid arthritis. A renal biopsy was performed that led to the final diagnosis of cryoglobulinemic vasculitis. Patient was managed according to the standard guidelines for therapy (plasmafiltration and immunosuppression). It is challenging to manage a patient of RA, in the presence of Felty syndrome-related granulocytopenia and thrombocytopenia. Patient initially showed signs of improvement, but finally succumbed to complications of therapy. The case provides insight into the diagnosis and management of such cases.

Publication Type

Case Reports. Journal Article.

Year of Publication

2012

<470>

Unique Identifier

22984268

Title

ZAP-70+ B cell subset influences response to B cell depletion therapy and early repopulation in rheumatoid arthritis.

Source

Journal of Rheumatology. 39(12):2276-85, 2012 Dec.

VI 1

Status

MEDLINE

Authors

Gremese E; Tolusso B; Fedele AL; Canestri S; Alivernini S; Ferraccioli G.

Authors Full Name

Gremese, Elisa; Tolusso, Barbara; Fedele, Anna Laura; Canestri, Silvia; Alivernini, Stefano; Ferraccioli, Gianfranco.

Institution

Gremese, Elisa. Division of Rheumatology, Institute of Rheumatology and Affine Sciences (IRSA), School of Medicine, Catholic University of the Sacred Heart, Rome, Italy.

Abstract

OBJECTIVE: To define the role of ZAP-70+ B cells (CD19+/ZAP-70+) as a biomarker of response to B cell depletion therapy (BCDT), their relationship with clinical outcome, and their behavior during repopulation of peripheral blood in patients with rheumatoid arthritis (RA).

METHODS: Thirty-one patients with RA underwent BCDT and were followed for 12 months. Disease activity was assessed with the European League Against Rheumatism (EULAR) criteria. Cytofluorimetric analysis of peripheral blood B cell subsets at baseline and at 6- and 12-month intervals after BCDT was performed using surface markers (CD45, CD3, CD56, CD19, IgD, CD38, CD27) and intracellular ZAP-70.

RESULTS: A moderate/good EULAR response was achieved in 66.6% of the RA cohort. The baseline percentage of CD19+/ZAP-70+ cells was lower in good responder patients (1.8% +/- 1.7%) compared to poor responders (5.6% +/- 4.9%; p = 0.02). A decrease of plasmablasts (IgD-CD27+CD38+) and pre-switch memory (IgD+CD27+) B cells occurred after BCDT. Recovery of B

cells in peripheral blood after the first course of BCDT was characterized by the reappearance of B cell subtypes that showed a naive, activated phenotype, coupled with a decrease in memory cells. B cells carrying intracytoplasmic ZAP-70 increased significantly from the baseline value of 4.4% +/- 4.5% to 12.4% +/- 9.2% (p = 0.001) at the 6-month and to 9.4% +/- 6.4% (p = 0.002) at the 12-month followup.

CONCLUSION: Baseline percentage of CD19+/ZAP-70+ cells is associated with the clinical outcome after BCDT in patients with RA. Depletion of plasmablasts and pre-switch memory B cells and increase of CD19+/ZAP-70+ cells are features of the recovery of the B cell pool after BCDT.

Publication Type

Journal Article. Research Support, Non-U.S. Gov't.

Year of Publication

2012

<471>

Unique Identifier

22974785

Title

Influence on effectiveness of early treatment with anti-TNF therapy in rheumatoid arthritis.

Source

Journal of Pharmacy & Pharmaceutical Sciences. 15(3):355-60, 2012.

VI 1

Status

MEDLINE

Authors

Escudero-Vilaplana V; Ramirez-Herraiz E; Trovato-Lopez N; Alanon-Plaza E; Bellini MJ; Herranz-Alonso A; Bellon-Cano JM; Morell-Baladron A; Sanjurjo-Saez M.

Authors Full Name

Escudero-Vilaplana, Vicente; Ramirez-Herraiz, Esther; Trovato-Lopez, Nicolas; Alanon-Plaza, Estefania; Bellini, Maria Jose; Herranz-Alonso, Ana; Bellon-Cano, Jose Maria; Morell-Baladron, Alberto; Sanjurjo-Saez, Maria.

Institution

Escudero-Vilaplana, Vicente. Pharmacy Department, Gregorio Maranon University General Hospital, Madrid, Spain. vescudero.hgugm@salud.madrid.org
Abstract

PURPOSE: To evaluate the association between starting early treatment with anti-TNF and effectiveness as well as the possibility of applying therapeutic spacing in daily practice in patients with rheumatoid arthritis (RA).

METHODS: Observational, retrospective study conducted in two universitary hospitals in Spain. RA patients who received the first anti-TNF (adalimumab: ADA, etanercept: ETN or infliximab: IFX) during the study period (October 2006-2010) were included. Demographic data, time since diagnosis, disease activity (DAS28-ESR) and anti-TNF dosage were analyzed. Therapeutic objective was defined as DAS28 DAS28 < 2.6. Also the response related to criteria of the European League Against Rheumatism (EULAR) was evaluated. Therapeutic spacing was defined as the use of a lower dose or a higher interval according to label doses. The main endpoint was to assess the association between the effectiveness and the moment when the anti-TNF therapy begins. The secondary target was to evaluate the association between RA activity at the beginning of treatment with anti-TNF and dose used. Results . 82 patients were included. The prescription profile was: ADA (48.8%), ETN (31.7%) and IFX (19.5%). 71.4% of patients treated with anti-TNF during the first year since diagnosis, 57.1% of those who started after 1-5 years and 30.6% of patients who started after 5 years were in remission when the study ended. De-escalation strategy was performed in 25.6% of patients: ETN (38.5%), ADA (20.0%) and IFX (18.8%). The patients treated with a higher dose according to label doses were: IFX (81%), ADA, (12.5%) and ETN (7.7%).

CONCLUSIONS: Results suggest that early treatment with anti-TNF can achieve a higher percentage of remissions. Therapeutic spacing is established as a strategy that improves the efficiency in those patients in remission, being the ETN the anti-TNF most susceptible for spacing, although a relation between the early beginning with anti-TNF and the used dose was not found.
Publication Type

Journal Article. Multicenter Study. Research Support, Non-U.S. Gov't.

Year of Publication

2012

<472>

Unique Identifier

22969816

Title

Hematopoietic stem cell transplantation for systemic lupus erythematosus. [Review]

Source

Clinical & Developmental Immunology. 2012:380391, 2012.

VI 1

Status

MEDLINE

Authors

Marmont du Haut Champ AM.

Authors Full Name

Marmont du Haut Champ, Alberto M.

Institution

Marmont du Haut Champ, Alberto M. Division of Hematology and Stem Cell Transplantation, IRCCS Azienda Ospedaliera Universitaria San Martino-IST, Genoa, Italy. alberto.marmont@hsanmartino.it

Abstract

Two streams of research are at the origin of the utilization of hematopoietic stem cell transplantation (HSCT) for severe autoimmune diseases (SADs). The allogeneic approach came from experimental studies on lupus mice, besides clinical results in coincidental diseases. The autologous procedure was encouraged by researches on experimental neurological and rheumatic disorders. At present the number of allogeneic HSCT performed for human SADs can be estimated to not over 100 patients, and the results are not greatly encouraging, considering the significant transplant-related mortality (TRM) and the occasional development of a new autoimmune disorder and/or relapses notwithstanding full donor chimerism. Autologous HSCT for refractory SLE has become a major target. Severe cases have been salvaged, TRM is low and diminishing, and prolonged clinical remissions are obtainable. Two types of immune resetting have been established, "re-education" and regulatory T cell (Tregs) normalization. Allogeneic HSCT for SLE seems best indicated for patients with disease complicated by an oncohematologic malignancy. Autologous HSCT is a powerful salvage therapy for otherwise intractable SLE. The duration of remission in uncertain, but a favorable response to previously inactive treatments is a generally constant feature. The comparison with new biological agents, or the combination of both, are to be ascertained.

Publication Type

Journal Article. Review.
Year of Publication
2012

<473>
Unique Identifier
22925480
Title
Rheumatoid arthritis and the incidence of influenza and influenza-related complications: a retrospective cohort study.
Source
BMC Musculoskeletal Disorders. 13:158, 2012 Aug 27.
VI 1
Status
MEDLINE
Authors
Blumentals WA; Arreglado A; Napalkov P; Toovey S.
Authors Full Name
Blumentals, William A; Arreglado, Anna; Napalkov, Pavel; Toovey, Stephen.
Institution
Blumentals, William A. Hoffmann-La Roche, Inc, Nutley, NJ, USA.
Abstract
BACKGROUND: Patients with rheumatoid arthritis (RA) are known to be at increased risk of infection, particularly if they are taking drugs with immunomodulatory effects. There is a need for more information on the risk of influenza in patients with RA.

METHODS: A retrospective cohort study was carried out using data gathered from a large US commercial health insurance database (Thomson Reuters Medstat MarketScan) from 1 January 2000 to 31 December 2007. Patients were >=18 years of age, with at least two RA claims diagnoses. The database was scanned for incidence of seasonal influenza and its complications on or up to 30 days after an influenza diagnosis in RA patients and matched controls. Other factors accounted for included medical conditions, use of disease-modifying anti-rheumatic drugs (DMARDs), use of biological agents, influenza vaccination and high- or low-dose corticosteroids.

Incidence rate ratios (IRRs) were calculated for influenza and its complications in patients with RA.

RESULTS: 46,030 patients with RA and a matching number of controls had a median age of 57 years. The incidence of influenza was higher in RA patients than in controls (409.33 vs 306.12 cases per 100,000 patient-years), and there was a 2.75-fold increase in incidence of complications in RA. Presence or absence of DMARDs or biologics had no significant effect. The adjusted IRR of influenza was statistically significant in patients aged 60-69 years, and especially among men. A significantly increased rate of influenza complications was observed in women and in both genders combined (but not in men only) when all age groups were combined. In general, the risk of influenza complications was similar in RA patients not receiving DMARDs or biologics to that in all RA patients. Pneumonia rates were significantly higher in women with RA. Rates of stroke/myocardial infarction (MI) were higher in men, although statistical significance was borderline.

CONCLUSIONS: RA is associated with increased incidence of seasonal influenza and its complications. Gender- and age-specific subgroup data indicate that women generally have a greater rate of complications than men, but that men primarily have an increased rate of stroke and MI complications. Concomitant DMARD or biological use appears not to significantly affect the rate of influenza or its complications.

Publication Type

Journal Article. Research Support, Non-U.S. Gov't.

Year of Publication

2012

<474>

Unique Identifier

22875602

Title

Relapse of systemic juvenile idiopathic arthritis after influenza vaccination in a patient receiving tocilizumab.

Source

Clinical & Vaccine Immunology: CVI. 19(10):1700-2, 2012 Oct.

VI 1
Status
  MEDLINE
Authors
  Shimizu M; Ueno K; Yachie A.
Authors Full Name
  Shimizu, Masaki; Ueno, Kazuyuki; Yachie, Akihiro.
Institution
  Shimizu, Masaki. Department of Pediatrics, School of Medicine, Institute of Medical, Pharmaceutical, and Health Sciences, Kanazawa University, Japan. shimizum@staff.kanazawa-u.ac.jp
Abstract
  We report the case of a patient with systemic juvenile idiopathic arthritis (s-JIA) receiving tocilizumab (TCZ) who experienced relapses of s-JIA after receiving influenza vaccination. Systemic symptoms of s-JIA might be masked during TCZ therapy. Careful observation with the monitoring of serum interleukin (IL)-18 and IL-6 levels may be useful.
Publication Type
  Case Reports. Journal Article.
Year of Publication
  2012

<475>
Unique Identifier
  22871954
Title
  Updates on B-cell immunotherapies for systemic lupus erythematosus and Sjogren's syndrome. [Review]
Source
  Current Opinion in Rheumatology. 24(5):451-6, 2012 Sep.
VI 1
Status
  MEDLINE
Authors

Coca A; Sanz I.
Authors Full Name
Coca, Andreea; Sanz, Inaki.
Institution
Coca, Andreea. Division of Allergy, Immunology and Rheumatology, University of Rochester School of Medicine, Rochester, New York, USA. Andreea_Coca@URMC.Rochester.edu
Abstract
PURPOSE OF REVIEW: Last year was marked by important clinical and mechanistic studies that improved our understanding of B-cell immunotherapy for systemic lupus erythematosus (SLE) and Sjogren's syndrome. Here, we will highlight the most relevant studies published in the last 18 months.

RECENT FINDINGS: The highlight of the year was the approval of belimumab on the basis of two major trials. On the flip side, the disappointing results of rituximab in lupus nephritis provided a clinical and mechanistic counterpoint in SLE. Still, major limitations in the LUpus Nephritis Assessment with Rituximab (LUNAR) trial, positive subset analysis and new open studies and registries continue to provide hope for and major insights into the use of B-cell depletion. In Sjogren's syndrome, the role of B-cell depletion has been further investigated, both for glandular and extraglandular manifestations of the disease with mixed results in a disease in which outcomes are notoriously hard to measure.

SUMMARY: The approval of anti-B cell activating factor therapy and an increasing body of open studies with rituximab as well as subset studies and secondary analysis of the Efficacy and Safety of Rituximab in Moderately-to-Severely Active Systemic Lupus Erythematosus (EXPLORER) and LUNAR trials provide hope for B-cell immunotherapy and significant insight into its mechanisms of action and utilization in a selected subset of patients. Ongoing clinical trials of other B-cell targeting agents are eagerly anticipated.
Publication Type
Journal Article. Review.
Year of Publication
2012

<476>

Unique Identifier
22864995
Title
Vaccination coverage in children with juvenile idiopathic arthritis followed at a paediatric tertiary care centre.
Source
Rheumatology. 51(11):2046-50, 2012 Nov.
VI 1
Status
MEDLINE
Authors
Morin MP; Quach C; Fortin E; Chedeville G.
Authors Full Name
Morin, Marie-Paule; Quach, Caroline; Fortin, Elise; Chedeville, Gaelle.
Institution
Morin, Marie-Paule. Division of Rheumatology, The Montreal Children's Hospital, 2300 Tupper Street, Montreal, Quebec, H3H 1P3, Canada.
Abstract
OBJECTIVE: To evaluate the vaccination coverage rate of patients with JIA followed at a paediatric tertiary care centre and to determine the coverage rate for individual vaccines required as per the Quebec Immunization Protocol.

METHODS: Consecutive JIA patients coming for their scheduled visit were included if they were between 2 and 18 years of age and if they had an available written immunization record. Descriptive statistics were used to evaluate the proportion of children with complete vaccination status according to the Quebec Immunization Protocol at 2.5, 10.5 years and at their last clinic visit.

RESULTS: A total of 200 patients were included. Complete vaccination according to schedule was identified in only 52% of patients at 2.5 years, 68% at 10.5 years and 61% at their last clinic visit. The vaccination coverage rate for individual vaccines was good overall with the exception of low measles, mumps and rubella vaccine coverage at 2.5 years (58%).

CONCLUSION: Despite overall good vaccination coverage rate for individual vaccines, only 61% of our cohort had a complete vaccination status at their last clinic visit. Measures to optimize vaccination coverage, such as catch-up vaccination, should be implemented when possible.
Publication Type

Journal Article.
Year of Publication
2012

<477>
Unique Identifier
22859946
Title
Biomarkers of good EULAR response to the B cell depletion therapy in all seropositive rheumatoid arthritis patients: clues for the pathogenesis.
Source
PLoS ONE [Electronic Resource]. 7(7):e40362, 2012.
VI 1
Status
MEDLINE
Authors
Ferraccioli G; Tolusso B; Bobbio-Pallavicini F; Gremese E; Ravagnani V; Benucci M; Podesta E; Atzeni F; Mannocci A; Biasi D; Manfredi M; Sarzi-Puttini P; Lagana B; Montecucco C.
Authors Full Name
Ferraccioli, Gianfranco; Tolusso, Barbara; Bobbio-Pallavicini, Francesca; Gremese, Elisa; Ravagnani, Viviana; Benucci, Maurizio; Podesta, Edoardo; Atzeni, Fabiola; Mannocci, Alice; Biasi, Domenico; Manfredi, Mariangela; Sarzi-Puttini, Piercarlo; Lagana, Bruno; Montecucco, Carlomaurizio.
Institution
Ferraccioli, Gianfranco. Division of Rheumatology, Catholic University of the Sacred Heart, Rome, Italy. gf.ferraccioli@rm.unicatt.it
Abstract
OBJECTIVE: To find out whether a high number of auto-antibodies can increase the probability of a "good-EULAR response" and to identify the possible biomarkers of response in seropositive rheumatoid arthritis (RA) patients undergoing the B cell depletion therapy (BCDT).

PATIENTS AND METHODS: One hundred and thirty-eight patients with long standing RA (LSRA), 75% non or poorly responsive to one or more TNFalpha blockers, all seropositive for at

least one autoantibody (AAB) (RF-IgM, RF-IgA, RF-IgG, anti-MCV, ACPA-IgG, ACPA-IgA, ACPA-IgM) received one full course of BCDT. The major outcomes (moderate or good-EULAR response) were assessed after 6 months of therapy. The IL6 and BAFF levels were also determined.

RESULTS: At a 6-month follow-up, 33 (23.9%) of the RA patients achieved a good EULAR response. Having up to 5-AABs positivity increased the chances for treatment response. After a logistic regression analysis, however, only 4 baseline factors arose as associated with a good-EULAR response: no steroid therapy (OR = 6.25), a lymphocyte count <1875/uL (OR = 10.74), a RF-IgG level >52.1 IU/ml (OR = 8.37) and BAFF levels <1011 pg/ml (OR = 7.38). When all the AABs, except for RF-IgM and ACPA-IgG, were left in the analysis, the two final predictors were no-steroid therapy and low lymphocyte count.

DISCUSSION: The number of AABs increased the chances of being a "good-EULAR" responder. The only predictors, however, at the baseline of a good response in this seropositive cohort of RA patients were 2 simple variables--no steroids and lymphocyte count--and two laboratory assays--IgG-RF and BAFF.

Publication Type

Journal Article. Research Support, Non-U.S. Gov't.

Year of Publication

2012

<478>

Unique Identifier

22848374

Title

Helminth antigens enable CpG-activated dendritic cells to inhibit the symptoms of collagen-induced arthritis through Foxp3+ regulatory T cells.

Source

PLoS ONE [Electronic Resource]. 7(7):e40356, 2012.

VI 1

Status

MEDLINE

Authors
Carranza F; Falcon CR; Nunez N; Knubel C; Correa SG; Bianco I; Maccioni M; Fretes R; Triquell MF; Motran CC; Cervi L.
Authors Full Name
Carranza, Franco; Falcon, Cristian Roberto; Nunez, Nicolas; Knubel, Carolina; Correa, Silvia Graciela; Bianco, Ismael; Maccioni, Mariana; Fretes, Ricardo; Triquell, Maria Fernanda; Motran, Claudia Cristina; Cervi, Laura.
Institution
Carranza, Franco. Departamento de Bioquimica Clinica, CIBICI-CONICET, Facultad de Ciencias Quimicas, Universidad Nacional de Cordoba, Cordoba, Argentina.
Abstract
Dendritic cells (DC) have the potential to control the outcome of autoimmunity by modulating the immune response. In this study, we tested the ability of Fasciola hepatica total extract (TE) to induce tolerogenic properties in CpG-ODN (CpG) maturated DC, to then evaluate the therapeutic potential of these cells to diminish the inflammatory response in collagen induced arthritis (CIA). DBA/1J mice were injected with TE plus CpG treated DC (T/C-DC) pulsed with bovine collagen II (CII) between two immunizations with CII and clinical scores CIA were determined. The levels of CII-specific IgG2 and IgG1 in sera, the histological analyses in the joints, the cytokine profile in the draining lymph node (DLN) cells and in the joints, and the number, and functionality of CD4+CD25+Foxp3+ T cells (Treg) were evaluated. Vaccination of mice with CII pulsed T/C-DC diminished the severity and incidence of CIA symptoms and the production of the inflammatory cytokine, while induced the production of anti-inflammatory cytokines. The therapeutic effect was mediated by Treg cells, since the adoptive transfer of CD4+CD25+ T cells, inhibited the inflammatory symptoms in CIA. The in vitro blockage of TGF-beta in cultures of DLN cells plus CII pulsed T/C-DC inhibited the expansion of Treg cells. Vaccination with CII pulsed T/C-DC seems to be a very efficient approach to diminish exacerbated immune response in CIA, by inducing the development of Treg cells, and it is therefore an interesting candidate for a cell-based therapy for rheumatoid arthritis (RA).
Publication Type
Journal Article. Research Support, Non-U.S. Gov't.
Year of Publication
2012

<479>
Unique Identifier
22841377
Title
B-cell populations and sub-populations in Sjogren's syndrome. [Review]
Source
Presse Medicale. 41(9 Pt 2):e475-83, 2012 Sep.
VI 1
Status
MEDLINE
Authors
Hamza N; Bos NA; Kallenberg CG.
Authors Full Name
Hamza, Nishath; Bos, Nicolaas A; Kallenberg, Cees G M.
Institution
Hamza, Nishath. University of Groningen, University Medical Center Groningen, Department of Rheumatology and Clinical Immunology, 9700 RB Groningen, The Netherlands.
Abstract
Sjogren's Syndrome (SS) is a chronic inflammatory disorder affecting exocrine glands, in particular the lacrimal and salivary glands. The disease can be primary (pSS) or secondary to other systemic autoimmune diseases such as rheumatoid arthritis, systemic lupus erythematosus and others. The systemic autoimmune character of pSS is also apparent from the occurrence of (non-organ specific) autoantibodies in this disease. Histopathologically, glandular involvement is characterized by focal accumulation of lymphocytes, particularly around epithelial ducts, with, sometimes, germinal center-like structures. The infiltrates largely consist of T-cells, with a preponderance of CD4-positive T-cells. As a result, the pathology in SS was primarily attributed to T cells. However, a break with the fixation on the role of T cells in pSS came when therapeutic B-cell depletion strategies proved remarkably efficacious in this disease, thereby indicating a major role for B-cells in the immunopathogenesis of pSS. In this regard, a closer look at the composition of B-cells and B-cell sub-populations, both in the peripheral blood and in target tissues, is worthwhile. In this review, we discuss current data on B-cells in pSS. B-cell depletion offers a unique possibility to study the recurrence of (pathogenic) B-cells and their characteristics in pSS patients treated with rituximab. Data on B-cell sub-populations in the peripheral blood and B-cell repertoire in the target tissues following rituximab treatment are discussed as well. We also address their state of activation, repertoire, and relation to B-cell activating factor (BAFF).
Copyright © 2012 Elsevier Masson SAS. All rights reserved.
Publication Type

Journal Article. Review.
Year of Publication
2012

<480>
Unique Identifier
22840370
Title
Rhodococcus erythropolis encephalitis in patient receiving rituximab.
Source
Emerging Infectious Diseases. 18(8):1377-9, 2012 Aug.
VI 1
Status
MEDLINE
Authors
Bagdure SR; Fisher MA; Ryan ME; Khasawneh FA.
Authors Full Name
Bagdure, Satish R; Fisher, Mark A; Ryan, Michael E; Khasawneh, Faisal A.
Publication Type
Case Reports. Letter.
Year of Publication
2012

<481>
Unique Identifier
22832287
Title
Knowledge, attitudes, and clinical practice of rheumatologists in vaccination of the at-risk rheumatology patient population.

Source
JCR: Journal of Clinical Rheumatology. 18(5):237-41, 2012 Aug.
VI 1
Status
MEDLINE
Authors
McCarthy EM; Azeez MA; Fitzpatrick FM; Donnelly S.
Authors Full Name
McCarthy, Eoghan M; Azeez, Maha Abdul; Fitzpatrick, Fidelma M; Donnelly, Suzanne.
Institution
McCarthy, Eoghan M. Department of Rheumatology, Mater Misericordiae University Hospital, Dublin, Ireland. eoghanmccarthy@yahoo.com
Abstract
BACKGROUND: Patients with inflammatory arthritis are at increased risk of infection. Much of the burden of infection in this population is vaccine preventable. A number of international rheumatology organizations have published expert recommendations for vaccination in adult patients. Despite this, reported vaccination rates remain low among patients with inflammatory arthritis.

OBJECTIVES: We sought to establish the knowledge, attitudes, and clinical practice of rheumatologists with respect to vaccination.

METHODS: Rheumatologists practicing in Ireland in 2009 were surveyed by postal questionnaire. Data collected was entered into Microsoft Excel and statistical analysis was carried out using SPSS18 software.

RESULTS: Eighty (100%) practicing rheumatologists were surveyed. Response rate was 55% (44/80). Of those surveyed, 57% (25/44) had no written departmental vaccination guidelines. Although 90% of those surveyed agreed that the responsibility for ensuring vaccine compliance rests with health professionals, only 5% considered that the rheumatology clinic was the best setting in which to accomplish this. Half (50%, n = 22) of practicing rheumatologists do not inquire about vaccination history in the clinic, with a minority (9%, n = 4) recording vaccination history in their clinical notes. A significant percentage of rheumatologists do not perform screening about prior vaccination before initiation of either anti-tumor necrosis factor (34%) or disease-modifying antirheumatic disease (42%) therapy. Moreover, 57% (n = 25) considered the responsibility for vaccination the domain of the patients' general practitioners with the favored strategy to improve vaccine compliance being led by the primary care physicians (48%, n = 21).

CONCLUSIONS: The practice of Irish rheumatologists with regard to vaccination in this survey was suboptimal. Most neither recommend nor record vaccination history in their clinical notes, with the majority feeling that the rheumatology clinic is not the appropriate setting in which to target strategies to improve vaccine compliance. Although a more proactive role needs to be taken by rheumatologists as the principal prescribers of immunosuppressive therapy on this issue, our survey respondents suggest that strategies to improve vaccine uptake should be developed outside the rheumatology clinic and, in particular, involve primary care. The circulation of currently available international guidelines on vaccination specific for rheumatology patients to primary care physicians should be used to inform practices to ensure improved vaccine compliance.

Publication Type

Journal Article.

Year of Publication

2012

<482>

Unique Identifier

22813350

Title

Risk factors for reported influenza and influenza-like symptoms in patients with rheumatoid arthritis.

Source

Scandinavian Journal of Rheumatology. 41(5):359-65, 2012 Oct.

VI 1

Status

MEDLINE

Authors

Dirven L; Huizinga TW; Allaart CF.

Authors Full Name

Dirven, L; Huizinga, T W J; Allaart, C F.

Institution

Dirven, L. Leiden University Medical Centre, Department of Rheumatology, Leiden, The Netherlands. l.dirven@lumc.nl
Abstract
OBJECTIVES: To determine the prevalence and predictors of influenza and influenza-like symptoms in patients with rheumatoid arthritis (RA).

METHOD: Questionnaires were sent to patients registered as having RA and they were asked to fill in per month any period and details of influenza-like symptoms and vaccination. An experienced rheumatologist assessed the level of disease activity and use of anti-rheumatic medication. The prevalence of reported influenza (fever > 38degreeC, headache, muscle soreness, and coughing and/or dyspnoea) and influenza-like symptoms was determined and risk factors were identified by logistic regression analysis.

RESULTS: Of the 1692 patients approached, 783 (46%) patients were eligible for follow-up. Fifty per cent of the patients reported influenza-like symptoms, 5.9% had symptoms suggesting influenza, and 74% reported vaccination. The prevalence of influenza and influenza-like symptoms per month ranged from 0.0% to 2.3% and from 10.4% to 19.7%, respectively. Anti-tumour necrosis factors (anti-TNFs) [odds ratio (OR) 2.4, 95% confidence interval (CI) 1.2-4.8] and body mass index (BMI) (OR 1.06, 95% CI 1.0-1.1) were independently associated with symptoms of influenza. A trend was found for patients not in remission, patients using leflunomide, and patients with previous lung conditions. Independent risk factors of influenza-like symptoms were age (OR 0.98, 95% CI 0.97-0.99), female gender (OR 1.8, 95% CI 1.3-2.5), influenza vaccination (OR 1.6, 95% CI 1.1-2.4), and previous lung condition (OR 1.7, 95% CI 1.2-2.4).

CONCLUSIONS: In 2009-2010, the prevalence of reported influenza in patients with RA was 5.9%. Patients using anti-TNFs and with higher BMI seemed to be more at risk for influenza symptoms. Milder upper respiratory tract infections were reported more often by females, younger patients, and those vaccinated against influenza or with previous lung conditions.
Publication Type
Journal Article.
Year of Publication
2012

<483>

Unique Identifier

22771605

Title

Challenges in the diagnosis & treatment of miliary tuberculosis. [Review]

Source

Indian Journal of Medical Research. 135(5):703-30, 2012 May.

VI 1

Status

MEDLINE

Authors

Sharma SK; Mohan A; Sharma A.

Authors Full Name

Sharma, Surendra K; Mohan, Alladi; Sharma, Abhishek.

Institution

Sharma, Surendra K. Department of Medicine, All India Institute of Medical Sciences, New Delhi, India. sksharma.aiims@gmail.com

Abstract

Miliary tuberculosis (TB) is a potentially lethal disease if not diagnosed and treated early. Diagnosing miliary TB can be a challenge that can perplex even the most experienced clinicians. Clinical manifestations are nonspecific, typical chest radiograph findings may not be evident till late in the disease, high resolution computed tomography (HRCT) shows randomly distributed miliary nodules and is relatively more sensitive. Ultrasonography, CT and magnetic resonance imaging (MRI) are useful in discerning the extent of organ involvement by lesions of miliary TB in extra-pulmonary locations. Fundus examination for choroid tubercles, histopathological examination of tissue biopsy specimens, conventional and rapid culture methods for isolation of Mycobacterium tuberculosis, drug-susceptibility testing, along with use of molecular biology tools in sputum, body fluids, other body tissues are useful in confirming the diagnosis. Although several prognostic markers have been described which predict mortality, yet untreated miliary TB has a fatal outcome within one year. A high index of clinical suspicion and early diagnosis and timely institution of anti-tuberculosis treatment can be life-saving. Response to first-line anti-tuberculosis drugs is good but drug-induced hepatotoxicity and drug-drug interactions in human immunodeficiency virus/acquired immunodeficiency syndrome (HIV/AIDS) patients pose significant problems during treatment. However, sparse data are available from randomized controlled trials to define the optimum regimen and duration of treatment in patients with drug-sensitive as well as drug-resistant miliary TB, including those with HIV/AIDS.

Publication Type
  Journal Article. Review.
Year of Publication
  2012

<484>
Unique Identifier
  22770665
Title
  Critical involvement of macrophage infiltration in the development of Sjogren's syndrome-associated dry eye.
Source
  American Journal of Pathology. 181(3):753-60, 2012 Sep.
VI 1
Status
  MEDLINE
Authors
  Zhou D; Chen YT; Chen F; Gallup M; Vijmasi T; Bahrami AF; Noble LB; van Rooijen N; McNamara NA.
Authors Full Name
  Zhou, Delu; Chen, Ying-Ting; Chen, Feeling; Gallup, Marianne; Vijmasi, Trinka; Bahrami, Ahmad F; Noble, Lisa B; van Rooijen, Nico; McNamara, Nancy A.
Institution
  Zhou, Delu. Francis I. Proctor Foundation, University of California, San Francisco, USA.
Abstract
  Lymphocytic infiltration of the lacrimal gland and ocular surface in autoimmune diseases such as Sjogren's syndrome (SS) causes an aqueous-deficient dry eye that is associated with significant morbidity. Previous studies from our laboratory and others have established autoimmune regulator (Aire)-deficient mice as a useful model to examine exocrinopathy and ocular surface disease associated with SS. Consistent with human SS, autoreactive CD4(+) T cells play an indispensible role in the development of exocrine and ocular surface disease in Aire knockout mice. We report that in addition to CD4(+) T cells, a large number of macrophages infiltrate the corneal stroma, limbus, and lacrimal glands of diseased mice. Adoptive transfer of autoreactive

CD4(+) T cells from Aire knockout mice led to local infiltration of macrophages and ocular surface damage in immunodeficient recipients. Depletion of local macrophages, through subconjunctival injection of clodronate liposome, attenuated lissamine green staining and improved ocular phenotype. Alternatively, systemic depletion of macrophages had no effect on ocular phenotype but led to significant improvements in lacrimal gland exocrinopathy and tear secretion. Our results suggested that autoreactive CD4(+) T cells provoked macrophage infiltration to the eye and lacrimal gland, where they played a functional role in directing the development of autoimmune dry eye. Copyright © 2012 American Society for Investigative Pathology. Published by Elsevier Inc. All rights reserved.

Publication Type

Journal Article. Research Support, N.I.H., Extramural.

Year of Publication

2012

<485>

Unique Identifier

22768242

Title

Ganglioside GM3 has an essential role in the pathogenesis and progression of rheumatoid arthritis.

Source

PLoS ONE [Electronic Resource]. 7(6):e40136, 2012.

VI 1

Status

MEDLINE

Authors

Tsukuda Y; Iwasaki N; Seito N; Kanayama M; Fujitani N; Shinohara Y; Kasahara Y; Onodera T; Suzuki K; Asano T; Minami A; Yamashita T.

Authors Full Name

Tsukuda, Yukinori; Iwasaki, Norimasa; Seito, Naoki; Kanayama, Masashi; Fujitani, Naoki; Shinohara, Yasuro; Kasahara, Yasuhiko; Onodera, Tomohiro; Suzuki, Koji; Asano, Tsuyoshi; Minami, Akio; Yamashita, Tadashi.

Institution

Tsukuda, Yukinori. Department of Orthopaedic Surgery, Hokkaido University Graduate School of Medicine, Sapporo, Japan.
Abstract
Rheumatoid arthritis (RA), a chronic systemic inflammatory disorder that principally attacks synovial joints, afflicts over 2 million people in the United States. Interleukin (IL)-17 is considered to be a master cytokine in chronic, destructive arthritis. Levels of the ganglioside GM3, one of the most primitive glycosphingolipids containing a sialic acid in the structure, are remarkably decreased in the synovium of patients with RA. Based on the increased cytokine secretions observed in in vitro experiments, GM3 might have an immunologic role. Here, to clarify the association between RA and GM3, we established a collagen-induced arthritis mouse model using the null mutation of the ganglioside GM3 synthase gene. GM3 deficiency exacerbated inflammatory arthritis in the mouse model of RA. In addition, disrupting GM3 induced T cell activation in vivo and promoted overproduction of the cytokines involved in RA. In contrast, the amount of the GM3 synthase gene transcript in the synovium was higher in patients with RA than in those with osteoarthritis. These findings indicate a crucial role for GM3 in the pathogenesis and progression of RA. Control of glycosphingolipids such as GM3 might therefore provide a novel therapeutic strategy for RA.
Publication Type
Journal Article. Research Support, Non-U.S. Gov't.
Year of Publication
2012

<486>
Unique Identifier
22749831
Title
B cells in Sjogren's syndrome: from pathophysiology to diagnosis and treatment. [Review]
Source
Journal of Autoimmunity. 39(3):161-7, 2012 Sep.
VI 1
Status
MEDLINE
Authors

Cornec D; Devauchelle-Pensec V; Tobon GJ; Pers JO; Jousse-Joulin S; Saraux A.
Authors Full Name
Cornec, Divi; Devauchelle-Pensec, Valerie; Tobon, Gabriel J; Pers, Jacques-Olivier; Jousse-Joulin, Sandrine; Saraux, Alain.
Institution
Cornec, Divi. Rheumatology, Brest teaching hospital, Brest, France; EA 2216, Immunology and Pathology, Brest University, Brest, France.
Abstract
Primary Sjogren's syndrome (pSS) is a chronic autoimmune systemic disease, characterized by a lymphoplasmocytic infiltration and a progressive destruction of salivary and lachrymal glands, leading to ocular and mouth dryness. T cells were originally considered to play the initiating role in the autoimmune process, while B cells were restricted to autoantibody production. However, recent years have seen growing evidence that the roles of B cells in pSS pathophysiology are multiple, and that these cells may actually play a central role in the development of the disease. B cells are over-stimulated and produce excessive amounts of immunoglobulins and various autoantibodies. Peripheral blood and salivary-gland B-cell subset distribution is altered, leading to the constitution of ectopic germinal centers where auto-reactive clones may escape tolerance checkpoints. B cells control T-cell activation by different means: B effector cells guide Th1 or Th2 differentiation, whereas regulatory B cells inhibit T-cell proliferation. Several B-cell specific cytokines, such as BAFF or Flt-3L, are instrumental in the occurrence of B-cell dysfunction. Chronic and excessive stimulation of B cells may lead to the development of lymphoma in pSS patients. Autoantibodies and blood B-cell subset analysis are major contributors of a clinical diagnosis of pSS. These considerations led to the development of B-cell depletion therapies for the management of pSS. Rituximab, a monoclonal antibody to CD20, is the best studied biologics in pSS, but other treatments hold promise, targeting for example CD22 or BAFF. Thus, during the last 20 years, the understanding of the multifaceted roles of B cells in pSS has revolutionized the management of this complex disease. Copyright © 2012 Elsevier Ltd. All rights reserved.
Publication Type
Journal Article. Review.
Year of Publication
2012

<487>

Unique Identifier

22642385

Title

Rhenium-188: availability from the (188)W/(188)Re generator and status of current applications. [Review]

Source

Current Radiopharmaceuticals. 5(3):228-43, 2012 Jul.

VI 1

Status

MEDLINE

Authors

Pillai MR; Dash A; Knapp FF Jr.

Authors Full Name

Pillai, M R A; Dash, Ashutosh; Knapp, F F Jr.

Institution

Pillai, M R A. Radiopharmceuticals Division, Bhabha Atomic Research Centre, Mumbai 400 085, India. mrap@barc.gov.in

Abstract

Rhenium-188 is one of the most readily available generator derived and useful radionuclides for therapy emitting beta(-) particles (2.12 MeV, 71.1% and 1.965 MeV, 25.6%) and imageable gammas (155 keV, 15.1%). The (188)W/(188)Re generator is an ideal source for the long term (4-6 months) continuous availability of no carrier added (nca) (188)Re suitable for the preparation of radiopharmaceuticals for radionuclide therapy. The challenges associated with the double neutron capture route of production of the parent (188)W radionuclide have been a major impediment in the progress of application of (188)Re. Tungsten-188 of adequate specific activity can be prepared only in 2-3 of the high flux reactors operating in the World. Several useful technologies have been developed for the preparation of clinical grade (188)W/(188)Re generators. Since the specific activity of (188)W used in the generator is relatively low 185 GBq(< 5 Ci)/g], the eluted (188)ReO(4)(-) can have low radioactive concentration often insufficient for radiopharmaceutical preparation. However, several efficient post elution concentration techniques have been developed that yield clinically useful (188)ReO(4)(-) solutions. Rhenium-188 has been used for the preparation of therapeutic radiopharmaceuticals for the management of diseases such as bone metastasis, rheumatoid arthritis and primary cancers. Several early phase clinical studies using radiopharmaceuticals based on (188)Re-labeled phosphonates, antibodies, peptides, lipiodol and particulates have been reported. This article reviews the availability and use of (188)Re including a discussion of why broader use of (188)Re has not progressed as expected as a popular radionuclide for therapy.

Publication Type
  Journal Article. Review.
Year of Publication
  2012

<488>
Unique Identifier
  22619829
Title
  [Good preparation is necessary: travel with rheumatoid arthritis]. [German]
Source
  MMW Fortschritte der Medizin. 154(4):20, 22, 2012 Mar 08.
VI 1
Status
  MEDLINE
Authors
  Neumaier J.
Authors Full Name
  Neumaier, Judith.
Publication Type
  News.
Year of Publication
  2012

<489>
Unique Identifier
  22615459
Title

Persistence of immunoglobulin-producing cells in parotid salivary glands of patients with primary Sjogren's syndrome after B cell depletion therapy.

Source

Annals of the Rheumatic Diseases. 71(11):1881-7, 2012 Nov.

VI 1

Status

MEDLINE

Authors

Hamza N; Bootsma H; Yuvaraj S; Spijkervet FK; Haacke EA; Pollard RP; Visser A; Vissink A; Kallenberg CG; Kroese FG; Bos NA.

Authors Full Name

Hamza, Nishath; Bootsma, Hendrika; Yuvaraj, Saravanan; Spijkervet, Fred K L; Haacke, Erlin A; Pollard, Rodney P E; Visser, Annie; Vissink, Arjan; Kallenberg, Cees G M; Kroese, Frans G M; Bos, Nicolaas A.

Institution

Hamza, Nishath. Department of Rheumatology and Clinical Immunology, University Medical Center Groningen, University of Groningen, Groningen, Netherlands.

Abstract

OBJECTIVES: To assess the persistence of immunoglobulin-producing cell populations in the parotid salivary glands of patients with primary Sjogren's syndrome (pSS) after B cell depletion therapy with rituximab.

METHODS: Thirteen patients with pSS and four control patients were included in this study. Patients with pSS were treated with rituximab or placebo. Sequence analysis was carried out on IgA- and IgG-encoding transcripts extracted from parotid salivary gland biopsy specimens taken before treatment and at 12-16 and 36-52 weeks after treatment.

RESULTS: At baseline, many clonally related sequences were seen in patients with pSS. The number of clonal expansions was significantly higher in patients with pSS than in control patients. Clonal expansions were composed of IgA- and/or IgG-expressing cells. Rituximab did not significantly alter the degree of clonal expansions. Groups of clonally related cells had members which were shared between biopsy specimens taken before and after treatment. Mutation frequencies of immunoglobulin sequences from clonally related cells in patients with pSS were higher after treatment.

CONCLUSIONS: Rituximab treatment does not alter the characteristic features of increased clonal expansions seen in the parotid salivary glands of patients with pSS. The presence of

clonally related immunoglobulin-producing cells before and after rituximab treatment strongly suggests that immunoglobulin-producing cells persist in salivary glands of patients with pSS despite B cell depletion. The presence of mixed isotype expression within groups of clonally related cells indicates local class switching in salivary glands of patients with pSS. Persistent immunoglobulin-producing cells may underlie disease relapse after treatment.

Publication Type

Journal Article. Research Support, Non-U.S. Gov't.

Year of Publication

2012

<490>

Unique Identifier

22595194

Title

Modulatory effect of mycophenolate mofetil on carrageenan-induced inflammation in the mouse air pouch model.

Source

International Immunopharmacology. 13(4):476-82, 2012 Aug.

VI 1

Status

MEDLINE

Authors

Dalmarco EM; Astolfi G; de Liz R; de Cordova CM; Frode TS.

Authors Full Name

Dalmarco, Eduardo Monguilhott; Astolfi, Giliard; de Liz, Rafael; de Cordova, Caio Mauricio Mendes; Frode, Tania Silvia.

Institution

Dalmarco, Eduardo Monguilhott. Department of Clinical Analyses, Centre of Health Sciences, Federal University of Santa Catarina, Campus Universitario, Trindade, 88040-970, Florianopolis, SC, Brazil.

Abstract

UNLABELLED: The treatment of some inflammatory diseases, such as rheumatoid arthritis, remains an important target for studies because some patients are refractory to conventional

treatment. Mycophenolate mofetil (MMF), an immunosuppressive drug, has been shown to have a beneficial effect on the therapy of inflammatory and autoimmune diseases. In the present study, we aimed to analyse the anti-inflammatory effect of MMF administered by oral route in the mouse carrageenan-induced air pouch model.

RESULTS: MMF significantly inhibited the influx of leukocytes, exudate concentrations (P<0.01), activities of myeloperoxidase (MPO) and adenosine deaminase (ADA), levels of nitrite/nitrate (NO(x)) and inducible nitric oxide synthase (iNOS) mRNA expression, as well as the levels of mRNA expression and proteins of tumor necrosis factor-alpha (TNF-alpha), Interleukin-beta (IL-1beta) and vascular endothelial growth factor-alpha (VEGF-alpha) (P<0.05). These results provide evidence that MMF has an important anti-inflammatory effect in reducing the influx of leukocytes and exudate concentrations. These inhibitory effects are correlated with the inhibition of specific pro-inflammatory enzymes (MPO, ADA and iNOS), and the levels of mRNA expression and proteins of TNF-alpha, IL-1beta and VEGF-alpha. Copyright © 2012 Elsevier B.V. All rights reserved.

Publication Type

Journal Article. Research Support, Non-U.S. Gov't.

Year of Publication

2012

<491>

Unique Identifier

22593620

Title

CD23+ CD21(high) CD1d(high) B cells in inflamed lymph nodes are a locally differentiated population with increased antigen capture and activation potential.

Source

Journal of Immunology. 188(12):5944-53, 2012 Jun 15.

VI 1

Status

MEDLINE

Authors

Moshkani S; Kuzin II; Adewale F; Jansson J; Sanz I; Schwarz EM; Bottaro A.

Authors Full Name

Moshkani, Safiehkhatoon; Kuzin, Igor I; Adewale, Funmilola; Jansson, Johan; Sanz, Inaki; Schwarz, Edward M; Bottaro, Andrea.

Institution

Moshkani, Safiehkhatoon. Department of Medicine, University of Rochester School of Medicine and Dentistry, Rochester, NY 14642, USA.

Abstract

CD23(+)CD21(high)CD1d(high) B cells in inflamed nodes (Bin cells) accumulate in the lymph nodes (LNs) draining inflamed joints of the TNF-alpha-transgenic mouse model of rheumatoid arthritis and are primarily involved in the significant histological and functional LN alterations that accompany disease exacerbation in this strain. In this study, we investigate the origin and function of Bin cells. We show that adoptively transferred GFP(+) sorted mature follicular B (FoB) cells home preferentially to inflamed LNs of TNF-alpha-transgenic mice where they rapidly differentiate into Bin cells, with a close correlation with the endogenous Bin fraction. Bin cells are also induced in wild-type LNs after immunization with T-dependent Ags and display a germinal center phenotype at higher rates compared with FoB cells. Furthermore, we show that Bin cells can capture and process Ag-immune complexes in a CD21-dependent manner more efficiently than can FoB cells, and they express greater levels of MHC class II and costimulatory Ags CD80 and CD86. We propose that Bin cells are a previously unrecognized inflammation-induced B cell population with increased Ag capture and activation potential, which may facilitate normal immune responses but may contribute to autoimmunity when chronic inflammation causes their accumulation and persistence in affected LNs.

Publication Type

Journal Article. Research Support, N.I.H., Extramural.

Year of Publication

2012

<492>

Unique Identifier

22518822

Title

Arthritogenic T cells drive the recovery of autoantibody-producing B cell homeostasis and the adoptive transfer of arthritis in SCID mice.

Source

International Immunology. 24(8):507-17, 2012 Aug.

VI 1

Status

MEDLINE

Authors

Kis-Toth K; Radacs M; Olasz K; van Eden W; Mikecz K; Glant TT.

Authors Full Name

Kis-Toth, Katalin; Radacs, Marianna; Olasz, Katalin; van Eden, Willem; Mikecz, Katalin; Glant, Tibor T.

Institution

Kis-Toth, Katalin. Department of Molecular Medicine, Rush University Medical Center, Chicago, IL 60612, USA. kkistoth@bidmc.harvard.edu

Abstract

T cells orchestrate joint inflammation in rheumatoid arthritis (RA), but B cells/B cell-derived factors are also involved in disease pathogenesis. The goal of this study was to understand the role of antigen-specific T and B cells in the pathological events of arthritis, which is impossible to study in humans due to the small number of antigen-specific cells. To determine the significance of antigen-specific lymphocytes and antibodies in the development of an autoimmune mouse model of RA, we generated TCR transgenic (TCR-Tg) mice specific for the dominant arthritogenic epitope of cartilage proteoglycan (PG) and performed a series of combined transfers of T cells, B cells and autoantibodies into BALB/c.Scid mice. The adoptive transfer of highly purified T cells from naive TCR-Tg, arthritic TCR-Tg or arthritic wild-type mice induced arthritis in SCID recipients, but the onset and severity of the disease were dependent on the sequential events of the T cell-supported reconstitution of PG-specific B cells and autoantibodies. The presence of activated PG-specific T cells was critical for disease induction, establishing a unique milieu for the selective homeostasis of autoantibody-producing B cells. In this permissive environment, anti-PG autoantibodies bound to cartilage and induced activation of the complement cascade, leading to irreversible cartilage destruction in affected joints. These findings may lead to a better understanding of the complex molecular and cellular mechanisms of RA.

Publication Type

Journal Article. Research Support, N.I.H., Extramural. Research Support, Non-U.S. Gov't.

Year of Publication

2012

<493>

Unique Identifier

22509979

Title

B-cell activating factor levels in rheumatoid arthritis patients in response to treatment with biologics.

Source

Journal of Interferon & Cytokine Research. 32(7):338-40, 2012 Jul.

VI 1

Status

MEDLINE

Authors

Pyrpasopoulou A; Balaska E; Triantafyllou A; Anyfanti P; Aslanidis S; Douma S.

Authors Full Name

Pyrpasopoulou, Athina; Balaska, Ekaterini; Triantafyllou, Areti; Anyfanti, Panagiota; Aslanidis, Spyros; Douma, Stella.

Institution

Pyrpasopoulou, Athina. 2nd Propedeutic Department of Internal Medicine, Hippokration General Hospital, Thessaloniki, Greece. a.pyrpasopoulou@doctors.org.uk

Abstract

The B-cell-activating factor (BAFF), a member of the tumor necrosis factor (TNF) family, has recently attracted attention as a potent cytokine, involved in B-cell stimulation and survival of autoimmune cells. Despite its significance in the pathogenesis of autoimmune diseases, data is limited and inconclusive regarding its expression in different stages of rheumatoid arthritis (RA). The aim of this study was to assess BAFF in biologic-naive RA patients with early versus established disease and monitor its levels in response to anti-TNF treatment in seronegative- and seropositive patients. Based on our results, B-cell-activating factor (BAFF) did not appear to be overexpressed or differentially expressed early (<=2 years duration) in comparison to established rheumatoid arthritis (RA). Moreover, tumor necrosis factor (TNF) blockade did not appear to affect BAFF levels in either seropositive or seronegative RA patients, despite the association of anti-TNF treatment with the development of autoantibodies and the known anti-apoptotic effects of BAFF. As expected, BAFF became induced after B-cell depletion. Investigation of the effect of different biologics on the expression of BAFF and other cytokines will help elucidate the interconnecting immune pathways involved in the initiation and perpetuation of the inflammatory process.

Publication Type

Journal Article.

Year of Publication

2012

<494>

Unique Identifier

22483657

Title

Pneumococcal vaccine in patients with rheumatoid arthritis.

Source

Reumatologia Clinica. 8(4):229, 2012 Jul-Aug.

VI 1

Status

MEDLINE

Authors

Hernandez-Garcia I; Escribano Hernandez A.

Authors Full Name

Hernandez-Garcia, Ignacio; Escribano Hernandez, Alfonso.

Comments

Comment on (CON)

Publication Type

Letter. Comment.

Year of Publication

2012

<495>

Unique Identifier

22447884

Title

Immunity 12 years after alemtuzumab in RA: CD5+ B-cell depletion, thymus-dependent T-cell reconstitution and normal vaccine responses.

Source

Rheumatology. 51(8):1397-406, 2012 Aug.

VI 1

Status

MEDLINE

Authors

Anderson AE; Lorenzi AR; Pratt A; Wooldridge T; Diboll J; Hilkens CM; Isaacs JD.

Authors Full Name

Anderson, Amy E; Lorenzi, Alice R; Pratt, Arthur; Wooldridge, Tom; Diboll, Julie; Hilkens, Catharien M U; Isaacs, John D.

Institution

Anderson, Amy E. Institute of Cellular Medicine, Musculoskeletal Research Group, 4th Floor Catherine Cookson Building, Medical School, Newcastle University, Framlington Place, Newcastle upon Tyne NE2 4HH, UK.

Abstract

OBJECTIVES: Lymphocyte depleting therapies have been used to treat refractory autoimmune disease, including RA, but treatment may be associated with long-term lymphopenia. It is unclear whether delayed reconstitution preferentially affects lymphocyte subsets, how this modulates immune challenges and whether thymic function influences the outcome. These questions are now addressed in a detailed analysis of RA patients 12 years after alemtuzumab (anti-CD52) treatment.

METHODS: Blood was obtained from 20 RA patients 12 years after alemtuzumab treatment. Lymphocyte subsets were enumerated by flow cytometry. T-cell receptor excision circles (TRECs)/ml were determined to quantify thymic function, and serological responses to neoantigens and recall antigens were assessed.

RESULTS: RA patients remained lymphopenic 12 years after their first dose of alemtuzumab. CD5(+) B cells, which may be associated with autoantibody production, were significantly reduced in alemtuzumab-treated patients compared with age-matched disease controls. In addition, naive and memory CD4(+) T-cell subsets were present in altered proportions in patients who had received alemtuzumab, with increased effector memory CD4(+) T cells, and decreased naive and central memory CD4(+) T cells. TRECs were detectable in alemtuzumab-treated

patients and correlated with CD4(+) lymphocyte counts. Vaccine responses to neoantigens and recall antigens fell within the normal range for an ageing population.

CONCLUSIONS: Alemtuzumab therapy resulted in long-term alterations in lymphocyte subsets. The significance of these changes remains uncertain but patients respond normally to antigenic challenges. Thymic function remains an important determinant of T-cell reconstitution even several years after lymphocytotoxic therapy.

Publication Type

Journal Article. Research Support, Non-U.S. Gov't.

Year of Publication

2012

<496>

Unique Identifier

22409963

Title

Induction of long-term B-cell depletion in refractory rheumatoid arthritis patients preferentially affects autoreactive more than protective humoral immunity.

Source

Arthritis Research & Therapy. 14(2):R57, 2012 Mar 12.

VI 1

Status

MEDLINE

Authors

Teng YK; Wheater G; Hogan VE; Stocks P; Levarht EW; Huizinga TW; Toes RE; van Laar JM.

Authors Full Name

Teng, Y K Onno; Wheater, Gillian; Hogan, Vanessa E; Stocks, Philip; Levarht, E W Nivine; Huizinga, Tom W J; Toes, Rene E M; van Laar, Jacob M.

Institution

Teng, Y K Onno. Department of Rheumatology, C1-R, Leiden University Medical Center, PO Box 9600, NL-2300 RC Leiden, The Netherlands. y.k.o.teng@lumc.nl

Abstract

INTRODUCTION: B-cell depletion has become a common treatment strategy in anti-TNF-refractory rheumatoid arthritis (RA). Although the exact mechanism of how B-cell depletion leads to clinical amelioration in RA remains to be elucidated, repetitive treatment with B-cell-depleting agents leading to long-term B-cell depletion has been reported to be beneficial. The latter has led to the hypothesis that the beneficial effects of B-cell depletion might act through their influence on pathogenic autoreactive plasma cells.

METHODS: In this study, we investigated the effects of a fixed retreatment regimen with anti-CD20 mAbs on the humoral (auto)immune system in a cohort of therapy-refractory RA patients.

RESULTS: Fixed retreatment led to long-term B-cell depletion in peripheral blood, bone marrow and, to a lesser extent, synovium. Also, pathologic autoantibody secretion (that is, anticitrullinated peptide antibodies (ACPAs)) was more profoundly affected by long-term depletion than by physiological protective antibody secretion (that is, against measles, mumps and rubella). This was further illustrated by a significantly shorter estimated life span of ACPA-IgG secretion compared to total IgG secretion as well as protective antibody secretion.

CONCLUSION: By studying plasma cell function during an extensive 2-year period of B-cell depletion, autoantibody secretion was significantly shorter-lived than physiologically protective antibody secretion. This suggests that the longevity of autoreactive plasma cells is different from protective long-lived plasma cells and might indicate a therapeutic window for therapies that target plasma cells.

Publication Type

Clinical Trial, Phase I. Clinical Trial, Phase II. Journal Article. Research Support, Non-U.S. Gov't.

Year of Publication

2012

<497>

Unique Identifier

22370806

Title

[Practical problems by implementation of vaccination recommendations]. [German]

Source

Zeitschrift fur Rheumatologie. 71(2):147-50, 153-5, 2012 Feb.

VI 1

Status

MEDLINE

Authors

Fendler C; Saracbasi E; Dybowski F; Heldmann F; Braun J.

Authors Full Name

Fendler, C; Saracbasi, E; Dybowski, F; Heldmann, F; Braun, J.

Institution

Fendler, C. Rheumazentrum Ruhrgebiet Herne, Ruhr-Universitat Bochum, Herne, Deutschland. fendler@rheumazentrum-ruhrgebiet.de

Abstract

Patients with inflammatory rheumatic diseases are known to have an increased risk of infections due to the rheumatic disease itself and due to therapy with immunosuppressive agents. The most important procedure to prevent infections is vaccinations, which are usually well-tolerated. The German National Commission for Immunization (STIKO) has published recommendations for patients with an immunodeficiency. The German Society of Rheumatology (DGRh) has generally implemented these recommendations for patients with chronic inflammatory rheumatic diseases. The immunization status of patients with rheumatic diseases is of increasing importance in routine patient care because some of the recently approved drugs may influence the strength of the immune response to vaccination. However, there is almost no information about the current immunization status and the willingness of patients with rheumatic diseases to undergo vaccination procedures in Germany. There are also no epidemiologic data on the implementation of recommendations for immunization at the level of general practitioners. Here we present the results of a prospective study on the efficacy of standardized recommendations for immunization given to different patient groups with rheumatic diseases treated in a hospital specialized in rheumatology.

Publication Type

English Abstract. Journal Article.

Year of Publication

2012

<498>

Unique Identifier

22341852

Title

Immune regulation and B-cell depletion therapy in patients with primary Sjogren's syndrome. [Review]

Source

Journal of Autoimmunity. 39(1-2):103-11, 2012 Aug.

VI 1

Status

MEDLINE

Authors

Abdulahad WH; Kroese FG; Vissink A; Bootsma H.

Authors Full Name

Abdulahad, Wayel H; Kroese, Frans G M; Vissink, Arjan; Bootsma, Hendrika.

Institution

Abdulahad, Wayel H. Department Rheumatology and Clinical Immunology, University Medical Center Groningen, University of Groningen, P.O. Box 30.001, 9700 RB Groningen, The Netherlands.

Abstract

Primary Sjogren's syndrome (pSS) is an autoimmune exocrinopathy characterized by chronic inflammation and destruction of the salivary and lacrimal glands. B- and T- lymphocyte infiltrations in the salivary glands with development of germinal center-like structures are characteristic for pSS. Overexpression of soluble factors, such as interferon alpha (IFNalpha) and B-cell activating factor (BAFF), are supposed to be important factors in the initiation and continuation of this disorder. The efficacy and success of B-cell depleting therapy in reducing disease activity in pSS patients for about six to nine months supports the notion that B-cells are major key players in disease manifestation of pSS. In addition to B-cells, also Th-cells (mainly Th17) seem to be involved in the pathogenetic process. In this review, we will discuss recent research findings regarding the cytokines IFNalpha and BAFF as wells as the role of B- and T-cells in pSS. Emphasis will be put on the impact of B-cell depletion therapy as well as on the presumed impact of therapies aimed for targeting BAFF, either as a sole modality or as a combined treatment with B-cell depletion. Copyright © 2012 Elsevier Ltd. All rights reserved.

Publication Type

Journal Article. Review.

Year of Publication

2012

<499>

Unique Identifier

22253030

Title

Total serum immunoglobulin levels in patients with RA after multiple B-cell depletion cycles based on rituximab: relationship with B-cell kinetics.

Source

Rheumatology. 51(5):833-40, 2012 May.

VI 1

Status

MEDLINE

Authors

De La Torre I; Leandro MJ; Valor L; Becerra E; Edwards JC; Cambridge G.

Authors Full Name

De La Torre, Inmaculada; Leandro, Maria J; Valor, Lara; Becerra, Elena; Edwards, Jonathan C W; Cambridge, Geraldine.

Institution

De La Torre, Inmaculada. Division of Rheumatology, Department of Medicine, University College London, Rayne Institute, 5 University Street, London WC1E 6JF, UK.

Abstract

OBJECTIVE: To investigate whether the incidence of secondary hypogammaglobulinaemia in patients with RA following rituximab was related to patterns of B-cell return and relapse.

METHODS: CD19(+) B-cell and serum immunoglobulin (sIg) determinations were done every 2 or 3 months in 137 consecutive patients treated with one or more courses of rituximab-based B-cell depletion therapy. The pattern of B-cell return, either concordant or discordant with relapse, was also recorded.

RESULTS: There were 119 responders. Before treatment, three patients had low IgM and four had low IgG. After the first cycle, low IgM or IgG was present in 9.2% (11/119) and 11.8% (14/119) of the patients, respectively, increasing to 38.8% (8/18) and 22.2% (4/18) after five cycles. The mean percent maximum sIg decrease/cycle was relatively constant. The CD19(+) B-

cell count at repopulation was not correlated with immunoglobulin (Ig) levels after each cycle. Patients discordant for B-cell return and relapse developed significantly lower serum IgM and more low IgM episodes than concordant patients (P < 0.05).

CONCLUSION: Patients with lower baseline sIg levels tended to develop persistent IgM and IgG hypogammaglobulinaemia, resulting from an accumulation of incremental decreases after repeat cycles. This was not due to lower numbers of returning B cells in those developing low sIgs. The association of low IgM in patients with a discordant pattern of relapse suggests that underlying defects in B cells relating to survival and maturation into Ig-secreting cells, as well as attrition of IgG plasma cells may be contributing to low sIg levels in some patients.

Publication Type

Journal Article. Research Support, Non-U.S. Gov't.

Year of Publication

2012

<500>

Unique Identifier

22241404

Title

Biological therapy for rheumatoid arthritis: where are we now?. [Review]

Source

British Journal of Hospital Medicine. 73(1):12-8, 2012 Jan.

VI 1

Status

MEDLINE

Authors

Horton SC; Emery P.

Authors Full Name

Horton, Sarah C; Emery, Paul.

Institution

Horton, Sarah C. Division of Musculoskeletal Disease, Leeds Institute of Molecular Medicine, University of Leeds and NIHR Leeds Musculoskeletal Biomedical Research Unit, Leeds Teaching Hospitals NHS Trust, Leeds LS7 4SA, UK.

Abstract

Since the introduction of targeted biological therapies, the implications of a new diagnosis of rheumatoid arthritis have changed dramatically. There are now several therapeutic options available for these patients and the target of treatment - remission - is now a realistic goal.

Publication Type

Journal Article. Review.

Year of Publication

2012

<501>

Unique Identifier

22235050

Title

Autoimmune response following influenza vaccination in patients with autoimmune inflammatory rheumatic disease.

Source

Lupus. 21(2):175-83, 2012 Feb.

VI 1

Status

MEDLINE

Authors

Perdan-Pirkmajer K; Thallinger GG; Snoj N; Cucnik S; Zigon P; Kveder T; Logar D; Praprotnik S; Tomsic M; Sodin-Semrl S; Ambrozic A.

Authors Full Name

Perdan-Pirkmajer, K; Thallinger, G G; Snoj, N; Cucnik, S; Zigon, P; Kveder, T; Logar, D; Praprotnik, S; Tomsic, M; Sodin-Semrl, S; Ambrozic, A.

Institution

Perdan-Pirkmajer, K. University Medical Centre Ljubljana, Department of Rheumatology, Ljubljana, Slovenia.

Abstract

Vaccines have undoubtedly brought overwhelming benefits to mankind and are considered safe and effective. Nevertheless, they can occasionally stimulate autoantibody production or even a recently defined syndrome known as autoimmune/inflammatory syndrome induced by adjuvants

(ASIA). There is scarce data regarding autoimmune response after seasonal/influenza A (H1N1) vaccine in patients with autoimmune inflammatory rheumatic disease (AIRD). The objective of our study was therefore to determine autoimmune response in a large group of AIRD patients vaccinated against seasonal and/or H1N1 influenza. We conducted a prospective cohort study with a 6-month follow-up. Two-hundred and eighteen patients with AIRD (50 vaccinated against seasonal influenza, six against H1N1, 104 against both, 58 non-vaccinated controls) and 41 apparently healthy controls (nine vaccinated against seasonal influenza, three against H1N1, 18 against both, 11 non-vaccinated controls) were included. Blood samples were taken and screened for autoantibodies [antinuclear antibody (ANA), anti-extractable nuclear antigen (anti-ENA), anticardiolipin (aCL) IgG/IgM antibodies, anti-beta 2-glycoprotein I (anti-beta2GPI)] at inclusion in the study, before each vaccination, 1 month after the last vaccination and 6 months after inclusion. For non-vaccinated participants (patients and healthy controls) blood samples were taken at the time of inclusion in the study and 6 months later. We report that after the administration of seasonal/H1N1 vaccine there were mostly transient changes in autoantibody production in AIRD patients and in healthy participants. However, a small subset of patients, especially ANA-positive patients, had a tendency towards anti-ENA development. Although no convincing differences between the seasonal and H1N1 vaccines were observed, our results imply that there might be a slight tendency of the H1N1 vaccine towards aCL induction. Although seasonal and H1N1 vaccines are safe and effective, they also have the potential to induce autoantibodies in selected AIRD patients and healthy adults. Follow-up of such individuals is proposed and further research is needed.

Publication Type

Journal Article. Research Support, Non-U.S. Gov't.

Year of Publication

2012

<502>

Unique Identifier

22235048

Title

Immunization of patients with autoimmune inflammatory rheumatic diseases (the EULAR recommendations). [Review]

Source

Lupus. 21(2):162-7, 2012 Feb.
VI 1
Status
MEDLINE
Authors
van Assen S; Bijl M.
Authors Full Name
van Assen, S; Bijl, M.
Institution
van Assen, S. Department of Internal Medicine, Division of Infectious Diseases, University Medical Center Groningen, University of Groningen, The Netherlands.
Abstract
The European League Against Rheumatism (EULAR) recommendations for vaccination in adult patients with autoimmune inflammatory rheumatic diseases (AIIRD) have been recently published. These evidence-based recommendations were based on existing literature in combination with expert opinion. Although patients with AIIRD are at increased risk of suffering from (complicated) infectious diseases--and vaccination seems a tool to reduce this risk--still many questions and controversies remain for the individual patient. In this overview, taking influenza as an example, the background of the recommendations, their clinical implications, and the direction of future research are discussed. The increase in knowledge on vaccine-preventable infections will allow us to further improve vaccination strategies.
Publication Type
Journal Article. Review.
Year of Publication
2012

<503>
Unique Identifier
22235046
Title
Giant cell arteritis and polymyalgia rheumatica after influenza vaccination: report of 10 cases and review of the literature. [Review]
Source

Lupus. 21(2):153-7, 2012 Feb.
VI 1
Status
MEDLINE
Authors
Soriano A; Verrecchia E; Marinaro A; Giovinale M; Fonnesu C; Landolfi R; Manna R.
Authors Full Name
Soriano, A; Verrecchia, E; Marinaro, A; Giovinale, M; Fonnesu, C; Landolfi, R; Manna, R.
Institution
Soriano, A. Clinical Autoimmunity Unit, Catholic University of the Sacred Heart, Rome, Italy.
Abstract
Giant cell arteritis (GCA) and polymyalgia rheumatica (PMR) are inflammatory rheumatic diseases common in people over the age of 50 years. Herein, we report 10 cases of previously healthy subjects who developed GCA/PMR within 3 months of influenza vaccination (Inf-V). A Medline search uncovered additional 11 isolated cases of GCA/PMR occurring after Inf-V. We discuss the role of individual susceptibility, the potential function of immune adjuvants as triggers of autoimmunity post-vaccination, and the correlation of our observation with the 'ASIA' syndrome, i.e. autoimmune/inflammatory syndrome induced by adjuvants and including post-vaccination phenomena.
Publication Type
Case Reports. Journal Article. Review.
Year of Publication
2012

<504>
Unique Identifier
22231738
Title
Interleukin-1 receptor mediates the interplay between CD4+ T cells and ocular resident cells to promote keratinizing squamous metaplasia in Sjogren's syndrome.
Source
Laboratory Investigation. 92(4):556-70, 2012 Apr.
VI 1

Status

MEDLINE

Authors

Chen YT; Lazarev S; Bahrami AF; Noble LB; Chen FY; Zhou D; Gallup M; Yadav M; McNamara NA.

Authors Full Name

Chen, Ying-Ting; Lazarev, Stanislav; Bahrami, Ahmad F; Noble, Lisa B; Chen, Feeling Y T; Zhou, Delu; Gallup, Marianne; Yadav, Mahesh; McNamara, Nancy A.

Institution

Chen, Ying-Ting. Francis I Proctor Foundation, University of California at San Francisco, San Francisco, CA 94143, USA.

Abstract

Keratinizing squamous metaplasia (SQM) of the ocular mucosal epithelium is a blinding corneal disease characterized by the loss of conjunctival goblet cells (GCs), pathological ocular surface keratinization and tissue recruitment of immune cells. Using the autoimmune regulator (Aire)-deficient mouse as a model for Sjogren's syndrome (SS)-associated SQM, we identified CD4(+) T lymphocytes as the main immune effectors driving SQM and uncovered a pathogenic role for interleukin-1 (IL-1). IL-1, a pleiotropic cytokine family enriched in ocular epithelia, governs tissue homeostasis and mucosal immunity. Here, we used adoptive transfer of autoreactive CD4(+) T cells to dissect the mechanism whereby IL-1 promotes SQM. CD4(+) T cells adoptively transferred from both Aire knockout (KO) and Aire/IL-1 receptor type 1 (IL-1R1) double KO donors conferred SQM to severe-combined immunodeficiency (scid) recipients with functional IL-1R1, but not scid recipients lacking IL-1R1. In the lacrimal gland, IL-1R1 was primarily immunolocalized to ductal epithelium surrounded by CD4(+) T cells. In the eye, IL-1R1 was expressed on local mucosal epithelial and stromal cells, but not on resident antigen-presenting cells or infiltrating immune cells. In both tissues, autoreactive CD4(+) T-cell infiltration was only observed in the presence of IL-1R1-postive resident cells. Moreover, persistent activation of IL-1R1 signaling led to chronic immune-mediated inflammation by retaining CD4(+) T cells in the local microenvironment. Following IL-1R1-dependent infiltration of CD4(+) T cells, we observed SQM hallmarks in local tissues-corneal keratinization, conjunctival GC mucin acidification and epithelial cell hyperplasia throughout the ocular surface mucosa. Proinflammatory IL-1 expression in ocular epithelial cells significantly correlated with reduced tear secretion, while CD4(+) T-cell infiltration of the lacrimal gland predicted the development of ocular SQM. Collectively, data in this study indicated a central role for IL-1 in orchestrating a functional interplay between immune cells and resident cells of SS-targeted tissues in the pathogenesis of SQM.

Publication Type

Journal Article. Research Support, N.I.H., Extramural.

Year of Publication
2012

<505>
Unique Identifier
22190690
Title
Pro-resolution immunological networks: binding immunoglobulin protein and other resolution-associated molecular patterns. [Review]
Source
Rheumatology. 51(5):780-8, 2012 May.
VI 1
Status
MEDLINE
Authors
Shields AM; Thompson SJ; Panayi GS; Corrigall VM.
Authors Full Name
Shields, Adrian M; Thompson, Stephen J; Panayi, Gabriel S; Corrigall, Valerie M.
Institution
Shields, Adrian M. Department of Academic Rheumatology, Centre for Molecular and Cellular Biology of Inflammation, 1st Floor New Hunts House, Guy's Campus, King's College London, London SE 1UL, UK.
Abstract
Appropriate regulation and subsequent resolution of acute inflammatory events is critical to the prevention of autoinflammatory diseases. Indeed, the chronic inflammation observed in diseases such as RA is at least partially consequent on the failure of endogenous immunoregulation. Current RA therapies (e.g. anti-TNF-alpha inhibitors and MTX) inhibit components of the inflammatory disease process without directly promoting the resolution of inflammation. We propose that the next generation of RA therapeutics will complement and augment endogenous immunoregulatory and pro-resolution immunological networks, thus promoting the definitive resolution of inflammation rather than temporary immunological control. Of particular interest with respect to this therapeutic approach is binding immunoglobulin protein [BiP; also known as glucose-regulated protein-78 (GRP78)], a member of the recently defined resolution-associated

molecular pattern (RAMP) family of molecules. In this review, we consider the preclinical evidence from experiments in mouse and man that suggests BiP and other members of the RAMP family have the potential to herald a new generation of immunotherapeutics.
Publication Type
  Journal Article. Research Support, Non-U.S. Gov't. Review.
Year of Publication
  2012

<506>
Unique Identifier
  22180424
Title
  Acute myeloid leukemia developing in patients with autoimmune diseases. [Review]
Source
  Haematologica. 97(6):805-17, 2012 Jun.
VI 1
Status
  MEDLINE
Authors
  Ramadan SM; Fouad TM; Summa V; Hasan SKh; Lo-Coco F.
Authors Full Name
  Ramadan, Safaa M; Fouad, Tamer M; Summa, Valentina; Hasan, Syed Kh; Lo-Coco, Francesco.
Institution
  Ramadan, Safaa M. Department of Medical Oncology, NCI-Cairo University, 11796 Cairo, Egypt. safaanci@yahoo.com
Abstract
  Therapy-related acute myeloid leukemia is an unfortunate complication of cancer treatment, particularly for patients with highly curable primary malignancies and favorable life expectancy. The risk of developing therapy-related acute myeloid leukemia also applies to patients with non-malignant conditions, such as autoimmune diseases treated with cytotoxic and/or immunosuppressive agents. There is considerable evidence to suggest that there is an increased occurrence of hematologic malignancies in patients with autoimmune diseases compared to the

general population, with a further increase in risk after exposure to cytotoxic therapies. Unfortunately, studies have failed to reveal a clear correlation between leukemia development and exposure to individual agents used for the treatment of autoimmune diseases. Given the dismal outcome of secondary acute myeloid leukemia and the wide range of available agents for treatment of autoimmune diseases, an increased awareness of this risk and further investigation into the pathogenetic mechanisms of acute leukemia in autoimmune disease patients are warranted. This article will review the data available on the development of acute myeloid leukemia in patients with autoimmune diseases. Possible leukemogeneic mechanisms in these patients, as well as evidence supporting the association of their primary immunosuppressive status and their exposure to specific therapies, will also be reviewed. This review also supports the idea that it may be misleading to label leukemias that develop in patients with autoimmune diseases who are exposed to cytotoxic agents as 'therapy-related leukemias'. A better understanding of the molecular defects in autoimmune disease patients who develop acute leukemia will lead to a better understanding of the association between these two diseases entities.

Publication Type

Journal Article. Research Support, Non-U.S. Gov't. Review.

Year of Publication

2012

<507>

Unique Identifier

22171015

Title

Protective effect of A/H1N1 vaccination in immune-mediated disease--a prospectively controlled vaccination study.

Source

Rheumatology. 51(4):695-700, 2012 Apr.

VI 1

Status

MEDLINE

Authors

Adler S; Krivine A; Weix J; Rozenberg F; Launay O; Huesler J; Guillevin L; Villiger PM.

Authors Full Name
Adler, Sabine; Krivine, Anne; Weix, Janine; Rozenberg, Flore; Launay, Odile; Huesler, Juerg; Guillevin, Loic; Villiger, Peter M.
Institution
Adler, Sabine. Department of Rheumatology, Clinical Immunology and Allergology, University Hospital and University of Bern, Freiburgstrasse, Bern 3010, Switzerland.
Abstract
OBJECTIVES: To assess the 2009 influenza vaccine A/H1N1 on antibody response, side effects and disease activity in patients with immune-mediated diseases.

METHODS: Patients with RA, SpA, vasculitis (VAS) or CTD (n = 149) and healthy individuals (n = 40) received a single dose of adjuvanted A/H1N1 influenza vaccine. Sera were obtained before vaccination, and 3 weeks, 6 weeks and 6 months thereafter. A/H1N1 antibody titres were measured by haemagglutination inhibition (HAI) assay. Seroprotection was defined as specific antibody titre >= 1 : 40, seroconversion as 4-fold increase in antibody titre.

RESULTS: Titres increased significantly in patients and controls with a maximum at Week 3, declining to levels below protection at Month 6 (P < 0.001). Seroprotection was more frequently reached in SpA and CTD than in RA and VAS (80 and 82% and 57 and 47%, respectively). There was a significantly negative impact by MTX (P < 0.001), rituximab (P = 0.0031) and abatacept (P = 0.045). Other DMARDs, glucocorticoids and TNF blockers did not significantly suppress response (P = 0.06, 0.11 and 0.81, respectively). A linear decline in response was noted in patients with increasing age (P < 0.001). Disease reactivation possibly related to vaccination was suspected in 8/149 patients. No prolonged side effects or A/H1N1 infections were noted.

CONCLUSIONS: The results show that vaccination response is a function of disease type, intensity and character of medication and age. A single injection of adjuvanted influenza vaccine is sufficient to protect a high percentage of patients. Therefore, differential vaccination recommendations might in the future reduce costs and increase vaccination acceptance.
Publication Type
Controlled Clinical Trial. Journal Article. Research Support, Non-U.S. Gov't.
Year of Publication
2012

<508>

Unique Identifier

22132879

Title

Translational Mini-Review Series on B cell subsets in disease. Transitional B cells in systemic lupus erythematosus and Sjogren's syndrome: clinical implications and effects of B cell-targeted therapies. [Review]

Source

Clinical & Experimental Immunology. 167(1):7-14, 2012 Jan.

VI 1

Status

MEDLINE

Authors

Vossenkamper A; Lutalo PM; Spencer J.

Authors Full Name

Vossenkamper, A; Lutalo, P M K; Spencer, J.

Institution

Vossenkamper, A. Centre for Immunology and Infectious Disease, Barts and The London School of Medicine and Dentistry, Blizard Institute, London, UK. a.vossenkaemper@qmul.ac.uk

Abstract

Systemic lupus erythematosus (SLE) and Sjogren's syndrome are autoimmune disorders which are characterized by a disturbed B cell homeostasis which leads ultimately to dysfunction of various organs. One of the B cell subsets that appear in abnormal numbers is the population of transitional B cells, which is increased in the blood of patients with SLE and Sjogren's syndrome. Transitional B cells are newly formed B cells. In mice, transitional B cells undergo selection checks for unwanted specificity in the bone marrow and the spleen in order to eliminate autoreactive B cells from the circulating naive B cell population. In humans, the exact anatomical compartments and mechanisms of the specificity check-points for transitional B cells remain unclear, but appear to be defective in SLE and Sjogren's syndrome. This review aims to highlight the current understanding of transitional B cells and their defects in the two disorders before and after B cell-targeted therapies. Copyright © 2011 The Authors. Clinical and Experimental Immunology © 2011 British Society for Immunology.

Publication Type

Journal Article. Research Support, Non-U.S. Gov't. Review.

Year of Publication

2012

<509>

Unique Identifier

22130974

Title

Avidity maturation of anti-citrullinated protein antibodies in rheumatoid arthritis.

Source

Arthritis & Rheumatism. 64(5):1323-8, 2012 May.

VI 1

Status

MEDLINE

Authors

Suwannalai P; van de Stadt LA; Radner H; Steiner G; El-Gabalawy HS; Zijde CM; van Tol MJ; van Schaardenburg D; Huizinga TW; Toes RE; Trouw LA.

Authors Full Name

Suwannalai, P; van de Stadt, L A; Radner, H; Steiner, G; El-Gabalawy, H S; Zijde, C M Jol-van der; van Tol, M J; van Schaardenburg, D; Huizinga, T W J; Toes, R E M; Trouw, L A.

Institution

Suwannalai, P. Leiden University Medical Center, Leiden, The Netherlands.

Abstract

OBJECTIVE: Anti-citrullinated protein antibodies (ACPAs) are highly specific for rheumatoid arthritis (RA) and are present years before the onset of symptoms. The avidity of autoantibodies can have a strong impact on their effector potency. This study was undertaken to analyze the avidity of ACPAs in serum samples obtained from ACPA-positive healthy individuals (predisease), patients with early disease, and patients with established RA as well as the avidity maturation over time in samples from healthy subjects who later developed RA.

METHODS: We measured ACPA avidity in serum samples from ACPA-positive healthy individuals, symptomatic individuals, and patients with established RA in 5 collections from The Netherlands, Canada, and Austria. We determined the dynamics of avidity maturation of ACPAs from the predisease stage to established disease in 1 case from the native North American population and in 10 cases from a Dutch blood donor cohort.

RESULTS: The overall ACPA response was characterized by low-avidity antibodies. Higher-avidity ACPAs were observed in symptomatic patients only, while low-avidity ACPAs were observed in both healthy subjects and patients. In longitudinal samples obtained from subjects prior to disease onset, ACPA avidity increased over time until disease onset. No further avidity maturation was observed after disease onset.

CONCLUSION: Our findings indicate that avidity maturation of the ACPA response takes place prior to disease onset. Copyright © 2012 by the American College of Rheumatology.

Publication Type

Comparative Study. Journal Article. Research Support, Non-U.S. Gov't.

Year of Publication

2012

<510>

Unique Identifier

22119345

Title

Vaccination in patients with chronic or autoimmune rheumatic diseases: the ego, the id and the superego.

Source

Joint, Bone, Spine: Revue du Rhumatisme. 79(1):1-3, 2012 Jan.

VI 1

Status

MEDLINE

Authors

Perricone C; Agmon-Levin N; Valesini G; Shoenfeld Y.

Authors Full Name

Perricone, Carlo; Agmon-Levin, Nancy; Valesini, Guido; Shoenfeld, Yehuda.

Publication Type

Editorial.

Year of Publication

2012

<511>

Unique Identifier

22056979

Title

Controversies in rheumatism and autoimmunity.

Source

Autoimmunity Reviews. 11(8):555-7, 2012 Jun.

VI 1

Status

MEDLINE

Authors

Doria A; Putterman C; Sarzi-Puttini P; Szekanecz Z; Shoenfeld Y.

Authors Full Name

Doria, Andrea; Putterman, Chaim; Sarzi-Puttini, Piercarlo; Szekanecz, Zoltan; Shoenfeld, Yehuda.

Institution

Doria, Andrea. Division of Rheumatology, Department of Clinical and Experimental Medicine, University of Padova, Italy. adoria@unipd.it

Publication Type

Editorial.

Year of Publication

2012

<512>

Unique Identifier

22037117

Title

To switch or not to switch after a poor response to a TNFalpha blocker? It is not only a matter of ACR20 OR ACR50. [Review]

Source

Autoimmunity Reviews. 11(8):558-62, 2012 Jun.

VI 1

Status

MEDLINE

Authors

Buch MH; Rubbert-Roth A; Ferraccioli G.

Authors Full Name

Buch, Maya H; Rubbert-Roth, Andrea; Ferraccioli, Gianfranco.

Institution

Buch, Maya H. Section of Musculoskeletal Disease, Leeds Institute of Molecular Medicine, University of Leeds, UK.

Abstract

The introduction in the therapeutic armamentarium of TNF inhibitors (TNFi) has greatly advanced the chance of obtaining a control of clinical manifestations and of structural damage progression in an important proportion of patients with rheumatoid arthritis (RA) Methotrexate (MTX)-poor responders. However not more than 50% of TNFi treated patients can reach relevant clinical benefits. Therefore the unmet medical question is: should we continue the therapeutic approach with a second or a third TNFi, or should we use other drugs, and change the mode of action of the second drug? These are practical issues that still do not have a definite answer. The real problem is that up to this moment no real biomarker is available to make the appropriate choice. The only clear-cut biomarker is represented by the positivity of rheumatoid factor (RF) or anti citrullinated peptide autoantibodies (ACPA). Seropositive patients seem to respond better than seronegative ones to B cell depletion therapy (Rituximab). This paper discusses the pros and cons of switching or swapping in RA patients poorly responder to the first TNFi. Copyright © 2011 Elsevier B.V. All rights reserved.

Publication Type

Journal Article. Review.

Year of Publication

2012

<513>

Unique Identifier

22037116

Title

Vaccination of patients with auto-immune inflammatory rheumatic diseases requires careful benefit-risk assessment. [Review]

Source

Autoimmunity Reviews. 11(8):572-6, 2012 Jun.

VI 1

Status

MEDLINE

Authors

Bijl M; Agmon-Levin N; Dayer JM; Israeli E; Gatto M; Shoenfeld Y.

Authors Full Name

Bijl, M; Agmon-Levin, N; Dayer, J-M; Israeli, E; Gatto, M; Shoenfeld, Y.

Institution

Bijl, M. Department of Rheumatology and Clinical Immunology, University Medical Center Groningen, University of Groningen, The Netherlands. m.bijl@reuma.umcg.nl

Abstract

Will vaccination raise the incidence of autoimmune diseases, what is the impact of increasingly crowded vaccination schedules, the vaccination in age groups and the risk of coincidental temporal association? All these issues are still under debate. However, for the time being, to avoid confusion in the medical community and the media, we have to adhere to guidelines established consensually by experts while ensuring a strict surveillance and reporting possible side effects. Recommendation for vaccination in patients with autoimmune inflammatory rheumatic diseases (AIIRD) based on the currently available evidence and expert opinion were recently formulated by an EULAR task force. Major recommendations for AIIRD include: i) vaccination should ideally be administered during stable disease; ii) influenza vaccination and pneumococcal vaccination should be strongly considered; iii) vaccination can be administered during the use of DMARDs and TNF-inhibitors, but before starting rituximab; iv) live attenuated vaccines should be avoided whenever possible in immunosuppressed patients; v) BCG vaccination is not recommended. Copyright © 2011 Elsevier B.V. All rights reserved.

Publication Type

Journal Article. Review.

Year of Publication

2012

<514>

Unique Identifier

22028728

Title

Polymerized-type I collagen induces upregulation of Foxp3-expressing CD4 regulatory T cells and downregulation of IL-17-producing CD4+ T cells (Th17) cells in collagen-induced arthritis.

Source

Clinical & Developmental Immunology. 2012:618608, 2012.

VI 1

Status

MEDLINE

Authors

Furuzawa-Carballeda J; Macip-Rodriguez P; Galindo-Feria AS; Cruz-Robles D; Soto-Abraham V; Escobar-Hernandez S; Aguilar D; Alpizar-Rodriguez D; Ferez-Blando K; Llorente L.

Authors Full Name

Furuzawa-Carballeda, Janette; Macip-Rodriguez, Perla; Galindo-Feria, Angeles S; Cruz-Robles, David; Soto-Abraham, Virgina; Escobar-Hernandez, Sergio; Aguilar, Diana; Alpizar-Rodriguez, Deshire; Ferez-Blando, Karen; Llorente, Luis.

Institution

Furuzawa-Carballeda, Janette. Department of Immunology and Rheumatology, Instituto Nacional de Ciencias Medicas y Nutricion Salvador Zubiran, Vasco de Quiroga No. 15, Colonia Seccion XVI, Tlalpan, 14000 Mexico City, DF, Mexico. jfuruzawa@gmail.com

Abstract

Previous studies showed that polymerized-type I collagen (polymerized collagen) exhibits potent immunoregulatory properties. This work evaluated the effect of intramuscular administration of polymerized collagen in early and established collagen-induced arthritis (CIA) in mice and analyzed changes in Th subsets following therapy. Incidence of CIA was of 100% in mice challenged with type II collagen. Clinimorphometric analysis showed a downregulation of inflammation after administration of all treatments ($P < 0.05$). Histological analysis showed that the CIA-mice group had extensive bone erosion, pannus and severe focal inflammatory infiltrates. In contrast, there was a remarkable reduction in the severity of arthritis in mice under polymerized collagen, methotrexate or methotrexate/polymerized collagen treatment. Polymerized Collagen but not methotrexate induced tissue joint regeneration. Polymerized Collagen and methotrexate/polymerized collagen but not methotrexate alone induces downregulation of CD4(+)/IL17A(+) T cells and upregulation of Tregs and CD4(+)/IFN-gamma(+) T cells. Thus,

Polymerized Collagen could be an effective therapeutic agent in early and established rheumatoid arthritis by exerting downregulation of autoimmune inflammation.
Publication Type
Journal Article. Research Support, Non-U.S. Gov't.
Year of Publication
2012

<515>
Unique Identifier
21941580
Title
RP105-negative B cells in systemic lupus erythematosus. [Review]
Source
Clinical & Developmental Immunology. 2012:259186, 2012.
VI 1
Status
MEDLINE
Authors
Koarada S; Tada Y.
Authors Full Name
Koarada, Syuichi; Tada, Yoshifumi.
Institution
Koarada, Syuichi. Division of Rheumatology, Faculty of Medicine, Saga University, 5-1-1 Nabeshima, Saga 849-8501, Japan. koarada@post.saga-med.ac.jp
Abstract
Systemic lupus erythematosus (SLE) is a multisystem disease characterized by B cells producing autoantibodies against nuclear proteins and DNA, especially anti-double-strand DNA (dsDNA) antibodies. RP105 (CD180), the toll-like receptor- (TLR-) associated molecule, is expressed on normal B cells. However, RP105-negative B cells increase in peripheral blood from patients with active SLE. RP105 may regulate B-cell activation, and RP105-negative B cells produce autoantibodies and take part in pathophysiology of SLE. It is possible that targeting RP105-negative B cells is one of the treatments of SLE. In this paper, we discuss the RP105 biology and clinical significance in SLE.

Publication Type
  Journal Article. Research Support, Non-U.S. Gov't. Review.
Year of Publication
  2012

<516>
Unique Identifier
  21403999
Title
  Skin manifestations induced by TNF-alpha inhibitors in juvenile idiopathic arthritis.
Source
  Clinical Reviews in Allergy & Immunology. 42(2):131-4, 2012 Apr.
VI 1
Status
  MEDLINE
Authors
  Pontikaki I; Shahi E; Frasin LA; Gianotti R; Gelmetti C; Gerloni V; Meroni PL.
Authors Full Name
  Pontikaki, Irene; Shahi, Edit; Frasin, Lucretia Adina; Gianotti, Raffaele; Gelmetti, Carlo; Gerloni, Valeria; Meroni, Pier Luigi.
Institution
  Pontikaki, Irene. Unit of Pediatric Rheumatology, G.Pini Institute, Chair of Rheumatology, University of Milan, Milan, Italy.
Abstract
  The tumor necrosis factor alpha (TNFalpha) inhibitors have been used with good clinical results in the treatment of juvenile idiopathic arthritis (JIA). Anti TNFalpha therapy is generally well tolerated. Besides the site injection reactions, other various cutaneous manifestations have been encountered as adverse events. Here, we report four young patients receiving treatment with anti-TNFalpha (infliximab, adalimumab, and etanercept) for JIA developing different skin manifestations more than 1 year after the initiation of therapy. They underwent a dermatological exam. All four patients were ACR-Ped 30 responders to anti-TNF drugs. The first patient developed cutaneous vasculitis, the second one had lichen planus manifestations, while the third and the fourth developed psoriatic palmoplantar pustulosis accompanied by plaque-type psoriasis

localized to the scalp. None of the patients had a personal or family history of dermatological diseases. In the first two patients, skin lesions healed with topical treatment after the discontinuation of anti-TNF agent, while psoriatic lesions did not resolve despite discontinuation of the drug and dermatological treatment. TNF inhibition can be both anti-inflammatory and pro-inflammatory. Cutaneous manifestations could be considered as a paradoxical adverse event of the anti-TNF-alpha treatment not only in rheumatoid arthritis but also in juvenile idiopathic arthritis.

Publication Type

Case Reports. Journal Article.

Year of Publication

2012

<517>

Unique Identifier

21327432

Title

Vaccination survey in patients with rheumatoid arthritis: a cross-sectional study.

Source

Rheumatology International. 32(6):1533-9, 2012 Jun.

VI 1

Status

MEDLINE

Authors

Feuchtenberger M; Kleinert S; Schwab S; Roll P; Scharbatke EC; Ostermeier E; Voll RE; Schafer A; Tony HP.

Authors Full Name

Feuchtenberger, Martin; Kleinert, Stefan; Schwab, Sven; Roll, Petra; Scharbatke, Eva Christina; Ostermeier, Eva; Voll, Reinhard E; Schafer, Arne; Tony, Hans-Peter.

Institution

Feuchtenberger, Martin. Medizinische Klinik, Universitat Wuerzburg, Schwerpunkt Rheumatologie/Klinische Immunologie, Oberdurrbacherstrasse 6, 97080, Wuerzburg, Germany. Feuchtenbe_M@klinik.uni-wuerzburg.de

Abstract

The objective of this study is to evaluate the vaccination status in rheumatoid arthritis (RA) patients during routine clinical practice, data from a German non-interventional cross-sectional study. In this prospective study, patients with rheumatoid arthritis were interviewed using a standardized questionnaire focusing on vaccination. Available vaccination documents were evaluated, and titers for common vaccination antigens (hepatitis B, rubella, mumps, measles, diphtheria, tetanus) were analyzed with special regard to the underlying treatment and age of patients. A total of 301 RA patients treated with conventional DMARDs alone (cohort I, n = 125), TNF-blocking agents (cohort II, n = 117), or B-cell depletion with rituximab (cohort III, n = 59) have been studied. Significantly more patients in the biologic cohorts II and III were aware of an increased risk of infections (I: 67.7%, II: 83.8%*, III: 89.9%*, P < 0.05). Pneumococcal vaccination rate was significantly higher (I: 20.2%, II 36.8%* and III: 39.0%*, P < 0.05) compared with cohort I. Differences were less evident for influenza. Significantly more patients >=60 years of age have been vaccinated against Streptococcus pneumoniae and influenza. An obvious discrepancy existed between vaccination awareness and actual vaccination rates for all cohorts. No significant differences in vaccination titers could be seen between the three cohorts. Awareness of infectious complications was more present in patients treated with biologicals, and also, the rate of patients vaccinated against Streptococcus pneumoniae increased significantly depending on the underlying treatment. Nevertheless, there was a discrepancy between vaccination awareness and actual vaccination rates for all cohorts.

Publication Type

Journal Article. Research Support, Non-U.S. Gov't.

Year of Publication

2012

<518>

Unique Identifier

22243559

Title

Chronic widespread pain and fibromyalgia: could there be some relationships with infections and vaccinations?. [Review]

Source

Clinical & Experimental Rheumatology. 29(6 Suppl 69):S118-26, 2011 Nov-Dec.

VI 1

Status

MEDLINE

Authors

Cassisi G; Sarzi-Puttini P; Cazzola M.

Authors Full Name

Cassisi, G; Sarzi-Puttini, P; Cazzola, M.

Institution

Cassisi, G. Rheumatology Branch, Specialist Outpatient Department, Belluno, Italy. cassisi.agordo@libero.it

Abstract

Chronic widespread pain (CWP) is a common symptom within the community, and may be part of or arise as a result of various diseases or conditions. Fibromyalgia (FM) is probably the most common and best known disease whose cardinal symptom is CWP. Many authors, however, indistinctively describe pain as 'widespread', 'diffuse' or 'generalised', and this may lead to misunderstandings about true clinical or scientific significance. Widespread pain has been variously defined, over the years, beginning from the American College of Rheumatology (ACR) classification criteria for FM in 1990, and the CWP Manchester definition in 1996. A comprehensive and brief core sets for CWP was developed in 2003, by the WHO International Classification of Functioning Consensus Conference, and finally, the ACR proposed new preliminary diagnostic criteria for FM in 2010. Research into CWP and/or FM is therefore difficult and can lead to conflicting results. CWP and (particularly) FM are multifactorial disorders. There is increasing evidence that they may be triggered by environmental factors, and many authors have highlighted a relationship with various infectious agents and some have suggested that vaccinations may play a role. This review analyses the available data concerning the relationships between FM and widespread pain (in its various meanings) with infections and vaccinations, from the earliest report to the most recent contributions. Considering all scientific papers, various levels of possible associations emerge. There is no clear-cut evidence of FM or CWP due to infections or vaccinations, no correlations with persistent infection, and no proven relationship between infection, antimicrobial therapies and pain improvement. A higher prevalence of FM and chronic pain has been found in patients with Lyme disease, and HIV or HCV infection, and, perhaps, also in patients with mycoplasmas, HBV, HTLV I, and parvovirus B19 infections. Some unconfirmed evidence and case reports suggest that vaccinations may trigger FM or chronic pain.

Publication Type

Journal Article. Review.

Year of Publication

2011

<519>

Unique Identifier

22127692

Title

Blood memory B cells are disturbed and predict the response to rituximab in patients with rheumatoid arthritis.

Source

Arthritis & Rheumatism. 63(12):3692-701, 2011 Dec.

VI 1

Status

MEDLINE

Authors

Sellam J; Rouanet S; Hendel-Chavez H; Abbed K; Sibilia J; Tebib J; Le Loet X; Combe B; Dougados M; Mariette X; Taoufik Y.

Authors Full Name

Sellam, Jeremie; Rouanet, Stephanie; Hendel-Chavez, Houria; Abbed, Karim; Sibilia, Jean; Tebib, Jacques; Le Loet, Xavier; Combe, Bernard; Dougados, Maxime; Mariette, Xavier; Taoufik, Yassine.

Institution

Sellam, Jeremie. Hopital Bicetre, AP-HP, INSERM U1012, and Universite Paris-Sud 11, Le Kremlin Bicetre, France. jeremie.sellam@sat.aphp.fr

Abstract

OBJECTIVE: To examine blood B cell subsets in patients with rheumatoid arthritis (RA) prior to B cell depletion therapy and to assess their potential as predictors of clinical response to rituximab (RTX).

METHODS: Blood B cell subsets were assessed by flow cytometry in 208 RA patients included in an RTX retreatment study (assessed prior to RTX treatment) and in 47 age-matched controls. Expression of BAFF receptor (BAFF-R) on B cells and serum B cell biomarkers was also measured. B cell subsets and BAFF-R expression were compared between RA patient and control populations. Univariate and multivariate analyses were performed to identify baseline

factors associated with a European League Against Rheumatism response 24 weeks after 1 cycle of RTX.

RESULTS: Mean +/- SD counts of both CD27- naive and CD27+ memory B cells were decreased in RA patients (188.6 +/- 121.4/mm(3)) compared with controls (257.3 +/- 154.1/mm(3)) (P = 0.001) and were partially restored in patients treated with methotrexate (MTX) plus anti-tumor necrosis factor compared with patients treated with MTX alone. Within the CD27+ memory B cells, the CD27+IgD- switched memory subtype was selectively decreased, irrespective of treatment. The frequency of CD27+ memory B cells correlated inversely with levels of several B cell activation biomarkers in RA. Serum BAFF level and BAFF-R expression was comparable in RA patients and controls. A low baseline CD27+ memory B cell frequency was associated with a greater clinical response to RTX (odds ratio 0.97 [95% confidence interval 0.95-0.99], P = 0.0015).

CONCLUSION: In B cell depletion therapy-naive RA patients, a low frequency of CD27+ memory B cells correlated with levels of serum B cell activation biomarkers and may predict response to RTX. These results suggest that low memory B cell frequency may be indicative of a B cell-driven RA subtype that is more sensitive to B cell depletion therapy. Copyright © 2011 by the American College of Rheumatology.

Publication Type

Journal Article. Randomized Controlled Trial. Research Support, Non-U.S. Gov't.

Year of Publication

2011

<520>

Unique Identifier

22106908

Title

Clinical features and prognosis of Boston type I keratoprosthesis-associated corneal melt.

Source

Ocular Immunology & Inflammation. 19(6):413-8, 2011 Dec.

VI 1

Status

MEDLINE

Authors

Utine CA; Tzu JH; Akpek EK.

Authors Full Name

Utine, Canan Asli; Tzu, Jonathan H; Akpek, Esen K.

Institution

Utine, Canan Asli. Ocular Surface Disease and Dry Eye Clinic, Cornea and External Disease Service, The Wilmer Eye Institute, The Johns Hopkins University School of Medicine, Baltimore, Maryland 21287-9238, USA.

Abstract

PURPOSE: To describe the clinical features and outcomes of corneal melt associated with Boston type I keratoprosthesis (KPro) implantation.

METHODS: Medical records of patients who experienced corneal melt following KPro implantation were reviewed retrospectively.

RESULTS: Sixty-six adult patients had KPro implantation from January 2004 to November 2010. Six patients had an underlying inflammatory ocular surface disorder. Four experienced corneal melt (6.1%) 5-42 months after the initial surgery. One patient was diagnosed with Sjogren's syndrome as a result of diagnostic workup following melt. Three patients were treated with systemic immunomodulatory therapy; two experienced fungal keratitis and subsequent endophthalmitis. KPro had to be explanted and replaced with donor cornea in all cases.

CONCLUSIONS: KPro-associated corneal melt is uncommon and appears to occur in patients with preexisting inflammatory disorders, which might not have been previously diagnosed. Timely explantation of KPro and replacement with donor cornea may prevent a poor outcome.

Publication Type

Journal Article.

Year of Publication

2011

<521>

Unique Identifier

22078703

Title

[Vaccines and chemo-prophylaxis in rhemautoid arthritis: is a vaccine calendar necessary?]. [Review] [Spanish]

Source

Reumatologia Clinica. 7(6):412-6, 2011 Nov-Dec.

VI 1

Status

MEDLINE

Authors

Garrido Lopez BC; Navarro Compain MV; Navarro Sarabia F.

Authors Full Name

Garrido Lopez, Belen Carmen; Navarro Compain, Maria Victoria; Navarro Sarabia, Federico.

Institution

Garrido Lopez, Belen Carmen. Servicio de Reumatologia, Hospital Universitario Virgen Macarena, Sevilla, Espana. belencgl@hotmail.com

Comments

Comment in (CIN)

Abstract

Patients with rheumatoid arthritis (RA) are at increased risk of infection compared to healthy individuals. The increased risk may be associated with the underlying disease, comorbidities and immunosuppressive therapy required to control RA activity. In several recent studies, influenza, pneumococcal and hepatitis B vaccines administered to RA patients were reported to be safe and serologically effective. However, several lines of evidence suggest a possible aberrant immunologic response following vaccination due to the compromised immunity of these patients. Therefore, vaccination of RA patients prior to immunosuppressive treatment may serve as an alternative prophylactic approach and should be considered for future investigation. Besides, prophylactic health measures should be taken to avoid latent chronic infections as tuberculosis and hepatitis B, during therapy with biological agents. Copyright © 2010 Elsevier Espana, S.L. All rights reserved.

Publication Type

English Abstract. Journal Article. Review.

Year of Publication

2011

<522>

Unique Identifier

22078700

Title

[Meningeal and Guillain-Barre syndrome in a patient with rheumatoid arthritis receiving adalimumab therapy]. [Spanish]

Source

Reumatologia Clinica. 7(6):401-3, 2011 Nov-Dec.

VI 1

Status

MEDLINE

Authors

Lopez Mendez P; Martin Santana I; del Pino Reyes Yanez M; Ruano Hernandez A; Hernandez Beriain JA; Hervas Garcia M.

Authors Full Name

Lopez Mendez, Pino; Martin Santana, Idaira; del Pino Reyes Yanez, Maria; Ruano Hernandez, Arminda; Hernandez Beriain, Jose Angel; Hervas Garcia, Miguel.

Institution

Lopez Mendez, Pino. Servicio de Neurologia, Hospital Universitario Insular de Gran Canaria, Las Palmas de Gran Canaria, Espana. plm_mf@hotmail.com

Abstract

Adalimumab is a recombinant human monoclonal antibody that blocks the effects of tumor necrosis factor-alpha, and is presently used for treatment of rheumatoid arthritis, with demyelination being a potential adverse effect. A 31 year-old male with seropositive rheumatoid arthritis presented with diarrhea after the second injection of adalimumab. He was treated with ciprofloxacin. In a few days he developed a Guillain-Barre syndrome confirmed by electromyography, and his cerebrospinal fluid was compatible with meningeal syndrome or partially treated bacterial meningitis. Adalimumab may be associated with the development of demyelination and infectious diseases. Moreover, both the central nervous system and the peripheral nervous system can be affected. Copyright © 2010 Elsevier Espana, S.L. All rights reserved.

Publication Type

Case Reports. English Abstract. Journal Article.

Year of Publication

2011

<523>
Unique Identifier
22029963
Title
MTRX1011A, a humanized anti-CD4 monoclonal antibody, in the treatment of patients with rheumatoid arthritis: a phase I randomized, double-blind, placebo-controlled study incorporating pharmacodynamic biomarker assessments.
Source
Arthritis Research & Therapy. 13(5):R177, 2011.
VI 1
Status
MEDLINE
Authors
Scheerens H; Su Z; Irving B; Townsend MJ; Zheng Y; Stefanich E; Chindalore V; Bingham CO 3rd; Davis JC Jr.
Authors Full Name
Scheerens, Heleen; Su, Zheng; Irving, Bryan; Townsend, Michael J; Zheng, Yanan; Stefanich, Eric; Chindalore, Vishala; Bingham, Clifton O 3rd; Davis, John C Jr.
Institution
Scheerens, Heleen. Genentech Research and Early Development, 1 DNA Way, South San Francisco, CA 94080, USA.
Abstract
INTRODUCTION: The purpose of this study was to evaluate the safety, tolerability, pharmacokinetics (PK) and pharmacodynamics (PD) of the humanized anti-CD4 monoclonal antibody MTRX1011A in a randomized, double-blind placebo-controlled Phase 1 study in patients with rheumatoid arthritis (RA).

METHODS: In the single ascending dose (SAD) portion of the study, patients received single doses of a placebo or MTRX1011A at 0.3, 1.0, 3.5 and 7.0 mg/kg intravenously (i.v.) or 1.0 and 3.5 mg/kg subcutaneously (s.c.), followed by five weeks of evaluation. In the multi-dose (MD) portion of the study, placebo or MTRX1011A was administered weekly for eight doses at 1.5 or 3.5 mg/kg s.c., or 5 mg/kg i.v., followed by eight weeks of evaluation.

RESULTS: MTRX1011A was well tolerated in the SAD phase up to 7 mg/kg i.v. and in the MD phase up to 1.5 mg/kg s.c.. At weekly doses of 3.5 mg/kg s.c. and 5 mg/kg i.v., a moderate pruritic papular rash was observed in some MTRX1011A-treated patients, which was considered a dose-limiting toxicity for this clinical indication. No serious adverse events occurred in any cohort. Reduction in disease activity was modest. PD assessments demonstrated that MTRX1011A induced a dose-dependent down-modulation of CD4 expression on peripheral blood CD4 T cells, CD4 receptor occupancy, increases in serum sCD4-MTRX1011A complexes and up-regulation of CD69 on T cells, but was non-depleting.

CONCLUSIONS: The maximum tolerated dose of MTRX1011A was 1.5 mg/kg SC administered weekly. At this dose MTRX1011A did not achieve maximum PD activity expected to be required for reduction in disease activity.

Publication Type

Clinical Trial, Phase I. Journal Article. Multicenter Study. Randomized Controlled Trial. Research Support, Non-U.S. Gov't.

Year of Publication

2011

<524>

Unique Identifier

21948985

Title

B cell depletion enhances T regulatory cell activity essential in the suppression of arthritis.

Source

Journal of Immunology. 187(9):4900-6, 2011 Nov 01.

VI 1

Status

MEDLINE

Authors

Hamel KM; Cao Y; Ashaye S; Wang Y; Dunn R; Kehry MR; Glant TT; Finnegan A.

Authors Full Name

Hamel, Keith M; Cao, Yanxia; Ashaye, Susan; Wang, Yumei; Dunn, Robert; Kehry, Marilyn R; Glant, Tibor T; Finnegan, Alison.
Institution
Hamel, Keith M. Department of Immunology/Microbiology, Rush University Medical Center, Chicago, IL 60612, USA.
Comments
Comment in (CIN)
Abstract
The efficacy of B cell-depletion therapy in rheumatoid arthritis has driven interest in understanding the mechanism. Because the decrease in autoantibodies in rheumatoid arthritis does not necessarily correlate with clinical outcome, other mechanisms may be operative. We previously reported that in proteoglycan-induced arthritis (PGIA), B cell-depletion inhibits autoreactive T cell responses. Recent studies in B cell-depletion therapy also indicate a role for B cells in suppressing regulatory mechanisms. In this study, we demonstrate that B cells inhibited both the expansion and function of T regulatory (Treg) cells in PGIA. Using an anti-CD20 mAb, we depleted B cells from mice with PGIA and assessed the Treg cell population. Compared to control Ab-treated mice, Treg cell percentages were elevated in B cell-depleted mice, with a higher proportion of CD4(+) T cells expressing Foxp3 and CD25. On a per-cell basis, CD4(+)CD25(+) cells from B cell-depleted mice expressed increased amounts of Foxp3 and were significantly more suppressive than those from control Ab-treated mice. The depletion of Treg cells with an anti-CD25 mAb concurrent with B cell-depletion therapy restored the severity of PGIA to levels equal to untreated mice. Although titers of autoantibodies did not recover to untreated levels, CD4(+) T cell recall responses to the immunizing Ag returned as measured by T cell proliferation and cytokine production. Thus, B cells have the capacity to regulate inflammatory responses by enhancing effector T cells along with suppressing Treg cells.
Publication Type
Journal Article. Research Support, N.I.H., Extramural.
Year of Publication
2011

<525>
Unique Identifier
21907543

Title
Immune-mediated adverse effects of biologicals used in the treatment of rheumatic diseases. [Review]

Source
Journal of Autoimmunity. 37(4):273-88, 2011 Dec.
VI 1
Status
MEDLINE

Authors
Borchers AT; Leibushor N; Cheema GS; Naguwa SM; Gershwin ME.
Authors Full Name
Borchers, Andrea T; Leibushor, Naama; Cheema, Gurtej S; Naguwa, Stanley M; Gershwin, M Eric.
Institution
Borchers, Andrea T. Division of Rheumatology, Allergy and Clinical Immunology, University of California at Davis School of Medicine, Davis, CA 95616, USA.

Abstract
Biological agents represent a major advance in the treatment of rheumatic diseases, most particularly in the prevention of irreversible structural damage. While generally well tolerated, their increasing use continues to reveal a variety of immune-mediated adverse effects. The most frequent adverse events are infusion reactions and injection site reactions, but despite their fairly common occurrence the precise mechanisms are not fully understood. Another adverse event that became appreciated early in the era of biologicals is the increased risk of Mycobacterium tuberculosis and other granulomatous infections in patients treated with tumor necrosis factor (TNFalpha) antagonists. Although it is evident that this enhanced susceptibility to intracellular infections must be due to immunosuppression arising from the blockade of TNFalpha, the mechanisms have not been fully elucidated; such an understanding is likely to provide important insights into the role of TNFalpha in granulomatous and other infectious diseases. In addition, the biologicals may paradoxically induce autoimmunity. The development of autoantibodies is seen in a considerable proportion of patients, but clinical autoimmune disease develops much less commonly, including systemic lupus erythematosus, multiple sclerosis and other demyelinating diseases, psoriasis, sarcoidosis, and interstitial lung disease. The mechanisms leading to their induction are very poorly understood, but an intriguing hypothesis is that interferon alpha provides a common link, at least for lupus, psoriasis and possibly sarcoidosis. Finally, the potential risk of infection with use of the biologicals is an issue that clinicians should always be aware of. These comments aside, the biologics are the most important advance in the treatment of rheumatic disease in the history of rheumatology and their usage has not only greatly helped patient care,

but also provided key data on the immunobiology of the disease processes. Copyright 2011 Elsevier Ltd. All rights reserved.

Publication Type

Journal Article. Review.

Year of Publication

2011

<526>

Unique Identifier

21896342

Title

Vaccination in paediatric patients with auto-immune rheumatic diseases: a systemic literature review for the European League against Rheumatism evidence-based recommendations. [Review]

Source

Autoimmunity Reviews. 11(2):112-22, 2011 Dec.

VI 1

Status

MEDLINE

Authors

Heijstek MW; Ott de Bruin LM; Borrow R; van der Klis F; Kone-Paut I; Fasth A; Minden K; Ravelli A; Abinun M; Pileggi G; Borte M; Bijl M; Wulffraat NM.

Authors Full Name

Heijstek, M W; Ott de Bruin, L M; Borrow, R; van der Klis, F; Kone-Paut, I; Fasth, A; Minden, K; Ravelli, A; Abinun, M; Pileggi, G; Borte, M; Bijl, M; Wulffraat, N M.

Institution

Heijstek, M W. Department of Paediatric Immunology, Wilhelmina Children's Hospital, University Medical Center Utrecht, The Netherlands. m.w.heijstek@umcutrecht.nl

Abstract

OBJECTIVES: To analyze available evidence on vaccinations in paediatric patients with rheumatic and autoinflammatory diseases. This evidence formed the basis of the recently constructed European League against Rheumatism (EULAR) recommendations for vaccination of these patients.

METHODS: A systematic literature review in the MEDLINE and EMBASE databases was conducted using various terms for vaccinations, paediatric rheumatic and autoinflammatory diseases and immunosuppressive drugs. Only papers on paediatric patients (<18 years of age) were selected. A panel of 13 experts in the field graded methodological quality and extracted data using predefined criteria.

RESULTS: 27 papers were available. No studies were found on autoinflammatory diseases. 14 studies considered live-attenuated vaccines. Evidence so far supports the safety and immunogenicity of non-live composite vaccines, although studies were underpowered to accurately assess safety. Live-attenuated vaccines did not cause disease flares or severe adverse events, not even in patients on methotrexate and low dose glucocorticosteroids. Seven patients on anti-TNFalpha therapy were described receiving the live-attenuated measles, mumps, rubella (n=5) or varicella (n=2) booster without severe adverse events.

CONCLUSIONS: Data on safety and efficacy of vaccinations in paediatric patients with rheumatic diseases is reassuring, but too limited to draw definite conclusions. More research is needed on the safety and efficacy of especially live-attenuated vaccines in patients with rheumatic and autoinflammatory diseases using high dose immunosuppressive drugs. Copyright © 2011 Elsevier B.V. All rights reserved.

Publication Type

Journal Article. Research Support, Non-U.S. Gov't. Review. Systematic Review.

Year of Publication

2011

<527>

Unique Identifier

21884580

Title

Inhibitory effects of the JAK inhibitor CP690,550 on human CD4(+) T lymphocyte cytokine production.

Source

BMC Immunology. 12:51, 2011 Aug 31.

VI 1

Status

MEDLINE

Authors

Migita K; Miyashita T; Izumi Y; Koga T; Komori A; Maeda Y; Jiuchi Y; Aiba Y; Yamasaki S; Kawakami A; Nakamura M; Ishibashi H.

Authors Full Name

Migita, Kiyoshi; Miyashita, Taiichiro; Izumi, Yasumori; Koga, Tomohiro; Komori, Atsumasa; Maeda, Yumi; Jiuchi, Yuka; Aiba, Yoshihiro; Yamasaki, Satoshi; Kawakami, Atsushi; Nakamura, Minoru; Ishibashi, Hiromi.

Institution

Migita, Kiyoshi. Department of Rheumatology and Clinical Research Center, NHO Nagasaki Medical Center, Kubara 2-1001-1, Omura 856-8652, Japan. migita@nmc.hosp.go.jp

Abstract

BACKGROUND: The new JAK3 inhibitor, CP690,550, has shown efficacy in the treatment of rheumatoid arthritis. The present study was undertaken to assess the effects of CP690,550 on cytokine production and cellular signaling in human CD4(+) T cells.

RESULTS: CD4(+) T cells produced IL-2, IL-4, IL-17, IL-22 and IFN-gamma in following stimulation with a CD3 antibody. At the optimal concentration, CP690,550 almost completely inhibited the production of IL-4, IL-17, IL-22 and IFN-gamma from these activated CD4(+) T cells, but only had marginal effects on IL-2 production. Moreover CP690,550 inhibited anti-CD3-induced phosphorylation of STAT1, STAT3, STAT4, STAT5, and STAT6, but not the TCR-associated phosphorylation of ZAP-70.

CONCLUSIONS: Therefore, CP690,550-mediated modification of the JAK/STAT pathway may be a new immunosuppressive strategy in the treatment of autoimmune diseases.

Publication Type

Journal Article.

Year of Publication

2011

<528>

Unique Identifier

21880506

Title

Therapeutic potential of Tregs to treat rheumatoid arthritis. [Review]

Source

Seminars in Immunology. 23(3):195-201, 2011 Jun.

VI 1

Status

MEDLINE

Authors

Wright GP; Stauss HJ; Ehrenstein MR.

Authors Full Name

Wright, Graham P; Stauss, Hans J; Ehrenstein, Michael R.

Institution

Wright, Graham P. Centre for Rheumatology Research, University College London, Rayne Building, 5 University Street, London WC1E 6JF, United Kingdom. graham.wright@ucl.ac.uk

Abstract

There is accumulating evidence for regulatory T cell defects in rheumatoid arthritis and that some biologic interventions, in particular anti-TNF, can target this population. Despite the challenges in defining regulatory T cells in patients, there are a number of approaches currently being developed to utilise their potent immunosuppressive properties. Through genetic manipulation Tregs can be generated ex vivo or in vivo that target antigens present in the inflamed joint. Here we discuss these approaches, their refinement to restore tolerance in patients with rheumatoid arthritis, and strategies to prevent their conversion towards a Th17 phenotype. Copyright © 2011 Elsevier Ltd. All rights reserved.

Publication Type

Journal Article. Research Support, Non-U.S. Gov't. Review.

Year of Publication

2011

<529>

Unique Identifier

21859687

Title

Vaccination leads to an aberrant FOXP3 T-cell response in non-remitting juvenile idiopathic arthritis.

Source

Annals of the Rheumatic Diseases. 70(11):2037-43, 2011 Nov.

VI 1

Status

MEDLINE

Authors

Ronaghy A; de Jager W; Zonneveld-Huijssoon E; Klein MR; van Wijk F; Rijkers GT; Kuis W; Wulffraat NM; Prakken BJ.

Authors Full Name

Ronaghy, Arash; de Jager, Wilco; Zonneveld-Huijssoon, Evelien; Klein, Mark R; van Wijk, Femke; Rijkers, Ger T; Kuis, Wietse; Wulffraat, Nico M; Prakken, Berent J.

Institution

Ronaghy, Arash. Department of Pediatrics, Center for Cellular and Molecular Intervention, Wilhelmina Children's Hospital, University Medical Center Utrecht, Utrecht, The Netherlands.

Abstract

OBJECTIVE: To investigate how meningococcal C vaccination in patients with remitting (oligoarticular) or progressive (polyarticular) juvenile idiopathic arthritis (JIA) influences the specific T-cell response to both the vaccine and heat shock protein 60, a regulatory auto-antigen in JIA.

METHODS: Twenty six oligoarticular, 28 polyarticular JIA patients and 20 healthy adults were studied before and after MenC vaccination in a prospective follow-up study. T-cell proliferation assay, flow cytometry, carboxyfluorescein diacetate succinimidyl ester staining and multiplex immunoassay were performed to quantify and qualify the antigen-specific immune responses.

RESULTS: Peripheral blood mononuclear cells (PBMC) from polyarticular JIA exemplified higher antigen-specific CD4 T-cell proliferation, interleukin 2 (IL-2) and tumour necrosis factor alpha (TNFalpha) production when compared with oligoarticular JIA or healthy individuals after vaccination. Furthermore, in polyarticular JIA antigen-induced CD4+CD25(bright) or CD4+FOXP3+ T cells did not increase upon vaccination.

CONCLUSION: Polyarticular JIA CD4+FOXP3+ T cells did not respond to vaccination and demonstrated a higher percentage of cells irrespective of vaccination when compared with oligoarticular JIA. These cells are either activated T cells and/or regulatory cells unable to

regulate the antigen-specific immune response after vaccination. When compared with oligoarticular JIA, the increased IL-2 and TNFalpha production underline the immune hyperresponsiveness of polyarticular JIA PBMC to an antigenic trigger. As this may hold a risk for derailment, these findings could provide a cellular basis for the presumed relationship between environmental triggers and disease in human autoimmune diseases.

Publication Type

Journal Article. Research Support, Non-U.S. Gov't.

Year of Publication

2011

<530>

Unique Identifier

21858123

Title

Impact of Schistosoma japonicum infection on collagen-induced arthritis in DBA/1 mice: a murine model of human rheumatoid arthritis.

Source

PLoS ONE [Electronic Resource]. 6(8):e23453, 2011.

VI 1

Status

MEDLINE

Authors

Song X; Shen J; Wen H; Zhong Z; Luo Q; Chu D; Qi Y; Xu Y; Wei W.

Authors Full Name

Song, Xiaorong; Shen, Jilong; Wen, Huiqin; Zhong, Zhengrong; Luo, Qinli; Chu, Deyong; Qi, Yao; Xu, Yuanhong; Wei, Wei.

Institution

Song, Xiaorong. Institute of Clinical Pharmacology, Anhui Medical University, Hefei, Anhui, China.

Abstract

BACKGROUND: The hygiene hypothesis suggests that helminth infections prevent a range of autoimmune diseases.

METHODOLOGY/PRINCIPAL FINDINGS: To investigate the effects of S. japonicum infection on collagen-induced arthritis (CIA), male DBA/1 mice were challenged with unisexual or bisexual S. japonicum cercariae two weeks prior to bovine type II collagen (CII) immunization or at the onset of CIA. S. japonicum infection prior to CII immunization significantly reduced the severity of CIA. ELISA (enzyme linked immunosorbent assay) showed that the levels of anti-CII IgG and IgG2a were reduced in prior schistosome-infected mice, while anti-CII IgG1 was elevated. Splenocyte proliferation against both polyclonal and antigen-specific stimuli was reduced by prior schistosome infection as measured by tritiated thymidine incorporation ((3)H-TdR). Cytokine profiles and CD4(+) T cells subpopulation analysis by ELISA and flow cytometry (FCM) demonstrated that prior schistosome infection resulted in a significant down-regulation of pro-inflammatory cytokines (IFN-gamma, TNF-alpha, IL-1beta and IL-6) and Th1 cells, together with up-regulation of the anti-inflammatory cytokine IL-10 and Th2 cells. Interestingly, the expansion of Treg cells and the reduction of Th17 cells were only observed in bisexually infected mice. In addition, prior schistosome infection notably reduced the expression of pro-inflammatory cytokines and receptor activator of NF-kappaB ligand (RANKL) in the inflamed joint. However, the disease was exacerbated at one week after infection when established CIA mice were challenged with bisexual cercariae.

CONCLUSION/SIGNIFICANCE: Our data provide direct evidence that the Th2 response evoked by prior S. japonicum infection can suppress the Th1 response and pro-inflammatory mediator and that bisexual infection with egg-laying up-regulates the Treg response and down-regulates the Th17 response, resulting in an amelioration of autoimmune arthritis. The beneficial effects might depend on the establishment of a Th2-dominant response rather than the presence of the eggs. Our results suggest that anti-inflammatory molecules from the parasite could treat autoimmune diseases.

Publication Type

Journal Article. Research Support, Non-U.S. Gov't.

Year of Publication

2011

<531>

Unique Identifier

21843497

Title

Desiccating stress induces CD4+ T-cell-mediated Sjogren's syndrome-like corneal epithelial apoptosis via activation of the extrinsic apoptotic pathway by interferon-gamma.

Source

American Journal of Pathology. 179(4):1807-14, 2011 Oct.

VI 1

Status

MEDLINE

Authors

Zhang X; Chen W; De Paiva CS; Volpe EA; Gandhi NB; Farley WJ; Li DQ; Niederkorn JY; Stern ME; Pflugfelder SC.

Authors Full Name

Zhang, Xiaobo; Chen, Wei; De Paiva, Cintia S; Volpe, Eugene A; Gandhi, Niral B; Farley, William J; Li, De-Quan; Niederkorn, Jerry Y; Stern, Michael E; Pflugfelder, Stephen C.

Institution

Zhang, Xiaobo. Ocular Surface Center, Cullen Eye Institute, and the Department of Ophthalmology, Baylor College of Medicine, Houston, TX 77030, USA.

Abstract

We investigated the role of CD4(+) T-cell-produced interferon (IFN)-gamma on corneal epithelial apoptosis in a murine desiccating stress (DS) model that resembles Sjogren's syndrome. The DS model was generated in C57BL/6 (B6) and B6 IFN-gamma-knockout (B6gammaKO) mice. Adoptive transfer of CD4(+) T cells from DS-exposed donor to recombination activating gene (RAG)-1(-/-) recipient mice and topical neutralization of IFN-gamma were performed to determine whether IFN-gamma produced by pathogenic CD4(+) T cells promotes corneal epithelial apoptosis. Apoptosis in corneal epithelia was assessed by evaluating the expression and activity of caspases 3, 8, and 9. The activation of caspase-8 mediated increased corneal epithelial apoptosis in B6 mice after DS, and this was exacerbated by subconjunctival IFN-gamma injection. B6gammaKO mice were resistant to DS-induced apoptosis; however, B6gammaKO mice receiving IFN-gamma developed apoptosis similar to that observed in B6 wild-type mice. Adoptive transfer of CD4(+) T cells from donors subjected to DS increased corneal epithelial apoptosis via activation of caspase-8 in recipients, similar to that in the donor mice. Topical neutralization of IFN-gamma in adoptive transfer recipients decreased corneal epithelial apoptosis. DS, IFN-gamma administration, or CD4(+) T-cell adoptive transfer had no effect on the expression and activation of the intrinsic apoptosis mediator, caspase-9. CD4(+) T-cell-produced IFN-gamma plays a pivotal role in DS-induced corneal epithelial apoptosis via activation of the extrinsic apoptotic pathway. Copyright © 2011 American Society for Investigative Pathology. Published by Elsevier Inc. All rights reserved.

Publication Type
  Journal Article. Research Support, N.I.H., Extramural. Research Support, Non-U.S. Gov't.
Year of Publication
  2011

<532>
Unique Identifier
  21841730
Title
  Prophylaxis of hepatitis B reactivation with immunosuppressive therapy in rheumatic diseases. Orientations for clinical practice. [Review]
Source
  Acta Reumatologica Portuguesa. 36(2):110-8, 2011 Apr-Jun.
VI 1
Status
  MEDLINE
Authors
  Nunes J; Marinho RT; Fonseca JE; Pereira da Silva JA; Velosa J.
Authors Full Name
  Nunes, Joana; Marinho, Rui Tato; Fonseca, J E; Pereira da Silva, Josa A; Velosa, Josa.
Institution
  Nunes, Joana. Department of Gastroenterology and Hepatology, Hospital Santa Maria, Lisbon, Portugal. joanamnunes@gmail.com
Abstract
  Reactivation of infection with hepatitis B virus (HBV) is a potentially serious complication of immunosuppression, which can be identified and efficiently prevented. There have been an increasing number of cases of HBV reactivation in patients receiving immunosuppression in the context of rheumatic diseases such as rheumatoid arthritis or systemic lupus erythematosus. The recommendations in this area should be individualized taking into account two aspects: immunosuppressive regimens used (high or low risk of reactivation) and the different stages of HBV infection: chronic hepatitis B, inactive HBV carrier, occult hepatitis B infection defined by HB surface antigen (HBsAg) negative and antibody anti-HB core (anti-HBc) positive. In patients with rheumatic diseases that will start high-risk immunosuppressive drugs, we propose a universal

screening with serological tests for hepatitis B (HBsAg, anti-HBs and anti-HBc). Patients with chronic hepatitis B (HBsAg positive, HBV DNA >= 2000 IU/ml, elevated ALT) should initiate antiviral therapy. Inactive HBV carriers (HBsAg positive, HBV DNA <2000 IU / ml, normal aminotransferases) exposed to high risk immunosuppressive therapy should undergo prophylaxis of HBV reactivation. Prophylaxis should be started 2 to 4 weeks before the beginning of immunosuppressive therapy and maintained for at least 6 to 12 months after its suspension. It is recommended to use entecavir or tenofovir as first line antiviral agents. In inactive HBsAg carriers under low-risk immunosuppressive therapy and patients with HBsAg negative/anti-HBc positive (HBV infection in the past), the strategy should be monitoring of viral reactivation with aminotransferases and HBV DNA determination in every 6 months.

Publication Type

Journal Article. Review.

Year of Publication

2011

<533>

Unique Identifier

21840621

Title

Anti-TNF therapy in patients with rheumatoid arthritis decreases Th1 and Th17 cell populations and expands IFN-gamma-producing NK cell and regulatory T cell subsets.

Source

Immunobiology. 216(12):1256-63, 2011 Dec.

VI 1

Status

MEDLINE

Authors

Aravena O; Pesce B; Soto L; Orrego N; Sabugo F; Wurmann P; Molina MC; Alfaro J; Cuchacovich M; Aguillon JC; Catalan D.

Authors Full Name

Aravena, Octavio; Pesce, Barbara; Soto, Lilian; Orrego, Natalia; Sabugo, Francisca; Wurmann, Pamela; Molina, Maria Carmen; Alfaro, Jorge; Cuchacovich, Miguel; Aguillon, Juan Carlos; Catalan, Diego.

Institution

Aravena, Octavio. Programa Disciplinario de Inmunologia, Instituto de Ciencias Biomedicas-ICBM, Facultad de Medicina, Universidad de Chile and Millennium Institute on Immunology and Immunotherapy P09-016-F, Av. Independencia #1027, Santiago, Chile.

Abstract

The aim of this work was to study the effect of anti-TNF treatment on CD4+ Th1, Th17 and regulatory T cells (Tregs), together with CD8+ T cells and NK cells from rheumatoid arthritis (RA) patients. For this purpose, 18 RA patients received adalimumab during 16weeks and their peripheral blood lymphocytes were assessed by flow cytometry at the beginning and at the end of the study. We found that the proportion of Th17 cells was directly correlated with Th1 cells, but inversely correlated with IFN-gamma-producing NK cells. A decrease was observed in Th1, Th17 cells and IFN-gamma-producing CD8+ T cells by anti-TNF therapy. Conversely, the proportion of Tregs increased, as did the percentage of IFN-gamma-producing NK cells. We postulate that a rise in IFN-gamma production due to recovery of NK cells' function, together with expanded Tregs, contribute to decrease the Th17 response in anti-TNF-treated RA patients. Copyright © 2011 Elsevier GmbH. All rights reserved.

Publication Type

Journal Article. Research Support, Non-U.S. Gov't.

Year of Publication

2011

<534>

Unique Identifier

21828127

Title

Functional human regulatory T cells fail to control autoimmune inflammation due to PKB/c-akt hyperactivation in effector cells.

Source

Blood. 118(13):3538-48, 2011 Sep 29.

VI 1

Status

MEDLINE

Authors

Wehrens EJ; Mijnheer G; Duurland CL; Klein M; Meerding J; van Loosdregt J; de Jager W; Sawitzki B; Coffer PJ; Vastert B; Prakken BJ; van Wijk F.
Authors Full Name
Wehrens, Ellen J; Mijnheer, Gerdien; Duurland, Chantal L; Klein, Mark; Meerding, Jenny; van Loosdregt, Jorg; de Jager, Wilco; Sawitzki, Birgit; Coffer, Paul J; Vastert, Bas; Prakken, Berent J; van Wijk, Femke.
Institution
Wehrens, Ellen J. Center for Molecular and Cellular Intervention, Department of Immunology, Wilhelmina Children's Hospital, University Medical Center Utrecht, Utrecht, The Netherlands. ewehrens@umcutrecht.nl
Abstract
During the last decade research has focused on the application of FOXP3(+) regulatory T cells (Tregs) in the treatment of autoimmune disease. However, thorough functional characterization of these cells in patients with chronic autoimmune disease, especially at the site of inflammation, is still missing. Here we studied Treg function in patients with juvenile idiopathic arthritis (JIA) and observed that Tregs from the peripheral blood as well as the inflamed joints are fully functional. Nevertheless, Treg-mediated suppression of cell proliferation and cytokine production by effector cells from the site of inflammation was severely impaired, because of resistance to suppression. This resistance to suppression was not caused by a memory phenotype of effector T cells or activation status of antigen presenting cells. Instead, activation of protein kinase B (PKB)/c-akt was enhanced in inflammatory effector cells, at least partially in response to TNFalpha and IL-6, and inhibition of this kinase restored responsiveness to suppression. We are the first to show that PKB/c-akt hyperactivation causes resistance of effector cells to suppression in human autoimmune disease. Furthermore, these findings suggest that for a Treg enhancing strategy to be successful in the treatment of autoimmune inflammation, resistance because of PKB/c-akt hyperactivation should be targeted as well.
Publication Type
Journal Article. Research Support, Non-U.S. Gov't.
Year of Publication
2011

<535>

Unique Identifier

21813547

Title

EULAR recommendations for vaccination in paediatric patients with rheumatic diseases.

Source

Annals of the Rheumatic Diseases. 70(10):1704-12, 2011 Oct.

VI 1

Status

MEDLINE

Authors

Heijstek MW; Ott de Bruin LM; Bijl M; Borrow R; van der Klis F; Kone-Paut I; Fasth A; Minden K; Ravelli A; Abinun M; Pileggi GS; Borte M; Wulffraat NM; EULAR.

Authors Full Name

Heijstek, M W; Ott de Bruin, L M; Bijl, M; Borrow, R; van der Klis, F; Kone-Paut, I; Fasth, A; Minden, K; Ravelli, A; Abinun, M; Pileggi, G S; Borte, M; Wulffraat, N M; EULAR.

Institution

Heijstek, M W. Department of Paediatric Immunology, Wilhelmina Children's Hospital, University Medical Centre Utrecht, Room KC 03.063.0, PO Box 85090, 3508 AB Utrecht, The Netherlands. m.w.heijstek@umcutrecht.nl

Abstract

Evidence-based recommendations for vaccination of paediatric patients with rheumatic diseases (PaedRD) were developed by following the EULAR standardised procedures for guideline development. The EULAR task force consisted of (paediatric) rheumatologists/immunologists, one expert in vaccine evaluation, one expert in public health and infectious disease control, and one epidemiologist. A systematic literature review was conducted in MEDLINE, EMBASE, and abstracts of the EULAR and American College of Rheumatology meetings of 2008/9. The level of evidence and strength of recommendation were based on customary scoring systems. Delphi voting was applied to assess the level of agreement between task force members. 107 papers and eight abstracts were used. The majority of papers considered seasonal influenza (41) or pneumococcal (23) vaccination. 26 studies were performed specifically in paediatric patients, and the majority in adult rheumatoid arthritis and systemic lupus erythematosus patients. Fifteen recommendations were developed with an overall agreement of 91.7%. More research is needed on the safety and immunogenicity of (live-attenuated) vaccination in PaedRD, particularly in those using biologicals, and the effect of vaccination on prevention of infections.

Publication Type

Journal Article. Practice Guideline. Research Support, Non-U.S. Gov't.

Year of Publication

2011

<536>

Unique Identifier

21810616

Title

Comment on "Interplay between TNF and regulatory T cells in a TNF-driven murine model of arthritis".

Source

Journal of Immunology. 187(4):1527; author reply 1527-8, 2011 Aug 15.

VI 1

Status

MEDLINE

Authors

Chen X; Oppenheim JJ.

Authors Full Name

Chen, Xin; Oppenheim, Joost J.

Comments

Comment on (CON)

Publication Type

Comment. Letter.

Year of Publication

2011

<537>

Unique Identifier

21804099

Title

Developing tolerogenic dendritic cell therapy for rheumatoid arthritis: what can we learn from mouse models?. [Review]

Source

Annals of the Rheumatic Diseases. 70(9):1526-33, 2011 Sep.

VI 1

Status

MEDLINE

Authors

Stoop JN; Robinson JH; Hilkens CM.

Authors Full Name

Stoop, Jeroen N; Robinson, John H; Hilkens, Catharien M U.

Institution

Stoop, Jeroen N. Institute of Cellular Medicine, Musculoskeletal Research Group, Newcastle University, Newcastle upon Tyne, UK.

Abstract

One of the therapeutic strategies under development for the treatment of rheumatoid arthritis is based on reinstating immune tolerance by vaccination with autologous dendritic cells with potent tolerogenic function. These tolerogenic dendritic cells (TolDC) can be generated ex vivo and have beneficial therapeutic effects in animal models of arthritis. Although experimental animal models have been instrumental in the development of this novel immunotherapeutic tool, several outstanding questions regarding the application of TolDC remain to be addressed. This paper reviews what has been learnt to date from studying the therapeutic potential of TolDC in animal models of arthritis and discusses issues relating to preventive versus curative effects of TolDC, the antigen specificity of TolDC therapy, the route, dose and frequency of TolDC administration and the safety of TolDC treatment. Lessons learnt from animal models will aid the design of clinical trials with TolDC.

Publication Type

Journal Article. Research Support, Non-U.S. Gov't. Review.

Year of Publication

2011

<538>

Unique Identifier

21794777

Title

[Consensus on the Use of Rituximab in Rheumatoid Arthritis. A document with evidence-based recommendations. Grupo de Expertos en Rituximab.]. [Spanish]

Source

Reumatologia Clinica. 7(1):30-44, 2011 Jan-Feb.

VI 1

Status

MEDLINE

Authors

Martin Mola E; Hernandez B; Garcia-Arias M; Alvaro-Gracia JM; Balsa A; Reino JG; Marenco de la Fuente JL; Martinez-Taboada V; Ivorra JA; Sanmarti R; el grupo de Expertos en Rituximab.

Authors Full Name

Martin Mola, Emilio; Hernandez, Blanca; Garcia-Arias, Miriam; Alvaro-Gracia, Jose Maria; Balsa, Alejandro; Reino, Juan Gomez; Marenco de la Fuente, Jose L; Martinez-Taboada, Victor; Ivorra, Jose Andres Roman; Sanmarti, Raimon; el grupo de Expertos en Rituximab.

Institution

Martin Mola, Emilio. Servicio de Reumatologia, Hospital Universitario La Paz, Madrid, Espana. emartinmola.hulp@salud.madrid.org

Abstract

INTRODUCTION: Rituximab has been employed successfully for the treatment of Rheumatoid Arthritis (RA). However, its particular mechanism of action, as well as a lack of concrete guidelines for its management have generated doubts on its use.

OBJECTIVE: To establish recommendations that facilitates the use of rituximab in common clinical practice.

METHODS: In a first Delphi round, 9 expert rheumatologists got together to develop questions on those subjects generating most doubts on the efficacy and safety of the drug. These were adapted to perform a systematic review of the evidence, which was presented in a second meeting. Nominal groups were formed to respond to each question and give a recommendation. These recommendations were presented in a second Delphi round to a larger group of experts in rheumatology. Once again recommendations were discussed, modified and voted upon. Once approved, a vote on the degree of agreement for each recommendation was carried out.

RESULTS: 17 recommendations were established, 10 regarding efficacy and 7 safety. All of the efficacy recommendations except 3 presented a good or moderate degree of evidence. Among the safety recommendations, 3 had a good or moderate degree of evidence while in the rest it

was indirect, scarce or non-existent and a product of expert recommendation. The degree of agreement between experts was elevated for most of the recommendations.

CONCLUSIONS: These recommendations attempt to clear doubts on the use of rituximab and establish guidelines for its use in daily practice. Efficacy recommendations have a high degree of evidence, allowing the clinician to be guided in therapeutic decisions. Safety recommendations have a lower degree of evidence. Copyright © 2010 Elsevier Espana, S.L. All rights reserved.

Publication Type

Consensus Development Conference. Journal Article. Practice Guideline. Research Support, Non-U.S. Gov't. Systematic Review.

Year of Publication

2011

<539>

Unique Identifier

21777703

Title

B-cells and their targeting in rheumatoid arthritis--current concepts and future perspectives. [Review]

Source

Autoimmunity Reviews. 11(1):28-34, 2011 Nov.

VI 1

Status

MEDLINE

Authors

Nakken B; Munthe LA; Konttinen YT; Sandberg AK; Szekanecz Z; Alex P; Szodoray P.

Authors Full Name

Nakken, Britt; Munthe, Ludvig A; Konttinen, Yrjo T; Sandberg, Anna Klokk; Szekanecz, Zoltan; Alex, Philip; Szodoray, Peter.

Institution

Nakken, Britt. Institute of Immunology, Rikshospitalet, Oslo University Hospital, Sognsvannsveien 20, Oslo, Norway.

Abstract

Rheumatoid arthritis (RA) is a chronic, autoimmune disease that affects primarily the joints and without proper treatment results in their progressive destruction. In addition to T-cells, B-cells play a central role in the pathogenesis of this disease. The synovial tissue is an active site of B-cell accumulation, plasma cell differentiation and in situ antibody-production in RA. As part of the complex role of B-cells in the joints and synovial membrane of RA patients, B cells secrete chemokines and cytokines and may function as antigen presenting cells. The multifaceted pathogenic function of B-cells identifies them as excellent targets for immunosuppressive therapy. B-cell targeting involves a wide spectrum of molecules, for example the B-cell antigen CD20 that allows specific and effective B-cell depletion. Another target, CD79, expressed by B-cell and plasma cell precursors is an obvious candidate that induces apoptosis as well as inhibition of B-cell receptor (BCR) activation and possibly depletion of ectopic germinal centers (GC). Inhibition of B-cell co-stimulatory molecules such as CD40, CD80/86 and ICOS, can lead to diminished B-cell activation. Moreover, anti-chemokine and anti-cytokine therapies can be efficacious in RA by the disruption of B-cell activation and autoantibody production, B-cell synovial migration and ectopic GC formation. Finally, targeting the signal transduction pathways required for proximal BCR signaling has also been found efficacious in early clinical trials in RA. Even so, some B cells inhibit immune responses, these regulatory B cells may play a part in immune regulation in patients and it is unclear what effects B cell depletion strategies have in terms of such B cell subsets. This review discusses current strategies of targeting B-cells as therapeutic candidates in the management of RA. Better insights into the pathogenic role of B-cells provide efficacious opportunities to improve both therapy and prognosis of patients with RA. Copyright A© 2011 Elsevier B.V. All rights reserved.

Publication Type

Journal Article. Review.

Year of Publication

2011

<540>

Unique Identifier

21763385

Title

Risk of rheumatoid arthritis following vaccination with tetanus, influenza and hepatitis B vaccines among persons 15-59 years of age.

Source
Vaccine. 29(38):6592-7, 2011 Sep 02.
VI 1
Status
MEDLINE
Authors
Ray P; Black S; Shinefield H; Dillon A; Carpenter D; Lewis E; Ross P; Chen RT; Klein NP; Baxter R; Vaccine Safety Datalink Team.
Authors Full Name
Ray, Paula; Black, Steven; Shinefield, Henry; Dillon, Aileen; Carpenter, Diane; Lewis, Edwin; Ross, Pat; Chen, Robert T; Klein, Nicola P; Baxter, Roger; Vaccine Safety Datalink Team.
Institution
Ray, Paula. Kaiser Permanente Vaccine Study Center, Oakland, CA, USA.
Abstract
BACKGROUND: Associations between vaccinations, particularly hepatitis B, and onset of rheumatoid arthritis (RA) have been reported, but examined in few large-scale studies.

METHOD: Onset of RA cases and dates of vaccination against hepatitis B, tetanus, and influenza were identified in a retrospective chart review of approximately 1 million Kaiser Permanente Northern California members ages 15-59 years from 1997 through 1999. In a cohort analysis, rates of new-onset RA were compared between vaccinated and unvaccinated within 90, 180, and 365 days. In a case-control analysis, rates of vaccination during exposure intervals (90, 180, 365, and 730 days) were compared between cases and controls using conditional logistic regression.

RESULTS: 378 RA cases were included in the cohort analysis; 37 additional cases were included in the case-control analysis. In the cohort analysis the relative risks of RA onset within 90, 180, or 365 days of hepatitis B vaccination were not significant (R.R.=1.44, p=0.53; R.R.=1.67, p=0.22; R.R.=1.23, p=0.59 respectively). We found a possible association between RA and influenza vaccine in the previous 180 and 365 days in the cohort analysis (R.R=1.36, p=0.03; R.R.=1.34, p=0.01 respectively), but in the case-control analysis, cases were no more likely than controls to have received any of the three vaccines.

CONCLUSIONS: In this large retrospective study we found no statistically significant association between exposure to hepatitis B vaccine and onset of RA. A possible association between RA and influenza vaccination in the cohort study was not borne out in the larger case-control analysis. Copyright © 2011 Elsevier Ltd. All rights reserved.

Publication Type
  Journal Article. Research Support, U.S. Gov't, P.H.S..
Year of Publication
  2011

<541>
Unique Identifier
  21744183
Title
  Active immunological profile is associated with systemic Sjogren's syndrome.
Source
  Journal of Clinical Immunology. 31(5):840-7, 2011 Oct.
VI 1
Status
  MEDLINE
Authors
  Martel C; Gondran G; Launay D; Lalloue F; Palat S; Lambert M; Ly K; Loustaud-Ratti V; Bezanahary H; Hachulla E; Jauberteau MO; Vidal E; Hatron PY; Fauchais AL.
Authors Full Name
  Martel, Clothilde; Gondran, Guillaume; Launay, David; Lalloue, Fabrice; Palat, Sylvain; Lambert, Marc; Ly, Kim; Loustaud-Ratti, Veronique; Bezanahary, Holly; Hachulla, Eric; Jauberteau, Marie Odile; Vidal, Elisabeth; Hatron, Pierre Yves; Fauchais, Anne Laure.
Institution
  Martel, Clothilde. Department of Internal Medicine, Limoges University Hospital, Limoges, France.
Abstract
  BACKGROUND: The aim of this paper was to study the evolution of primary Sjogren's syndrome (pSS) immunological profile, its impact on pSS activity and long-term evolution in a bicentric cohort of French patients with pSS (n = 445, mean age 53.6 +/- 14 years, mean follow-up 76.1 +/- 51 months).

   METHODS: This is a retrospective cohort study.

RESULTS: Two hundred twelve patients were Sjogren's syndrome A (SSA) positive, and 131 were both SSA and Sjogren's syndrome B (SSB) positive. Sixty-eight patients (15%) had cryoglobulinemia. Active systemic profile (i.e., hypergammaglobulinemia, rheumatoid factor (RF), and anti-Sjogren's syndrome A (anti-SSA), anti-Sjogren's syndrome B (anti-SSB) positivity), associated with multisystemic involvement, leads to an increased utilization of corticosteroid and hydroxychloroquine. Multivariate analysis pointed out independent statistical association between hypergammaglobulinemia, anti-SSA, anti-SSB, and RF. Cryoglobulinemia is associated with multi-systemic involvement, lymphoma, and pSS-related death.

CONCLUSION: The subset of patients with active immunological profile is characterized by systemic complications leading to immunosuppressive drug utilization and polyclonal B-cell activation profile.

Publication Type

Journal Article.

Year of Publication

2011

<542>

Unique Identifier

21732015

Title

TRAIL is associated with impaired regulation of CD4+CD25- T cells by regulatory T cells in patients with rheumatoid arthritis.

Source

Journal of Clinical Immunology. 31(6):1112-9, 2011 Dec.

VI 1

Status

MEDLINE

Authors

Xiao H; Wang S; Miao R; Kan W.

Authors Full Name

Xiao, Hong; Wang, Shun; Miao, Runsheng; Kan, Wusheng.

Institution

Xiao, Hong. Wuhan No.1 Hospital, No. 375 Zhongshan Avenue, Wuhan, 430000, China.
Abstract
Thirty-five rheumatoid arthritis (RA) patients and 27 healthy volunteers were enrolled in the study. Regulatory T (Treg) cell numbers were significantly reduced in RA patients. RA Treg cells exhibited an impaired capacity to inhibit proliferation and cytokine secretion of autologous T effector (Teff) cells. However, the crossover experiments further indicated that this impaired suppression was due to resistance of Teff cells but not to an intrinsic defect of Treg cells in RA patients. RA Teff cells showed a higher expression of membrane tumor necrosis factor-related apoptosis-inducing ligand and secreted more soluble tumor necrosis factor-related apoptosis-inducing ligand (TRAIL). TRAIL could induce apoptosis in Treg cells. Neutralization of TRAIL restored the regulation of Teff by Treg in RA patients. In summary, our data suggest that reduced peripheral Treg cell numbers and an increased resistance of Teff cells to suppression by Treg cells were present in RA patients, and TRAIL may be an underlying mechanism for the impaired regulation of Teff cells by Treg cells.
Publication Type
Journal Article. Research Support, Non-U.S. Gov't.
Year of Publication
2011

<543>
Unique Identifier
21732014
Title
KRN/I-Ag7 mouse arthritis is independent of complement C3.
Source
Journal of Clinical Immunology. 31(5):857-63, 2011 Oct.
VI 1
Status
MEDLINE
Authors
Tsao PY; Arora V; Ji MQ; Wright AC; Eisenberg RA.
Authors Full Name
Tsao, Patricia Y; Arora, Vaishali; Ji, Mei Qing; Wright, Alexander C; Eisenberg, Robert A.

Institution
Tsao, Patricia Y. Department of Medicine, Division of Rheumatology, University of Pennsylvania, Philadelphia, PA 19104-6160, USA.
Abstract
BACKGROUND: KRN/I-A(g7) (KxB/N) is a mouse model of inflammatory arthritis, which resembles human rheumatoid arthritis. Arthritis in these animals is caused by autoreactivity to a ubiquitously expressed autoantigen, glucose-6 phosphate isomerase. Tolerance is broken at both the T cell and B cell level. The sera from KRN/I-A(g7) mice can induce mouse arthritis in healthy mice. Complement components of the alternative complement pathway, including C3, have been shown to be required in induction of mouse arthritis by serum transfer.

METHODS: We have bred KRN/I-A(g7) mice onto a C3-deficient background and followed cohorts for the spontaneous appearance of arthritis. We have also transferred KxB/N serum to B6.I-A (g7) recipients.

RESULTS: C3-deficient KRN/I-A(g7) mice spontaneously developed severe, destructive arthritis, comparable to that seen in C3-intact KRN/I-A(g7) mice. However, serum transfer experiments confirmed the strong requirement for C3 in the passive model.

CONCLUSION: The pathogenesis of spontaneous KRN/I-A(g7) arthritis can largely proceed by complement-independent pathways and must have pathology effector mechanisms in addition to those seen in the passive serum transfer model.
Publication Type
Journal Article. Research Support, N.I.H., Extramural. Research Support, Non-U.S. Gov't.
Year of Publication
2011

<544>
Unique Identifier
21722505
Title
A cross-sectional audit of the uptake of seasonal and H1N1 influenza vaccination amongst patients with rheumatoid arthritis in a London hospital.

Source
  Clinical & Experimental Rheumatology. 29(3):596, 2011 May-Jun.
VI 1
Status
  MEDLINE
Authors
  Clarke AJ; Gulati P; Abraham SM.
Authors Full Name
  Clarke, A J; Gulati, P; Abraham, S M.
Publication Type
  Letter.
Year of Publication
  2011

<545>
Unique Identifier
  21721382
Title
  Successful rapid rituximab desensitization for hypersensitivity reactions to monoclonal antibodies in a patient with rheumatoid arthritis: a remarkable option.
Source
  Journal of Investigational Allergology & Clinical Immunology. 21(4):319-21, 2011.
VI 1
Status
  MEDLINE
Authors
  Abadoglu O; Epozturk K; Atayik E; Kaptanoglu E.
Authors Full Name
  Abadoglu, O; Epozturk, K; Atayik, E; Kaptanoglu, E.
Institution
  Abadoglu, O. Cumhuriyet University, Faculty of Medicine, Chest Diseases Dept., Immunology and Allergic Diseases Subdepartment, Sivas, Turkey. oaallerji@yahoo.com
Publication Type

Case Reports. Journal Article.
Year of Publication
2011

<546>
Unique Identifier
21704841
Title
Laser scanning cytometry: capturing the immune system in situ.
Source
Methods in Cell Biology. 102:231-60, 2011.
VI 1
Status
MEDLINE
Authors
McGrath MA; Morton AM; Harnett MM.
Authors Full Name
McGrath, Mairi A; Morton, Angela M; Harnett, Margaret M.
Institution
McGrath, Mairi A. Institute of Infection, Immunity and Inflammation, College of Medical Veterinary & Life Sciences, Glasgow Biomedical Research Centre, University of Glasgow, Scotland, UK.
Abstract
Until recently, it has not been possible to image and functionally correlate the key molecular and cellular events underpinning immunity and tolerance in the intact immune system. Certainly, the field has been revolutionized by the advent of tetramers to identify physiologically relevant specificities of T cells, and the introduction of models in which transgenic T-cell receptor and/or B-cell receptor-bearing lymphocytes are adoptively transferred into normal mice and can then be identified by clonotype-specific antibodies using flow cytometry in vitro, or immunohistochemistry ex vivo. However, these approaches do not allow for quantitative analysis of the precise anatomical, phenotypic, signaling, and functional parameters required for dissecting the development of immune responses in health and disease in vivo. Traditionally, assessment of signal transduction pathways has required biochemical or molecular biological analysis of isolated

and highly purified subsets of immune system cells. Inevitably, this creates potential artifacts and does not allow identification of the key signaling events for individual cells present in their microenvironment in situ. These difficulties have now been overcome by new methodologies in cell signaling analysis that are sufficiently sensitive to detect signaling events occurring in individual cells in situ and the development of technologies such as laser scanning cytometry that provide the tools to analyze physiologically relevant interactions between molecules and cells of the innate and the adaptive immune system within their natural environmental niche in vivo.
Copyright © 2011 Elsevier Inc. All rights reserved.
Publication Type
  Journal Article. Research Support, Non-U.S. Gov't.
Year of Publication
  2011

<547>
Unique Identifier
  21654650
Title
  Pfizer's JAK inhibitor sails through phase 3 in rheumatoid arthritis.
Source
  Nature Biotechnology. 29(6):467-8, 2011 Jun 07.
VI 1
Status
  MEDLINE
Authors
  Garber K.
Authors Full Name
  Garber, Ken.
Publication Type
  News.
Year of Publication
  2011

<548>

Unique Identifier

21649536

Title

Abatacept: a biologic immune modulator for rheumatoid arthritis.

Source

Expert Opinion on Biological Therapy. 11(8):1113-29, 2011 Aug.

VI 1

Status

MEDLINE

Authors

Papagoras C; Drosos AA.

Authors Full Name

Papagoras, Charalampos; Drosos, Alexandros A.

Institution

Papagoras, Charalampos. University of Ioannina, Medical School, Rheumatology Clinic, Department of Internal Medicine, Ioannina, Greece.

Abstract

INTRODUCTION: Abatacept is a biologic drug that belongs to the class of T-cell co-stimulation modulators and is used for the treatment of rheumatoid arthritis (RA).

AREAS COVERED: This article covers major randomized clinical trials and meta-analyses concerning abatacept in the treatment of RA, as identified in a Pubmed search. Scientific meeting abstracts describing long-term extension data of the identified trials are also included. Efficacy outcomes and the safety profile are the focus of this evaluation.

EXPERT OPINION: Abatacept in combination with methotrexate (MTX) or other synthetic disease-modifying anti-rheumatic drugs (DMARD) has been proven effective for the treatment of RA in different groups of patients: with early RA and no prior exposure to DMARD; with DMARD-resistant RA; and with RA not responding to TNF-alpha-blocking agents. Significant reductions of disease activity are achieved, with 1-year remission rates reaching up to 41% of DMARD-naive patients with early RA receiving a combination of abatacept plus MTX. Abatacept treatment has been shown to improve function and quality of life and to suppress radiographic progression. No major safety issues have emerged during clinical trials and long-term extensions. Therefore,

abatacept is a drug with a favorable efficacy and safety profile, which may offer substantial benefits to RA patients.
Publication Type
Journal Article.
Year of Publication
2011

<549>
Unique Identifier
21624763
Title
Adalimumab desensitization after anaphylactic reaction.
Source
Annals of Allergy, Asthma, & Immunology. 106(6):547-8, 2011 Jun.
VI 1
Status
MEDLINE
Authors
Quercia O; Emiliani F; Foschi FG; Stefanini GF.
Authors Full Name
Quercia, Oliviero; Emiliani, Francesca; Foschi, Francesco Giuseppe; Stefanini, Giuseppe Francesco.
Publication Type
Case Reports. Letter.
Year of Publication
2011

<550>
Unique Identifier

21622275

Title

Advances in homeopathy and immunology: a review of clinical research. [Review]

Source

Frontiers in Bioscience. 3:1363-89, 2011 Jun 01.

VI 1

Status

MEDLINE

Authors

Bellavite P; Marzotto M; Chirumbolo S; Conforti A.

Authors Full Name

Bellavite, Paolo; Marzotto, Marta; Chirumbolo, Salvatore; Conforti, Anita.

Institution

Bellavite, Paolo. Department of Pathology and Diagnostics, University of Verona, Verona, Italy. paolo.bellavite@univr.it

Abstract

The present paper reviews the clinical research carried out over the past three decades to evaluate the effectiveness of homeopathy for the treatment of respiratory allergies, common upper respiratory tract infections, otorhinolaryngologic complaints, and rheumatic diseases. We include in the analysis both randomised and non-randomised trials, assigning them different weightings in the final balance of evidence, on the basis of semi-quantitative criteria. Overall, the literature concerning a total of 83 original studies suggests that homeopathy may have significant effects in some conditions, e.g. Galphimia glauca (low homeopathic dilutions/dynamizations) in allergic oculorhinitis, Anas barbariae (high homeopathic dilution/dynamization) in influenza-like syndromes, classical individualised homeopathy in otitis, in allergic complaints and in fibromyalgia, and a few low-potency homeopathic complexes in sinusitis, rhinoconjunctivitis, arthritis. The evidence for individualised homeopathic therapy in the field of upper respiratory tract infections and for homeopathic immunotherapy in respiratory allergies is more conflicting. Pragmatic equivalence trials suggest that, in primary care, homeopathic treatment is not inferior to conventional treatment. A larger number of observational studies and of clinical trials --conducted in a methodologically correct manner without altering the treatment setting-- are needed before sure conclusions concerning the application of homeopathy for specific diseases can be drawn.

Publication Type

Journal Article. Research Support, Non-U.S. Gov't. Review.

Year of Publication

2011

<551>

Unique Identifier

21621001

Title

Autoimmunity: when the immune system becomes the self-ish giant.

Source

Autoimmunity Reviews. 10(10):575-6, 2011 Aug.

VI 1

Status

MEDLINE

Authors

Perricone R; Perricone C; Shoenfeld Y.

Authors Full Name

Perricone, Roberto; Perricone, Carlo; Shoenfeld, Yehuda.

Institution

Perricone, Roberto. Rheumatology, Allergology and Clinical Immunology, Department of Internal Medicine, University of Rome Tor Vergata, Italy. roberto.perricone@uniroma2.it

Publication Type

Congress.

Year of Publication

2011

<552>

Unique Identifier

21598016

Title

[Vaccination prior to travelling for patients with rheumatic diseases]. [Review] [German]

Source

Zeitschrift fur Rheumatologie. 70(4):292-8, 2011 Jun.
VI 1
Status
MEDLINE
Authors
Ehrenstein B.
Authors Full Name
Ehrenstein, B.
Institution
Ehrenstein, B. Klinik und Poliklinik fur Rheumatologie und Klinische Immunologie, Asklepios Klinikum Bad Abbach, Kaiser-Karl V.-Allee 3, 93077, Bad Abbach, Deutschland. b.ehrenstein@asklepios.com
Abstract
Rheumatologists increasingly face patient questions about the need, the safety and the effectiveness of travel-related vaccinations. Currently, there are no guidelines on travel vaccinations for patients with inflammatory rheumatic diseases. The use of live attenuated vaccines remains contraindicated in patients receiving relevant immunosuppressive therapy despite some encouraging results from initial pilot studies. However, many inactivated travel vaccines can safely be used for patients with rheumatic diseases. Furthermore, rheumatologists should be vigilant in identifying and closing gaps in the routine vaccinations for patients.
Publication Type
Journal Article. Review.
Year of Publication
2011

<553>
Unique Identifier
21560117
Title
Late-onset neutropenia following rituximab therapy in rheumatic diseases: association with B lymphocyte depletion and infections.
Source
Arthritis & Rheumatism. 63(8):2209-14, 2011 Aug.

VI 1

Status

MEDLINE

Authors

Tesfa D; Ajeganova S; Hagglund H; Sander B; Fadeel B; Hafstrom I; Palmblad J.

Authors Full Name

Tesfa, Daniel; Ajeganova, Sofia; Hagglund, Hans; Sander, Birgitta; Fadeel, Bengt; Hafstrom, Ingiald; Palmblad, Jan.

Institution

Tesfa, Daniel. Karolinska Institutet at Karolinska University Hospital Huddinge, Stockholm, Sweden. daniel.tesfa@karolinska.se

Comments

Comment in (CIN)

Abstract

OBJECTIVE: Late-onset neutropenia following rituximab therapy is a well-recognized side effect in lymphoma patients, but only a few cases of late-onset neutropenia have been reported in patients with autoimmune disorders. The purpose of this study was to define the incidence, clinical features, and some of the underlying mechanisms of late-onset neutropenia in relation to rituximab use in several rheumatic diseases.

METHODS: We conducted a retrospective analysis of a cohort of 209 consecutive patients with rheumatic diseases who had been treated with rituximab at a university hospital between June 2003 and March 2009.

RESULTS: Eleven patients with late-onset neutropenia were identified. The highest incidence was observed in granulomatosis with polyangiitis (Wegener's) and systemic lupus erythematosus patients (23% and 20%, respectively), whereas the incidence in rheumatoid arthritis patients was 3%. The median time to onset of neutropenia was 102 days (range 40-362 days) and coincided with the entire period of B lymphocyte depletion; this depletion was more pronounced in patients with late-onset neutropenia (P = 0.002) than in a control group of 20 matched patients without late-onset neutropenia. Serum IgM levels decreased during the same time and to a significantly greater amount in patients with late-onset neutropenia than in controls (P = 0.027). No patient with late-onset neutropenia displayed specific antineutrophil antibodies. Seven patients were hospitalized because of infections (6 with sepsis and 1 with febrile neutropenia) that required intravenous antibiotics. Six were treated with granulocyte colony-stimulating factor.

CONCLUSION: In patients treated with rituximab for rheumatic diseases, late-onset neutropenia is a clinically significant adverse event associated with marked B lymphocyte depletion and severe infections. The incidence of late-onset neutropenia appears to vary with autoimmune disease type. Copyright © 2011 by the American College of Rheumatology.

Publication Type

Journal Article. Research Support, Non-U.S. Gov't.

Year of Publication

2011

<554>

Unique Identifier

21550006

Title

Evaluation of immune response to hepatitis A vaccination and vaccine safety in juvenile idiopathic arthritis.

Source

Journal of the Chinese Medical Association: JCMA. 74(5):205-8, 2011 May.

VI 1

Status

MEDLINE

Authors

Erguven M; Kaya B; Hamzah OY; Tufan F.

Authors Full Name

Erguven, Muferet; Kaya, Bulent; Hamzah, Oznur Yilmaz; Tufan, Fatma.

Institution

Erguven, Muferet. Department of Pediatrics, Ministry of Health, Goztepe Training and Research Hospital, Istanbul, Turkey. muferete@yahoo.com

Abstract

BACKGROUND: Autoimmune mechanisms and drugs used in treatment increase the risk of liver disease in patients with juvenile idiopathic arthritis (JIA) and hepatitis A virus (HAV) vaccination is important, especially in intermediate-endemicity areas like Turkey. In our study, we aimed to evaluate the immune response to hepatitis A vaccine and vaccine safety in children with JIA.

METHODS: This study was carried out in our hospital's Pediatric Rheumatology outpatient clinic and Healthy Child clinic between the years 2003 and 2008. The study group consisted of 47 children with JIA (23 male and 24 female) diagnosed according to International League of Associations for Rheumatology diagnostic criteria. The control group consisted of 67 healthy children (31 female, 36 male) who did not have a history of hepatitis A infection or vaccination. Both groups were vaccinated with two doses of hepatitis A vaccine at 6-month intervals. Anti-HAV IgG >80 MIU was accepted as positive response.

RESULTS: There was no significant difference between the groups in terms of age and sex. None of the patients with JIA had fever, clinical worsening, or disease activation after vaccination. Anti-HAV IgG positivity rate was significantly higher in the control group ($p < 0.05$). Anti-HAV IgG was negative in only four cases, and they were all male patients with systemic JIA who had active disease under anti-tumor necrosis factor treatment.

CONCLUSION: Hepatitis A vaccine was safe in patients with JIA, and response to vaccine did not differ between healthy children and patients with JIA except for children with active systemic JIA receiving anti-tumor necrosis factor alpha drugs. Copyright © 2011. Published by Elsevier B.V.

Publication Type

Journal Article.

Year of Publication

2011

<555>

Unique Identifier

21540203

Title

Immunogenicity and safety of the 2009 non-adjuvanted influenza A/H1N1 vaccine in a large cohort of autoimmune rheumatic diseases.

Source

Annals of the Rheumatic Diseases. 70(6):1068-73, 2011 Jun.

VI 1

Status

MEDLINE

Authors

Saad CG; Borba EF; Aikawa NE; Silva CA; Pereira RM; Calich AL; Moraes JC; Ribeiro AC; Viana VS; Pasoto SG; Carvalho JF; Franca IL; Guedes LK; Shinjo SK; Sampaio-Barros PD; Caleiro MT; Goncalves CR; Fuller R; Levy-Neto M; Timenetsky Mdo C; Precioso AR; Bonfa E.

Authors Full Name

Saad, Carla G S; Borba, Eduardo F; Aikawa, Nadia E; Silva, Clovis A; Pereira, Rosa M R; Calich, Ana Luisa; Moraes, Julio C B; Ribeiro, Ana C M; Viana, Vilma S T; Pasoto, Sandra G; Carvalho, Jozelio F; Franca, Ivan L A; Guedes, Lissiane K N; Shinjo, Samuel K; Sampaio-Barros, Percival D; Caleiro, Maria T; Goncalves, Celio R; Fuller, Ricardo; Levy-Neto, Mauricio; Timenetsky, Maria do Carmo S; Precioso, Alexander R; Bonfa, Eloisa.

Institution

Saad, Carla G S. Division of Rheumatology, Faculdade de Medicina da Universidade de Sao Paulo-Disciplina de Reumatologia, Sao Paulo, Brazil.

Abstract

BACKGROUND: Despite the WHO recommendation that the 2010-2011 trivalent seasonal flu vaccine must contain A/California/7/2009/H1N1-like virus there is no consistent data regarding its immunogenicity and safety in a large autoimmune rheumatic disease (ARD) population.

METHODS: 1668 ARD patients (systemic lupus erythematosus (SLE), rheumatoid arthritis (RA), ankylosing spondylitis (AS), systemic sclerosis, psoriatic arthritis (PsA), Behcet's disease (BD), mixed connective tissue disease, primary antiphospholipid syndrome (PAPS), dermatomyositis (DM), primary Sjogren's syndrome, Takayasu's arteritis, polymyositis and Granulomatosis with polyangiitis (Wegener's) (GPA)) and 234 healthy controls were vaccinated with a non-adjuvanted influenza A/California/7/2009(H1N1) virus-like strain flu. Subjects were evaluated before vaccination and 21 days post-vaccination. The percentage of seroprotection, seroconversion and the factor increase in geometric mean titre (GMT) were calculated.

RESULTS: /st> After immunisation, seroprotection rates (68.5% vs 82.9% p<0.0001), seroconversion rates (63.4% vs 76.9%, p<0.001) and the factor increase in GMT (8.9 vs 13.2 p<0.0001) were significantly lower in ARD than controls. Analysis of specific diseases revealed that seroprotection significantly reduced in SLE (p<0.0001), RA (p<0.0001), PsA (p=0.0006), AS (p=0.04), BD (p=0.04) and DM (p=0.04) patients than controls. The seroconversion rates in SLE (p<0.0001), RA (p<0.0001) and PsA (p=0.0006) patients and the increase in GMTs in SLE (p<0.0001), RA (p<0.0001) and PsA (p<0.0001) patients were also reduced compared with controls. Moderate and severe side effects were not reported.

CONCLUSIONS: The novel recognition of a diverse vaccine immunogenicity profile in distinct ARDs supports the notion that a booster dose may be recommended for diseases with suboptimal immune responses. This large study also settles the issue of vaccine safety. (ClinicalTrials.gov #NCT01151644).

Publication Type

Evaluation Study. Journal Article. Research Support, Non-U.S. Gov't.

Year of Publication

2011

<556>

Unique Identifier

21531476

Title

CD8alpha+ dendritic cells improve collagen-induced arthritis in CC chemokine receptor (CCR)-2 deficient mice.

Source

Immunobiology. 216(9):971-8, 2011 Sep.

VI 1

Status

MEDLINE

Authors

Ibarra JM; Quinones MP; Estrada CA; Jimenez F; Martinez HG; Ahuja SS.

Authors Full Name

Ibarra, Jessica M; Quinones, Marlon P; Estrada, Carlos A; Jimenez, Fabio; Martinez, Hernan G; Ahuja, Seema S.

Institution

Ibarra, Jessica M. Department of Medicine, University of Texas Health Science Center at San Antonio, San Antonio, TX 78229-3900, USA.

Abstract

OBJECTIVE: Dendritic cells (DCs) have long been recognized as potential therapeutic targets of rheumatoid arthritis (RA). Increasing evidence has showed that DCs are capable of suppressing autoimmunity by expanding FoxP3+ regulatory T cells (T(reg)), which in turn exert

immunosuppression by increasing TGFbeta-1. In the SKG mice, activated DC prime autoreactive T cells causing autoantibody production and an inflammatory arthritic response. Recently, we reported that CC-chemokine receptor-2 deficient (Ccr2-/-) mice had impaired DCs migration and reduced CD8alpha+ DCs in the C57Bl/6J mice strain and that these mice were more susceptible to collagen antibody-induced arthritis (CAIA), compared to wild type mice. To examine the mechanism by which DCs contribute to the increased susceptibility of arthritis in Ccr2-/- mice, we tested the hypothesis that CD8alpha+ DCs are protective (tolerogenic) against autoimmune arthritis by examining the role of CD8alpha+ DCs in Ccr2-/- and SKG mice.

METHODS: To examine the mechanism by which DCs defects lead to the development of arthritis, we used two murine models of experimental arthritis: collagen-induced arthritis (CIA) in DBA1/J mice and zymosan-induced arthritis in SKG mice. DBA1/J mice received recombinant fms-like tyrosine kinase 3 ligand (Flt3L) injections to expand endogenous DCs populations or adoptive transfers of CD8alpha+ DCs.

RESULTS: Flt3L-mediated expansion of endogenous CD8alpha+ DCs resulted in heightened susceptibility of CIA. In contrast, supplementation with exogenous CD8alpha+ DCs ameliorated arthritis in Ccr2-/- mice and enhanced TGFbeta1 production by T cells. Furthermore, SKG mice with genetic inactivation of CCR2 did not affect the numbers of DCs nor improve the arthritis phenotype.

CONCLUSION: CD8alpha+ DCs were tolerogenic to the development of arthritis. CD8alpha+ DCs deficiency heightened the sensitivity to arthritis in Ccr2-/- mice. Ccr2 deficiency did not alter the arthritic phenotype in SKG mice suggesting the arthritis in Ccr2-/- mice was T cell-independent. Copyright Published by Elsevier GmbH.

Publication Type

Journal Article. Research Support, N.I.H., Extramural. Research Support, U.S. Gov't, Non-P.H.S..

Year of Publication

2011

<557>

Unique Identifier

21524918

Title

The level of serum visfatin (PBEF) is associated with total number of B cells in patients with rheumatoid arthritis and decreases following B cell depletion therapy.

Source

Cytokine. 55(1):116-21, 2011 Jul.

VI 1

Status

MEDLINE

Authors

Senolt L; Krystufkova O; Hulejova H; Kuklova M; Filkova M; Cerezo LA; Belacek J; Haluzik M; Forejtova S; Gay S; Pavelka K; Vencovsky J.

Authors Full Name

Senolt, Ladislav; Krystufkova, Olga; Hulejova, Hana; Kuklova, Marketa; Filkova, Maria; Cerezo, Lucie Andres; Belacek, Jaromir; Haluzik, Martin; Forejtova, Sarka; Gay, Steffen; Pavelka, Karel; Vencovsky, Jiri.

Institution

Senolt, Ladislav. Institute of Rheumatology, Department of Experimental and Clinical Rheumatology, First Faculty of Medicine, Charles University in Prague, Czech Republic. seno@revma.cz

Abstract

OBJECTIVE: Visfatin, also known as pre-B cell colony-enhancing factor, was recently characterized as a potent pro-inflammatory mediator in rheumatoid arthritis (RA). The aim of this study was to determine the effect of B cell depletion with rituximab on serum visfatin levels in patients with active RA.

METHODS: We evaluated 31 patients with RA starting rituximab therapy at baseline and after 16 and 24 weeks using disease activity score (DAS28). The control group consisted of 33 gender and age-matched healthy individuals. CD19(+) B cells were assessed by flow cytometry and serum levels of visfatin and B cell-activating factor of the TNF family (BAFF) were measured by ELISA at baseline and week 16.

RESULTS: Total number of B cells correlated positively with serum visfatin levels (rs=0.417, P=0.025) and negatively with serum BAFF levels (rs=-0.486, P=0.008) at baseline. Serum visfatin levels were significantly higher in patients with RA compared with healthy controls (P=0.026), and significantly decreased (P=0.010), while BAFF increased (P<0.001), and both proteins became negatively correlated following treatment with rituximab (rs=-0.438, P=0.017). Visfatin levels did

not correlate with the disease activity, but lack of change in the serum visfatin levels between baseline and week 16 predicted worsening disease activity between weeks 16 and 24 (rs=0.452, P=0.014).

CONCLUSION: In patients with active RA, serum visfatin levels are related to the number of B cells rather than to disease activity and decrease in response to treatment with rituximab. Further studies are necessary to show if visfatin is a marker with predictive value for deterioration of RA.
Copyright © 2011 Elsevier Ltd. All rights reserved.
Publication Type
Journal Article. Research Support, Non-U.S. Gov't.
Year of Publication
2011

<558>
Unique Identifier
21515601
Title
Pneumococcal antibody levels after pneumovax in patients with rheumatoid arthritis on methotrexate.
Source
Annals of the Rheumatic Diseases. 70(7):1289-91, 2011 Jul.
VI 1
Status
MEDLINE
Authors
Coulson E; Saravanan V; Hamilton J; So KL; Morgan L; Heycock C; Rynne M; Kelly C.
Authors Full Name
Coulson, Elizabeth; Saravanan, Vadivelu; Hamilton, Jennifer; So, Kin Long; Morgan, Lynn; Heycock, Carol; Rynne, Martin; Kelly, Clive.
Institution
Coulson, Elizabeth. Department of Medicine and Rheumatology, Queen Elizabeth Hospital, Gateshead, UK.
Comments

Erratum in (EIN)

Abstract

INTRODUCTION: Immunisation against pneumococcus has been shown to reduce pneumonia in rheumatoid arthritis (RA). There is concern that methotrexate may reduce its efficacy. There are very few objective data on the effect of methotrexate on the efficacy of pneumococcal vaccination with pneumovax, and no objective evidence on whether revaccination is necessary in RA patients on methotrexate.

METHODS: The authors collected information from 180 RA patients on methotrexate relating to their vaccination status and assayed their pneumococcal antibody levels. Data on pulmonary infection were retrieved in the same patients over the preceding decade.

RESULTS: Full data were available for 152 patients, of whom 28 had never been vaccinated against pneumococcus. Median levels were significantly higher in those who had been vaccinated. Unvaccinated patients and those taking oral prednisone were more likely to have had pneumonia in the previous 10 years. The RR for developing pneumonia among non-vaccinated patients was 9.7 (p=0.005) and among steroid-treated patients was 6.5 (p=0.001), after adjusting for age, gender, disease duration and comorbidity. No significant correlation was found between pneumococcal antibody levels and time since vaccination.

CONCLUSIONS: This study suggests that a single administration of pneumovax early in RA offers up to 10 years protection against the development of pneumococcal pneumonia in RA patients on methotrexate.

Publication Type

Journal Article.

Year of Publication

2011

<559>

Unique Identifier

21514322

Title

Selective elimination of pathogenic synovial fluid T-cells from rheumatoid arthritis and juvenile idiopathic arthritis by targeted activation of Fas-apoptotic signaling.

Source

Immunology Letters. 138(2):161-8, 2011 Aug 30.

VI 1

Status

MEDLINE

Authors

Bremer E; Abdulahad WH; de Bruyn M; Samplonius DF; Kallenberg CG; Armbrust W; Brouwers E; Wajant H; Helfrich W.

Authors Full Name

Bremer, Edwin; Abdulahad, Wayel H; de Bruyn, Marco; Samplonius, Douwe F; Kallenberg, Cees G M; Armbrust, Wineke; Brouwers, E; Wajant, Harald; Helfrich, Wijnand.

Institution

Bremer, Edwin. Department of Surgery, Surgical Research Laboratory, University Medical Center Groningen, University of Groningen, Groningen, The Netherlands.

Abstract

In Rheumatoid Arthritis (RA) and Juvenile Idiopathic Arthritis (JIA) elimination of autoreactive T-cells by FasL/Fas-mediated Activation-Induced Cell Death (AICD) appears to be inhibited resulting in the perpetuation of the inflammatory response and concomitant progressive tissue destruction. Here, we report on a novel strategy that aims to overcome the local inhibition of AICD by using rationally designed recombinant fusion proteins in which sFasL is genetically fused to a T-cell selective targeting domain. The series included sFasL fusion proteins with engineered binding specificity for various T-cell surface-expressed proteins including CD7, CD28, RANKL and CD40L. The proposed mode of action is that selective binding of a given sFasL fusion protein results in its accretion at the cell surface of T-cells only, displaying a surplus of sFasL that is available to reactivate AICD in pathogenic synovial T-cells. Of the series of T-cell targeting FasL fusion proteins a CD7-targeted fusion protein, designated scFvCD7:sFasL, proved to be the most potent, with significant pro-apoptotic activity towards synovial fluid T-cells in all patient samples tested (RA; n=22: JIA; n=6). Treatment with scFvCD7:sFasL induced up to 80% apoptosis in CD3-positive synovial T-cells. Importantly, scFvCD7:sFasL potently activated Fas-signaling in synovial T(H1)-cells as well as synovial T(reg) cells, but not in synovial T(H2) cells. These findings indicate that scFvCD7:sFasL may be of therapeutic value for the selective elimination of pathogenic synovial T-cells of the T(H1) subtype in both RA and JIA. Copyright © 2011 Elsevier B.V. All rights reserved.

Publication Type

Journal Article.

Year of Publication

2011

<560>

Unique Identifier

21504398

Title

Immunological risk factors for infection after immunosuppressive and biologic therapies. [Review]

Source

Expert Review of Antiinfective Therapy. 9(4):405-13, 2011 Apr.

VI 1

Status

MEDLINE

Authors

Carbone J; del Pozo N; Gallego A; Sarmiento E.

Authors Full Name

Carbone, Javier; del Pozo, Nadia; Gallego, Antonio; Sarmiento, Elizabeth.

Institution

Carbone, Javier. Transplant Immunology Group, Clinical Immunology Department, University Hospital Gregorio Maranon, Doctor Esquerdo 46, 28007 Madrid, Spain. jcarbone.hgugm@salud.madrid.org

Abstract

Immunosuppressive and biologic therapies are costly and can involve a considerable risk of infection. Noninvasive diagnostic tools for early prediction of infection before and after administration of these therapies are of major interest. Serial longitudinal immune monitoring would provide data on immunocompetence and complement clinical follow-up protocols. Biomarkers of immune response may be useful to identify patients at risk of developing infection and who could be candidates for immunosuppressant dose reduction. This article focuses on the potential use of biomarkers of immune response to predict development of infection after immunosuppressive and biologic therapies in selected settings of autoimmune disease (rituximab for treatment of rheumatoid arthritis) and solid organ transplantation.

Publication Type

Journal Article. Research Support, Non-U.S. Gov't. Review.
Year of Publication
2011

<561>
Unique Identifier
21489680
Title
Rapid oral desensitisation to prophylactic isoniazid.
Source
Allergologia et Immunopathologia. 39(5):311-2, 2011 Sep-Oct.
VI 1
Status
MEDLINE
Authors
Abadoglu O; Epozturk K; Atayik E.
Authors Full Name
Abadoglu, Oznur; Epozturk, Kursat; Atayik, Emel.
Publication Type
Case Reports. Letter.
Year of Publication
2011

<562>
Unique Identifier
21482543
Title
Vaccination of children and adolescents with rheumatic diseases. [Review]
Source

Rheumatology. 50(8):1358-65, 2011 Aug.

VI 1

Status

MEDLINE

Authors

Dell' Era L; Esposito S; Corona F; Principi N.

Authors Full Name

Dell' Era, Laura; Esposito, Susanna; Corona, Fabrizia; Principi, Nicola.

Institution

Dell' Era, Laura. Department of Maternal and Pediatric Sciences, Universita degli Studi di Milano, Fondazione IRCCS Ca' Granda Ospedale Maggiore Policlinico, Via Commenda 9, 20122 Milano, Italy.

Abstract

Children with rheumatic diseases (RDs) are at greater risk of infection because of their aberrant immunity and frequent use of immunosuppressive drugs. However, the use of vaccinations in such children is debated by many experts who think that the patients' immune response is insufficient to assure protection; some of them are also afraid that vaccines could trigger a persistent autoimmune response and lead to severe clinical problems including a relapse of the RD. This review describes the available data regarding the risks of vaccine administration, and the immunogenicity, efficacy and tolerability of the vaccines usually recommended for children with RDs. The data not only show that the schedule suggested for otherwise healthy children should be followed, but also that pneumococcal and influenza vaccinations should be strongly recommended because of the known risk of severe infections in patients with RD. However, there are areas in which further research is urgently required.

Publication Type

Journal Article. Research Support, Non-U.S. Gov't. Review.

Year of Publication

2011

<563>

Unique Identifier

21464925

Title

Location of immunization and interferon-gamma are central to induction of salivary gland dysfunction in Ro60 peptide immunized model of Sjogren's syndrome.

Source

PLoS ONE [Electronic Resource]. 6(3):e18003, 2011 Mar 28.

VI 1

Status

MEDLINE

Authors

Yin H; Vosters JL; Roescher N; D'Souza A; Kurien BT; Tak PP; Chiorini JA.

Authors Full Name

Yin, Hongen; Vosters, Jelle L; Roescher, Nienke; D'Souza, Anil; Kurien, Biji T; Tak, Paul P; Chiorini, John A.

Institution

Yin, Hongen. Molecular Physiology and Therapeutics Branch, National Institute of Dental and Craniofacial Research, National Institutes of Health, Bethesda, Maryland, United States of America.

Abstract

INTRODUCTION: Anti-Ro antibodies can be found in the serum of the majority of patients with Sjogren's syndrome (SS). Immunization with a 60-kDa Ro peptide has been shown to induce SS-like symptoms in mice. The aim of this study was to investigate factors involved in salivary gland (SG) dysfunction after immunization and to test whether the induction of SS could be improved.

METHODS: Ro60 peptide immunization was tested in Balb/c mice, multiple antigenic peptide (MAP)-Ro60 and Pertussis toxin (PTX) were tested in SJL/J mice. In addition, two injection sites were compared in these two strains: the abdominal area and the tailbase. Each group of mice was tested for a loss of SG function, SG lymphocytic infiltration, anti-Ro and anti-La antibody formation, and cytokine production in cultured cells or homogenized SG extracts.

RESULTS: Ro60 peptide immunization in the abdominal area of female Balb/c mice led to impaired SG function, which corresponded with increased Th1 cytokines (IFN-gamma and IL-12) systemically and locally in the SG. Moreover, changing the immunization conditions to MAP-Ro60 in the abdominal area, and to lesser extend in the tailbase, also led to impaired SG function in SJL/J mice. As was seen in the Balb/c mice, increased IFN-gamma in the SG draining lymph nodes accompanied the SG dysfunction. However, no correlation was observed with anti-MAP-Ro60 antibody titers, and there was no additional effect on disease onset or severity.

CONCLUSIONS: Effective induction of salivary gland dysfunction after Ro60 peptide immunization depended on the site of injection. Disease induction was not affected by changing the immunization conditions. However, of interest is that the mechanism of action of Ro60 peptide immunization appears to involve an increase in Th1 cytokines, resulting in the induction of SG dysfunction.

Publication Type

Journal Article. Research Support, Non-U.S. Gov't.

Year of Publication

2011

<564>

Unique Identifier

21457124

Title

Biological therapies in primary Sjogren's syndrome. [Review]

Source

Expert Opinion on Biological Therapy. 11(7):921-36, 2011 Jul.

VI 1

Status

MEDLINE

Authors

Ng WF; Bowman SJ.

Authors Full Name

Ng, Wan-Fai; Bowman, Simon J.

Institution

Ng, Wan-Fai. University of Newcastle, Institute of Cellular Medicine, Musculoskeletal Research Group, Newcastle upon Tyne, NE2 4HH, UK. wan-fai.ng@ncl.ac.uk

Abstract

INTRODUCTION: Primary Sjogren's syndrome (PSS) is a relatively common immune-mediated condition characterized by oral and ocular dryness, fatigue, musculoskeletal pain and poor health-related quality of life. Other extra-glandular organs can also be affected and PSS is associated with a markedly increased risk of lymphoma. Furthermore, the health-economic cost for PSS is substantial. There is currently no effective treatment available. With better

understanding of the pathophysiology of PSS and advances in technologies, it is now possible to develop biological therapies to target specific molecules or molecular pathways that are important in PSS pathogenesis. Indeed, a limited number of biological therapies have already been tested in PSS with mixed successes.

AREAS COVERED: Published data on the use of biological therapies in PSS, the possible roles for other biological therapies and the potential challenges for their use.

EXPERT OPINION: The use of biological agents targeting key cellular and molecular pathways in PSS pathogenesis represents a promising therapeutic strategy. Clinical trials assessing the efficacy of biological therapies in PSS should be encouraged but patient selection and outcome measures used in these studies must be carefully considered to ensure that the true effects of biological therapies on the outcomes of PSS are being appropriately evaluated.

Publication Type

Journal Article. Research Support, Non-U.S. Gov't. Review.

Year of Publication

2011

<565>

Unique Identifier

21453269

Title

Clinical applications of autoimmunity to citrullinated proteins in rheumatoid arthritis, from improving diagnostics to future therapies. [Review]

Source

Recent Patents on Inflammation & Allergy Drug Discovery. 5(2):108-27, 2011 May.

VI 1

Status

MEDLINE

Authors

Kinloch AJ; Ng K; Wright GP.

Authors Full Name

Kinloch, Andrew J; Ng, Karen; Wright, Graham P.

Institution

Kinloch, Andrew J. Department of Medicine, Knapp Center for Lupus and Immunology Research, University of Chicago, Chicago, IL 60637, USA. ajkinloch1@uchicago.edu

Abstract

Rheumatoid arthritis (RA), although widely considered to be the most commonly occurring autoimmune disease, has only truly been substantiated as a distinct autoimmune disease very recently. The lack of understanding of the specific autoimmune system/s at work in rheumatoid patients resulted in an absence of robust diagnostic tools and had meant that the rational choice for use and design of therapy was based on broad-spectrum immunosuppression. The revelation that the autoimmune response specific for patients with RA is to particular protein antigens bearing the post-translational modification 'citrulline' has therefore revolutionized diagnostics and has helped explain why patients carrying particular MHC alleles are predisposed to the disease. The last two decades have seen the characterization of citrullinated antigens targeted by both antibodies and T cells in rheumatoid patients. In more recent years, we have also witnessed the success of biological therapies in the treatment of RA that specifically target T cells and B cells. Ongoing mapping of antibody targets is increasing the percentage of patients who can be definitively diagnosed with, and prognosed to develop, RA. These advances have led to a great number of patents for citrullinated peptides that have been and may be, in the coming years, used in diagnostic test kits. More recently, characterization of T cell targets (citrullinated peptides) has resulted in the patenting of peptides that could be used in antigen specific therapy. This review focuses on the characterization of the autoimmune response to citrullinated protein targets in RA and how the community is translating this knowledge to improve diagnostics, prognostics and therapy.

Publication Type

Journal Article. Research Support, Non-U.S. Gov't. Review.

Year of Publication

2011

<566>

Unique Identifier

21452309

Title

Is there a need for immunopharmacologic guidance of anti-tumor necrosis factor therapies?.

Source

Arthritis & Rheumatism. 63(4):867-70, 2011 Apr.

VI 1

Status

MEDLINE

Authors

Bendtzen K.

Authors Full Name

Bendtzen, Klaus.

Comments

Comment on (CON)

Publication Type

Comment. Editorial.

Year of Publication

2011

<567>

Unique Identifier

21427579

Title

New therapies in the management of rheumatoid arthritis. [Review]

Source

Current Opinion in Rheumatology. 23(3):245-51, 2011 May.

VI 1

Status

MEDLINE

Authors

Buch MH; Emery P.

Authors Full Name

Buch, Maya H; Emery, Paul.

Institution

Buch, Maya H. Section of Musculoskeletal Disease, Leeds Institute of Molecular Medicine, University of Leeds, UK.

Abstract
PURPOSE OF REVIEW: The therapeutic landscape in the management of rheumatoid arthritis (RA) has witnessed significant changes over the past decade. The ambition to improve outcomes further, minimize safety concerns and provide more convenient means of administration are all factors that continue to drive continued drug development. This review summarizes novel therapies that have been most recently under investigation.

RECENT FINDINGS: More refined drug technology has seen the development of subcutaneous forms of existing therapies (abatacept, tocilizumab), as well as newer-generation monoclonal antibodies (e.g. B-cell-depleting agents, ocrelizumab and ofatumumab and the TNF-inhibitors certolizumab and golimumab). Alternative methods of targeting critical pathways, for example Blys inhibition (atacicept) and IL-6 as opposed to IL-6 receptor antagonism, have also been evaluated. Finally, small molecules are receiving increasing attention, with some of the protein kinases inhibitors particularly promising.

SUMMARY: The new emerging therapies for the management of RA illustrate much diversity, in terms of both drug technology as well as the immunological target. Although not all may succeed in reaching the market, important insights can still be gained. Challenging and exciting times lie ahead as these new technologies are embraced and efforts are made to determine how best to implement in practice.
Publication Type
Journal Article. Review.
Year of Publication
2011

<568>
Unique Identifier
21416774
Title
Merkel cell carcinoma in a patient treated with adalimumab: case report.
Source
Cutis. 87(2):81-4, 2011 Feb.
VI 1

Status

MEDLINE

Authors

Krishna SM; Kim CN.

Authors Full Name

Krishna, Sheila M; Kim, Christina N.

Institution

Krishna, Sheila M. David Geffen School of Medicine, University of California, Los Angeles, CA 90095, USA.

Comments

Erratum in (EIN)

Abstract

Merkel cell carcinoma (MCC) is a rare aggressive neuroendocrine tumor that manifests as an asymptomatic enlarging lesion often in the setting of immunosuppression, advanced age, or UV exposure. Immunosuppression has been associated with melanoma, lymphoma, and nonmelanoma skin cancer (NMSC). We present a case of a patient with a long-standing history of rheumatoid arthritis treated with adalimumab, methotrexate, and prednisone who developed a painless, rapidly enlarging lesion that was found to be MCC with lymph node involvement. As the use of tumor necrosis factor (TNF) alpha inhibitors becomes more popular, it is important to identify the potential long-term risks associated with chronic immune modulation. Systemic immunosuppression may be a risk factor for the development of advanced-stage MCC. Treatment with the TNF-alpha inhibitor adalimumab may enhance this risk.

Publication Type

Case Reports. Journal Article.

Year of Publication

2011

<569>

Unique Identifier

21384257

Title

Efficacy and safety of repeat courses of rituximab treatment in patients with severe refractory juvenile idiopathic arthritis.

Source

Clinical Rheumatology. 30(9):1163-72, 2011 Sep.

VI 1

Status

MEDLINE

Authors

Alexeeva EI; Valieva SI; Bzarova TM; Semikina EL; Isaeva KB; Lisitsyn AO; Denisova RV; Chistyakova EG.

Authors Full Name

Alexeeva, Ekaterina I; Valieva, Saniya I; Bzarova, Tatyana M; Semikina, Elena L; Isaeva, Kseniya B; Lisitsyn, Alexander O; Denisova, Rina V; Chistyakova, Evgeniya G.

Institution

Alexeeva, Ekaterina I. Rheumatology Department, Scientific Center for Children's Health, Russian Academy of Medical Sciences, 2/62 Lomonosovsky Avenue, Moscow, Russia. alekatya@yandex.ru

Abstract

Treatment of severe juvenile idiopathic arthritis (JIA) represents a serious challenge. This study investigates the efficacy and safety of repeat courses of rituximab in patients with different forms of JIA refractory to infliximab and standard immunosuppressive therapy. Patients (n = 55; age 2.3-17.0 years) with severe polyarticular and systemic JIA (International League of Association for Rheumatology diagnostic criteria) received rituximab (one intravenous infusion/week for 4 weeks, 375 mg/m(2) per dose). Efficacy was assessed using the American College of Rheumatology Pediatric (ACR Pedi) criteria. The primary endpoint was an ACR Pedi 30 response at week 24. At week 24, ACR Pedi 30, 50, and 70 responses were achieved by 98%, 50%, and 40% of patients, respectively. By week 96, ACR Pedi 30, 50, and 70 responses were achieved by 98%, 93%, and 93% of 25 patients, respectively. Remission was recorded in 25%, 52%, 75%, and 98% of patients following the first (24 weeks), second (48 weeks), third (72 weeks), and fourth (96 weeks) courses of rituximab, respectively. Rituximab treatment significantly reduced the number of systemic manifestations at week 12 and also enabled 52% of patients to achieve remission of arthritis by week 48. This study supports the efficacy of rituximab in patients with severe forms of JIA, refractory to several prior agents.

Publication Type

Journal Article. Research Support, Non-U.S. Gov't.

Year of Publication

2011

<570>

Unique Identifier

21360489

Title

Reduced-dose rituximab in rheumatoid arthritis: efficacy depends on degree of B cell depletion.

Source

Arthritis & Rheumatism. 63(3):603-8, 2011 Mar.

VI 1

Status

MEDLINE

Authors

Vital EM; Rawstron AC; Dass S; Henshaw K; Madden J; Emery P; McGonagle D.

Authors Full Name

Vital, Edward M; Rawstron, Andrew C; Dass, Shouvik; Henshaw, Karen; Madden, Julie; Emery, Paul; McGonagle, Dennis.

Institution

Vital, Edward M. National Institute for Health Research Leeds Musculoskeletal Biomedical Research Unit and University of Leeds, Leeds, UK.

Comments

Comment in (CIN)

Abstract

OBJECTIVE: Studies comparing 500 mg rituximab and 1,000 mg rituximab doses in rheumatoid arthritis have yielded conflicting data on clinical outcomes, but in all of these studies a subgroup of patients has had excellent responses at the lower dose. Historically, it was considered that rituximab uniformly depleted B cells at both doses. Using highly sensitive assays, we have shown that B cell depletion is variable and predictive of clinical response. Using the same techniques, we undertook the present study to test the hypothesis that the level of B cell depletion, rather than the rituximab dose, determines clinical response.

METHODS: Nineteen patients were treated with two 500-mg infusions of rituximab, and 61 patients were treated with two 1,000-mg infusions of rituximab. Highly sensitive flow cytometry was performed at 0, 2, 6, 14, and 26 weeks. European League Against Rheumatism (EULAR) response rates at 6 months were compared between patients with and those without complete depletion at each dose.

RESULTS: The median B cell count was numerically higher at all time points following therapy in the 500 mg rituximab group. Twenty-five percent of patients in the 500 mg rituximab group had complete depletion at 2 weeks, compared with 49% of those in the 1,000 mg rituximab group. Complete depletion at 2 weeks after treatment with 500 mg rituximab was associated with lower baseline preplasma cell counts (P = 0.047). Most patients responded after either dose, but response was related to B cell depletion. Notably, in the 500 mg rituximab group all patients with complete depletion had a EULAR good response (P = 0.011).

CONCLUSION: This pilot study suggests that the degree of B cell depletion, rather than the dose of rituximab, determines clinical response. It may be possible to predict which patients will respond to lower-dose rituximab, and this may allow more cost-effective treatment. Copyright © 2011 by the American College of Rheumatology.

Publication Type

Journal Article. Research Support, Non-U.S. Gov't.

Year of Publication

2011

<571>

Unique Identifier

21360487

Title

More or less rituximab? Biology and clinic, regulators and researchers.

Source

Arthritis & Rheumatism. 63(3):594-6, 2011 Mar.

VI 1

Status

MEDLINE

Authors

van Vollenhoven RF.

Authors Full Name

van Vollenhoven, Ronald F.

Comments

Comment on (CON)

Publication Type

Editorial. Comment.

Year of Publication

2011

<572>

Unique Identifier

21346237

Title

Interplay between TNF and regulatory T cells in a TNF-driven murine model of arthritis.

Source

Journal of Immunology. 186(7):3899-910, 2011 Apr 01.

VI 1

Status

MEDLINE

Authors

Biton J; Semerano L; Delavallee L; Lemeiter D; Laborie M; Grouard-Vogel G; Boissier MC; Bessis N.

Authors Full Name

Biton, Jerome; Semerano, Luca; Delavallee, Laure; Lemeiter, Delphine; Laborie, Marion; Grouard-Vogel, Geraldine; Boissier, Marie-Christophe; Bessis, Natacha.

Institution

Biton, Jerome. EA4222, Physiopatologie et Biotherapies de la Polyarthrite Rhumatoide, Sorbonne Paris Cite-Universite Paris 13, 93000 Bobigny, France.

Comments

Comment in (CIN)

Abstract

CD4(+)CD25(+)Foxp3(+) regulatory T cells (Treg) are involved in several autoimmune diseases, including rheumatoid arthritis. TNF-alpha blockers induce therapeutic benefits in rheumatoid arthritis via a variety of mechanisms. We aimed to characterize the impact on Treg of TNF-alpha overexpression in vivo and of TNF-alpha inhibiting treatments. We used human TNF-alpha transgenic mice as a model of strictly TNF-alpha-dependent arthritis. Our study showed that initial

Treg frequency was lower in TNF-alpha transgenic mice than in wild-type mice. However, the course of arthritis was marked by elevation of Treg frequency and a dramatic increase in expression of TNFR2. Antagonizing TNF-alpha with either the anti-human TNF-alpha Ab (infliximab) or active immunotherapy (TNF-kinoid) increased the Treg frequency and upregulated CTLA-4, leading to enhancement of suppressor activity. Moreover, both anti-TNF-alpha strategies promoted the differentiation of a CD62L(-) Treg population. In conclusion, in an in vivo model of TNF-alpha-driven arthritis, Treg frequency increased with inflammation but failed to control the inflammatory process. Both passive and active TNF-alpha-inhibiting strategies restored the suppressor activity of Treg and induced the differentiation of a CD62L(-) Treg population.

Publication Type

Comparative Study. Journal Article. Research Support, Non-U.S. Gov't.

Year of Publication

2011

<573>

Unique Identifier

21327453

Title

Onset of adult-onset Still's disease following influenza vaccination.

Source

Modern Rheumatology. 21(4):432-5, 2011 Aug.

VI 1

Status

MEDLINE

Authors

Yoshioka K; Fujimoto S; Oba H; Minami M; Aoki T.

Authors Full Name

Yoshioka, Katsunobu; Fujimoto, Shunsuke; Oba, Hiroko; Minami, Mieko; Aoki, Tetsuya.

Institution

Yoshioka, Katsunobu. Department of Internal Medicine, Osaka City Sumiyoshi Hospital, 1-2-16 Higashikagaya, Suminoe-ku, Osaka, Osaka, 559-0012, Japan. kmyoshi@rr.iij4u.or.jp

Abstract

We describe that case of a 61-year-old woman who developed high spiking fever, sore throat, polyarthralgia, and salmon pink evanescent rash following influenza vaccination. A diagnosis of adult-onset Still's disease (AOSD) was made based on clinical and laboratory findings. Methylprednisolone pulse therapy followed by oral prednisolone resulted in a favorable outcome. This is the second published case in which a causal relationship between vaccination and onset of AOSD is suggested. Bystander activation would appear to play an important role in inducing the immune reaction.

Publication Type

Case Reports. Journal Article.

Year of Publication

2011

<574>

Unique Identifier

21305522

Title

Proteoglycan-induced arthritis and recombinant human proteoglycan aggrecan G1 domain-induced arthritis in BALB/c mice resembling two subtypes of rheumatoid arthritis.

Source

Arthritis & Rheumatism. 63(5):1312-21, 2011 May.

VI 1

Status

MEDLINE

Authors

Glant TT; Radacs M; Nagyeri G; Olasz K; Laszlo A; Boldizsar F; Hegyi A; Finnegan A; Mikecz K.

Authors Full Name

Glant, Tibor T; Radacs, Marianna; Nagyeri, Gyorgy; Olasz, Katalin; Laszlo, Anna; Boldizsar, Ferenc; Hegyi, Akos; Finnegan, Alison; Mikecz, Katalin.

Institution

Glant, Tibor T. Rush University Medical Center, Chicago, Illinois. Tibor_Glant@rsh.net

Abstract

OBJECTIVE: To develop a simplified and relatively inexpensive version of cartilage proteoglycan-induced arthritis (PGIA), an autoimmunity model of rheumatoid arthritis (RA), and to evaluate the extent to which this new model replicates the disease parameters of PGIA and RA.

METHODS: Recombinant human G1 domain of human cartilage PG containing "arthritogenic" T cell epitopes was generated in a mammalian expression system and used for immunization of BALB/c mice. The development and progression of arthritis in recombinant human PG G1-immunized mice (designated recombinant human PG G1-induced arthritis [GIA]) was monitored, and disease parameters were compared with those in the parent PGIA model.

RESULTS: GIA strongly resembled PGIA, although the clinical symptoms and immune responses in mice with GIA were more uniform than in those with PGIA. Mice with GIA showed evidence of stronger Th1 and Th17 polarization than those with PGIA, and anti-mouse PG autoantibodies were produced in different isotype ratios in the 2 models. Rheumatoid factor (RF) and anti-cyclic citrullinated peptide (anti-CCP) antibodies were detected in both models; however, serum levels of IgG-RF and anti-CCP antibodies were different in GIA and PGIA, and both parameters correlated better with disease severity in GIA than in PGIA.

CONCLUSION: GIA is a novel model of seropositive RA that exhibits all of the characteristics of PGIA. Although the clinical phenotypes are similar, GIA and PGIA are characterized by different autoantibody profiles, and the 2 models may represent 2 subtypes of seropositive RA, in which more than 1 type of autoantibody can be used to monitor disease severity and response to treatment. Copyright © 2011 by the American College of Rheumatology.

Publication Type

Journal Article. Research Support, N.I.H., Extramural. Research Support, Non-U.S. Gov't.

Year of Publication

2011

<575>

Unique Identifier

21303474

Title

Vaccination as a triggering agent for the development of rheumatoid arthritis.

Source

International Journal of Rheumatic Diseases. 14(1):e8-9, 2011 Feb.

VI 1

Status

MEDLINE

Authors

Sharma A; Agarwal D; Kapoor S; Garg SR; Malaviya AN.

Authors Full Name

Sharma, Amit; Agarwal, Divya; Kapoor, Sanjeev; Garg, Shri Ram; Malaviya, Anand Narayan.

Publication Type

Case Reports. Letter.

Year of Publication

2011

<576>

Unique Identifier

21298002

Title

Prostaglandin E2 synthesizing enzymes in rheumatoid arthritis B cells and the effects of B cell depleting therapy on enzyme expression.

Source

PLoS ONE [Electronic Resource]. 6(1):e16378, 2011 Jan 27.

VI 1

Status

MEDLINE

Authors

Gheorghe KR; Thurlings RM; Westman M; Boumans MJ; Malmstrom V; Trollmo C; Korotkova M; Jakobsson PJ; Tak PP.

Authors Full Name

Gheorghe, Karina Roxana; Thurlings, Rogier M; Westman, Marie; Boumans, Maartje J; Malmstrom, Vivianne; Trollmo, Christina; Korotkova, Marina; Jakobsson, Per-Johan; Tak, Paul-Peter.

Institution

Gheorghe, Karina Roxana. Rheumatology Unit, Department of Medicine, Karolinska Institute, Karolinska University Hospital Solna, Stockholm, Sweden.
Abstract
INTRODUCTION: B cells may play an important role in promoting immune activation in the rheumatoid synovium and can produce prostaglandin E(2) (PGE(2)) when activated. In its turn, PGE(2) formed by cyclooxygenase (COX) and microsomal prostaglandin E(2) synthase 1 (MPGES1) contributes to the rheumatoid arthritis (RA) pathological process. Therapeutic depletion of B cells results in important improvement in controlling disease activity in rheumatoid patients. Therefore we investigated the expression of PGE(2) pathway enzymes in RA B cells and evaluated the effects of B cell depleting therapy on their expression in RA tissue.

METHODS: B cells expressing MPGES1 and COX-2 were identified by flow cytometry in in vitro stimulated and control mononuclear cells isolated from synovial fluid and peripheral blood of RA patients. Synovial biopsies were obtained from 24 RA patients before and at two consecutive time points after rituximab therapy. Expression of MPGES1, COX-1 and COX-2, as well as interleukin (IL)-1beta and IL-6, known inducers of MPGES1, was quantified in immunostained biopsy sections using computerized image analysis.

RESULTS: Expression of MPGES1 or COX-2 was significantly upregulated upon stimulation of B cells from blood and synovial fluid while control cells displayed no detectable enzymes. In synovial biopsy sections, the expression of MPGES1, COX-1 or COX-2 was resistant to rituximab therapy at 8 or 16 weeks after start of treatment. Furthermore expression of IL-1beta in the synovial tissue remained unchanged, while IL-6 tended to decrease after therapy.

CONCLUSIONS: Therapy with B cell depleting agents, although efficient in achieving good clinical and radiographic response in RA patients, leaves important inflammatory pathways in the rheumatoid synovium essentially unaffected.
Publication Type
Journal Article. Research Support, Non-U.S. Gov't.
Year of Publication
2011

<577>

Unique Identifier

21253584

Title

Current concepts: mouse models of Sjogren's syndrome. [Review]

Source

Journal of Biomedicine & Biotechnology. 2011:549107, 2011.

VI 1

Status

MEDLINE

Authors

Lavoie TN; Lee BH; Nguyen CQ.

Authors Full Name

Lavoie, Tegan N; Lee, Byung Ha; Nguyen, Cuong Q.

Institution

Lavoie, Tegan N. Department of Oral Biology, College of Dentistry, University of Florida, P.O. Box 100424, 1600 SW Archer Road, Gainesville, FL 32610, USA.

Abstract

Sjogren's syndrome (SjS) is a complex chronic autoimmune disease of unknown etiology which primarily targets the exocrine glands, resulting in eventual loss of secretory function. The disease can present as either primary SjS or secondary SjS, the latter of which occurs concomitantly with another autoimmune disease such as rheumatoid arthritis, systemic lupus erythematosus, scleroderma, or primary biliary cirrhosis. Current advancements in therapeutic prevention and treatment for SjS are impeded by lack of understanding in the pathophysiological and clinical progression of the disease. Development of appropriate mouse models for both primary and secondary SjS is needed in order to advance knowledge of this disease. This paper details important features, advantages, and pitfalls of current animal models of SjS, including spontaneous, transgenic, knockout, immunization, and transplantation chimera mouse models, and emphasizes the need for a better model in representing the human SjS phenotype.

Publication Type

Journal Article. Research Support, N.I.H., Extramural. Research Support, Non-U.S. Gov't. Review.

Year of Publication

2011

<578>

Unique Identifier

21188451

Title

Disseminated cutaneous and visceral Kaposi's sarcoma in a patient with rheumatoid arthritis receiving corticosteroids and tacrolimus.

Source

Modern Rheumatology. 21(3):309-12, 2011 Jun.

VI 1

Status

MEDLINE

Authors

Taniguchi T; Asano Y; Kawaguchi M; Kogure A; Mitsui H; Sugaya M; Sato S.

Authors Full Name

Taniguchi, Takashi; Asano, Yoshihide; Kawaguchi, Makiko; Kogure, Asako; Mitsui, Hiroshi; Sugaya, Makoto; Sato, Shinichi.

Institution

Taniguchi, Takashi. Department of Dermatology, Faculty of Medicine, University of Tokyo, 7-3-1 Hongo, Bunkyo-ku, Tokyo 113-8655, Japan.

Abstract

Kaposi's sarcoma (KS) is a vascular lesion of low-grade malignant potential caused by the complex interactions between geographic, genetic, environmental, and immunological factors. We recently experienced a rare case of KS associated with rheumatoid arthritis in a patient receiving corticosteroids and tacrolimus; the KS demonstrated unusually aggressive clinical behavior. We herein report the details of the clinical course and discuss the possible contribution of corticosteroids and tacrolimus to the development of aggressive KS in the present case.

Publication Type

Case Reports. Journal Article.

Year of Publication

2011

<579>

Unique Identifier

21182987

Title

Vaccination in adult patients with auto-immune inflammatory rheumatic diseases: a systematic literature review for the European League Against Rheumatism evidence-based recommendations for vaccination in adult patients with auto-immune inflammatory rheumatic diseases. [Review]

Source

Autoimmunity Reviews. 10(6):341-52, 2011 Apr.

VI 1

Status

MEDLINE

Authors

van Assen S; Elkayam O; Agmon-Levin N; Cervera R; Doran MF; Dougados M; Emery P; Geborek P; Ioannidis JP; Jayne DR; Kallenberg CG; Muller-Ladner U; Shoenfeld Y; Stojanovich L; Valesini G; Wulffraat NM; Bijl M.

Authors Full Name

van Assen, S; Elkayam, O; Agmon-Levin, N; Cervera, R; Doran, M F; Dougados, M; Emery, P; Geborek, P; Ioannidis, J P A; Jayne, D R W; Kallenberg, C G M; Muller-Ladner, U; Shoenfeld, Y; Stojanovich, L; Valesini, G; Wulffraat, N M; Bijl, M.

Institution

van Assen, S. Department of Internal Medicine, University Medical Center Groningen, Groningen, The Netherlands. s.van.assen@int.umcg.nl

Abstract

OBJECTIVES: To present the systematic literature review (SLR), which formed the basis for the European League Against Rheumatism (EULAR) evidence-based recommendations for vaccination in adult patients with auto-immune inflammatory rheumatic diseases (AIIRD).

METHODS: AIIRD, vaccines and immunomodulating drugs, as well as eight key questions were defined by the multidisciplinary expert committee commissioned by EULAR for developing the recommendations. A SLR was performed using MedLine through October 2009 and including data from meta-analyses, systematic reviews, randomized trials, and observational studies, excluding case series with <= 5 participants. Articles in English and regarding patients >= 16 years of age, were eligible.

RESULTS: Several vaccine-preventable infections (VPI) occur more often in AIIRD-patients and most vaccines are efficacious in AIIRD-patients, even when treated with immunomodulating

agents, except rituximab. There does not appear to be an increase in vaccination-related harms in vaccinated patients with AIIRD in comparison with unvaccinated patients with AIIRD. However, these studies are underpowered and therefore not conclusive.

CONCLUSION: Based on the current evidence from the literature, recommendations for vaccination in patients with AIIRD were made. However, more research is needed in particular regarding incidence of VPI, harms of vaccination and the influence of (new and established) immunomodulating agents on vaccination efficacy. Copyright © 2011 Elsevier B.V. All rights reserved.

Publication Type

Journal Article. Research Support, Non-U.S. Gov't. Review. Systematic Review.

Year of Publication

2011

<580>

Unique Identifier

21169045

Title

The uptake of influenza and pneumococcal vaccination among immunocompromised patients attending rheumatology outpatient clinics.

Source

Joint, Bone, Spine: Revue du Rhumatisme. 78(4):374-7, 2011 Jul.

VI 1

Status

MEDLINE

Authors

Haroon M; Adeeb F; Eltahir A; Harney S.

Authors Full Name

Haroon, Muhammad; Adeeb, Fahd; Eltahir, Ahmed; Harney, Sinead.

Institution

Haroon, Muhammad. Department of Rheumatology, Cork University Hospital, Cork, Ireland. mharoon301@hotmail.com

Abstract

PURPOSE AND OBJECTIVES: The patients using immunosuppressive agents are considered at high risk for acquiring different infections. Accordingly, international guidelines recommend vaccinating such patients against influenza and pneumococcal organisms. The aims of this study were two-fold: (1) to assess the influenza and pneumococcal vaccination uptake among our rheumatology outpatients who are immunosuppressed; (2) to identify the factors influencing immunisation uptake among our sample of patients.

METHODS: This was a questionnaire-based study. Patients were eligible to partake in this study if they were using immunosuppressive drugs. During the study period (4 weeks), 337 patients were screened, and 110 patients fulfilled the criteria for inclusion.

RESULTS: Positive vaccination uptake of our cohort was as follows: common influenza alone (34%, 37 out of 110), pneumonia alone (11%, 12 out of 110), and both pneumococcal and influenza vaccination (11%). The status of influenza A (H1N1) vaccination was not recorded as a part of this audit. The two most common reasons cited by patients for non-uptake of vaccinations were: 'not offered' and 'thought it was unnecessary'. Of 37 patients who had influenza vaccination, 33 patients (89%) had additional risk factors, and there were only four patients who had influenza vaccine solely because they were taking immunosuppressive drugs. All pneumococcal vaccinated patients (n=12) were noted to have additional risk factors.

CONCLUSION: There is suboptimal uptake of influenza and pneumococcal vaccinations among immunosuppressed patients attending rheumatology outpatient clinics. These results are a cause of concern given the morbidity and mortality of associated infections. Copyright © 2010 Societe francaise de rhumatologie. Published by Elsevier SAS. All rights reserved.

Publication Type

Journal Article.

Year of Publication

2011

<581>

Unique Identifier

21131643

Title

EULAR recommendations for vaccination in adult patients with autoimmune inflammatory rheumatic diseases.

Source

Annals of the Rheumatic Diseases. 70(3):414-22, 2011 Mar.

VI 1

Status

MEDLINE

Authors

van Assen S; Agmon-Levin N; Elkayam O; Cervera R; Doran MF; Dougados M; Emery P; Geborek P; Ioannidis JP; Jayne DR; Kallenberg CG; Muller-Ladner U; Shoenfeld Y; Stojanovich L; Valesini G; Wulffraat NM; Bijl M.

Authors Full Name

van Assen, S; Agmon-Levin, N; Elkayam, O; Cervera, R; Doran, M F; Dougados, M; Emery, P; Geborek, P; Ioannidis, J P A; Jayne, D R W; Kallenberg, C G M; Muller-Ladner, U; Shoenfeld, Y; Stojanovich, L; Valesini, G; Wulffraat, N M; Bijl, M.

Institution

van Assen, S. Department of Internal Medicine, Division of Infectious Diseases, University Medical Centre Groningen, AA41 P O Box 30 001, 9700 RB Groningen, The Netherlands. s.van.assen@int.umcg.nl

Abstract

OBJECTIVES: To develop evidence-based European League Against Rheumatism (EULAR) recommendations for vaccination in patients with autoimmune inflammatory rheumatic diseases (AIIRD).

METHODS: A EULAR task force was composed of experts representing 11 European countries, consisting of eight rheumatologists, four clinical immunologists, one rheumatologist/clinical immunologist, one infectious disease physician, one nephrologist, one paediatrician/rheumatologist and one clinical epidemiologist. Key questions were formulated and the eligible spectrum of AIIRD, immunosuppressive drugs and vaccines were defined in order to perform a systematic literature review. A search was made of Medline from 1966 to October 2009 as well as abstracts from the EULAR meetings of 2008 and 2009 and the American College of Rheumatology (ACR) meetings of 2007 and 2008. Evidence was graded in categories I-IV, the strength of recommendations was graded in categories A-D and Delphi voting was applied to determine the level of agreement between the experts of the task force.

RESULTS: Eight key questions and 13 recommendations addressing vaccination in patients with AIIRD were formulated. The strength of each recommendation was determined. Delphi

voting revealed a very high level of agreement with the recommendations among the experts of the task force. Finally, a research agenda was proposed.

CONCLUSION: Recommendations for vaccination in patients with AIIRD based on the currently available evidence and expert opinion were formulated. More research is needed, particularly regarding the incidence of vaccine-preventable infectious diseases and the safety of vaccination in patients with AIIRD.

Publication Type

Consensus Development Conference. Journal Article. Practice Guideline. Research Support, Non-U.S. Gov't. Systematic Review.

Year of Publication

2011

<582>

Unique Identifier

21075597

Title

Th17 cells can provide B cell help in autoantibody induced arthritis.

Source

Journal of Autoimmunity. 36(1):65-75, 2011 Feb.

VI 1

Status

MEDLINE

Authors

Hickman-Brecks CL; Racz JL; Meyer DM; LaBranche TP; Allen PM.

Authors Full Name

Hickman-Brecks, Cynthia L; Racz, Jennifer L; Meyer, Debra M; LaBranche, Timothy P; Allen, Paul M.

Institution

Hickman-Brecks, Cynthia L. Department of Pathology and Immunology, Washington University, St. Louis, MO, USA.

Abstract

K/BxN mice develop a spontaneous destructive arthritis driven by T cell dependent anti-glucose-6-phosphate isomerase (GPI) antibody production. In this study, a modified version of the K/BxN model, the KRN-cell transfer model (KRN-CTM), was established to determine the contribution of Th17 cells in the development of chronic arthritis. The transfer of naive KRN T cells into B6.TCR.Calpha(-/-)H-2(b/g7) T cell deficient mice induced arthritis by day 10 of transfer. Arthritis progressively developed for a period of up to 14 days following T cell transfer, thereafter the disease severity declined, but did not resolve. Both IL-17A and IFNgamma were detected in the recovered T cells from the popliteal lymph nodes and ankles. The transfer of KRN Th17 polarized KRN CD4(+) T cells expressing IL-17A and IFNgamma induced arthritis in all B6.TCR.Calpha(-/-)H-2(b/g7) mice however the transfer of Th1 polarized KRN CD4(+) T cells expressing IFNgamma alone induced disease in only 2/3 of the mice and disease induction was delayed compared to Th17 transfers. Th17 polarized KRN/T-bet(-/-) cells induced arthritis in all mice and surprisingly, IFNgamma was produced demonstrating that T-bet expression is not critical for arthritis induction, regardless of the cytokine expression. Neutralization of IFNgamma in KRN Th17 transfers resulted in earlier onset of disease while the neutralization of IL-17A delayed disease development. Consistent with K/BxN mice, naive KRN T cell transfers and Th17 polarized KRN/T-bet(-/-) transfers induced anti-GPI IgG(1) dominant responses while KRN Th17 cells induced high levels of IgG(2b). These data demonstrate that Th17 cells can participate in the production of autoantibodies that can induce arthritis. Copyright © 2010 Elsevier Ltd. All rights reserved.

Publication Type

Journal Article. Research Support, N.I.H., Extramural. Research Support, Non-U.S. Gov't.

Year of Publication

2011

<583>

Unique Identifier

20668905

Title

Prevalence of reactivation of hepatitis B virus replication in rheumatoid arthritis patients.

Source

Modern Rheumatology. 21(1):16-23, 2011 Feb.

VI 1

Status

MEDLINE

Authors

Urata Y; Uesato R; Tanaka D; Kowatari K; Nitobe T; Nakamura Y; Motomura S.

Authors Full Name

Urata, Yukitomo; Uesato, Ryoko; Tanaka, Dai; Kowatari, Kenji; Nitobe, Taisuke; Nakamura, Yoshihide; Motomura, Shigeru.

Institution

Urata, Yukitomo. Department of Rheumatology, Seihoku Chuo Hospital, 41 Nunoyacho, Gosyogawara, 037-0053, Japan. yurata@dream.com

Abstract

Reactivation of hepatitis B involves the reappearance of active necroinflammatory liver disease after an inactive hepatitis B surface antigen (HBsAg) carrier state or resolved hepatitis B, occurring during or after immunosuppression therapy or chemotherapy. We prospectively investigated the reactivation rate for hepatitis B virus (HBV) DNA replication in cases of rheumatoid arthritis (RA) with resolved hepatitis B. HBV markers were evaluated in 428 RA patients. Patients with positive findings of HBsAg or HBV DNA at enrolment were excluded. The study population comprised 422 RA patients, with resolved hepatitis B diagnosed in 135 patients based on HBsAg-negative and antihepatitis B core antibody/antihepatitis B surface antibody-positive results. HBV DNA was measured every 3 months in this group, and if HBV DNA became positive after enrolment, measurement was repeated every month. HBV DNA became positive (>=3.64 log copies/mL) in 7 of 135 patients for 12 months. Use of biologic agents was significantly more frequent in patients who developed reactivation of HBV DNA replication (85.7%) than in patients who did not (36.0%, p = 0.008). Hazard ratios for use of biologic agents and etanercept were 10.9 (p = 0.008) and 6.9 (p = 0.001), respectively. RA patients with resolved hepatitis B need careful monitoring when receiving biologic agents, regardless of HBV DNA levels.

Publication Type

Journal Article.

Year of Publication

2011

<584>

Unique Identifier

20013267

Title

Anti-TNF therapies: a comprehensive analysis of adverse effects associated with immunosuppression.

Source

Rheumatology International. 31(3):327-37, 2011 Mar.

VI 1

Status

MEDLINE

Authors

Connor V.

Authors Full Name

Connor, Victoria.

Institution

Connor, Victoria. University of Liverpool, Liverpool, UK. md0u7301@liv.ac.uk

Abstract

Knowledge and understanding about the immunosuppressive properties of anti-TNF therapies and the adverse effects these causes have advanced over the last 10 years since the first of these drugs was approved. These drugs work by inhibiting tumour necrosis factor (TNF) in the body, which plays an essential role in the immune response to invading pathogens. Anti-TNF drugs have therapeutic value because high levels of TNF are thought to be part of the pathophysiology of many chronic inflammatory disorders such as rheumatoid arthritis and Crohn's disease. Anti-TNF drugs are usually well-tolerated, however, there have been reports of many potentially serious adverse effects. This article will comprehensively analyse these adverse effects; the incidence, symptoms and mechanisms will be discussed. In addition, the contraindications of this class of drugs will be explored and the detection and prevention methods that should be put in place by health care professionals who treat patients on these drugs will be described.

Publication Type

Journal Article.

Year of Publication

2011
